# Supplementary material for: Visible light-triggered selective C(sp2)-H/C(sp3)-H coupling of benzenes with aliphatic hydrocarbons
Source: Nat Commun. 2023 Oct 11;14:6366. doi: 10.1038/s41467-023-42191-9 (PMC10567795; doi:10.1038/s41467-023-42191-9)
Supplement: Supplementary file 1 — Supplementary Information [file 41467_2023_42191_MOESM1_ESM.pdf]

Supplementary Information

Visible light-triggered selective C(sp^2)-H/C(sp^3)-H coupling of benzenes with aliphatic hydrocarbons

Qian-Yu Li[1], Shiyan Cheng[1], Ziqi Ye[1], Tao Huang[1], Fuxing Yang[1], Yu-Mei Lin[1]* & Lei Gong[1,2]*

[1] Key Laboratory of Chemical Biology of Fujian Province, College of Chemistry and Chemical Engineering, Xiamen University, Xiamen, Fujian 361005, China.

[2] Innovation Laboratory for Sciences and Technologies of Energy Materials of Fujian Province (IKKEM), Xiamen 361005, China.

E-mail: linyum@xmu.edu.cn, gongl@xmu.edu.cn

Table of Contents

Supplementary Methods **S3**

1. General Information S3

2. Synthesis of Substrates S4

3. Photochemical C(sp^2)-H/C(sp^3)-H Coupling Reactions S13

   3.1 Optimization of Reaction Conditions S13

   3.2 Substrate Scope S14

   3.3 Unsuccessful Substrates S73

   3.4 A Scale-up Reaction S73

   3.5 Identification of Side Products S74

4. Mechanistic Investigations S76

   4.1 Radical Probing Experiments S76

      4.1.1 Investigation of Benzyl Radical S76

      4.1.2 Investigation of Chlorine Free Radical S77

   4.2 Light-Off/on Experiments S80

   4.3 XPS Spectroscopy S81

   4.4 UV/Vis-Absorption Spectroscopy S82

   4.5 Reaction Interfered with Competitive Nucleophiles S83

   4.6 Kinetic Isotope Effect (KIE) Experiments S84

   4.7 Probing Other Intermediates S84

5. X-Ray Diffraction S87

   5.1 Crystal Structure of Product **35** S87

   5.2 Crystal Structure of Product **38** S87

   5.3 Crystal Structure of Product **56** S89

6. Computational Studies S92

   6.1 General Information S92

   6.2 Hirshfeld Charge Analysis S92

7. ^{1}H and ^{13}C NMR Spectra S95

Supplementary References **S200**

Supplementary Methods

1. General Information

General Remarks

Substrates **1zh**–**1zm**, **1zn**, **1zo**, **1zp** and **1zr**–**1zu**, **2za**, **2zm**, **83** were synthesized according to the published procedures. [1-6] $FeCl_3 \cdot 6H_2O$ was purchased from Sigma-Aldrich (purity ≥ 99.99%). All others reagents were purchased from commercial suppliers (TCI, Sigma-Aldrich, Alfa Aesar, Macklin, Energy Chemical, Laajoo, Leyan, Bide Pharmatech, Adamas-beta® and *J&K* Scientific) and used without further purification. Synthesis of the substrates were carried out under an argon atmosphere with magnetic stirring unless stated otherwise. Visible-light photochemical reactions were performed in 10 mL Schlenk tubes at the indicated temperature under an argon atmosphere and under irradiation with a 50 W LED lamp (λ_{max} = 395 nm, λ_{max} = 410 nm, λ_{max} = 425 nm and λ_{max} = 455 nm; commercial supplier: Hong Chang Lighting Co. Ltd., website: http://hongchang-led.taobao.com) or a 40 W Kessil LED lamp (PR160L, λ_{max} = 370 nm; commercial supplier: Anhui Kemi Instrument Co. Ltd., website: http://www.ahkemi.com).

Purification Techniques

Flash column chromatography was performed with silica gel (300–400 mesh, pH = 6.7–7.0). Thin layer chromatography (TLC) were carried out on Leyan HPTLC Silica Gel 60 GF254, visualized with UV light (254 nm).

Analytical Techniques

^1H NMR and ^{13}C NMR spectra were recorded on a Bruker AM (500 MHz) spectrometer at ambient temperature. NMR standards were used as follows: $CDCl_3$ = 7.26 ppm (^1H NMR), 77.16 ppm (^{13}C NMR). IR spectra were recorded on a Nicolet Avatar 330 FT-IR spectrophotometer. High-resolution mass spectra were recorded on an Agilent 1290-G6545XT QTOF instrument using ESI technique. Power X-ray diffraction data were recorded on a XtaLAB Synergy four-circle diffractometer with monochromatic Cu Kα radiation (λ = 1.54184 Å) at 100 K, 99.9 K and a Gemini S Ultra Synergy four-circle diffractometer with monochromatic Cu Kα radiation (λ = 0.71073 Å) at 293 K. Emission spectra were recorded on a Hitachi F-7000. X-ray photoelectron spectroscopy (XPS) measurements were carried out with a Qtac-100 LEISS-XPS spectrometer with a hemispherical electron energy analyzer and a home-made reaction chamber. Monochromatic Al Kα X-ray source (1486.6 eV, anode operating at 300 W) was used as the excitation source. The energy analysis error of the measurement was ± 0.2 eV for both binding energy and Auger energy. The binding energies in all the spectra were calibrated according to C 1s peak at 284.6 eV.

2. Synthesis of Substrates

According to the published procedure, [1] compounds **1zh**–**1zm** were prepared according to **general procedure A**.

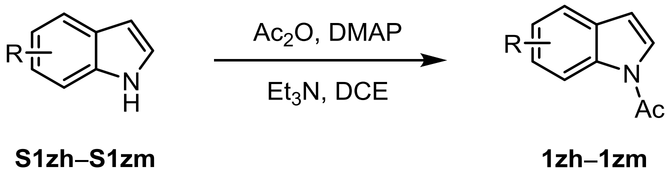

General procedure A: To a solution of indole derivative (**S1zh**–**S1zm**, 10.0 mmol) in 1,2-dichloroethane (DCE) (20 mL), acetic anhydride (40.0 mmol), Et_3N (30.0 mmol) and 4-dimethylaminopyridine (DMAP) (3.8 mmol) were added, and the mixture was stirred at room temperature for 24 h. After completion of the reaction as monitored by TLC, the residue was washed with a saturated solution of NH_4Cl (30 mL) and extracted with CH_2Cl_2 (3×20 mL). The combined organic extracts were died over Na_2SO_4 and concentrated under vacuum. Purification using silica gel column chromatography (elution with petroleum ether (PE):ethyl acetate (EtOAc) = 9:1) afforded the pure product (**1zh**–**1zm**).

1-(1*H*-indol-1-yl)ethan-1-one (1zh)

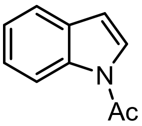

According to general procedure **A**, compound **1zh** was synthesized as a white solid (1.56 g, 9.80 mmol, 98% yield).

^{1}H NMR (500 MHz, $CDCl_3$) δ 8.13 – 8.09 (m, 1H), 8.01 – 7.98 (m, 1H), 7.88 – 7.83 (m, 1H), 7.38 – 7.34 (m, 1H), 6.95 – 6.88 (m, 1H), 6.57 – 6.54(m, 1H), 2.65 (s, 3H).

^{13}C NMR (126 MHz, $CDCl_3$) δ 167.1, 135.8, 128.6, 127.4, 123.8, 122.7, 121.9, 116.5, 110.3, 24.5.

IR (film): *ν* (cm^{-1}) 1746, 1510, 1487, 1452, 1440, 1330, 1246, 1233, 887.

HRMS (ESI-TOF, m/z) calculated for $C_{10}H_{10}NO^+$ $[M+H]^+$ 160.0757, found 160.0763.

Mp: 187.2–188.1 ℃

1-(4-fluoro-1*H*-indol-1-yl)ethan-1-one (1zi)

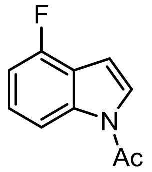

According to general procedure **A**, compound **1zi** was synthesized as a white solid (1.56 g, 8.80 mmol, 88% yield).

^{1}H NMR (500 MHz, $CDCl_3$) δ 7.91 – 7.86 (m, 1H), 7.71 – 7.65 (m, 1H), 7.06 – 6.99 (m, 1H), 6.98 – 6.96 (m, 1H), 6.59 – 6.54 (m, 1H), 2.65 (s, 3H).

^{13}C NMR (126 MHz, $CDCl_3$) δ 167.1, 156.5, 154.5, 136.8, 130.0, 124.5, 118.8, 111.8, 110.8, 24.5.

IR (film): *ν* (cm^{-1}) 1753, 1611, 1502, 1477, 13590, 1211, 1006, 887, 753, 746.

HRMS (ESI-TOF, m/z) calculated for $C_{10}H_8FNO^+$ $[M+H]^+$ 178.0663, found 178.0660.

Mp: 35.0–36.8 ℃

1-(4-chloro-1*H*-indol-1-yl)ethan-1-one (1zj)

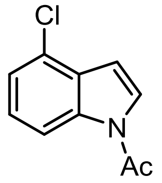

According to general procedure **A**, compound **1zj** was synthesized as a white solid (1.74 g, 8.98 mmol, 90% yield).

^{1}H NMR (500 MHz, $CDCl_3$) δ 8.04 – 7.95 (m, 1H), 7.86 – 7.77 (m, 1H), 7.00 (d, *J* = 10.0 Hz, 2H), 6.60 – 6.52 (m, 1H), 2.67 (s, 3H).

^{13}C NMR (126 MHz, $CDCl_3$) δ 167.2, 137.1, 130.1, 128.2, 126.8, 124.6, 121.6, 114.9, 112.3, 24.5.

IR (film): *ν* (cm^{-1}) 1679, 1614, 1432, 1415, 1344, 1070, 1017, 894, 781, 627.

HRMS (ESI-TOF, m/z) calculated for $C_{10}H_9ClNO^+$ $[M+H]^+$ 194.0367, found 194.0361.

Mp: 33.8–35.1 ℃

1-(4-bromo-1*H*-indol-1-yl)ethan-1-one (1zk)

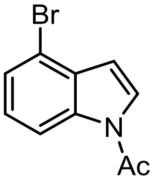

According to general procedure **A**, compound **1zk** was synthesized as a white solid (2.02 g, 8.48 mmol, 85% yield).

^{1}H NMR (500 MHz, $CDCl_3$) δ 8.07 – 8.02 (m, 1H), 7.85 – 7.80 (m, 1H), 7.32 – 7.25 (m, 1H), 6.97 – 6.90 (m, 1H), 6.59 – 6.52 (m, 1H), 2.65 (s, 3H).

^{13}C NMR (126 MHz, $CDCl_3$) δ 167.1, 138.8, 129.5, 128.5, 128.3, 123.2, 116.8, 115.6, 109.0, 24.5.

IR (film): *v* (cm^{-1}) 1749, 1502, 1460, 1423, 1417, 1311, 1238, 1116, 751.

HRMS (ESI-TOF, m/z) calculated for $C_{10}H_9BrNO^+$ $[M+H]^+$ 237.9862, found 237.9869.

Mp: 36.5–37.3 ℃

1-(4-methyl-1*H*-indol-1-yl)ethan-1-one (1zl)

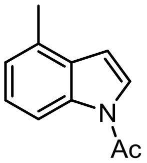

According to general procedure **A**, compound **1zl** was synthesized as a white solid (1.61 g, 9.29 mmol, 93% yield).

^{1}H NMR (500 MHz, $CDCl_3$) δ 8.02 – 7.98 (m, 1H), 7.78 – 7.76 (m, 1H), 7.02 – 6.98 (m, 1H), 6.96 – 6.92 (m, 1H), 6.57 – 6.54 (m, 1H), 2.65 (s, 3H), 2.58 (s, 3H).

^{13}C NMR (126 MHz, $CDCl_3$) δ 167.1, 136.3, 136.1, 125.2, 125.0, 122. 5, 122.4, 113.7, 106.1, 24.5, 20.3.

IR (film): *v* (cm^{-1}) 1746, 1584, 1501, 1461, 1433, 1440, 1428, 1355, 1246, 1238, 892, 781, 721.

HRMS (ESI-TOF, m/z) calculated for $C_{11}H_{12}NO^+$ $[M+H]^+$ 174.0913, found 174.0909.

Mp: 66.2–43.7 ℃

1-(5-bromo-1*H*-indol-1-yl)ethan-1-one (1zm)

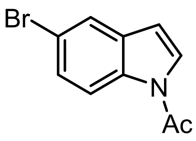

According to general procedure **A**, compound **1zm** was synthesized as a white solid (2.00 g, 8.47 mmol, 85% yield).

^{1}H NMR (500 MHz, $CDCl_3$) δ 7.88 (d, *J* = 15.5 Hz, 2H), 7.41 (d, *J* = 5.0 Hz, 2H), 6.58 – 6.54 (m, 1H), 2.65 (s, 3H).

^{13}C NMR (126 MHz, $CDCl_3$) δ 167.1, 137.4, 127.8, 127.3, 125.8, 124.7, 122.1, 119.0, 111.1, 24.5.

IR (film): *v* (cm^{-1}) 1750, 1500, 1471, 1423, 1409, 1321, 1307, 1233, 1118, 776, 749.

HRMS (ESI-TOF, m/z) calculated for $C_{10}H_9BrNO^+$ $[M+H]^+$ 237.9862, found 237.9856.

Mp: 36.5–37.3 ℃

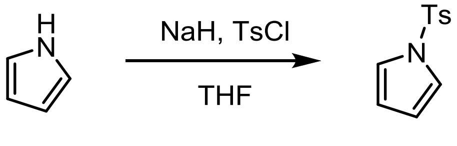

According to the published procedure, [2] compound **1zn** was synthesized. To a solution of pyrrole (**S1zn**, 10.0 mmol) in THF (15 mL), NaH (15.0 mmol) was added. The suspension was stirred for 30 min, then tosyl chloride (12.0 mmol) in THF (5.0 mL) was added. The mixture was stirred for additional 3 h, then quenched with water (30 mL), and extracted with EtOAc (3×20 mL). The combined organic layers were dried over Na_2SO_4 and concentrated under reduced pressure. Purification using silica gel column chromatography (elution with PE:EtOAc = 2:1) afforded the pure product **1zn** as a white solid (1.81 g, 8.18 mmol, 82% yield) .

1-tosyl-1*H*-pyrrole (1zn)

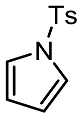

^{1}H NMR (500 MHz, $CDCl_3$) δ 7.78 – 7.72 (m, 2H), 7.62 – 7.54 (m, 2H), 7.47 – 7.41 (m, 2H), 6.28 – 6.22 (m, 2H), 2.43 (s, 3H).

^{13}C NMR (126 MHz, $CDCl_3$) δ 144.3, 136.9, 129.7, 127.2, 127.2, 101.7, 21.2.

IR (film): ν (cm^{-1}) 1596, 1455, 1364, 1187, 1168, 1057, 734, 673.

HRMS (ESI-TOF, m/z) calculated for $C_{11}H_{12}NO_2S^+$ $[M+H]^+$ 222.0583, found 222.0584.

Mp: 96.3–97.6 ℃

According to the published procedure, [3] compounds **1zo**, **1zp** and **1zr**–**1zu** were prepared according to **general procedure B**.

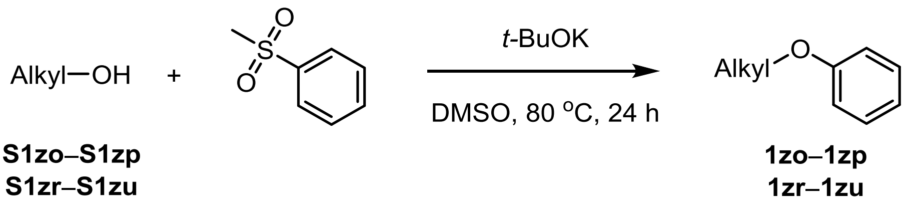

General procedure B: A Schlenk tube was added *t*-BuOK (4.0 mmol), (methylsulfonyl)benzene (2.0 mmol) under an argon atmosphere in a glove box. DMSO (2.0 mL)and alkyl alcohol (**S1zo**–**S1zp**, **S1zr**–**S1zu**, 4.0 mmol) were added *via* syringe under an argon atmosphere. The reaction mixture was heated to 80 ℃ in an oil bath and stirred for 24 h. Upon completion of the reaction, the sealed vial was cooled to room temperature. H_2O (20 mL) was added to quench the reaction. The mixture was extracted with dichloromethane (DCM) (3×10 mL). The combined organic layers were dried over Na_2SO_4 and concentrated under reduced pressure. Purification using silica gel column chromatography (elution with hexane) afforded the compounds (**1zo**–**1zp** and **1zr**–**1zu**).

2-(phenoxymethyl)tetrahydrofuran (1zo)

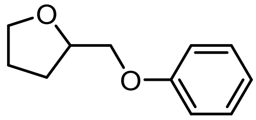

According to **general procedure B**, compound **1zo** was synthesized as a colorless oil (0.32 g, 1.80 mmol, 90% yield).

^{1}H NMR (500 MHz, $CDCl_3$) δ 7.53 – 7.22 (m, 2H), 6.97 – 6.88 (m, 3H), 4.35 – 4.21 (m, 1H), 4.15 – 3.94 (m, 2H), 3.91 – 3.74 (m, 2H), 2.05 – 1.86 (m, 1H), 1.75 – 1.60 (m, 3H).

^{13}C NMR (126 MHz, $CDCl_3$) δ 159.2, 129.8, 121.5, 115.8, 78.3, 70.3, 69.2, 30.3, 25.6.

1-(phenoxymethyl)adamantane (1zp)

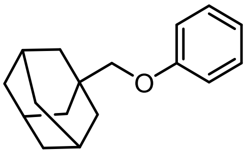

According to **general procedure B**, compound **1zp** was synthesized as a colorless oil (0.45 g, 1.86 mmol, 92% yield).

^{1}H NMR (500 MHz, $CDCl_3$) δ 7.51 – 7.23 (m, 2H), 6.97 – 6.88 (m, 3H), 3.66 (s, 2H), 2.04 – 1.95 (m, 3H), 1.74 (d, *J* = 33.0 Hz, 6H), 1.70 (t, 6H).

^{13}C NMR (126 MHz, $CDCl_3$) δ 159.1, 129.5, 121.9, 115.9, 80.3, 39.3, 37.4, 33.2, 29.6.

(((1*S*,2*R*,5*R*)-5-isopropyl-2-methylcyclohexyl)oxy)benzene (1zr)

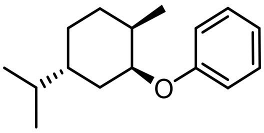

According to **general procedure B**, compound **1zr** was synthesized as a colorless oil (0.38 g, 1.64 mmol, 82% yield).

^{1}H NMR (500 MHz, $CDCl_3$) δ 7.28 – 7.23 (m, 2H), 6.92 – 6.85 (m, 3H), 4.05 – 3.99 (m, 1H), 2.26 – 2.14 (m, 2H), 1.73 – 1.70 (m, 2H), 1.53 – 1.44 (m, 2H), 1.14 – 0.99 (m, 2H), 0.93 – 0.89 (m, 7H), 0.77 (d, *J* = 7.0 Hz, 3H)

^{13}C NMR (126 MHz, $CDCl_3$) δ 158.4, 129.5, 120.4, 115.9, 48.1, 40.4, 34.6, 31.5, 26.1, 23.8, 22.2, 20.8, 16.6.

(1*R*,2*R*,4*S*)-1,3,3-trimethyl-2-phenoxybicyclo[2.2.1]heptane (1zs)

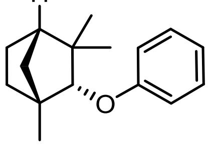

According to **general procedure B**, compound **1zs** was synthesized as a colorless oil (0.39 g, 1.69 mmol, 85% yield).

^{1}H NMR (500 MHz, $CDCl_3$) δ 7.26 – 7.21 (m, 2H), 6.91 – 6.86 (m, 3H), 3.86 (d, *J* = 1.5 Hz, 1H), 2.05 – 2.02 (m, 1H), 1.77 – 1.72 (m, 2H), 1.60 – 1.47 (m, 1H), 1.51 – 1.43 (m, 1H), 1.21 – 1.17 (m, 4H), 1.12 – 1.05 (m, 4H), 0.86 (s, 3H).

^{13}**C NMR** (126 MHz, CDCl$_3$) δ 160.3, 129.3, 120.1, 115.8, 90.0, 49.5, 49.1, 41.4, 40.0, 30.5, 26.4, 25.9, 20.4, 19.9.

(1*R*,2*S*,4*R*)-1,3,7,7-tetramethyl-2-phenoxybicyclo[2.2.1]heptane (1zt)

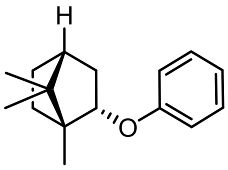

According to **general procedure B**, compound **1zt** was synthesized as a colorless oil (0.32 g, 1.39 mmol, 70% yield).

^{1}**H NMR** (500 MHz, CDCl$_3$) δ 7.31 (t, *J* = 8.0 Hz, 2H), 6.98 – 6.84 (m, 3H), 4.40 – 4.35 (m, 1H), 2.42 (td, *J* = 9.3, 4.6 Hz, 1H), 2.30 (dt, *J* = 7.6, 4.7 Hz, 1H), 1.85 – 1.78 (m, 2H), 1.43 – 1.37 (m, 1H), 1.33 – 1.29 (m, 1H), 1.18 (dd, *J* = 13.3, 3.3 Hz, 1H), 1.00 (s, 3H), 0.97 (d, *J* = 5.0 Hz, 6H).

^{13}**C NMR** (126 MHz, CDCl$_3$) δ 159.2, 129.4, 120.1, 115.5, 82.7, 49.5, 47.6, 45.2, 36.9, 28.0, 26.8, 19.8, 19.0, 13.8.

(3*R*,5*R*,8*S*,9*R*,10*R*,13*R*,14*R*)-10,13-dimethyl-3-phenoxyhexadecahydro-17*H*-cyclopenta[*a*]phenanthren-17-one (1zu)

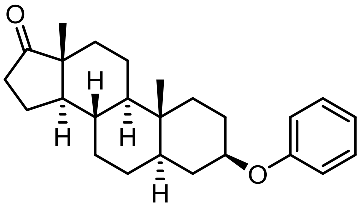

According to **general procedure B**, compound **1zu** was synthesized as a colorless oil (0.44 g, 1.20 mmol, 60% yield).

^{1}**H NMR** (500 MHz, CDCl$_3$) δ 7.28 – 7.24 (m, 2H), 7.01 – 6.86 (m, 3H), 3.66 – 3.52 (m, 1H), 2.37 – 2.12 (m, 2H), 1.94 – 1.77 (m, 4H), 1.72 – 1.46 (m, 6H), 1.45 – 1.26 (m, 5H), 1.23 – 1.13 (m, 2H), 1.11 – 0.99 (m, 2H), 0.86 (s, 3H), 0.82 (s, 3H), 0.80 – 0.68 (m, 1H).

^{13}**C NMR** (126 MHz, CDCl$_3$) δ 219.9, 157.5, 130.1, 121.7, 117.1, 78.2, 54.4, 52.1, 48.6, 44.7, 37.7, 36.3, 36.2, 36.0, 33.6, 33.0, 31.2, 28.3, 26.3, 22.1, 22.0, 14.6, 13.4.

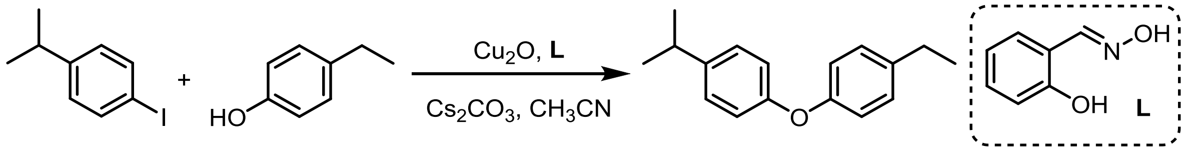

2za

According to the published procedure, [4] compound **2za** was synthesized. To a solution of Cu_2O (2.0 mmol) and ligand (2.0 mmol) in DMF (6.0 mL), 4-ethylphenol (10.0 mmol), 1-iodo-4-isopropylbenzene (15.0 mmol) and cesium carbonate (20.0 mmol) were added at 110 ℃ for 30 h. Purification using silica gel column chromatography (elution with hexane) afforded the compound **2z** as a colorless oil (1.56 g, 6.50 mmol, 65% yield).

1-ethyl-4-(4-isopropylphenoxy)benzene (2za)

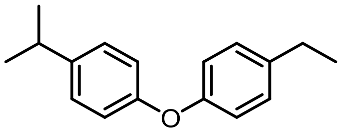

^{1}H NMR (500 MHz, $CDCl_3$) δ 7.58 – 7.33 (m, 5H), 7.32 – 7.30 (m, 1H), 7.17 – 7.11 (m, 2H), 2.95 – 2.80 (m, 1H), 2.72 (q, 2H), 1.21 (s, 3H), 1.19 (s, 3H), 1.18 (t, 3H).

^{13}C NMR (126 MHz, $CDCl_3$) δ 154.5, 153.6, 142.5, 139.6, 129.2, 128.5, 120.1, 119.4, 34.0, 27.8, 23.4, 13.2.

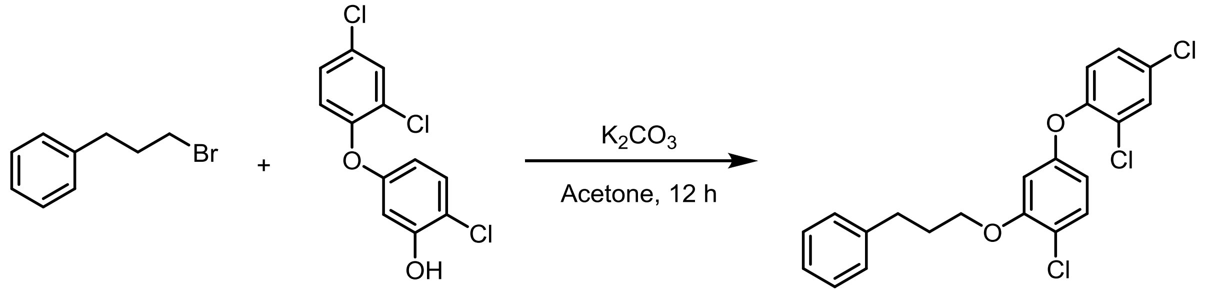

2zm

According to the published procedure, [5] compound **2zm** was synthesized. The mixture of triclosan (7.5 mmol), (3-bromopropyl)benzene (5.0 mmol) and K_2CO_3 (10.0 mmol) in acetone (20 mL) was heated to reflux. Upon completion stirring for 12 h, the reaction mixture was cooled to room temperature and filtered. Purification using silica gel column chromatography (elution with PE:EtOAc = 10:1) afforded the compound **2zm** as a colorless oil (1.46 g, 3.60 mmol, 72% yield).

2,4-dichloro-1-(4-chloro-2-(3-phenylpropoxy)phenoxy)benzene (2zm)

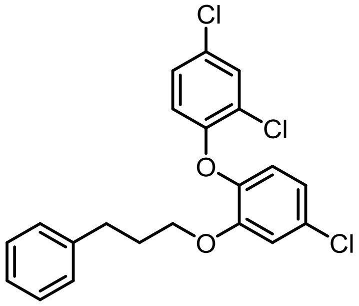

^{1}H NMR (500 MHz, $CDCl_3$) δ 7.43 (d, *J* = 2.5 Hz, 1H), 7.27 – 7.24 (m, 2H), 7.19 – 7.16 (m, 1H), 7.10 – 7.06 (m, 3H), 7.00 – 6.97 (m, 1H), 6.93 – 6.91 (m, 2H), 6.65 (d, *J* = 8.8 Hz, 1H), 3.89 (d, *J* = 6.1 Hz, 2H), 2.55 (dd, *J* = 8.4, 6.7 Hz, 2H), 1.95 – 1.91 (m, 2H).

^{13}C NMR (126 MHz, $CDCl_3$) δ 152.2, 148. 6, 145.1, 142.0, 131.3, 130.5, 129.9, 128.5, 128.4, 128.4, 126.2, 124.2, 123.4, 120.5, 117.4, 116.1, 69.2, 32.7, 30.2.

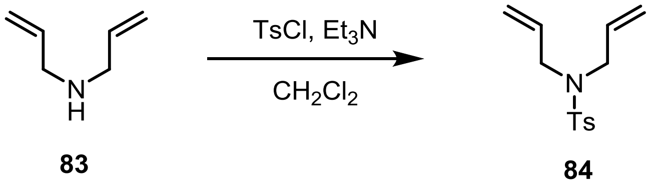

According to the published procedure, [6] compound **84** was synthesized. In a round-bottom flask on ice, triethylamine (12.0 mmol) was added dropwise to a solution of diallylamine (**83**, 12.0 mmol) in DCM (20 mL). The reaction was stirred at 0 ℃ for 30 min and 4-toluenesulfonyl chloride (10.0 mmol) was added slowly to the reaction. The reaction was slowly warmed to room temperature and stirred overnight. The reaction was washed with water and brine. The organic layer was dried over sodium sulfate and concentrated under reduced pressure afforded the compound **84** as a colorless oil (2.05 g, 8.20 mmol, 68% yield).

***N,N*-diallyl-4-methylbenzenesulfonamide (84)**

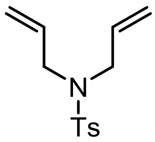

^{1}H NMR (500 MHz, $CDCl_3$) δ 7.70 (d, *J* = 8.3 Hz, 2H), 7.29 (d, *J* = 8.1 Hz, 2H), 5.64 – 5.56 (m, 2H), 5.15 (dd, *J* = 2.5, 1.2 Hz, 2H), 5.14 – 5.11 (m, 2H), 3.80 (d, *J* = 6.3 Hz, 4H), 2.42 (s, 3H).

^{13}C NMR (126 MHz, $CDCl_3$) δ 143.0, 137.3, 132.5, 129.5, 126.9, 118.7, 49.2, 21.2.

3. Photochemical C(sp^2)-H/C(sp^3)-H Coupling Reactions

3.1 Optimization of Reaction Conditions

A Schlenk tube (10 mL) was charged with anisole (**1a**, 32.4 mg, 0.30 mmol), 4-ethylbiphenyl (**2a**, 164.0 mg, 0.90 mmol), $FeCl_3$ $6H_2O$ (16.2 mg, 0.060 mmol) and $(CHCl_2)_2$ (0.75 mL). The mixture was degassed *via* three freeze-pump-thaw cycles, then filled with dry air. The Schlenk tube was positioned approximately 5 cm away from a 50 W LED lamp (λ_{max} = 410 nm). After being stirred at 25 ℃ for 48 h, the reaction mixture was concentrated to dryness. Purification using silica gel column chromatography (elution with petroleum ether (PE): dichloromethane (DCM) = 5:1) gave the pure product.

Supplementary Table 1. Investigation of other conditions. [a]

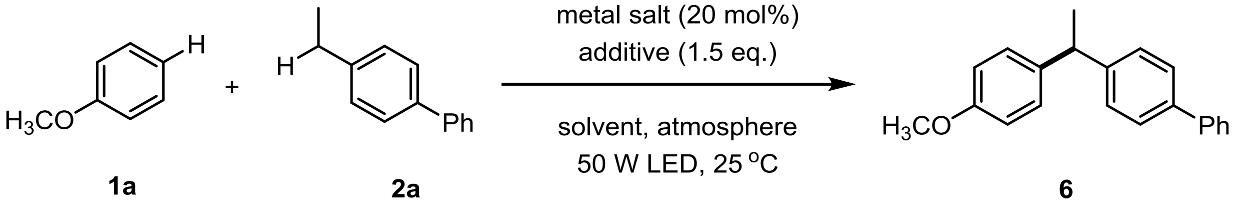

Entry	Metal salt	Atmosphere	Additive	Solvent	Light source (nm)	Yield (%) [b]
1	$FeCl_3$ $6H_2O$	Ar	-	$(CHCl_2)_2$	410	24
2	$FeCl_3$ $6H_2O$	dry air	-	$(CHCl_2)_2$	370	37
3	$FeCl_3$ $6H_2O$	dry air	-	$(CHCl_2)_2$	425	60
4	$FeCl_3$ $6H_2O$	dry air	-	$(CHCl_2)_2$	440	33
5	$FeCl_3$ $6H_2O$	dry air	H_2O	$(CHCl_2)_2$	410	22
6	$FeCl_3$ $6H_2O$	dry air	K_2CO_3	$(CHCl_2)_2$	410	trace
7	$FeCl_3$ $6H_2O$	dry air	2,2'-bipyridine	$(CHCl_2)_2$	410	0
8	$FeCl_3$ $6H_2O$	dry air	HCl in H_2O (1.0 M)	$(CHCl_2)_2$	410	trace
9	$FeCl_3$ $6H_2O$	dry air	HCl in Et_2O (1.0 M)	$(CHCl_2)_2$	410	33
10	$FeCl_3$ $6H_2O$	dry air	AcOH	$(CHCl_2)_2$	410	trace
11	$FeCl_3$ $6H_2O$	dry air	CF_3COOH	$(CHCl_2)_2$	410	37
12	$FeCl_3$ $6H_2O$	dry air	$ZnCl_2$	$(CHCl_2)_2$	410	73
13	$FeCl_3$ $6H_2O$	dry air	$Cu(OTf)_2$	$(CHCl_2)_2$	410	60
14 [c]	$CuCl_2$	N_2	LiCl	MeCN	390	0

[a] Reaction conditions: **1a** (0.30 mmol), **2a** (0.90 mmol), metal salt (0.060 mmol), indicated additive (0.45 mmol), solvent (0.75 mL), under indicated atmosphere, irradiation with a 50 W

LED lamp, 25 ℃, 48 h. [b] Isolated yield. [c] LiCl as additive (0.15 mmol), 60 ℃.

3.2 Substrate Scope

4-(1-(4-methoxyphenyl)ethyl)-1,1'-biphenyl (3)

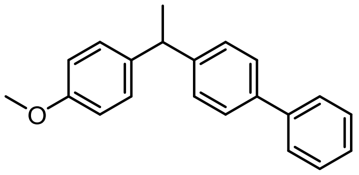

A Schlenk tube (10 mL) was charged with anisole (**1a**, 32.4 mg, 0.30 mmol), 4-ethylbiphenyl (**2a**, 164.0 mg, 0.90 mmol), $FeCl_3$ $6H_2O$ (16.2 mg, 0.060 mmol) and $(CHCl_2)_2$ (0.75 mL). The mixture was degassed *via* three freeze-pump-thaw cycles, then filled with dry air. The Schlenk tube was positioned approximately 5 cm away from a 50 W LED lamp (λ_{max} = 410 nm). After being stirred at 25 ℃ for 48 h, the reaction mixture was concentrated to dryness. Purification using silica gel column chromatography (elution with PE:DCM = 5:1) gave the pure product **3** as a white solid (65.7 mg, 0.228 mmol, 76% yield).

^{1}H NMR (500 MHz, $CDCl_3$) δ 7.63 – 7.61 (m, 2H), 7.56 (d, *J* = 8.3 Hz, 2H), 7.46 (t, *J* = 7.6 Hz, 2H), 7.39 – 7.36 (m, 1H), 7.33 (d, *J* = 8.1 Hz, 2H), 7.23 (d, *J* = 8.4 Hz, 2H), 6.92 – 6.89 (m, 2H), 4.20 (q, *J* = 7.2 Hz, 1H), 3.82 (s, 3H), 1.70 (d, *J* = 7.3 Hz, 3H).

^{13}C NMR (126 MHz, $CDCl_3$) δ 158.0, 146.0, 141.1, 139.0, 138.6, 128.8, 128.7, 128.1, 127.2, 127.2, 127.1, 113.9, 55.4, 43.8, 22.2.

IR (film): *v* (cm^{-1}) 3500, 2964, 2813, 1867, 1661, 1536, 1407, 1251, 1078, 831, 621.

HRMS (ESI-TOF, m/z) calculated for $C_{21}H_{21}O^+$ $[M+H]^+$ 289.1587, found 289.1600.

4-(1-(4-ethoxyphenyl)ethyl)-1,1'-biphenyl (4)

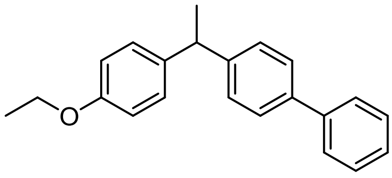

A Schlenk tube (10 mL) was charged with ethoxybenzene (**1b**, 36.6 mg, 0.30 mmol), 4-ethylbiphenyl (**2a**, 164.0 mg, 0.90 mmol), $FeCl_3$ $6H_2O$ (16.2 mg, 0.060 mmol) and $(CHCl_2)_2$ (0.75 mL). The mixture was degassed *via* three freeze-pump-thaw cycles, then filled with dry air. The Schlenk tube was positioned approximately 5 cm away from a 50 W LED lamp (λ_{max} = 410 nm). After being stirred at 25 ℃ for 48 h, the reaction mixture was concentrated to dryness. Purification using silica gel column chromatography (elution with PE:DCM = 5:1) gave the pure product **4** as a colorless oil (67.1 mg, 0.207 mmol, 74% yield).

^{1}**H NMR** (500 MHz, $CDCl_3$) δ 7.62 – 7.60 (m, 2H), 7.57 – 7.54 (m, 2H), 7.46 (t, *J* = 7.7 Hz, 2H), 7.38 – 7.35 (m, 1H), 7.33 (d, *J* = 8.2 Hz, 2H), 7.21 (d, *J* = 8.6 Hz, 2H), 6.90 – 6.87 (m, 2H), 4.19 (q, *J* = 7.2 Hz, 1H), 4.05 (q, *J* = 7.0 Hz, 2H), 1.69 (d, *J* = 7.2 Hz, 3H), 1.44 (t, *J* = 7.0 Hz, 3H).

^{13}**C NMR** (126 MHz, $CDCl_3$) δ 157.4, 146.1, 141.2, 139.0, 138.4, 128.8, 128.6, 128.1, 127.2, 127.1, 127.1, 114.5, 63.5, 43.8, 22.2, 15.0.

IR (film): *v* (cm^{-1}) 3505, 2942, 2776, 2531, 1665, 1573, 1407, 1150, 1078, 895, 569.

HRMS (ESI-TOF, m/z) calculated for $C_{22}H_{23}O^+$ $[M+H]^+$ 325.1563, found 325.1570.

4-(1-(4-(benzyloxy)phenyl)ethyl)-1, 1'-biphenyl (5)

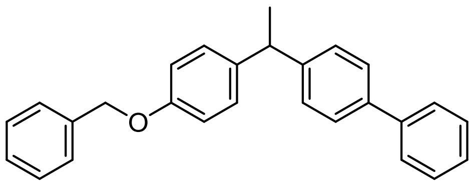

A Schlenk tube (10 mL) was charged with (benzyloxy)benzene (**1c**, 55.2 mg, 0.30 mmol), 4-ethylbiphenyl (**2a**, 164.0 mg, 0.90 mmol), $FeCl_3 \cdot 6H_2O$ (16.2 mg, 0.060 mmol) and $(CHCl_2)_2$ (0.75 mL). The mixture was degassed *via* three freeze-pump-thaw cycles, then filled with dry air. The Schlenk tube was positioned approximately 5 cm away from a 50 W LED lamp (λ_{max} = 410 nm). After being stirred at 25 ℃ for 48 h, the reaction mixture was concentrated to dryness. Purification using silica gel column chromatography (elution with PE:DCM = 5:1) gave the pure product **5** as a colorless oil (60.1 mg, 0.165 mmol, 55% yield).

^{1}**H NMR** (500 MHz, $CDCl_3$) δ 7.59 (d, *J* = 7.6 Hz, 2H), 7.53 (d, *J* = 8.1 Hz, 2H), 7.42 (m, 6H), 7.32 (m, 4H), 7.20 (d, *J* = 8.6 Hz, 2H), 6.95 (d, *J* = 8.6 Hz, 2H), 5.06 (s, 2H), 4.17 (q, *J* = 7.2 Hz, 1H), 1.67 (d, *J* = 7.2 Hz, 3H).

^{13}**C NMR** (126 MHz, $CDCl_3$) δ 157.3, 146.0, 141.2, 139.0, 138.9, 137.3, 128.8, 128.7, 128.7, 128.1, 128.1, 127.6, 127.2, 127.2, 127.2, 114.9, 70.2, 43.8, 22.2.

IR (film): *v* (cm^{-1}) 3500, 3337, 2927, 1705, 1669, 1506, 1363, 1053, 825, 706, 672.

HRMS (ESI-TOF, m/z) calculated for $C_{27}H_{25}O^+$ $[M+H]^+$ 365.1900, found 365.1907.

4-(1-(4-phenoxyphenyl)ethyl)-1,1'-biphenyl (6)

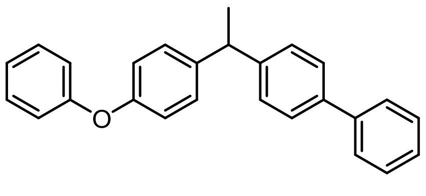

A Schlenk tube (10 mL) was charged with diphenyl ether (**1d**, 51.1 mg, 0.30 mmol),

4-ethylbiphenyl (**2a**, 164.0 mg, 0.90 mmol), $FeCl_3 \cdot 6H_2O$ (16.2 mg, 0.060 mmol) and $(CHCl_2)_2$ (0.75 mL). The mixture was degassed *via* three freeze-pump-thaw cycles, then filled with dry air. The Schlenk tube was positioned approximately 5 cm away from a 50 W LED lamp (λ_{max} = 410 nm). After being stirred at 25 ℃ for 48 h, the reaction mixture was concentrated to dryness. Purification using silica gel column chromatography (elution with PE:DCM = 5:1) gave the pure product **6** as a white solid (78.8 mg, 0.225 mmol, 75% yield).

^{1}H NMR (500 MHz, $CDCl_3$) δ 7.60 – 7.58 (m, 2H), 7.54 (d, *J* = 8.2 Hz, 2H), 7.44 (t, *J* = 7.7 Hz, 2H), 7.36 – 7.30 (m, 5H), 7.23 (d, *J* = 8.6 Hz, 2H), 7.09 (t, *J* = 7.4 Hz, 1H), 7.04 – 6.95 (m, 4H), 4.20 (q, *J* = 7.2 Hz, 1H), 1.69 (d, *J* = 7.2 Hz, 3H).

^{13}C NMR (126 MHz, $CDCl_3$) δ 157.6, 155.5, 145.7, 141.3, 141.1, 139.1, 129.8, 129.0, 128.9, 128.1, 127.3, 127.2, 127.2, 123.2, 119.0, 118.9, 44.0, 22.2.

IR (film): ν (cm^{-1}) 3488, 3407, 2844, 1705, 1683, 1546, 1363, 1235, 1053, 825, 672.

HRMS (ESI-TOF, m/z) calculated for $C_{26}H_{23}O^+$ $[M+H]^+$ 351.1743, found 351.1749.

4-(1-(3-bromo-4-methoxyphenyl)ethyl)-1,1'-biphenyl (7)

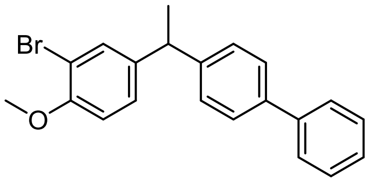

A Schlenk tube (10 mL) was charged with 2-bromoanisole (**1e**, 56.1 mg, 0.30 mmol), 4-ethylbiphenyl (**2a**, 164.0 mg, 0.90 mmol), $FeCl_3 \cdot 6H_2O$ (16.2 mg, 0.060 mmol) and $(CHCl_2)_2$ (0.75 mL). The mixture was degassed *via* three freeze-pump-thaw cycles, then filled with dry air. The Schlenk tube was positioned approximately 5 cm away from a 50 W LED lamp (λ_{max} = 410 nm). After being stirred at 25 ℃ for 48 h, the reaction mixture was concentrated to dryness. Purification using silica gel column chromatography (elution with PE:DCM = 5:1) gave the pure product **7** as a white solid (74.7 mg, 0.204 mmol, 68% yield).

^{1}H NMR (500 MHz, $CDCl_3$) δ 7.60 – 7.57 (m, 2H), 7.55 – 7.52 (m, 2H), 7.46 (d, *J* = 2.2 Hz, 1H), 7.44 (t, *J* = 7.7 Hz, 2H), 7.34 (t, *J* = 7.4 Hz, 1H), 7.28 (d, *J* = 8.2 Hz, 2H), 7.16 (m, 1H), 6.85 (d, *J* = 8.5 Hz, 1H), 4.13 (q, *J* = 7.2 Hz, 1H), 3.88 (s, 3H), 1.65 (d, *J* = 7.2 Hz, 3H).

^{13}C NMR (126 MHz, $CDCl_3$) δ 154.3, 145.2, 141.0, 140.2, 139.3, 132.5, 128.9, 128.0, 127.7, 127.3, 127.2, 127.1, 112.0, 111.7, 56.4, 43.5, 22.1.

IR (film): ν (cm^{-1}) 3500, 3337, 3147, 2927, 1681, 1669, 1506, 1363, 1323, 1053, 825, 706, 672, 493.

HRMS (ESI-TOF, m/z) calculated for $C_{21}H_{19}BrNaO^+$ $[M+Na]^+$ 389.0511, found 389.0517.

4-(1-(2-bromo-4-methoxyphenyl)ethyl)-1,1'-biphenyl (8)

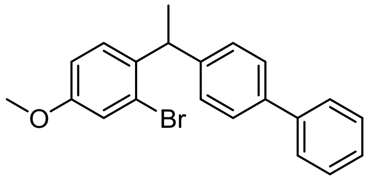

A Schlenk tube (10 mL) was charged with 3-bromoanisole (**1f**, 56.1 mg, 0.30 mmol), 4-ethylbiphenyl (**2a**, 164.0 mg, 0.90 mmol), $FeCl_3 \cdot 6H_2O$ (16.2 mg, 0.060 mmol) and $(CHCl_2)_2$ (0.75 mL). The mixture was degassed *via* three freeze-pump-thaw cycles, then filled with dry air. The Schlenk tube was positioned approximately 5 cm away from a 50 W LED lamp (λ_{max} = 410 nm). After being stirred at 25 ℃ for 48 h, the reaction mixture was concentrated to dryness. Purification using silica gel column chromatography (elution with PE:DCM = 5:1) gave the pure product **8** as a colorless oil (68.1 mg, 0.186 mmol, 62% yield).

^{1}H NMR (500 MHz, $CDCl_3$) δ 7.65 – 7.62 (m, 2H), 7.58 (d, *J* = 8.2 Hz, 2H), 7.47 (t, *J* = 7.7 Hz, 2H), 7.37 (m, 3H), 7.22 – 7.19 (m, 2H), 6.89 (m, 1H), 4.68 (q, *J* = 7.2 Hz, 1H), 3.81 (s, 3H), 1.68 (d, *J* = 7.2 Hz, 3H).

^{13}C NMR (126 MHz, $CDCl_3$) δ 158.4, 144.6, 141.1, 139.0, 137.4, 129.2, 128.8, 128.2, 127.2, 127.1, 127.1, 124.9, 118.0, 114.0, 55.6, 42.5, 21.6.

IR (film): *ν* (cm^{-1}) 3500, 3337, 3147, 2927, 1681, 1669, 1506, 1480, 1323, 1053, 988, 735, 672, 467.

HRMS (ESI-TOF, m/z) calculated for $C_{21}H_{19}BrNaO^+$ $[M+Na]^+$ 389.0511, found 389.0521.

4-(1-(3,4-dimethoxyphenyl)ethyl)-1,1'-biphenyl (9)

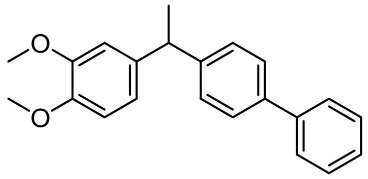

A Schlenk tube (10 mL) was charged with 1,2-dimethoxybenzene (**1g**, 41.4 mg, 0.30 mmol), 4-ethylbiphenyl (**2a**, 164.0 mg, 0.90 mmol), $FeCl_3 \cdot 6H_2O$ (16.2 mg, 0.060 mmol) and $(CHCl_2)_2$ (0.75 mL). The mixture was degassed *via* three freeze-pump-thaw cycles, then filled with dry air. The Schlenk tube was positioned approximately 5 cm away from a 50 W LED lamp (λ_{max} = 410 nm). After being stirred at 25 ℃ for 48 h, the reaction mixture was concentrated to dryness. Purification using silica gel column chromatography (elution with PE:DCM = 3:1) gave the pure product **9** as a white solid (66.8 mg, 0.210 mmol, 70% yield).

^{1}H NMR (500 MHz, $CDCl_3$) δ 7.61 – 7.56 (m, 2H), 7.54 (d, *J* = 8.2 Hz, 2H), 7.43 (t, *J* = 7.7 Hz, 2H), 7.34 (d, *J* = 7.2 Hz, 1H), 7.31 (d, *J* = 8.2 Hz, 2H), 6.82 (d, *J* = 11.5 Hz, 2H), 6.78 (d, *J* = 5.0 Hz, 1H), 4.16 (q, *J* = 7.2 Hz, 1H), 3.87 (s, 3H), 3.85 (s, 3H), 1.69 (d, *J* = 7.2 Hz, 3H).

^{13}C NMR (126 MHz, $CDCl_3$) δ 149.0, 147.5, 145.9, 141.1, 139.0, 139.0, 128.8, 128.0, 127.2, 127.2, 127.1, 119.5, 111.4, 111.2, 56.0, 56.0, 44.2, 22.2.

IR (film): *v* (cm^{-1}) 3507, 2972, 2013, 1867, 1661, 1431, 1407, 1326, 1251, 1078, 831, 621, 597, 550.

HRMS (ESI-TOF, m/z) calculated for $C_{22}H_{23}O_2^+$ $[M+H]^+$ 319.1693, found 319.1685.

4-(1-(3,4,5-trimethoxyphenyl)ethyl)-1,1'-biphenyl (10)

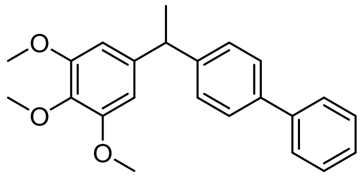

A Schlenk tube (10 mL) was charged with 1,2,3-trimethoxybenzene (**1h**, 50.5 mg, 0.30 mmol), 4-ethylbiphenyl (**2a**, 164.0 mg, 0.90 mmol), $FeCl_3$ $6H_2O$ (16.2 mg, 0.060 mmol) and $(CHCl_2)_2$ (0.75 mL). The mixture was degassed *via* three freeze-pump-thaw cycles, then filled with dry air. The Schlenk tube was positioned approximately 5 cm away from a 50 W LED lamp (λ_{max} = 410 nm). After being stirred at 25 ℃ for 48 h, the reaction mixture was concentrated to dryness. Purification using silica gel column chromatography (elution with PE:DCM = 2:1) gave the pure product **10** as a white solid (70.0 mg, 0.201 mmol, 67% yield).

^{1}H NMR (500 MHz, $CDCl_3$) δ 7.59 (d, *J* = 7.8 Hz, 2H), 7.54 – 7.51 (m, 2H), 7.45 – 7.41 (m, 2H), 7.33 (t, *J* = 8.1 Hz, 3H), 6.95 (d, *J* = 8.6 Hz, 1H), 6.68 (d, *J* = 8.6 Hz, 1H), 4.52 (q, *J* = 7.1 Hz, 1H), 3.89 (s, 3H), 3.86 (s, 3H), 3.70 (s, 3H), 1.63 (d, *J* = 7.3 Hz, 3H).

^{13}C NMR (126 MHz, $CDCl_3$) δ 152.2, 151.7, 146.2, 142.4, 141.2, 138.7, 132.5, 128.8, 128.1, 127.1, 127.1, 127.0, 122.0, 107.2, 60.9, 60.8, 56.1, 37.7, 21.8.

IR (film): *v* (cm^{-1}) 3507, 2972, 1867, 1608, 1431, 1396, 1326, 1314, 1251, 1078, 831, 666, 585, 542.

HRMS (ESI-TOF, m/z) calculated for $C_{23}H_{25}O_3^+$ $[M+H]^+$ 349.1798, found 349.1803.

1-(1-([1,1'-biphenyl]-4-yl)ethyl)-4-methoxynaphthalene (11)

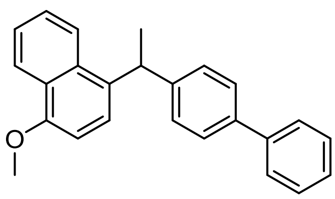

A Schlenk tube (10 mL) was charged with 1-methoxynaphthalene (**1i**, 47.5 mg, 0.30 mmol), 4-ethylbiphenyl (**2a**, 164.0 mg, 0.90 mmol), $FeCl_3$ $6H_2O$ (16.2 mg, 0.060 mmol) and $(CHCl_2)_2$ (0.75 mL). The mixture was degassed *via* three freeze-pump-thaw cycles, then

filled with dry air. The Schlenk tube was positioned approximately 5 cm away from a 50 W LED lamp (λ_{max} = 410 nm). After being stirred at 25 ℃ for 48 h, the reaction mixture was concentrated to dryness. Purification using silica gel column chromatography (elution with PE:DCM = 4:1) gave the pure product **11** as a white solid (55.8 mg, 0.165 mmol, 55% yield).

^{1}H NMR (500 MHz, $CDCl_3$) δ 7.59 (d, *J* = 8.9 Hz, 1H), 7.53 (d, *J* = 7.6 Hz, 2H), 7.46 – 7.44 (m, 2H), 7.40 (d, *J* = 8.2 Hz, 2H), 7.29 (t, *J* = 7.6 Hz, 2H), 7.20 (d, *J* = 8.1 Hz, 4H), 7.03 (m, 1H), 6.98 (d, *J* = 2.2 Hz, 1H), 4.20 (q, *J* = 7.1 Hz, 1H), 3.76 (s, 3H), 1.63 (d, *J* = 7.2 Hz, 3H).

^{13}C NMR (126 MHz, $CDCl_3$) δ 157.5, 145.7, 141.5, 141.1, 139.1, 133.3, 129.3, 129.1, 128.8, 128.3, 127.4, 127.2, 127.2, 127.1, 127.0, 125.4, 118.8, 105.8, 55.4, 44.5, 22.0.

IR (film): *ν* (cm^{-1}) 3507, 3096, 1955, 1587, 1431, 1390, 1274, 1251, 1003, 845, 618, 497, 472.

HRMS (ESI-TOF, m/z) calculated for $C_{25}H_{23}O^+$ $[M+H]^+$ 339.1743, found 339.1744.

5-(1-([1,1'-biphenyl]-4-yl)ethyl)-2,3-dihydrobenzofuran (12)

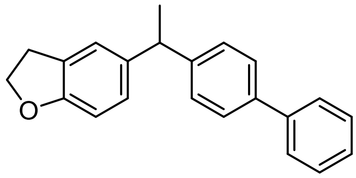

A Schlenk tube (10 mL) was charged with 2,3-dihydrobenzofuran (**1j**, 36.0 mg, 0.30 mmol), 4-ethylbiphenyl (**2a**, 164.0 mg, 0.90 mmol), $FeCl_3 \cdot 6H_2O$ (16.2 mg, 0.060 mmol) and $(CHCl_2)_2$ (0.75 mL). The mixture was degassed *via* three freeze-pump-thaw cycles, then filled with dry air. The Schlenk tube was positioned approximately 5 cm away from a 50 W LED lamp (λ_{max} = 410 nm). After being stirred at 25 ℃ for 48 h, the reaction mixture was concentrated to dryness. Purification using silica gel column chromatography (elution with PE:DCM = 5:1) gave the pure product **12** as a colorless oil (51.3 mg, 0.171 mmol, 57% yield).

^{1}H NMR (500 MHz, $CDCl_3$) δ 7.60 (m, 2H), 7.56 – 7.54 (m, 2H), 7.45 (t, *J* = 7.7 Hz, 2H), 7.36 – 7.32 (m, 3H), 7.10 (s, 1H), 7.06 – 7.04 (m, 1H), 6.76 (d, *J* = 8.2 Hz, 1H), 4.56 (t, *J* = 8.7 Hz, 2H), 4.17 (q, *J* = 7.2 Hz, 1H), 3.19 (t, *J* = 8.7 Hz, 2H), 1.68 (d, *J* = 7.2 Hz, 3H).

^{13}C NMR (126 MHz, $CDCl_3$) δ 158.5, 146.2, 141.1, 138.9, 138.6, 128.8, 128.0, 127.2, 127.2, 127.1, 124.2, 109.1, 71.3, 44.0, 30.0, 29.8, 22.3.

IR (film): *ν* (cm^{-1}) 3067, 3012, 2779, 1856, 1751, 1478, 1407, 1266, 1251, 1038, 851, 550.

HRMS (ESI-TOF, m/z) calculated for $C_{22}H_{20}NaO^+$ $[M+Na]^+$ 323.1406, found 323.1412.

5-(1-([1,1'-biphenyl]-4-yl)ethyl)benzo[*d*][1,3]dioxole (13)

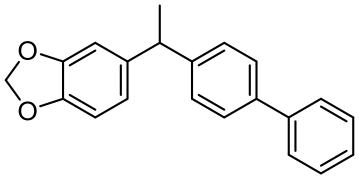

A Schlenk tube (10 mL) was charged with 1,3-benzodioxole (**1k**, 36.6 mg, 0.30 mmol), 4-ethylbiphenyl (**2a**, 164.0 mg, 0.90 mmol), $FeCl_3 \cdot 6H_2O$ (16.2 mg, 0.060 mmol) and $(CHCl_2)_2$ (0.75 mL). The mixture was degassed *via* three freeze-pump-thaw cycles, then filled with dry air. The Schlenk tube was positioned approximately 5 cm away from a 50 W LED lamp (λ_{max} = 410 nm). After being stirred at 25 ℃ for 48 h, the reaction mixture was concentrated to dryness. Purification using silica gel column chromatography (elution with PE:DCM = 5:1) gave the pure product **13** as a white solid (62.5 mg, 0.207 mmol, 69% yield).

^{1}H NMR (500 MHz, $CDCl_3$) δ 7.59 (d, *J* = 7.6 Hz, 2H), 7.54 (d, *J* = 8.1 Hz, 2H), 7.44 (t, *J* = 7.6 Hz, 2H), 7.35 (d, *J* = 7.5 Hz, 1H), 7.31 (d, *J* = 8.2 Hz, 2H), 6.78 – 6.74 (m, 3H), 5.93 (s, 2H), 4.14 (q, *J* = 7.2 Hz, 1H), 1.66 (d, *J* = 7.2 Hz, 3H).

^{13}C NMR (126 MHz, $CDCl_3$) δ 147.8, 145.9, 145.7, 141.1, 140.5, 139.1, 128.8, 128.0, 127.3, 127.2, 127.2, 120.5, 108.3, 108.2, 101.0, 44.3, 22.2.

IR (film): *v* (cm^{-1}) 3601, 3061, 3033, 2770, 1751, 1447, 1407, 1266, 1251, 1091, 851, 737, 550.

HRMS (ESI-TOF, m/z) calculated for $C_{21}H_{19}O_2^+$ $[M+H]^+$ 303.1380, found 303.1383.

6-(1-([1,1'-biphenyl]-4-yl)ethyl)-2,3-dihydrobenzo[*b*][1,4]dioxine (14)

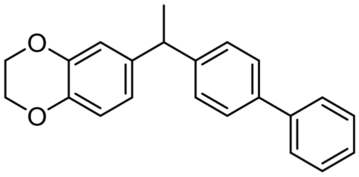

A Schlenk tube (10 mL) was charged with 1,4-benzodioxan (**1l**, 58.1 mg, 0.30 mmol), 4-ethylbiphenyl (**2a**, 164.0 mg, 0.90 mmol), $FeCl_3 \cdot 6H_2O$ (16.2 mg, 0.060 mmol) and $(CHCl_2)_2$ (0.75 mL). The mixture was degassed *via* three freeze-pump-thaw cycles, then filled with dry air. The Schlenk tube was positioned approximately 5 cm away from a 50 W LED lamp (λ_{max} = 410 nm). After being stirred at 25 ℃ for 48 h, the reaction mixture was concentrated to dryness. Purification using silica gel column chromatography (elution with PE:DCM = 5:1) gave the pure product **14** as a colorless oil (65.4 mg, 0.207 mmol, 69% yield).

^{1}H NMR (500 MHz, $CDCl_3$) δ 7.57 (d, *J* = 7.3 Hz, 2H), 7.52 (d, *J* = 8.2 Hz, 2H), 7.42 (t, *J* = 7.6 Hz, 2H), 7.31 (dd, *J* = 18.2, 7.7 Hz, 3H), 6.77 (m, 3H), 4.24 (s, 4H), 4.10 (q, *J* = 7.2 Hz,

1H), 1.64 (d, J = 7.2 Hz, 3H).

^{13}C NMR (126 MHz, $CDCl_3$) δ 145.8, 143.4, 142.0, 141.2, 139.9, 139.1, 128.8, 128.0, 127.3, 127.2, 120.7, 117.2, 116.4, 64.6, 64.5, 44.0, 22.1.

IR (film): v (cm^{-1}) 3450, 3057, 2995, 2762, 1798, 1502, 1478, 1427, 1212, 1176, 1008, 715, 535.

HRMS (ESI-TOF, m/z) calculated for $C_{22}H_{21}O_2^+$ $[M+H]^+$ 317.1536, found 317.1543.

(4-(1-([1,1'-biphenyl]-4-yl)ethyl)phenyl)(methyl)sulfane (15)

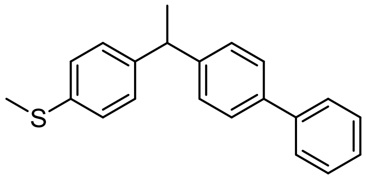

A Schlenk tube (10 mL) was charged with thioanisole (**1m**, 37.3 mg, 0.30 mmol), 4-ethylbiphenyl (**2a**, 164.0 mg, 0.90 mmol), $FeCl_3$ $6H_2O$ (16.2 mg, 0.060 mmol) and DCE (0.75 mL). The mixture was degassed *via* three freeze-pump-thaw cycles, then filled with dry air. The Schlenk tube was positioned approximately 5 cm away from a 50 W LED lamp (λ_{max} = 410 nm). After being stirred at 25 ℃ for 48 h, the reaction mixture was concentrated to dryness. Purification using silica gel column chromatography (elution with PE:DCM = 3:1) gave the pure product **15** as a colorless oil (48.4 mg, 0.159 mmol, 53% yield).

^{1}H NMR (500 MHz, $CDCl_3$) δ 7.57 – 7.53 (m, 2H), 7.51 – 7.48 (m, 2H), 7.42 – 7.38 (m, 2H), 7.32 – 7.28 (m, 1H), 7.26 (d, J = 8.1 Hz, 2H), 7.21 – 7.16 (m, 4H), 4.14 (q, J = 7.2 Hz, 1H), 2.43 (s, 3H), 1.64 (d, J = 7.2 Hz, 3H).

^{13}C NMR (126 MHz, $CDCl_3$) δ 145.5, 143.5, 141.1, 139.1, 135.9, 128.8, 128.3, 128.1, 127.3, 127.2, 127.1, 127.1, 44.1, 21.9, 16.3.

IR (film): v (cm^{-1}) 3510, 3076, 3062, 2985, 1791, 1302, 1272, 1095, 1025, 691, 478, 435.

HRMS (ESI-TOF, m/z) calculated for $C_{21}H_{21}S^+$ $[M+H]^+$ 305.1358, found 305.1355.

(4-(1-([1,1'-biphenyl]-4-yl)ethyl)phenyl)(phenyl)sulfane (16)

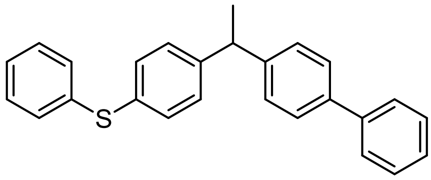

A Schlenk tube (10 mL) was charged with diphenylsulfane (**1n**, 55.8 mg, 0.30 mmol), 4-ethylbiphenyl (**2a**, 164.0 mg, 0.90 mmol), $FeCl_3$ $6H_2O$ (16.2 mg, 0.060 mmol) and DCE (0.75 mL). The mixture was degassed *via* three freeze-pump-thaw cycles, then filled with dry

air. The Schlenk tube was positioned approximately 5 cm away from a 50 W LED lamp (λ_{max} = 410 nm). After being stirred at 25 ℃ for 48 h, the reaction mixture was concentrated to dryness. Purification using silica gel column chromatography (elution with PE:DCM = 5:1) gave the pure product **16** as a white solid (54.9 mg, 0.150 mmol, 50% yield).

^{1}H NMR (500 MHz, $CDCl_3$) δ 7.57 – 7.54 (m, 2H), 7.51 (d, *J* = 8.2 Hz, 2H), 7.40 (t, *J* = 7.7 Hz, 2H), 7.36 – 7.23 (m, 9H), 7.22 – 7.18 (m, 3H), 4.16 (q, *J* = 7.2 Hz, 1H), 1.65 (d, *J* = 7.2 Hz, 3H).

^{13}C NMR (126 MHz, $CDCl_3$) δ 145.7, 145.2, 141.0, 139.3, 136.3, 132.9, 131.6, 130.8, 129.3, 128.9, 128.7, 128.1, 127.3, 127.2, 127.1, 126.9, 44.3, 21.9.

IR (film): *v* (cm^{-1}) 3503, 3019, 3004, 2856, 1723, 1685, 1483, 1075, 1027, 694, 478, 431.

HRMS (ESI-TOF, m/z) calculated for $C_{26}H_{23}S^+$ $[M+H]^+$ 367.1515, found 367.1506.

(4-(1-([1,1'-biphenyl]-4-yl)ethyl)phenyl)(cyclopropyl)sulfane (17)

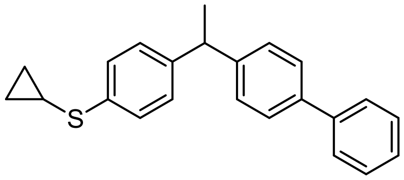

A Schlenk tube (10 mL) was charged with cyclopropyl(phenyl)sulfane (**1o**, 45.0 mg, 0.30 mmol), 4-ethylbiphenyl (**2a**, 164.0 mg, 0.90 mmol), $FeCl_3$ $6H_2O$ (16.2 mg, 0.060 mmol) and DCE (0.75 mL). The mixture was degassed *via* three freeze-pump-thaw cycles, then filled with dry air. The Schlenk tube was positioned approximately 5 cm away from a 50 W LED lamp (λ_{max} = 410 nm). After being stirred at 25 ℃ for 48 h, the reaction mixture was concentrated to dryness. Purification using silica gel column chromatography (elution with PE:DCM = 5:1) gave the pure product **17** as a colorless oil (49.5 mg, 0.150 mmol, 50% yield).

^{1}H NMR (500 MHz, $CDCl_3$) δ 7.63 – 7.60 (m, 2H), 7.56 (d, *J* = 8.2 Hz, 2H), 7.46 (t, *J* = 7.7 Hz, 2H), 7.38 – 7.32 (m, 5H), 7.23 (d, *J* = 8.3 Hz, 2H), 4.20 (q, *J* = 7.2 Hz, 1H), 2.21 (tt, *J* = 7.4, 4.4 Hz, 1H), 1.71 (d, *J* = 7.2 Hz, 3H), 1.09 – 1.05 (m, 2H), 0.74 – 0.71 (m, 2H).

^{13}C NMR (126 MHz, $CDCl_3$) δ 145.5, 143.4, 141.1, 139.1, 136.2, 128.8, 128.2, 128.1, 127.2, 127.2, 127.1, 126.9, 44.1, 22.0, 12.4, 8.6.

IR (film): *v* (cm^{-1}) 3492, 3053, 3012, 2766, 1723, 1685, 1480, 1095, 1074, 691, 476, 467.

HRMS (ESI-TOF, m/z) calculated for $C_{23}H_{23}S^+$ $[M+H]^+$ 331.1515, found 331.1510.

4-(1-([1,1'-biphenyl]-4-yl)ethyl)phenol (18)

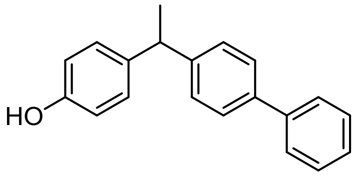

A Schlenk tube (10 mL) was charged with phenol (**1p**, 28.2 mg, 0.30 mmol), 4-ethylbiphenyl (**2a**, 164.0 mg, 0.90 mmol), $FeCl_3$ $6H_2O$ (16.2 mg, 0.060 mmol) and $CHCl_3$ (0.75 mL). The mixture was degassed *via* three freeze-pump-thaw cycles, then filled with dry air. The Schlenk tube was positioned approximately 5 cm away from a 50 W LED lamp (λ_{max} = 410 nm). After being stirred at 25 ℃ for 48 h, the reaction mixture was concentrated to dryness. Purification using silica gel column chromatography (elution with PE:DCM = 2:1) gave the pure product **18** as a colorless oil (45.2 mg, 0.164 mmol, 55% yield).

^{1}H NMR (500 MHz, $CDCl_3$) δ 7.60 (d, *J* = 7.4 Hz, 2H), 7.55 (d, *J* = 8.2 Hz, 2H), 7.45 (t, *J* = 7.7 Hz, 2H), 7.35 (t, *J* = 7.4 Hz, 1H), 7.31 (d, *J* = 8.2 Hz, 2H), 7.15 (d, *J* = 8.5 Hz, 2H), 6.79 (d, *J* = 8.5 Hz, 2H), 5.00 (br, s, 1H), 4.17 (q, *J* = 7.2 Hz, 1H), 1.67 (d, *J* = 7.2 Hz, 3H).

^{13}C NMR (126 MHz, $CDCl_3$) δ 153.9, 146.0, 141.1, 139,0, 138.7, 128.9, 128.8, 128.0, 127.2, 127.2, 127.1, 115.4, 43.8, 22.2.

IR (film): *ν* (cm^{-1}) 3226, 3219, 3204, 2956, 1723, 1632, 1473, 1295, 1237, 1118, 832, 694, 535, 478.

HRMS (ESI-TOF, m/z) calculated for $C_{20}H_{18}NaO^+$ $[M+Na]^+$ 297.1250, found 297.1257.

5-(1-([1,1'-biphenyl]-4-yl)ethyl)-[1,1'-biphenyl]-2-ol (19)

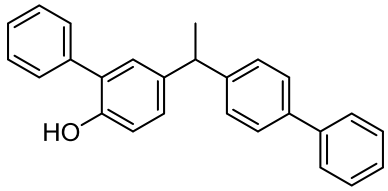

A Schlenk tube (10 mL) was charged with [1,1'-biphenyl]-2-ol (**1q**, 51.0 mg, 0.30 mmol), 4-ethylbiphenyl (**2a**, 164.0 mg, 0.90 mmol), $FeCl_3$ $6H_2O$ (16.2 mg, 0.060 mmol) and $CHCl_3$ (0.75 mL). The mixture was degassed *via* three freeze-pump-thaw cycles, then filled with dry air. The Schlenk tube was positioned approximately 5 cm away from a 50 W LED lamp (λ_{max} = 410 nm). After being stirred at 25 ℃ for 48 h, the reaction mixture was concentrated to dryness. Purification using silica gel column chromatography (elution with PE:DCM = 2:1) gave the pure product **19** as a white solid (47.3 mg, 0.135 mmol, 45% yield).

^{1}H NMR (500 MHz, $CDCl_3$) δ 7.54 (d, *J* = 7.3 Hz, 2H), 7.49 (d, *J* = 8.2 Hz, 2H), 7.44 – 7.41 (m, 4H), 7.37 (t, *J* = 7.7 Hz, 2H), 7.35 – 7.31 (m, 1H), 7.27 (d, *J* = 8.1 Hz, 3H), 7.11 (d, *J* =

8.6 Hz, 2H), 6.88 (d, *J* = 8.1 Hz, 1H), 5.14 (br, s, 1H), 4.14 (d, *J* = 7.2 Hz, 1H), 1.64 (d, *J* = 7.2 Hz, 3H).

^{13}C NMR (126 MHz, $CDCl_3$) δ 150.8, 145.9, 141.1, 139.0, 138.8, 137.4, 129.4, 129.3, 129.2, 128.8, 128.4, 128.0, 127.9, 127.2, 127.2, 127.1, 115.9, 43.9, 22.2.

IR (film): *v* (cm^{-1}) 3230, 3215, 3042, 2926, 1632, 1500, 1473, 1335, 1238, 881, 832, 691, 599, 551.

HRMS (ESI-TOF, m/z) calculated for $C_{26}H_{22}NaO^+$ $[M+Na]^+$ 373.1563, found 373.1555.

4-(1-([1,1'-biphenyl]-4-yl)ethyl)-2-isopropylphenol (20)

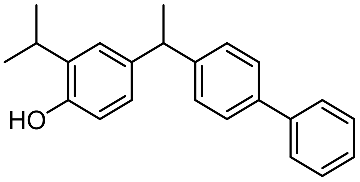

A Schlenk tube (10 mL) was charged with 2-isopropylphenol (**1r**, 40.9 mg, 0.30 mmol), 4-ethylbiphenyl (**2a**, 164.0 mg, 0.90 mmol), $FeCl_3$ $6H_2O$ (16.2 mg, 0.060 mmol) and $CHCl_3$ (0.75 mL). The mixture was degassed *via* three freeze-pump-thaw cycles, then filled with dry air. The Schlenk tube was positioned approximately 5 cm away from a 50 W LED lamp (λ_{max} = 410 nm). After being stirred at 25 ℃ for 48 h, the reaction mixture was concentrated to dryness. Purification using silica gel column chromatography (elution with PE:DCM = 1:1) gave the pure product **20** as a white solid (58.8 mg, 0.186 mmol, 62% yield).

^{1}H NMR (500 MHz, $CDCl_3$) δ 7.60 (d, *J* = 7.4 Hz, 2H), 7.54 (d, *J* = 8.1 Hz, 2H), 7.45 (t, *J* = 7.7 Hz, 2H), 7.33 (m, 3H), 7.13 (d, *J* = 2.3 Hz, 1H), 6.95 (m, 1H), 6.70 (d, *J* = 8.2 Hz, 1H), 4.74 (br, s, 1H), 4.18 – 4.14 (m, 1H), 3.21 (p, *J* = 6.9 Hz, 1H), 1.67 (d, *J* = 7.2 Hz, 3H), 1.28 (m, 6H).

^{13}C NMR (126 MHz, $CDCl_3$) δ 151.2, 146.2, 141.2, 138.9, 138.7, 134.3, 128.8, 128.0, 127.2, 127.1, 125.9, 125.7, 115.3, 44.1, 27.4, 22.7, 22.4.

IR (film): *v* (cm^{-1}) 3575, 3026, 2964, 2856, 1632, 1592, 1505, 1473, 1365, 1237, 1108, 793, 802, 674, 585, 528.

HRMS (ESI-TOF, m/z) calculated for $C_{23}H_{24}NaO^+$ $[M+Na]^+$ 339.1719, found 339.1724.

4-(1-([1,1'-biphenyl]-4-yl)ethyl)-2,6-dimethylphenol (21)

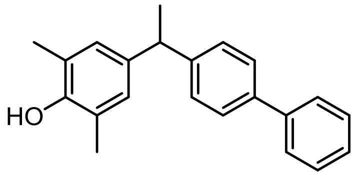

A Schlenk tube (10 mL) was charged with 2,6-dimethylphenol (**1s**, 36.6 mg, 0.30 mmol), 4-ethylbiphenyl (**2a**, 164.0 mg, 0.90 mmol), $FeCl_3 \cdot 6H_2O$ (16.2 mg, 0.060 mmol) and $CHCl_3$ (0.75 mL). The mixture was degassed *via* three freeze-pump-thaw cycles, then filled with dry air. The Schlenk tube was positioned approximately 5 cm away from a 50 W LED lamp (λ_{max} = 410 nm). After being stirred at 25 ℃ for 48 h, the reaction mixture was concentrated to dryness. Purification using silica gel column chromatography (elution with PE:DCM = 1:1) gave the pure product **21** as a white solid (65.3 mg, 0.216 mmol, 72% yield).

^{1}H NMR (500 MHz, $CDCl_3$) δ 7.58 (dd, *J* = 8.2, 1.1 Hz, 2H), 7.53 – 7.50 (m, 2H), 7.42 (t, *J* = 7.7 Hz, 2H), 7.31 (dd, *J* = 16.7, 7.8 Hz, 3H), 6.88 (s, 2H), 4.50 (br, s, 1H), 4.07 (q, *J* = 7.2 Hz, 1H), 2.23 (s, 6H), 1.63 (d, *J* = 7.2 Hz, 3H).

^{13}C NMR (126 MHz, $CDCl_3$) δ 150.6, 146.2, 141.2, 138.9, 138.1, 128.8, 128.0, 127.8, 127.2, 127.1, 123.0, 43.9, 22.2, 16.2.

IR (film): *ν* (cm^{-1}) 3506, 3056, 2938, 2856, 1606, 1585, 1443, 1365, 1237, 1082, 789, 670, 58, 525.

HRMS (ESI-TOF, m/z) calculated for $C_{22}H_{22}NaO^+$ $[M+Na]^+$ 325.1563, found 325.1572.

4-(1-([1,1'-biphenyl]-4-yl)ethyl)naphthalen-1-ol (22)

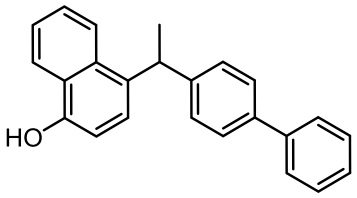

A Schlenk tube (10 mL) was charged with naphthalen-1-ol (**1t**, 43.3 mg, 0.30 mmol), 4-ethylbiphenyl (**2a**, 164.0 mg, 0.90 mmol), $FeCl_3 \cdot 6H_2O$ (16.2 mg, 0.060 mmol) and $CHCl_3$ (0.75 mL). The mixture was degassed *via* three freeze-pump-thaw cycles, then filled with dry air. The Schlenk tube was positioned approximately 5 cm away from a 50 W LED lamp (λ_{max} = 410 nm). After being stirred at 25 ℃ for 48 h, the reaction mixture was concentrated to dryness. Purification using silica gel column chromatography (elution with PE:DCM = 2:1) gave the pure product **22** as a white solid (41.9 mg, 0.129 mmol, 43% yield).

^{1}H NMR (500 MHz, $CDCl_3$) δ 8.12 – 8.10 (m, 1H), 7.83 – 7.81 (m, 1H), 7.56 (t, *J* = 8.6 Hz, 5H), 7.51 (s, 1H), 7.46 (d, *J* = 3.6 Hz, 2H), 7.43 (d, *J* = 7.6 Hz, 2H), 7.39 (d, *J* = 8.0 Hz, 2H), 7.35 (t, *J* = 7.4 Hz, 1H), 5.20 (br, s, 1H), 4.55 (q, *J* = 7.2 Hz, 1H), 1.79 (d, *J* = 7.2 Hz, 3H).

^{13}C NMR (126 MHz, $CDCl_3$) δ 148.5, 144.0, 140.8, 139.9, 133.6, 129.0, 128.1, 127.8, 127.7, 127.4, 127.2, 126.0, 125.9, 125.5, 125.2, 125.1, 121.3, 120.7, 39.1, 21.2.

IR (film): *ν* (cm^{-1}) 3607, 3056, 1906, 1517, 1461, 1408, 1314, 1298, 1169, 1082, 1040, 958, 852, 712, 577, 520, 472.

HRMS (ESI-TOF, m/z) calculated for $C_{24}H_{21}O^+$ $[M+H]^+$ 325.1587, found 325.1580.

4-(1-([1,1'-biphenyl]-4-yl)ethyl)-2-bromobenzene-1,3-diol (23)

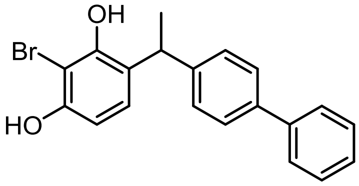

A Schlenk tube (10 mL) was charged with 2-bromoresorcinol (**1u**, 56.7 mg, 0.30 mmol), 4-ethylbiphenyl (**2a**, 164.0 mg, 0.90 mmol), $FeCl_3$ $6H_2O$ (16.2 mg, 0.060 mmol) and $CHCl_3$ (0.75 mL). The mixture was degassed *via* three freeze-pump-thaw cycles, then filled with dry air. The Schlenk tube was positioned approximately 5 cm away from a 50 W LED lamp (λ_{max} = 410 nm). After being stirred at 25 ℃ for 48 h, the reaction mixture was concentrated to dryness. Purification using silica gel column chromatography (elution with PE:DCM = 1:1) gave the pure product **23** as a white solid (60.9 mg, 0.165 mmol, 55% yield).

^{1}H NMR (500 MHz, $CDCl_3$) δ 7.59 – 7.57 (m, 2H), 7.53 (d, *J* = 8.2 Hz, 2H), 7.43 (t, *J* = 7.7 Hz, 2H), 7.33 (t, *J* = 7.7 Hz, 3H), 7.11 (d, *J* = 8.5 Hz, 1H), 6.63 (d, *J* = 8.5 Hz, 1H), 5.47 (br, s, 1H), 5.35 (br, s, 1H), 4.49 (q, *J* = 7.2 Hz, 1H), 1.64 (d, *J* = 7.3 Hz, 3H).

^{13}C NMR (126 MHz, $CDCl_3$) δ 151.1, 150.1, 144.8, 141.0, 139.1, 128.7, 127.9, 127.4, 127.2, 127.1, 127.1, 125.5, 107.7, 100.0, 38.2, 21.0.

IR (film): *ν* (cm^{-1}) 3500, 3074, 2639, 2488, 1909, 1777, 1352, 1248, 1184, 1166, 1042, 933, 847, 839, 555, 541, 488.

HRMS (ESI-TOF, m/z) calculated for $C_{20}H_{18}BrO_2^+$ $[M+H]^+$ 369.0485, found 369.0493.

5-(1-([1,1'-biphenyl]-4-yl)ethyl)-2-methoxy-4-methylphenol (24)

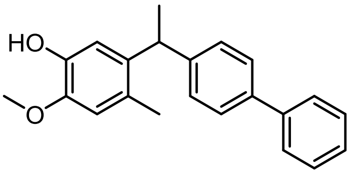

A Schlenk tube (10 mL) was charged with 2-methoxy-4-methylphenol (**1v**, 41.45 mg, 0.30 mmol), 4-ethylbiphenyl (**2a**, 164.0 mg, 0.90 mmol), $FeCl_3$ $6H_2O$ (16.2 mg, 0.060 mmol) and $CHCl_3$ (0.75 mL). The mixture was degassed *via* three freeze-pump-thaw cycles, then filled with dry air. The Schlenk tube was positioned approximately 5 cm away from a 50 W LED lamp (λ_{max} = 410 nm). After being stirred at 25 ℃ for 48 h, the reaction mixture was concentrated to dryness. Purification using silica gel column chromatography (elution with PE:DCM = 1:1) gave the pure product **24** as a white solid (64.9 mg, 0.204 mmol, 68% yield).

^{1}H NMR (500 MHz, $CDCl_3$) δ 7.60 (d, *J* = 7.4 Hz, 2H), 7.52 (d, *J* = 8.2 Hz, 2H), 7.45 (t, *J* = 7.6 Hz, 2H), 7.35 (t, *J* = 7.4 Hz, 1H), 7.27 (d, *J* = 8.1 Hz, 2H), 6.96 (s, 1H), 6.69 (s, 1H), 5.51 (br, s, 1H), 4.30 (q, *J* = 7.1 Hz, 1H), 3.88 (s, 3H), 2.25 (s, 3H), 1.64 (d, *J* = 7.2 Hz, 3H).

^{13}C NMR (126 MHz, $CDCl_3$) δ 145.7, 144.6, 143.7, 141.2, 138.8, 136.9, 128.8, 128.1, 127.4, 127.2, 127.1, 113.5, 113.1, 56.1, 40.4, 22.3, 19.4.

IR (film): *ν* (cm^{-1}) 3662, 3490, 2961, 2939, 2843, 1514, 1466, 1367, 1272, 1123, 1040, 845, 681, 557.

HRMS (ESI-TOF, m/z) calculated for $C_{22}H_{23}O_2^+$ $[M+H]^+$ 319.1693, found 319.1688.

5-(1-([1,1'-biphenyl]-4-yl)ethyl)-2-methoxyphenol (25)

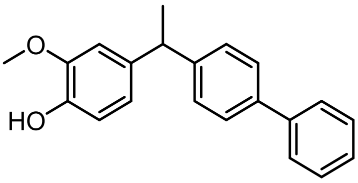

A Schlenk tube (10 mL) was charged with 2-methoxyphenol (**1w**, 37.2 mg, 0.30 mmol), 4-ethylbiphenyl (**2a**, 164.0 mg, 0.90 mmol), $FeCl_3$ $6H_2O$ (16.2 mg, 0.060 mmol) and $CHCl_3$ (0.75 mL). The mixture was degassed *via* three freeze-pump-thaw cycles, then filled with dry air. The Schlenk tube was positioned approximately 5 cm away from a 50 W LED lamp (λ_{max} = 410 nm). After being stirred at 25 ℃ for 48 h, the reaction mixture was concentrated to dryness. Purification using silica gel column chromatography (elution with PE:DCM = 1:1) gave the pure product **25** as a white solid (63.9 mg, 0.210 mmol, 70% yield).

^{1}H NMR (500 MHz, $CDCl_3$) δ 7.60 – 7.57 (m, 2H), 7.53 (d, *J* = 8.2 Hz, 2H), 7.43 (t, *J* = 7.7 Hz, 2H), 7.34 (d, *J* = 7.3 Hz, 1H), 7.30 (d, *J* = 8.1 Hz, 2H), 6.88 (d, *J* = 8.1 Hz, 1H), 6.80 (dd, *J* = 8.1, 1.6 Hz, 1H), 6.74 (d, *J* = 1.7 Hz, 1H), 5.51 (br, s, 1H), 4.14 (q, *J* = 7.2 Hz, 1H), 3.85 (s, 3H), 1.67 (d, *J* = 7.2 Hz, 3H).

^{13}C NMR (126 MHz, $CDCl_3$) δ 146.6, 146.0, 144.1, 141.1, 139.0, 138.4, 128.9, 128.0, 127.2, 127.2, 120.1, 114.3, 110.5, 56.0, 44.3, 22.3.

IR (film): *ν* (cm^{-1}) 3490, 2853, 1514, 1407, 1273, 1129, 1035, 838, 651, 595.

HRMS (ESI-TOF, m/z) calculated for $C_{21}H_{21}O_2^+$ $[M+H]^+$ 305.1536, found 305.1544.

4-(1-(4-methoxy-2-methylphenyl)ethyl)-1,1'-biphenyl (26)

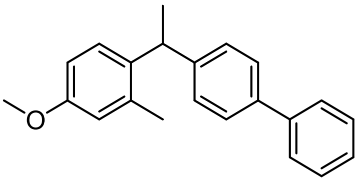

A Schlenk tube (10 mL) was charged with 1-methoxy-3-methylbenzene (**1x**, 36.7 mg, 0.30 mmol), 4-ethylbiphenyl (**2a**, 164.0 mg, 0.90 mmol), $FeCl_3$ $6H_2O$ (16.2 mg, 0.060 mmol) and $(CHCl_2)_2$ (0.75 mL). The mixture was degassed *via* three freeze-pump-thaw cycles, then

filled with dry air. The Schlenk tube was positioned approximately 5 cm away from a 50 W LED lamp (λ_{max} = 410 nm). After being stirred at 25 ℃ for 48 h, the reaction mixture was concentrated to dryness. Purification using silica gel column chromatography (elution with PE:DCM = 5:1) gave the pure product **26** as a white solid (59.8 mg, 0.198 mmol, 66% yield).

^{1}H NMR (500 MHz, $CDCl_3$) δ 7.59 – 7.56 (m, 2H), 7.51 – 7.49 (m, 2H), 7.43 (d, *J* = 5.6 Hz, 2H), 7.33 (s, 1H), 7.23 (dd, *J* = 8.4, 2.6 Hz, 3H), 6.78 (dd, *J* = 8.5, 2.7 Hz, 1H), 6.74 (d, *J* = 2.7 Hz, 1H), 4.31 (d, *J* = 7.2 Hz, 1H), 3.80 (s, 3H), 2.26 (s, 3H), 1.64 (d, *J* = 7.2 Hz, 3H).

^{13}C NMR (126 MHz, $CDCl_3$) δ 157.9, 145.9, 141.2, 138.8, 137.6, 136.3, 128.8, 128.1, 127.8, 127.2, 127.1, 127.1, 116.3, 111.1, 55.3, 40.2, 22.5, 20.2.

IR (film): *v* (cm^{-1}) 3473, 3452, 3031, 2953, 2858, 1513, 1465, 1435, 1298, 1176, 1125, 1110, 817, 646, 595.

HRMS (ESI-TOF, m/z) calculated for $C_{22}H_{22}NaO^+$ $[M+Na]^+$ 325.1563, found 325.1556.

triphenylmethane (27)

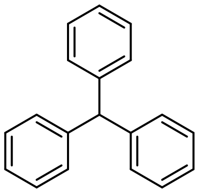

A Schlenk tube (10 mL) was charged with benzene (**1y**, 69.6 mg, 0.30 mmol), diphenylmethane (**2n**, 164.8 mg, 0.90 mmol), $FeBr_3$ (17.7 mg, 0.11 mmol) and DCE (0.75 mL). The mixture was degassed *via* three freeze-pump-thaw cycles, then filled with dry air. The Schlenk tube was positioned approximately 5 cm away from a 50 W LED lamp (λ_{max} = 410 nm). After being stirred at 25 ℃ for 48 h, the reaction mixture was concentrated to dryness. Purification using silica gel column chromatography (elution with PE) gave the pure product **27** as a yellow oil (34.2 mg, 0.140 mmol, 45% yield).

^{1}H NMR (500 MHz, $CDCl_3$) δ 7.31 (t, *J* = 7.5 Hz, 6H), 7.24 (t, *J* = 7.3 Hz, 3H), 7.15 (d, *J* = 7.5 Hz, 6H), 5.58 (s, 1H).

^{13}C NMR (126 MHz, $CDCl_3$) δ 144.0, 129.6, 128.4, 126.4, 57.0.

IR (film): *v* (cm^{-1}) 3103, 3082, 3066, 3000, 1899, 1594, 1493, 1446, 1078, 1032, 1004, 977, 920, 857, 700, 620, 494, 466.

HRMS (ESI-TOF, m/z) calculated for $C_{19}H_{17}^+$ $[M+H]^+$ 245.1325, found 245.1327.

1-bromo-4-(1-(*p*-tolyl)ethyl)benzene (28)

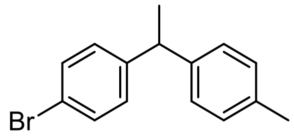

A Schlenk tube (10 mL) was charged with toluene (**1z**, 27.6 mg, 0.30 mmol), 4-bromoethylbenzene (**2f**, 164.8 mg, 0.90 mmol), $FeBr_3$ (17.7 mg, 0.11 mmol) and DCE (0.75 mL). The mixture was degassed *via* three freeze-pump-thaw cycles, then filled with dry air. The Schlenk tube was positioned approximately 5 cm away from a 50 W LED lamp (λ_{max} = 410 nm). After being stirred at 25 ℃ for 48 h, the reaction mixture was concentrated to dryness. Purification using silica gel column chromatography (elution with PE) gave the pure product **28** as a light yellow oil (50.1 mg, 0.183 mmol, 61% yield).

^{1}H NMR (500 MHz, $CDCl_3$) δ 7.40 (d, *J* = 8.3 Hz, 2H), 7.14 – 7.06 (m, 6H), 4.08 (dd, *J* = 14.3, 7.1 Hz, 1H), 2.32 (s, 3H), 1.61 (d, *J* = 7.2 Hz, 3H).

^{13}C NMR (126 MHz, $CDCl_3$) δ 145.8, 142.9, 135.9, 131.5, 129.5, 129.3, 127.5, 119.9, 44.0, 21.9, 21.1.

IR (film): *v* (cm^{-1}) 3079, 3040, 2943, 2874, 1774, 1593, 1488, 1453, 1375, 1317, 1234, 1205, 1011, 977, 705, 513.

HRMS (ESI-TOF, m/z) calculated for $C_{15}H_{16}Br^+$ $[M+H]^+$ 275.0430, found 275.0438.

1-bromo-4-(1-(4-(*tert*-butyl)phenyl)ethyl)benzene (29)

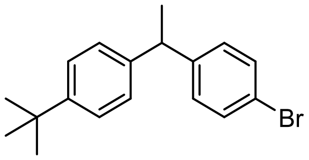

A Schlenk tube (10 mL) was charged with *tert*-butylbenzene (**1za**, 40.3 mg, 0.30 mmol), 4-bromoethylbenzene (**2f**, 164.8 mg, 0.90 mmol), $FeBr_3$ (17.7 mg, 0.11 mmol) and DCE (0.75 mL). The mixture was degassed *via* three freeze-pump-thaw cycles, then filled with dry air. The Schlenk tube was positioned approximately 5 cm away from a 50 W LED lamp (λ_{max} = 410 nm). After being stirred at 25 ℃ for 48 h, the reaction mixture was concentrated to dryness. Purification using silica gel column chromatography (elution with PE) gave the pure product **29** as a light yellow oil (44.3 mg, 0.140 mmol, 47% yield).

^{1}H NMR (500 MHz, $CDCl_3$) δ 7.40 (d, *J* = 8.3 Hz, 2H), 7.32 (d, *J* = 8.2 Hz, 2H), 7.12 (dd, *J* = 10.8, 6.1 Hz, 4H), 4.09 (dd, *J* = 14.9, 7.7 Hz, 1H), 1.62 (d, *J* = 7.0 Hz, 3H), 1.31 (s, 9H).

^{13}C NMR (126 MHz, $CDCl_3$) δ 149.2, 145.8, 142.7, 131.5, 129.6, 127.2, 125.5, 119.9, 44.4, 43.9, 34.5, 31.5, 21.9.

IR (film): *v* (cm^{-1}) 3079, 3040, 2943, 2874, 1942, 1787, 1488, 1453, 1375, 1317, 1205, 1119,

927, 845, 553.

HRMS (ESI-TOF, m/z) calculated for $C_{18}H_{22}Br^+$ $[M+H]^+$ 317.0899, found 317.0885.

4-(1-(4-bromophenyl)ethyl)-1,1'-biphenyl (30)

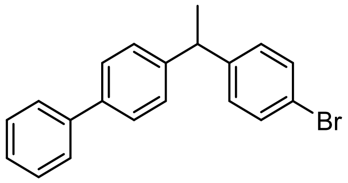

A Schlenk tube (10 mL) was charged with 1,1'-biphenyl (**1zb**, 46.3 mg, 0.30 mmol), 4-bromoethylbenzene (**2f**, 164.8 mg, 0.90 mmol), $FeBr_3$ (17.7 mg, 0.11 mmol) and DCE (0.75 mL). The mixture was degassed *via* three freeze-pump-thaw cycles, then filled with dry air. The Schlenk tube was positioned approximately 5 cm away from a 50 W LED lamp (λ_{max} = 410 nm). After being stirred at 25 ℃ for 48 h, the reaction mixture was concentrated to dryness. Purification using silica gel column chromatography (elution with PE) gave the pure product **30** as a light yellow oil (53.8 mg, 0.160 mmol, 53% yield).

^{1}H NMR (500 MHz, $CDCl_3$) δ 7.57 (t, *J* = 6.5 Hz, 2H), 7.53 (d, *J* = 8.2 Hz, 2H), 7.44 – 7.41 (m, 4H), 7.34 (t, *J* = 7.4 Hz, 1H), 7.27 (d, *J* = 8.1 Hz, 2H), 7.14 (d, *J* = 8.3 Hz, 2H), 4.16 (q, *J* = 7.2 Hz, 1H), 1.66 (d, *J* = 7.2 Hz, 3H).

^{13}C NMR (126 MHz, $CDCl_3$) δ 145.4, 144.9, 141.0, 139.3, 131.6, 129.6, 128.9, 128.1, 127.3, 127.3, 127.2, 120.0, 44.1, 21.9.

IR (film): v (cm^{-1}) 3111, 3079, 3066, 2973, 2876, 1944, 1594, 1401, 1317, 1155, 1011, 917, 765, 613, 478.

HRMS (ESI-TOF, m/z) calculated for $C_{20}H_{18}Br^+$ $[M+H]^+$ 337.0586, found 337.0580.

4-(1-(4-bromophenyl)ethyl)-4'-chloro-1,1'-biphenyl (31)

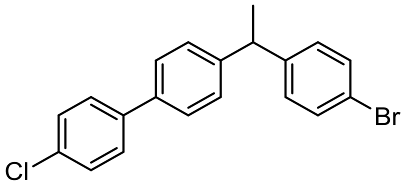

A Schlenk tube (10 mL) was charged with 4-chloro-1,1'-biphenyl (**1zc**, 56.6 mg, 0.30 mmol), 4-bromoethylbenzene (**2f**, 164.8 mg, 0.90 mmol), $FeBr_3$ (17.7 mg, 0.11 mmol) and DCE (0.75 mL). The mixture was degassed *via* three freeze-pump-thaw cycles, then filled with dry air. The Schlenk tube was positioned approximately 5 cm away from a 50 W LED lamp (λ_{max} = 410 nm). After being stirred at 25 ℃ for 48 h, the reaction mixture was concentrated to dryness. Purification using silica gel column chromatography (elution with PE) gave the pure product **31** as a light yellow oil (50.0 mg, 0.135 mmol, 45% yield).

^{1}**H NMR** (500 MHz, $CDCl_3$) δ 7.49 (dd, *J* = 8.2, 5.8 Hz, 4H), 7.42 (dd, *J* = 16.5, 8.4 Hz, 4H), 7.27 (s, 1H), 7.25 (s, 1H), 7.12 (d, *J* = 8.4 Hz, 2H), 4.15 (q, *J* = 7.2 Hz, 1H), 1.65 (d, *J* = 7.2 Hz, 3H).

^{13}**C NMR** (126 MHz, $CDCl_3$) δ 145.4, 145.3, 139.4, 138.1, 133.4, 131.6, 129.5, 129.0, 128.4, 128.2, 127.2, 120.1, 44.1, 21.8.

IR (film): *ν* (cm^{-1}) 3088, 3035, 1969, 1902, 1597, 1566, 1479, 1400, 1388, 1312, 1123, 1099, 1005, 911, 833, 707, 687, 544, 498.

HRMS (ESI-TOF, m/z) calculated for $C_{20}H_{17}BrCl^+$ $[M+H]^+$ 371.0197, found 371.0191.

4-bromo-4'-(1-(4-bromophenyl)ethyl)-1,1'-biphenyl (32)

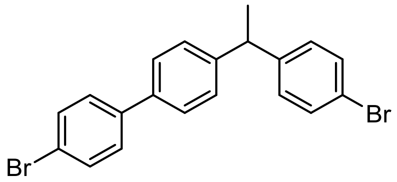

A Schlenk tube (10 mL) was charged with 4-bromo-1,1'-biphenyl (**1zd**, 69.9 mg, 0.30 mmol), 4-bromoethylbenzene (**2f**, 164.8 mg, 0.90 mmol), $FeBr_3$ (17.7 mg, 0.11 mmol) and DCE (0.75 mL). The mixture was degassed *via* three freeze-pump-thaw cycles, then filled with dry air. The Schlenk tube was positioned approximately 5 cm away from a 50 W LED lamp (λ_{max} = 410 nm). After being stirred at 25 ℃ for 48 h, the reaction mixture was concentrated to dryness. Purification using silica gel column chromatography (elution with PE) gave the pure product **32** as a yellow oil (58.3 mg, 0.141 mmol, 47% yield).

^{1}**H NMR** (500 MHz, $CDCl_3$) δ 7.53 (d, *J* = 8.2 Hz, 2H), 7.46 (d, *J* = 7.9 Hz, 2H), 7.41 (d, *J* = 8.3 Hz, 4H), 7.25 – 7.23 (m, 2H), 7.11 (d, *J* = 8.2 Hz, 2H), 4.14 (q, *J* = 7.1 Hz, 1H), 1.64 (d, *J* = 7.1 Hz, 3H).

^{13}**C NMR** (126 MHz, $CDCl_3$) δ 145.4, 145.3, 139.9, 138.1, 132.0, 131.7, 129.5, 128.7, 128.2, 127.1, 121.5, 120.1, 44.1, 21.8.

IR (film): *ν* (cm^{-1}) 3128, 3031, 2066, 1908, 1587, 1551, 1449, 1388, 1309, 1019, 1007, 991, 830, 755, 691, 636, 544.

HRMS (ESI-TOF, m/z) calculated for $C_{20}H_{17}Br_2^+$ $[M+H]^+$ 414.9692, found 414.9698.

3-(1-([1,1'-biphenyl]-4-yl)ethyl)-1*H*-indole (33)

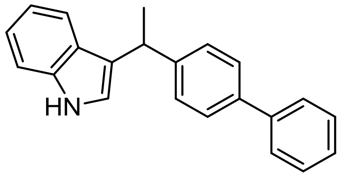

A Schlenk tube (10 mL) was charged with indole (**1ze**, 35.1 mg, 0.30 mmol), 4-ethylbiphenyl (**2a**, 164.0 mg, 0.90 mmol), $FeCl_3$ $6H_2O$ (16.2 mg, 0.060 mmol) and CH_2Cl_2 (0.75 mL). The mixture was degassed *via* three freeze-pump-thaw cycles, then filled with dry air. The Schlenk tube was positioned approximately 5 cm away from a 50 W LED lamp (λ_{max} = 410 nm). After being stirred at 25 ℃ for 48 h, the reaction mixture was concentrated to dryness. Purification using silica gel column chromatography (elution with PE:DCM = 2:1) gave the pure product **33** as a yellow oil (51.7 mg, 0.174 mmol, 58% yield).

^{1}H NMR (500 MHz, $CDCl_3$) δ 7.98 (s, 1H), 7.59 (d, *J* = 1.3 Hz, 1H), 7.57 (d, *J* = 0.9 Hz, 1H), 7.51 (d, *J* = 8.2 Hz, 2H), 7.41 (d, *J* = 7.9 Hz, 3H), 7.38 (s, 1H), 7.37 – 7.35 (m, 2H), 7.33 (d, *J* = 7.4 Hz, 1H), 7.19 – 7.15 (m, 1H), 7.06 – 7.01 (m, 2H), 4.44 (q, *J* = 7.1 Hz, 1H), 1.75 (d, *J* = 7.2 Hz, 3H).

^{13}C NMR (126 MHz, $CDCl_3$) δ 146.1, 141.3, 138.9, 136.7, 128.8, 128.0, 127.2, 127.1, 127.1, 127.0, 122.2, 121.5, 121.2, 119.9, 119.4, 111.2, 36.7, 22.5 .

IR (film): *v* (cm^{-1}) 3888, 3497, 3400, 3060, 2856, 1613, 1577, 1487, 1358, 1092, 1011, 933, 870, 775, 738, 612, 487.

HRMS (ESI-TOF, m/z) calculated for $C_{22}H_{20}N^+$ $[M+H]^+$ 298.1590, found 29.1596.

7-bromo-3-(1-(4-fluorophenyl)ethyl)-1*H*-indole (34)

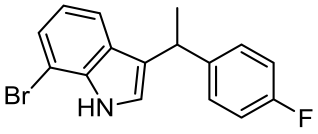

A Schlenk tube (10 mL) was charged with 7-bromo-1*H*-indole (**1zf**, 58.8 mg, 0.30 mmol), 1-bromo-4-isopropylbenzene (**2d,** 179.2 mg, 0.90 mmol), $FeCl_3$ $6H_2O$ (16.2 mg, 0.060 mmol) and CH_2Cl_2 (0.75 mL). The mixture was degassed *via* three freeze-pump-thaw cycles, then filled with dry air. The Schlenk tube was positioned approximately 5 cm away from a 50 W LED lamp (λ_{max} = 410 nm). After being stirred at 25 ℃ for 48 h, the reaction mixture was concentrated to dryness. Purification using silica gel column chromatography (elution with PE:DCM = 2:1) gave the pure product **34** as a light yellow oil (47.7 mg, 0.149 mmol, 50% yield).

^{1}H NMR (500 MHz, $CDCl_3$) δ 8.18 (s, 1H), 7.31 (d, *J* = 7.3 Hz, 1H), 7.24 – 7.19 (m, 3H), 7.09 (dd, *J* = 2.2, 0.8 Hz, 1H), 6.95 (td, *J* = 6.6, 3.3 Hz, 2H), 6.89 (t, *J* = 7.8 Hz, 1H), 4.33 (q, *J* = 7.1 Hz, 1H), 1.68 (d, *J* = 7.1 Hz, 3H).

^{13}C NMR (126 MHz, $CDCl_3$) δ 161.4, 142.2, 135.5, 128.9, 128.1, 124.5, 122.6, 121.7, 120.6, 119.0, 115.2, 104.8, 36.5, 22.6.

IR (film): *v* (cm^{-1}) 3870, 3512, 3411, 3123, 2907, 1663, 1607, 1423, 1366, 1083, 999, 941, 795, 765, 723, 506, 487.

HRMS (ESI-TOF, m/z) calculated for $C_{16}H_{14}BrFN^+$ $[M+H]^+$ 318.0288, found 318.0297.

7-bromo-3-(2-(4-bromophenyl)propan-2-yl)-1*H*-indole (35)

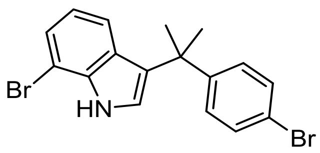

A Schlenk tube (10 mL) was charged with 7-bromo-1*H*-indole (**1zf**, 58.8 mg, 0.30 mmol), 1-bromo-4-isopropylbenzene (**2zk,** 179.2 mg, 0.90 mmol), $FeCl_3$ $6H_2O$ (16.2 mg, 0.060 mmol) and CH_2Cl_2 (0.75 mL). The mixture was degassed *via* three freeze-pump-thaw cycles, then filled with dry air. The Schlenk tube was positioned approximately 5 cm away from a 50 W LED lamp (λ_{max} = 410 nm). After being stirred at 25 ℃ for 48 h, the reaction mixture was concentrated to dryness. Purification using silica gel column chromatography (elution with PE:DCM = 2:1) gave the pure product **35** as a light yellow oil (57.8 mg, 0.147 mmol, 49% yield).

^{1}H NMR (500 MHz, $CDCl_3$) δ 8.17 (s, 1H), 7.42 – 7.39 (m, 2H), 7.34 – 7.32 (m, 1H), 7.25 – 7.22 (m, 2H), 7.19 (d, *J* = 2.4 Hz, 1H), 7.02 (d, *J* = 8.0 Hz, 1H), 6.83 (t, *J* = 7.8 Hz, 1H), 1.78 (s, 6H).

^{13}C NMR (126 MHz, $CDCl_3$) δ 148.7, 135.8, 131.2, 128.4, 127.1, 126.8, 124.3, 121.3, 120.43, 120.4, 119.7, 104.9, 39.0, 30.6.

IR (film): ν (cm^{-1}) 3300, 3085, 2927, 2887, 1663, 1555, 1423, 1326, 1300, 10832, 921, 906, 533.

HRMS (ESI-TOF, m/z) calculated for $C_{17}H_{16}Br_2N^+$ $[M+H]^+$ 393.9624, found 393.9622.

3-(1-([1,1'-biphenyl]-4-yl)ethyl)-5-bromo-1-methyl-1*H*-indole (36)

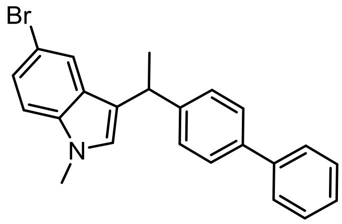

A Schlenk tube (10 mL) was charged with 5-bromo-1-methyl-1*H*-indole (**1zg**, 63.0 mg, 0.30 mmol), 4-ethylbiphenyl (**2a**, 164.0 mg, 0.90 mmol), $FeCl_3$ $6H_2O$ (16.2 mg, 0.060 mmol) and CH_2Cl_2 (0.75 mL). The mixture was degassed *via* three freeze-pump-thaw cycles, then filled with dry air. The Schlenk tube was positioned approximately 5 cm away from a 50 W LED lamp (λ_{max} = 410 nm). After being stirred at 25 ℃ for 48 h, the reaction mixture was concentrated to dryness. Purification using silica gel column chromatography (elution with PE:DCM = 2:1) gave the pure product **36** as a yellow oil (71.2 mg, 0.183 mmol, 61% yield).

^{1}H NMR (500 MHz, $CDCl_3$) δ 7.63 – 7.58 (m, 3H), 7.55 (d, *J* = 8.0 Hz, 2H), 7.45 (t, *J* = 7.6 Hz, 2H), 7.37 (d, *J* = 8.1 Hz, 2H), 7.36 – 7.33 (m, 1H), 7.29 (dd, *J* = 8.6, 1.3 Hz, 1H), 7.16 (d, *J* = 8.7 Hz, 1H), 6.89 (s, 1H), 4.38 (q, *J* = 7.1 Hz, 1H), 3.74 (s, 3H), 1.74 (d, *J* = 7.2 Hz, 3H).

^{13}C NMR (126 MHz, $CDCl_3$) δ 145.6, 141.1, 139.0, 136.04 128.9, 128.8, 127.8, 127.2, 127.1, 124.5, 122.2, 119.6, 112.2, 110.7, 36.4, 32.9, 22.5.

IR (film): *ν* (cm^{-1}) 3436, 2907, 1632, 1503, 1334, 1250, 1197, 899, 854, 606.

HRMS (ESI-TOF, m/z) calculated for $C_{23}H_{21}BrN^+$ $[M+H]^+$ 390.0852, found 390.0859.

1-(3-(1-([1,1'-biphenyl]-4-yl)ethyl)-1*H*-indol-1-yl)ethan-1-one (37)

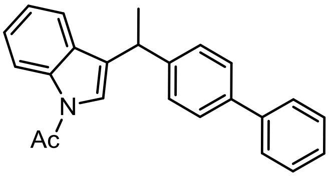

A Schlenk tube (10 mL) was charged with 1-(1*H*-indol-1-yl)ethan-1-one (**1zh**, 47.8 mg, 0.30 mmol), 4-ethylbiphenyl (**2a**, 164.0 mg, 0.90 mmol), $FeCl_3$ $6H_2O$ (16.2 mg, 0.060 mmol) and CH_2Cl_2 (0.75 mL). The mixture was degassed *via* three freeze-pump-thaw cycles, then filled with dry air. The Schlenk tube was positioned approximately 5 cm away from a 50 W LED lamp (λ_{max} = 410 nm). After being stirred at 25 ℃ for 48 h, the reaction mixture was concentrated to dryness. Purification using silica gel column chromatography (elution with PE:DCM = 2:1) gave the pure product **37** as a light yellow oil (71.3 mg, 0.210 mmol, 70% yield).

^{1}H NMR (500 MHz, $CDCl_3$) δ 8.46 (s, 1H), 7.60 (d, *J* = 7.5 Hz, 2H), 7.56 (d, *J* = 8.2 Hz, 2H), 7.44 (t, *J* = 7.7 Hz, 2H), 7.39 – 7.29 (m, 6H), 7.21 (d, *J* = 7.4 Hz, 1H), 4.36 (q, *J* = 7.0 Hz, 1H), 2.66 (s, 3H), 1.77 (d, *J* = 7.1 Hz, 3H).

^{13}C NMR (126 MHz, $CDCl_3$) δ 168.6, 144.2, 140.9, 139.4, 136.4, 130.2, 128.8, 127.8, 127.5, 127.4, 127.3, 127.1, 125.3, 123.5, 121.9, 120.0, 116.7, 36.5, 24.2, 22.0.

IR (film): *ν* (cm^{-1}) 3103, 1751, 1748, 1511, 1498, 1388, 1365, 1300, 1227, 1078, 993, 755, 739, 480.

HRMS (ESI-TOF, m/z) calculated for $C_{24}H_{22}NO^+$ $[M+H]^+$ 340.1696, found 340.1703.

1-(3-(1-([1,1'-biphenyl]-4-yl)ethyl)-4-fluoro-1*H*-indol-1-yl)ethan-1-one (38)

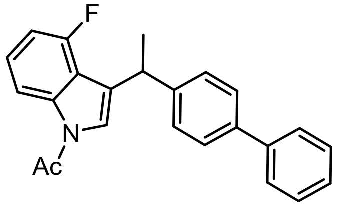

A Schlenk tube (10 mL) was charged with 1-(4-fluoro-1*H*-indol-1-yl)ethan-1-one (**1zi**, 53.1 mg, 0.30 mmol), 4-ethylbiphenyl (**2a**, 164.0 mg, 0.90 mmol), $FeCl_3 \cdot 6H_2O$ (16.2 mg, 0.060 mmol) and CH_2Cl_2 (0.75 mL). The mixture was degassed *via* three freeze-pump-thaw cycles, then filled with dry air. The Schlenk tube was positioned approximately 5 cm away from a 50 W LED lamp (λ_{max} = 410 nm). After being stirred at 25 ℃ for 48 h, the reaction mixture was concentrated to dryness. Purification using silica gel column chromatography (elution with PE:DCM = 2:1) gave the pure product **38** as a light yellow oil (68.6 mg, 0.192 mmol, 64% yield).

^{1}H NMR (500 MHz, $CDCl_3$) δ 8.30 (d, *J* = 7.5 Hz, 1H), 7.64 (d, *J* = 7.7 Hz, 2H), 7.59 (d, *J* = 8.0 Hz, 2H), 7.47 (t, *J* = 7.6 Hz, 2H), 7.42 (d, *J* = 7.9 Hz, 2H), 7.37 (t, *J* = 7.3 Hz, 1H), 7.31 – 7.28 (m, 1H), 7.19 (s, 1H), 6.94 (dd, *J* = 10.2, 8.5 Hz, 1H), 4.61 (q, *J* = 6.9 Hz, 1H), 2.65 (s, 3H), 1.78 (d, *J* = 7.1 Hz, 3H).

^{13}C NMR (126 MHz, $CDCl_3$) δ 168.6, 156.4, 144.3, 141.0, 139.3, 138.4 (d, $J_{C\text{-}F}$ = 40.0 Hz), 128.8, 127.9, 127.2, 127.2, 127.1, 126.6, 126.2, 121.9, 118.5 (d, $J_{C\text{-}F}$ = 75.0 Hz), 112.8, 109.6 (d, $J_{C\text{-}F}$ = 75.0 Hz), 37.0, 24.2, 22.3.

IR (film): *v* (cm^{-1}) 3133, 1762, 1750, 1563, 1505, 1383, 1375, 1290, 1287, 1118, 1074, 988, 753, 750, 741, 550.

HRMS (ESI-TOF, m/z) calculated for $C_{24}H_{21}FNO^+$ $[M+H]^+$ 358.1602, found 358.1599.

1-(3-(1-([1,1'-biphenyl]-4-yl)ethyl)-4-chloro-1*H*-indol-1-yl)ethan-1-one (39)

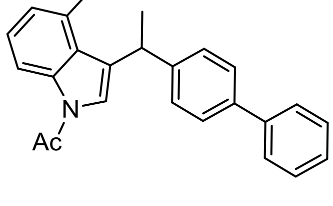

A Schlenk tube (10 mL) was charged with 1-(4-chloro-1*H*-indol-1-yl)ethan-1-one (**1zj**, 57.9 mg, 0.30 mmol), 4-ethylbiphenyl (**2a**, 164.0 mg, 0.90 mmol), $FeCl_3 \cdot 6H_2O$ (16.2 mg, 0.060 mmol) and CH_2Cl_2 (0.75 mL). The mixture was degassed *via* three freeze-pump-thaw cycles, then filled with dry air. The Schlenk tube was positioned approximately 5 cm away from a 50 W LED lamp (λ_{max} = 410 nm). After being stirred at 25 ℃ for 48 h, the reaction mixture was

concentrated to dryness. Purification using silica gel column chromatography (elution with PE:DCM = 2:1) gave the pure product **39** as a light yellow oil (78.4 mg, 0.210 mmol, 70% yield).

^{1}H NMR (500 MHz, $CDCl_3$) δ 8.46 (d, *J* = 7.8 Hz, 1H), 7.60 (d, *J* = 7.3 Hz, 2H), 7.55 (d, *J* = 8.2 Hz, 2H), 7.43 (t, *J* = 7.7 Hz, 2H), 7.36 – 7.31 (m, 3H), 7.25 – 7.19 (m, 3H), 5.01 (q, *J* = 7.0 Hz, 1H), 2.61 (s, 3H), 1.74 (d, *J* = 7.1 Hz, 3H).

^{13}C NMR (126 MHz, $CDCl_3$) δ 168.4, 144.9, 141.0, 139.1, 137.8, 128.8, 128.1, 127.7, 127.2, 127.1, 126.9, 126.5, 126.0, 125.1, 123.7, 115.4, 36.2, 24.2, 23.3.

IR (film): *v* (cm^{-1}) 3133, 1762, 1750, 1563, 1505, 1383, 1375, 1290, 1287, 1118, 1074, 988, 753, 750, 741, 550.

HRMS (ESI-TOF, m/z) calculated for $C_{24}H_{21}ClNO^+$ $[M+H]^+$ 374.1306, found 374.1310.

1-(3-(1-([1,1'-biphenyl]-4-yl)ethyl)-4-bromo-1*H*-indol-1-yl)ethan-1-one (40)

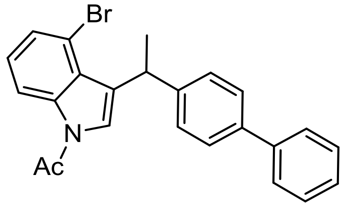

A Schlenk tube (10 mL) was charged with 1-(4-bromo-1*H*-indol-1-yl)ethan-1-one (**1zk**, 71.1 mg, 0.30 mmol), 4-ethylbiphenyl (**2a**, 164.0 mg, 0.90 mmol), $FeCl_3 \cdot 6H_2O$ (16.2 mg, 0.060 mmol) and CH_2Cl_2 (0.75 mL). The mixture was degassed *via* three freeze-pump-thaw cycles, then filled with dry air. The Schlenk tube was positioned approximately 5 cm away from a 50 W LED lamp (λ_{max} = 410 nm). After being stirred at 25 ℃ for 48 h, the reaction mixture was concentrated to dryness. Purification using silica gel column chromatography (elution with PE:DCM = 2:1) gave the pure product **40** as a light yellow oil (90.0 mg, 0.216 mmol, 72% yield).

^{1}H NMR (500 MHz, $CDCl_3$) δ 8.48 (d, *J* = 8.3 Hz, 1H), 7.54 (d, *J* = 7.6 Hz, 2H), 7.49 (d, *J* = 8.2 Hz, 2H), 7.35 (dd, *J* = 8.2, 7.4 Hz, 3H), 7.28 (d, *J* = 8.1 Hz, 3H), 7.17 (s, 1H), 7.10 (t, *J* = 8.1 Hz, 1H), 5.06 (q, *J* = 7.0 Hz, 1H), 2.51 (s, 3H), 1.68 (d, *J* = 7.1 Hz, 3H).

^{13}C NMR (126 MHz, $CDCl_3$) δ 168.3, 144.8, 140.9, 139.0, 137.7, 128.8, 128.6, 128.2, 128.1, 127.9, 127.2, 127.1, 127.0, 126.2, 124.1, 115.9, 114.2, 35.7, 24.1, 23.3.

IR (film): *v* (cm^{-1}) 3099, 1801, 1766, 1569, 1522, 1381, 1352, 1287, 1279, 1101, 1032, 996, 773, 741, 670.

HRMS (ESI-TOF, m/z) calculated for $C_{24}H_{21}BrNO^+$ $[M+H]^+$ 418.0801, found 418.0800.

1-(3-(1-([1,1'-biphenyl]-4-yl)ethyl)-4-methyl-1*H*-indol-1-yl)ethan-1-one (41)

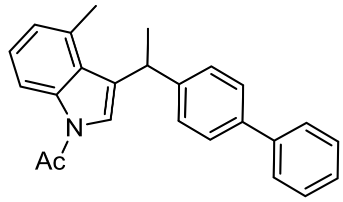

A Schlenk tube (10 mL) was charged with 1-(5-methyl-1*H*-indol-1-yl)ethan-1-one (**1zl**, 51.9 mg, 0.30 mmol), 4-ethylbiphenyl (**2a**, 164.0 mg, 0.90 mmol), $FeCl_3 \cdot 6H_2O$ (16.2 mg, 0.060 mmol) and CH_2Cl_2 (0.75 mL). The mixture was degassed *via* three freeze-pump-thaw cycles, then filled with dry air. The Schlenk tube was positioned approximately 5 cm away from a 50 W LED lamp (λ_{max} = 410 nm). After being stirred at 25 ℃ for 48 h, the reaction mixture was concentrated to dryness. Purification using silica gel column chromatography (elution with PE:DCM = 2:1) gave the pure product **41** as a light yellow oil (72.0 mg, 0.203 mmol, 68% yield).

^{1}H NMR (500 MHz, $CDCl_3$) δ 8.42 (d, *J* = 7.6 Hz, 1H), 7.56 (d, *J* = 7.2 Hz, 2H), 7.55 (d, *J* = 8.2 Hz, 2H), 7.48 (d, *J* = 7.5 Hz, 2H), 7.37 (t, *J* = 6.7 Hz, 2H), 7.29 (dd, *J* = 10.7, 2.5 Hz, 3H), 7.25 (d, J = 8.0 Hz, 1H), 6.99 (d, *J* = 7.3 Hz, 1H), 2.71 (s, 3H), 2.45 (s, 3H), 1.76 (d, *J* = 7.1 Hz, 3H).

^{13}C NMR (126 MHz, $CDCl_3$) δ 168.6, 145.5, 140.9, 139.2, 137.0, 131.3, 128.9, 127.9, 127.7, 127.4, 127.3, 127.1, 125.9, 125.4, 122.8, 114.5, 37.4, 24.4, 24.0, 20.5.

IR (film): *v* (cm^{-1}) 3406, 3104, 3034, 2903, 1771, 1584, 1436, 1411, 1356, 1238, 1081, 1033, 895, 721, 502.

HRMS (ESI-TOF, m/z) calculated for $C_{25}H_{24}NO^+$ $[M+H]^+$ 354.1852, found 354.1857.

1-(3-(1-([1,1'-biphenyl]-4-yl)ethyl)-5-bromo-1*H*-indol-1-yl)ethan-1-one (42)

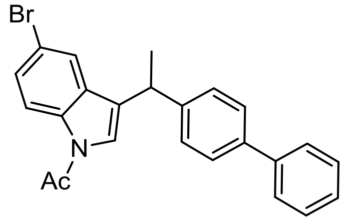

A Schlenk tube (10 mL) was charged with 1-(5-bromo-1*H*-indol-1-yl)ethan-1-one (**1zm**, 71.1 mg, 0.30 mmol), 4-ethylbiphenyl (**2a**, 164.0 mg, 0.90 mmol), $FeCl_3 \cdot 6H_2O$ (16.2 mg, 0.060 mmol) and CH_2Cl_2 (0.75 mL). The mixture was degassed *via* three freeze-pump-thaw cycles, then filled with dry air. The Schlenk tube was positioned approximately 5 cm away from a 50 W LED lamp (λ_{max} = 410 nm). After being stirred at 25 ℃ for 48 h, the reaction mixture was concentrated to dryness. Purification using silica gel column chromatography (elution with PE:DCM = 2:1) gave the pure product **42** as a light yellow oil (91.3 mg, 0.219 mmol, 73%

yield).

^{1}H NMR (500 MHz, $CDCl_3$) δ 8.60 (d, *J* = 8.3 Hz, 1H), 7.66 (d, *J* = 7.3 Hz, 2H), 7.61 (d, *J* = 8.2 Hz, 2H), 7.48 (t, *J* = 7.9 Hz, 3H), 7.40 (d, *J* = 8.0 Hz, 3H), 7.29 (s, 1H), 7.22 (t, *J* = 8.1 Hz, 1H), 5.19 (q, *J* = 7.0 Hz, 1H), 2.63 (s, 3H), 1.80 (d, *J* = 7.1 Hz, 3H).

^{13}C NMR (126 MHz, $CDCl_3$) δ 168.3, 144.8, 140.9, 139.0, 137.7, 128.8, 128.6, 128.1, 128.1, 127.9, 127.2, 127.1, 127.0, 126.2, 124.1, 115.9, 114.2, 35.7, 24.1, 23.3.

IR (film): *ν* (cm^{-1}) 3413, 3108, 2923, 1571, 1569, 1436, 1463, 1642, 1109, 1081, 929, 883, 733, 506.

HRMS (ESI-TOF, m/z) calculated for $C_{24}H_{21}BrNO^+$ $[M+H]^+$ 418.0801, found 418.0817.

2-(1-([1,1'-biphenyl]-4-yl)ethyl)-1-tosyl-1*H*-pyrrole (43)

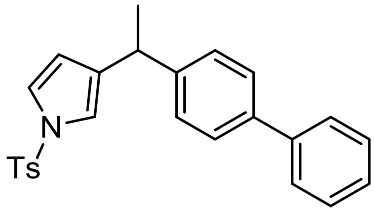

A Schlenk tube (10 mL) was charged with 1-tosyl-1*H*-pyrrole (**1zn**, 66.3 mg, 0.30 mmol), 4-ethylbiphenyl (**2a**, 164.0 mg, 0.90 mmol), $FeCl_3$ $6H_2O$ (16.2 mg, 0.060 mmol) and CH_2Cl_2 (0.75 mL). The mixture was degassed *via* three freeze-pump-thaw cycles, then filled with dry air. The Schlenk tube was positioned approximately 5 cm away from a 50 W LED lamp (λ_{max} = 410 nm). After being stirred at 25 ℃ for 48 h, the reaction mixture was concentrated to dryness. Purification using silica gel column chromatography (elution with PE:DCM = 2:1) gave the pure product **43** as a yellow oil (54.1 mg, 0.135 mmol, 45% yield).

^{1}H NMR (500 MHz, $CDCl_3$) δ 7.54 – 7.51 (m, 2H), 7.43 (t, *J* = 7.7 Hz, 2H), 7.36 – 7.32 (m, 2H), 7.29 (d, *J* = 8.2 Hz, 2H), 7.25 (d, *J* = 8.3 Hz, 2H), 6.97 (t, *J* = 7.9 Hz, 4H), 6.31 (t, *J* = 3.4 Hz, 1H), 6.27 (s, 1H), 4.72 (q, *J* = 7.1 Hz, 1H), 2.19 (s, 3H), 1.53 (d, *J* = 7.2 Hz, 3H).

^{13}C NMR (126 MHz, $CDCl_3$) δ 144.2, 144.2, 141.0, 139.1, 138.9, 136.3, 129.6, 128.9, 127.9, 127.2, 127.0, 126.9, 126.7, 123.1, 112. 7, 111.2, 36.9, 23.9, 21.5.

IR (film): *ν* (cm^{-1}) 3111, 3097, 1750, 1662, 1500, 1342, 1286, 1084, 1072, 1002, 975, 959, 742, 662, 511.

HRMS (ESI-TOF, m/z) calculated for $C_{25}H_{24}NO_2S^+$ $[M+H]^+$ 402.1522, found 402.1530.

1-phenoxy-4-(1-phenylethyl)benzene (44)

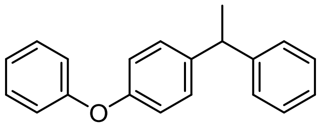

A Schlenk tube (10 mL) was charged with diphenyl ether (**1d**, 51.1 mg, 0.30 mmol), ethylbenzene (**2b**, 95.6 mg, 0.90 mmol), $FeCl_3$ $6H_2O$ (16.2 mg, 0.060 mmol) and $(CHCl_2)_2$ (0.75 mL). The mixture was degassed *via* three freeze-pump-thaw cycles, then filled with dry air. The Schlenk tube was positioned approximately 5 cm away from a 50 W LED lamp (λ_{max} = 410 nm). After being stirred at 25 ℃ for 48 h, the reaction mixture was concentrated to dryness. Purification using silica gel column chromatography (elution with PE:DCM = 5:1) gave the pure product **44** as a colorless oil (57.5 mg, 0.209 mmol, 70% yield).

^{1}H NMR (500 MHz, $CDCl_3$) δ 7.36 (q, *J* = 7.8 Hz, 4H), 7.29 (t, *J* = 7.6 Hz, 2H), 7.23 (d, *J* = 8.5 Hz, 3H), 7.13 (t, *J* = 7.2 Hz, 1H), 7.05 (d, *J* = 8.3 Hz, 2H), 6.98 (d, *J* = 8.4 Hz, 2H), 4.19 (q, *J* = 7.1 Hz, 1H), 1.69 (d, *J* = 7.2 Hz, 3H).

^{13}C NMR (126 MHz, $CDCl_3$) δ 157.6, 155.4, 146.6, 141.5, 129.8, 128.9, 128.5, 127.7, 126.2, 123.2, 119.0, 118.8, 44.3, 22.2.

IR (film): *v* (cm^{-1}) 3110, 3084, 3074, 2963, 2877, 1981, 1496, 1488, 1336, 1321, 1240, 1076, 1031, 964, 881, 492, 480.

HRMS (ESI-TOF, m/z) calculated for $C_{20}H_{19}O^+$ $[M+H]^+$ 275.1430, found 275.1434.

1-ethyl-4-(1-(4-phenoxyphenyl)ethyl)benzene (45)

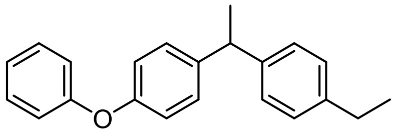

A Schlenk tube (10 mL) was charged with diphenyl ether (**1d**, 51.1 mg, 0.30 mmol), 1,4-diethylbenzene (**2c,** 120.8 mg, 0.90 mmol), $FeCl_3$ $6H_2O$ (16.2 mg, 0.060 mmol) and $(CHCl_2)_2$ (0.75 mL). The mixture was degassed *via* three freeze-pump-thaw cycles, then filled with dry air. The Schlenk tube was positioned approximately 5 cm away from a 50 W LED lamp (λ_{max} = 410 nm). After being stirred at 25 ℃ for 48 h, the reaction mixture was concentrated to dryness. Purification using silica gel column chromatography (elution with PE:DCM = 5:1) gave the pure product **45** as a colorless oil (67.0 mg, 0.222 mmol, 74% yield).

^{1}H NMR (500 MHz, $CDCl_3$) δ 7.28 (m, 2H), 7.16 (d, *J* = 8.5 Hz, 2H), 7.14 – 7.10 (m, 4H), 7.04 (t, *J* = 7.4 Hz, 1H), 6.97 (d, *J* = 7.7 Hz, 2H), 6.94 – 6.88 (m, 2H), 4.09 (q, *J* = 7.2 Hz, 1H), 2.60 (q, *J* = 7.6 Hz, 2H), 1.60 (d, *J* = 7.2 Hz, 3H), 1.21 (t, *J* = 7.6 Hz, 3H).

^{13}C NMR (126 MHz, $CDCl_3$) δ 157.6, 155.3, 143.8, 142.0, 141.7, 129.8, 128.9, 128.0, 127.6, 123.1, 118.9, 118.8, 43.9, 28.5, 22.2, 15.7.

IR (film): *ν* (cm^{-1}) 3134, 3091, 3078, 2929, 1790, 1673, 1516, 1312, 1234, 1022, 985, 971, 943, 800, 611, 547.

HRMS (ESI-TOF, m/z) calculated for $C_{22}H_{23}O^+$ $[M+H]^+$ 303.1743, found 303.1737.

1-fluoro-4-(1-(4-phenoxyphenyl)ethyl)benzene (46)

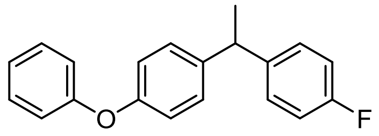

A Schlenk tube (10 mL) was charged with diphenyl ether (**1d**, 51.1 mg, 0.30 mmol), 1-ethyl-4-fluorobenzene (**2d**, 111.7 mg, 0.90 mmol), $FeCl_3$ $6H_2O$ (16.2 mg, 0.060 mmol) and $(CHCl_2)_2$ (0.75 mL). The mixture was degassed *via* three freeze-pump-thaw cycles, then filled with dry air. The Schlenk tube was positioned approximately 5 cm away from a 50 W LED lamp (λ_{max} = 410 nm). After being stirred at 25 ℃ for 48 h, the reaction mixture was concentrated to dryness. Purification using silica gel column chromatography (elution with PE:DCM = 5:1) gave the pure product **46** as a colorless oil (60.4 mg, 0.207 mmol, 69% yield).

^{1}H NMR (500 MHz, $CDCl_3$) δ 7.34 (t, *J* = 7.9 Hz, 2H), 7.21 – 7.16 (m, 4H), 7.10 (t, *J* = 7.4 Hz, 1H), 7.07 – 6.92 (m, 6H), 4.14 (q, *J* = 7.2 Hz, 1H), 1.63 (d, *J* = 7.2 Hz, 3H).

^{13}C NMR (126 MHz, $CDCl_3$) δ 162.4, 160.4, 157.5, 155.6, 142.2, 141.2, 129.8 (d, $J_{C\text{-}F}$ = 30.0 Hz), 129.1, 128.8, 123.2, 118.9 (d, $J_{C\text{-}F}$ = 45.0 Hz), 115.3 (d, $J_{C\text{-}F}$ = 85.0 Hz), 43.5, 22.3.

IR (film): *ν* (cm^{-1}) 3066, 3055, 1597, 1526, 1501, 1476, 1342, 1286, 1223, 1058, 1032, 1011, 975, 961, 899, 742, 519, 481.

HRMS (ESI-TOF, m/z) calculated for $C_{20}H_{18}FO^+$ $[M+H]^+$ 293.1336, found 293.1344.

1-chloro-4-(1-(4-phenoxyphenyl)ethyl)benzene (47)

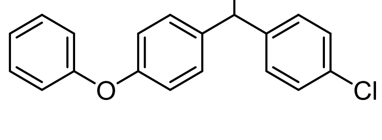

A Schlenk tube (10 mL) was charged with diphenyl ether (**1d**, 51.1 mg, 0.30 mmol), 1-chloro-4-ethylbenzene (**2e**, 126.5 mg, 0.90 mmol), $FeCl_3$ $6H_2O$ (16.2 mg, 0.060 mmol) and $(CHCl_2)_2$ (0.75 mL). The mixture was degassed *via* three freeze-pump-thaw cycles, then filled with dry air. The Schlenk tube was positioned approximately 5 cm away from a 50 W LED lamp (λ_{max} = 410 nm). After being stirred at 25 ℃ for 48 h, the reaction mixture was

concentrated to dryness. Purification using silica gel column chromatography (elution with PE:DCM = 5:1) gave the pure product **47** as a colorless oil (62.9 mg, 0.204 mmol, 68% yield).

^{1}H NMR (500 MHz, $CDCl_3$) δ 7.34 (t, *J* = 7.9 Hz, 2H), 7.28 (d, *J* = 8.4 Hz, 2H), 7.19 – 7.16 (m, 4H), 7.11 (t, *J* = 7.4 Hz, 1H), 7.02 (d, *J* = 8.0 Hz, 2H), 6.96 (d, *J* = 8.5 Hz, 2H), 4.13 (q, *J* = 7.2 Hz, 1H), 1.64 (d, *J* = 7.2 Hz, 3H).

^{13}C NMR (126 MHz, $CDCl_3$) δ 157.5, 155.6, 145.0, 140.8, 131.9, 129.8, 129.1, 128.9, 128.6, 123.3, 119.0, 118.9, 43.7, 22.1.

IR (film): *v* (cm^{-1}) 3081, 3072, 3042, 3019, 1861, 1788, 1584, 1532, 1446, 1377, 1187, 1120, 1044, 1024, 1014, 935, 921, 702, 523, 469.

HRMS (ESI-TOF, m/z) calculated for $C_{20}H_{18}ClO^+$ $[M+H]^+$ 309.1041, found 309.1038.

1-bromo-4-(1-(4-phenoxyphenyl)ethyl)benzene (48)

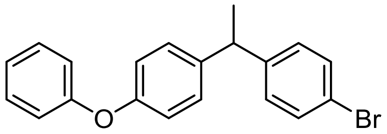

A Schlenk tube (10 mL) was charged with diphenyl ether (**1d**, 51.1 mg, 0.30 mmol), 4-bromoethylbenzene (**2f**, 166.6 mg, 0.90 mmol), $FeCl_3$ $6H_2O$ (16.2 mg, 0.060 mmol) and DCE (0.75 mL). The mixture was degassed *via* three freeze-pump-thaw cycles, then filled with dry air. The Schlenk tube was positioned approximately 5 cm away from a 50 W LED lamp (λ_{max} = 410 nm). After being stirred at 25 ℃ for 48 h, the reaction mixture was concentrated to dryness. Purification using silica gel column chromatography (elution with PE:DCM = 5:1) gave the pure product **48** as a colorless oil (73.9 mg, 0.210 mmol, 70% yield).

^{1}H NMR (500 MHz, $CDCl_3$) δ 7.41 – 7.38 (m, 2H), 7.30 (m, 2H), 7.15 – 7.12 (m, 2H), 7.10 – 7.07 (m, 3H), 7.01 – 6.97 (m, 2H), 6.94 – 6.91 (m, 2H), 4.08 (q, *J* = 7.2 Hz, 1H), 1.60 (d, *J* = 7.2 Hz, 3H).

^{13}C NMR (126 MHz, $CDCl_3$) δ 157.4, 155.6, 145.5, 140.7, 131.6, 129.8, 129.5, 128.8, 123.2, 120.0, 119.0, 118.9, 43.7, 22.0.

IR (film): *v* (cm^{-1}) 3079, 3065, 3044, 3003, 1956, 1862, 1790, 1330, 1247, 1002, 866, 830, 683, 672, 544, 457.

HRMS (ESI-TOF, m/z) calculated for $C_{20}H_{18}BrO^+$ $[M+H]^+$ 353.0536, found 353.0542.

1-bromo-3-(1-(4-phenoxyphenyl)ethyl)benzene (49)

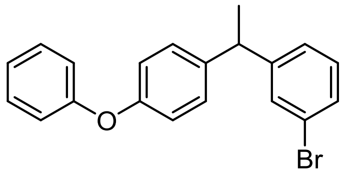

A Schlenk tube (10 mL) was charged with diphenyl ether (**1d**, 51.1 mg, 0.30 mmol), 1-bromo-3-ethylbenzene (**2g**, 166.6 mg, 0.90 mmol), $FeCl_3 \cdot 6H_2O$ (16.2 mg, 0.060 mmol) and DCE (0.75 mL). The mixture was degassed *via* three freeze-pump-thaw cycles, then filled with dry air. The Schlenk tube was positioned approximately 5 cm away from a 50 W LED lamp (λ_{max} = 410 nm). After being stirred at 25 ℃ for 48 h, the reaction mixture was concentrated to dryness. Purification using silica gel column chromatography (elution with PE:DCM = 5:1) gave the pure product **49** as a white solid (76.0 mg, 0.216 mmol, 72% yield).

^{1}H NMR (500 MHz, $CDCl_3$) δ 7.38 – 7.30 (m, 4H), 7.16 (d, *J* = 7.9 Hz, 4H), 7.09 (t, *J* = 7.4 Hz, 1H), 7.03 – 6.98 (m, 2H), 6.96 – 6.92 (m, 2H), 4.10 (q, *J* = 7.2 Hz, 1H), 1.62 (d, *J* = 7.2 Hz, 3H).

^{13}C NMR (126 MHz, $CDCl_3$) δ 157.4, 155.7, 149.0, 140.5, 130.8, 130.1, 129.8, 129.4, 128.9, 126.4, 123.3, 122.7, 119.0, 118.9, 44.0, 22.0.

IR (film): *v* (cm^{-1}) 3068, 3042, 3016, 2001, 1989, 1885, 1401, 1332, 1250, 980, 812, 695, 688, 651, 504, 403.

HRMS (ESI-TOF, m/z) calculated for $C_{20}H_{18}BrO^+$ $[M+H]^+$ 353.0536, found 353.0539.

1-iodo-4-(1-(4-phenoxyphenyl)ethyl)benzene (50)

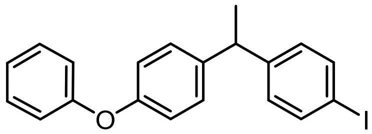

A Schlenk tube (10 mL) was charged with diphenyl ether (**1d**, 51.1 mg, 0.30 mmol), 1-ethyl-4-iodobenzene (**2h**, 208.9 mg, 0.90 mmol), $FeCl_3 \cdot 6H_2O$ (16.2 mg, 0.060 mmol) and DCE (0.75 mL). The mixture was degassed *via* three freeze-pump-thaw cycles, then filled with dry air. The Schlenk tube was positioned approximately 5 cm away from a 50 W LED lamp (λ_{max} = 410 nm). After being stirred at 25 ℃ for 48 h, the reaction mixture was concentrated to dryness. Purification using silica gel column chromatography (elution with PE:DCM = 5:1) gave the pure product **50** as a yellow solid (72.0 mg, 0.180 mmol, 60% yield).

^{1}H NMR (500 MHz, $CDCl_3$) δ 7.61 (d, *J* = 8.2 Hz, 2H), 7.33 (t, *J* = 7.8 Hz, 2H), 7.15 (d, *J* = 8.5 Hz, 2H), 7.09 (t, *J* = 7.3 Hz, 1H), 6.99 (t, *J* = 8.5 Hz, 4H), 6.94 (d, *J* = 8.4 Hz, 2H), 4.09 (q, *J* = 7.2 Hz, 1H), 1.61 (d, *J* = 7.2 Hz, 3H).

^{13}C NMR (126 MHz, $CDCl_3$) δ 157.5, 155.7, 146.3, 140.7, 137.6, 129.8, 128.9, 123.3, 119.0, 118.9, 91.4, 43.8, 22.0.

IR (film): *v* (cm^{-1}) 3477, 3461, 3113, 3059, 2993, 1943, 1883, 1571, 1300, 1188, 1167, 1058, 1015, 996, 903, 835, 667, 614, 460.

HRMS (ESI-TOF, m/z) calculated for $C_{20}H_{18}IO^+$ $[M+H]^+$ 401.0397, found 401.0341.

1-methoxy-4-(1-(4-phenoxyphenyl)ethyl)benzene (51)

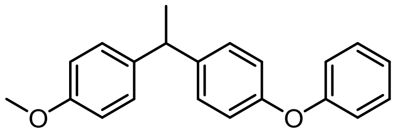

A Schlenk tube (10 mL) was charged with anisole (**1a**, 32.4 mg, 0.30 mmol), 1-ethyl-4-phenoxybenzene (**2i**, 178.3 mg, 0.90 mmol), $FeCl_3$ $6H_2O$ (16.2 mg, 0.060 mmol) and DCE (0.75 mL). The mixture was degassed *via* three freeze-pump-thaw cycles, then filled with dry air. The Schlenk tube was positioned approximately 5 cm away from a 50 W LED lamp (λ_{max} = 410 nm). After being stirred at 25 ℃ for 48 h, the reaction mixture was concentrated to dryness. Purification using silica gel column chromatography (elution with PE:DCM = 6:1) gave the pure product **51** as a colorless oil (50.2 mg, 0.165 mmol, 55% yield).

^{1}H NMR (500 MHz, $CDCl_3$) δ 7.36 – 7.29 (m, 2H), 7.19 – 7.14 (m, 4H), 7.08 (t, *J* = 7.4 Hz, 1H), 7.03 – 6.96 (m, 2H), 6.93 (d, *J* = 8.6 Hz, 2H), 6.85 (d, *J* = 8.7 Hz, 2H), 4.10 (q, *J* = 7.2 Hz, 1H), 3.79 (s, 3H), 1.62 (d, *J* = 7.2 Hz, 3H).

^{13}C NMR (126 MHz, $CDCl_3$) δ 158.0, 157.7, 155.4, 141.9, 138.75, 129.8, 128.9, 128.6, 123.13, 119.0, 118.8, 113.9, 55.4, 43.5, 22.4.

IR (film): *v* (cm^{-1}) 3071, 3066, 3003, 2959, 2944, 2803, 1671, 1450, 1411, 1253, 1107, 1015, 882, 656, 556, 436.

HRMS (ESI-TOF, m/z) calculated for $C_{21}H_{21}O_2^+$ $[M+H]^+$ 305.1536, found 305.1541.

1-phenoxy-4-(1-phenylpropyl)benzene (52)

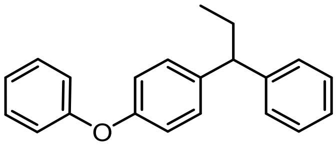

A Schlenk tube (10 mL) was charged with diphenyl ether (**1d**, 51.1 mg, 0.30 mmol), propylbenzene (**2j**, 108.2 mg, 0.90 mmol), $FeCl_3$ $6H_2O$ (16.2 mg, 0.060 mmol) and $(CHCl_2)_2$ (0.75 mL). The mixture was degassed *via* three freeze-pump-thaw cycles, then filled with dry air. The Schlenk tube was positioned approximately 5 cm away from a 50 W LED lamp (λ_{max}

= 410 nm). After being stirred at 25 ℃ for 48 h, the reaction mixture was concentrated to dryness. Purification using silica gel column chromatography (elution with PE:DCM = 5:1) gave the pure product **52** as a colorless oil (61.4 mg, 0.213 mmol, 71% yield).

^{1}H NMR (500 MHz, $CDCl_3$) δ 7.33 – 7.25 (m, 4H), 7.24 – 7.21 (m, 2H), 7.20 – 7.16 (m, 3H), 7.13 – 6.85 (m, 5H), 3.77 (t, *J* = 7.8 Hz, 1H), 2.07 – 2.03 (m, 2H), 0.90 (t, *J* = 7.3 Hz, 3H).

^{13}C NMR (126 MHz, $CDCl_3$) δ 157.6, 155.4, 145.3, 140.3, 129.8, 129.2, 128.5, 128.0, 126.2, 123.1, 119.0, 118.8, 52.7, 28. 9, 12.9.

IR (film): *v* (cm^{-1}) 3108, 3066, 3028, 2978, 2898, 1583, 1445, 1341, 1178, 1111, 1083, 909, 619, 592, 585, 481.

HRMS (ESI-TOF, m/z) calculated for $C_{21}H_{21}O^+$ $[M+H]^+$ 289.1587, found 289.1585.

1-phenoxy-4-(1-phenylbutyl)benzene (53)

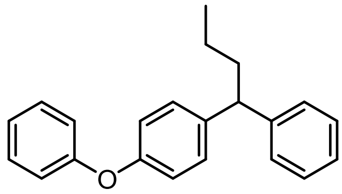

A Schlenk tube (10 mL) was charged with diphenyl ether (**1d**, 51.1 mg, 0.30 mmol), butylbenzene (**2k**, 120.8 mg, 0.90 mmol), $FeCl_3$ $6H_2O$ (16.2 mg, 0.060 mmol) and $(CHCl_2)_2$ (0.75 mL). The mixture was degassed *via* three freeze-pump-thaw cycles, then filled with dry air. The Schlenk tube was positioned approximately 5 cm away from a 50 W LED lamp (λ_{max} = 410 nm). After being stirred at 25 ℃ for 48 h, the reaction mixture was concentrated to dryness. Purification using silica gel column chromatography (elution with PE:DCM = 5:1) gave the pure product **53** as a white solid (63.4 mg, 0.210 mmol, 70% yield).

^{1}H NMR (500 MHz, $CDCl_3$) δ 7.32 – 7.26 (m, 4H), 7.23 (d, *J* = 7.2 Hz, 2H), 7.20 – 7.16 (m, 3H), 7.06 (t, *J* = 7.4 Hz, 1H), 6.94 (m, 4H), 3.89 (t, *J* = 7.8 Hz, 1H), 2.00 (d, *J* = 7.7 Hz, 2H), 1.30 – 1.27 (m, 2H), 0.92 (t, *J* = 7.3 Hz, 3H).

^{13}C NMR (126 MHz, $CDCl_3$) δ 157.6, 155.4, 145.5, 140.5, 129.8, 129.2, 128.5, 128.0, 126.2, 123.1, 119.0, 118.8, 50.5, 38.2, 21.3, 14.2.

IR (film): *v* (cm^{-1}) 3108, 3087, 3029, 3003, 2873, 2860, 1953, 1800, 1604, 1583, 1489, 1379, 1364, 1188, 1132, 955, 934, 779, 746, 661, 570, 503.

HRMS (ESI-TOF, m/z) calculated for $C_{22}H_{23}O^+$ $[M+H]^+$ 303.1743, found 303.1749.

4-(1-(4-phenoxyphenyl)butyl)-1,1'-biphenyl (54)

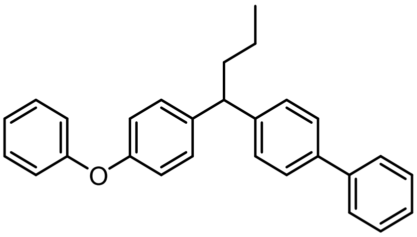

A Schlenk tube (10 mL) was charged with diphenyl ether (**1d**, 51.1 mg, 0.30 mmol), 4-butyl-1,1'-biphenyl (**2l**, 189.3 mg, 0.90 mmol), $FeCl_3$ $6H_2O$ (16.2 mg, 0.060 mmol) and $(CHCl_2)_2$ (0.75 mL). The mixture was degassed *via* three freeze-pump-thaw cycles, then filled with dry air. The Schlenk tube was positioned approximately 5 cm away from a 50 W LED lamp (λ_{max} = 410 nm). After being stirred at 25 ℃ for 48 h, the reaction mixture was concentrated to dryness. Purification using silica gel column chromatography (elution with PE:DCM = 5:1) gave the pure product **54** as a colorless oil (73.7 mg, 0.195 mmol, 65% yield).

^{1}H NMR (500 MHz, $CDCl_3$) δ 7.57 – 7.54 (m, 2H), 7.50 (d, *J* = 8.2 Hz, 2H), 7.39 (t, *J* = 7.7 Hz, 2H), 7.29 (m, 5H), 7.21 (d, *J* = 8.7 Hz, 2H), 7.05 (t, *J* = 7.4 Hz, 1H), 7.00 – 6.96 (m, 2H), 6.93 (d, *J* = 8.6 Hz, 2H), 3.92 (t, *J* = 7.8 Hz, 1H), 2.06 – 2.00 (m, 2H), 1.34 – 1.29 (m, 2H), 0.94 (t, *J* = 7.3 Hz, 3H).

^{13}C NMR (126 MHz, $CDCl_3$) δ 157.5, 155.5, 144.6, 141.1, 140.3, 139.1, 129.8, 129.2, 128.8, 128.3, 127.3, 127.2, 127.1, 123.2, 119.0, 118.9, 50.2, 38.2, 21.3, 14.2.

IR (film): *v* (cm^{-1}) 3091, 3065, 3030, 2888, 2812, 1942, 1838, 1593, 1558, 1454, 1342, 1009, 1130, 935, 759, 733, 694, 510.

HRMS (ESI-TOF, m/z) calculated for $C_{28}H_{27}O^+$ $[M+H]^+$ 379.2056, found 379.2062.

1-(2-methyl-1-phenylpropyl)-4-phenoxybenzene (55)

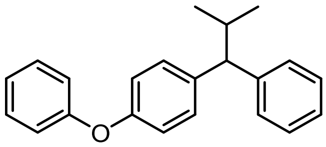

A Schlenk tube (10 mL) was charged with diphenyl ether (**1d**, 51.1 mg, 0.30 mmol), isobutylbenzene (**2m**, 120.8 mg, 0.90 mmol), $FeCl_3$ $6H_2O$ (16.2 mg, 0.060 mmol) and DCE (0.75 mL). The mixture was degassed *via* three freeze-pump-thaw cycles, then filled with dry air. The Schlenk tube was positioned approximately 5 cm away from a 50 W LED lamp (λ_{max} = 410 nm). After being stirred at 25 ℃ for 48 h, the reaction mixture was concentrated to dryness. Purification using silica gel column chromatography (elution with PE:DCM = 5:1) gave the pure product **55** as a white solid (59.8 mg, 0.198 mmol, 66% yield).

^{1}**H NMR** (500 MHz, $CDCl_3$) δ 7.29 (m, 2H), 7.27 (s, 2H), 7.26 (d, *J* = 4.0 Hz, 2H), 7.24 – 7.22 (m, 2H), 7.14 (m, 1H), 7.07 – 7.03 (m, 1H), 6.97 – 6.94 (m, 2H), 6.91 – 6.88 (m, 2H), 3.38 (d, *J* = 10.8 Hz, 1H), 2.44 (m, 1H), 0.89 (d, *J* = 6.5 Hz, 3H), 0.87 (d, *J* = 6.5 Hz, 3H).

^{13}**C NMR** (126 MHz, $CDCl_3$) δ 157.6, 155.3, 145.1, 140.1, 129.8, 129.3, 128.6, 128.1, 126.1, 123.1, 119.0, 118.8, 60.3, 32.2, 22.0, 22.0.

IR (film): *ν* (cm^{-1}) 3086, 3065, 3028, 2924, 2849, 1665, 1375, 1324, 1178, 1168, 1122, 1058, 933, 922, 784, 737, 618, 591, 481.

HRMS (ESI-TOF, m/z) calculated for $C_{22}H_{23}O^+$ $[M+H]^+$ 303.1743, found 303.1726.

((4-phenoxyphenyl)methylene)dibenzene (56)

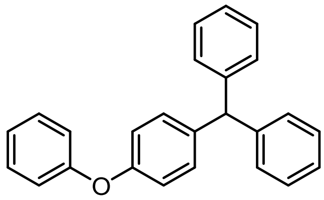

A Schlenk tube (10 mL) was charged with diphenyl ether (**1d**, 51.1 mg, 0.30 mmol), diphenylmethanel (**2n**, 151.4 mg, 0.90 mmol), $FeCl_3 \cdot 6H_2O$ (16.2 mg, 0.060 mmol) and $(CHCl_2)_2$ (0.75 mL). The mixture was degassed *via* three freeze-pump-thaw cycles, then filled with dry air. The Schlenk tube was positioned approximately 5 cm away from a 50 W LED lamp (λ_{max} = 410 nm). After being stirred at 25 ℃ for 48 h, the reaction mixture was concentrated to dryness. Purification using silica gel column chromatography (elution with PE:DCM = 5:1) gave the pure product **56** as a white solid (72.6 mg, 0.216 mmol, 72% yield).

^{1}**H NMR** (500 MHz, $CDCl_3$) δ 7.35 – 7.31 (m, 3H), 7.30 (d, *J* = 7.7 Hz, 3H), 7.23 (t, *J* = 7.3 Hz, 2H), 7.14 (d, *J* = 7.2 Hz, 4H), 7.11 – 7.06 (m, 3H), 7.04 – 7.00 (m, 2H), 6.96 – 6.92 (m, 2H), 5.55 (s, 1H).

^{13}**C NMR** (126 MHz, $CDCl_3$) δ 157.4, 155.8, 144.1, 138.9, 130.8, 129.8, 129.5, 128.5, 126.5, 123.3, 119.00 118.8, 56.3.

IR (film): *ν* (cm^{-1}) 3088, 3066, 3006, 2913, 1959, 1943, 1600, 1547, 1498, 1453, 1337, 1326, 1202, 1183, 1108, 1031, 1003, 933, 890, 632, 608, 464.

HRMS (ESI-TOF, m/z) calculated for $C_{25}H_{21}O^+$ $[M+H]^+$ 337.1592, found 337.1599.

(1-(4-phenoxyphenyl)ethane-1,2-diyl)dibenzene (57)

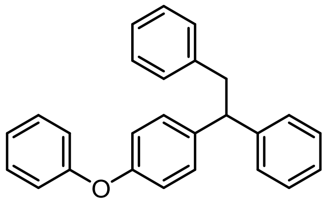

A Schlenk tube (10 mL) was charged with diphenyl ether (**1d**, 51.1 mg, 0.30 mmol), 1,2-diphenylethane (**2o**, 164.0 mg, 0.90 mmol), $FeCl_3$ $6H_2O$ (16.2 mg, 0.060 mmol) and DCE (0.75 mL). The mixture was degassed *via* three freeze-pump-thaw cycles, then filled with dry air. The Schlenk tube was positioned approximately 5 cm away from a 50 W LED lamp (λ_{max} = 410 nm). After being stirred at 25 ℃ for 48 h, the reaction mixture was concentrated to dryness. Purification using silica gel column chromatography (elution with PE:DCM = 5:1) gave the pure product **57** as a white solid (69.3 mg, 0.198 mmol, 66% yield).

^{1}H NMR (500 MHz, $CDCl_3$) δ 7.28 – 7.18 (m, 6H), 7.15 – 7.09 (m, 6H), 7.03 (t, *J* = 7.4 Hz, 1H), 7.00 – 6.92 (m, 4H), 6.87 (d, *J* = 8.6 Hz, 2H), 4.19 (t, *J* = 7.8 Hz, 1H), 3.32 (m, 2H).

^{13}C NMR (126 MHz, $CDCl_3$) δ 157.5, 155.5, 144.6, 140.3, 139.6, 129.8, 129.4, 129.2, 128.5, 128.2, 128.1, 126.4, 126.0, 123.1, 119.0, 118.8, 52.6, 42.4.

IR (film): *ν* (cm^{-1}) 3120, 3083, 3058, 3027,2943, 2918, 1953, 1946, 1751, 1146, 1065, 1003, 982, 959, 910, 841, 768, 752, 622, 528, 510.

HRMS (ESI-TOF, m/z) calculated for $C_{26}H_{23}O^+$ $[M+H]^+$ 351.1743, found 351.1752.

1-(3-chloro-1-phenylpropyl)-4-phenoxybenzene (58)

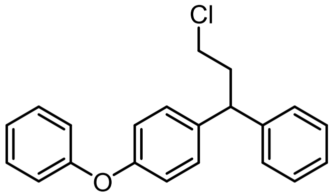

A Schlenk tube (10 mL) was charged diphenyl ether (**1d**, 51.1 mg, 0.30 mmol), (3-chloropropyl)benzene (**2p**, 139.2 mg, 0.90 mmol), $FeCl_3$ $6H_2O$ (16.2 mg, 0.060 mmol) and DCE (0.75 mL). The mixture was degassed *via* three freeze-pump-thaw cycles, then filled with dry air. The Schlenk tube was positioned approximately 5 cm away from a 50 W LED lamp (λ_{max} = 410 nm). After being stirred at 25 ℃ for 48 h, the reaction mixture was concentrated to dryness. Purification using silica gel column chromatography (elution with PE:DCM = 4:1) gave the pure product **58** as a light yellow oil (57.0 mg, 0.177 mmol, 59% yield).

^{1}H NMR (500 MHz, $CDCl_3$) δ 7.33 – 7.28 (m, 4H), 7.24 (d, *J* = 8.2 Hz, 2H), 7.21 (dd, *J* = 11.8, 4.9 Hz, 3H), 7.08 (t, *J* = 7.4 Hz, 1H), 7.00 – 6.96 (m, 2H), 6.95 – 6.91 (m, 2H), 4.20 (t, *J* = 7.8 Hz, 1H), 3.45 (t, *J* = 6.6 Hz, 2H), 2.47 (q, *J* = 5.0 Hz, 2H).

^{13}C NMR (126 MHz, $CDCl_3$) δ 157.3, 155.9, 143.7, 138.5, 129.8, 129.2, 128.8, 128.0, 126.7, 123.3, 119.1, 119.0, 47.3, 43.3, 38.4.

IR (film): *ν* (cm^{-1}) 3108, 3086, 3064, 2942, 2851, 1688, 1606, 1583, 1480, 1443, 1432, 1355, 1290, 1230, 1204, 1180, 1080, 1033, 914, 864, 745, 700, 658, 562.

HRMS (ESI-TOF, m/z) calculated for $C_{21}H_{20}ClO^+$ $[M+H]^+$ 323.1197, found 323.1201.

1-(3-bromo-1-phenylpropyl)-4-phenoxybenzene (59)

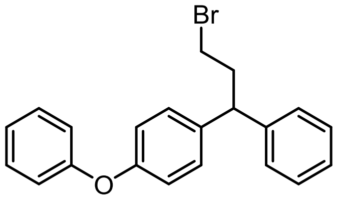

A Schlenk tube (10 mL) was charged diphenyl ether (**1d**, 51.1 mg, 0.30 mmol), (3-bromopropyl)benzene (**2q**, 179.2 mg, 0.90 mmol), $FeCl_3$ $6H_2O$ (16.2 mg, 0.060 mmol) and DCE (0.75 mL). The mixture was degassed *via* three freeze-pump-thaw cycles, then filled with dry air. The Schlenk tube was positioned approximately 5 cm away from a 50 W LED lamp (λ_{max} = 410 nm). After being stirred at 25 ℃ for 48 h, the reaction mixture was concentrated to dryness. Purification using silica gel column chromatography (elution with PE:DCM = 4:1) gave the pure product **59** as a light yellow oil (56.0 mg, 0.153 mmol, 51% yield).

^{1}H NMR (500 MHz, $CDCl_3$) δ 7.33 – 7.29 (m, 4H), 7.25 (d, *J* = 6.4 Hz, 2H), 7.20 (t, *J* = 8.3 Hz, 3H), 7.10 – 7.06 (m, 1H), 7.00 – 6.91 (m, 4H), 4.18 (t, *J* = 7.7 Hz, 1H), 3.32 (t, *J* = 6.7 Hz, 2H), 2.55 (dd, *J* = 14.3, 6.9 Hz, 2H).

^{13}C NMR (126 MHz, $CDCl_3$) δ 157.3, 155.9, 143.6, 138.4, 129.9, 129.2, 128.8, 128.0, 126.7, 123.4, 119.1, 119.0, 48.6, 38.6, 32.1.

IR (film): *v* (cm^{-1}) 3077, 3063, 3004, 2939, 2858, 1616, 1580, 1453, 1428, 1365, 1333, 1244, 1208, 1180, 1062, 1031, 910, 822, 745, 659, 651, 481.

MS (ESI) mass calculated for $C_{21}H_{20}BrO^+$ $[M+H]^+$ 367.0716, found 367.0717.

1-(4-phenoxyphenyl)-2,3-dihydro-1*H*-indene (60)

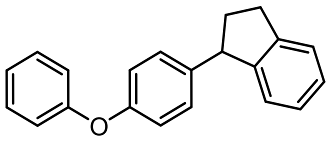

A Schlenk tube (10 mL) was charged diphenyl ether (**1d**, 51.1 mg, 0.30 mmol), indan (**2r**, 106.4 mg, 0.90 mmol), $FeCl_3$ $6H_2O$ (16.2 mg, 0.060 mmol) and DCE (0.75 mL). The mixture was degassed *via* three freeze-pump-thaw cycles, then filled with dry air. The Schlenk tube was positioned approximately 5 cm away from a 50 W LED lamp (λ_{max} = 410 nm). After being stirred at 25 ℃ for 48 h, the reaction mixture was concentrated to dryness. Purification using silica gel column chromatography (elution with PE:DCM = 5:1) gave the pure product **60** as a colorless oil (42.9 mg, 0.150 mmol, 50% yield).

^{1}H NMR (500 MHz, $CDCl_3$) δ 7.42 – 7.27 (m, 3H), 7.23 (t, *J* = 7.3 Hz, 1H), 7.20 – 7.16 (m, 3H), 7.12 (t, *J* = 7.4 Hz, 1H), 7.12 – 6.91 (m, 5H), 4.36 (t, *J* = 8.3 Hz, 1H), 3.08 (m, 1H), 3.02 – 2.95 (m, 1H), 2.62 (m, 1H), 2.11 – 2.05 (m, 1H).

^{13}C NMR (126 MHz, $CDCl_3$) δ 157.6, 155.7, 147.0, 144.4, 140. 5, 129.8, 129.4, 126.7, 126.5, 125.0, 124.5, 123.2, 119.1, 118.8, 51.1, 36.8, 31.9.

IR (film): *ν* (cm^{-1}) 3363, 2922, 2849, 1588, 1488, 1408, 1237, 1166, 1022, 867, 751, 691, 535.

HRMS (ESI-TOF, m/z) calculated for $C_{21}H_{19}O^+$ $[M+H]^+$ 287.1430, found 287.1438.

1-(4-methoxyphenyl)-2,3-dihydro-1*H*-indene (61)

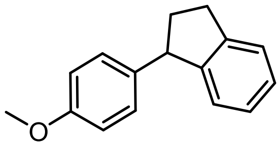

A Schlenk tube (10 mL) was charged with anisole (**1a**, 32.4 mg, 0.30 mmol), indan (**2r**, 106.4 mg, 0.90 mmol), $FeCl_3$ $6H_2O$ (16.2 mg, 0.060 mmol) and DCE (0.75 mL). The mixture was degassed *via* three freeze-pump-thaw cycles, then filled with dry air. The Schlenk tube was positioned approximately 5 cm away from a 50 W LED lamp (λ_{max} = 410 nm). After being stirred at 25 ℃ for 48 h, the reaction mixture was concentrated to dryness. Purification using silica gel column chromatography (elution with PE:DCM = 5:1) gave the pure product **61** as a colorless oil (34.3 mg, 0.153 mmol, 51% yield).

^{1}H NMR (500 MHz, $CDCl_3$) δ 7.31 (d, *J* = 7.4 Hz, 1H), 7.22 – 7.19 (m, 1H), 7.16 (d, *J* = 7.5 Hz, 1H), 7.15 – 7.13 (m, 2H), 6.98 (d, *J* = 7.5 Hz, 1H), 6.90 – 6.87 (m, 2H), 4.32 (t, *J* = 8.3 Hz, 1H), 3.82 (s, 3H), 3.09 – 3.03 (m, 1H), 2.99 – 2.94 (m, 1H), 2.61 – 2.55 (m, 1H), 2.08 – 2.02 (m, 1H).

^{13}C NMR (126 MHz, $CDCl_3$) δ 158.2, 147.3, 144.4, 137.6, 129.2, 126.6, 126.4, 125.0, 124.4, 114.0, 55.4, 51.0, 36.9, 31.9.

IR (film): *ν* (cm^{-1}) 3371, 2920, 2857, 1578, 1473, 1410, 1178, 1013, 865, 741, 676, 551.

MS (ESI) mass calculated for $C_{16}H_{17}O^+$ $[M+H]^+$ 225.1274, found 225.1278.

1-(4-phenoxyphenyl)-1,2,3,4-tetrahydronaphthalene (62)

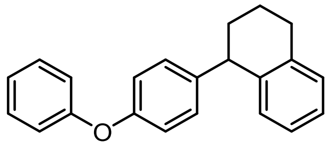

A Schlenk tube (10 mL) was charged diphenyl ether (**1d**, 51.1 mg, 0.30 mmol), 1,2,3,4-tetrahydronaphthalene (**2s**, 119.0 mg, 0.90 mmol), $FeCl_3$ $6H_2O$ (16.2 mg, 0.060 mmol) and DCE (0.75 mL). The mixture was degassed *via* three freeze-pump-thaw cycles, then filled with dry air. The Schlenk tube was positioned approximately 5 cm away from a 50 W LED lamp (λ_{max} = 410 nm). After being stirred at 25 ℃ for 48 h, the reaction mixture was

concentrated to dryness. Purification using silica gel column chromatography (elution with PE:DCM = 5:1) gave the pure product **62** as a white solid (47.7 mg, 0.159 mmol, 53% yield).

^{1}H NMR (500 MHz, $CDCl_3$) δ 7.36 (t, *J* = 7.8 Hz, 2H), 7.17 (d, *J* = 5.5 Hz, 2H), 7.09 (m, 6H), 6.97 (d, *J* = 8.4 Hz, 2H), 6.92 (d, *J* = 7.6 Hz, 1H), 4.15 (t, *J* = 6.6 Hz, 1H), 2.96 – 2.92 (m, 1H), 2.89 (q, *J* = 5.2, 4.5 Hz, 1H), 2.23 – 2.18 (m, 1H), 1.95 – 1.89 (m, 2H), 1.84 – 1.79 (m, 1H).

^{13}C NMR (126 MHz, $CDCl_3$) δ 157.6, 155.4, 142.6, 139.5, 137.7, 130.3, 130.1, 129.8, 129.1, 126.1, 125.8, 123.1, 118.9, 118.8, 45.0, 33.4, 29.9, 21.0.

IR (film): *v* (cm^{-1}) 3363, 2938, 2852, 2841, 1505, 1482, 1455, 1408, 1301, 1223, 1160, 1022, 751, 739, 691, 541.

HRMS (ESI-TOF, m/z) calculated for $C_{22}H_{21}O^+$ $[M+H]^+$ 301.1587, found 301.1581.

1-(1-(4-phenoxyphenyl)ethyl)naphthalene (63)

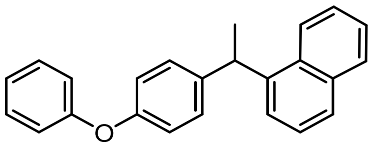

A Schlenk tube (10 mL) was charged with diphenyl ether (**1d**, 51.1 mg, 0.30 mmol), 1-ethylnaphthalene (**2t**, 140.6 mg, 0.90 mmol), $FeCl_3$ $6H_2O$ (16.2 mg, 0.060 mmol) and $(CHCl_2)_2$ (0.75 mL). The mixture was degassed *via* three freeze-pump-thaw cycles, then filled with dry air. The Schlenk tube was positioned approximately 5 cm away from a 50 W LED lamp (λ_{max} = 410 nm). After being stirred at 25 ℃ for 48 h, the reaction mixture was concentrated to dryness. Purification using silica gel column chromatography (elution with PE:DCM = 5:1) gave the pure product **63** as a white solid (63.2 mg, 0.195 mmol, 65% yield).

^{1}H NMR (500 MHz, $CDCl_3$) δ 8.08 – 8.05 (m, 1H), 7.88 – 7.85 (m, 1H), 7.76 (d, *J* = 7.9 Hz, 1H), 7.49 – 7.44 (m, 4H), 7.31 (t, *J* = 7.3 Hz, 2H), 7.20 (d, *J* = 8.1 Hz, 2H), 7.08 (t, *J* = 7.4 Hz, 1H), 6.99 (d, *J* = 8.4 Hz, 2H), 6.94 – 6.90 (m, 2H), 4.93 (q, *J* = 7.0 Hz, 1H), 1.78 (d, *J* = 7.1 Hz, 3H).

^{13}C NMR (126 MHz, $CDCl_3$) δ 157.5, 155.4, 141.7, 141.7, 134.2, 131.8, 129.8, 129.0, 127.2, 126.0, 125.6, 125.5, 124.4, 124.1, 123.2, 119.0, 118.8, 40.0, 22.8.

IR (film): *v* (cm^{-1}) 3502, 3054, 3027, 2965, 2919, 2849, 1662, 1485, 1392, 1181, 841, 766, 738, 697, 570.

HRMS (ESI-TOF, m/z) calculated for $C_{24}H_{21}O^+$ $[M+H]^+$ 325.1587, found 325.1592.

1-phenoxy-4-(2-phenylpropan-2-yl)benzene (64)

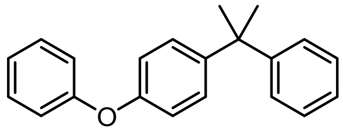

A Schlenk tube (10 mL) was charged with diphenyl ether (**1d**, 51.1 mg, 0.30 mmol), cumene (**2u**, 108.2 mg, 0.90 mmol), $FeCl_3$ $6H_2O$ (16.2 mg, 0.060 mmol) and DCE (0.75 mL). The mixture was degassed *via* three freeze-pump-thaw cycles, then filled with dry air. The Schlenk tube was positioned approximately 5 cm away from a 50 W LED lamp (λ_{max} = 410 nm). After being stirred at 25 ℃ for 48 h, the reaction mixture was concentrated to dryness. Purification using silica gel column chromatography (elution with PE:DCM = 5:1) gave the pure product **64** as a white solid (42.3 mg, 0.147 mmol, 49% yield).

^{1}H NMR (500 MHz,$CDCl_3$) δ 7.35 – 7.30 (m, 3H), 7.28 (d, *J* = 6.9 Hz, 3H), 7.19 (d, *J* = 8.6 Hz, 3H), 7.08 (t, *J* = 7.4 Hz, 1H), 7.01 (d, *J* = 8.0 Hz, 2H), 6.93 – 6.89 (m, 2H), 1.69 (s, 6H).

^{13}C NMR (126 MHz, $CDCl_3$) δ 157.5, 155.1, 150.8, 145.8, 129.8, 128.2, 128.2, 126.9, 125.8, 123.2, 118.9, 118.4, 42.7, 31.0.

IR (film): ν (cm^{-1}) 3085, 3068, 3029, 2927, 2871, 1944, 1867, 1801, 1702, 1632, 1550, 1494, 1466, 1458, 1364, 1323, 1282, 1108, 1050, 931, 906.

HRMS (ESI-TOF, m/z) calculated for $C_{21}H_{21}O^+$ $[M+H]^+$ 289.1587, found 289.1595.

4-(2-(4-methoxyphenyl)propan-2-yl)-1,1'-biphenyl (65)

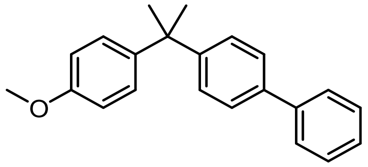

A Schlenk tube (10 mL) was charged with anisole (**1a**, 32.4 mg, 0.30 mmol), 4-isopropylbiphenyl (**2v**, 176.66 mg, 0.90 mmol), $FeCl_3$ $6H_2O$ (16.2 mg, 0.060 mmol) and DCE (0.75 mL). The mixture was degassed *via* three freeze-pump-thaw cycles, then filled with dry air. The Schlenk tube was positioned approximately 5 cm away from a 50 W LED lamp (λ_{max} = 410 nm). After being stirred at 25 ℃ for 48 h, the reaction mixture was concentrated to dryness. Purification using silica gel column chromatography (elution with PE:DCM = 5:1) gave the pure product **65** as a colorless oil (57.1 mg, 0.189 mmol, 63% yield).

^{1}H NMR (500 MHz, $CDCl_3$) δ 7.59 – 7.58 (m, 2H), 7.52 – 7.49 (m, 2H), 7.44 – 7.40 (m, 2H), 7.34 – 7.29 (m, 3H), 7.22 – 7.18 (m, 2H), 6.86 – 6.82 (m, 2H), 3.80 (s, 3H), 1.71 (s, 6H).

^{13}C NMR (126 MHz, $CDCl_3$) δ 157.6, 150.2, 142. 9, 141.1, 138.5, 128.8, 128.0, 127.3, 127.2, 127.1, 126.8, 113.5, 55.4, 42.3, 31.1.

IR (film): *v* (cm^{-1}) 3080, 3057, 2915, 2854, 1913, 1833, 1828, 1673, 1537, 1425, 1402, 1485, 1377, 1351, 1288, 1086, 913, 537.

HRMS (ESI-TOF, m/z) calculated for $C_{22}H_{23}O^+$ $[M+H]^+$ 303.1743, found 303.1749.

4-([1,1'-biphenyl]-4-ylmethyl)phenol (66)

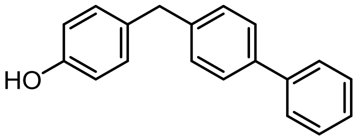

A Schlenk tube (10 mL) was charged with phenol (**1p**, 28.2 mg, 0.30 mmol), 4-methyl-1,1'-biphenyl (**2w**, 151.4 mg, 0.90 mmol), $FeCl_3$ $6H_2O$ (16.2 mg, 0.060 mmol) and $CHCl_3$ (0.75 mL). The mixture was degassed *via* three freeze-pump-thaw cycles, then filled with dry air. The Schlenk tube was positioned approximately 5 cm away from a 50 W LED lamp (λ_{max} = 410 nm). After being stirred at 25 ℃ for 48 h, the reaction mixture was concentrated to dryness. Purification using silica gel column chromatography (elution with PE:DCM = 2:1) gave the pure product **66** as a colorless oil (41.4 mg, 0.159 mmol, 53% yield).

^{1}H NMR (500 MHz, $CDCl_3$) δ 7.61 – 7.59 (m, 2H), 7.54 (d, *J* = 8.1 Hz, 2H), 7.46 (t, *J* = 7.7 Hz, 2H), 7.35 (t, *J* = 7.4 Hz, 1H), 7.27 (d, *J* = 8.3 Hz, 2H), 7.12 (d, *J* = 8.4 Hz, 2H), 6.81 (d, *J* = 8.5 Hz, 2H), 3.98 (s, 2H).

^{13}C NMR (126 MHz, $CDCl_3$) δ 154.1, 141.2, 140.8, 139.1, 133.4, 130.2, 129.3, 128.9, 127.3, 127.2, 127.2, 115.5, 40.8.

IR (film): *v* (cm^{-1}) 3382, 3037, 2918, 2849, 1601, 1514, 1248, 1100, 819, 777, 689, 530.

HRMS (ESI-TOF, m/z) calculated for $C_{19}H_{16}NaO^+$ $[M+Na]^+$ 283.1093, found 283.1100.

4-benzylphenol (67)

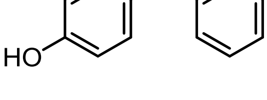

A Schlenk tube (10 mL) was charged with phenol (**1p**, 28.2 mg, 0.30 mmol), toluene (**2x**, 82.9 mg, 0.90 mmol), $FeCl_3$ $6H_2O$ (16.2 mg, 0.060 mmol) and $CHCl_3$ (0.75 mL). The mixture was degassed *via* three freeze-pump-thaw cycles, then filled with dry air. The Schlenk tube was positioned approximately 5 cm away from a 50 W LED lamp (λ_{max} = 410 nm). After being stirred at 25 ℃ for 48 h, the reaction mixture was concentrated to dryness. Purification using silica gel column chromatography (elution with PE:DCM = 2:1) gave the

pure product **67** as a white soild (31.0 mg, 0.168 mmol, 56% yield).

^{1}H NMR (500 MHz, $CDCl_3$) δ 7.36 – 7.31 (m, 2H), 7.26 – 7.21 (m, 3H), 7.11 (d, *J* = 8.4 Hz, 2H), 6.82 – 6.78 (m, 2H), 3.97 (s, 2H).

^{13}C NMR (126 MHz, $CDCl_3$) δ 153.8, 141.5, 133.5, 130.1, 128.8, 128.5, 126.0, 115.3, 41.0.

IR (film): *v* (cm^{-1}) 3241, 3062, 2918, 2690, 1614, 1588, 1511, 1235, 1099, 848, 811, 787, 683.

HRMS (ESI-TOF, m/z) calculated for $C_{13}H_{13}O^+$ $[M+H]^+$ 185.0961, found 185.0966.

1-(4-phenoxyphenyl)-3-(*p*-tolyl)adamantane (68)

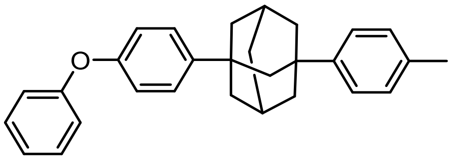

A Schlenk tube (10 mL) was charged with diphenyl ether (**1d**, 51.1 mg, 0.30 mmol), 1-(*p*-tolyl)adamantane (**2y**, 678.5 mg, 3.0 mmol), $FeCl_3$ $6H_2O$ (16.2 mg, 0.060 mmol) and DCE (0.75 mL). The mixture was degassed *via* three freeze-pump-thaw cycles, then filled with dry air. The Schlenk tube was positioned approximately 5 cm away from a 40 W LED lamp (λ_{max} = 370 nm). After being stirred at 80 ℃ for 60 h, the reaction mixture was concentrated to dryness. Purification using silica gel column chromatography (elution with PE) gave the pure product **68** as a colorless oil (61.5 mg, 0.156 mmol, 52% yield).

^{1}H NMR (500 MHz, $CDCl_3$) δ 7.36 (d, *J* = 8.7 Hz, 3H), 7.33 – 7.29 (m, 3H), 7.16 (d, *J* = 8.0 Hz, 2H), 7.09 (t, *J* = 7.4 Hz, 1H), 7.00 (dd, *J* = 17.2, 8.3 Hz, 4H), 2.34 (s, 3H), 2.03 (s, 3H), 1.96 (d, *J* = 2.2 Hz, 10H), 1.80 (s, 4H).

^{13}C NMR (126 MHz, $CDCl_3$) δ 157.7, 155.0, 147.8, 145.9, 135.3, 129.8, 129.0, 126.3, 124.9, 123.1, 118.8, 188.7, 49.4, 42.6, 42.5, 37.1, 37.1, 36.1, 29.8, 21.0.

IR (film): *v* (cm^{-1}) 3935, 3056, 2918, 2903, 2810, 2578, 2483, 1938, 1430, 1365, 1290, 1232, 978, 911, 859, 700.

HRMS (ESI-TOF, m/z) calculated for $C_{29}H_{30}NaO^+$ $[M+Na]^+$ 417.2189, found 417.2200.

1-methyl-4-(1-(4-phenoxyphenyl)ethyl)benzene (69)

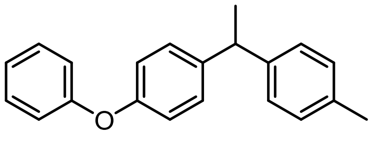

A Schlenk tube (10 mL) was charged with diphenyl ether (**1d**, 51.1 mg, 0.30 mmol), 1-ethyl-4-methylbenzene (**2z**, 108.2 mg, 0.90 mmol), $FeCl_3$ $6H_2O$ (16.2 mg, 0.060 mmol)

and $(CHCl_2)_2$ (0.75 mL). The mixture was degassed *via* three freeze-pump-thaw cycles, then filled with dry air. The Schlenk tube was positioned approximately 5 cm away from a 50 W LED lamp (λ_{max} = 410 nm). After being stirred at 25 ℃ for 48 h, the reaction mixture was concentrated to dryness. Purification using silica gel column chromatography (elution with PE:DCM = 5:1) gave the pure product **69** as a colorless oil (51.9 mg, 0.180 mmol, 60% yield).

^{1}H NMR (500 MHz, $CDCl_3$) δ 7.32 (t, *J* = 7.9 Hz, 2H), 7.19 (d, *J* = 8.4 Hz, 2H), 7.13 (d, *J* = 1.4 Hz, 4H), 7.08 (t, *J* = 7.4 Hz, 1H), 7.02 – 6.98 (m, 2H), 6.93 (d, *J* = 8.6 Hz, 2H), 4.12 (q, *J* = 7.2 Hz, 1H), 2.33 (s, 3H), 1.63 (d, *J* = 7.2 Hz, 3H).

^{13}C NMR (126 MHz, $CDCl_3$) δ 157.7, 155.4, 143.6, 141.7, 135.7, 129.8, 129.2, 128.9, 127.6, 123.1, 119.0, 118.8, 43.9, 22.2, 21.1.

IR (film): *ν* (cm^{-1}) 3135, 3094, 3005, 2929, 2873, 1893, 1791, 1516, 1377, 1234, 1118, 1022, 966, 816, 698, 468.

HRMS (ESI-TOF, m/z) calculated for $C_{21}H_{21}O^+$ $[M+H]^+$ 289.1587, found 289.1592.

1-ethyl-4-(4-phenoxybenzyl)benzene (69′)

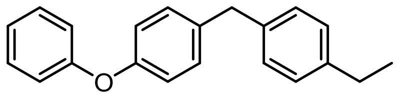

A Schlenk tube (10 mL) was charged with diphenyl ether (**1d**, 51.1 mg, 0.30 mmol), 1-ethyl-4-methylbenzene (**2z**, 108.2 mg, 0.90 mmol), $FeCl_3$ $6H_2O$ (16.2 mg, 0.060 mmol) and $(CHCl_2)_2$ (0.75 mL). The mixture was degassed *via* three freeze-pump-thaw cycles, then filled with dry air. The Schlenk tube was positioned approximately 5 cm away from a 50 W LED lamp (λ_{max} = 410 nm). After being stirred at 25 ℃ for 48 h, the reaction mixture was concentrated to dryness. Purification using silica gel column chromatography (elution with PE:DCM = 5:1) gave the pure product **69′** as a colorless oil (10.4 mg, 0.036 mmol, 12% yield).

^{1}H NMR (500 MHz, $CDCl_3$) δ 7.34 (t, *J* = 7.9 Hz, 2H), 7.20 – 7.12 (m, 6H), 7.10 (t, *J* = 7.4 Hz, 1H), 7.02 (d, *J* = 7.9 Hz, 2H), 6.96 (d, *J* = 8.5 Hz, 2H), 3.96 (s, 2H), 2.65 (q, *J* = 7.6 Hz, 2H), 1.26 (t, *J* = 7.6 Hz, 3H).

^{13}C NMR (126 MHz, $CDCl_3$) δ 157.7, 155.5, 142.2, 138.5, 136.5, 130.2, 129.8, 128.9, 128.1, 123.1, 119.2, 118.7, 40.9, 28.6, 15.8.

IR (film): *ν* (cm^{-1}) 3135, 3094, 2966, 2929, 2731, 1893, 1649, 1516, 1377, 1234, 1118, 1022, 960, 816, 698, 468.

HRMS (ESI-TOF, m/z) calculated for $C_{21}H_{21}O^+$ $[M+H]^+$ 289.1587, found 289.1592.

1-isopropyl-4-(4-(1-(4-methoxyphenyl)ethyl)phenoxy)benzene (70)

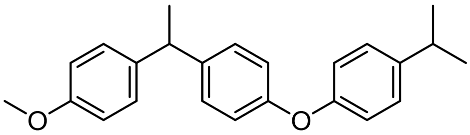

A Schlenk tube (10 mL) was charged with anisole (**1a**, 32.4 mg, 0.30 mmol), 1-ethyl-4-(4-isopropylphenoxy)benzene (**2za**, 216.3 mg, 0.90 mmol), $FeCl_3 \cdot 6H_2O$ (16.2 mg, 0.060 mmol) and DCE (0.75 mL). The mixture was degassed *via* three freeze-pump-thaw cycles, then filled with dry air. The Schlenk tube was positioned approximately 5 cm away from a 50 W LED lamp (λ_{max} = 410 nm). After being stirred at 25 ℃ for 48 h, the reaction mixture was concentrated to dryness. Purification using silica gel column chromatography (elution with PE:DCM = 6:1) gave the pure product **70** as a colorless oil (73.7 mg, 0.213 mmol, 71% yield).

^{1}H NMR (500 MHz, $CDCl_3$) δ 7.20 (t, *J* = 6.0 Hz, 6H), 6.98 – 6.94 (m, 4H), 6.88 (d, *J* = 8.9 Hz, 2H), 4.12 (q, *J* = 7.2 Hz, 1H), 3.81 (s, 3H), 2.97 – 2.88 (m, 1H), 1.64 (d, *J* = 7.2 Hz, 3H), 1.28 (d, *J* = 6.9 Hz, 6H).

^{13}C NMR (126 MHz, $CDCl_3$) δ 158.0, 155.8, 155.4, 143.8, 141.5, 138.8, 128.7, 128.6, 127.6, 118.8, 118.6, 113.9, 55.3, 43.4, 33.6, 24.3, 22.4.

IR (film): *ν* (cm^{-1}) 3101, 3065, 3032, 2933, 2877, 1944, 1801, 1666, 1558, 1494, 1452, 1351, 1323, 1028, 906, 533, 518.

HRMS (ESI-TOF, m/z) calculated for $C_{24}H_{27}O_2^+$ $[M+H]^+$ 347.2006, found 347.2011.

1-cyclohexyl-4-(1-(4-phenoxyphenyl)ethyl)benzene (71)

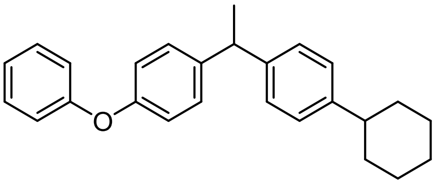

A Schlenk tube (10 mL) was charged with diphenyl ether (**1d**, 51.1 mg, 0.30 mmol), 1-cyclohexyl-4-ethylbenzene (**2zb**, 169.5 mg, 0.90 mmol), $FeCl_3 \cdot 6H_2O$ (16.2 mg, 0.060 mmol) and DCE (0.75 mL). The mixture was degassed *via* three freeze-pump-thaw cycles, then filled with dry air. The Schlenk tube was positioned approximately 5 cm away from a 50 W LED lamp (λ_{max} = 410 nm). After being stirred at 25 ℃ for 48 h, the reaction mixture was concentrated to dryness. Purification using silica gel column chromatography (elution with PE:DCM = 5:1) gave the pure product **71** as a colorless oil (64.1 mg, 0.180 mmol, 60% yield).

^{1}H NMR (500 MHz, $CDCl_3$) δ 7.32 (t, *J* = 7.9 Hz, 2H), 7.19 (d, *J* = 8.4 Hz, 2H), 7.15 (s, 4H), 7.08 (t, *J* = 7.4 Hz, 1H), 7.01 (d, *J* = 7.8 Hz, 2H), 6.94 (d, *J* = 8.5 Hz, 2H), 4.11 (t, *J* = 7.2 Hz,

1H), 2.48 (dd, J = 10.9, 7.9 Hz, 1H), 1.89 – 1.82 (m, 5H), 1.75 (d, J = 12.6 Hz, 1H), 1.63 (d, J = 7.2 Hz, 3H), 1.48 – 1.32 (m, 4H).

^{13}C NMR (126 MHz, $CDCl_3$) δ 157.6, 155.4, 145.9, 143.8, 141.8, 129.8, 129.0, 127.5, 126.9, 123.1, 118.9, 118.8, 44.2, 43.9, 34.6, 27.1, 26.3, 22.3.

IR (film): ν (cm^{-1}) 3080, 3066, 3001, 2777, 2669, 1939, 103, 1494, 1449, 1369, 1266, 1178, 1132, 1003, 986, 863, 765, 689, 526.

HRMS (ESI-TOF, m/z) calculated for $C_{26}H_{29}O^+$ $[M+H]^+$ 357.2213, found 357.2216.

1-cinnamyl-4-methoxybenzene (72)

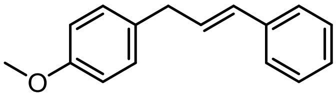

A Schlenk tube (10 mL) was charged with anisole (**1a**, 32.4 mg, 0.30 mmol), β-methylstyrene (**2zc**, 106.4 mg, 0.90 mmol), $FeCl_3$ $6H_2O$ (16.2 mg, 0.060 mmol) and DCE (0.75 mL). The mixture was degassed *via* three freeze-pump-thaw cycles, then filled with dry air. The Schlenk tube was positioned approximately 5 cm away from a 50 W LED lamp (λ_{max} = 410 nm). After being stirred at 25 ℃ for 48 h, the reaction mixture was concentrated to dryness. Purification using silica gel column chromatography (elution with PE:DCM = 5:1) gave the pure product **72** as a colorless oil (44.4 mg, 0.198 mmol, 66% yield).

^{1}H NMR (500 MHz, $CDCl_3$) δ 7.37 (d, J = 7.3 Hz, 2H), 7.30 (t, J = 7.6 Hz, 2H), 7.22 (d, J = 7.3 Hz, 1H), 7.17 (d, J = 8.6 Hz, 2H), 6.89 – 6.85 (m, 2H), 6.45 (d, J = 15.8 Hz, 1H), 6.35 (m, 1H), 3.81 (s, 3H), 3.50 (d, J = 6.6 Hz, 2H).

^{13}C NMR (126 MHz, $CDCl_3$) δ 158.2, 137.7, 132.3, 130.9, 129.8, 129.7, 128.6, 127.2, 126.2, 114.1, 55.4, 38.6.

IR (film): ν (cm^{-1}) 3103, 3083, 3061, 2934, 2915, 1663, 1644, 1578, 1443, 1408, 1379, 1105, 1081, 1069, 981, 964, 913, 806, 767, 613, 499.

HRMS (ESI-TOF, m/z) calculated for $C_{16}H_{17}O^+$ $[M+H]^+$ 225.1274, found 225.1279.

1-cinnamyl-4-phenoxybenzene (73)

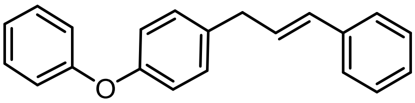

A Schlenk tube (10 mL) was charged with diphenyl ether (**1d**, 51.1 mg, 0.30 mmol), β-methylstyrene (**2zc**, 106.4 mg, 0.90 mmol), $FeCl_3$ $6H_2O$ (16.2 mg, 0.060 mmol) and DCE (0.75 mL). The mixture was degassed *via* three freeze-pump-thaw cycles, then filled with dry air. The Schlenk tube was positioned approximately 5 cm away from a 50 W LED lamp (λ_{max} = 410 nm). After being stirred at 25 ℃ for 48 h, the reaction mixture was concentrated to

dryness. Purification using silica gel column chromatography (elution with PE:DCM = 5:1) gave the pure product **72** as a colorless oil (58.4 mg, 0.204 mmol, 68% yield).

^{1}H NMR (500 MHz, $CDCl_3$) δ 7.36 (d, *J* = 7.4 Hz, 2H), 7.33 – 7.27 (m, 4H), 7.20 (m, 3H), 7.07 (t, *J* = 7.4 Hz, 1H), 7.02 – 6.92 (m, 4H), 6.46 (d, *J* = 15.8 Hz, 1H), 6.35 (m, 1H), 3.53 (d, *J* = 6.7 Hz, 2H).

^{13}C NMR (126 MHz, $CDCl_3$) δ 157.7, 155.6, 137.6, 135.3, 131.2, 130.0, 129.8, 129.4, 128.7, 127.3, 126.3, 123.1, 119.3, 118.7, 38.8.

IR (film): *v* (cm^{-1}) 3093, 3079, 3036, 2963, 2916, 1654, 1562, 1424, 1401, 1339, 1133, 1090, 1056, 963, 815, 735, 656, 513.

HRMS (ESI-TOF, m/z) calculated for $C_{21}H_{19}O^+$ $[M+H]^+$ 287.1430, found 287.1425.

(*E*)-1-(2-methyl-3-phenylallyl)-4-phenoxybenzene (74)

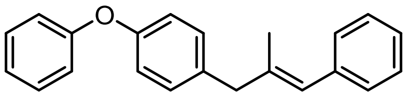

A Schlenk tube (10 mL) was charged with diphenyl ether (**1d**, 51.1 mg, 0.30 mmol), 2-methyl-1-phenylpropene (**2zd**, 119.0 mg, 0.90 mmol), $FeCl_3$ $6H_2O$ (16.2 mg, 0.060 mmol) and DCE (0.75 mL). The mixture was degassed *via* three freeze-pump-thaw cycles, then filled with dry air. The Schlenk tube was positioned approximately 5 cm away from a 50 W LED lamp (λ_{max} = 410 nm). After being stirred at 25 ℃ for 48 h, the reaction mixture was concentrated to dryness. Purification using silica gel column chromatography (elution with PE:DCM = 5:1) gave the pure product **74** as a white solid (52.2 mg, 0.174 mmol, 58% yield).

^{1}H NMR (500 MHz, $CDCl_3$) δ 7.32 (m, 5H), 7.28 (d, *J* = 2.8 Hz, 2H), 7.22 (d, *J* = 8.5 Hz, 2H), 7.10 (d, *J* = 7.4 Hz, 1H), 7.01 (d, *J* = 8.0 Hz, 2H), 6.97 (d, *J* = 8.4 Hz, 2H), 6.38 (s, 1H), 3.46 (s, 2H), 1.82 (s, 3H).

^{13}C NMR (126 MHz, $CDCl_3$) δ 157.7, 155.7, 138.4, 138.3, 134.9, 130.4, 129.8, 129.0, 128.2, 126.9, 126.3, 123.1, 119.1, 118.8, 46.5, 17.8.

IR (film): *v* (cm^{-1}) 3016, 2978, 2881, 2750, 1694, 1612, 1566, 1477, 1259, 1142, 1067, 1053, 992, 926, 832, 756, 642, 557.

HRMS (ESI-TOF, m/z) calculated for $C_{22}H_{21}O^+$ $[M+H]^+$ 301.1587, found 301.1588.

1-(4-phenoxyphenyl)adamantane (75)

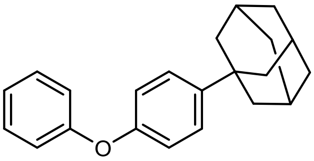

A Schlenk tube (10 mL) was charged with diphenyl ether (**1d**, 51.1 mg, 0.30 mmol), adamantane (**2ze**, 408.7 mg, 3.0 mmol), $FeCl_3$ $6H_2O$ (16.2 mg, 0.060 mmol) and DCE (0.75 mL). The mixture was degassed *via* three freeze-pump-thaw cycles, then filled with dry air. The Schlenk tube was positioned approximately 5 cm away from a 40 W LED lamp (λ_{max} = 370 nm). After being stirred at 80 ℃ for 60 h, the reaction mixture was concentrated to dryness. Purification using silica gel column chromatography (elution with PE) gave the pure product **75** as a white solid (44.7 mg, 0.147 mmol, 49% yield).

^{1}H NMR (500 MHz, $CDCl_3$) δ 7.35 – 7.30 (m, 4H), 7.08 (t, *J* = 7.4 Hz, 1H), 7.01 (dd, *J* = 8.6, 0.9 Hz, 2H), 6.98 – 6.94 (m, 2H), 2.10 (s, 3H), 1.91 (d, *J* = 2.5 Hz, 6H), 1.77 (q, *J* = 12.1 Hz, 6H).

^{13}C NMR (126 MHz, $CDCl_3$) δ 157.7, 154.9, 146.6, 129.8, 126.2, 123.0, 118.8, 118.6, 43.5, 36.9, 36.0, 29.1.

IR (film): *v* (cm^{-1}) 3939, 3068, 3040, 2933, 2901, 2822, 2552, 1954, 1456, 1363, 1333, 1289, 1102, 988, 899, 792, 869, 693.

HRMS (ESI-TOF, m/z) calculated for $C_{22}H_{25}O^+$ $[M+H]^+$ 305.1900, found 305.1911.

1-methyl-3-(4-phenoxyphenyl)adamantane (76)

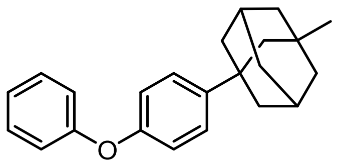

A Schlenk tube (10 mL) was charged with diphenyl ether (**1d**, 51.1 mg, 0.30 mmol), 1-methyladamantane (**2zf**, 450.8 mg, 3.0 mmol), $FeCl_3$ $6H_2O$ (16.2 mg, 0.060 mmol) and DCE (0.75 mL). The mixture was degassed *via* three freeze-pump-thaw cycles, then filled with dry air. The Schlenk tube was positioned approximately 5 cm away from a 40 W LED lamp (λ_{max} = 370 nm). After being stirred at 80 ℃ for 60 h, the reaction mixture was concentrated to dryness. Purification using silica gel column chromatography (elution with PE) gave the pure product **76** as a colorless oil (38.2 mg, 0.120 mmol, 40% yield).

^{1}H NMR (500 MHz, $CDCl_3$) δ 7.34 – 7.30 (m, 4H), 7.08 (t, *J* = 7.4 Hz, 1H), 7.01 (d, *J* = 8.2 Hz, 2H), 6.97 (d, *J* = 8.7 Hz, 2H), 2.16 (s, 2H), 1.83 (dd, *J* = 29.5, 11.8 Hz, 5H), 1.70 – 1.66 (m, 2H), 1.50 (q, *J* = 12.2 Hz, 5H), 0.88 (s, 3H).

^{13}C NMR (126 MHz, $CDCl_3$) δ 157.8, 154.9, 146.3, 129.8, 126.3, 123.0, 118.8, 118.7, 50.6, 43.9, 42.8, 36.9, 36.1, 31.3, 31.0, 29.7.

IR (film): *v* (cm^{-1}) 3931, 3055, 2921, 2895, 2814, 2498, 1931, 1433, 1343, 1290, 1110, 976, 902, 869, 703.

HRMS (ESI-TOF, m/z) calculated for $C_{23}H_{27}O^+$ $[M+H]^+$ 319.2056, found 319.2060.

3-cyclopentyl-1*H*-indole (77)

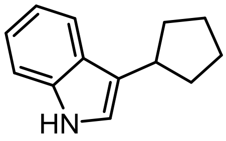

A Schlenk tube (10 mL) was charged with indole (**1ze**, 35.1 mg, 0.30 mmol), cyclopentane (**2zg**, 0.75 mL) and $FeBr_3$ (32.5 mg, 0.11 mmol). The mixture was degassed *via* three freeze-pump-thaw cycles, then filled with dry air. The Schlenk tube was positioned approximately 5 cm away from a 40 W LED lamp (λ_{max} = 370 nm). After being stirred at 25 ℃ for 120 h, the reaction mixture was concentrated to dryness. Purification using silica gel column chromatography (elution with PE:DCM = 2:1) gave the pure product **77** as a white solid (23.9 mg, 0.129 mmol, 43% yield).

^{1}H NMR (500 MHz, $CDCl_3$) δ 7.78 (d, *J* = 7.9 Hz, 1H), 7.72 (s, 1H), 7.37 (d, *J* = 8.1 Hz, 1H), 7.30 (t, *J* = 7.5 Hz, 1H), 7.23 (t, *J* = 7.4 Hz, 1H), 3.42 – 3.35 (m, 1H), 2.30 – 2.24 (m, 2H), 1.96 – 1.89 (m, 2H), 1.87 – 1.79 (m, 4H).

^{13}C NMR (126 MHz, $CDCl_3$) δ 136.7, 127.4, 121. 9, 121.3, 119.8, 119.7, 119.1, 111.2, 37.1, 33.3, 25.4.

IR (film): *v* (cm^{-1}) 3930, 3068, 3041, 2844, 2539, 1950, 1457, 1363, 1111, 983, 893, 788, 867, 663.

HRMS (ESI-TOF, m/z) calculated for $C_{13}H_{16}N^+$ $[M+H]^+$ 186.1277, found 186.1271.

3-cyclohexyl-1*H*-indole (78)

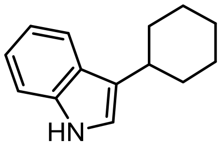

A Schlenk tube (10 mL) was charged with indole (**1ze**, 35.1 mg, 0.30 mmol), cyclohexane (**2zh**, 0.75 mL) and $FeBr_3$ (32.5 mg, 0.11 mmol). The mixture was degassed *via* three freeze-pump-thaw cycles, then filled with dry air. The Schlenk tube was positioned approximately 5 cm away from a 40 W LED lamp (λ_{max} = 370 nm). After being stirred at 25 ℃ for 120 h, the reaction mixture was concentrated to dryness. Purification using silica gel column chromatography (elution with PE:DCM = 2:1) gave the pure product **78** as a white solid (24.5 mg, 0.123 mmol, 41% yield).

^{1}H NMR (500 MHz, $CDCl_3$) δ 7.85 (s, 1H), 7.69 (d, *J* = 7.9 Hz, 1H), 7.36 (d, *J* = 8.1 Hz, 1H), 7.20 (t, *J* = 7.5 Hz, 1H), 7.13 (t, *J* = 7.5 Hz, 1H), 6.95 (d, *J* = 2.2 Hz, 1H), 2.91 – 2.81 (m, 1H), 2.15 (t, *J* = 9.6 Hz, 2H), 1.91 – 1.85 (m, 2H), 1.81 (d, *J* = 12.7 Hz, 1H), 1.50 (t, *J* = 9.9 Hz, 4H), 1.36 – 1.29 (m, 1H).

^{13}**C NMR** (126 MHz, $CDCl_3$) δ 136.5, 126.9, 123.4, 121.9, 119.5, 119.4, 119.1, 111.2, 35.6, 34.2, 27.1, 26.7.

IR (film): *ν* (cm^{-1}) 3939, 3068, 3040, 2933, 2901, 2822, 2552, 1954, 1456, 1363, 1333, 1289, 1102, 988, 899, 792, 869, 693.

HRMS (ESI-TOF, m/z) calculated for $C_{14}H_{18}N^+$ $[M+H]^+$ 200.1434, found 200.1440.

3-cycloheptyl-1*H*-indole (79)

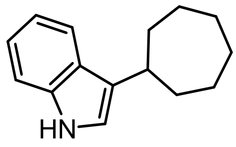

A Schlenk tube (10 mL) was charged with indole (**1ze**, 35.1 mg, 0.30 mmol), cycloheptane (**2zi**, 0.75 mL) and $FeBr_3$ (32.5 mg, 0.11 mmol). The mixture was degassed *via* three freeze-pump-thaw cycles, then filled with dry air. The Schlenk tube was positioned approximately 5 cm away from a 40 W LED lamp (λ_{max} = 370 nm). After being stirred at 25 ℃ for 120 h, the reaction mixture was concentrated to dryness. Purification using silica gel column chromatography (elution with PE:DCM = 2:1) gave the pure product **79** as a white solid (26.2 mg, 0.123 mmol, 41% yield).

^{1}**H NMR** (500 MHz, $CDCl_3$) δ 7.84 (s, 1H), 7.64 (d, *J* = 7.9 Hz, 1H), 7.35 (d, *J* = 8.1 Hz, 1H), 7.19 (t, *J* = 7.5 Hz, 1H), 7.11 (t, *J* = 7.5 Hz, 1H), 6.96 (d, *J* = 2.2 Hz, 1H), 3.09 – 3.03 (m, 1H), 2.12 (ddd, *J* = 8.9, 6.6, 3.1 Hz, 2H), 1.84 – 1.78 (m, 2H), 1.78 – 1.70 (m, 4H), 1.66 – 1.59 (m, 4H).

^{13}**C NMR** (126 MHz, $CDCl_3$) δ 136.6, 126.8, 124.6, 121.9, 119.5, 119.44, 119.1, 111.2, 37.4, 35.8, 28.5, 27.1.

IR (film): ν (cm^{-1}) 3944, 3077, 3053, 3046, 2941, 2801, 2664, 2553, 1936, 1476, 1338, 1323, 1269, 1024, 999, 876, 791, 866, 644, 593.

HRMS (ESI-TOF, m/z) calculated for $C_{15}H_{20}N^+$ $[M+H]^+$ 214.1590, found 214.1588.

3-(4,4-dimethylcyclohexyl)-1*H*-indole (80)

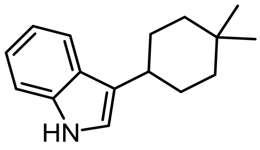

A Schlenk tube (10 mL) was charged with indole (**1ze**, 35.1 mg, 0.30 mmol), 1,1-dimethylcyclohexane (**2zj**, 0.75 mL) and $FeBr_3$ (32.5 mg, 0.11 mmol). The mixture was degassed *via* three freeze-pump-thaw cycles, then filled with dry air. The Schlenk tube was positioned approximately 5 cm away from a 40 W LED lamp (λ_{max} = 370 nm). After being

stirred at 25 ℃ for 120 h, the reaction mixture was concentrated to dryness. Purification using silica gel column chromatography (elution with PE:DCM = 2:1) gave the pure product **80** as a white solid (29.3 mg, 0.129 mmol, 43% yield).

^{1}H NMR (500 MHz, $CDCl_3$) δ 7.81 (d, *J* = 7.9 Hz, 1H), 7.78 (s, 1H), 7.41 (d, *J* = 8.0 Hz, 1H), 7.32 (t, *J* = 7.7 Hz, 1H), 7.25 (t, *J* = 7.4 Hz, 1H), 7.00 (d, *J* = 2.1 Hz, 1H), 2.89 (tt, *J* = 12.0, 3.5 Hz, 1H), 2.10 – 2.04 (m, 2H), 1.87 – 1.77 (m, 2H), 1.66 (d, *J* = 12.8 Hz, 2H), 1.60 – 1.52 (m, 2H), 1.15 (s, 3H), 1.14 (s, 3H).

^{13}C NMR (126 MHz, $CDCl_3$) δ 136.5, 127.0, 122.8, 121.9, 119.6, 119.5, 119.1, 111.3, 39.9, 35.6, 33.3, 30.2, 29.8, 24.6.

IR (film): ν (cm^{-1}) 3943, 3066, 3061, 2912, 2844, 2738, 1976, 1456, 1444, 1384, 1365, 1343, 1217, 1175, 1062, 974, 954, 939, 857, 843.

HRMS (ESI-TOF, m/z) calculated for $C_{16}H_{22}N^+$ $[M+H]^+$ 228.1747, found 228.1751.

2-((4-(1-([1,1'-biphenyl]-4-yl)ethyl)phenoxy)methyl)tetrahydrofuran (93)

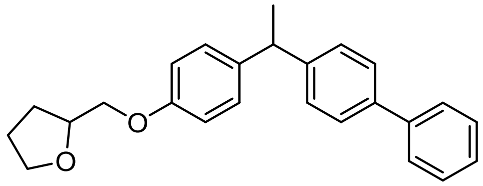

A Schlenk tube (10 mL) was charged with 2-(phenoxymethyl)tetrahydrofuran (**1zo**, 53.4 mg, 0.30 mmol), 4-ethylbiphenyl (**2a**, 164.0 mg, 0.90 mmol), $FeCl_3$ $6H_2O$ (16.2 mg, 0.060 mmol) and $(CHCl_2)_2$ (0.75 mL). The mixture was degassed *via* three freeze-pump-thaw cycles, then filled with dry air. The Schlenk tube was positioned approximately 5 cm away from a 50 W LED lamp (λ_{max} = 410 nm). After being stirred at 25 ℃ for 48 h, the reaction mixture was concentrated to dryness. Purification using silica gel column chromatography (elution with PE:DCM = 2:1) gave the pure product **93** as a white solid (64.5 mg, 0.180 mmol, 60% yield).

^{1}H NMR (500 MHz, $CDCl_3$) δ 7.58 – 7.55 (m, 2H), 7.53 – 7.49 (m, 2H), 7.42 (dd, *J* = 10.5, 4.8 Hz, 2H), 7.34 – 7.30 (m, 1H), 7.27 (d, *J* = 8.1 Hz, 2H), 7.16 (d, *J* = 8.6 Hz, 2H), 6.89 – 6.86 (m, 2H), 4.29 – 4.24 (m, 1H), 4.15 (q, *J* = 7.2 Hz, 1H), 3.98 – 3.95 (m, 1H), 3.94 – 3.90 (m, 2H), 3.83 (dd, *J* = 14.4, 7.6 Hz, 1H), 2.09 – 2.03 (m, 1H), 1.95 (dtd, *J* = 10.9, 8.3, 6.8 Hz, 2H), 1.79 – 1.73 (m, 1H), 1.65 (d, *J* = 7.2 Hz, 3H).

^{13}C NMR (126 MHz, $CDCl_3$) δ 157.2, 145.9, 141.0, 138.9, 138.6, 128.7, 128.5, 127.9, 127.1, 127.0, 114.5, 77.1, 70.5, 68.6, 43.7, 28.3, 25.7, 22.1.

IR (film): *ν* (cm^{-1}) 3600, 344, 2977, 2874, 1460, 1445, 1356, 1434, 1285, 1203, 1197, 1098, 1042, 975, 932, 901, 867, 648, 489.

HRMS (ESI-TOF, m/z) calculated for $C_{25}H_{27}O_2^+$ $[M+H]^+$ 359.2006, found 359.2014.

1-((4-(1-([1,1'-biphenyl]-4-yl)ethyl)phenoxy)methyl)adamantane (94)

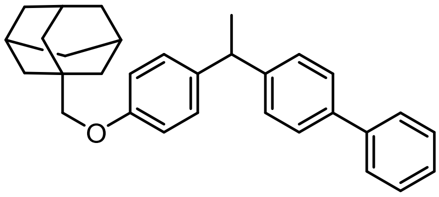

A Schlenk tube (10 mL) was charged with 1-(phenoxymethyl)adamantane (**1zp**, 72.7 mg, 0.30 mmol), 4-ethylbiphenyl (**2a**, 164.0 mg, 0.90 mmol), $FeCl_3$ $6H_2O$ (16.2 mg, 0.060 mmol) and $(CHCl_2)_2$ (0.75 mL). The mixture was degassed *via* three freeze-pump-thaw cycles, then filled with dry air. The Schlenk tube was positioned approximately 5 cm away from a 50 W LED lamp (λ_{max} = 410 nm). After being stirred at 25 ℃ for 48 h, the reaction mixture was concentrated to dryness. Purification using silica gel column chromatography (elution with PE:DCM = 2:1) gave the pure product **94** as a white solid (63.3 mg, 0.150 mmol, 50% yield).

^{1}H NMR (500 MHz, $CDCl_3$) δ 7.62 – 7.59 (m, 1H), 7.55 (dd, *J* = 6.3, 2.0 Hz, 1H), 7.44 (d, *J* = 1.6 Hz, 1H), 7.34 (d, *J* = 14.6 Hz, 1H), 7.21 (s, 1H), 6.90 (s, 1H), 4.18 (d, *J* = 7.2 Hz, 1H), 3.51 (s, 1H), 2.05 (dd, *J* = 6.2, 3.2 Hz, 1H), 1.80 – 1.73 (m, 1H), 1.70 – 1.68 (m, 1H).

^{13}C NMR (126 MHz, $CDCl_3$) δ 158.2, 146.2, 141.2, 138.9, 138.1, 128.8, 128.6, 128.0, 127.2, 127.1, 114.5, 78.4, 43.8, 39.7, 37.3, 33.9, 28.4, 22.2.

IR (film): *ν* (cm^{-1}) 3229, 2913, 2845, 2674, 2655, 1471, 1434, 1367, 1346, 1316, 1221, 1172, 1104, 1042, 998, 975, 939, 912, 805, 699, 600, 488.

HRMS (ESI-TOF, m/z) calculated for $C_{31}H_{35}O^+$ $[M+H]^+$ 423.2682, found 423.2683.

6-(1-([1,1'-biphenyl]-4-yl)ethyl)benzo[*d*][1,3]dioxol-5-ol (95)

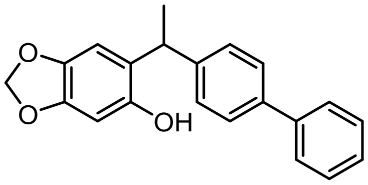

A Schlenk tube (10 mL) was charged with sesamol (**1zq**, 41.4 mg, 0.30 mmol), 4-ethylbiphenyl (**2a**, 164.0 mg, 0.90 mmol), $FeCl_3$ $6H_2O$ (16.2 mg, 0.060 mmol) and $CHCl_3$ (0.75 mL). The mixture was degassed *via* three freeze-pump-thaw cycles, then filled with dry air. The Schlenk tube was positioned approximately 5 cm away from a 50 W LED lamp (λ_{max} = 410 nm). After being stirred at 25 ℃ for 48 h, the reaction mixture was concentrated to dryness. Purification using silica gel column chromatography (elution with PE:DCM = 2:1) gave the pure product **95** as a white solid (59.2 mg, 0.186 mmol, 62% yield).

^{1}H NMR (500 MHz, $CDCl_3$) δ 7.59 – 7.56 (m, 2H), 7.54 (d, *J* = 8.2 Hz, 2H), 7.43 (t, *J* = 7.7 Hz, 2H), 7.35 – 7.32 (m, 3H), 6.78 (s, 1H), 6.40 (s, 1H), 5.90 (d, *J* = 3.5 Hz, 2H), 4.36 (q, *J* = 7.2 Hz, 1H), 1.62 (d, *J* = 7.2 Hz, 3H).

^{13}**C NMR** (126 MHz, $CDCl_3$) δ 147.7, 146.3, 144.6, 141.7, 140.9, 139.4, 128.8, 127.8, 127.5, 127.2, 127.1, 124.2, 107.4, 101.1, 98.9, 38.1, 21.3.

IR (film): *v* (cm^{-1}) 3474, 2964, 2927, 1503, 1485, 1436, 1169, 1037, 852, 766, 697, 590.

HRMS (ESI-TOF, m/z) calculated for $C_{21}H_{19}O_3^+$ [M+H]$^+$ 319.1329, found 319.1321.

4-(1-(4-(((1*R*,2*S*,5*R*)-2-isopropyl-5-methylcyclohexyl)oxy)phenyl)ethyl)-1,1'-biphenyl (96)

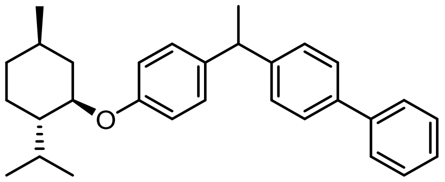

A Schlenk tube (10 mL) was charged with (((1*R*,2*S*,5*R*)-2-isopropyl-5-methylcyclohexyl)oxy)benzene (**1zr**, 69.7 mg, 0.30 mmol), 4-ethylbiphenyl (**2a**, 164.0 mg, 0.90 mmol), $FeCl_3 \cdot 6H_2O$ (16.2 mg, 0.060 mmol) and $(CHCl_2)_2$ (0.75 mL). The mixture was degassed *via* three freeze-pump-thaw cycles, then filled with dry air. The Schlenk tube was positioned approximately 5 cm away from a 50 W LED lamp (λ_{max} = 410 nm). After being stirred at 25 ℃ for 48 h, the reaction mixture was concentrated to dryness. Purification using silica gel column chromatography (elution with PE:DCM = 5:1) gave the pure product **96** as a colorless oil (90.2 mg, 0.219 mmol, 73% yield).

^{1}**H NMR** (500 MHz, $CDCl_3$) δ 7.59 (d, *J* = 7.3 Hz, 2H), 7.53 (d, *J* = 8.2 Hz, 2H), 7.43 (t, *J* = 7.7 Hz, 2H), 7.35 – 7.30 (m, 3H), 7.16 (d, *J* = 8.5 Hz, 2H), 6.86 (d, *J* = 8.6 Hz, 2H), 4.15 (q, *J* = 7.2 Hz, 1H), 4.01 (td, *J* = 10.5, 4.1 Hz, 1H), 2.23 (tt, *J* = 9.1, 3.5 Hz, 1H), 2.17 (d, *J* = 12.5 Hz, 1H), 1.75 – 1.70 (m, 2H), 1.67 (d, *J* = 7.2 Hz, 3H), 1.56 – 1.46 (m, 2H), 1.14 – 0.99 (m, 3H), 0.93 (dd, *J* = 9.5, 6.9 Hz, 6H), 0.79 (d, *J* = 6.9 Hz, 3H).

^{13}**C NMR** (126 MHz, $CDCl_3$) δ 156.8, 146.1, 141.2, 139.0, 138.3, 128.8, 128.67, 128.1, 127.21, 127.2, 115.9, 77.6, 48.2, 43.8, 40.5, 34.7, 31.6, 26.2, 23.9, 22.3, 22.2, 20.9, 16.7.

IR (film): *v* (cm^{-1}) 3344, 332, 2956, 2870, 2772, 1385, 1345, 1313, 1227, 1141, 1104, 1079, 1026, 994, 967, 845, 555, 541, 493.

HRMS (ESI-TOF, m/z) calculated for $C_{30}H_{37}O^+$ [M+H]$^+$ 413.2839, found 413.2848.

(1*S*,2*R*,4*S*)-2-(4-(1-([1,1'-biphenyl]-4-yl)ethyl)phenoxy)-1,7,7-trimethylbicyclo[2.2.1]heptane (97)

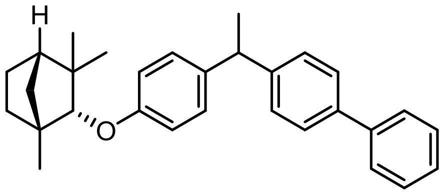

A Schlenk tube (10 mL) was charged with (1*R*,2*R*,4*S*)-2-(4-(1-([1,1'-biphenyl]-4-yl)ethyl)phenoxy)-1,3,3-trimethylbicyclo[2.2.1]heptane (**1zs**, 68.8 mg, 0.30 mmol), 4-ethylbiphenyl (**2a**, 164.0 mg, 0.90 mmol), $FeCl_3$ $6H_2O$ (16.2 mg, 0.060 mmol) and $(CHCl_2)_2$ (0.75 mL). The mixture was degassed *via* three freeze-pump-thaw cycles, then filled with dry air. The Schlenk tube was positioned approximately 5 cm away from a 50 W LED lamp (λ_{max} = 410 nm). After being stirred at 25 ℃ for 48 h, the reaction mixture was concentrated to dryness. Purification using silica gel column chromatography (elution with PE:DCM = 5:1) gave the pure product **97** as a colorless oil (68.9 mg, 0.168 mmol, 56% yield).

^{1}H NMR (500 MHz, $CDCl_3$) δ 7.59 (dd, *J* = 8.2, 1.4 Hz, 2H), 7.53 (s, 2H), 7.44 (dd, *J* = 9.7, 4.1 Hz, 2H), 7.33 (dd, *J* = 12.8, 8.1 Hz, 3H), 7.15 (dd, *J* = 6.5, 2.2 Hz, 2H), 6.86 (dd, *J* = 6.6, 2.1 Hz, 2H), 4.15 (dd, *J* = 14.4, 7.2 Hz, 1H), 3.85 – 3.83 (m, 1H), 2.07 – 2.02 (m, 1H), 1.79 (dd, *J* = 9.4, 6.2 Hz, 1H), 1.75 – 1.73 (m, 1H), 1.67 (d, *J* = 7.2 Hz, 3H), 1.59 (s, 1H), 1.48 (s, 1H), 1.22 – 1.20 (m, 1H), 1.18 (d, *J* = 3.9 Hz, 3H), 1.11 (s, 3H), 1.10 – 1.06 (m, 1H), 0.88 (s, 3H).

^{13}C NMR (126 MHz, $CDCl_3$) δ 158.7, 146.1, 141.1, 138.9, 137.8, 128.7, 128.4, 128.0, 127.1, 127.0, 127.0, 115.7, 90.2, 49.6, 49.1, 43.7, 41.4, 40.0, 30.5, 26.4, 25.9, 22.1, 20.5, 20.0.

IR (film): *ν* (cm^{-1}) 3426, 3081, 3000, 2832, 1611, 1589, 1407, 1342, 1310, 1166, 1017, 988, 800, 761, 724, 494.

HRMS (ESI-TOF, m/z) calculated for $C_{30}H_{35}O^+$ $[M+H]^+$ 411.2682, found 411.2677.

(1*S*,2*R*,4*S*)-2-(4-(1-([1,1'-biphenyl]-4-yl)ethyl)phenoxy)-1,7,7-trimethylbicyclo[2.2.1]heptane (98)

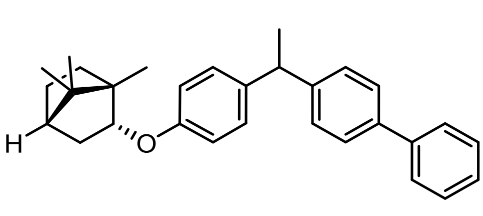

A Schlenk tube (10 mL) was charged with (1*S*,2*R*,4*S*)-1,7,7-trimethyl-2-phenoxybicyclo[2.2.1]heptane (**1zt**, 69.1 mg, 0.30 mmol), 4-ethylbiphenyl (**2a**, 164.0 mg, 0.90 mmol), $FeCl_3$ $6H_2O$ (16.2 mg, 0.060 mmol) and

$(CHCl_2)_2$ (0.75 mL). The mixture was degassed *via* three freeze-pump-thaw cycles, then filled with dry air. The Schlenk tube was positioned approximately 5 cm away from a 50 W LED lamp (λ_{max} = 410 nm). After being stirred at 25 ℃ for 48 h, the reaction mixture was concentrated to dryness. Purification using silica gel column chromatography (elution with PE:DCM = 5:1) gave the pure product **98** as a colorless oil (81.2 mg, 0.198 mmol, 66% yield).

^{1}H NMR (500 MHz, $CDCl_3$) δ 7.61 (d, *J* = 7.5 Hz, 2H), 7.55 (d, *J* = 7.9 Hz, 2H), 7.45 (t, *J* = 7.7 Hz, 2H), 7.37 – 7.32 (m, 3H), 7.19 (d, *J* = 8.5 Hz, 2H), 6.83 (d, *J* = 8.6 Hz, 2H), 4.33 (d, *J* = 9.1 Hz, 1H), 4.18 (q, *J* = 7.2 Hz, 1H), 2.41 – 2.36 (m, 1H), 2.30 – 2.26 (m, 1H), 1.81 – 1.76 (m, 2H), 1.69 (d, *J* = 7.2 Hz, 3H), 1.39 – 1.34 (m, 1H), 1.31 – 1.27 (m, 1H), 1.16 (dd, *J* = 13.3, 1.6 Hz, 1H), 0.97 (s, 3H), 0.95 (d, *J* = 3.4 Hz, 6H).

^{13}C NMR (126 MHz, $CDCl_3$) δ 157.6, 146.2, 141.2, 139.0, 138.0, 128.8, 128.6, 128.1, 127.2, 127.1, 115.4, 82.9, 49.6, 47.7, 45.3, 43.8, 37.0, 28.1, 26.9, 22.2, 19.9, 19.1, 13.9.

IR (film): *v* (cm^{-1}) 3426, 3081, 3000, 2832, 1611, 1589, 1407, 1342, 1310, 1166, 1017, 988, 800, 761, 724, 494.

HRMS (ESI-TOF, m/z) calculated for $C_{30}H_{35}O^+$ $[M+H]^+$ 411.2682, found 411.2680.

(3*R*,5*R*,8*S*,9*R*,10*R*,13*R*,14*R*)-3-(4-(1-([1,1'-biphenyl]-4-yl)ethyl)phenoxy)-10,13-dimethyl hexadecahydro-17H-cyclopenta[a]phenanthren-17-one (99)

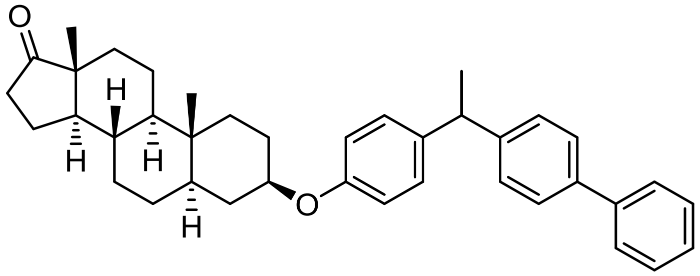

A Schlenk tube (10 mL) was charged with (3*R*,5*R*,8*S*,9*R*,10*R*,13*R*,14*R*)-10,13-dimethyl-3-phenoxyhexadecahydro-17*H*-cyclopenta[*a*]phenanthren-17-one (**1zu**, 109.9 mg, 0.30 mmol), 4-ethylbiphenyl (**2a**, 164.0 mg, 0.90 mmol), $FeCl_3$ $6H_2O$ (16.2 mg, 0.060 mmol) and $(CHCl_2)_2$ (0.75 mL). The mixture was degassed *via* three freeze-pump-thaw cycles, then filled with dry air. The Schlenk tube was positioned approximately 5 cm away from a 50 W LED lamp (λ_{max} = 410 nm). After being stirred at 25 ℃ for 48 h, the reaction mixture was concentrated to dryness. Purification using silica gel column chromatography (elution with PE:DCM = 2:1) gave the pure product **99** as a colorless oil (57.4 mg, 0.105 mmol, 35% yield).

^{1}H NMR (500 MHz, $CDCl_3$) δ 7.58 – 7.56 (m, 2H), 7.52 (d, *J* = 8.3 Hz, 2H), 7.42 (t, *J* = 7.7 Hz, 2H), 7.34 – 7.31 (m, 1H), 7.30 – 7.28 (m, 2H), 7.17 – 7.14 (m, 2H), 6.84 (d, *J* = 8.6 Hz, 2H), 4.52 – 4.47 (m, 1H), 4.14 (d, *J* = 7.2 Hz, 1H), 2.44 (d, *J* = 10.4 Hz, 1H), 2.12 – 2.03 (m, 2H), 1.97 – 1.91 (m, 2H), 1.80 (ddd, *J* = 13.8, 7.1, 3.3 Hz, 3H), 1.65 (d, *J* = 7.2 Hz, 6H), 1.61

– 1.55 (m, 2H), 1.52 – 1.46 (m, 3H), 1.40 (d, J = 9.6 Hz, 1H), 1.27 (d, J = 5.2 Hz, 3H), 1.03 (d, J = 18.6 Hz, 2H), 0.87 (s, 3H), 0.85 (s, 3H).

^{13}C NMR (126 MHz, $CDCl_3$) δ 156.1, 146.0, 141.0, 138.8, 138.1, 128.7, 128.5, 128.0, 127.1, 127.0, 127.0, 115.9, 71.90 54.3, 51.5, 47.9, 43.7, 39.6, 36.0, 35.9, 35.1, 32.8, 32.7, 31.6, 30.8, 28.2, 25.7, 22.1, 21.8, 20.1, 13.9, 11.5.

IR (film): ν (cm^{-1}) 3470, 2976, 2950, 2916, 2891, 2837, 1728, 1469, 1451, 1387, 1373, 1305, 1293, 1132, 1099, 1060, 1024, 1010, 901, 888, 614, 589.

HRMS (ESI-TOF, m/z) calculated for $C_{39}H_{47}O_2^+$ $[M+H]^+$ 547.3571, found 547.3579.

2,2-difluoro-5-(4-(1-(4-methoxyphenyl)ethyl)phenoxy)benzo[*d*][1,3]dioxole (100)

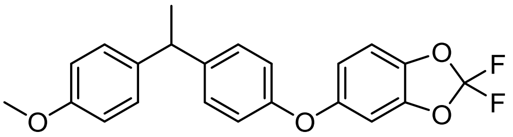

A Schlenk tube (10 mL) was charged with anisole (**1a**, 32.4 mg, 0.30 mmol), 5-(4-ethylphenoxy)-2,2-difluorobenzo[*d*][1,3]dioxole (**2zk**, 250.4 mg, 0.90 mmol), $FeCl_3$ $6H_2O$ (16.2 mg, 0.060 mmol) and DCE (0.75 mL). The mixture was degassed *via* three freeze-pump-thaw cycles, then filled with dry air. The Schlenk tube was positioned approximately 5 cm away from a 50 W LED lamp (λ_{max} = 410 nm). After being stirred at 25 ℃ for 48 h, the reaction mixture was concentrated to dryness. Purification using silica gel column chromatography (elution with PE:DCM = 5:1) gave the pure product **100** as a colorless oil (80.7 mg, 0.210 mmol, 70% yield).

^{1}H NMR (500 MHz, $CDCl_3$) δ 7.18 (d, J = 8.5 Hz, 2H), 7.14 (d, J = 8.5 Hz, 2H), 6.97 (d, J = 8.7 Hz, 1H), 6.90 (d, J = 8.6 Hz, 2H), 6.78 (d, J = 8.7 Hz, 2H), 6.76 – 6.68 (m, 2H), 4.10 (q, J = 7.2 Hz, 1H), 3.79 (s, 3H), 1.62 (d, J = 7.2 Hz, 3H).

^{13}C NMR (126 MHz, $CDCl_3$) δ 158.1, 155.3, 153.9, 144.4, 142.5, 139.6, 138.5, 129.0 (d, $J_{C\text{-}F}$ = 225.0 Hz), 118.7, 114.0, 113.4, 109.7, 102.0, 55.4, 43. 5, 22.3.

IR (film): ν (cm^{-1}) 3065, 3007, 2954, 2944, 2779, 1750, 1628, 1498, 1484, 1405, 1334, 1304, 1266, 1152, 1093, 1023, 882, 691, 533.

HRMS (ESI-TOF, m/z) calculated for $C_{22}H_{19}F_2O_4^+$ $[M+H]^+$ 385.1246, found 385.1244.

2-((5-bromo-2-methylphenyl)(4-methoxyphenyl)methyl)-5-(4-fluorophenyl)thiophene (101)

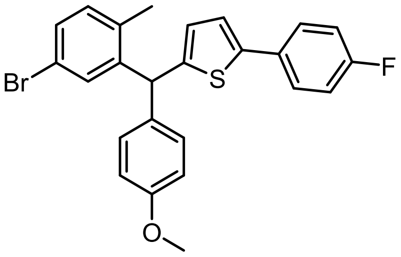

A Schlenk tube (10 mL) was charged with anisole (**1a**, 32.4 mg, 0.30 mmol), 2-(5-bromo-2-methylbenzyl)-5-(4-fluorophenyl)thiophene (**2zl**, 324.0 mg, 0.90 mmol), $FeCl_3$ $6H_2O$ (16.2 mg, 0.060 mmol) and DCE (0.75 mL). The mixture was degassed *via* three freeze-pump-thaw cycles, then filled with dry air. The Schlenk tube was positioned approximately 5 cm away from a 50 W LED lamp (λ_{max} = 410 nm). After being stirred at 25 ℃ for 48 h, the reaction mixture was concentrated to dryness. Purification using silica gel column chromatography (elution with PE:DCM = 2:1) gave the pure product **101** as a colorless oil (81.1 mg, 0.174 mmol, 58% yield).

^{1}H NMR (500 MHz, $CDCl_3$) δ 7.50 (dd, *J* = 8.4, 5.4 Hz, 2H), 7.31 – 7.29 (m, 1H), 7.18 (s, 1H), 7.09 (d, *J* = 8.6 Hz, 2H), 7.08 (d, *J* = 3.7 Hz, 1H), 7.04 (d, *J* = 8.4 Hz, 3H), 6.87 (d, *J* = 8.5 Hz, 2H), 6.57 (d, *J* = 3.5 Hz, 1H), 5.70 (s, 1H), 3.81 (s, 3H), 2.23 (s, 3H).

^{13}C NMR (126 MHz, $CDCl_3$) δ 163.3, 161.4, 158.7, 146.8, 144.6, 142.6, 135.4, 134.3, 131.9 (d, J_{C-F} = 340.0 Hz), 130.8 (d, J_{C-F} = 10.0 Hz), 130.1, 130.0, 127.8, 127.3 (d, J_{C-F} = 30.0 Hz), 122.71, 119.97, 115.9 (d, J_{C-F} = 80.0 Hz), 114.1, 55.4, 48.0, 19.4.

IR (film): *v* (cm^{-1}) 3937, 3828, 3087, 3077, 3055, 2920, 1942, 1769, 1687, 1579, 1526, 1555, 1251, 1034, 1000, 988, 903, 871, 831, 714, 519, 457, 447.

HRMS (ESI-TOF, m/z) calculated for $C_{25}H_{20}BrFNaOS^+$ $[M+Na]^+$ 489.0294, found 489.0289.

2,4-dichloro-1-(4-chloro-2-(3-(4-methoxyphenyl)-3-phenylpropoxy)phenoxy)benzene (102)

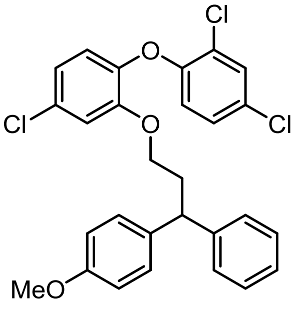

A Schlenk tube (10 mL) was charged with anisole (**1a**, 32.4 mg, 0.30 mmol),

2,4-dichloro-1-(4-chloro-2-(3-phenylpropoxy)phenoxy)benzene (**2zm**, 366.3 mg, 0.90 mmol), $FeCl_3$ $6H_2O$ (16.2 mg, 0.060 mmol) and DCE (0.75 mL). The mixture was degassed *via* three freeze-pump-thaw cycles, then filled with dry air. The Schlenk tube was positioned approximately 5 cm away from a 50 W LED lamp (λ_{max} = 410 nm). After being stirred at 25 ℃ for 48 h, the reaction mixture was concentrated to dryness. Purification using silica gel column chromatography (elution with PE:DCM = 2:1) gave the pure product **102** as a colorless oil (61.4 mg, 0.120 mmol, 40% yield).

^{1}H NMR (500 MHz, $CDCl_3$) δ 7.44 (d, *J* = 2.5 Hz, 1H), 7.24 (s, 1H), 7.17 (d, *J* = 7.4 Hz, 1H), 7.12 (dd, *J* = 9.5, 6.4 Hz, 4H), 7.02 (dd, *J* = 10.3, 8.7 Hz, 3H), 6.91 (dd, *J* = 8.5, 2.3 Hz, 1H), 6.83 – 6.81 (m, 3H), 6.65 (d, *J* = 8.8 Hz, 1H), 3.86 (d, *J* = 6.3 Hz, 1H), 3.82 (t, *J* = 6.0 Hz, 2H), 3.77 (s, 3H), 2.30 (dd, *J* = 12.7, 6.4 Hz, 2H).

^{13}C NMR (126 MHz, $CDCl_3$) δ 158.2, 153.0, 151.0, 144.4, 142.9, 136.1, 130.9, 130.4, 129.9, 128.9, 128.7, 127.9, 127.7, 126.4, 124.4, 122.7, 121.1, 117.6, 114.7, 114.0, 66.8, 55.4, 45.9, 35.0.

IR (film): *v* (cm^{-1}) 3245, 3001, 2987, 1612, 1573, 1499, 1450, 1249, 1200, 1081, 1035, 996, 869, 677, 547.

HRMS (ESI-TOF, m/z) calculated for $C_{28}H_{23}Cl_3NaO_3^+$ $[M+Na]^+$ 535.0605, found 535.0611.

4-(1,2,3,4-tetrahydronaphthalen-1-yl)phenol (103)

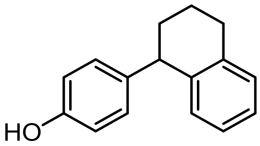

A Schlenk tube (10 mL) was charged phenol (**1p**, 28.2 mg, 0.30 mmol), 1,2,3,4-tetrahydronaphthalene (**2s**, 118.9 mg, 0.90 mmol), $FeCl_3$ $6H_2O$ (16.2 mg, 0.060 mmol) and $CHCl_3$ (0.75 mL). The mixture was degassed *via* three freeze-pump-thaw cycles, then filled with dry air. The Schlenk tube was positioned approximately 5 cm away from a 50 W LED lamp (λ_{max} = 410 nm). After being stirred at 25 ℃ for 48 h, the reaction mixture was concentrated to dryness. Purification using silica gel column chromatography (elution with PE:DCM = 2:1) gave the pure product **103** as a white solid (46.4 mg, 0.207 mmol, 69% yield).

^{1}H NMR (500 MHz, $CDCl_3$) δ 7.14 (d, *J* = 6.1 Hz, 2H), 7.06 (dd, *J* = 9.7, 4.2 Hz, 1H), 6.98 (d, *J* = 8.4 Hz, 2H), 6.87 (d, *J* = 7.8 Hz, 1H), 6.76 (d, *J* = 8.5 Hz, 2H), 4.87 (br, s, 1H), 4.08 (t, *J* = 6.5 Hz, 1H), 2.93 – 2.89 (m, 1H), 2.87 (t, *J* = 5.9 Hz, 1H), 2.17 – 2.13 (m, 1H), 1.91 – 1.85 (m, 2H), 1.80 – 1.76 (m, 1H).

^{13}C NMR (126 MHz, $CDCl_3$) δ 153.8, 140.0, 139.8, 137. 7, 130.2, 130.0, 129.1, 126.0, 125.7, 115.2, 44.9, 33.5, 29.9, 21.1.

IR (film): *v* (cm^{-1}) 3101, 3075, 3044, 2853, 2838, 1579, 1557, 1431, 1341, 1238, 986, 824,

822, 690, 618.

HRMS (ESI-TOF, m/z) calculated for $C_{16}H_{17}O^+$ $[M+H]^+$ 225.1274, found 225.1268.

ethyl 2-methyl-2-(4-(1,2,3,4-tetrahydronaphthalen-1-yl)phenoxy)propanoate (105)

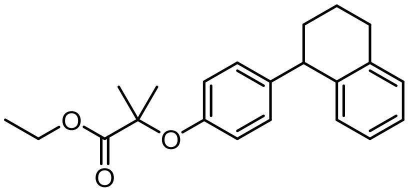

According to the supplementary reference, [7] a solution of 4-(1,2,3,4-tetrahydronaphthalen-1-yl)phenol (**103**, 64.0 mg, 0.28 mmol) in DMF (2.0 mL), $MgSO_4$ (35.0 mg, 0.29 mmol), K_2CO_3 (157.0 mg, 1.12 mmol) and *tert*-butyl α-bromoisobutyrate (**104**, 266 μL, 1.4 mmol) were added and the reaction was stirred at 100 ℃ for 24 h. After that time, the reaction was quenched with water (30 mL) and extracted three times with ethyl acetate (3 × 20 mL). The combined organic layers were dried over $MgSO_4$, the solvent was removed under reduced pressure. Purification using silica gel column chromatography (elution with PE:EtOAc = 5:1) gave the pure product **105** as a colorless oil (92.9 mg, 0.275 mmol, 98% yield).

^{1}H NMR (500 MHz, $CDCl_3$) δ 7.13 – 7.09 (m, 2H), 7.04 – 7.00 (m, 1H), 6.96 – 6.92 (m, 2H), 6.83 (d, *J* = 7.7 Hz, 1H), 6.77 – 6.73 (m, 2H), 4.23 (q, *J* = 7.1 Hz, 2H), 4.05 (t, *J* = 6.7 Hz, 1H), 2.93 – 2.78 (m, 2H), 2.17 – 2.07 (m, 1H), 1.88 – 1.73 (m, 3H), 1.58 (s, 6H), 1.24 (t, *J* = 7.1 Hz, 3H).

^{13}C NMR (126 MHz, $CDCl_3$) δ 173.4, 152.5, 140.1, 138.6, 136.5, 129.1, 128.3, 127.9, 124.8, 124.5, 118.0, 78.0, 60.3, 43.8, 32.2, 28.7, 24.4, 19.9, 13.0.

HRMS (ESI-TOF, m/z) calculated for $C_{22}H_{26}NaO_3^+$ $[M+Na]^+$ 361.1774, found 361.1769.

4-(1-(4-chlorophenyl)ethyl)phenol (106)

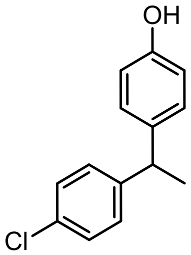

A Schlenk tube (10 mL) was charged phenol (**1p**, 28.2 mg, 0.30 mmol), 1-chloro-4-ethylbenzene (**2e**, 126.5 mg, 0.90 mmol), $FeCl_3$ $6H_2O$ (16.2 mg, 0.060 mmol) and $CHCl_3$ (0.75 mL). The mixture was degassed *via* three freeze-pump-thaw cycles, then filled with dry air. The Schlenk tube was positioned approximately 5 cm away from a 50 W LED

lamp (λ_{max} = 410 nm). After being stirred at 25 ℃ for 48 h, the reaction mixture was concentrated to dryness. Purification using silica gel column chromatography (elution with PE:DCM = 2:1) gave the pure product **106** as a colorless oil (43.2 mg, 0.186 mmol, 62% yield).

^{1}H NMR (500 MHz, $CDCl_3$) δ 7.28 (d, *J* = 4.9 Hz, 2H), 7.15 (d, *J* = 8.4 Hz, 2H), 7.08 (d, *J* = 8.4 Hz, 2H), 6.80 – 6.77 (m, 2H), 4.83 (br, s, 1H), 4.09 (q, *J* = 7.2 Hz, 1H), 1.61 (d, *J* = 7.2 Hz, 3H).

^{13}C NMR (126 MHz, $CDCl_3$) δ 153.9, 145.3, 138.2, 131.7, 128.9, 128.7, 128.4, 115.3, 43.4, 22.0.

IR (film): *ν* (cm^{-1}) 3234, 3221, 3200, 2945, 2939, 1758, 1622, 1501, 1285, 1229, 1100, 832, 604, 535, 498, 478.

HRMS (ESI-TOF, m/z) calculated for $C_{14}H_{14}ClO^+$ $[M+H]^+$ 233.0728, found 233.0720.

ethyl 2-(4-(1-(4-chlorophenyl)ethyl)phenoxy)-2-methylpropanoate (107)

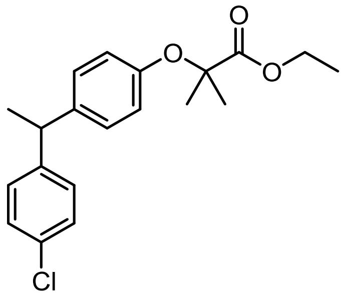

According to the published procedure, [7] a solution of 4-(1-(4-chlorophenyl)ethyl)phenol (**106**, 65.0 mg, 0.28 mmol) in DMF (2.0 mL), $MgSO_4$ (35.0 mg, 0.29 mmol), K_2CO_3 (157.0 mg, 1.12 mmol) and *tert*-butyl *α*-bromoisobutyrate (**104**, 266 μL, 1.4 mmol) were added and the reaction was stirred at 100 ℃ for 24 h. After that time, the reaction was quenched with water (30 mL) and extracted three times with ethyl acetate (3 × 20 mL). The combined organic layers were dried over $MgSO_4$, the solvent was removed under reduced pressure. Purification using silica gel column chromatography (elution with PE:EtOAc = 5:1) gave the pure product **107** as a colorless oil (74.6 mg, 0.216 mmol, 77% yield).

^{1}H NMR (500 MHz, $CDCl_3$) δ 7.25 – 7.21 (m, 2H), 7.11 (d, *J* = 8.4 Hz, 2H), 7.04 (d, *J* = 8.6 Hz, 2H), 6.78 – 6.74 (m, 2H), 4.22 (q, *J* = 7.1 Hz, 2H), 4.05 (q, *J* = 7.2 Hz, 1H), 1.58 – 1.56 (m, 9H), 1.24 (t, *J* = 7.1 Hz, 3H).

^{13}C NMR (126 MHz, $CDCl_3$) δ 174.3, 153.7, 145.1, 139.4, 131.7, 128.9, 128.4, 128.1, 119.2, 79.1, 61.4, 43.4, 25.4, 25.4, 22.0, 14.1.

MS (ESI) mass calculated for $C_{20}H_{23}ClNaO_3^+$ $[M+Na]^+$ 369.1228, found 369.1232.

3-cyclohexyl-4-methoxy-1*H*-indole (108)

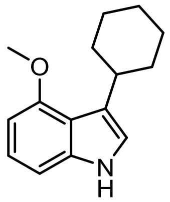

A Schlenk tube (10 mL) was charged with 4-methoxyindole (**1zv**, 44.2 mg, 0.30 mmol), cyclohexane (**2zh**, 0.75 mL) and $FeBr_3$ (32.5 mg, 0.11 mmol). The mixture was degassed *via* three freeze-pump-thaw cycles, then filled with dry air. The Schlenk tube was positioned approximately 5 cm away from a 40 W LED lamp (λ_{max} = 370 nm). After being stirred at 25 ℃ for 120 h, the reaction mixture was concentrated to dryness. Purification using silica gel column chromatography (elution with PE:DCM = 2:1) gave the pure product **108** as a white solid (26.1 mg, 0.114 mmol, 38% yield).

^{1}H NMR (500 MHz, $CDCl_3$) δ 7.81 (s, 1H), 7.07 (t, *J* = 7.9 Hz, 1H), 6.93 (d, *J* = 8.1 Hz, 1H), 6.81 (d, *J* = 2.1 Hz, 1H), 6.48 (d, *J* = 7.8 Hz, 1H), 3.92 (s, 3H), 3.12 (tt, *J* = 11.6, 3.1 Hz, 1H), 2.17 – 2.12 (m, 2H), 1.84 (dd, *J* = 9.7, 6.5 Hz, 2H), 1.79 – 1.74 (m, 1H), 1.47 (qd, *J* = 9.7, 6.4 Hz, 2H), 1.38 – 1.32 (m, 2H), 1.27 (dt, *J* = 12.8, 3.7 Hz, 1H).

^{13}C NMR (126 MHz, $CDCl_3$) δ 154.9, 138.0, 124.3, 122.5, 117.9, 116.9, 104.4, 99.4, 55.2, 36.3, 34.9, 27.1, 26.7.

IR (film): *v* (cm^{-1}) 3924, 3101, 2061, 3056, 3001, 2900, 2886, 2812, 2566, 1958, 1950, 1403, 1397, 1343, 1303, 990, 876, 755, 693.

HRMS (ESI-TOF, m/z) calculated for $C_{15}H_{20}NO^+$ $[M+H]^+$ 230.1539, found 230.1541.

3-cyclohexyl-1-(3-fluoro-4-methylphenyl)-4-methoxy-1*H*-indole (110)

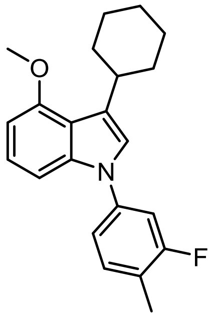

According to the published procedure, [8] an oven dried two-necked round bottom flask was charged with 2-fluoro-4-iodo-1-methylbenzene (**109**, 236.0 mg, 1.0 mmol), Cu-NP (101.7 mg, 1.6 mmol) and K_3PO_4 (424.5 mg, 2.0 mmol), evacuated and backfilled with an argon atmosphere. 3-cyclohexyl-4-methoxy-1*H*-indole (**108**, 229.2 mg, 1.0 mmol) and DMSO (2.0 mL) were added under argon. The flask was then immersed in a preheated oil bath at 80 ℃ until the conversion was completed. The cooled mixture was partitioned between EtOAc (10

mL) and saturated NH_4Cl (10 mL). The aqueous layer was extracted with EtOAc (2 × 10 mL), the organic layer was washed with brine (20 mL), dried over anhydrous Na_2SO_4 and concentrated in vacuum. Purification using silica gel column chromatography (elution with PE:EtOAc = 1:1) gave the pure product **110** as a white solid (276.3 mg, 0.819 mmol, 82% yield).

^{1}H NMR (500 MHz, $CDCl_3$) δ 7.29 – 7.27 (m, 1H), 7.24 (dd, *J* = 7.9, 3.7 Hz, 1H), 7.12 – 7.08 (m, 2H), 7.04 (d, *J* = 8.2 Hz, 1H), 6.90 (s, 1H), 6.54 (d, *J* = 7.6 Hz, 1H), 3.95 (s, 3H), 3.17 (tt, *J* = 11.5, 2.9 Hz, 1H), 2.34 (s, 3H), 2.18 (d, *J* = 12.1 Hz, 2H), 1.84 (d, *J* = 13.0 Hz, 2H), 1.77 (d, *J* = 12.8 Hz, 1H), 1.48 (tdd, *J* = 12.8, 9.7, 3.1 Hz, 2H), 1.41 – 1.34 (m, 2H), 1.30 – 1.26 (m, 1H).

^{13}C NMR (126 MHz, $CDCl_3$) δ 155.1, 138.0, 136.1, 127.6 (d, J_{C-F} = 25.0 Hz), 126.3, 126.1, 125.2, 123.5 (d, J_{C-F} = 35.0 Hz), 123.0, 122.4, 118.2, 115.9 (d, J_{C-F} = 95.0 Hz), 103.6, 100.2, 55.4, 36.4, 35.0, 27.2, 26.8, 14.8.

HRMS (ESI-TOF, m/z) calculated for $C_{22}H_{25}FNO^+$ $[M+H]^+$ 338.1915, found 338.1919.

3.3 Unsuccessful Substrates

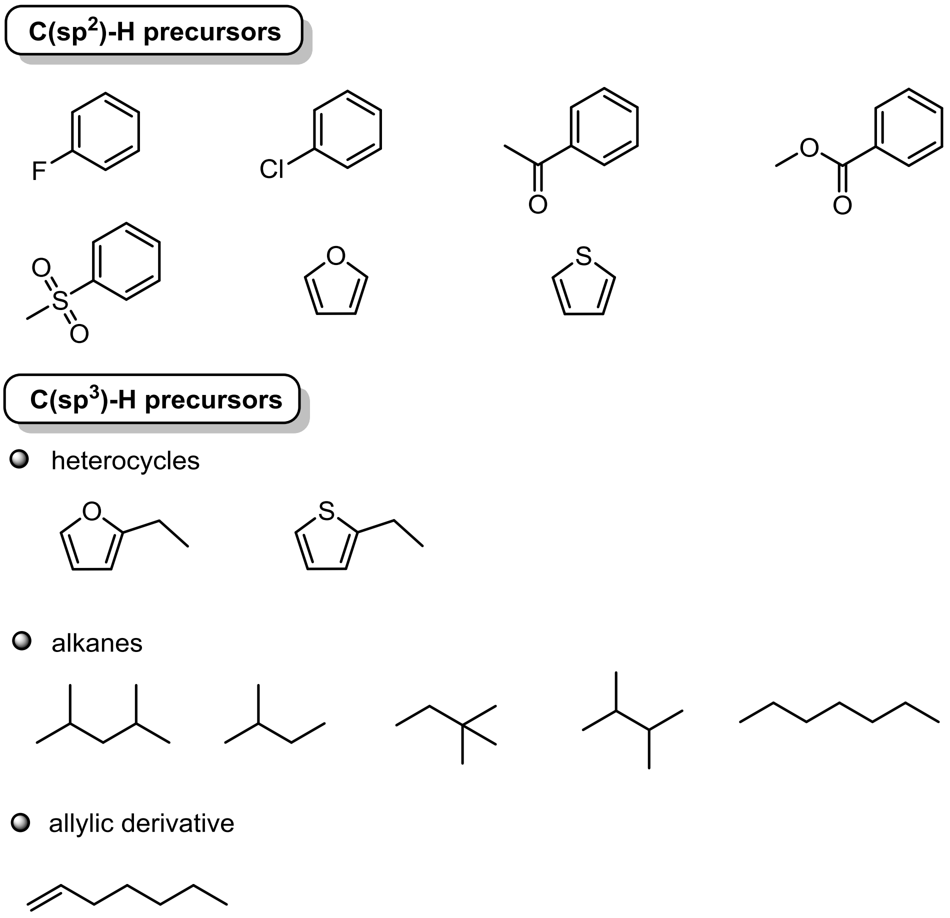

3.4 A Scale-up Reaction

3-cyclohexyl-4-methoxy-1*H*-indole (108)

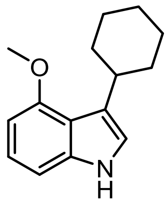

A solvent-free reaction: A Schlenk tube (10 mL) was charged with 4-methoxyindole (**1zv**, 839.8 mg, 5.7 mmol), cyclohexane (**2zh**, 20 mL) and $FeBr_3$ (591.0 mg, 0.11 mmol). The mixture was degassed *via* three freeze-pump-thaw cycles, then filled with dry air. The Schlenk tube was positioned approximately 5 cm away from a 40 W LED lamp (λ_{max} = 370 nm). After being stirred at 25 ℃ for 120 h, the reaction mixture was concentrated to dryness. Purification using silica gel column chromatography (elution with PE:DCM = 2:1) gave the

pure product **108** as a white solid (444.1 mg, 1.938 mmol, 34% yield).

A reaction in dichloromethane: A Schlenk tube (10 mL) was charged with 4-methoxyindole (**1zv**, 839.8 mg, 5.7 mmol), cyclohexane (**2zh**, 4.82 g, 57 mmol) and $FeBr_3$ (591.0 mg, 0.11 mmol). The mixture was degassed *via* three freeze-pump-thaw cycles, then filled with dry air. The Schlenk tube was positioned approximately 5 cm away from a 40 W LED lamp (λ_{max} = 370 nm). After being stirred at 25 ℃ for 120 h, the reaction mixture was concentrated to dryness. Purification using silica gel column chromatography (elution with PE:DCM = 2:1) gave the pure product **108** as a white solid (156.6 mg, 0.684 mmol, 12% yield).

3.5 Identification of Side Products

In the reaction of **1a+2a→3**, trace amounts of chloro-substituted products (**91**, **111**) and a self-coupling product (**92**) were detected and identified. Similarly, in the reaction of **1p+2a→18**, analogous side-products (**91**, **92**, **112**) were observed.

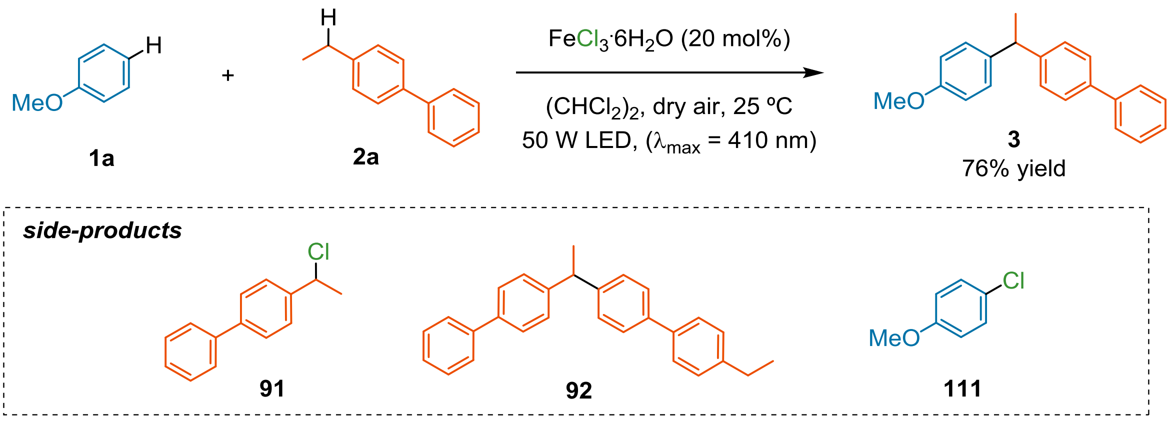

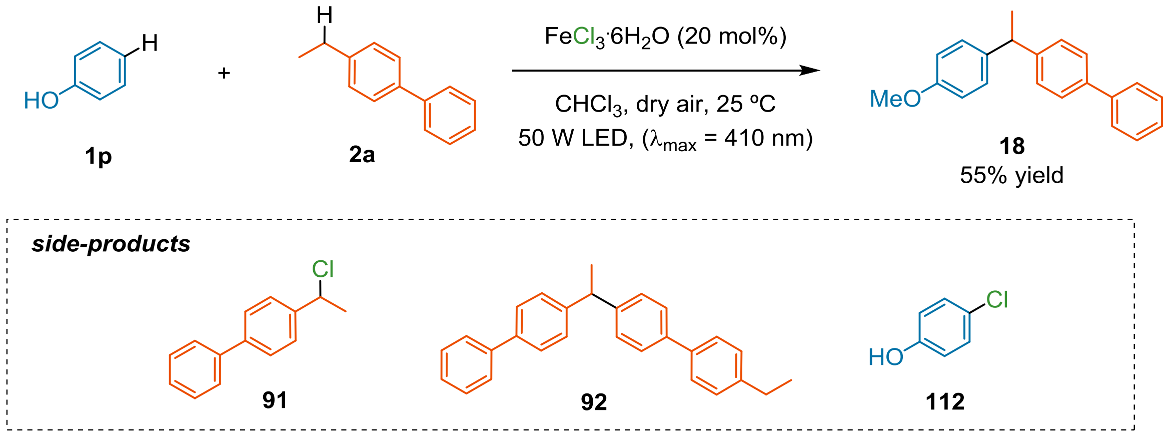

1-chloro-4-methoxybenzene (111)

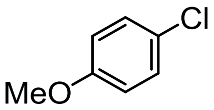

^{1}H NMR (500 MHz, $CDCl_3$) δ 7.24 – 7.18 (m, 2H), 6.83 – 6.78 (m, 2H), 3.76 (s, 3H).

^{13}C NMR (126 MHz, $CDCl_3$) δ 158.3, 129.4, 125.7, 115.3, 55.61.

HRMS (ESI-TOF, m/z) calculated for $C_7H_8ClO^+$ $[M+H]^+$ 143.0258, found 143.0233.

4-chlorophenol (112)

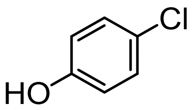

^{1}H NMR (500 MHz, $CDCl_3$) δ 7.20 – 7.16 (m, 2H), 6.79 – 6.75 (m, 2H), 6.34 (s, 1H).

^{13}C NMR (126 MHz, $CDCl_3$) δ154.2, 129.6, 125.7, 116.8.

HRMS (ESI-TOF, m/z) calculated for $C_6H_6ClO^+$ $[M+H]^+$ 129.0102, found 129.0100.

4. Mechanistic Investigations

4.1 Radical Probing Experiments

4.1.1 Probing Benzyl Radicals

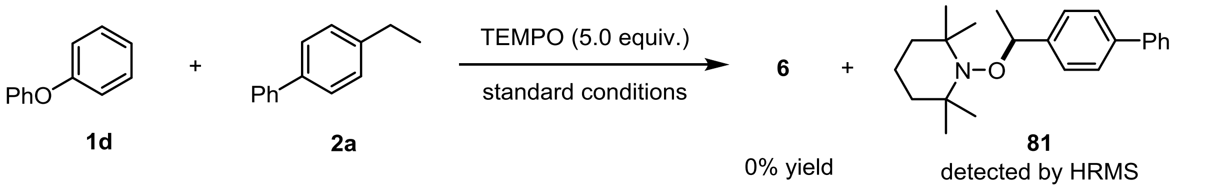

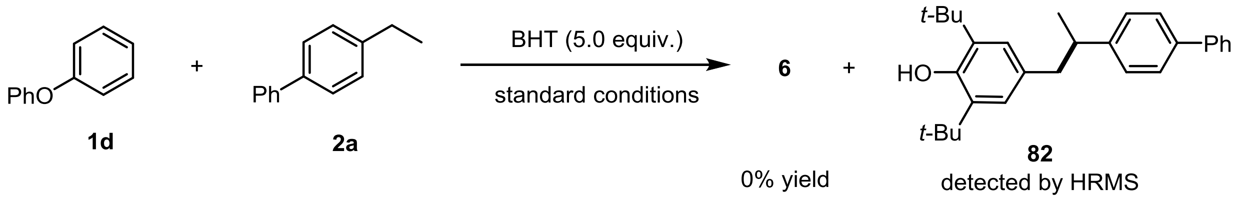

A Schlenk tube (10 mL) was charged with diphenyl ether (**1d**, 51.1 mg, 0.30 mmol), 4-ethylbiphenyl (**2a**, 164.0 mg, 0.90 mmol), $FeCl_3$ $6H_2O$ (16.2 mg, 0.060 mmol) and $(CHCl_2)_2$ (0.75 mL). 2,2,6,6-Tetramethylpiperidine-1-oxyl (TEMPO, 234.5 mg, 1.50 mmol) or butylated hydroxytoluene (BHT, 330.5 mg, 1.50 mmol) was then added. The mixture was degassed *via* three freeze-pump-thaw cycles, and filled with dry air. The Schlenk tube was positioned approximately 5 cm away from a 50 W LED lamp (λ_{max} = 410 nm). After being stirred at 25 ℃ for 1 h, the reaction mixture was detected by HRMS.

For compound **81**, HRMS (ESI-TOF, m/z): calculated for $C_{23}H_{32}NO^+$ $(M+H)^+$: 338.2478, found: 338.2480. For compound **82**, HRMS (ESI-TOF, m/z): calculated for $C_{29}H_{37}O^+$ $(M+H)^+$: 401.2839, found: 401.2829.

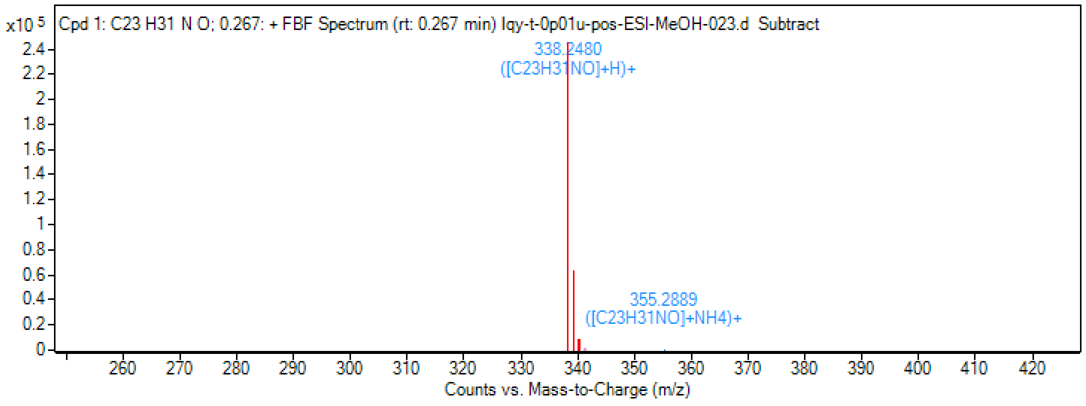

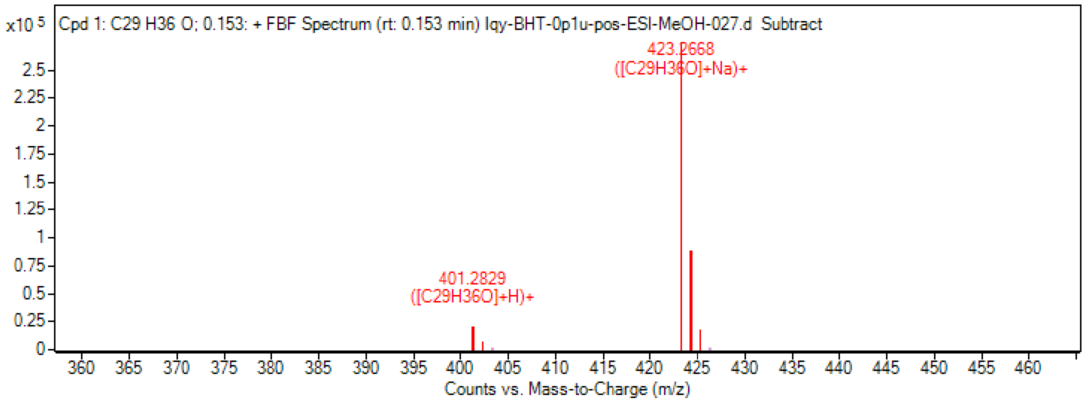

4.1.2 Probing Chlorine Radicals

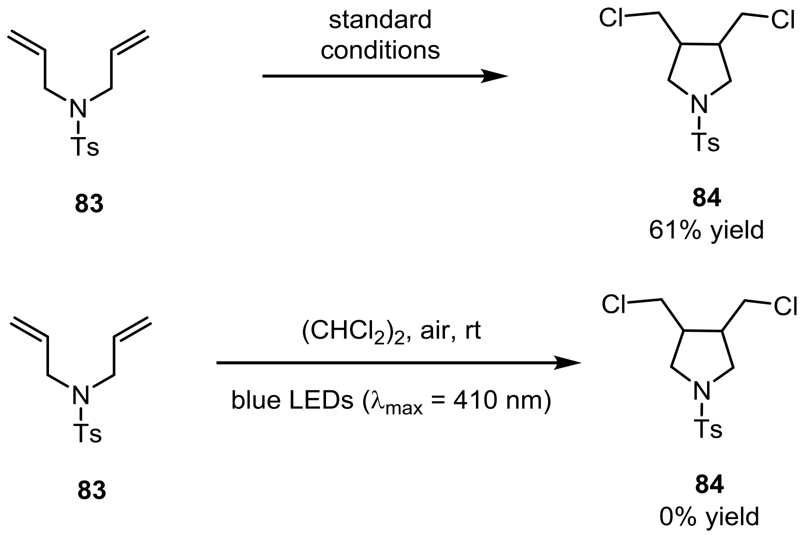

A Schlenk tube (10 mL) was charged with *N*,*N*-diallyl-4-methylbenzenesulfonamide (**83**, 75.3 mg, 0.30 mmol), $FeCl_3 \cdot 6H_2O$ (16.2 mg, 0.060 mmol) and $(CHCl_2)_2$ (0.75 mL). The mixture was degassed *via* three freeze-pump-thaw cycles, then filled with dry air. The Schlenk tube was positioned approximately 5 cm away from a 50 W LED lamp (λ_{max} = 410 nm). After being stirred at 25 ℃ for 48 h, the reaction mixture was concentrated to dryness. Purification using silica gel column chromatography (elution with PE:EtOAc = 3:1) gave the pure product **84** as a white solid (58.8 mg, 0.183 mmol, 61% yield).

3,4-bis(chloromethyl)-1-tosylpyrrolidine (84)

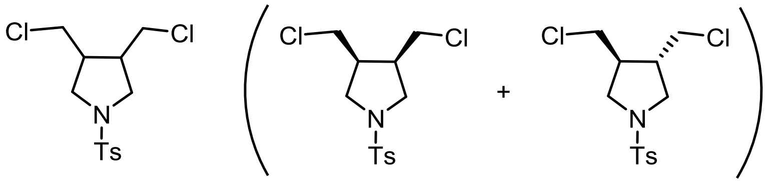

^{1}H NMR (500 MHz, $CDCl_3$) δ 7.71 (t, *J* = 8.7 Hz, 2H), 7.34 (d, *J* = 8.0 Hz, 2H), 3.50 – 3.43 (m, 4H), 3.31 – 3.24 (m, 3H), 3.12 (dd, *J* = 10.3, 6.1 Hz, 1H), 2.64 – 2.60 (m, 1H), 2.43 (s, 3H), 2.42 – 2.34 (m, 1H).

^{13}C NMR (126 MHz, $CDCl_3$) δ 144.1, 144.0, 133.5, 132.7, 129.9, 129.9, 127.9, 127.6, 51.2, 50.7, 45.3, 43.9, 43.3, 42.0, 21.7, 21.7.

HRMS (ESI-TOF, m/z) calculated for $C_{13}H_{18}Cl_2NO_2S^+$ $[M+H]^+$ 322.0430, found 322.0433.

Remarks: In the absence of $FeCl_3 \cdot 6H_2O$, compound **84** was not detected, indicating that chorine radicals derived from the iron salt rather than the solvent.

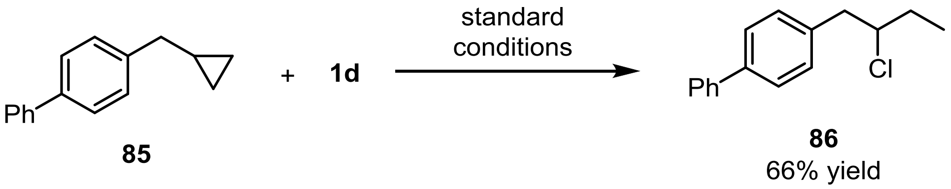

A Schlenk tube (10 mL) was charged with diphenyl ether (**1d**, 51.1 mg, 0.30 mmol), 4-(cyclopropylmethyl)-1,1'-biphenyl (**85**, 187.5 mg, 0.90 mmol), $FeCl_3 \cdot 6H_2O$ (16.2 mg, 0.060 mmol) and $(CHCl_2)_2$ (0.75 mL). The mixture was degassed *via* three freeze-pump-thaw cycles, then filled with dry air. The Schlenk tube was positioned approximately 5 cm away from a 50 W LED lamp (λ_{max} = 410 nm). After being stirred at 25 ℃ for 48 h, the reaction mixture was concentrated to dryness. Purification using silica gel column chromatography (elution with PE:DCM = 5:1) gave the pure product **86** as a white solid (48.3 mg, 0.198 mmol, 66% yield).

4-(2-chlorobutyl)-1,1'-biphenyl (86)

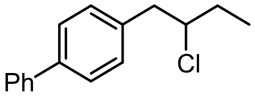

^{1}H NMR (500 MHz, $CDCl_3$) δ 7.63 – 7.58 (m, 2H), 7.56 (d, *J* = 8.1 Hz, 2H), 7.45 (t, *J* = 7.6 Hz, 2H), 7.39 – 7.27 (m, 3H), 4.16 – 4.04 (m, 1H), 3.10 (d, *J* = 6.9 Hz, 2H), 1.91 (dtd, *J* = 14.4, 7.2, 3.3 Hz, 1H), 1.74 (dt, *J* = 15.2, 7.6 Hz, 1H), 1.10 (t, *J* = 7.3 Hz, 3H).

^{13}C NMR (126 MHz, $CDCl_3$) δ 141.0, 139.8, 137.3, 129.9, 128.9, 127.3, 127.3, 127.2, 65.7, 44.41, 30.86, 11.08.

HRMS (ESI-TOF, m/z) calculated for $C_{16}H_{18}Cl^+$ $[M+H]^+$ 245.1092, found 245.1097.

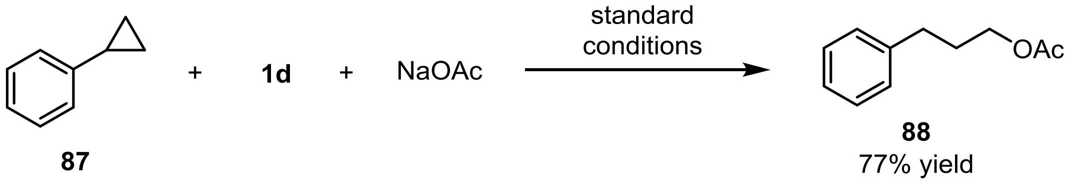

A Schlenk tube (10 mL) was charged with diphenyl ether (**1d**, 51.1 mg, 0.30 mmol), cyclopropylbenzene (**87**, 106.3 mg, 0.90 mmol), $FeCl_3\ 6H_2O$ (16.2 mg, 0.060 mmol) and $(CHCl_2)_2$ (0.75 mL). NaOAc (204.1 mg, 1.5 mmol) was then added. The mixture was degassed *via* three freeze-pump-thaw cycles, then filled with dry air. The Schlenk tube was positioned approximately 5 cm away from a 50 W LED lamp (λ_{max} = 410 nm). After being stirred at 25 ℃ for 48 h, the reaction mixture was concentrated to dryness. Purification using silica gel column chromatography (elution with PE:DCM = 3:1) gave the pure product **88** as a coloress oil (41.1 mg, 0.231 mmol, 77% yield).

A plausible mechanism for the formation of product 88:

According to a relevant literature supplementary reference, [15] we proposed a plausible mechanism for the formation of product **88**. Initially, cyclopropylbenzene (**87**) undergoes a single electron transfer with the chlorine radical generated in situ, resulting in the formation of a radical cation (**Int-A**) and a chloride anion. Subsequently, the intermediate **Int-A** is subjected to nucleophilic attack by either the chloride anion or the acetate ion, leading to the formation of a ring-opening radical species (**Int-B** or **Int-C**). The final step involves H-abstraction from the substrates or other reaction components, ultimately leading to the formation of either product **88** or a chloride analog. The latter can then undergo an S_N2 reaction with NaOAc to yield **88**. Although this mechanism initiates with a SET rather than an HAT process as proposed in the photochemical C(sp^2)-H alkylation, it can indirectly suggest the involvement of chlorine radicals.

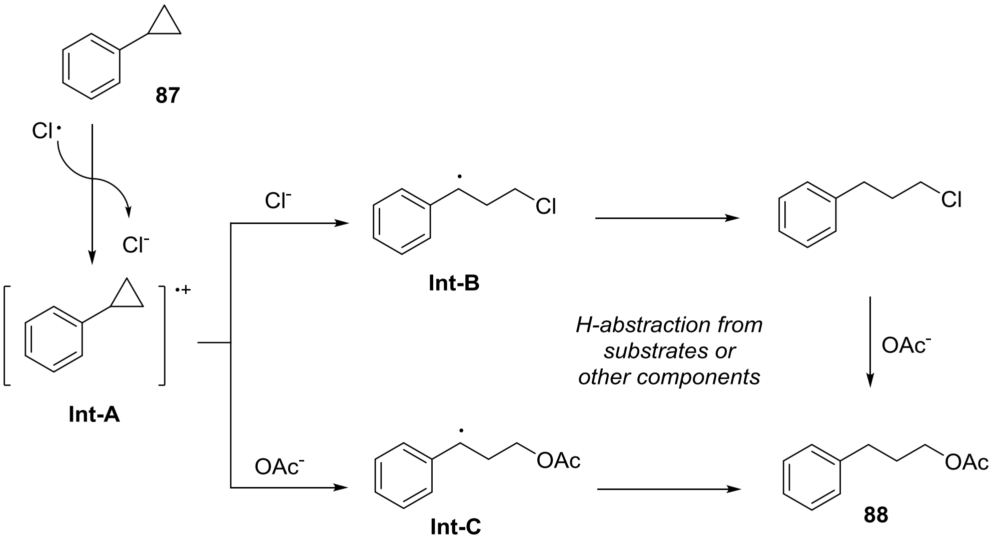

A related ref. (from **87** to **Int-B**): *Angew. Chem. Int. Ed.* **2019**, *58*, 8577–8780

3-phenylpropyl acetate (88)

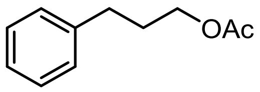

^{1}H NMR (500 MHz, CDCl3) δ 7.33 – 7.29 (m, 2H), 7.24 – 7.20 (m, 2H), 7.20 (s, 1H), 4.11 (t, J = 6.6 Hz, 2H), 2.73 – 2.69 (m, 2H), 2.06 (s, 3H), 1.98 (ddt, J = 13.1, 9.1, 6.5 Hz, 2H).

^{13}C NMR (126 MHz, CDCl3) δ 171.1, 141.2, 128.5, 128.4, 126.0, 63.9, 32.2, 30.2, 21.0.

HRMS (ESI-TOF, m/z) calculated for $C_{11}H_{15}O_2^+$ $[M+H]^+$ 179.2385, found 179.2389.

4.2 Light-Off/on Experiments

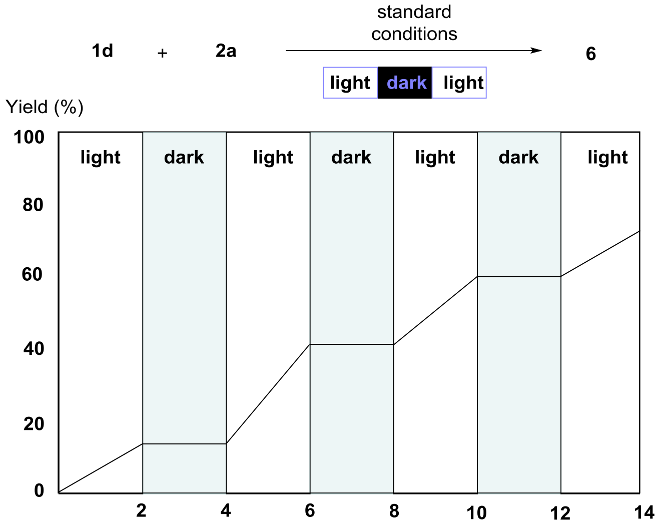

Supplementary Fig. 1 Light-off/on experiments

A Schlenk tube (10 mL) was charged with diphenyl ether (**1d**, 51.1 mg, 0.30 mmol), 4-ethylbiphenyl (**2a**, 164.0 mg, 0.90 mmol), $FeCl_3 \cdot 6H_2O$ (16.2 mg, 0.060 mmol) and $(CHCl_2)_2$ (0.75 mL). The mixture was degassed *via* three freeze-pump-thaw cycles, then filled with dry air. The Schlenk tube was positioned approximately 5 cm away from a 50 W LED lamp (λ_{max} = 410 nm). The reaction was stirred at room temperature for the indicated time. A solution (0.10 mL) was taken from the reaction mixture, and the yields were determined by crude ^{1}H NMR analysis using 1,3,5-trimethoxybenzene as an internal standard.

4.3 XPS Spectroscopy

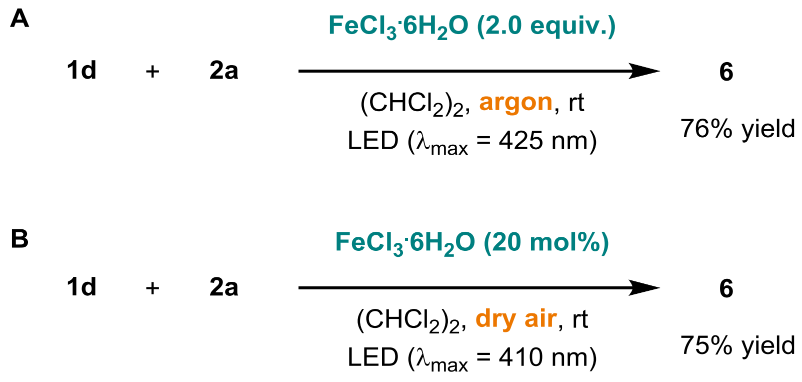

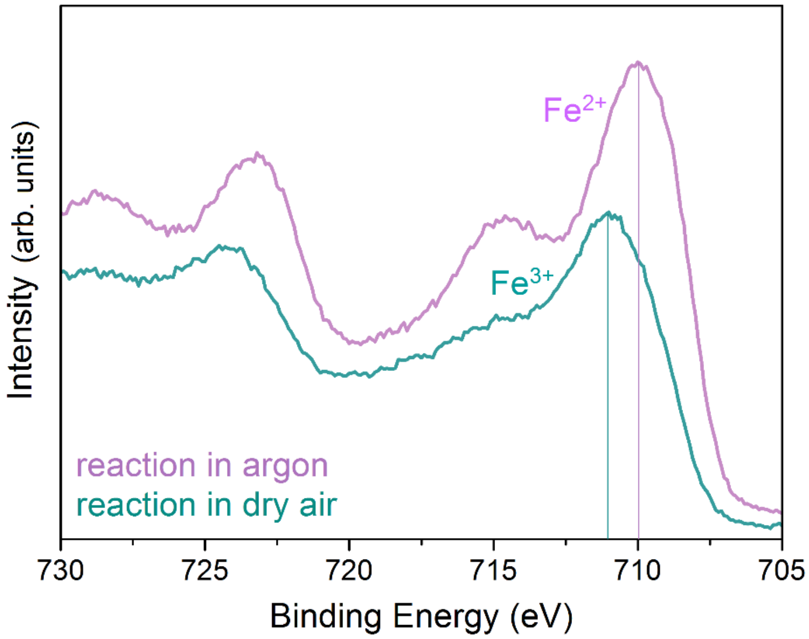

Supplementary Fig. 2 XPS analysis of reaction residues

Sample A (a residue derived from the reaction in argon): A Schlenk tube (10 mL) was charged with diphenyl ether (**1d**, 51.1 mg, 0.30 mmol), 4-ethylbiphenyl (**2a**, 164.0 mg, 0.90 mmol), $FeCl_3$ $6H_2O$ (162.2 mg, 0.60 mmol) and $(CHCl_2)_2$ (0.75 mL). The mixture was degassed *via* three freeze-pump-thaw cycles, then filled with argon. The Schlenk tube was positioned approximately 5 cm away from a 50 W LED lamp (λ_{max} = 425 nm). After being stirred at 25 ℃ for 48 h, the reaction mixture was filtered in argon and the residue was used for the X-ray photoelectron spectroscopy (XPS) analysis.

Sample B (a residue derived from the reaction in dry air):A Schlenk tube (10 mL) was charged with diphenyl ether (**1d**, 51.1 mg, 0.30 mmol), 4-ethylbiphenyl (**2a**, 164.0 mg, 0.90 mmol), $FeCl_3$ $6H_2O$ (16.2 mg, 0.060 mmol) and $(CHCl_2)_2$ (0.75 mL). The mixture was degassed *via* three freeze-pump-thaw cycles, then filled with dry air. The Schlenk tube was positioned approximately 5 cm away from a 50 W LED lamp (λ_{max} = 410 nm). After being

stirred at 25 ℃ for 48 h, the reaction mixture was filtered in argon and the residue was used for the XPS analysis.

4.4 UV/Vis-Absorption Spectra of the Reaction Components

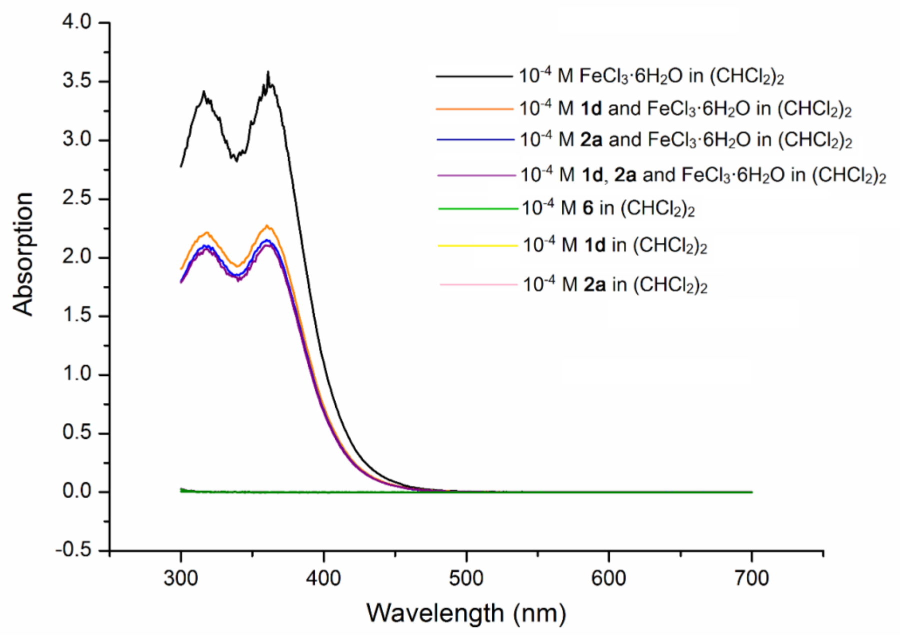

Supplementary Fig. 3 UV-Vis spectra of the reaction components

Preparation of samples for the measurement of UV-Vis spectra:

2-Ethylbenzene 2a in $(CHCl_2)_2$ (1.0×10^{-4} M): 2-ethylbenzene **2a** was dissolved in distilled $(CHCl_2)_2$ (10 mL).

Diphenyl ether 1d in $(CHCl_2)_2$ (1.0×10^{-4} M): diphenyl ether **1d** was dissolved in distilled $(CHCl_2)_2$ (10 mL).

$FeCl_3$ $6H_2O$ in $(CHCl_2)_2$ (1.0×10^{-4} M): $FeCl_3$ $6H_2O$ was dissolved in distilled $(CHCl_2)_2$ (10 mL).

2-Ethylbenzene 2a + $FeCl_3$ $6H_2O$ in $(CHCl_2)_2$ (1.0×10^{-4} M): 2-ethylbenzene **2a** and $FeCl_3$ $6H_2O$ were dissolved in distilled $(CHCl_2)_2$ (10 mL).

Diphenyl ether 1d + $FeCl_3$ $6H_2O$ in $(CHCl_2)_2$ (1.0×10^{-4} M): diphenyl ether **1d** and $FeCl_3$ $6H_2O$ were dissolved in distilled $(CHCl_2)_2$ (10 mL).

2-Ethylbenzene 2a + diphenyl ether 1d + $FeCl_3$ $6H_2O$ in $(CHCl_2)_2$ (1.0×10^{-4} M): 2-ethylbenzene **2a**, diphenyl ether **1d** and $FeCl_3$ $6H_2O$were dissolved in distilled $(CHCl_2)_2$ (10 mL).

Product 6 in $(CHCl_2)_2$ (1.0×10^{-4} M): product **6** was dissolved in distilled $(CHCl_2)_2$ (10 mL).

4.5 Reaction Interfered with Competitive Nucleophiles

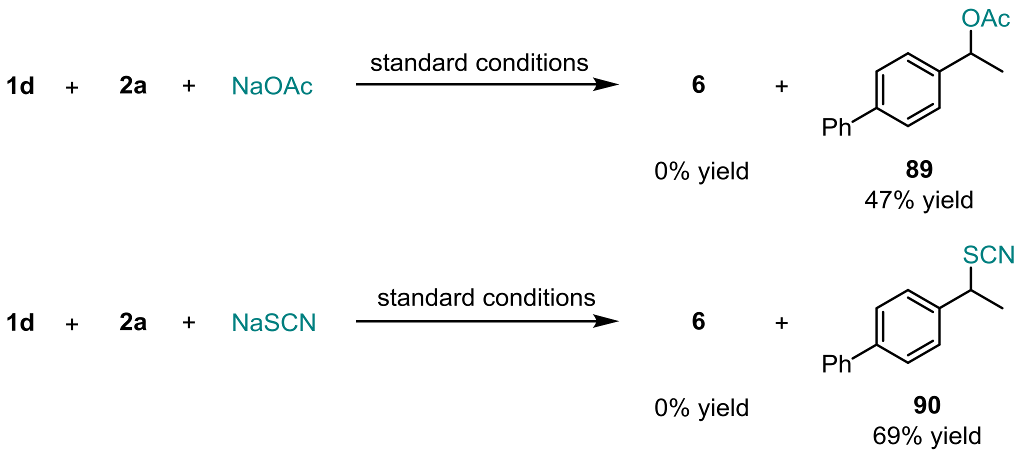

A Schlenk tube (10 mL) was charged with diphenyl ether (**1d**, 51.1 mg, 0.30 mmol), 4-ethylbiphenyl (**2a**, 164.0 mg, 0.90 mmol), $FeCl_3$ $6H_2O$ (16.2 mg, 0.060 mmol) and $(CHCl_2)_2$ (0.75 mL). Then NaOAc (204.1 mg, 1.5 mmol) or NaSCN (121.5 mg, 1.5 mmol) was added. The mixture was degassed *via* three freeze-pump-thaw cycles, then filled with dry air. The Schlenk tube was positioned approximately 5 cm away from a 50 W LED lamp (λ_{max} = 410 nm). After being stirred at 25 ℃ for 36 h, the reaction mixture was concentrated to dryness. Purification using silica gel column chromatography (elution with PE:EtOAc = 5:1) gave the pure product **89** as a colorless oil (33.9 mg, 0.141 mmol, 47% yield), or **90** as a white solid (49.5 mg, 0.207 mmol, 69% yield).

1-([1,1'-biphenyl]-4-yl)ethyl acetate (89)

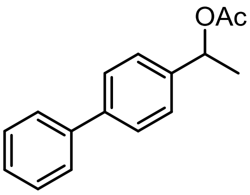

^{1}H NMR (500 MHz, CDC13) δ 7.57 (d, *J* = 7.9 Hz, 4H), 7.44 (t, *J* = 7.6 Hz, 4H), 7.35 (t, *J* = 7.4 Hz, 1H), 5.93 (q, *J* = 6.6 Hz, 1H), 2.09 (s, 3H), 1.57 (d, *J* = 4.0 Hz, 3H).

^{13}C NMR (126 MHz, CDC13) δ 170.5, 141.0, 140.9, 140.8, 128.9, 1275, 127.4, 127.3, 126.7, 72.3, 22.3, 21.5.

HRMS (ESI-TOF, m/z) calculated for $C_{16}H_{16}NaO_2^+$ $[M+Na]^+$ 263.1043, found 263.1049.

4-(1-thiocyanatoethyl)-1,1'-biphenyl (90)

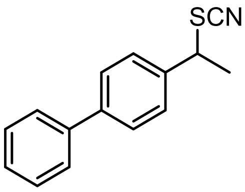

^{1}H NMR (500 MHz, $CDCl_3$) δ 7.61 – 7.56 (m, 4H), 7.44 (t, J = 7.6 Hz, 2H), 7.40 – 7.34 (m, 3H), 4.95 (q, J = 6.8 Hz, 1H), 1.70 (d, J = 6.8 Hz, 3H).

^{13}C NMR (126 MHz, $CDCl_3$) δ 141.4, 140.5, 139.3, 129.0, 127.8, 127.7, 127.2, 126.0, 56.9, 25.1.

HRMS (ESI-TOF, m/z) calculated for $C_{15}H_{13}NNaS^{+}$ $[M+Na]^{+}$ 262.0661, found 262.0666.

4.6 Kinetic Isotope Effect (KIE) Experiments

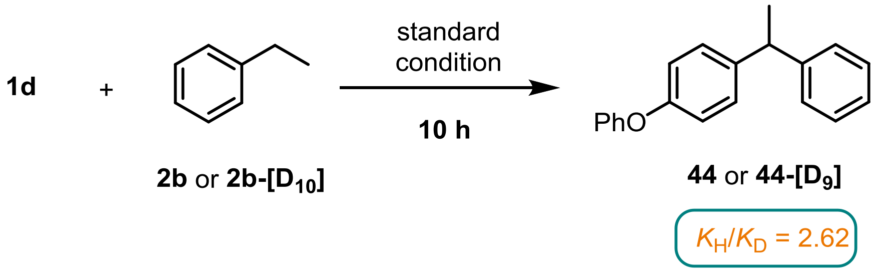

A Schlenk tube (10 mL) was charged with diphenyl etherscale (**1d**, 51.1 mg, 0.30 mmol), ethylbenzene (**2b**, 95.6 mg, 1.0 mmol) or ethylbenzene-[D_{10}] (**2b-[D_{10}]**, 104.4 mg, 1.0 mmol), $FeCl_3$ $6H_2O$ (16.2 mg, 0.060 mmol) and $(CHCl_2)_2$ (0.75 mL). The mixture was degassed *via* three freeze-pump-thaw cycles, then filled with dry air. The Schlenk tube was positioned approximately 5 cm away from a 50 W LED lamp (λ_{max} = 410 nm). The reaction was stirred at room temperature for 10 h. The reaction mixture of hydrogen to deuterium in the product was determined as 2.62:1, thus the KIE value was K_H/K_D = 2.62.

4.7 Probing Other Intermediates

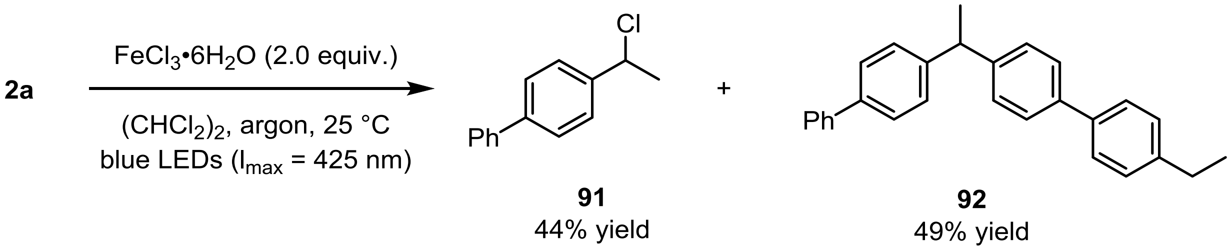

A Schlenk tube (10 mL) was charged with 4-ethylbiphenyl (**2a**, 54.6 mg, 0.30 mmol), $FeCl_3$ $6H_2O$ (162.2 mg, 0.60 mmol) and $(CHCl_2)_2$ (0.75 mL). The mixture was degassed *via*

three freeze-pump-thaw cycles, then filled with an argon atmosphere. The Schlenk tube was positioned approximately 5 cm away from a 50 W LED lamp (λ_{max} = 425 nm). After being stirred at 25 ℃ for 24 h, the reaction mixture was concentrated to dryness. Purification using silica gel column chromatography (elution with PE:DCM = 5:1) gave the pure product **91** as a white solid (28.5 mg, 0.132 mmol, 44% yield) and **92** as a white solid (53.2 mg, 0.147 mmol, 49% yield).

4-(1-chloroethyl)-1,1'-biphenyl (91)

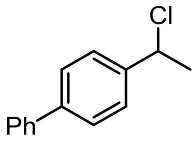

^{1}H NMR (500 MHz, $CDCl_3$) δ 7.62 – 7.58 (m, 4H), 7.47 – 7.43 (m, 4H), 7.37 – 7.34 (m, 1H), 4.97 (q, *J* = 7.0 Hz, 1H), 1.55 (d, *J* = 6.8 Hz, 3H).

^{13}C NMR (126 MHz, $CDCl_3$) δ 145.0, 141.0, 140.6, 128.9, 127.9, 127.4, 127.2, 126.0, 60.0, 24.0.

HRMS (ESI-TOF, m/z) calculated for $C_{14}H_{14}Cl^+$ $[M+H]^+$ 217.0779, found 217.0780.

4-(1-([1,1'-biphenyl]-4-yl)ethyl)-4'-ethyl-1,1'-biphenyl (92)

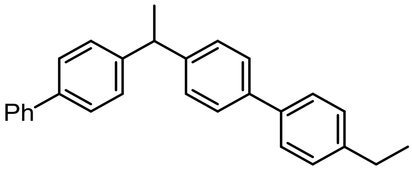

^{1}H NMR (500 MHz, $CDCl_3$) δ 7.62 (d, *J* = 7.3 Hz, 2H), 7.58 – 7.53 (m, 6H), 7.45 (t, *J* = 7.6 Hz, 2H), 7.37 (t, *J* = 7.9 Hz, 5H), 7.29 (d, *J* = 8.0 Hz, 2H), 4.29 – 4.25 (m, 1H), 2.75 – 2.70 (m, 2H), 1.75 (d, *J* = 7.2 Hz, 3H), 1.32 (d, *J* = 7.6 Hz, 3H).

^{13}C NMR (126 MHz, $CDCl_3$) δ 145.6, 145.2, 143.3, 141.1, 139.2, 138.5, 128.9, 128.4, 128.2, 128.1, 127.3, 127.2, 127.2, 127.2, 127.1, 44.3, 28.6, 22.0, 15.7.

HRMS (ESI-TOF, m/z) calculated for $C_{28}H_{27}^+$ $[M+H]^+$ 363.2107, found 363.2110.

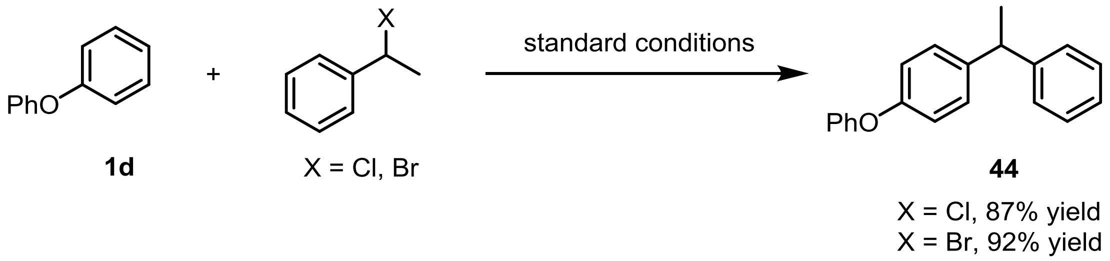

A Schlenk tube (10 mL) was charged with diphenyl ether (**1d**, 51.1 mg, 0.30 mmol), (1-chloroethyl)benzene (126.5 mg, 0.90 mmol) or (1-bromoethyl)benzene (166.6 mg, 0.90 mmol), $FeCl_3$ $6H_2O$ (16.2 mg, 0.060 mmol) and $(CHCl_2)_2$ (0.75 mL). The mixture was degassed *via* three freeze-pump-thaw cycles, then filled with dry air. The Schlenk tube was positioned approximately 5 cm away from a 50 W LED lamp (λ_{max} = 410 nm). After being stirred at 25 ℃ for 48 h, the reaction mixture was concentrated to dryness. Purification using silica gel column chromatography (elution with PE:DCM = 5:1) gave the pure product **44**. Obtained yields of 87% and 92%, respectively.

5. X-Ray Diffraction

5.1 Crystal Structure of Product 35

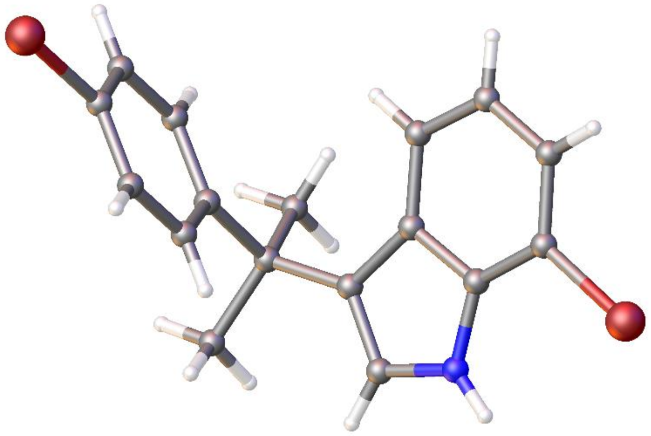

Supplementary Fig. 4 Ortep drawing of compound **35**

A procedure for crystallization. Compound **35** was dissolved in a mixture of $CHCl_3$ (0.5 mL) and hexane (1.0 mL). Single crystals were obtained after evaporation under ambient conditions for 4 days.

Data collection and solution. Data was collected on a XtaLAB Synergy four-circle diffractometer with monochromatic Cu Kα radiation (λ = 1.54184 Å) at 100 K. Data reduction and absorption correction were applied by using the multi-scan program. The structures were determined and refined using full-matrix least-squares based on F2 with SHELXT and SHELXL within Olex2. The structure is shown on Supplementary Fig. 4. Crystallographic data for complex **35** has been deposited with the Cambridge Crystallographic Data Centre as supplementary publication number CCDC 2251704.

5.2 Crystal Structure of Product 38

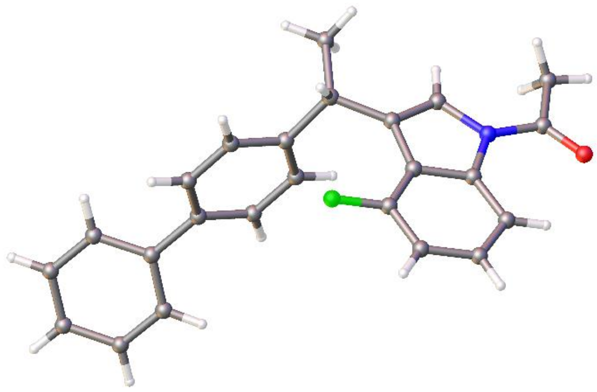

Supplementary Fig. 5 Ortep drawing of compound **38**

A procedure for crystallization. Compound **38** was dissolved in a mixture of $CHCl_3$ (0.5 mL) and hexane (1.0 mL). Single crystals were obtained after evaporation under ambient conditions for 5 days.

Data collection and solution. Data was collected on a XtaLAB Synergy four-circle diffractometer with monochromatic Cu K*α* radiation (λ = 1.54184 Å) at 100 K. Data reduction and absorption correction were applied by using the multi-scan program. The structures were determined and refined using full-matrix least-squares based on F2 with SHELXT and SHELXL within Olex2. The structure is shown on Supplementary Fig. 5. Crystallographic data for complex **38** has been deposited with the Cambridge Crystallographic Data Centre as supplementary publication number CCDC 2251707.

Supplementary Table 2. Data collection and refinement statistics for the compounds **35** and **38**

	**35**	**38**
CCDC	2251704	2251707
Empirical formula	$C_{17}H_{15}Br_2N$	$C_{24}H_{20}FNO$
Formula weight	1572.48	357.41
Temperature (K)	265	272
Crystal system	triclinic	triclinic
Space group	*P*-1	*P*-1
a, *b*, *c* (Å)	10.3195, 17.5630, 19.6201	9.6287, 9.8951, 10.0269
α, *β*, *γ* (°)	68, 75, 89	100, 97, 95
Volume (Å^3)	3176.62	928.57
Z	2	2
Density (calculated, mg/m^3)	1.644	1.278
μ (mm^{-1})	6.374	0.678
F(000)	1552.0	376.0
Crystal size (mm^3)	0.1 × 0.1 × 0.1	0.1 × 0.1 × 0.1
2Θ range for data collection/ °	5.042 to 177.252	9.134 to 150.266
Index ranges	$-12 \leq h \leq 12$, $-21 \leq k \leq 22$, $-24 \leq l \leq 24$	$-12 \leq h \leq 11$, $-12 \leq k \leq 12$, $-9 \leq l \leq 12$
Reflections collected	35049	9769

Independent reflections	12384 [R_{int} = 0.0395, R_{sigma} = 0.0411]	3616 [R_{int} = 0.0244, R_{sigma} = 0.0288]
Completeness	97.7%	99.4%
Absorption correction	multi-scan	multi-scan
Refinement method	Full-matrix least-squares on F^2	Full-matrix least-squares on F^2
Data / restraints / parameters	12384/0/729	3616/0/247
Goodness-of-fit on F^2	1.071	1.058
Final R indices [I>=2σ (I)]	R_1 = 0.0477, wR_2 = 0.1242	R_1 = 0.0427, wR_2 = 0.1210
R indices (all data)	R_1 = 0.0605, wR_2 = 0.1384	R_1 = 0.0493, wR_2 = 0.1276
Largest diff. peak/hole (e.Å^{-3})	0.15/-0.98	0.17/-0.16

5.3 Crystal Structure of Product 56

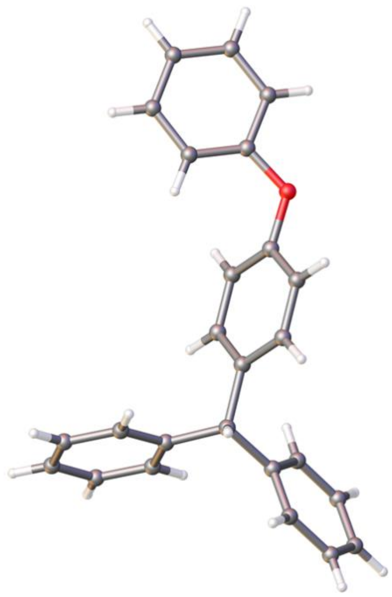

Supplementary Fig. 6 Ortep drawing of compound **56**

A procedure for crystallization. Compound **56** was dissolved in a mixture of $CHCl_3$ (0.5 mL) and hexane (1.0 mL). Single crystals were obtained after evaporation under ambient conditions for 5 days.

Data collection and solution. Data was collected on a XtaLAB Synergy four-circle diffractometer with monochromatic Cu Kα radiation (λ = 1.54184 Å) at 100 K. Data reduction and absorption correction were applied by using the multi-scan program. The structures were determined and refined using full-matrix least-squares based on F2 with SHELXT and SHELXL within Olex2. The structure is shown on Supplementary Fig. 6. Crystallographic data for complex **56** has been deposited with the Cambridge Crystallographic Data Centre as supplementary publication number CCDC 2098093.

Supplementary Table 3. Data collection and refinement statistics for the compounds **56**

	**56**
CCDC	2098093
Empirical formula	$C_{25}H_{20}O$
Formula weight	336.41
Temperature (K)	100.15
Crystal system	monoclinic
Space group	$P2_1/c$
a, b, c (Å)	10.8428, 9.3075, 17.9231
α, β, γ (°)	90, 98, 90
Volume (Å^3)	1790.17
Z	4
Density (calculated, mg/m^3)	1.248
μ (mm^{-1})	0.573
F(000)	712.0
Crystal size (mm^3)	0.3 × 0.2 × 0.2
2Θ range for data collection/ °	8.24 to 124.464
Index ranges	$-12 \leq h \leq 12$, $-10 \leq k \leq 10$, $-20 \leq l \leq 20$
Reflections collected	21892
Independent reflections	2798 [R_{int} = 0.0472, R_{sigma} = 0.0224]
Completeness	100%
Absorption correction	multi-scan
Refinement method	Full-matrix least-squares on F^2
Data / restraints / parameters	2798/0/236
Goodness-of-fit on F^2	1.032

Final R indices [I>=2σ (I)]	R_1 = 0.0364,<br>wR_2 = 0.0952
R indices (all data)	R_1 = 0.0394,<br>wR_2 = 0.0991
Largest diff. peak/hole (e.Å^{-3})	0.16/-0.16

6. Computational Studies

6.1 General Information

DFT calculations were performed with Gaussian 09. [9] Optimization of the geometries of key intermediates were conducted at M06-2X/def2-TZVP level of theory [10, 11] and solvation (dichloromethane) with SMD model. [12] All calculations are included semi-empirical dispersion correction (DFT-D3). [13] Hirshfeld charges were analyzed in Multiwfn. [14]

6.2 Hirshfeld Charge Analysis

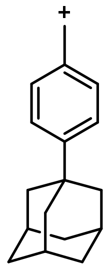

Int1

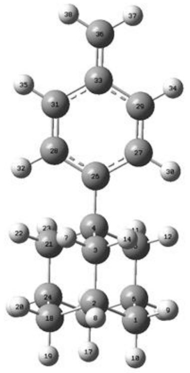

Atom		Hirshfeld charges (a.u.)
Atom	1(C):	-0.05241841
Atom	2(C):	-0.01445658
Atom	3(C):	-0.04045956
Atom	4(C):	0.01908687
Atom	5(C):	-0.04747637
Atom	6(C):	-0.01630316
Atom	7(H):	0.03525841
Atom	8(H):	0.03681989
Atom	9(H):	0.03039333
Atom	10(H):	0.03243369
Atom	11(H):	0.03494354
Atom	12(H):	0.03696399
Atom	13(H):	0.03495487
Atom	14(H):	0.03525850
Atom	15(C):	-0.05378989
Atom	16(H):	0.03016640
Atom	17(H):	0.03170680
Atom	18(C):	-0.05241834
Atom	19(H):	0.03243370
Atom	20(H):	0.03039341

Atom	21(C):	-0.04747653
Atom	22(H):	0.03696425
Atom	23(H):	0.03494377
Atom	24(C):	-0.01630304
Atom	25(H):	0.03495478
Atom	26(C):	0.10527252
Atom	27(C):	-0.01590009
Atom	28(C):	-0.01590037
Atom	29(C):	0.04409792
Atom	30(H):	0.06911611
Atom	31(C):	0.04409826
Atom	32(H):	0.06911581
Atom	33(C):	0.01240613
Atom	34(H):	0.08631337
Atom	35(H):	0.08631347
Atom	36(C):	0.12308173
Atom	37(H):	0.10270518
Atom	38(H):	0.10270564

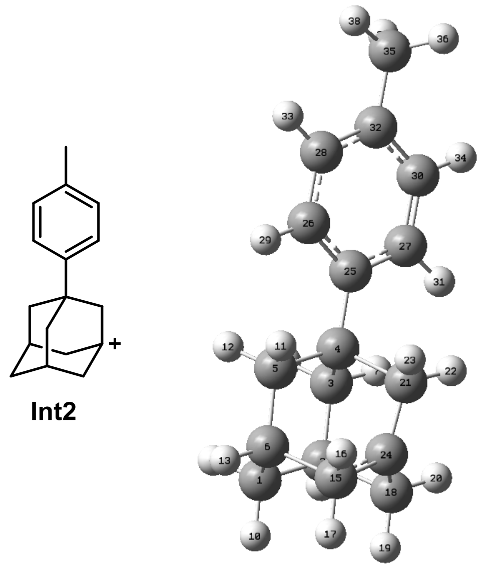

Atom		Hirshfeld charges (a.u.)
Atom	1(C):	-0.04236664
Atom	2(C):	0.02210931
Atom	3(C):	-0.04440973
Atom	4(C):	0.04652892
Atom	5(C):	-0.04312083
Atom	6(C):	0.02317372
Atom	7(H):	0.04771333

Atom	8(H):	0.06552631
Atom	9(H):	0.05699887
Atom	10(H):	0.05078392
Atom	11(H):	0.04955060
Atom	12(H):	0.05570427
Atom	13(H):	0.06601865
Atom	14(H):	0.05454294
Atom	15(C):	-0.02178332
Atom	16(H):	0.07305742
Atom	17(H):	0.07236105
Atom	18(C):	-0.02212643
Atom	19(H):	0.07212646
Atom	20(H):	0.07255407
Atom	21(C):	-0.02716023
Atom	22(H):	0.06715372
Atom	23(H):	0.06849306
Atom	24(C):	0.18245679
Atom	25(C):	-0.00747487
Atom	26(C):	-0.04689384
Atom	27(C):	-0.04373245
Atom	28(C):	-0.05091855
Atom	29(H):	0.04933962
Atom	30(C):	-0.04803735
Atom	31(H):	0.05157090
Atom	32(C):	0.00267212
Atom	33(H):	0.04955513
Atom	34(H):	0.05126095
Atom	35(C):	-0.08303650
Atom	36(H):	0.04344916
Atom	37(H):	0.04378190
Atom	38(H):	0.04257756

Remarks: Hirshfeld charge analysis revealed that the positive charge density of the benzyl carbocation (**Int1**) was lower than that of the adamantly carbocation (**Int2**) (0.123 to 0.182 a.u.).

7. ^{1}H and ^{13}C NMR Spectra

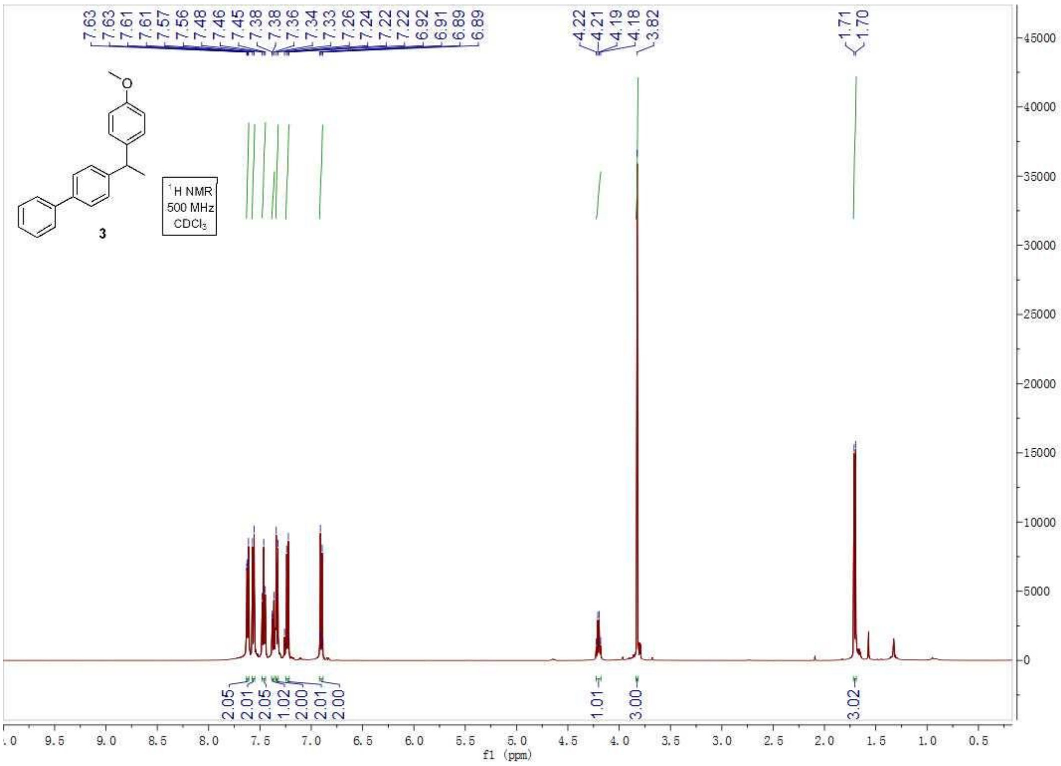

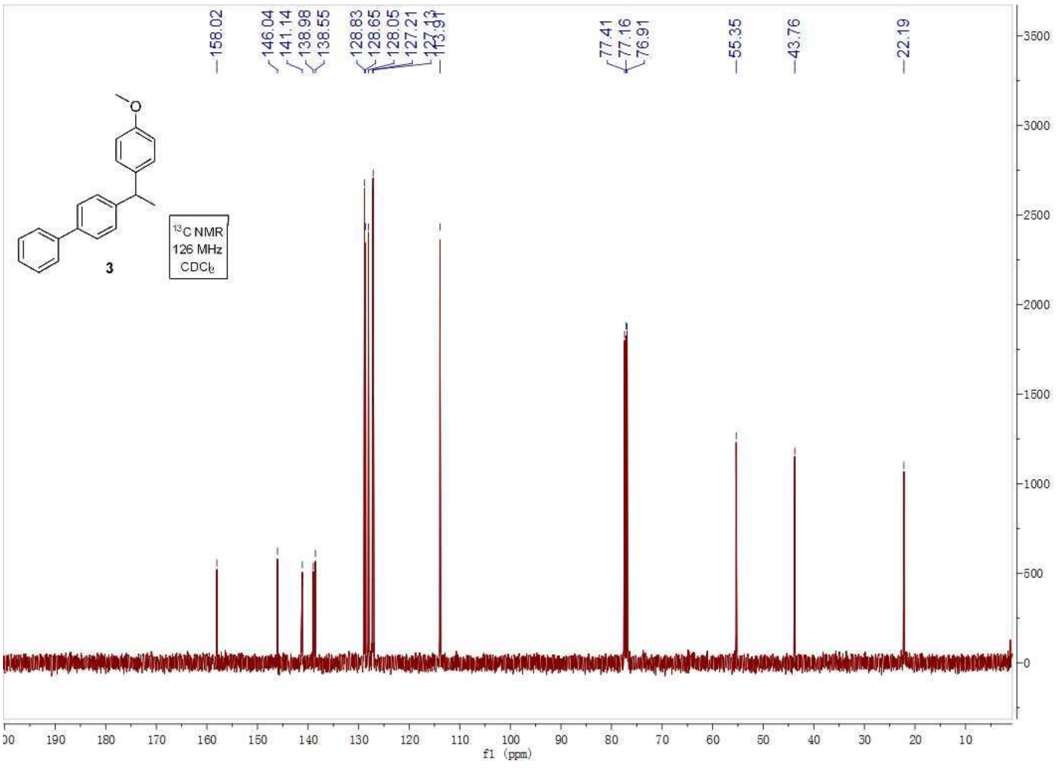

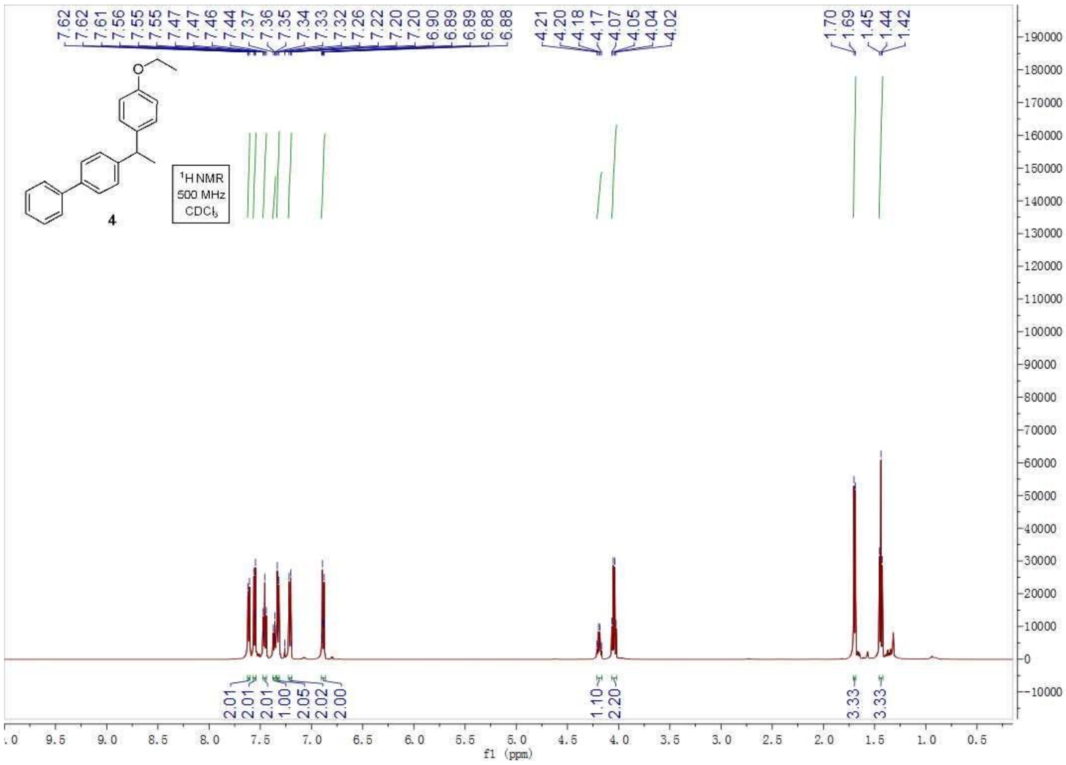

7.62
7.62
7.61
7.56
7.55
7.55
7.47
7.47
7.46
7.44
7.37
7.36
7.35
7.34
7.33
7.32
7.26
7.22
7.20
6.90
6.89
6.89
6.88
6.88
4.21
4.20
4.18
4.17
4.07
4.05
4.04
4.02
1.70
1.69
1.45
1.44
1.42
^{1}H NMR
500 MHz
$CDCl_3$
4
2.01
2.01
2.01
1.00
2.05
2.02
2.00
1.10
2.20
3.33
3.33
f1 (ppm)

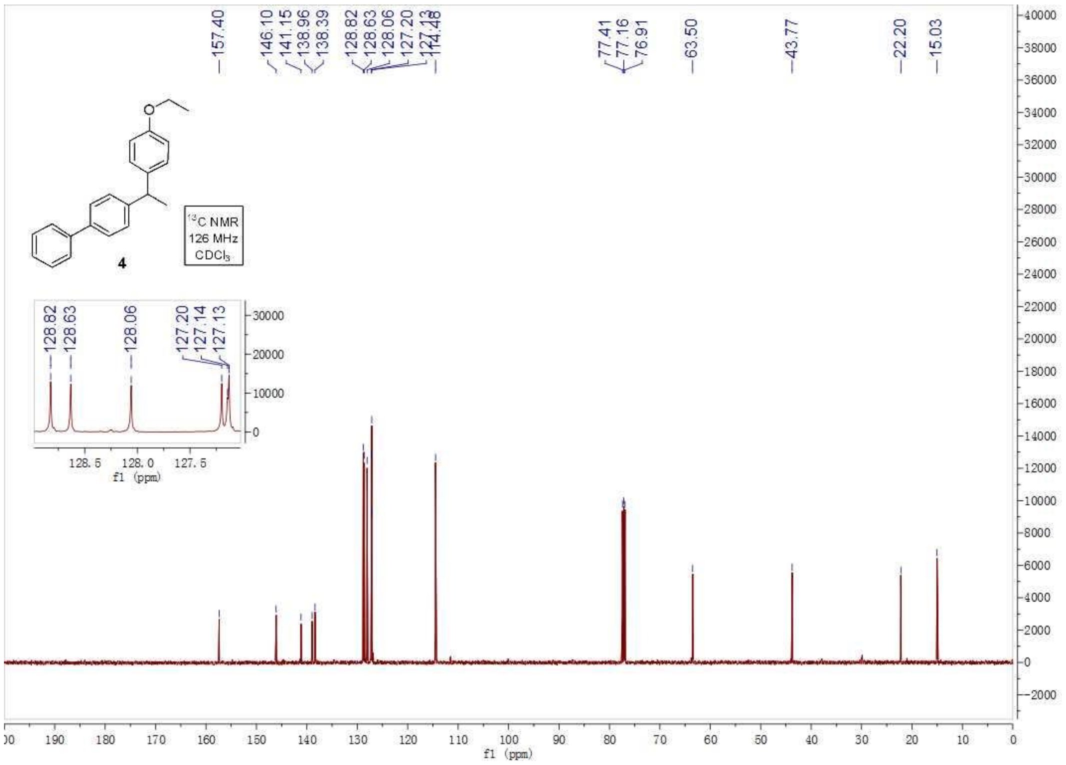

157.40
146.10
141.15
138.96
138.39
128.82
128.63
128.06
127.20
127.13
114.48
77.41
77.16
76.91
63.50
43.77
22.20
15.03
^{13}C NMR
126 MHz
$CDCl_3$
4
128.82
128.63
128.06
127.20
127.14
127.13
f1 (ppm)

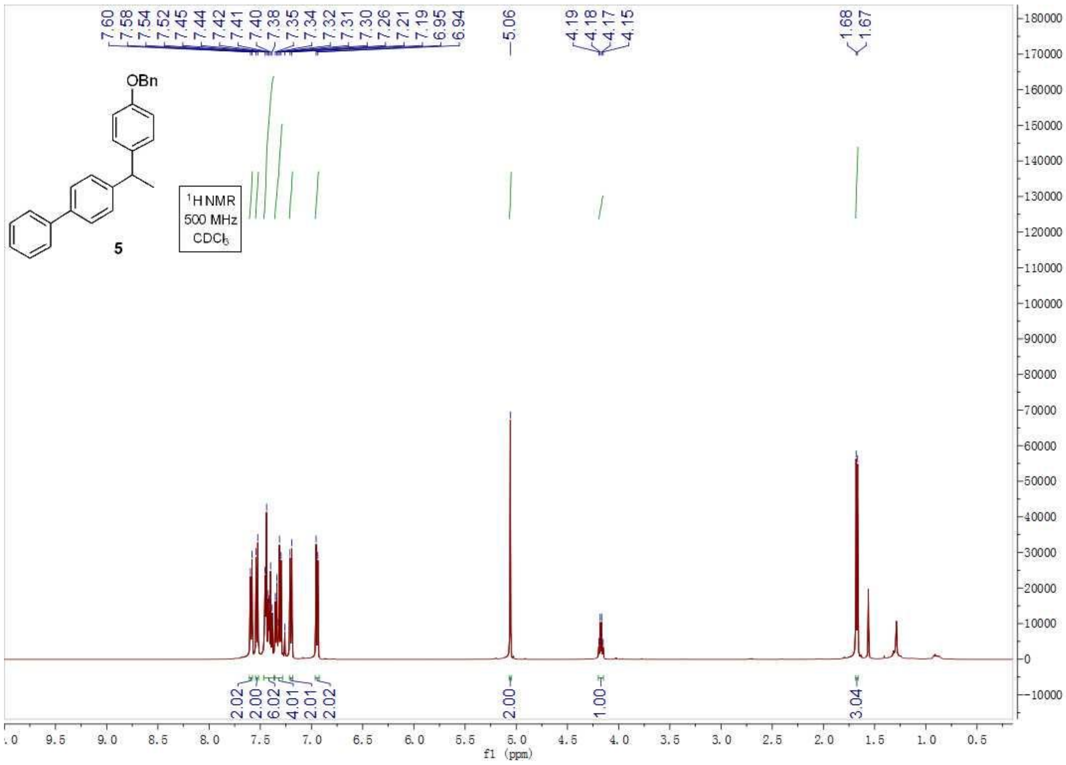

OBn
5
^{1}H NMR
500 MHz
$CDCl_3$
f1 (ppm)

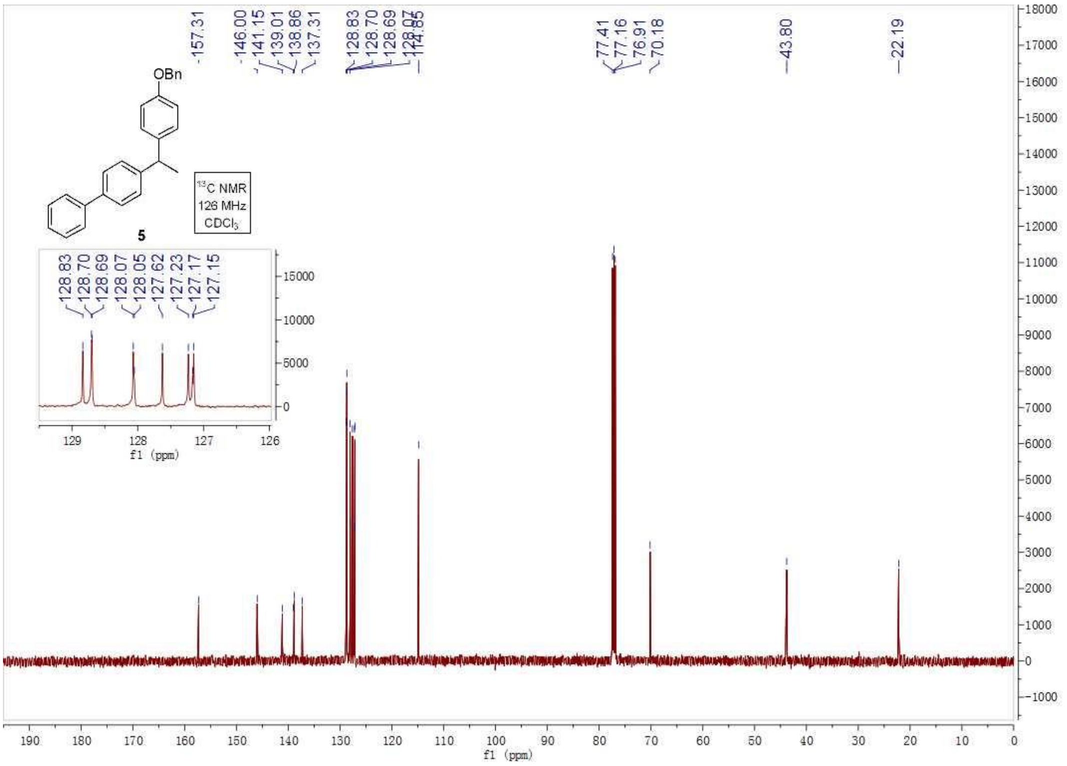

OBn
5
^{13}C NMR
126 MHz
$CDCl_3$
f1 (ppm)

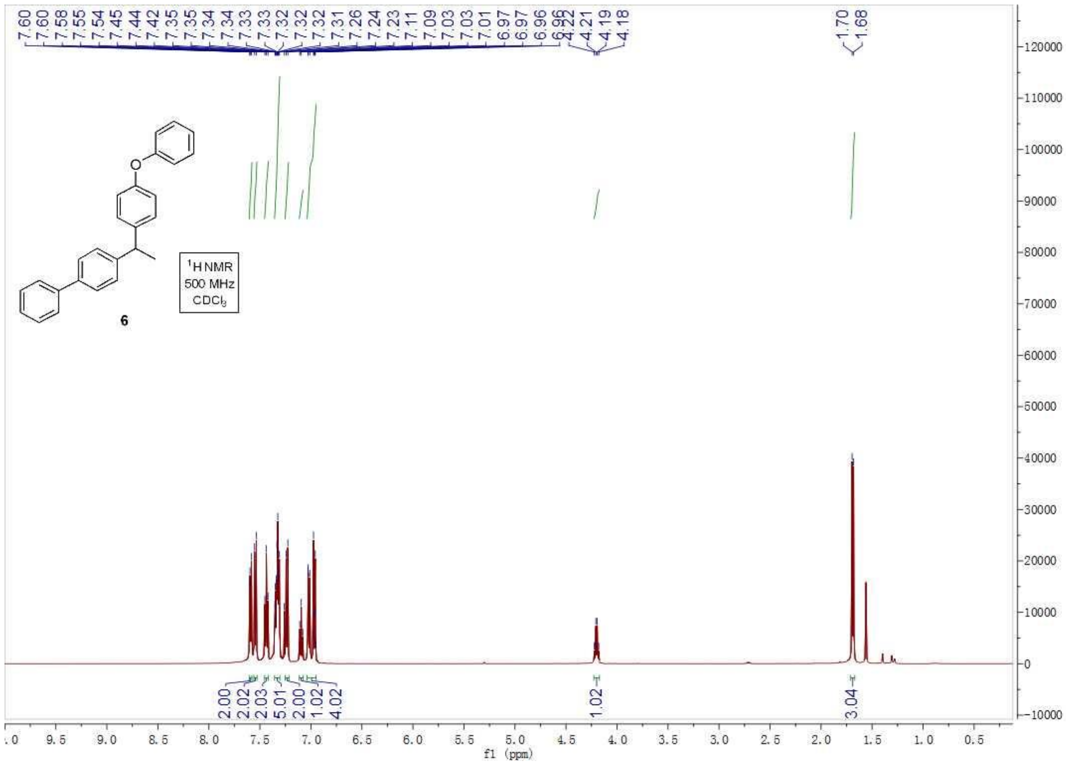

7.60 7.60 7.58 7.55 7.54 7.45 7.44 7.42 7.35 7.35 7.34 7.34 7.33 7.33 7.32 7.32 7.32 7.31 7.26 7.24 7.23 7.11 7.09 7.03 7.03 7.01 6.97 6.97 6.96 6.96 4.22 4.21 4.19 4.18
1.70 1.68
6
1H NMR
500 MHz
CDCl3
2.00 2.02 2.03 5.01 2.00 1.02 4.02
1.02
3.04
f1 (ppm)

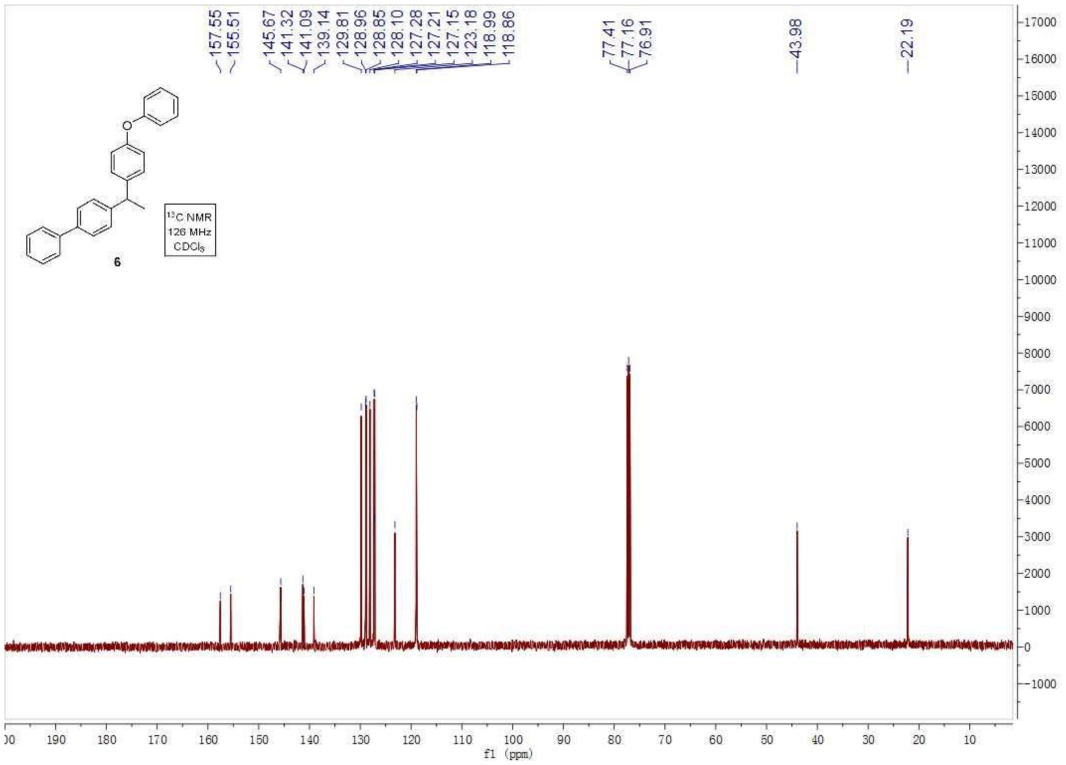

157.55 155.51 145.67 141.32 141.09 139.14 129.81 128.96 128.85 128.10 127.28 127.21 127.15 123.18 118.99 118.86
77.41 77.16 76.91
43.98
22.19
6
13C NMR
126 MHz
CDCl3
f1 (ppm)

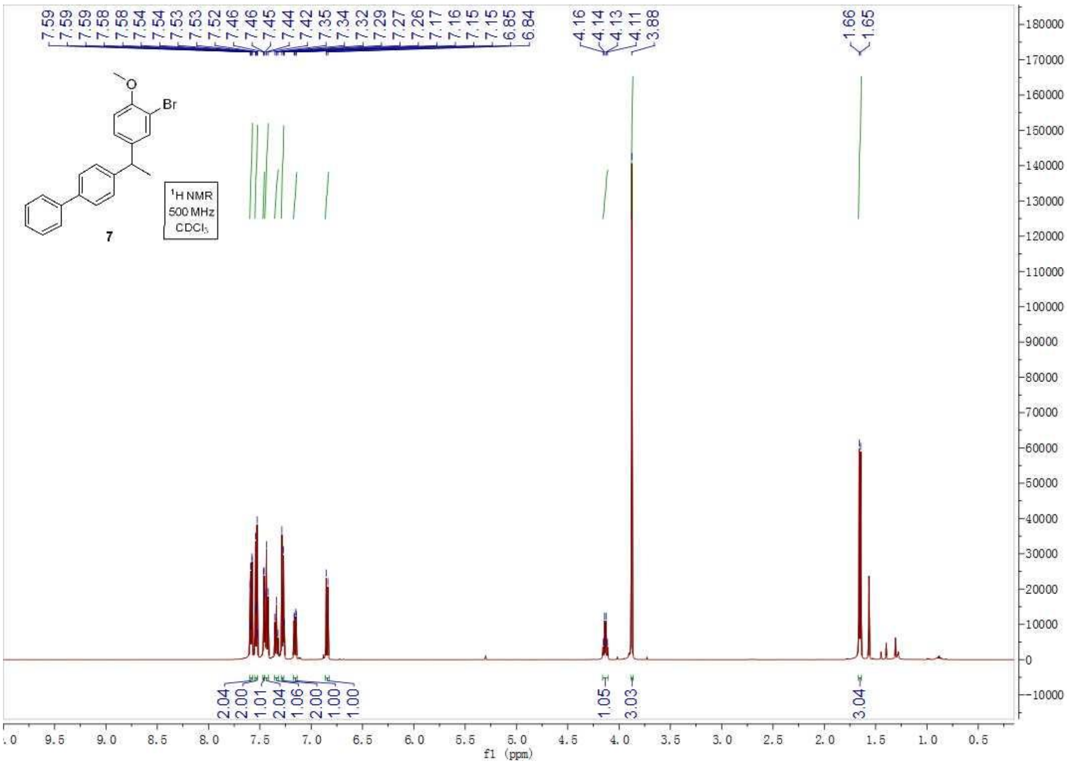

Br
¹H NMR
500 MHz
CDCl3
7

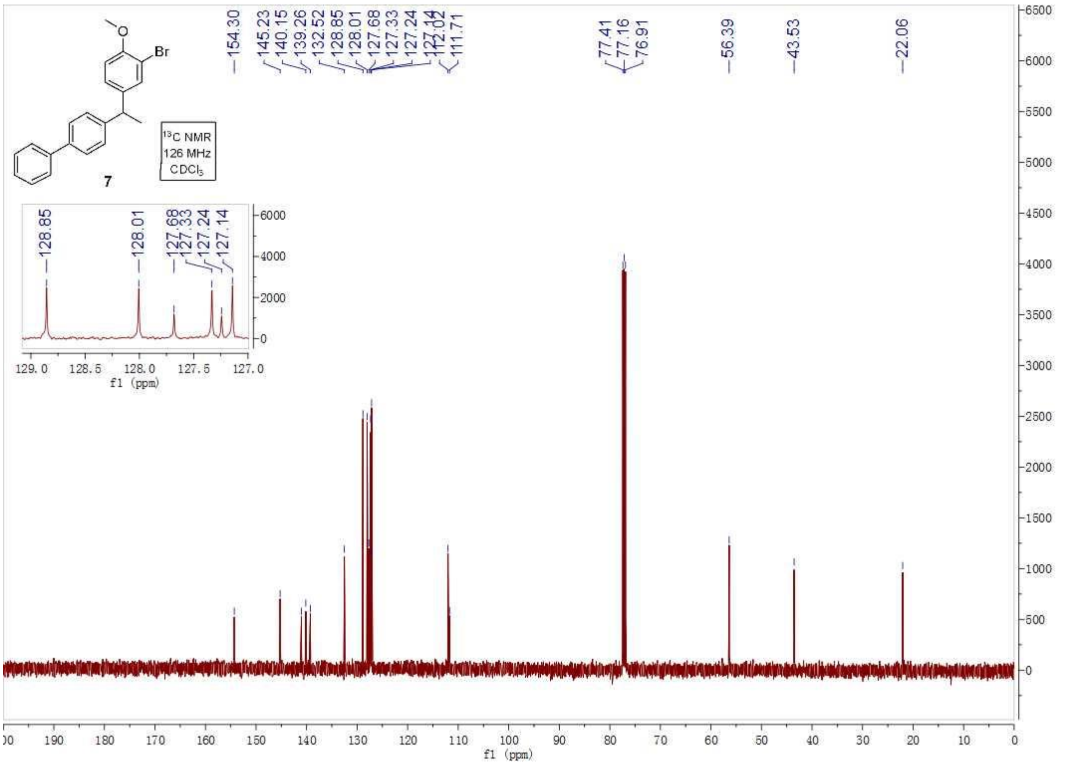

Br
¹³C NMR
126 MHz
CDCl3
7

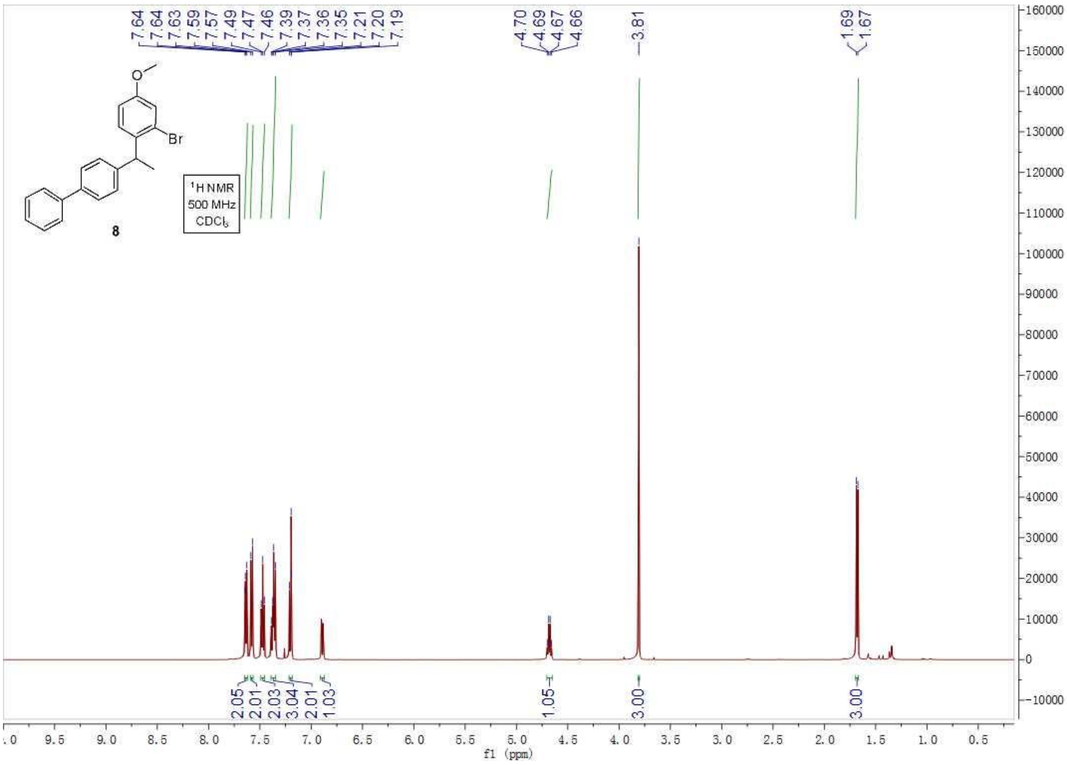

7.64
7.64
7.63
7.59
7.57
7.49
7.47
7.46
7.39
7.37
7.36
7.35
7.21
7.20
7.19
4.70
4.69
4.67
4.66
3.81
1.69
1.67
Br
^{1}H NMR
500 MHz
CDCl$_3$
8
2.05
2.01
2.03
3.04
2.01
1.03
1.05
3.00
3.00
f1 (ppm)

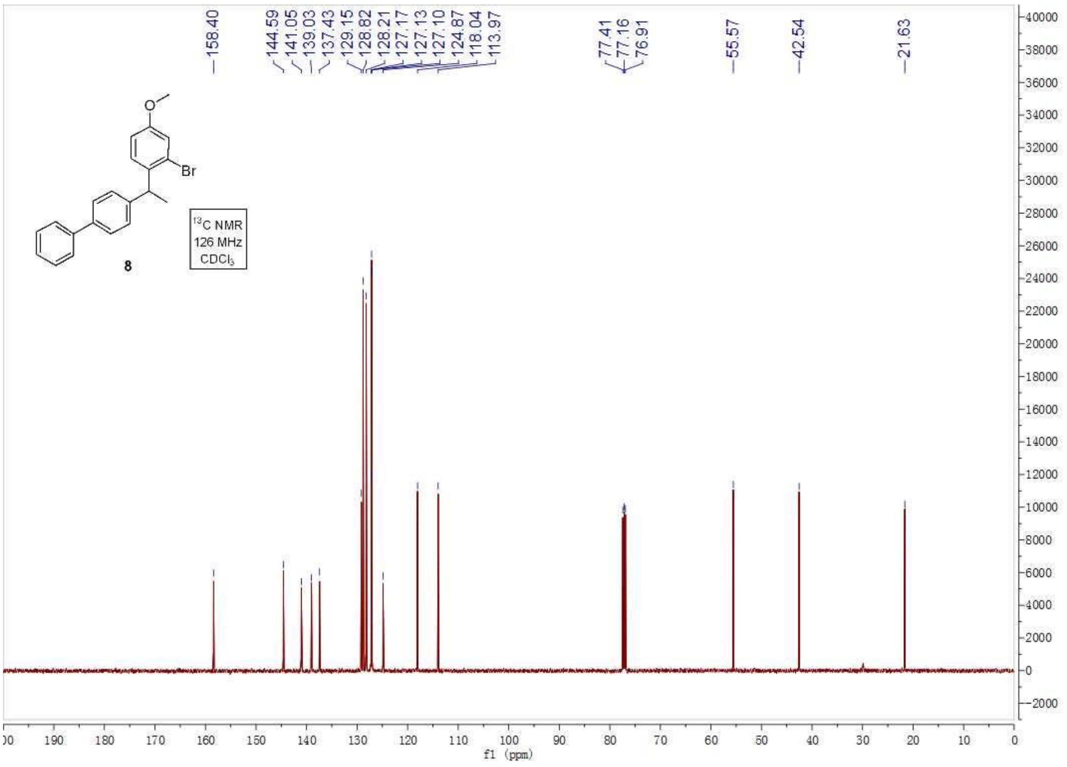

158.40
144.59
141.05
139.03
137.43
129.15
128.82
128.21
127.17
127.13
127.10
124.87
118.04
113.97
77.41
77.16
76.91
55.57
42.54
21.63
Br
^{13}C NMR
126 MHz
CDCl$_3$
8
f1 (ppm)

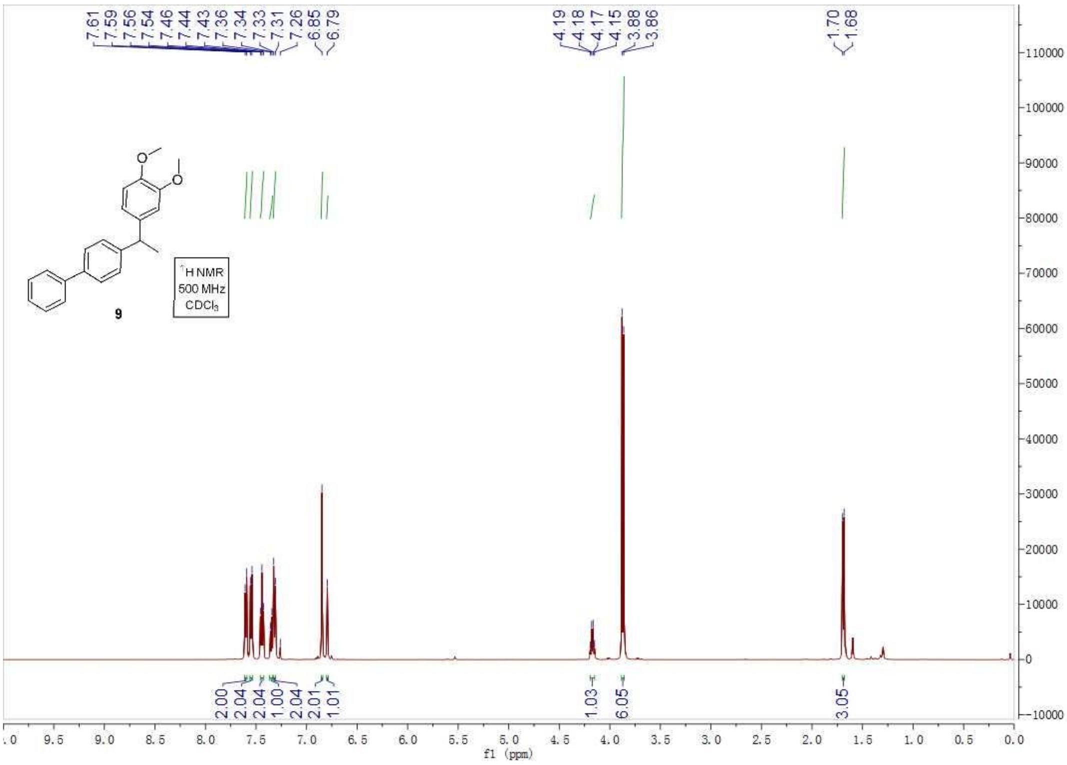
7.61
7.59
7.56
7.54
7.46
7.44
7.43
7.36
7.34
7.33
7.31
7.26
6.85
6.79
4.19
4.18
4.17
4.15
3.88
3.86
1.70
1.68
9
^{1}H NMR
500 MHz
$CDCl_3$
2.00
2.04
2.04
1.00
2.04
2.01
1.01
1.03
6.05
3.05
f1 (ppm)

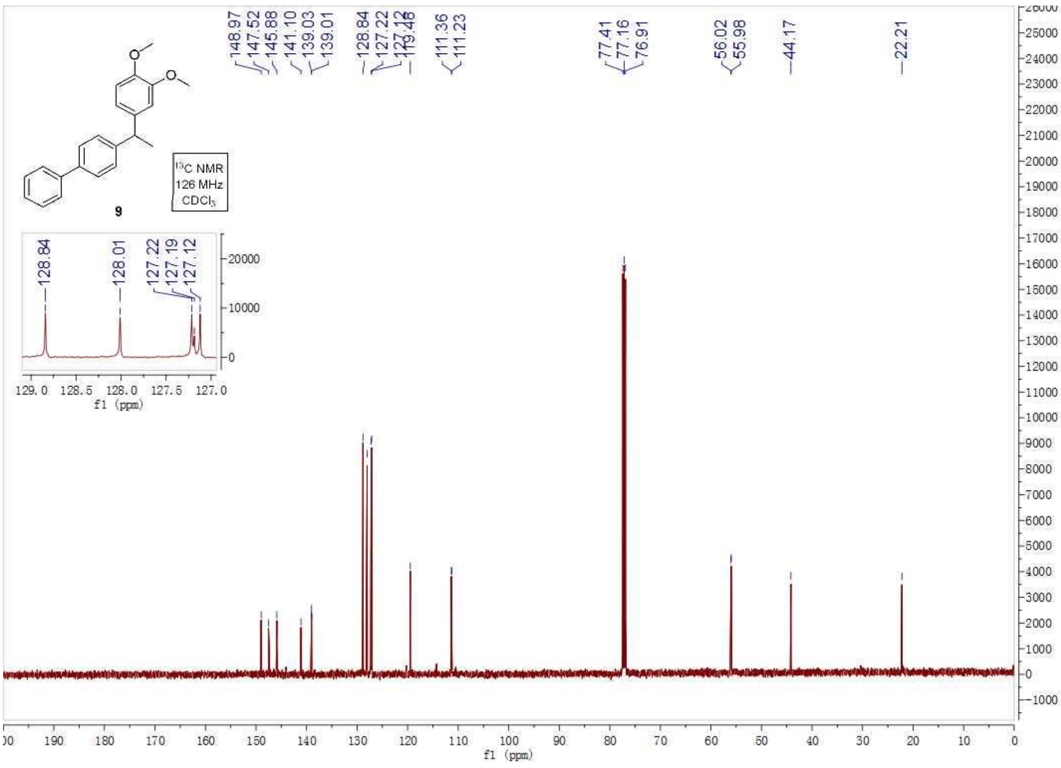
148.97
147.52
145.88
141.10
139.03
139.01
128.84
127.22
111.36
111.23
77.41
77.16
76.91
56.02
55.98
44.17
22.21
9
^{13}C NMR
126 MHz
$CDCl_3$
128.84
128.01
127.22
127.19
127.12
f1 (ppm)

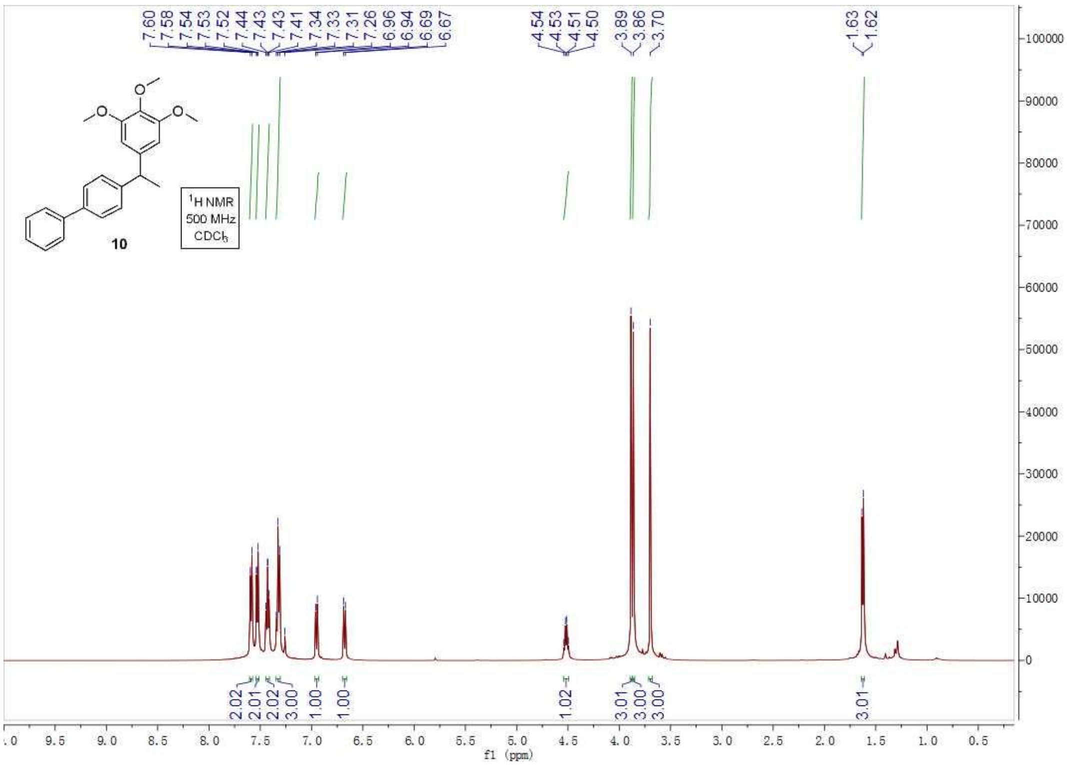

7.60
7.58
7.54
7.53
7.52
7.44
7.43
7.43
7.41
7.34
7.33
7.31
7.26
6.96
6.94
6.69
6.67
4.54
4.53
4.51
4.50
3.89
3.86
3.70
1.63
1.62
^{1}H NMR
500 MHz
$CDCl_3$
10
2.02
2.01
2.02
3.00
1.00
1.00
1.02
3.01
3.00
3.00
3.01
f1 (ppm)

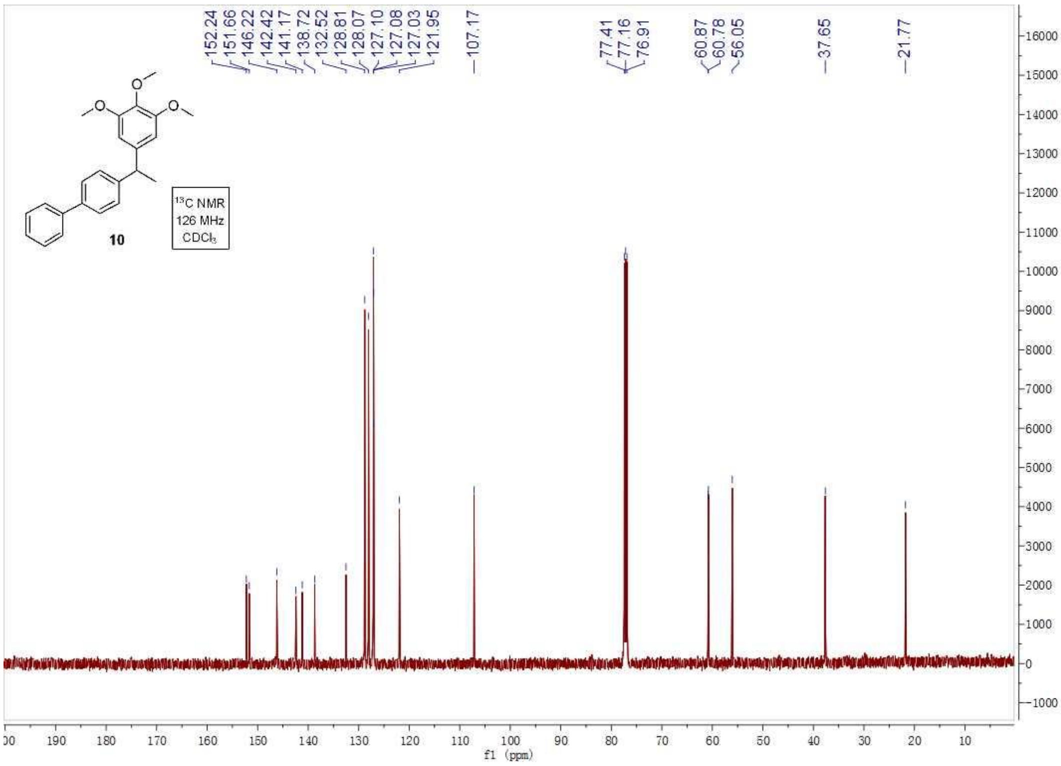

152.24
151.66
146.22
142.42
141.17
138.72
132.52
128.81
128.07
127.10
127.08
127.03
121.95
107.17
77.41
77.16
76.91
60.87
60.78
56.05
37.65
21.77
^{13}C NMR
126 MHz
$CDCl_3$
10
f1 (ppm)

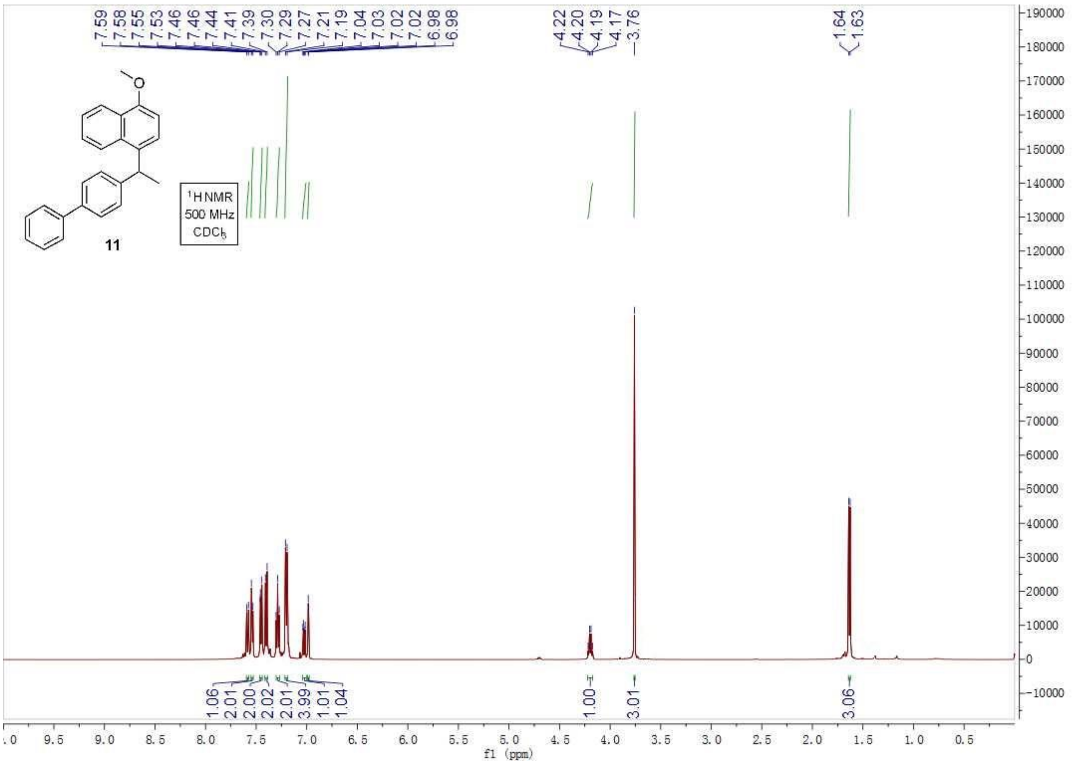

7.59 7.58 7.55 7.53 7.46 7.46 7.44 7.41 7.39 7.30 7.29 7.27 7.21 7.19 7.04 7.03 7.02 7.02 6.98 6.98
4.22 4.20 4.19 4.17 3.76
1.64 1.63
11
^{1}H NMR
500 MHz
$CDCl_3$
1.06 2.01 2.00 2.02 2.01 3.99 1.01 1.04
1.00 3.01
3.06
f1 (ppm)

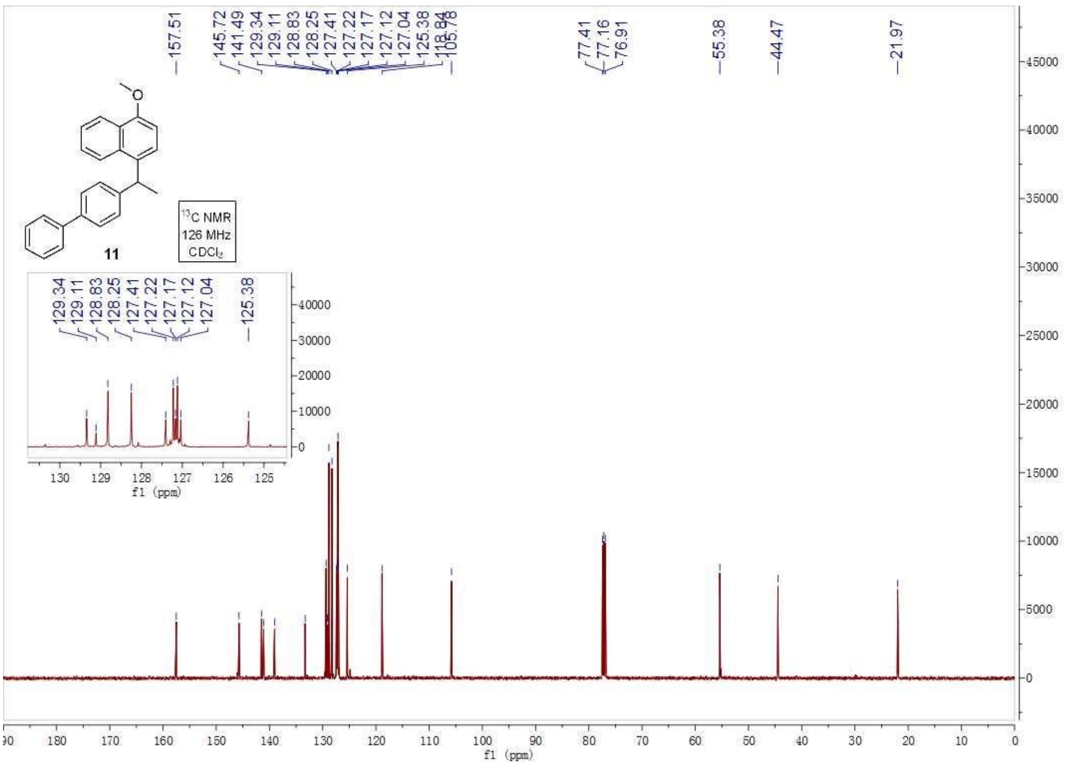

157.51
145.72 141.49 129.34 129.11 128.83 128.25 127.41 127.22 127.17 127.12 127.04 125.38
105.78
77.41 77.16 76.91
55.38
44.47
21.97
11
^{13}C NMR
126 MHz
$CDCl_3$
f1 (ppm)

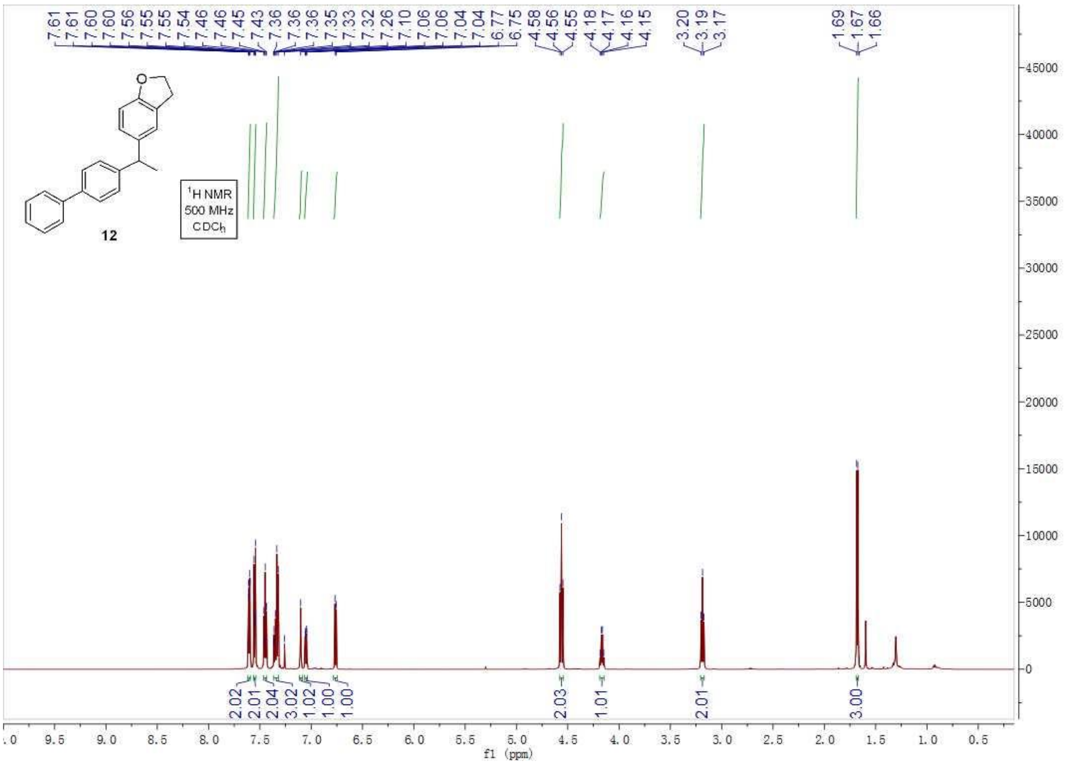

12
^{1}H NMR
500 MHz
CDCl$_3$
f1 (ppm)

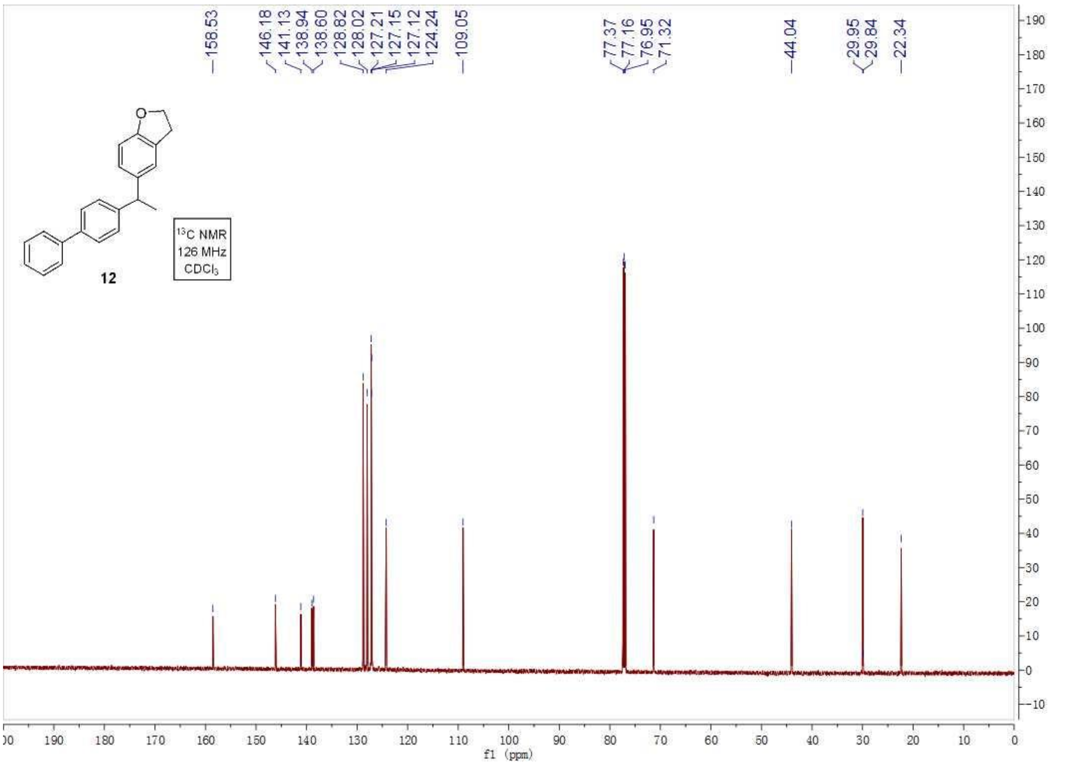

12
^{13}C NMR
126 MHz
CDCl$_3$
f1 (ppm)

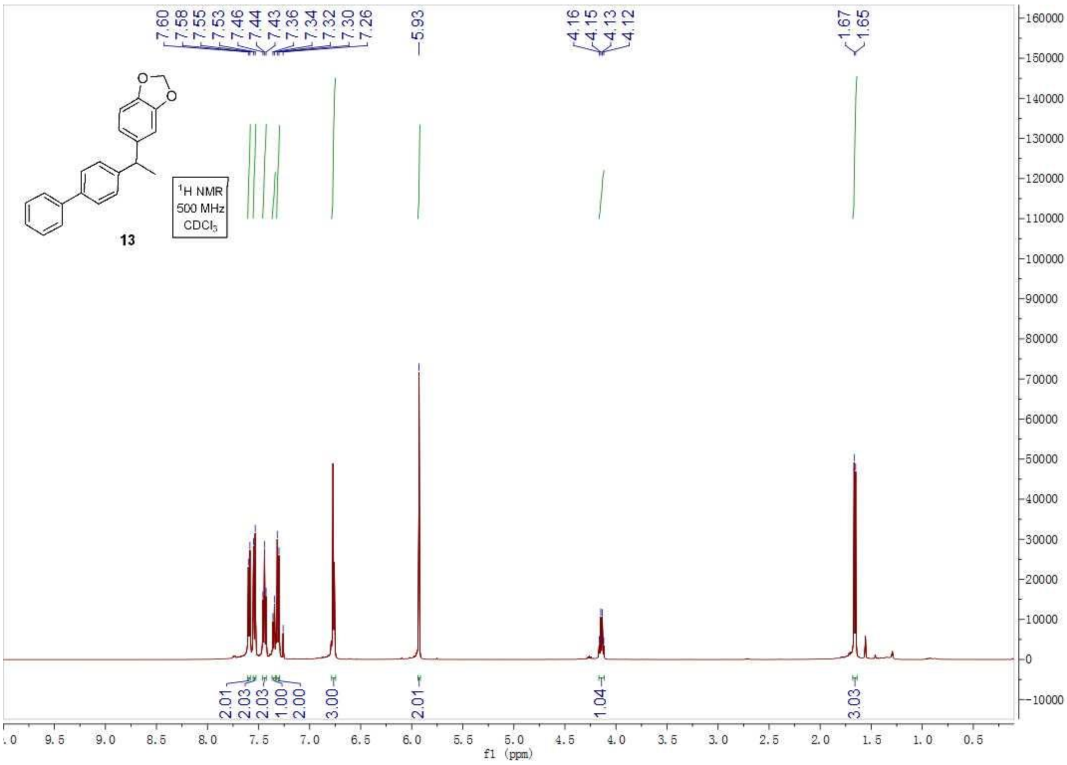

7.60
7.58
7.55
7.53
7.46
7.44
7.43
7.36
7.34
7.32
7.30
7.26
5.93
4.16
4.15
4.13
4.12
1.67
1.65
^{1}H NMR
500 MHz
CDCl3
13
2.01
2.03
2.03
1.00
2.00
3.00
2.01
1.04
3.03
f1 (ppm)

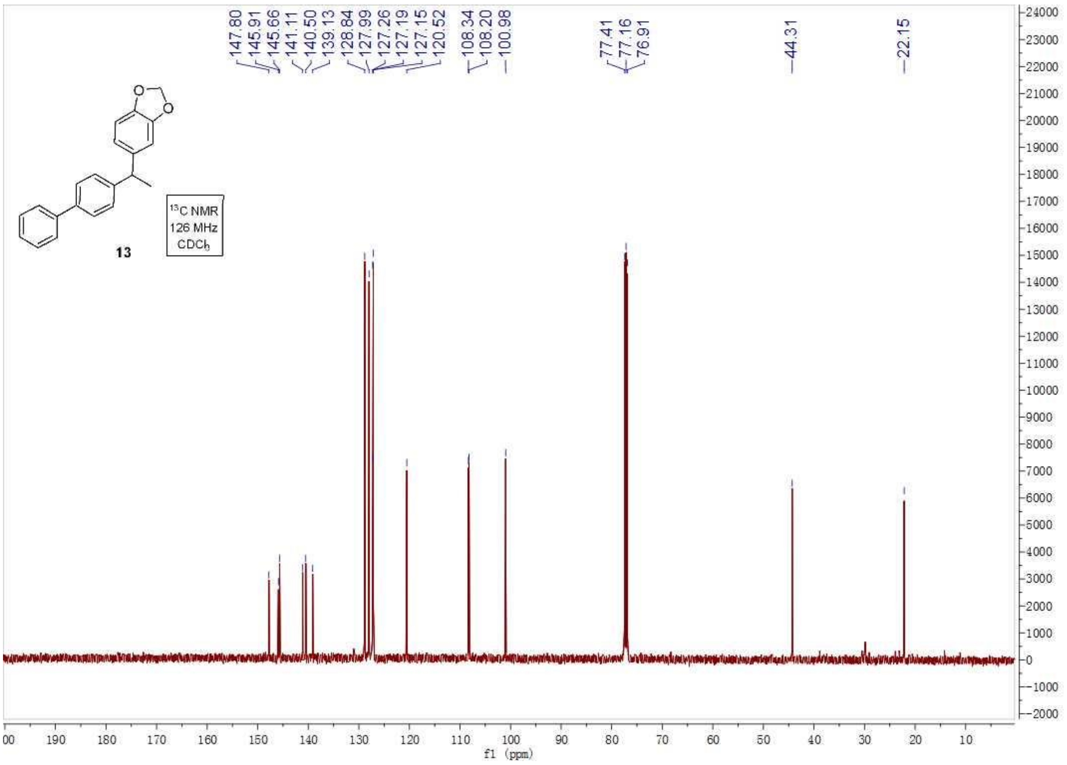

147.80
145.91
145.66
141.11
140.50
139.13
128.84
127.99
127.26
127.19
127.15
120.52
108.34
108.20
100.98
77.41
77.16
76.91
44.31
22.15
^{13}C NMR
126 MHz
CDCl3
13
f1 (ppm)

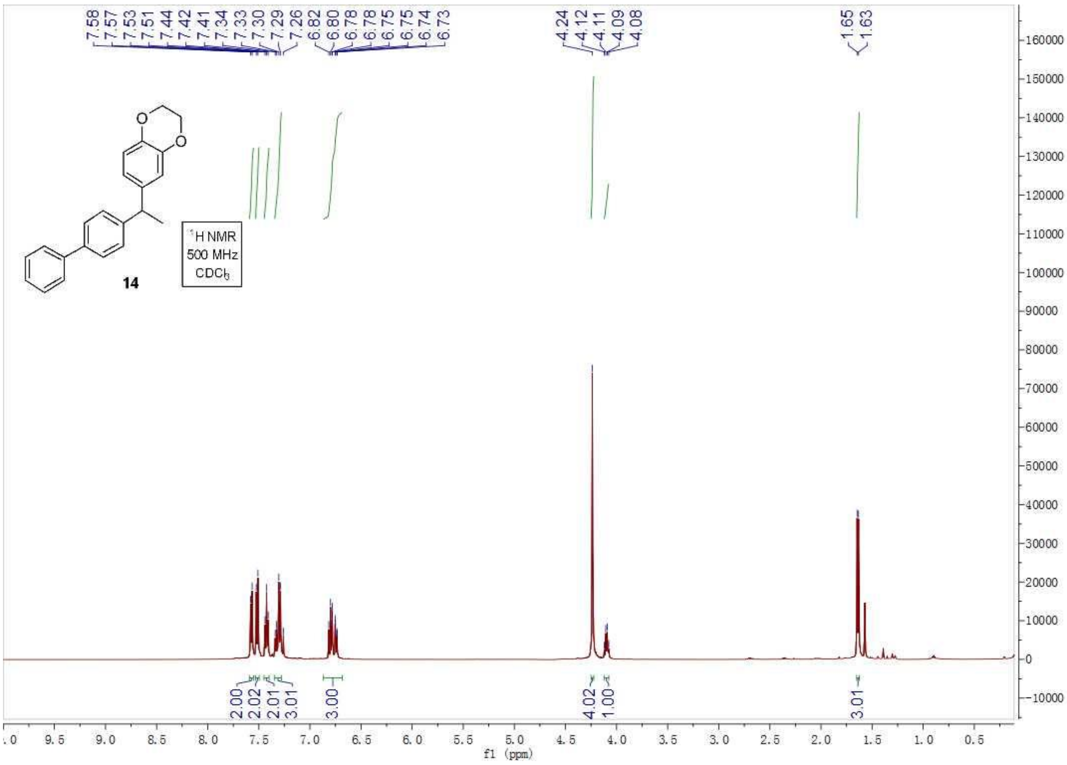
7.58 7.57 7.53 7.51 7.44 7.42 7.41 7.34 7.33 7.30 7.29 7.26 6.82 6.80 6.78 6.78 6.75 6.75 6.74 6.73
4.24 4.12 4.11 4.09 4.08
1.65 1.63
1H NMR
500 MHz
CDCl3
14
2.00 2.02 2.01 3.01 3.00 4.02 1.00 3.01
f1 (ppm)

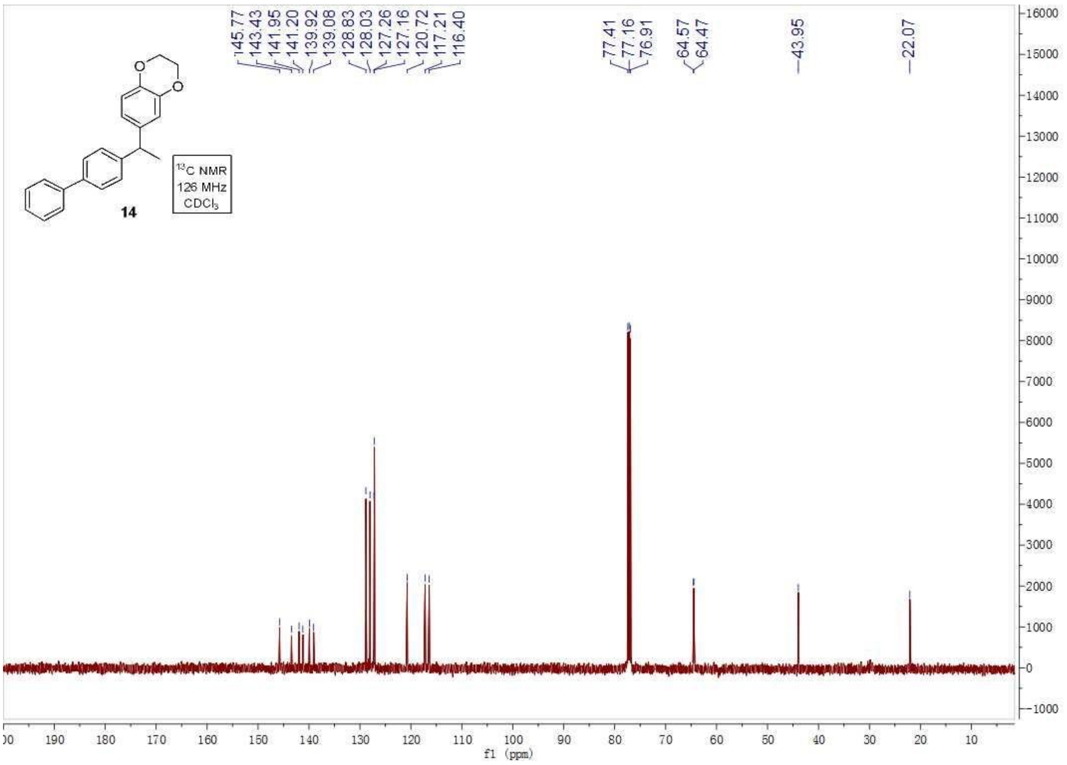
145.77 143.43 141.95 141.20 139.92 139.08 128.83 128.03 127.26 127.16 120.72 117.21 116.40
77.41 77.16 76.91 64.57 64.47
43.95
22.07
13C NMR
126 MHz
CDCl3
14
f1 (ppm)

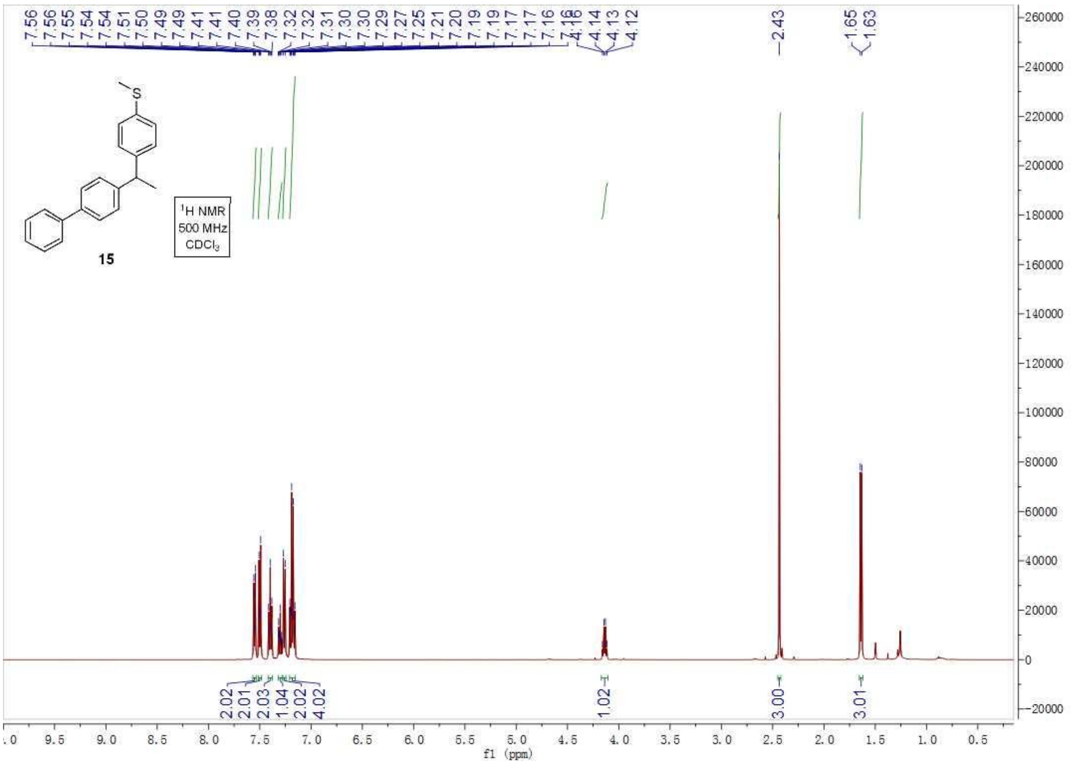

1H NMR
500 MHz
CDCl3
15
f1 (ppm)

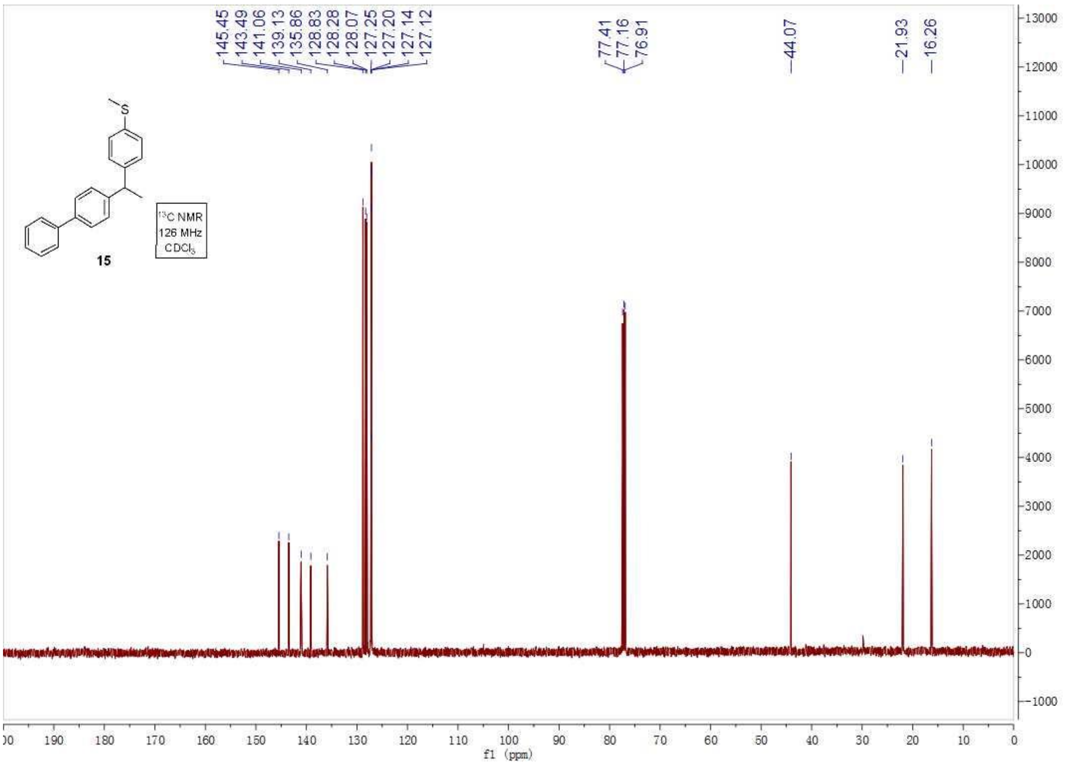

13C NMR
126 MHz
CDCl3
15
145.45
143.49
141.06
139.13
135.86
128.83
128.28
128.07
127.25
127.20
127.14
127.12
77.41
77.16
76.91
44.07
21.93
16.26
f1 (ppm)

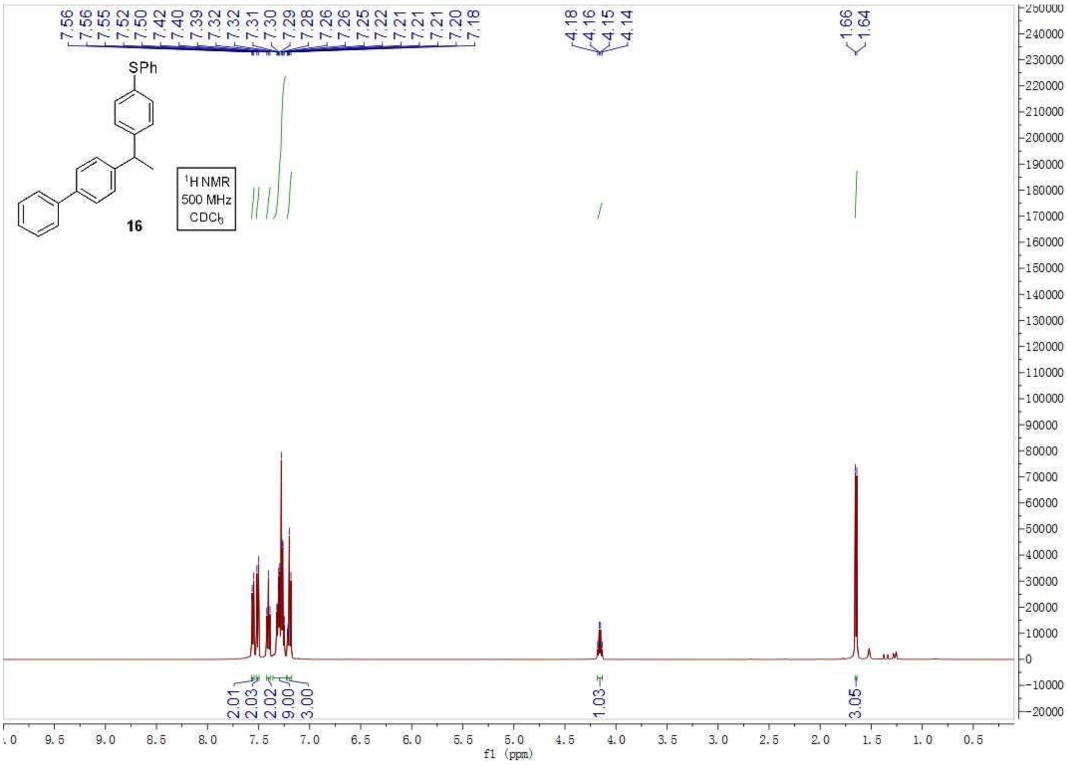
7.56
7.56
7.55
7.52
7.50
7.42
7.40
7.39
7.32
7.31
7.30
7.29
7.28
7.26
7.26
7.25
7.22
7.21
7.21
7.21
7.20
7.18
4.18
4.16
4.15
4.14
1.66
1.64
SPh
16
1H NMR
500 MHz
CDCl3
2.01
2.03
2.02
9.00
3.00
1.03
3.05
f1 (ppm)

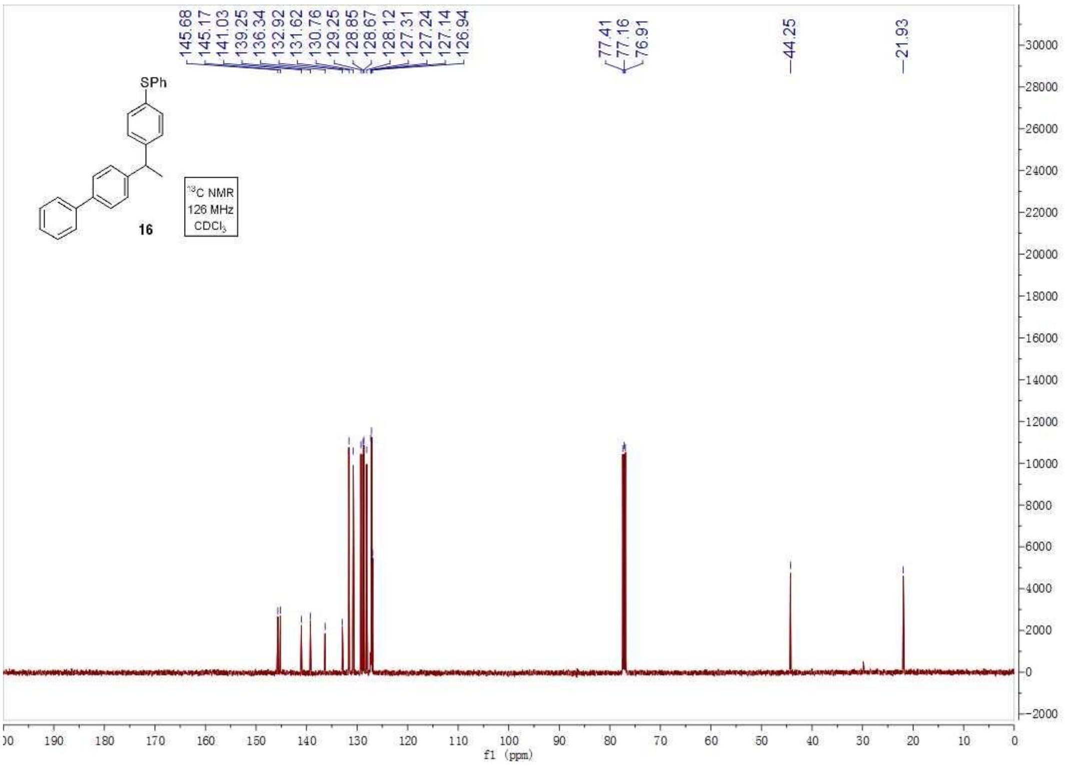
145.68
145.17
141.03
139.25
136.34
132.92
131.62
130.76
129.25
128.85
128.67
128.12
127.31
127.24
127.14
126.94
77.41
77.16
76.91
44.25
21.93
SPh
16
13C NMR
126 MHz
CDCl3
f1 (ppm)

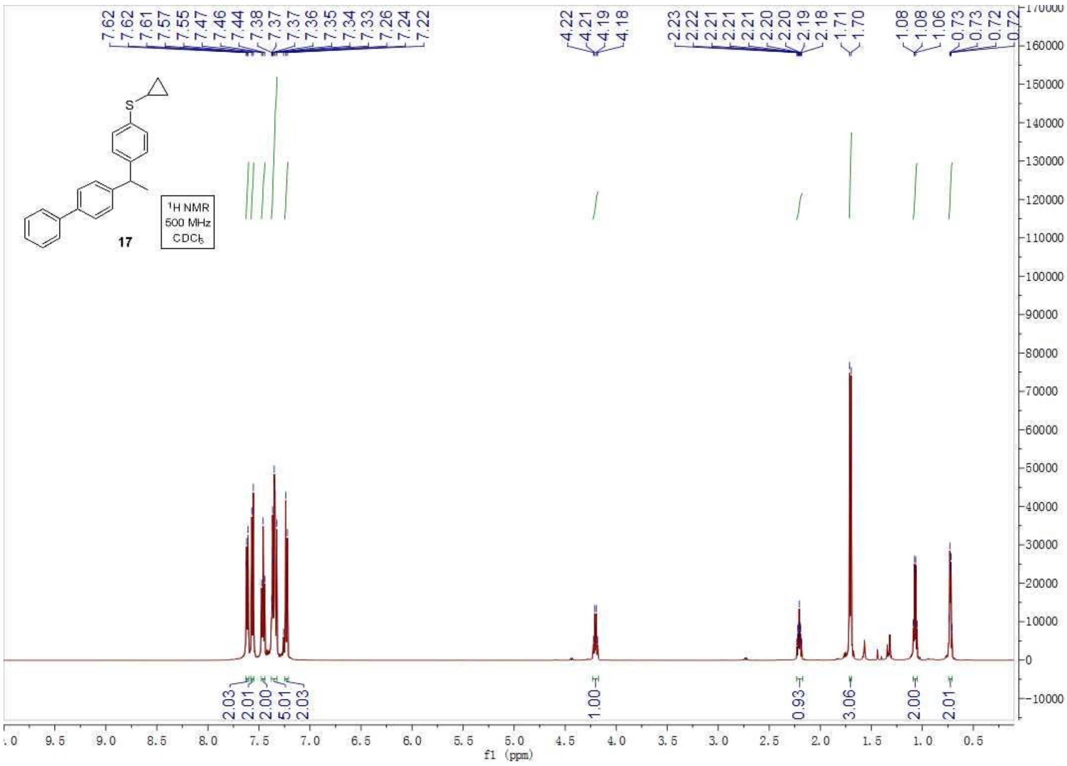

1H NMR
500 MHz
CDCl3
17
f1 (ppm)

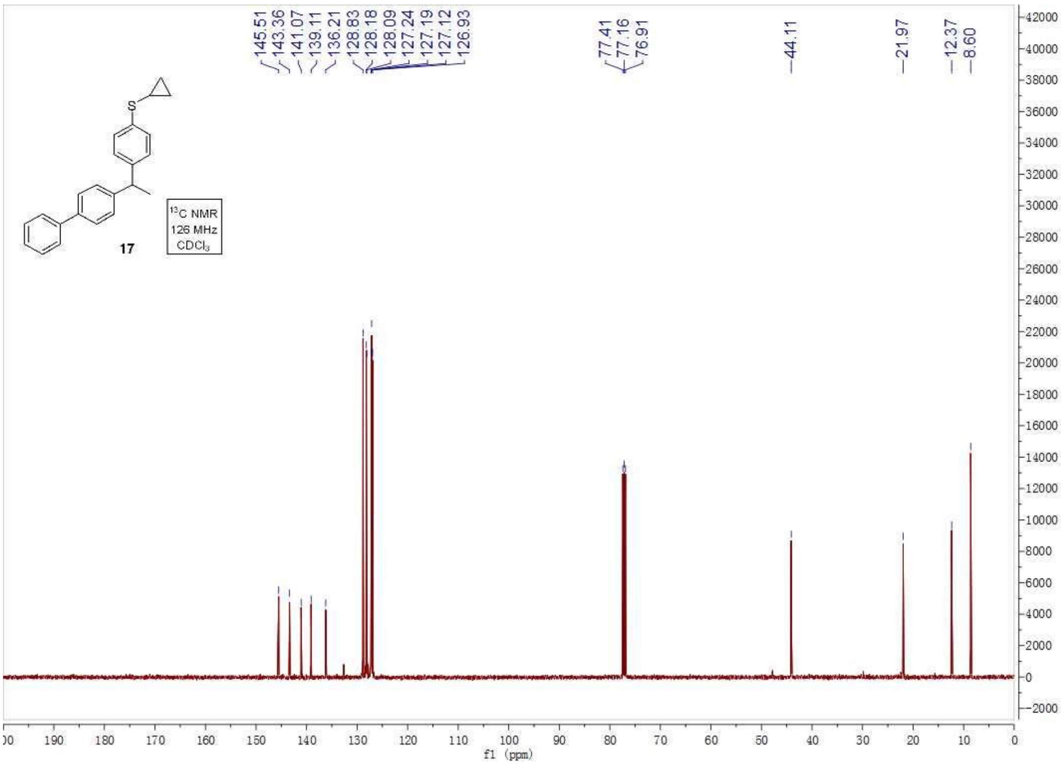

13C NMR
126 MHz
CDCl3
17
f1 (ppm)

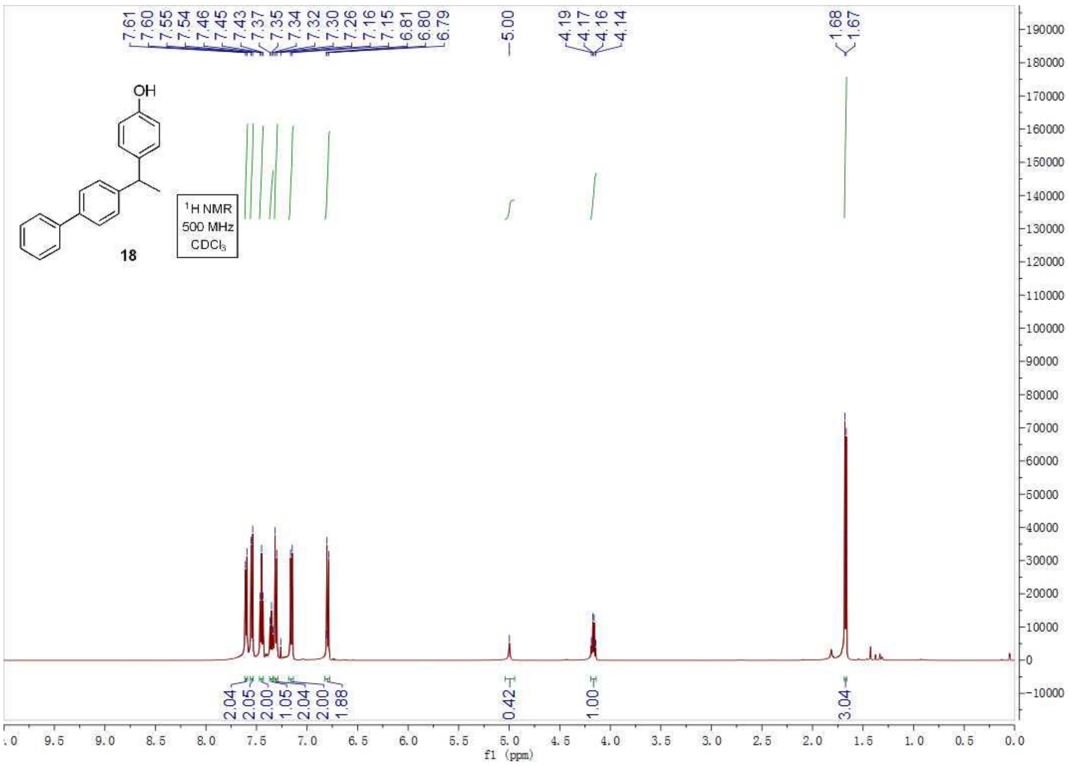
OH
18
^{1}H NMR
500 MHz
$CDCl_3$
7.61 7.60 7.55 7.54 7.46 7.45 7.43 7.37 7.35 7.34 7.32 7.30 7.26 7.16 7.15 6.81 6.80 6.79
5.00
4.19 4.17 4.16 4.14
1.68 1.67
2.04 2.05 2.00 1.05 2.04 2.00 1.88
0.42
1.00
3.04
f1 (ppm)

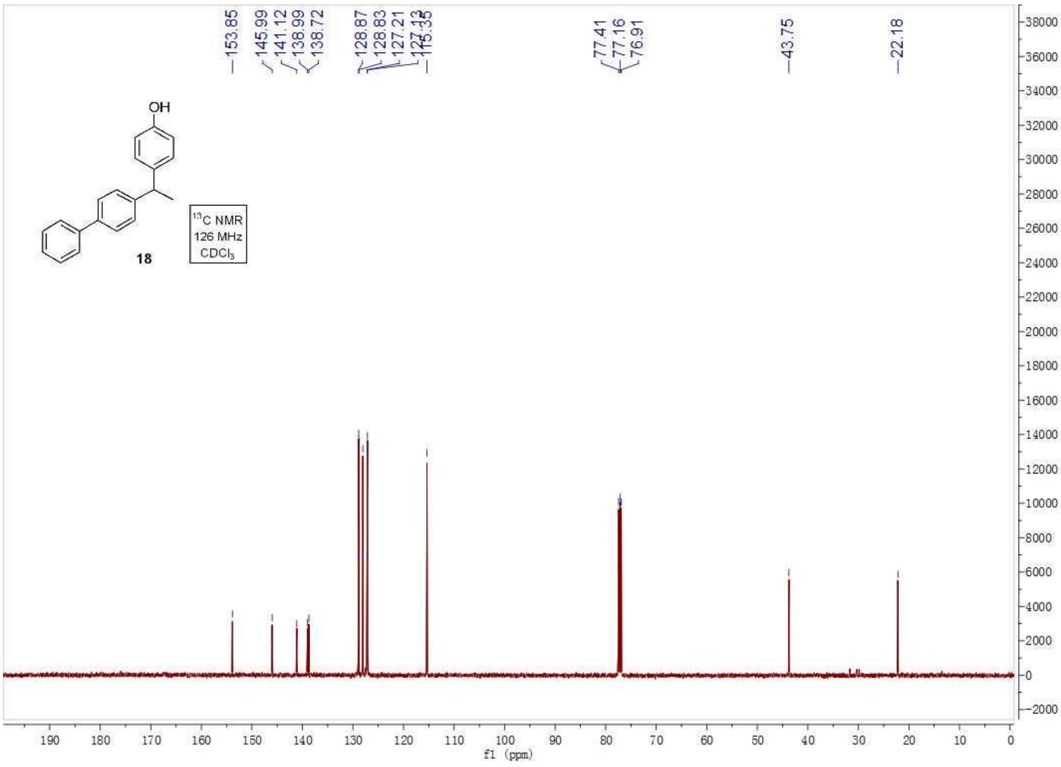
OH
18
^{13}C NMR
126 MHz
$CDCl_3$
153.85
145.99 141.12 138.99 138.72
128.87 128.83 127.21 127.13 115.35
77.41 77.16 76.91
43.75
22.18
f1 (ppm)

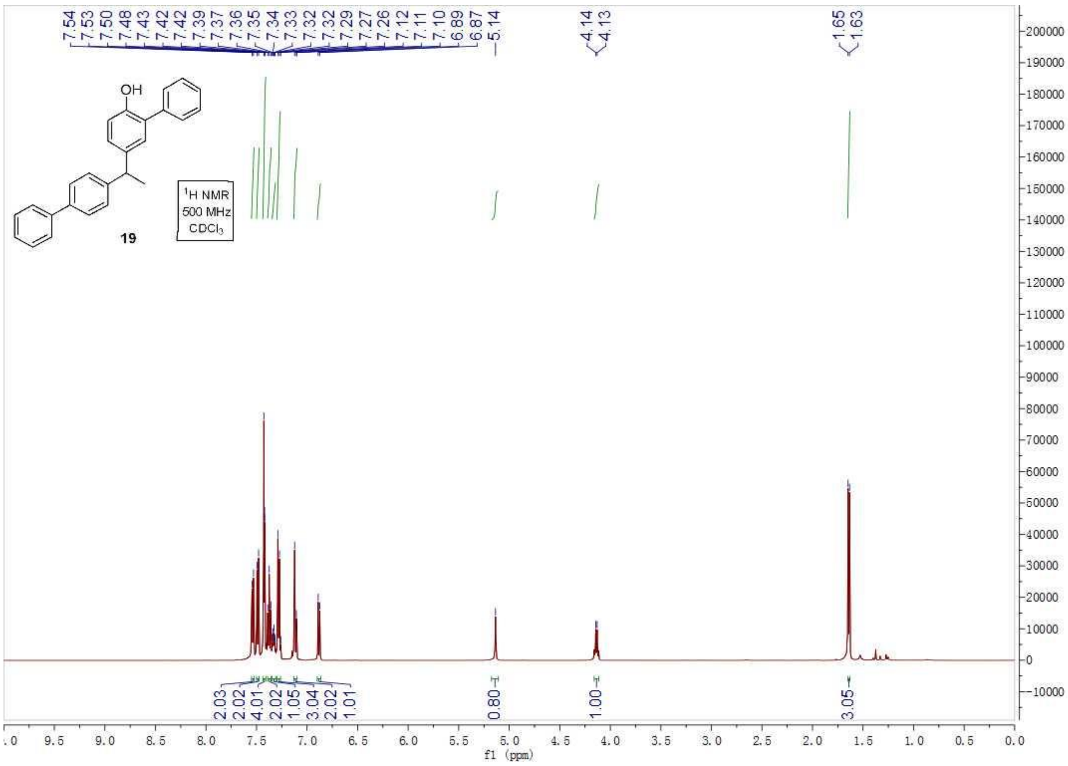

7.54
7.53
7.50
7.48
7.43
7.42
7.42
7.39
7.37
7.36
7.35
7.34
7.33
7.32
7.32
7.29
7.27
7.26
7.12
7.11
7.10
6.89
6.87
5.14
4.14
4.13
1.65
1.63
OH
19
1H NMR
500 MHz
CDCl3
2.03
2.02
4.01
2.02
1.05
3.04
2.02
1.01
0.80
1.00
3.05
f1 (ppm)

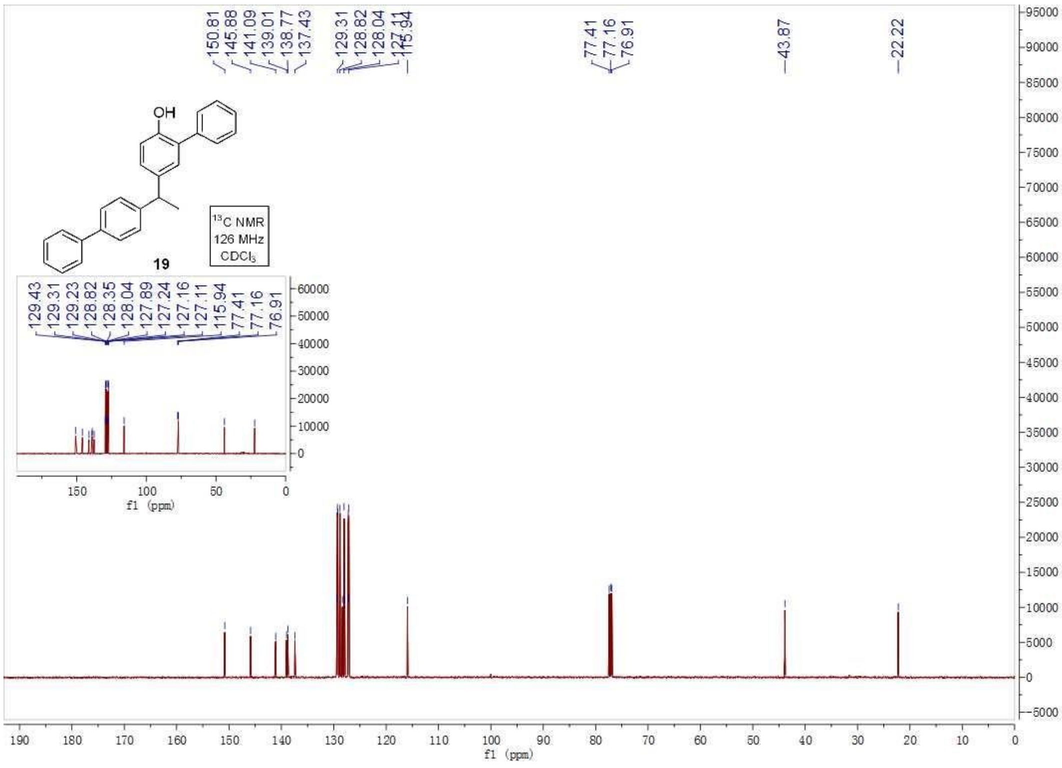

150.81
145.88
141.09
139.01
138.77
137.43
129.31
128.82
128.04
127.11
115.94
77.41
77.16
76.91
43.87
22.22
OH
19
13C NMR
126 MHz
CDCl3
129.43
129.31
129.23
128.82
128.35
128.04
127.89
127.24
127.16
127.11
115.94
77.41
77.16
76.91
f1 (ppm)

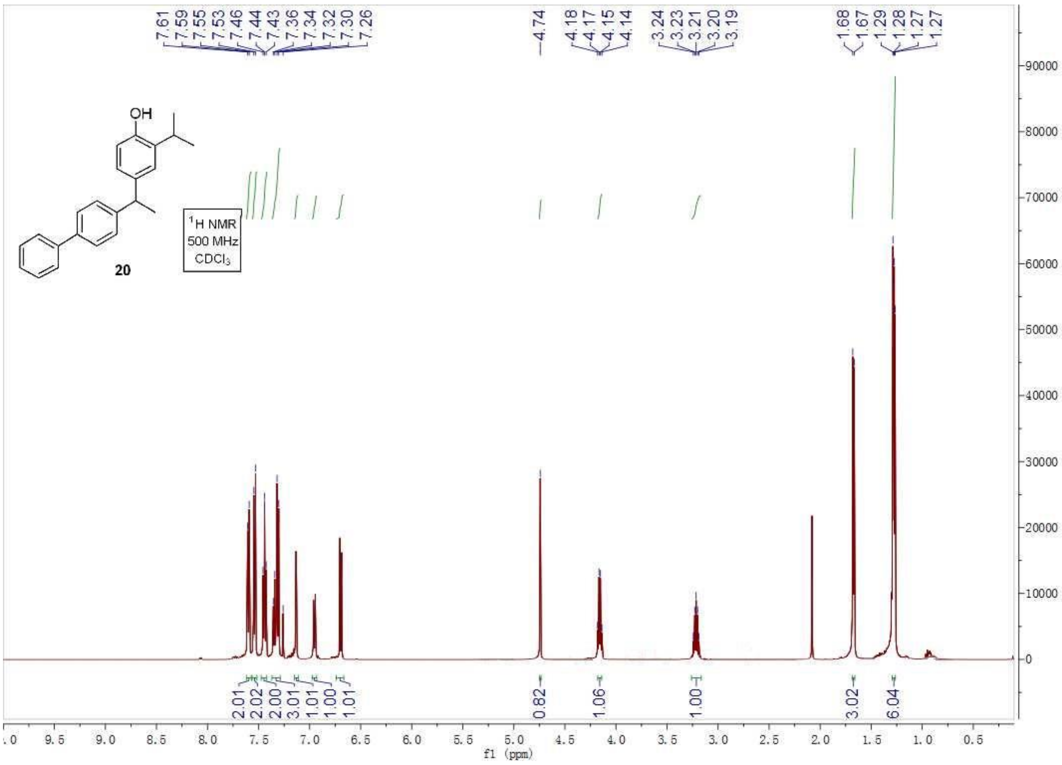

7.61
7.59
7.55
7.53
7.46
7.44
7.43
7.36
7.34
7.32
7.30
7.26
4.74
4.18
4.17
4.15
4.14
3.24
3.23
3.21
3.20
3.19
1.68
1.67
1.29
1.28
1.27
1.27
OH
20
^{1}H NMR
500 MHz
CDCl$_3$
2.01
2.02
2.00
3.01
1.01
1.00
1.01
0.82
1.06
1.00
3.02
6.04
f1 (ppm)

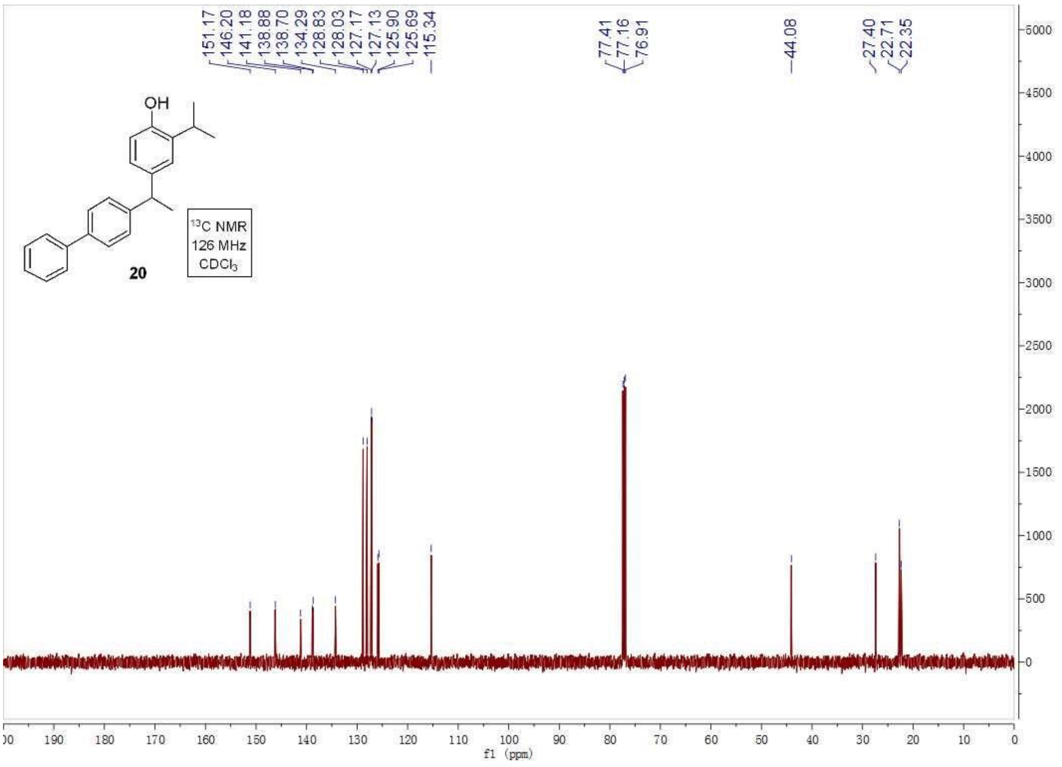

151.17
146.20
141.18
138.88
138.70
134.29
128.83
128.03
127.17
127.13
125.90
125.69
115.34
77.41
77.16
76.91
44.08
27.40
22.71
22.35
OH
20
^{13}C NMR
126 MHz
CDCl$_3$
f1 (ppm)

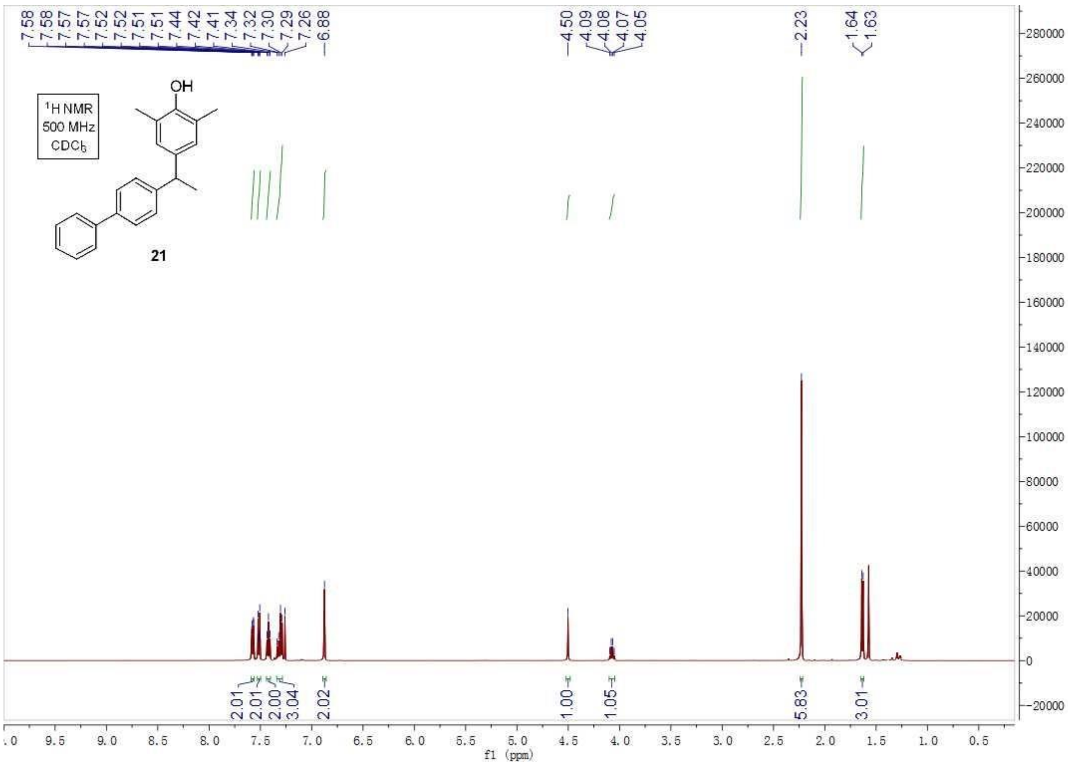

7.58 7.58 7.57 7.57 7.52 7.52 7.51 7.51 7.44 7.42 7.41 7.34 7.32 7.30 7.29 7.26 6.88
4.50 4.09 4.08 4.07 4.05
2.23
1.64 1.63
¹H NMR
500 MHz
CDCl₃
OH
21
2.01 2.01 2.00 3.04 2.02
1.00
1.05
5.83
3.01
f1 (ppm)

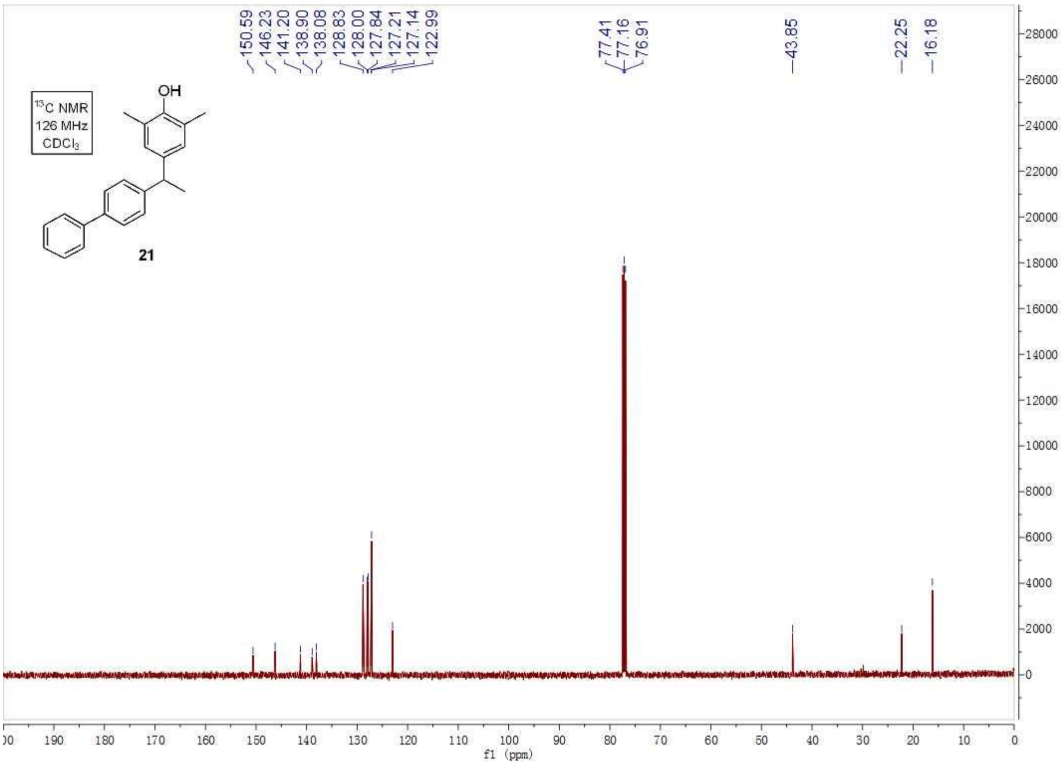

150.59 146.23 141.20 138.90 138.08 128.83 128.00 127.84 127.21 127.14 122.99
77.41 77.16 76.91
43.85
22.25
16.18
¹³C NMR
126 MHz
CDCl₃
OH
21
f1 (ppm)

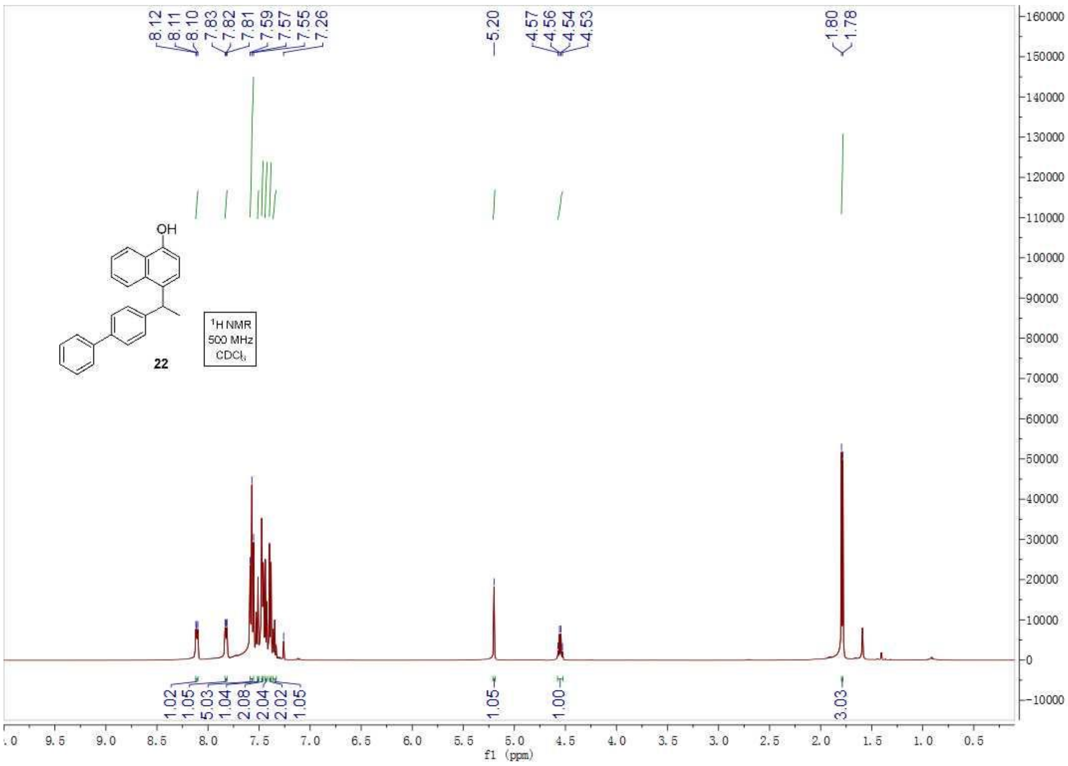
8.12
8.11
8.10
7.83
7.82
7.81
7.59
7.57
7.55
7.26
5.20
4.57
4.56
4.54
4.53
1.80
1.78
OH
22
^{1}H NMR
500 MHz
CDCl$_3$
1.02
1.05
5.03
1.04
2.08
2.04
2.02
1.05
1.05
1.00
3.03
f1 (ppm)

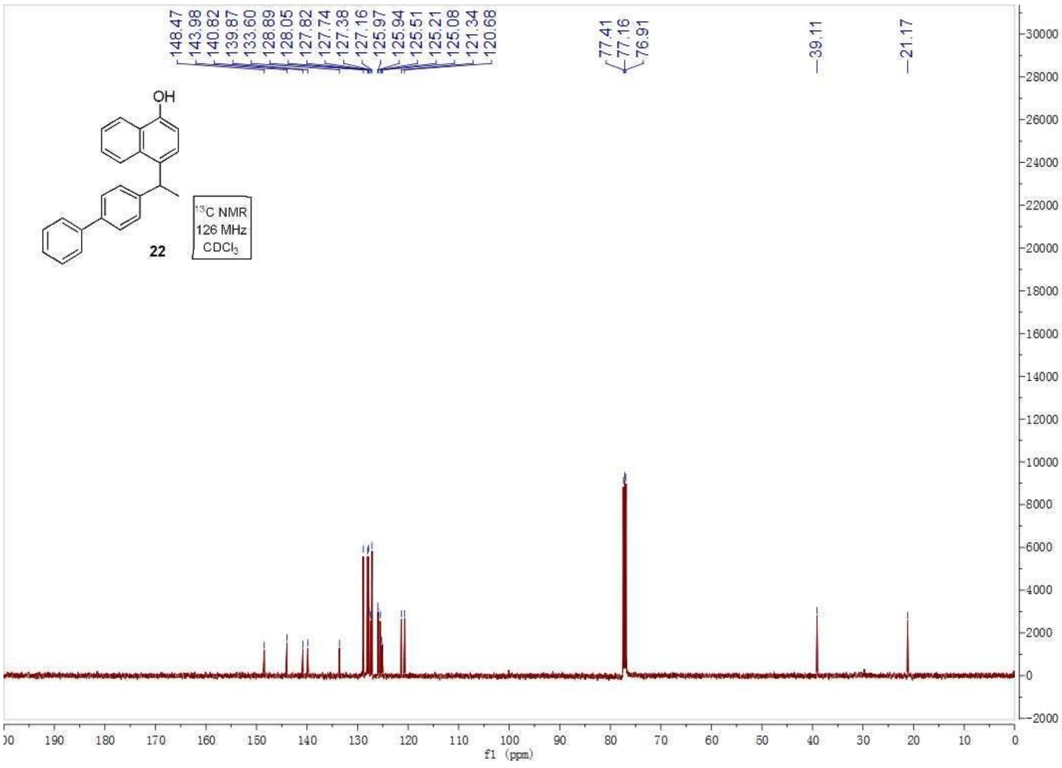
148.47
143.98
140.82
139.87
133.60
128.89
128.05
127.82
127.74
127.38
127.16
125.97
125.94
125.51
125.21
125.08
121.34
120.68
77.41
77.16
76.91
39.11
21.17
OH
22
^{13}C NMR
126 MHz
CDCl$_3$
f1 (ppm)

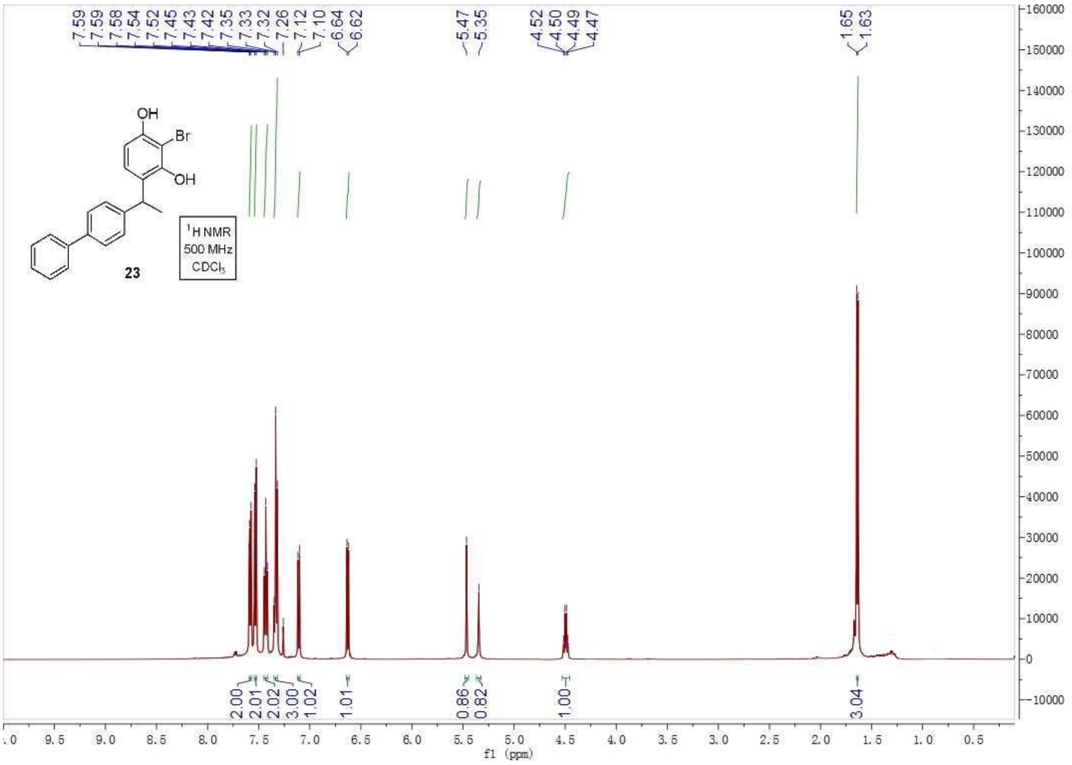

OH
Br
OH
23
^{1}H NMR
500 MHz
$CDCl_3$
7.59
7.59
7.58
7.54
7.52
7.45
7.43
7.42
7.35
7.33
7.32
7.26
7.12
7.10
6.64
6.62
5.47
5.35
4.52
4.50
4.49
4.47
1.65
1.63
2.00
2.01
2.02
3.00
1.02
1.01
0.86
0.82
1.00
3.04
f1 (ppm)

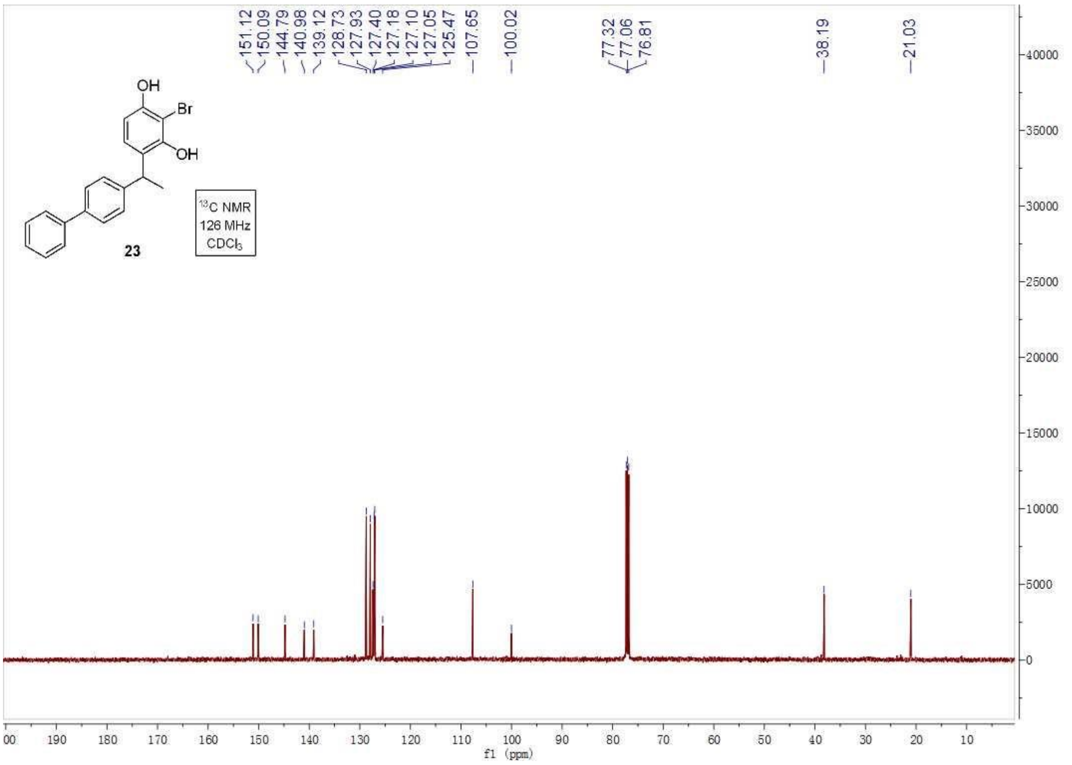

OH
Br
OH
23
^{13}C NMR
126 MHz
$CDCl_3$
151.12
150.09
144.79
140.98
139.12
128.73
127.93
127.40
127.18
127.10
127.05
125.47
107.65
100.02
77.32
77.06
76.81
38.19
21.03
f1 (ppm)

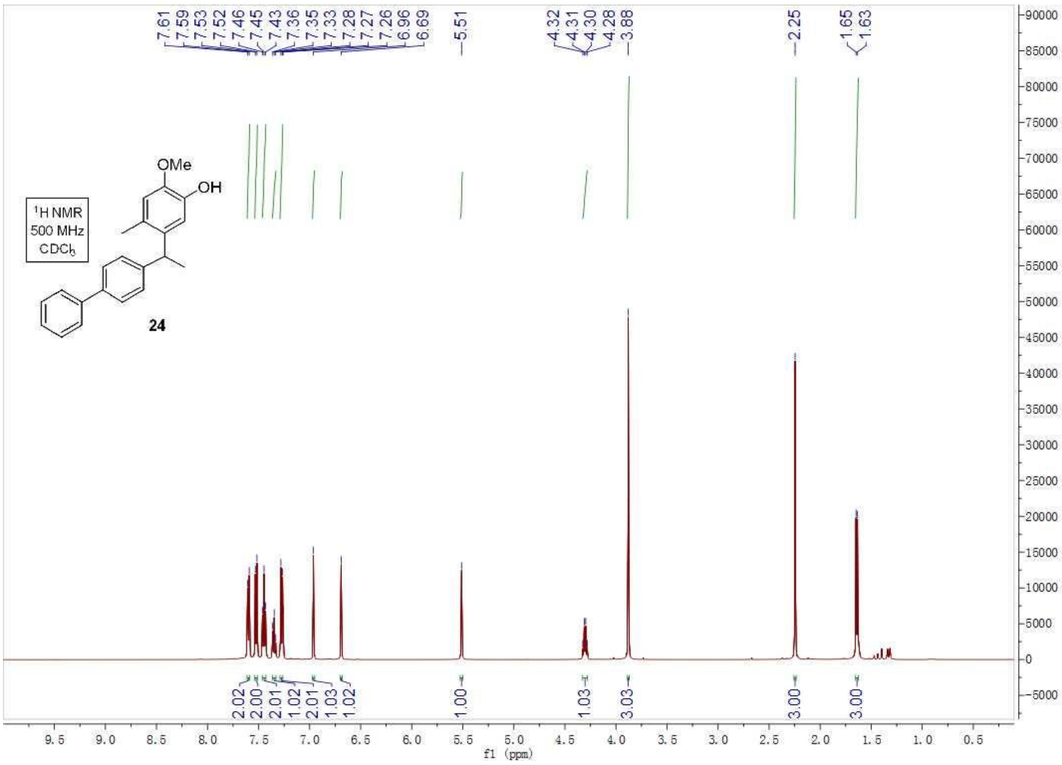
7.61
7.59
7.53
7.52
7.46
7.45
7.43
7.36
7.35
7.33
7.28
7.27
7.26
6.96
6.69
5.51
4.32
4.31
4.30
4.28
3.88
2.25
1.65
1.63
OMe
OH
1H NMR
500 MHz
CDCl3
24
2.02
2.00
2.01
1.02
2.01
1.03
1.02
1.00
1.03
3.03
3.00
3.00
f1 (ppm)

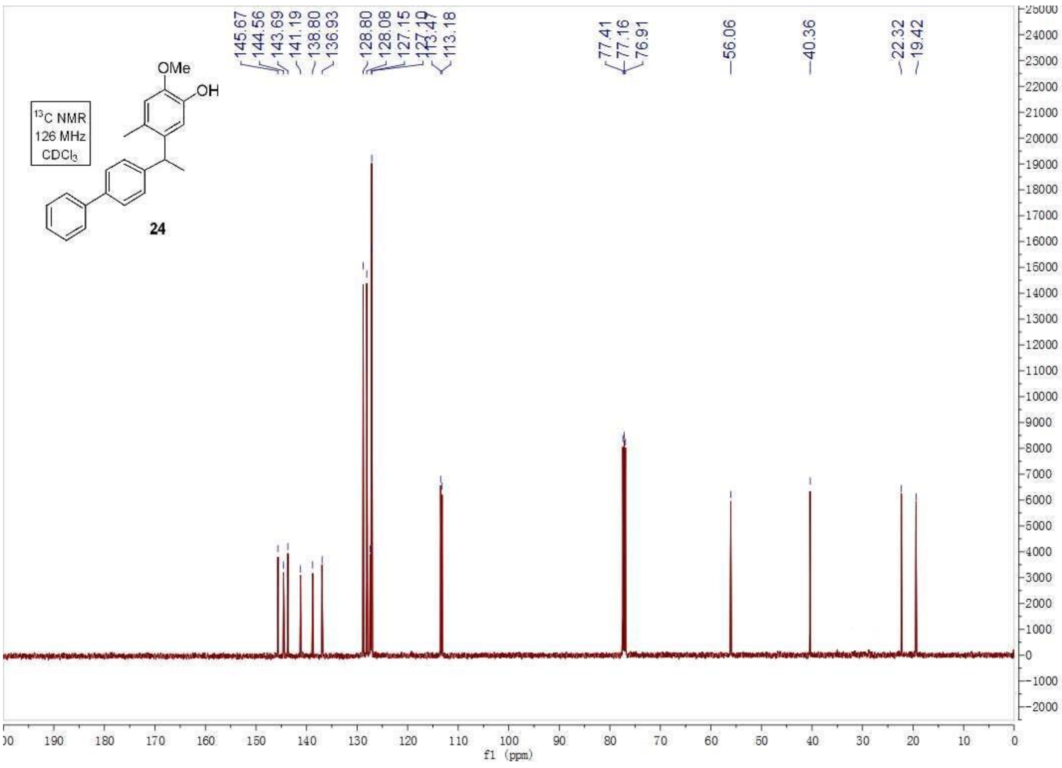
145.67
144.56
143.69
141.19
138.80
136.93
128.80
128.08
127.15
127.10
113.47
113.18
77.41
77.16
76.91
56.06
40.36
22.32
19.42
OMe
OH
13C NMR
126 MHz
CDCl3
24
f1 (ppm)

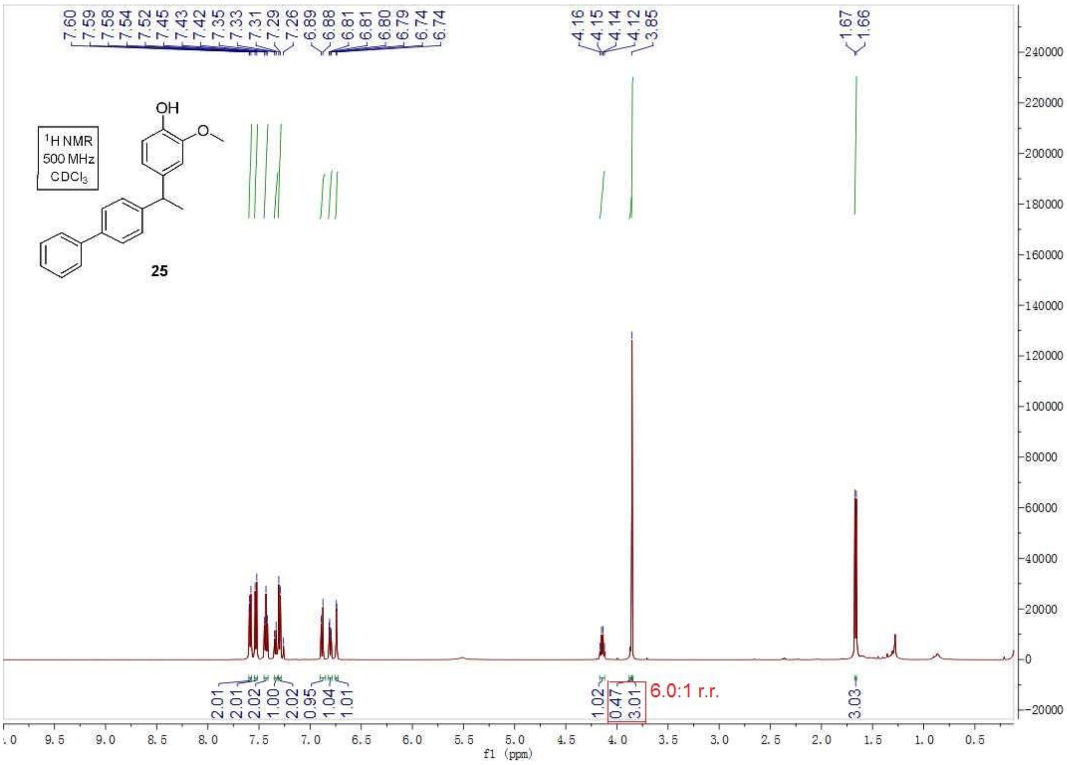

1H NMR
500 MHz
CDCl3
25
6.0:1 r.r.
f1 (ppm)

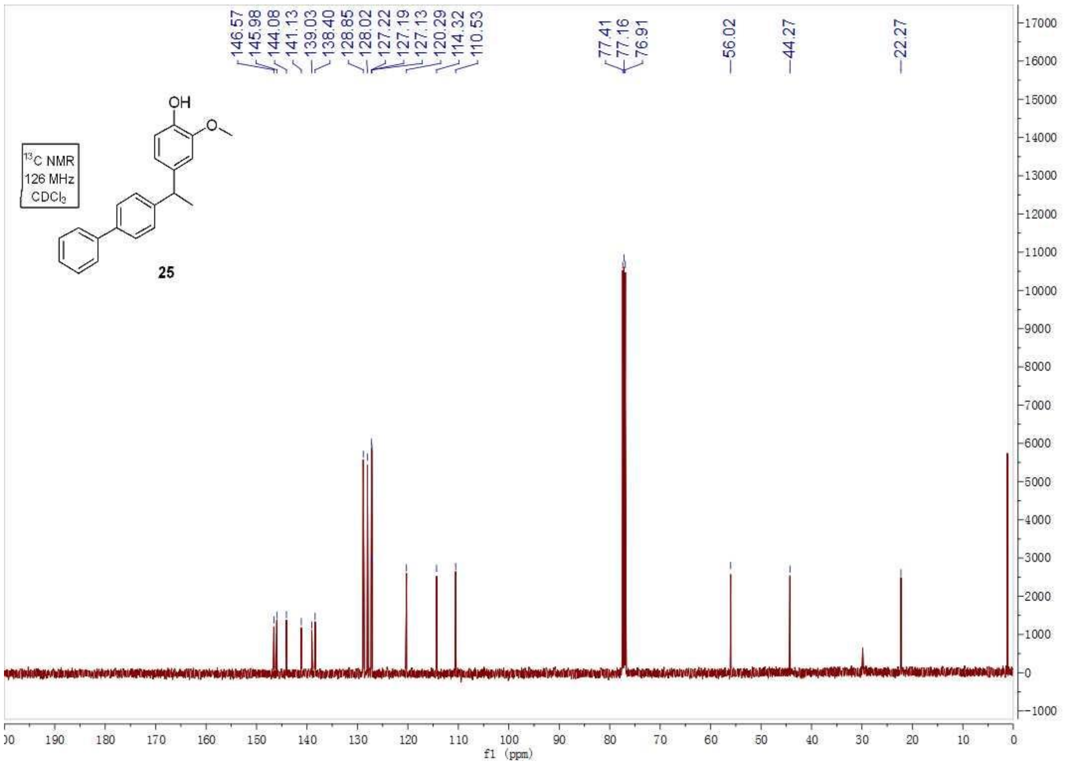

13C NMR
126 MHz
CDCl3
25
f1 (ppm)

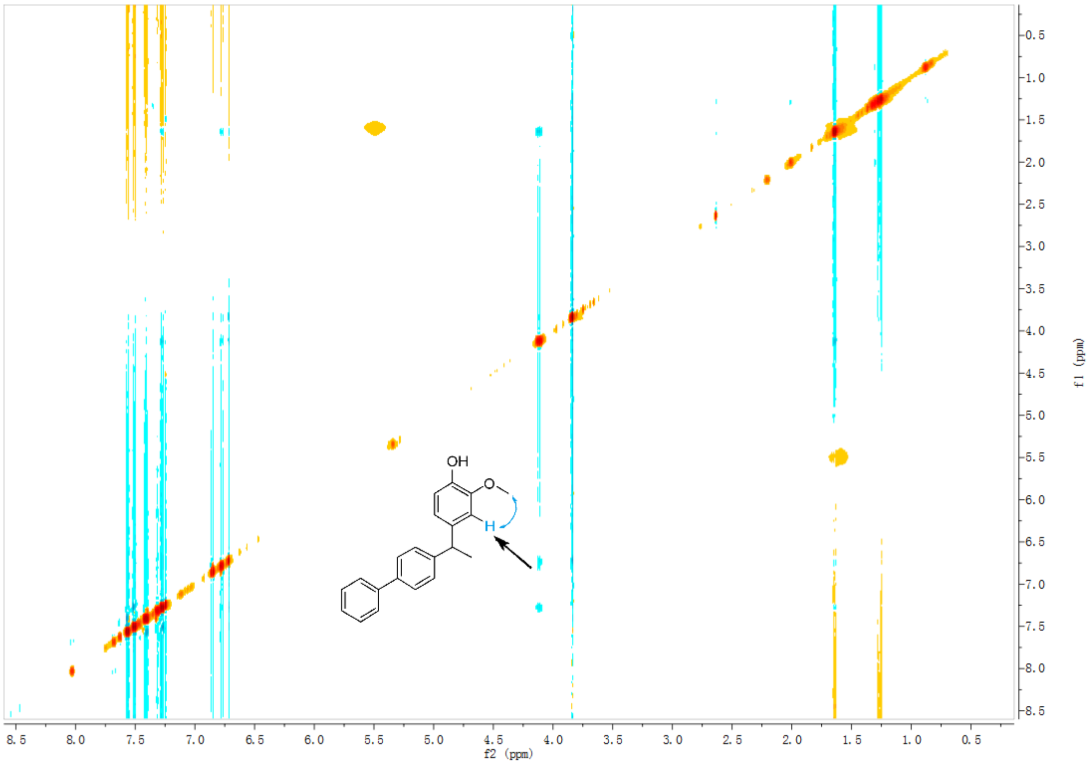
OH
O
H
f2 (ppm)
f1 (ppm)

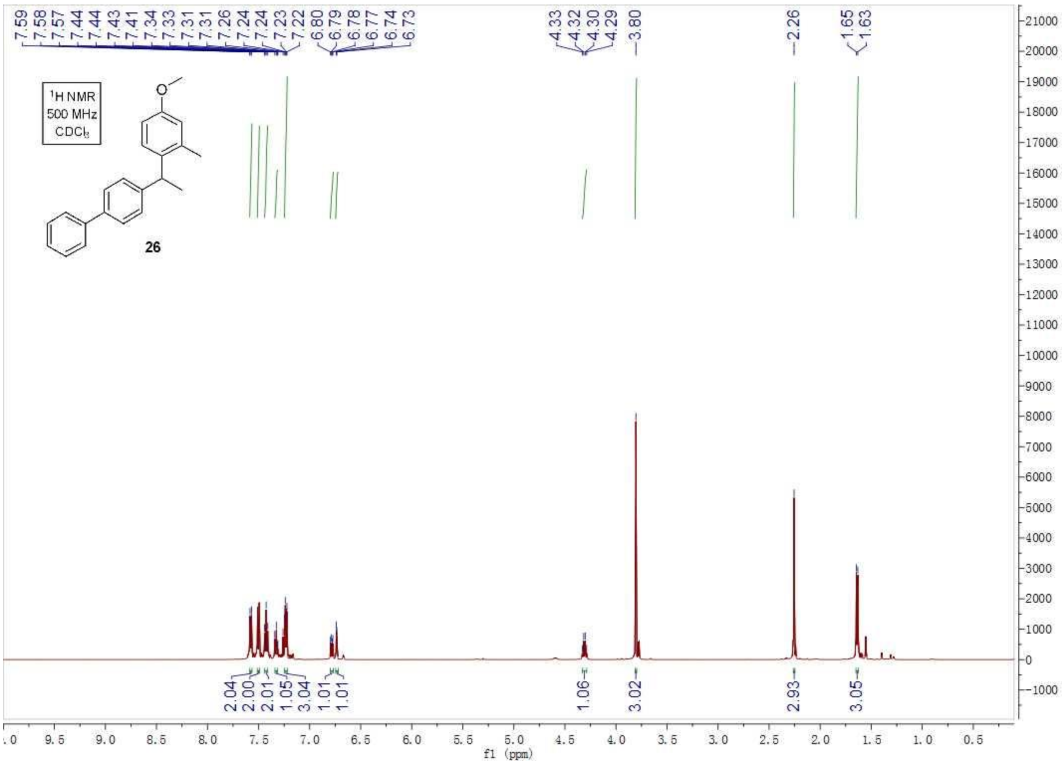

7.59
7.58
7.57
7.44
7.44
7.43
7.41
7.34
7.33
7.31
7.31
7.26
7.24
7.24
7.23
7.22
6.80
6.79
6.78
6.77
6.74
6.73
4.33
4.32
4.30
4.29
3.80
2.26
1.65
1.63
^{1}H NMR
500 MHz
$CDCl_3$
26
2.04
2.00
2.01
1.05
3.04
1.01
1.01
1.06
3.02
2.93
3.05
f1 (ppm)

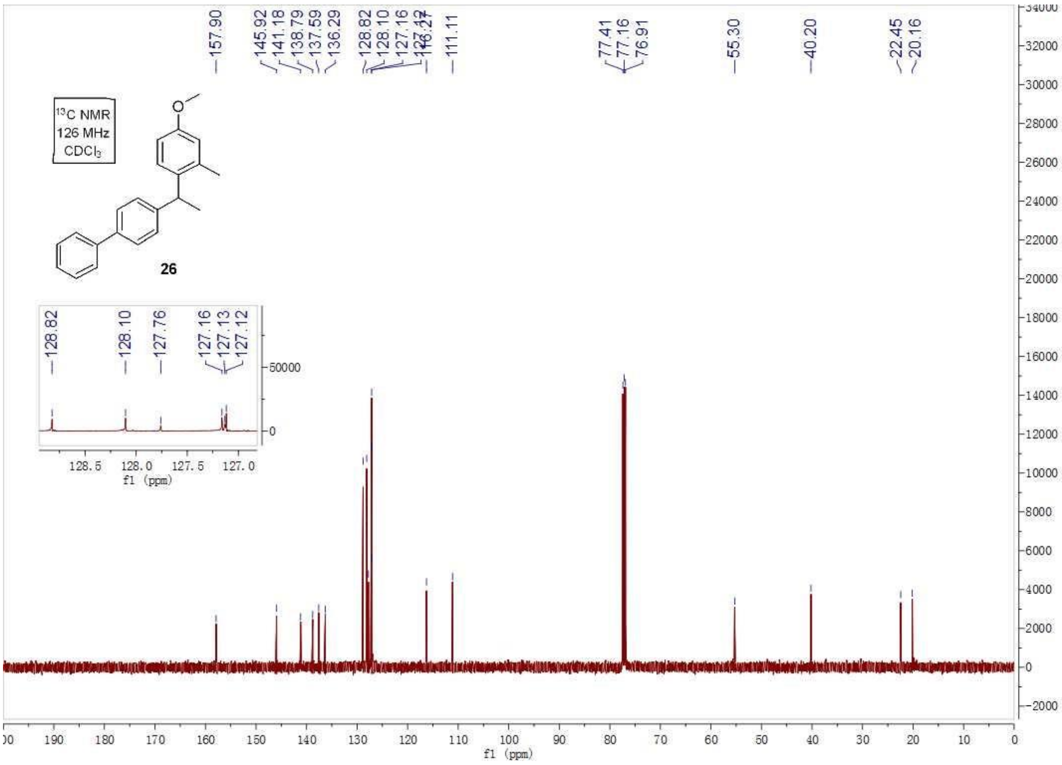

157.90
145.92
141.18
138.79
137.59
136.29
128.82
128.10
127.16
111.11
77.41
77.16
76.91
55.30
40.20
22.45
20.16
^{13}C NMR
126 MHz
$CDCl_3$
26
128.82
128.10
127.76
127.16
127.13
127.12
f1 (ppm)

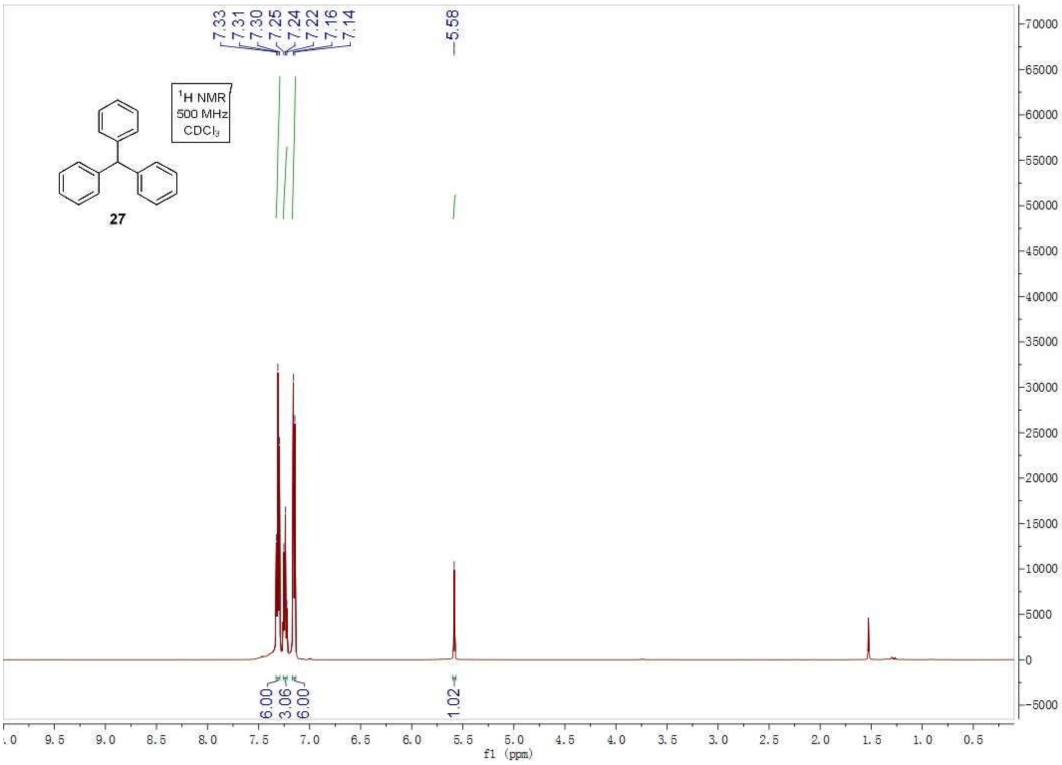
7.33
7.31
7.30
7.25
7.24
7.22
7.16
7.14
5.58
1H NMR
500 MHz
CDCl3
27
6.00
3.06
6.00
1.02
f1 (ppm)

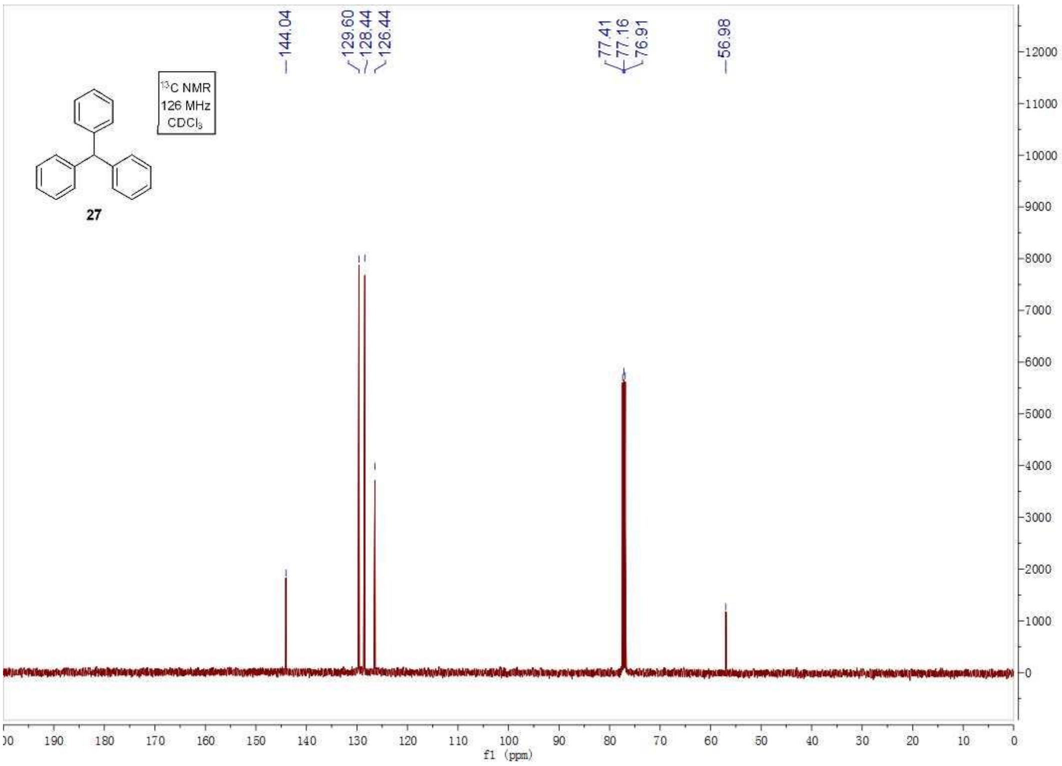
144.04
129.60
128.44
126.44
77.41
77.16
76.91
56.98
13C NMR
126 MHz
CDCl3
27
f1 (ppm)

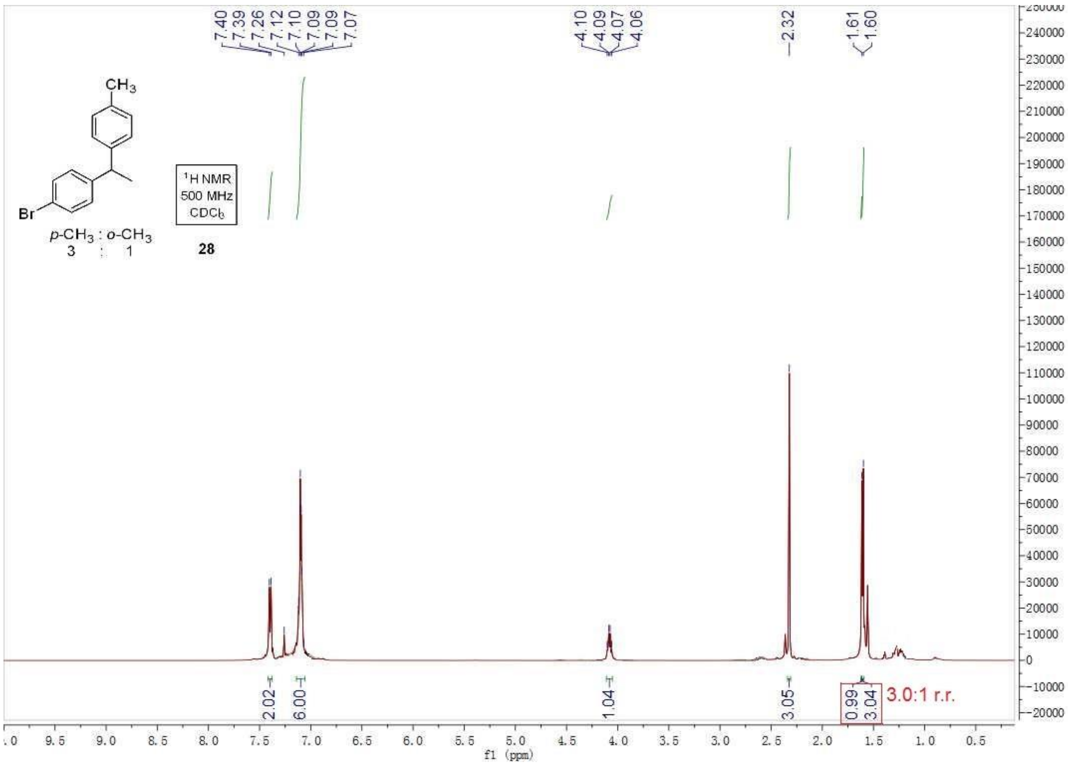

7.40
7.39
7.26
7.12
7.10
7.09
7.09
7.07
4.10
4.09
4.07
4.06
2.32
1.61
1.60
CH3
Br
p-CH3 : o-CH3
3 : 1
1H NMR
500 MHz
CDCl3
28
2.02
6.00
1.04
3.05
0.99
3.04
3.0:1 r.r.
f1 (ppm)

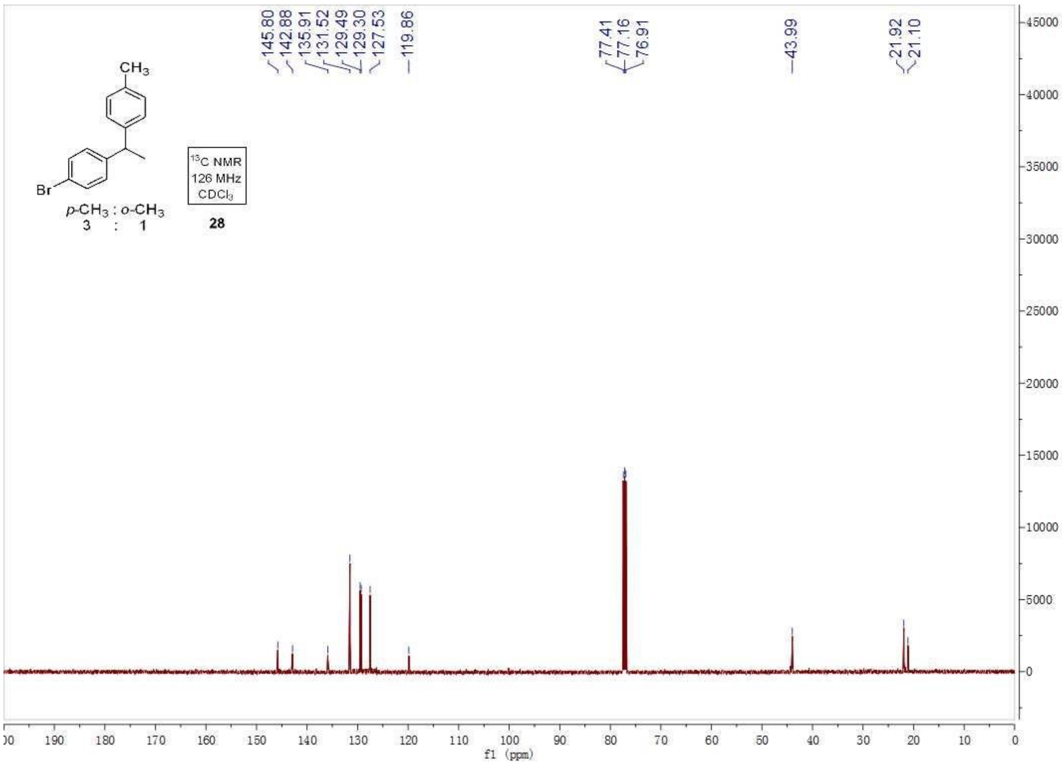

145.80
142.88
135.91
131.52
129.49
129.30
127.53
119.86
77.41
77.16
76.91
43.99
21.92
21.10
CH3
Br
p-CH3 : o-CH3
3 : 1
13C NMR
126 MHz
CDCl3
28
f1 (ppm)

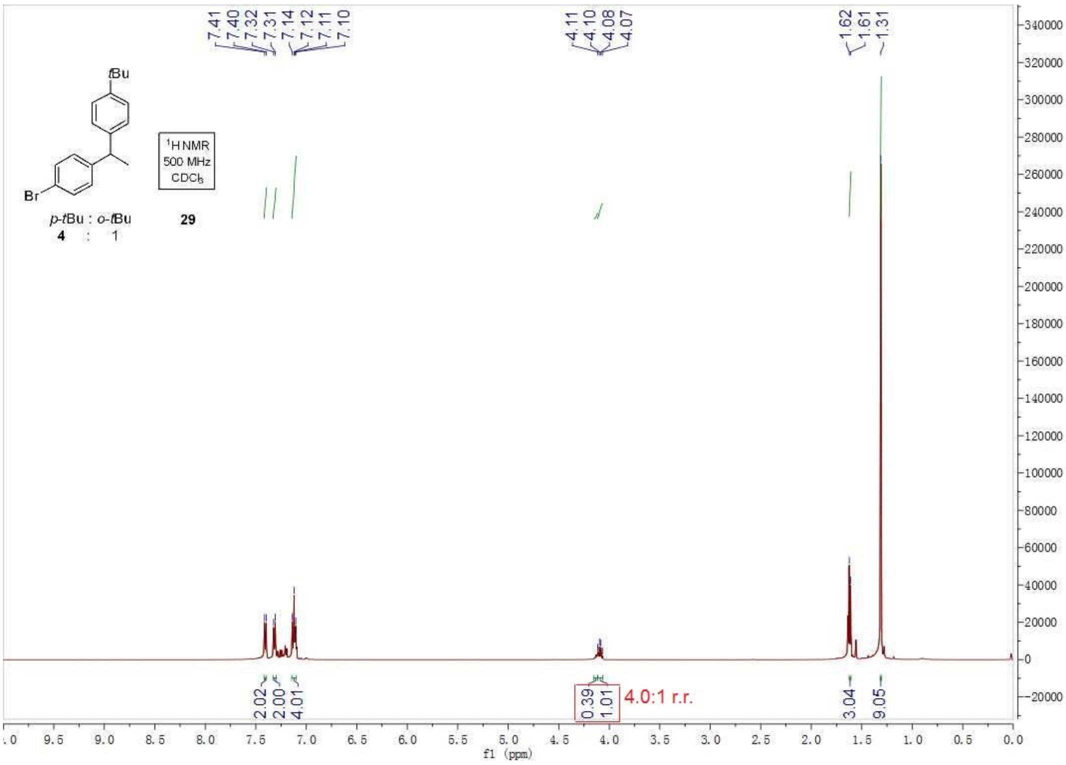

tBu
Br
p-tBu : o-tBu
4 : 1
1H NMR
500 MHz
CDCl3
29
7.41
7.40
7.32
7.31
7.14
7.12
7.11
7.10
4.11
4.10
4.08
4.07
1.62
1.61
1.31
2.02
2.00
4.01
0.39
1.01
4.0:1 r.r.
3.04
9.05
f1 (ppm)

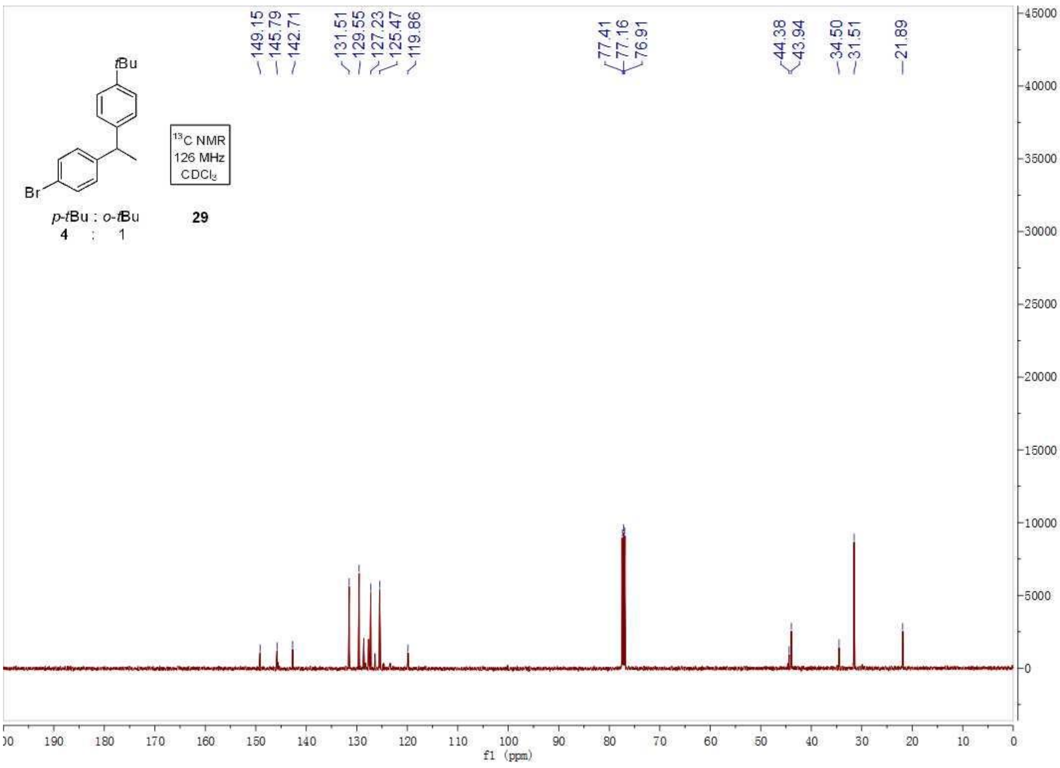

tBu
Br
p-tBu : o-tBu
4 : 1
13C NMR
126 MHz
CDCl3
29
149.15
145.79
142.71
131.51
129.55
127.23
125.47
119.86
77.41
77.16
76.91
44.38
43.94
34.50
31.51
21.89
f1 (ppm)

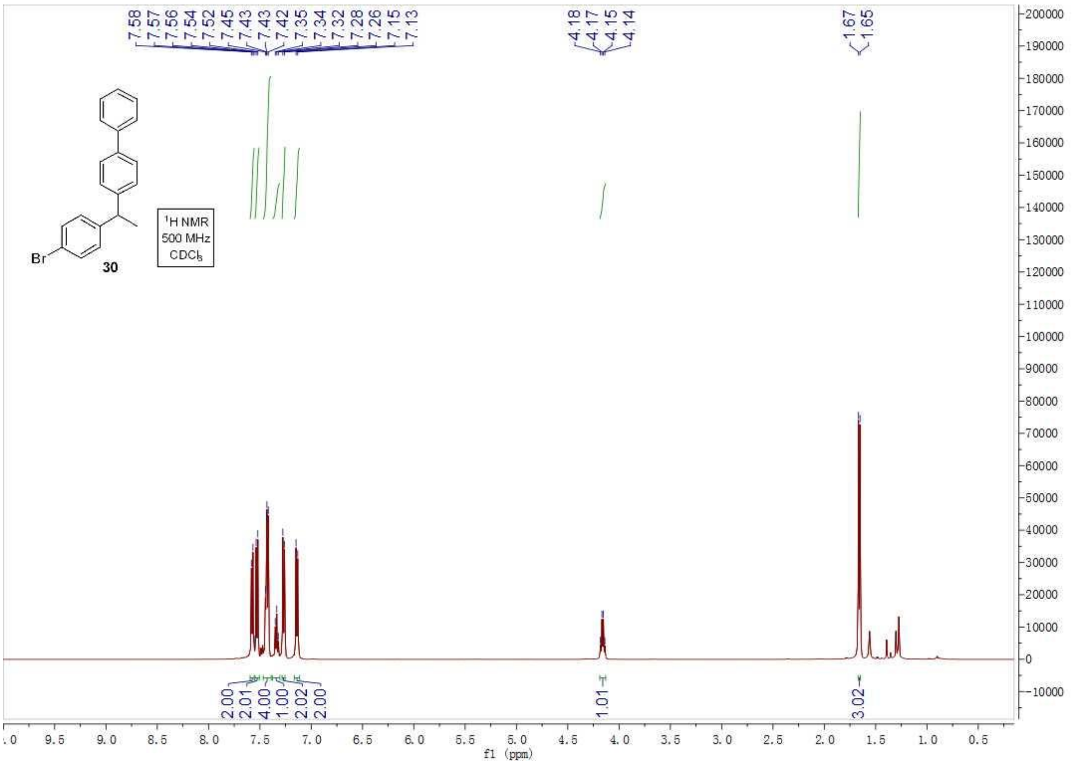
7.58
7.57
7.56
7.54
7.52
7.45
7.43
7.43
7.42
7.35
7.34
7.32
7.28
7.26
7.15
7.13
4.18
4.17
4.15
4.14
1.67
1.65
Br
30
^{1}H NMR
500 MHz
$CDCl_3$
2.00
2.01
4.00
1.00
2.02
2.00
1.01
3.02
f1 (ppm)

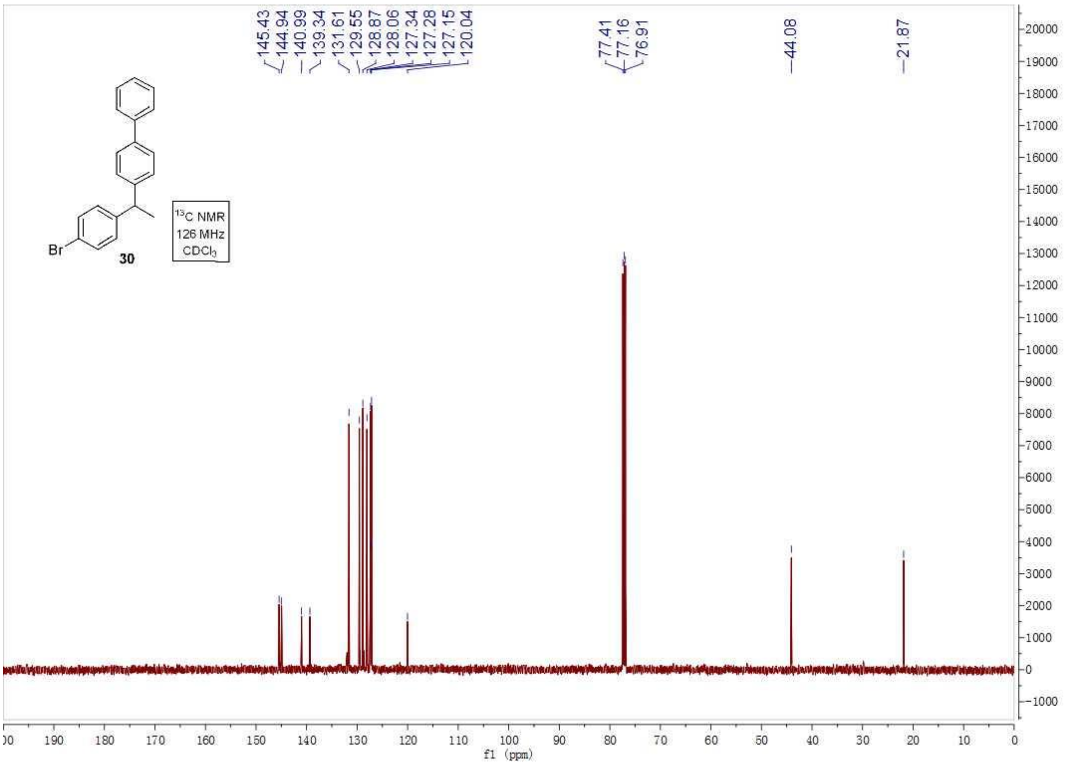
145.43
144.94
140.99
139.34
131.61
129.55
128.87
128.06
127.34
127.28
127.15
120.04
77.41
77.16
76.91
44.08
21.87
Br
30
^{13}C NMR
126 MHz
$CDCl_3$
f1 (ppm)

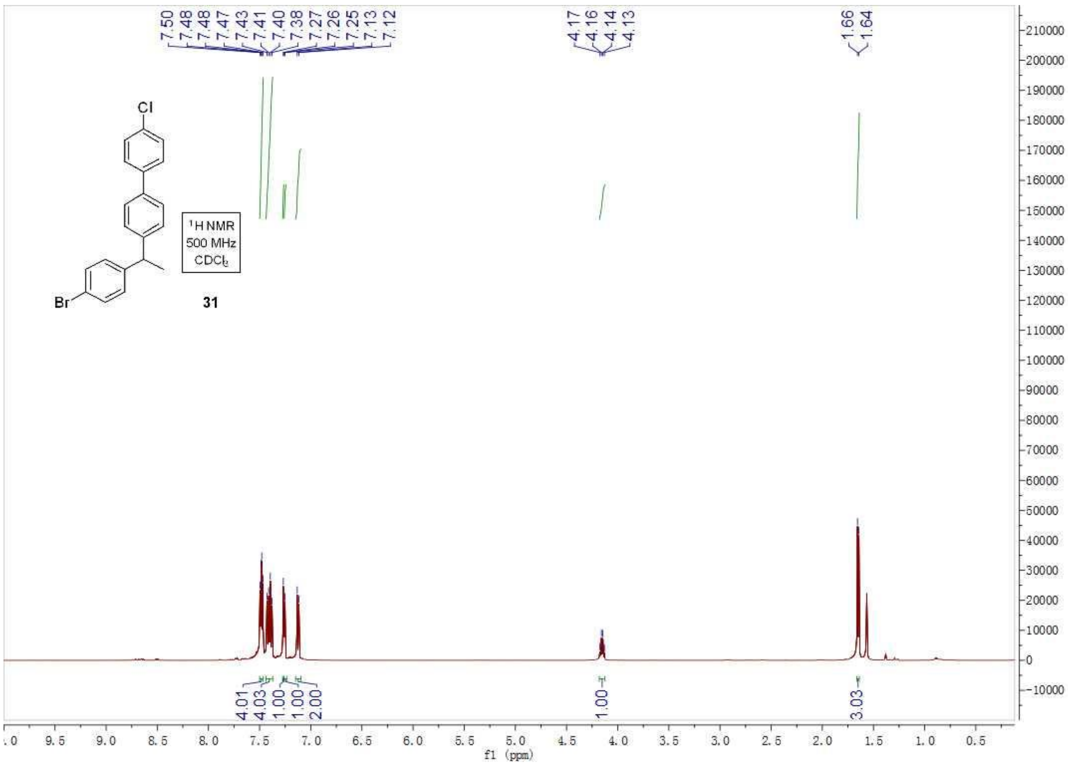
7.50
7.48
7.48
7.47
7.43
7.41
7.40
7.38
7.27
7.26
7.25
7.13
7.12
4.17
4.16
4.14
4.13
1.66
1.64
Cl
Br
^{1}H NMR
500 MHz
$CDCl_3$
31
4.01
4.03
1.00
1.00
2.00
1.00
3.03
f1 (ppm)

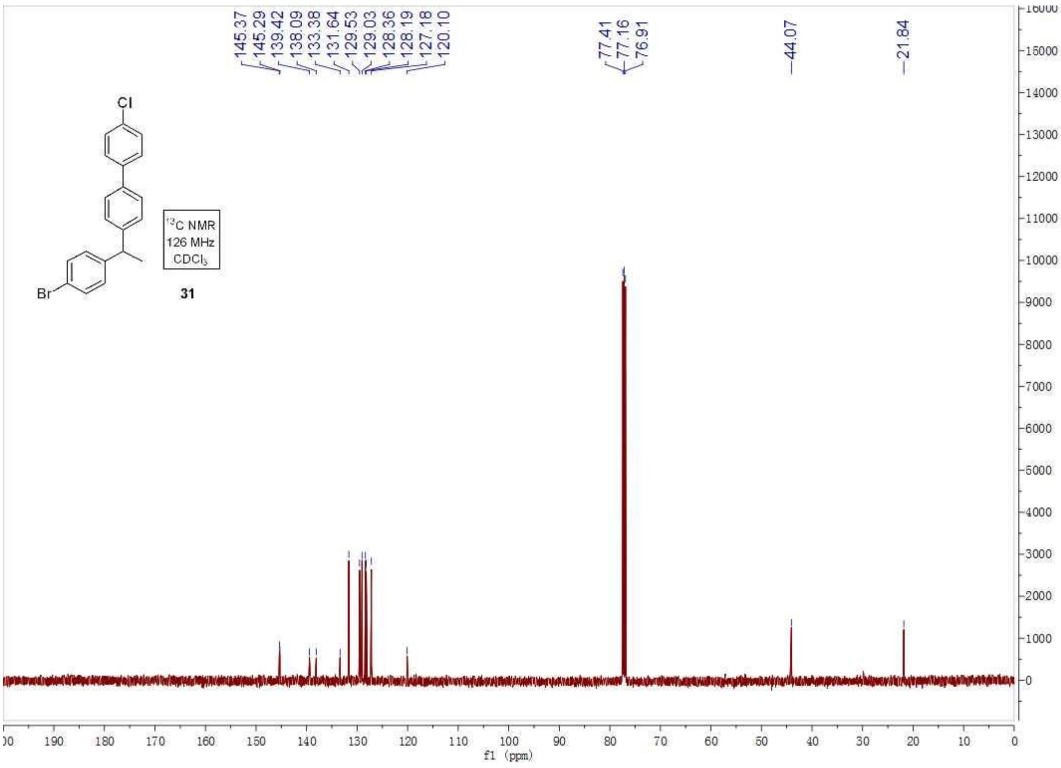
145.37
145.29
139.42
138.09
133.38
131.64
129.53
129.03
128.36
128.19
127.18
120.10
77.41
77.16
76.91
44.07
21.84
Cl
Br
^{13}C NMR
126 MHz
$CDCl_3$
31
f1 (ppm)

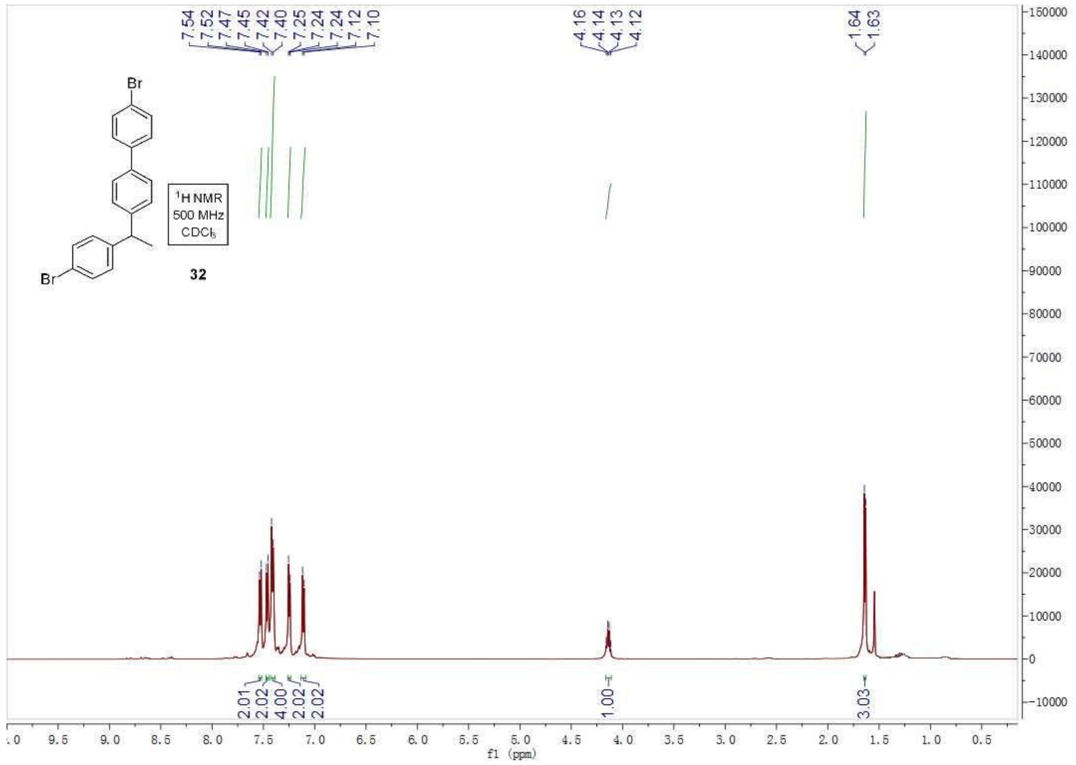

1H NMR
500 MHz
CDCl3
32

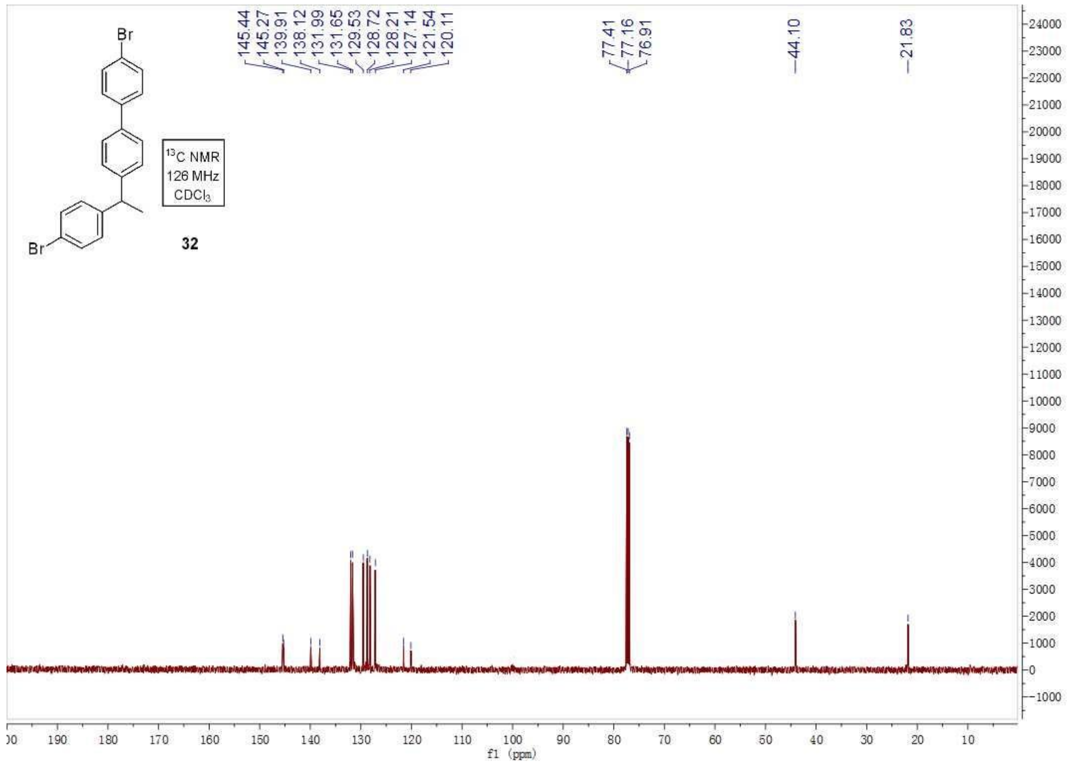

13C NMR
126 MHz
CDCl3
32

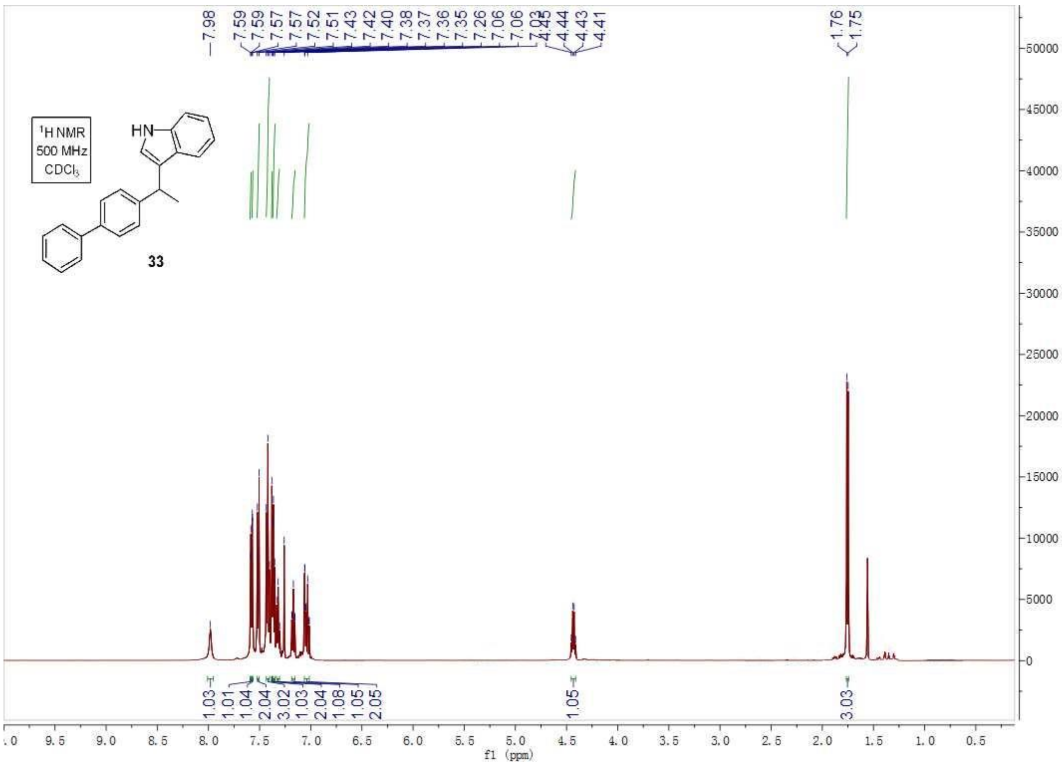

^{1}H NMR
500 MHz
CDCl$_3$
HN
33
f1 (ppm)

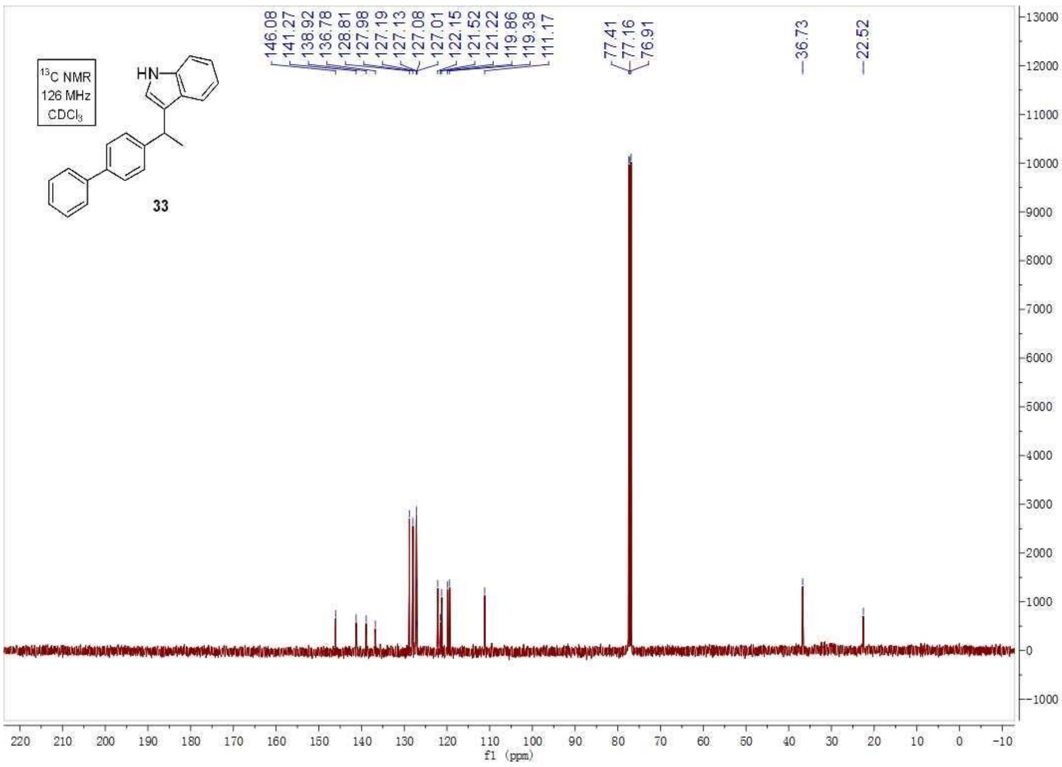

^{13}C NMR
126 MHz
CDCl$_3$
HN
33
f1 (ppm)

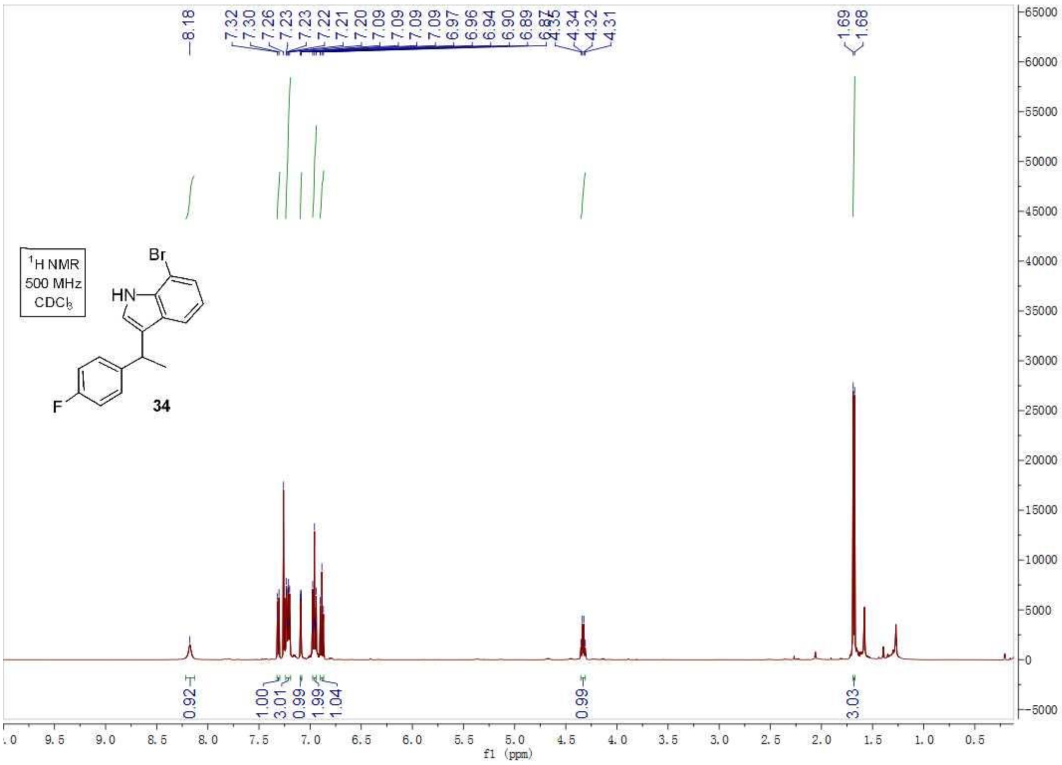

1H NMR
500 MHz
CDCl3
Br
HN
F
34

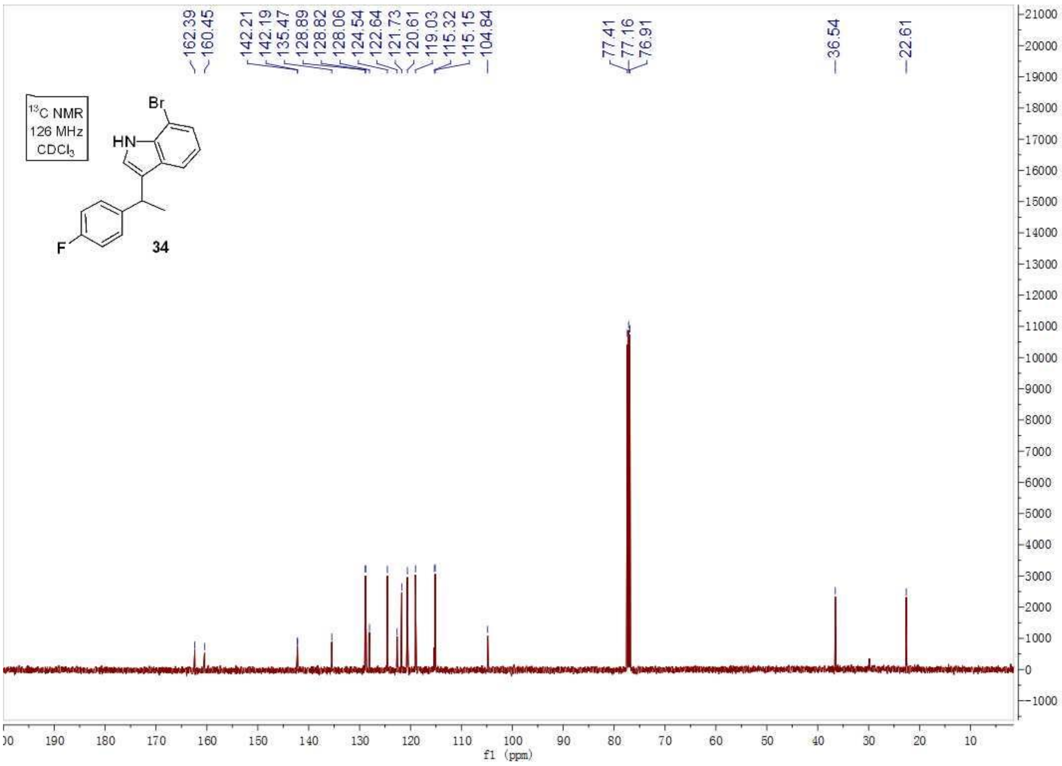

13C NMR
126 MHz
CDCl3
Br
HN
F
34

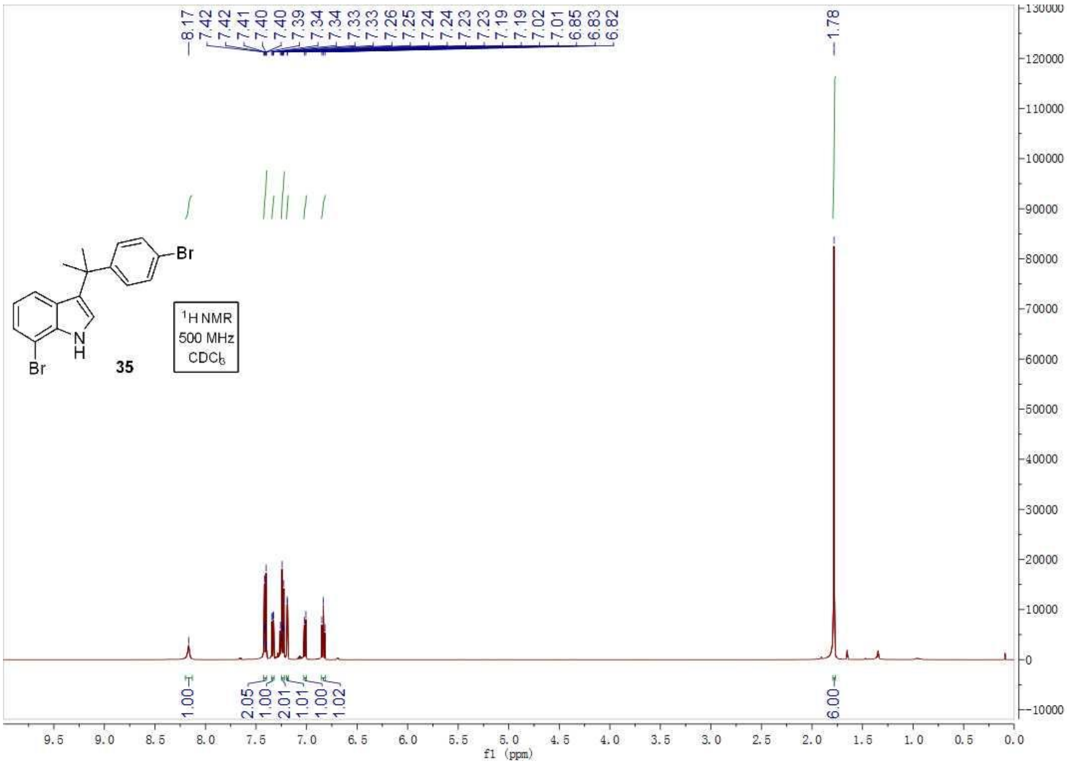

8.17
7.42
7.42
7.41
7.40
7.40
7.39
7.34
7.34
7.33
7.33
7.26
7.25
7.24
7.24
7.23
7.23
7.19
7.19
7.02
7.01
6.85
6.83
6.82
1.78
Br
Br
N
H
35
1H NMR
500 MHz
CDCl3
1.00
2.05
1.00
2.01
1.01
1.00
1.02
6.00
f1 (ppm)

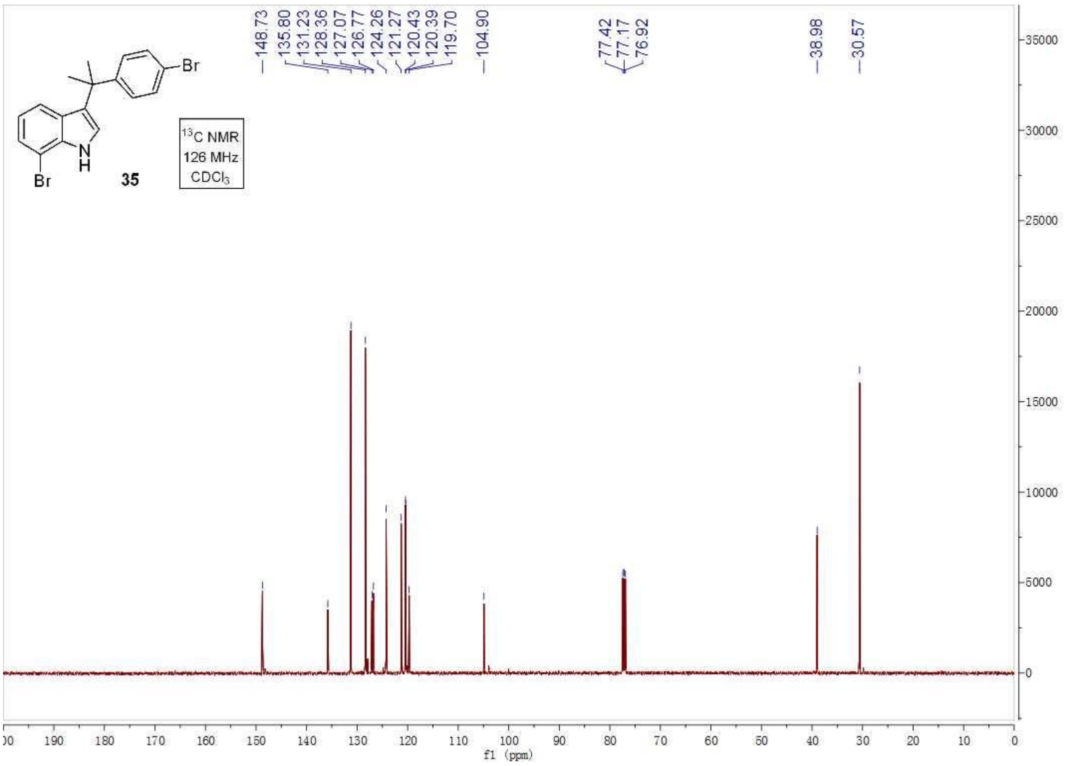

148.73
135.80
131.23
128.36
127.07
126.77
124.26
121.27
120.43
120.39
119.70
104.90
77.42
77.17
76.92
38.98
30.57
Br
Br
N
H
35
13C NMR
126 MHz
CDCl3
f1 (ppm)

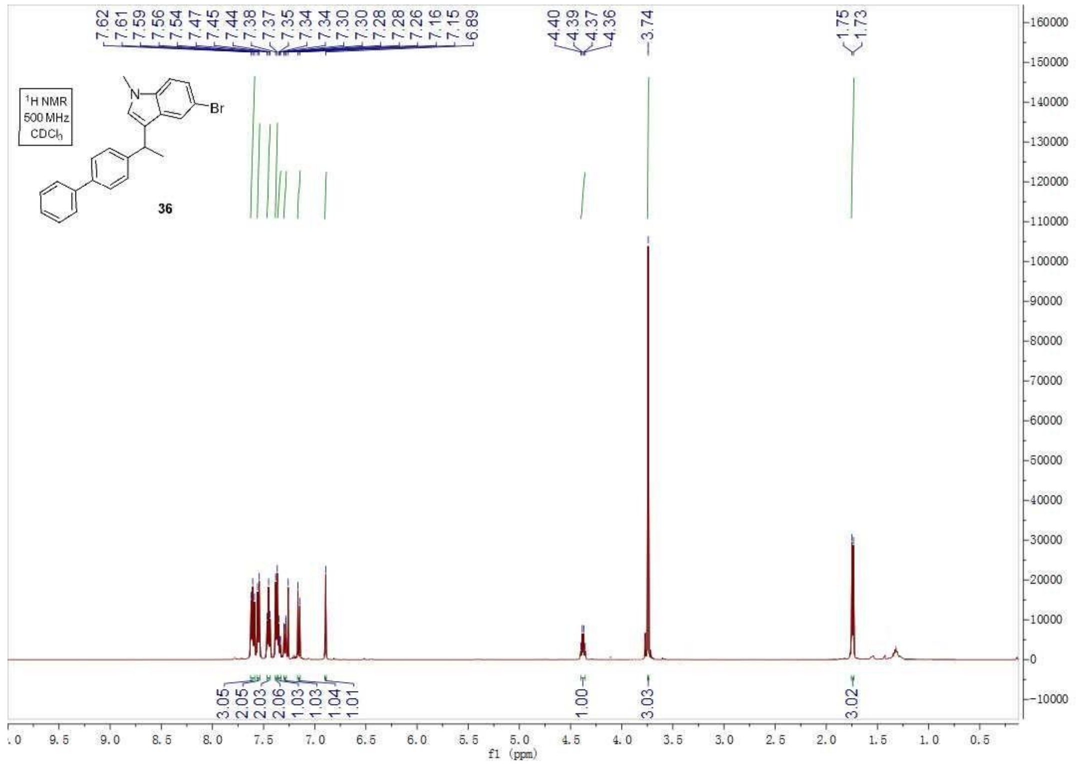

7.62
7.61
7.59
7.56
7.54
7.47
7.45
7.44
7.38
7.37
7.35
7.34
7.34
7.30
7.30
7.28
7.28
7.26
7.16
7.15
6.89
4.40
4.39
4.37
4.36
3.74
1.75
1.73
^{1}H NMR
500 MHz
$CDCl_3$
Br
N
36
3.05
2.05
2.03
2.06
1.03
1.03
1.04
1.01
1.00
3.03
3.02
f1 (ppm)

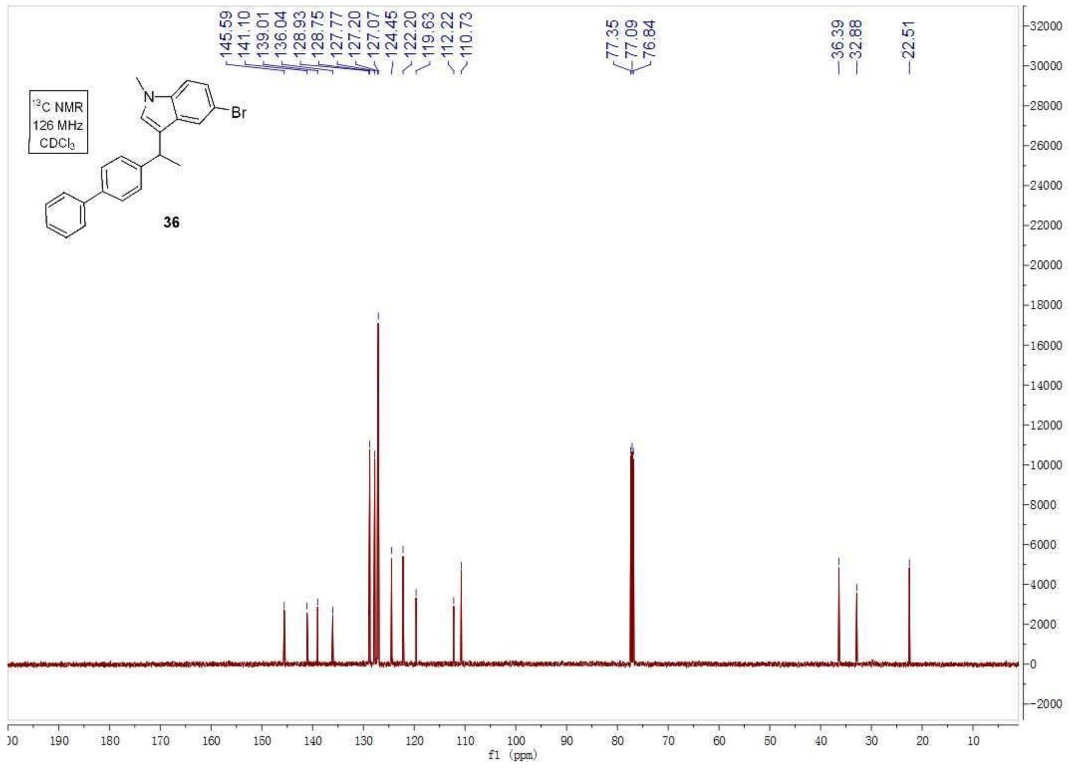

145.59
141.10
139.01
136.04
128.93
128.75
127.77
127.20
127.07
124.45
122.20
119.63
112.22
110.73
77.35
77.09
76.84
36.39
32.88
22.51
^{13}C NMR
126 MHz
$CDCl_3$
Br
N
36
f1 (ppm)

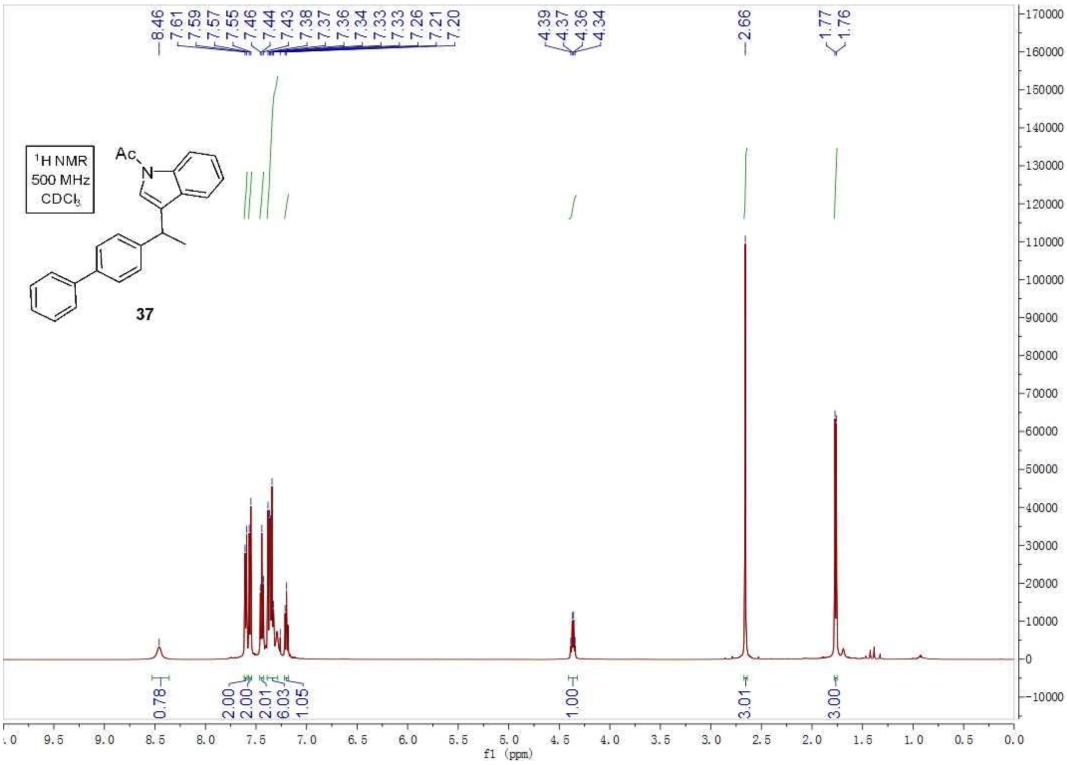

1H NMR
500 MHz
CDCl3
37

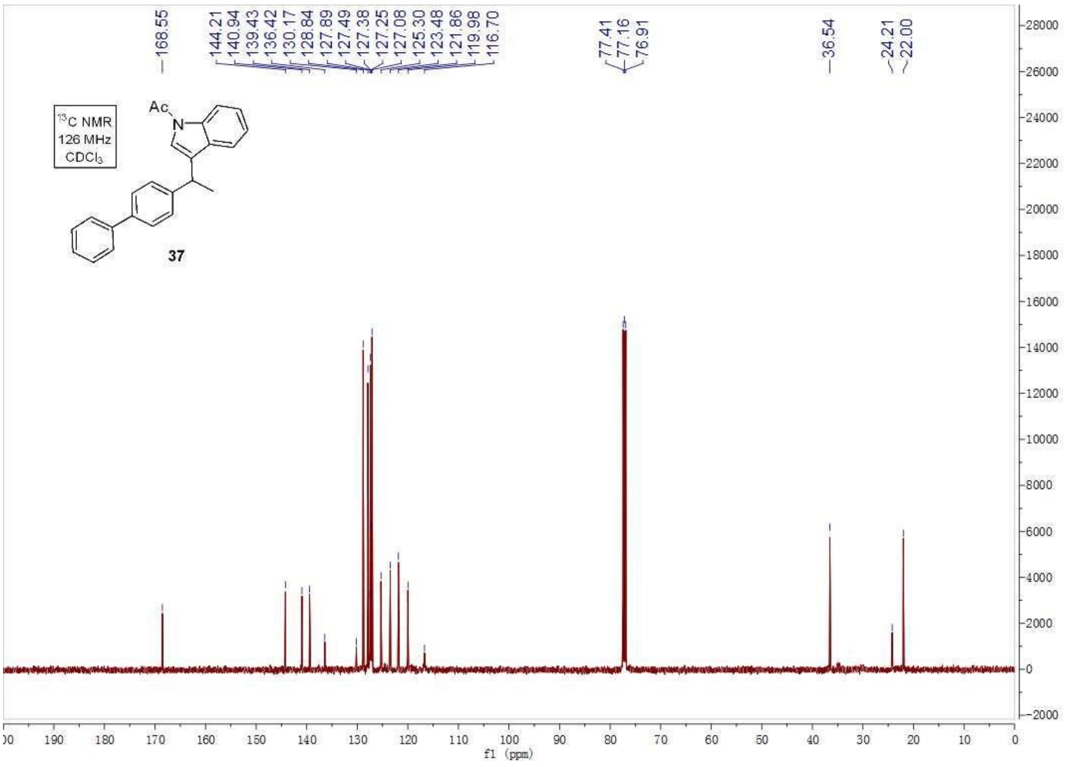

13C NMR
126 MHz
CDCl3
37

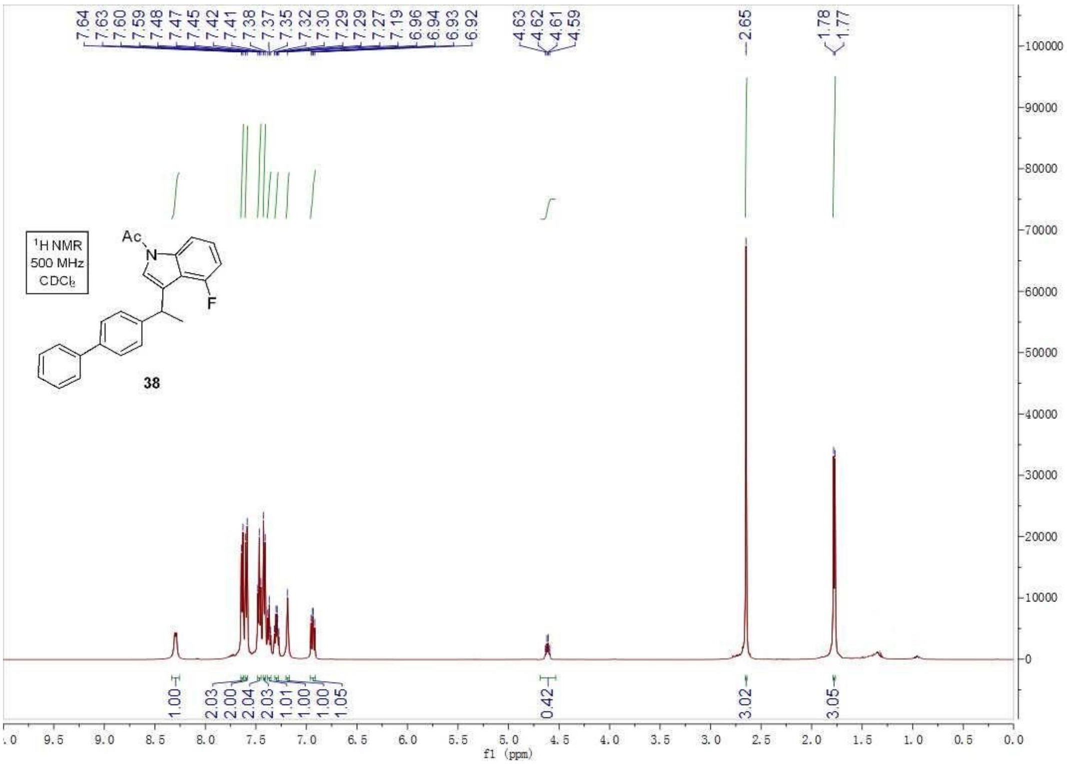

7.64
7.63
7.60
7.59
7.48
7.47
7.45
7.42
7.41
7.38
7.37
7.35
7.32
7.30
7.29
7.29
7.27
7.19
6.96
6.94
6.93
6.92
4.63
4.62
4.61
4.59
2.65
1.78
1.77
^{1}H NMR
500 MHz
$CDCl_3$
Ac
N
F
38
1.00
2.03
2.00
2.04
2.03
1.01
1.00
1.05
0.42
3.02
3.05
f1 (ppm)

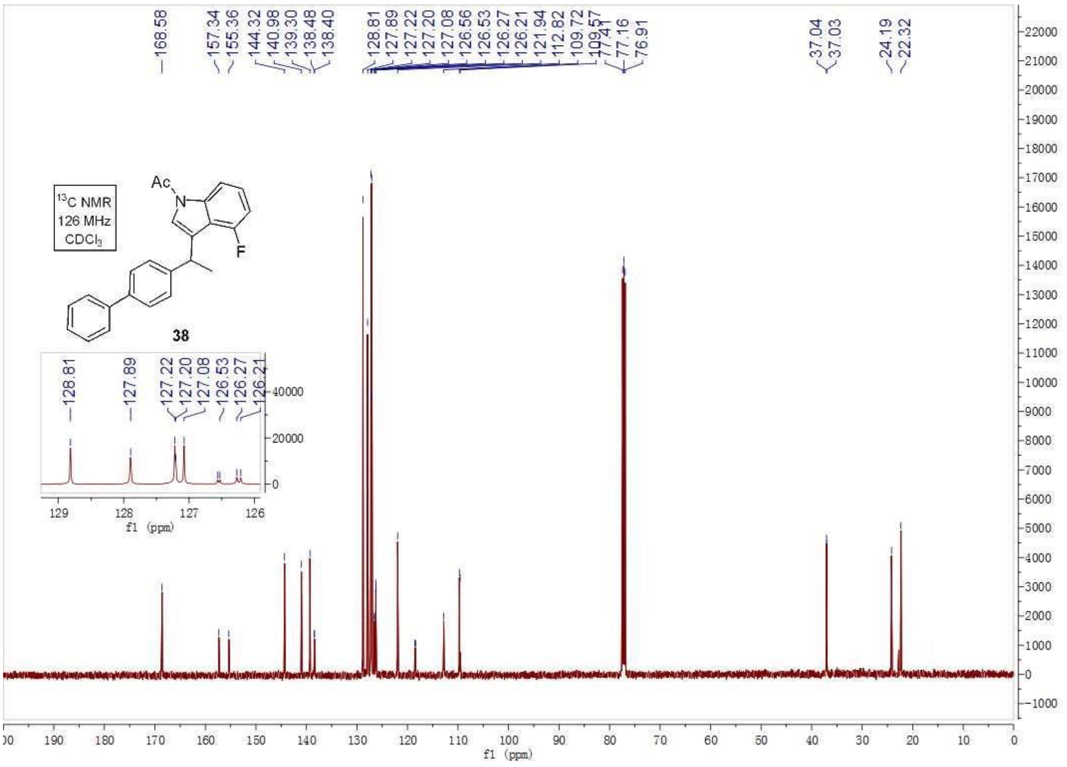

168.58
157.34
155.36
144.32
140.98
139.30
138.48
138.40
128.81
127.89
127.22
127.20
127.08
126.56
126.53
126.27
126.21
121.94
112.82
109.72
109.57
77.41
77.16
76.91
37.04
37.03
24.19
22.32
^{13}C NMR
126 MHz
$CDCl_3$
Ac
N
F
38
f1 (ppm)

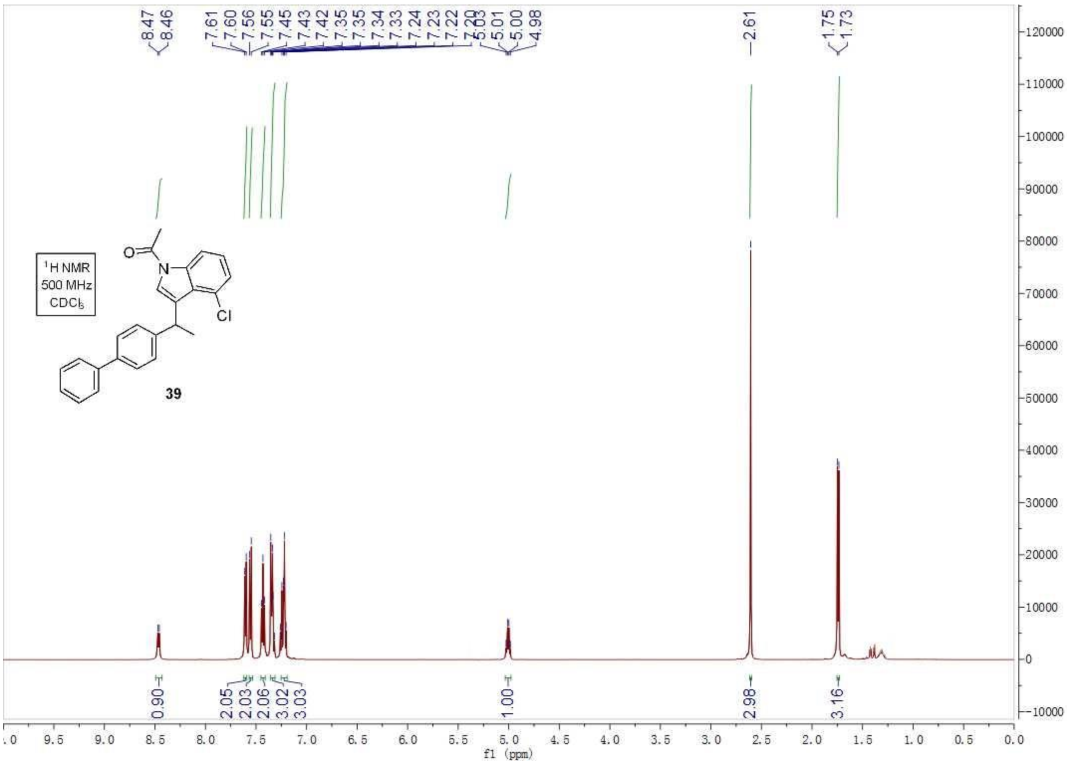

8.47
8.46
7.61
7.60
7.56
7.55
7.45
7.43
7.42
7.35
7.35
7.34
7.33
7.24
7.23
7.22
7.20
5.03
5.01
5.00
4.98
2.61
1.75
1.73
^{1}H NMR
500 MHz
CDCl$_3$
N
O
Cl
39
0.90
2.05
2.03
2.06
3.02
3.03
1.00
2.98
3.16
f1 (ppm)

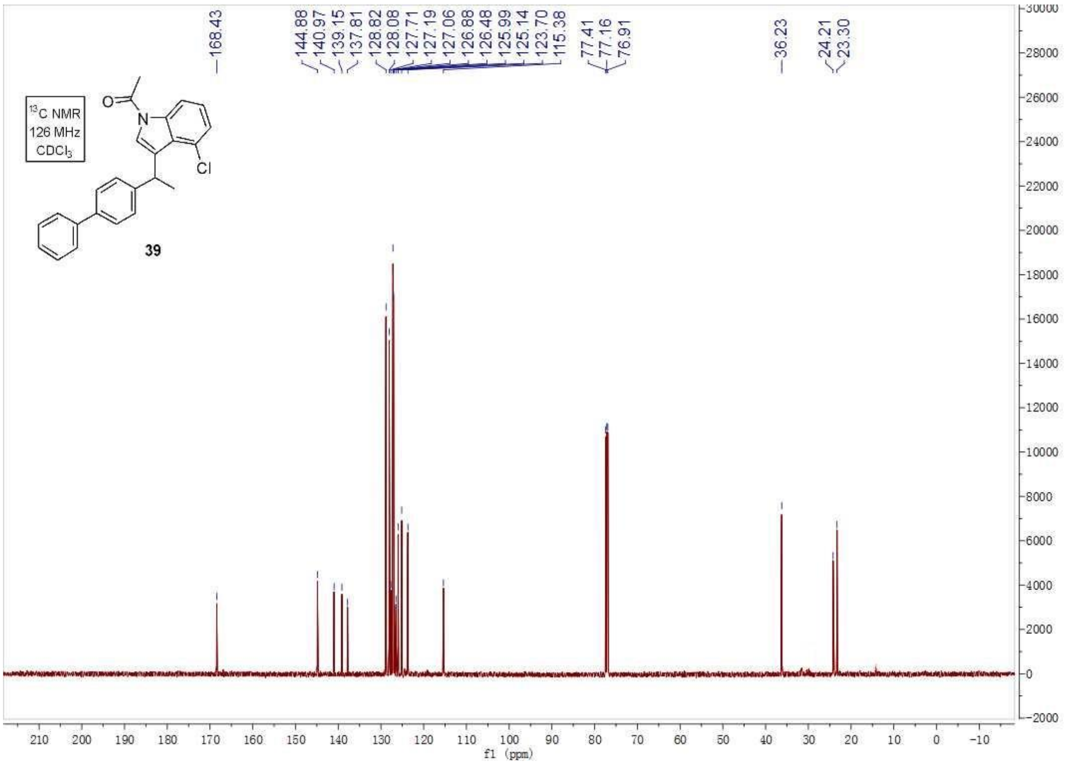

168.43
144.88
140.97
139.15
137.81
128.82
128.08
127.71
127.19
127.06
126.88
126.48
125.99
125.14
123.70
115.38
77.41
77.16
76.91
36.23
24.21
23.30
^{13}C NMR
126 MHz
CDCl$_3$
N
O
Cl
39
f1 (ppm)

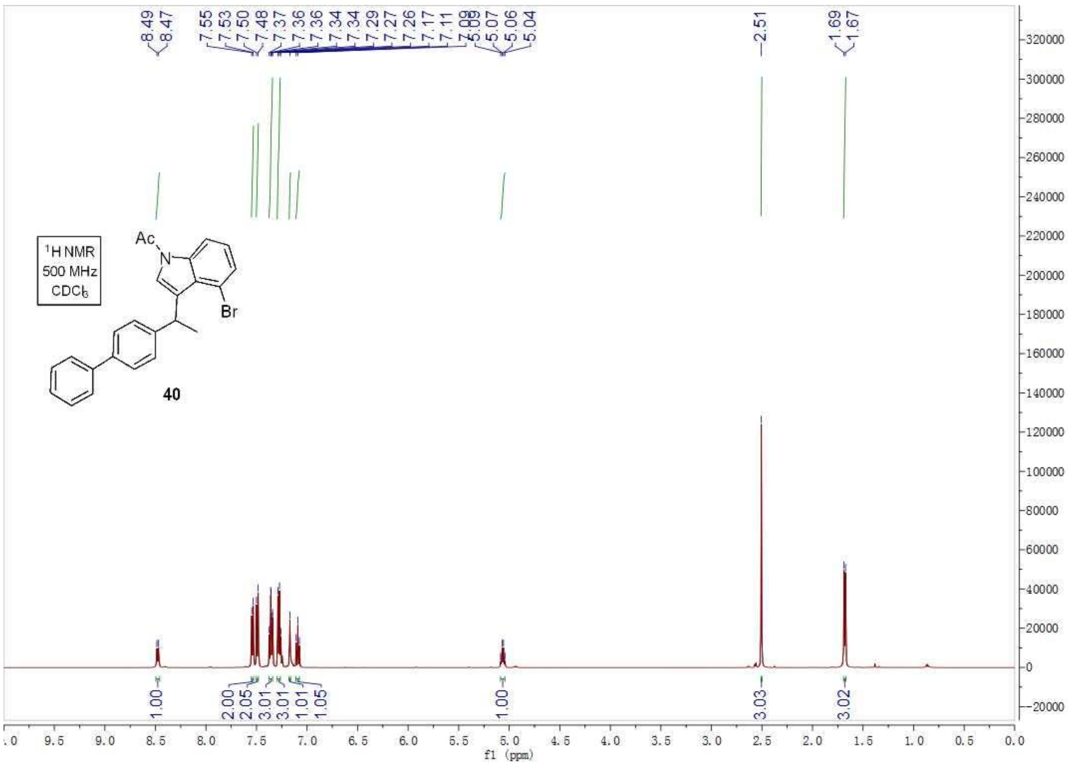

8.49
8.47
7.55
7.53
7.50
7.48
7.37
7.36
7.34
7.34
7.29
7.27
7.26
7.17
7.11
5.07
5.06
5.04
2.51
1.69
1.67
^{1}H NMR
500 MHz
$CDCl_3$
Ac
N
Br
40
1.00
2.00
2.05
3.01
3.01
1.01
1.05
1.00
3.03
3.02
f1 (ppm)

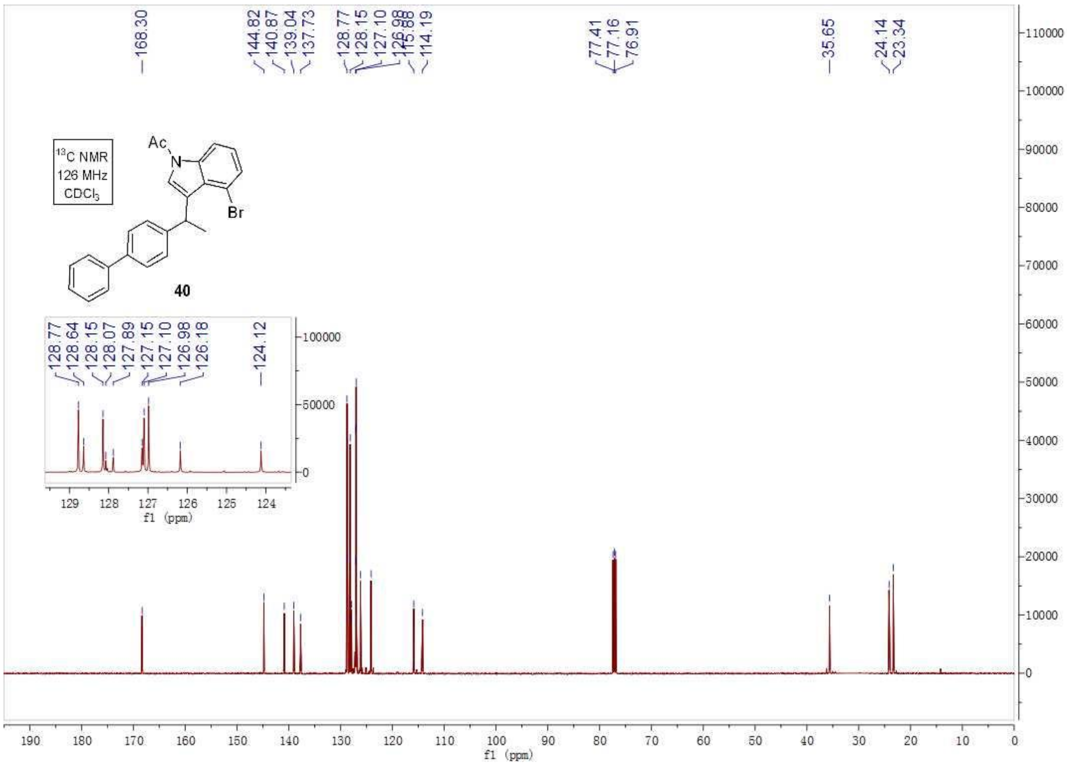

168.30
144.82
140.87
139.04
137.73
128.77
128.15
127.10
114.19
77.41
77.16
76.91
35.65
24.14
23.34
^{13}C NMR
126 MHz
$CDCl_3$
Ac
N
Br
40
128.77
128.64
128.15
128.07
127.89
127.15
127.10
126.98
126.18
124.12
f1 (ppm)

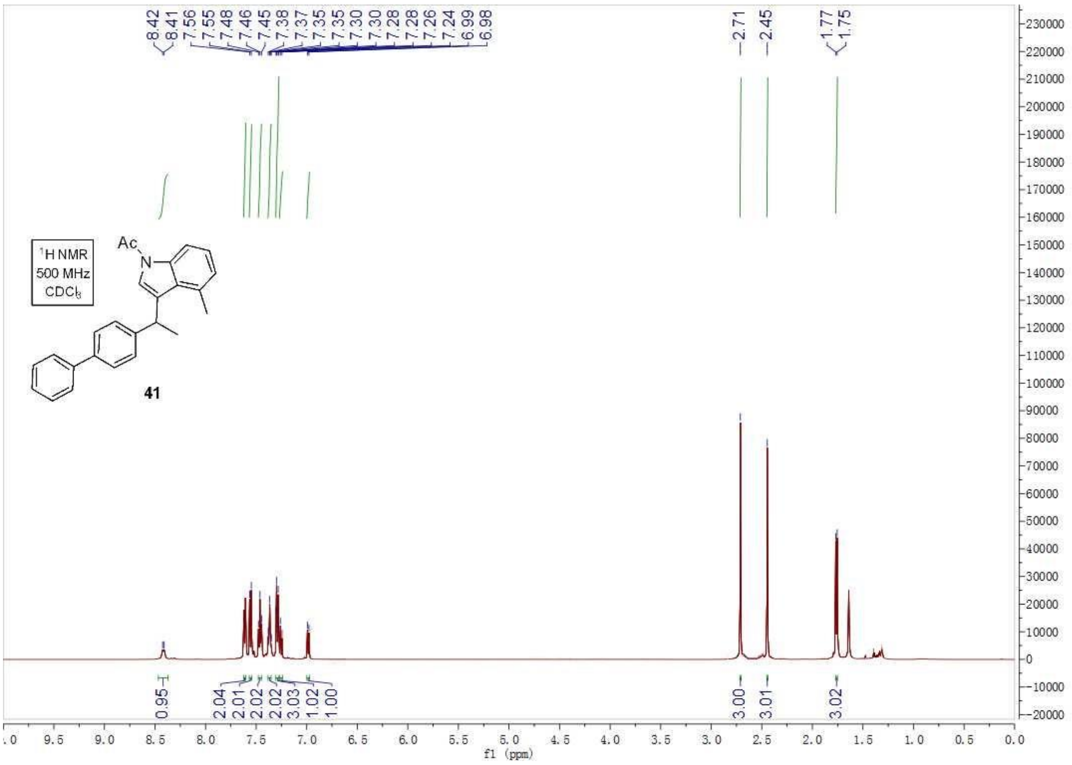

1H NMR
500 MHz
CDCl3
Ac
N
41

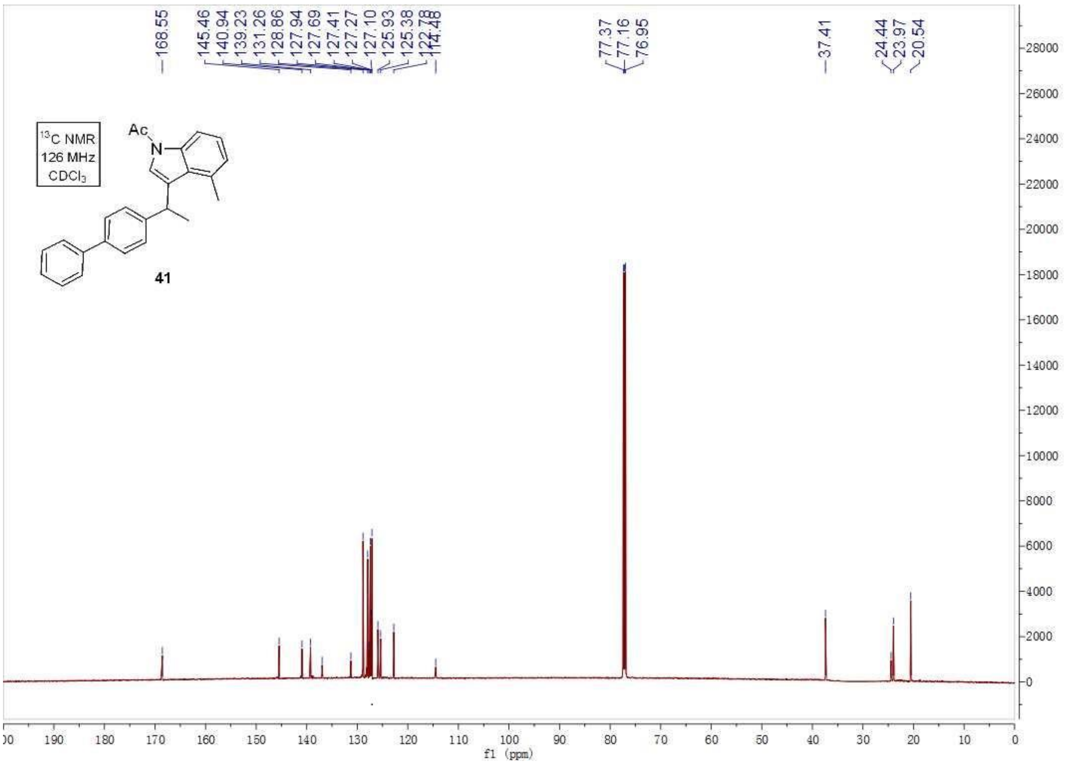

13C NMR
126 MHz
CDCl3
Ac
N
41

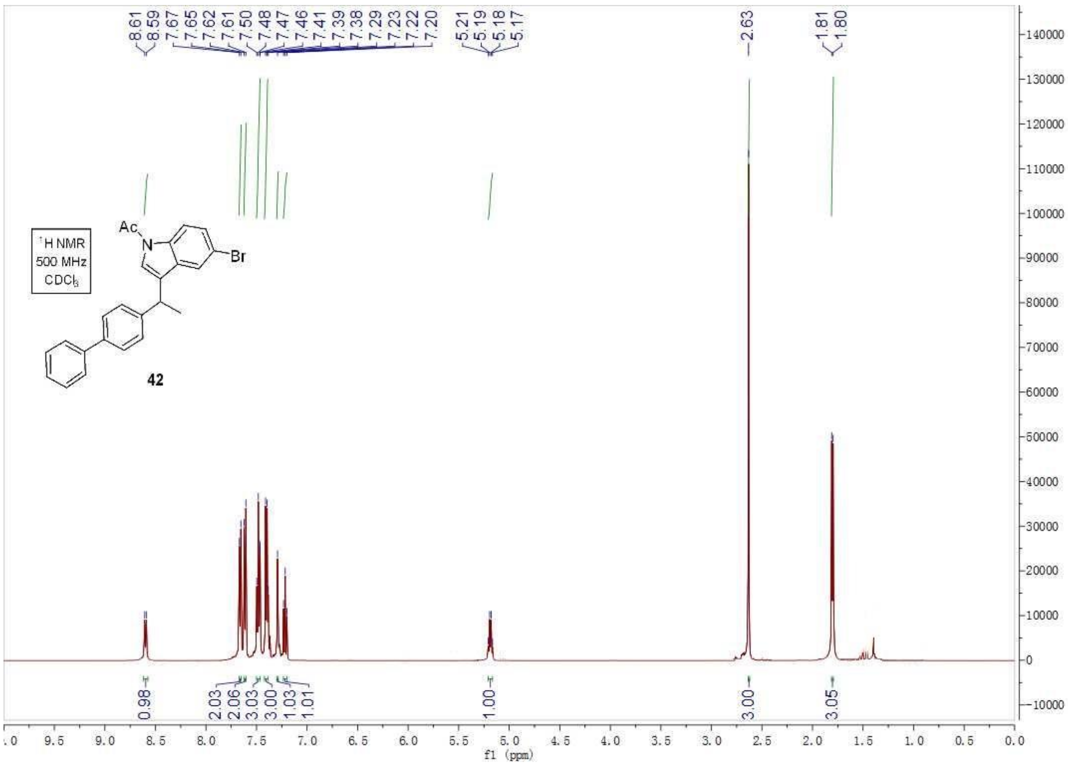

8.61
8.59
7.67
7.65
7.62
7.61
7.50
7.48
7.47
7.46
7.41
7.39
7.38
7.29
7.23
7.22
7.20
5.21
5.19
5.18
5.17
2.63
1.81
1.80
^{1}H NMR
500 MHz
CDCl$_3$
Ac
N
Br
42
0.98
2.03
2.06
3.03
3.00
1.03
1.01
1.00
3.00
3.05
f1 (ppm)

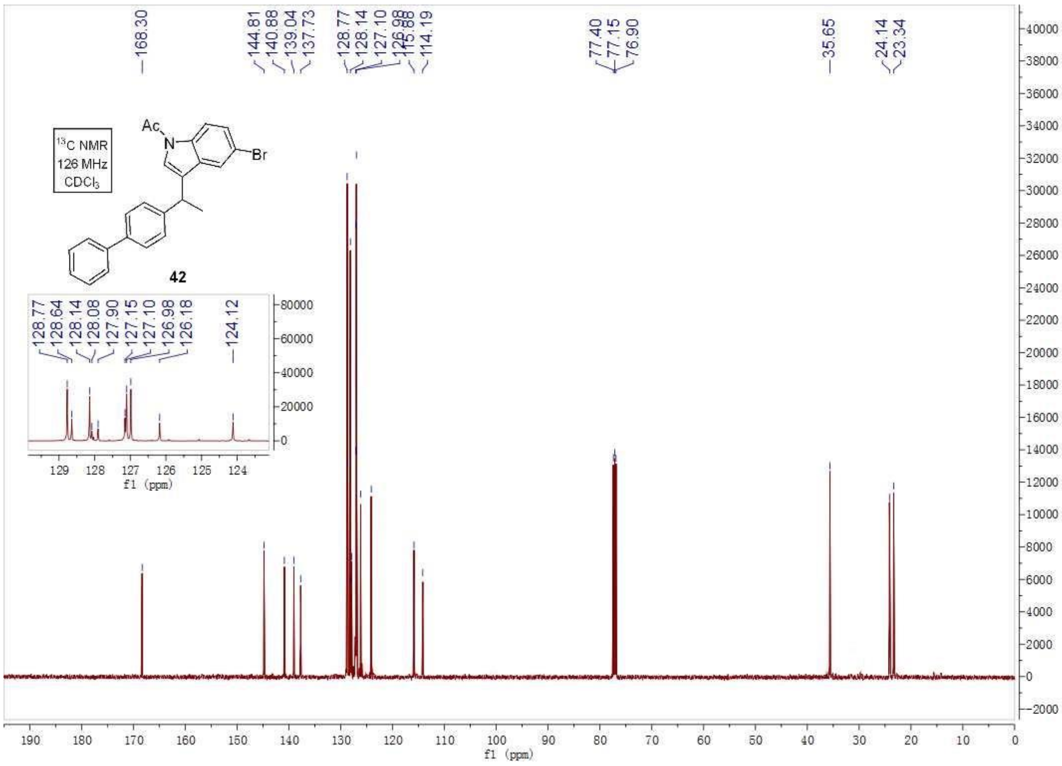

168.30
144.81
140.88
139.04
137.73
128.77
128.14
127.10
114.19
77.40
77.15
76.90
35.65
24.14
23.34
^{13}C NMR
126 MHz
CDCl$_3$
Ac
N
Br
42
128.77
128.64
128.14
128.08
127.90
127.15
127.10
126.98
126.18
124.12
f1 (ppm)

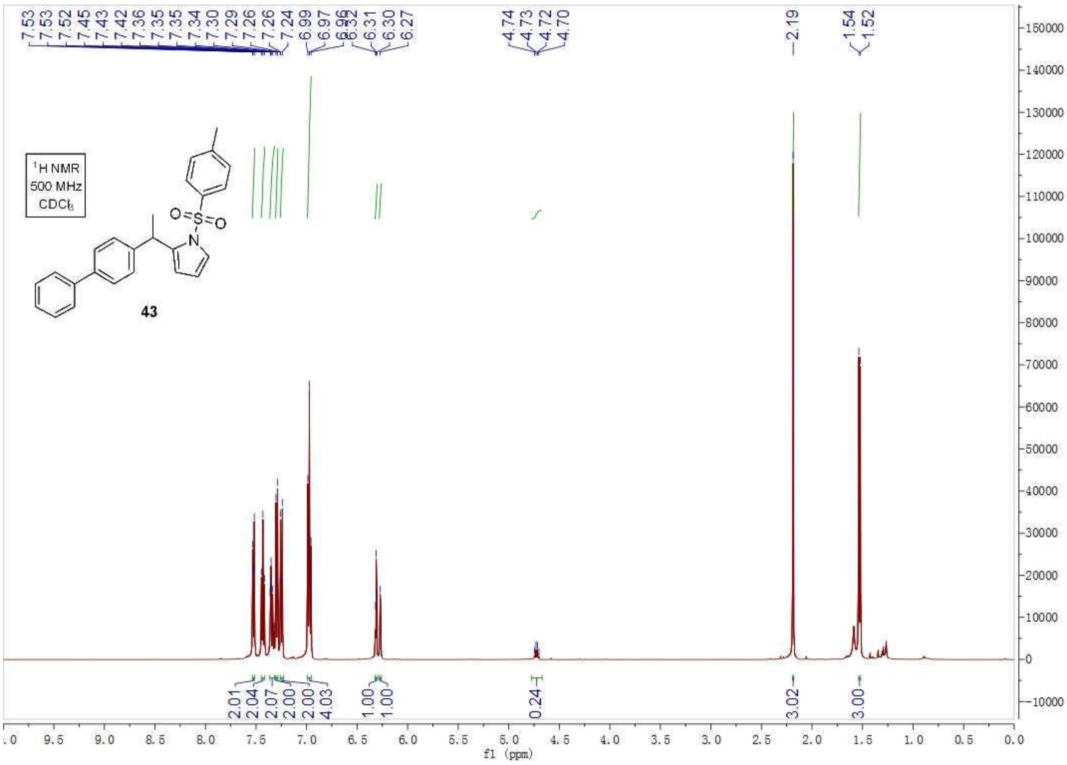

7.53 7.53 7.52 7.45 7.43 7.42 7.36 7.35 7.34 7.30 7.29 7.26 7.26 7.24 6.99 6.97
6.31 6.30 6.27
4.74 4.73 4.72 4.70
2.19
1.54 1.52
^{1}H NMR
500 MHz
$CDCl_3$
43
2.01 2.04 2.07 2.00 2.00 4.03 1.00 1.00 0.24 3.02 3.00
f1 (ppm)

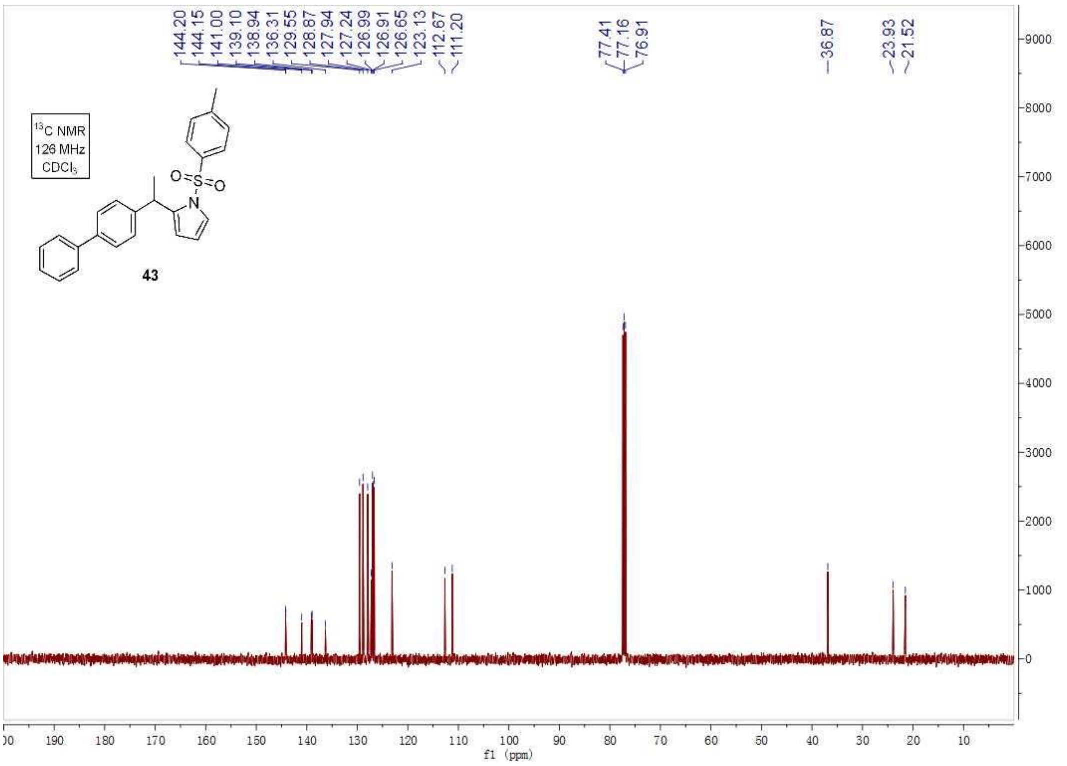

144.20 144.15 141.00 139.10 138.94 136.31 129.55 128.87 127.94 127.24 126.99 126.91 126.65 123.13 112.67 111.20
77.41 77.16 76.91
36.87
23.93 21.52
^{13}C NMR
126 MHz
$CDCl_3$
43
f1 (ppm)

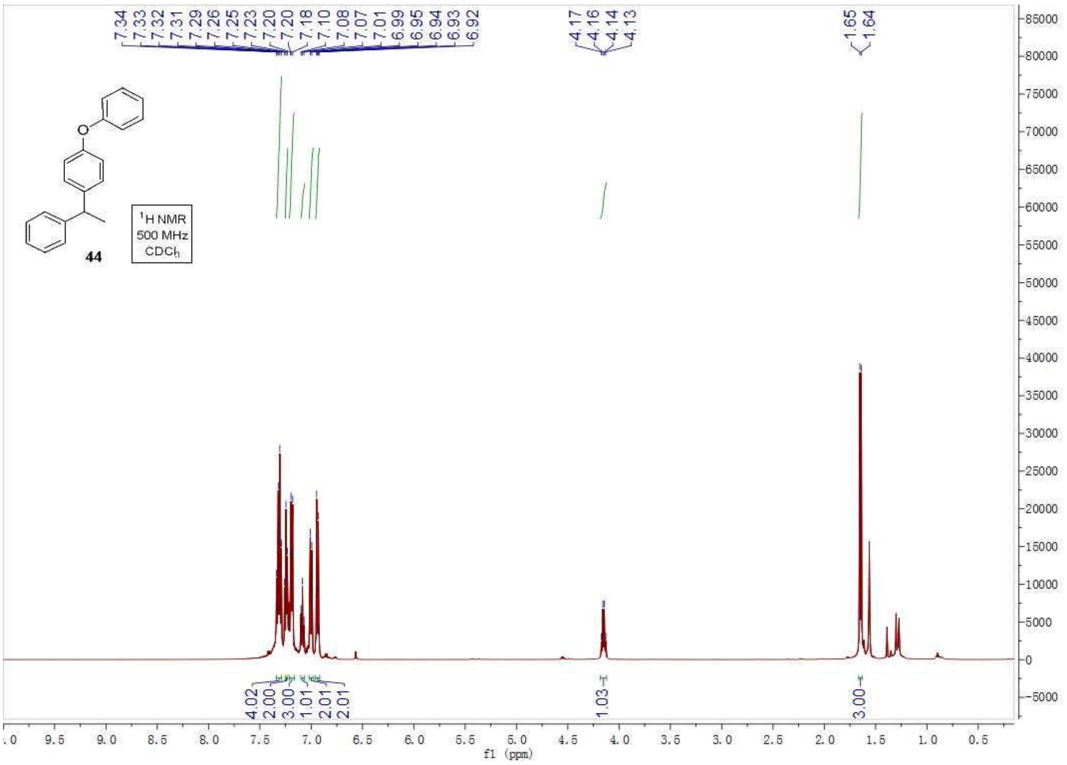

7.34
7.33
7.32
7.31
7.29
7.26
7.25
7.23
7.20
7.20
7.18
7.10
7.08
7.07
7.01
6.99
6.95
6.94
6.93
6.92
4.17
4.16
4.14
4.13
1.65
1.64
1H NMR
500 MHz
CDCl3
44
4.02
2.00
3.00
1.01
2.01
2.01
1.03
3.00
f1 (ppm)

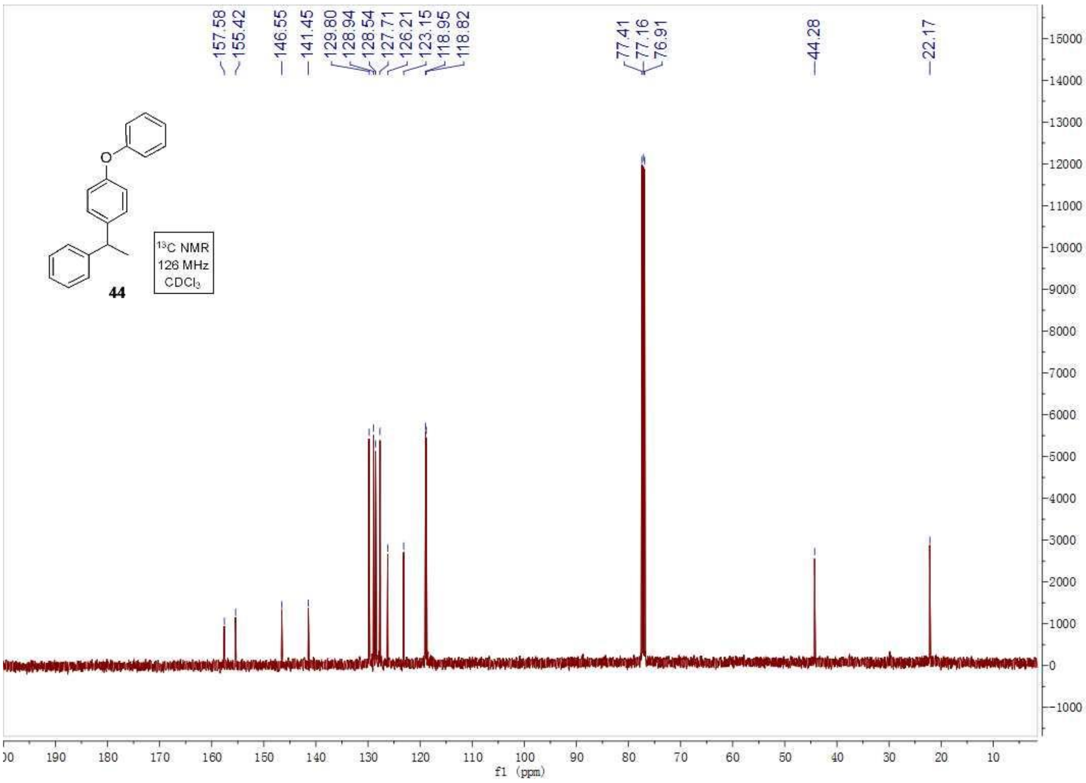

157.58
155.42
146.55
141.45
129.80
128.94
128.54
127.71
126.21
123.15
118.95
118.82
77.41
77.16
76.91
44.28
22.17
13C NMR
126 MHz
CDCl3
44
f1 (ppm)

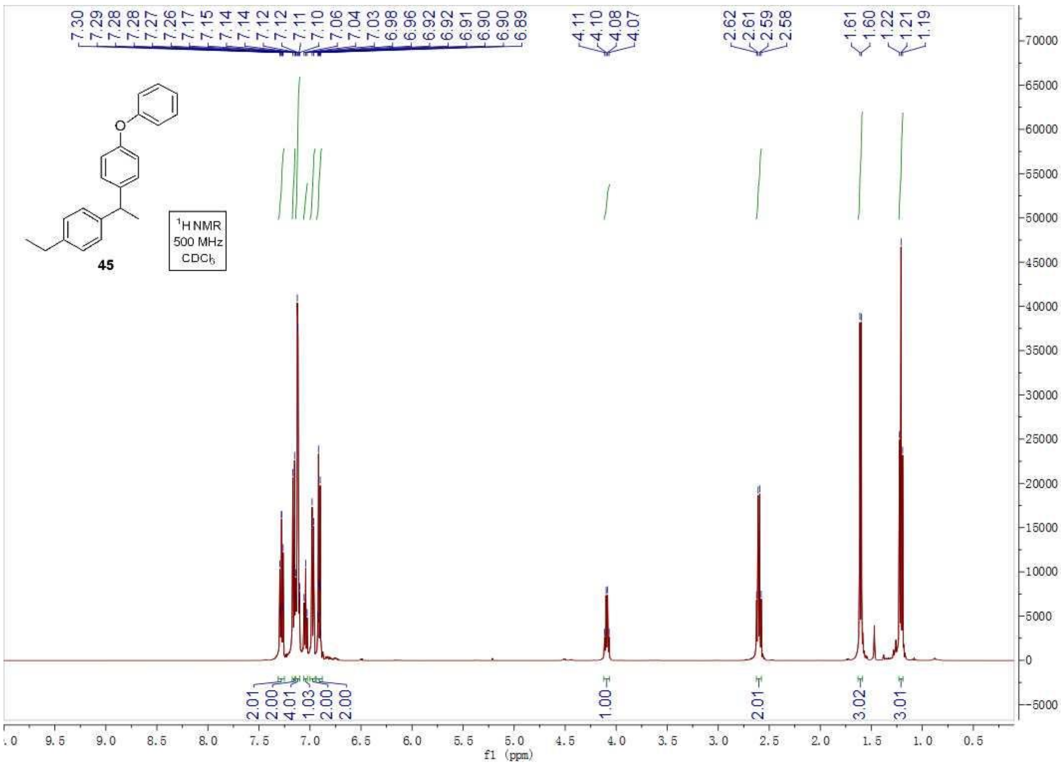
1H NMR
500 MHz
CDCl3
45

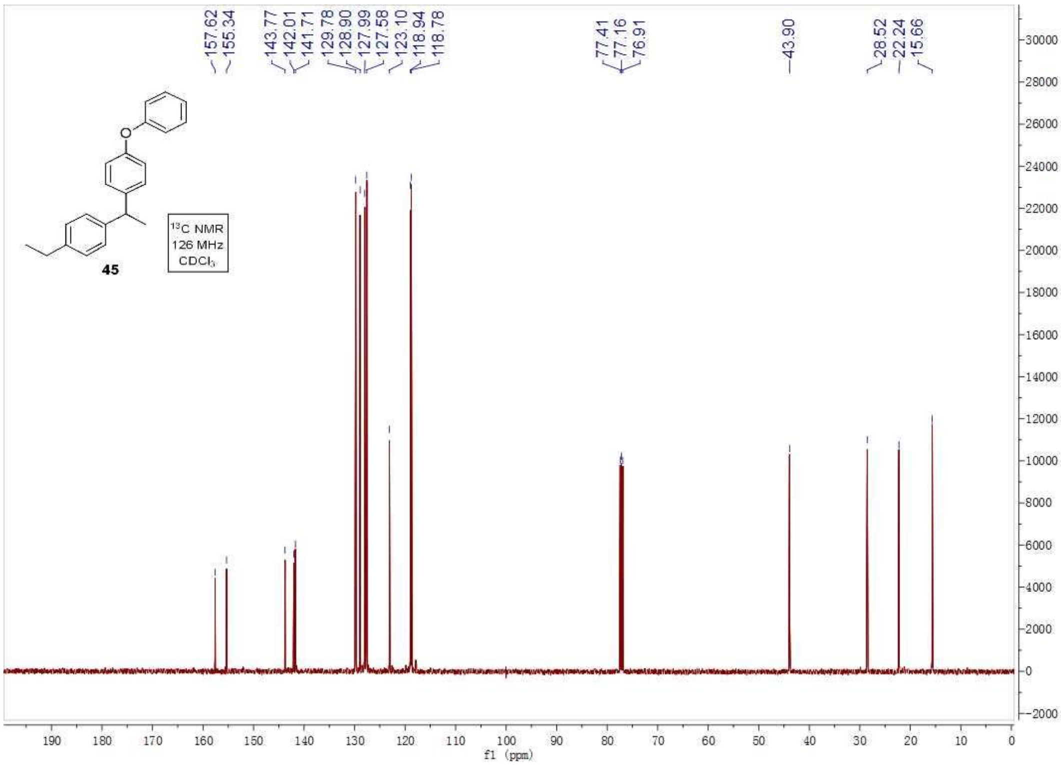
13C NMR
126 MHz
CDCl3
45

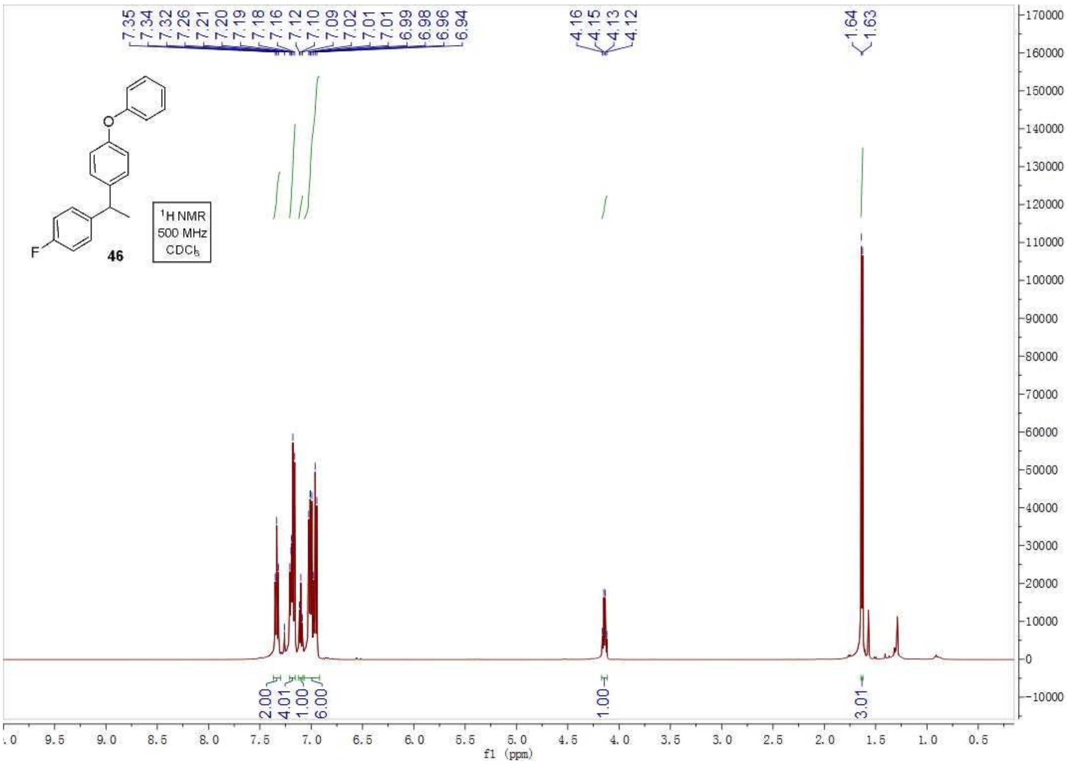

7.35
7.34
7.32
7.26
7.21
7.20
7.19
7.18
7.16
7.12
7.10
7.09
7.02
7.01
6.99
6.98
6.96
6.94
4.16
4.15
4.13
4.12
1.64
1.63
^{1}H NMR
500 MHz
$CDCl_3$
F
O
46
2.00
4.01
1.00
6.00
1.00
3.01
f1 (ppm)

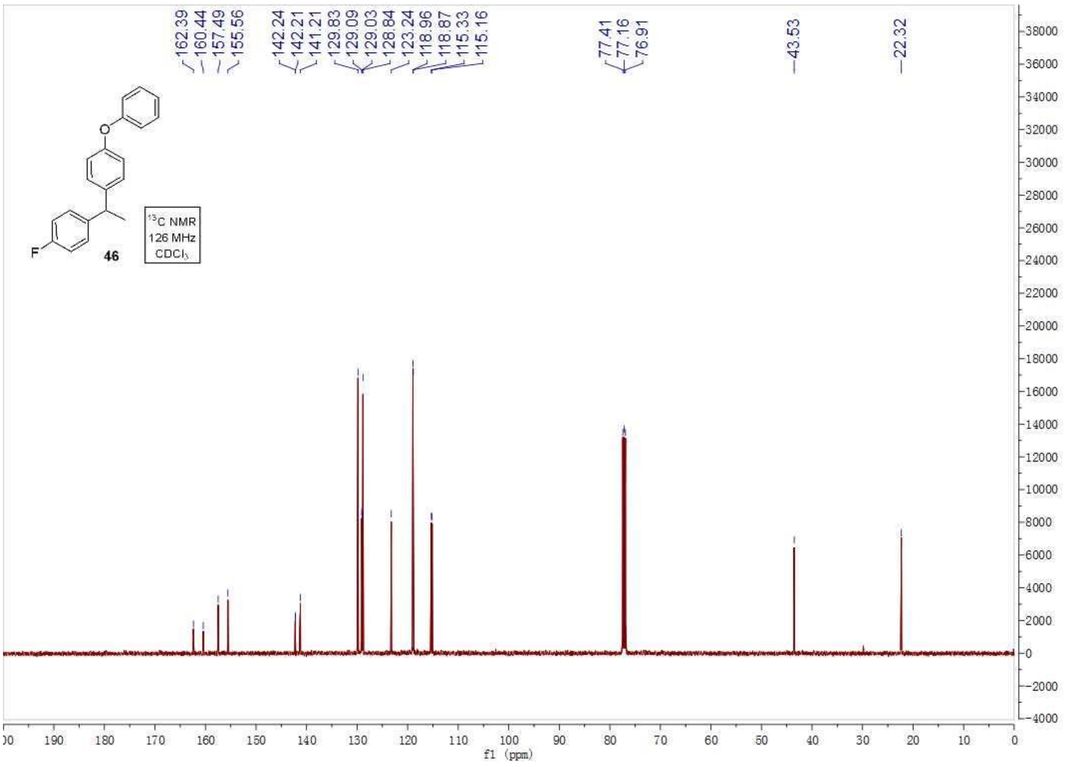

162.39
160.44
157.49
155.56
142.24
142.21
141.21
129.83
129.09
129.03
128.84
123.24
118.96
118.87
115.33
115.16
77.41
77.16
76.91
43.53
22.32
^{13}C NMR
126 MHz
$CDCl_3$
F
O
46
f1 (ppm)

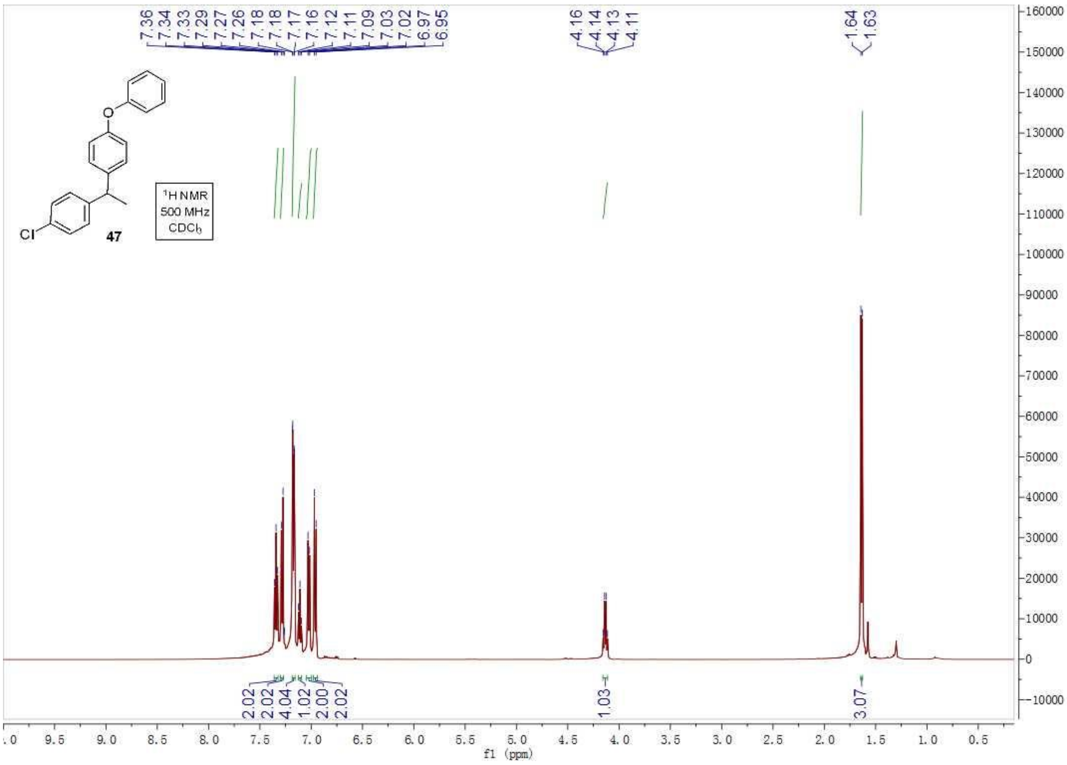
7.36
7.34
7.33
7.29
7.27
7.26
7.18
7.18
7.17
7.16
7.12
7.11
7.09
7.03
7.02
6.97
6.95
4.16
4.14
4.13
4.11
1.64
1.63
Cl
O
47
^{1}H NMR
500 MHz
$CDCl_3$
2.02
2.02
4.04
1.02
2.00
2.02
1.03
3.07
f1 (ppm)

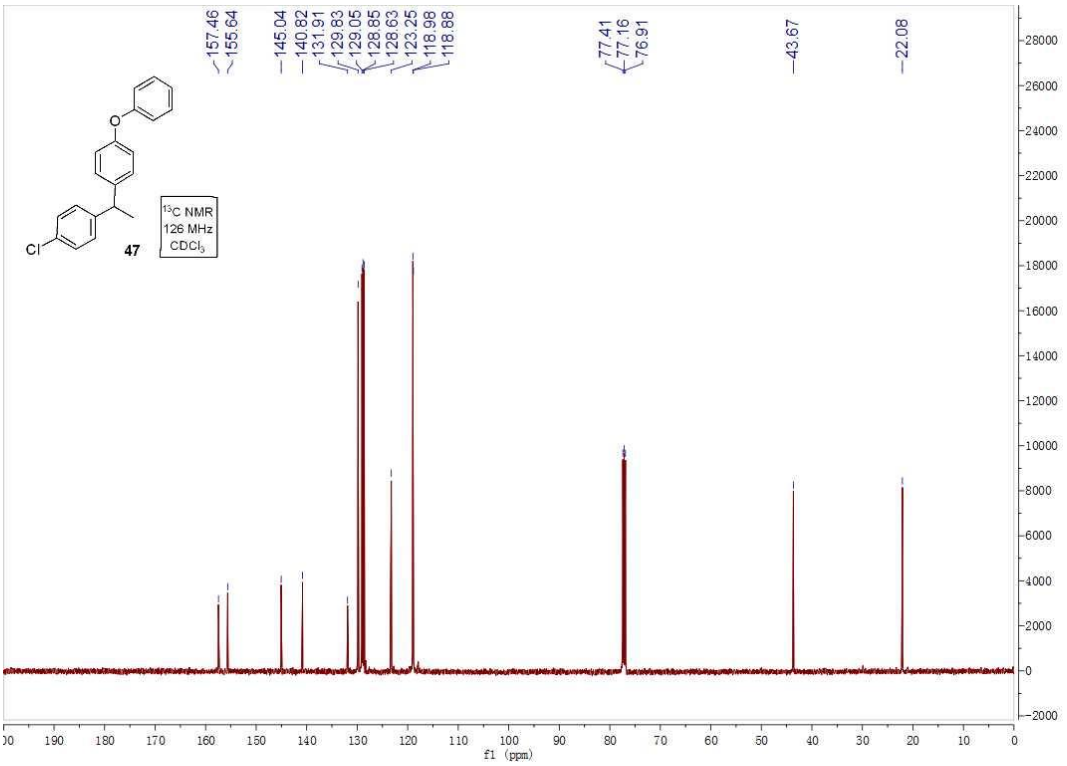
157.46
155.64
145.04
140.82
131.91
129.83
129.05
128.85
128.63
123.25
118.98
118.88
77.41
77.16
76.91
43.67
22.08
Cl
O
47
^{13}C NMR
126 MHz
$CDCl_3$
f1 (ppm)

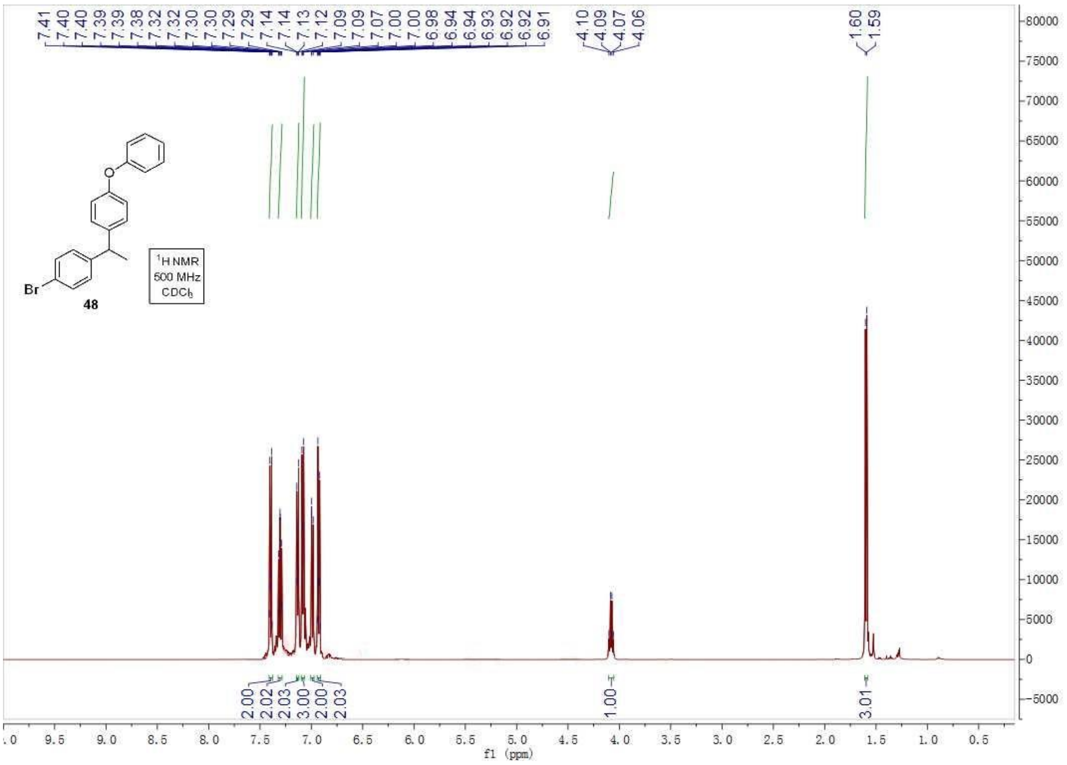
1H NMR
500 MHz
CDCl3
48
f1 (ppm)

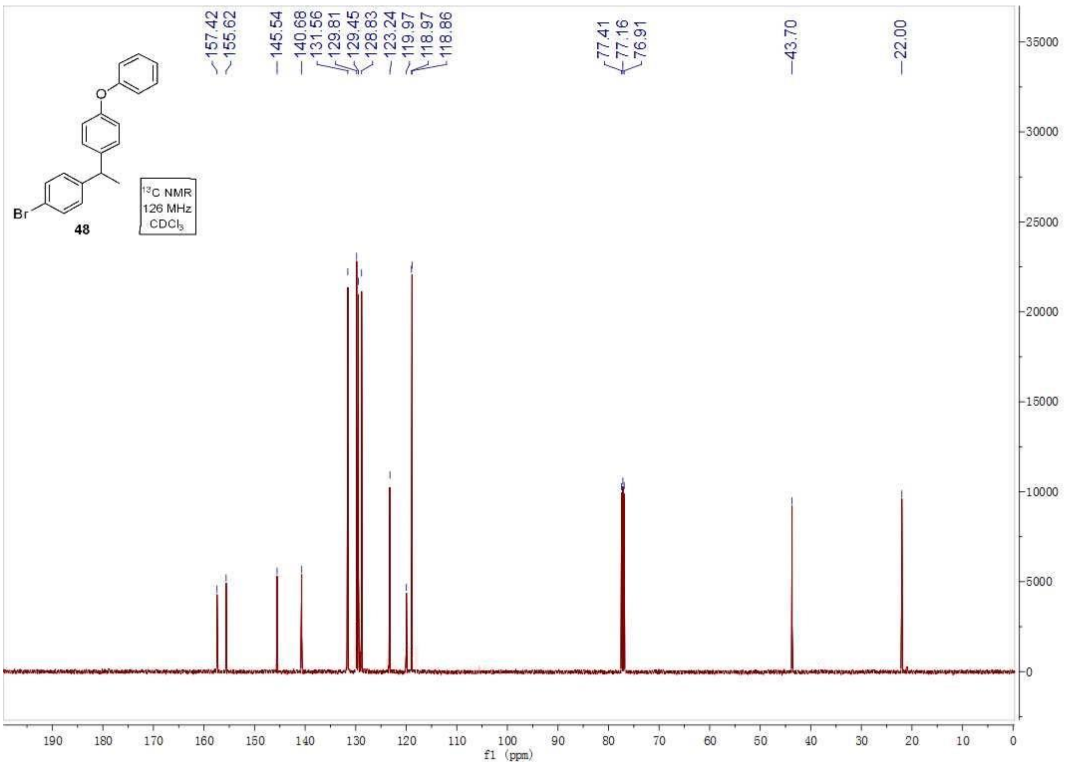
13C NMR
126 MHz
CDCl3
48
f1 (ppm)

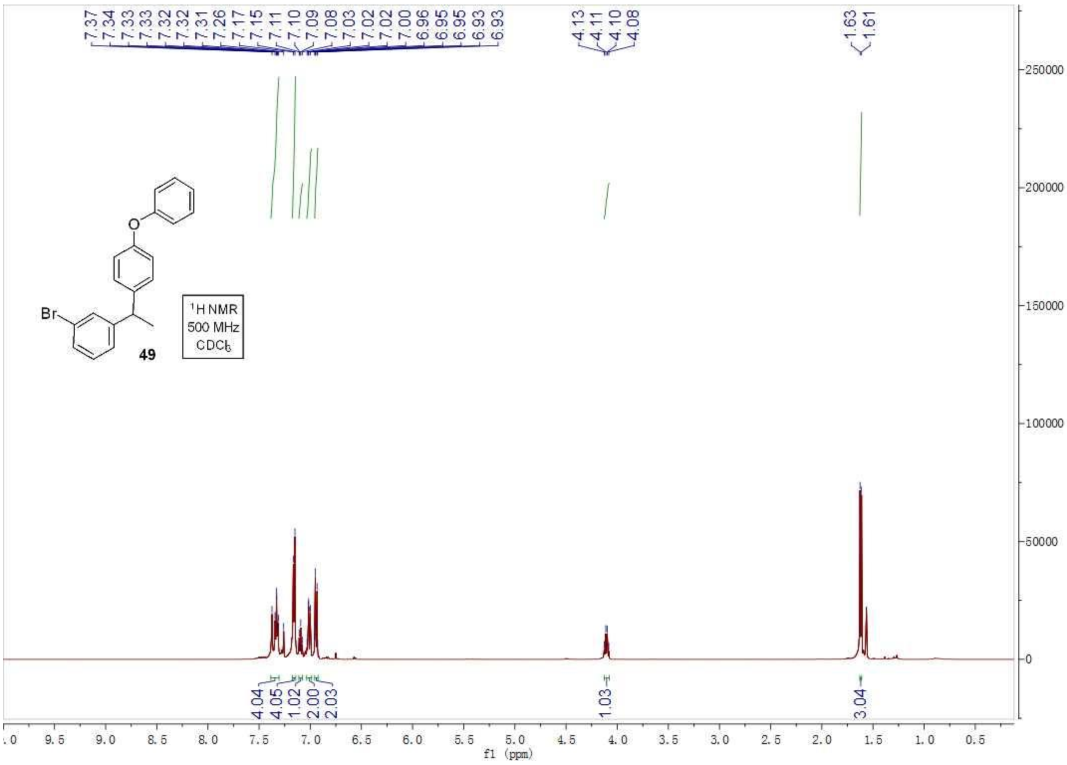
7.37 7.34 7.33 7.33 7.32 7.32 7.31 7.26 7.17 7.15 7.11 7.10 7.09 7.08 7.03 7.02 7.02 7.00 6.96 6.95 6.95 6.93 6.93
4.13 4.11 4.10 4.08
1.63 1.61
Br
O
49
^{1}H NMR
500 MHz
CDCl$_3$
4.04 4.05 1.02 2.00 2.03
1.03
3.04
f1 (ppm)

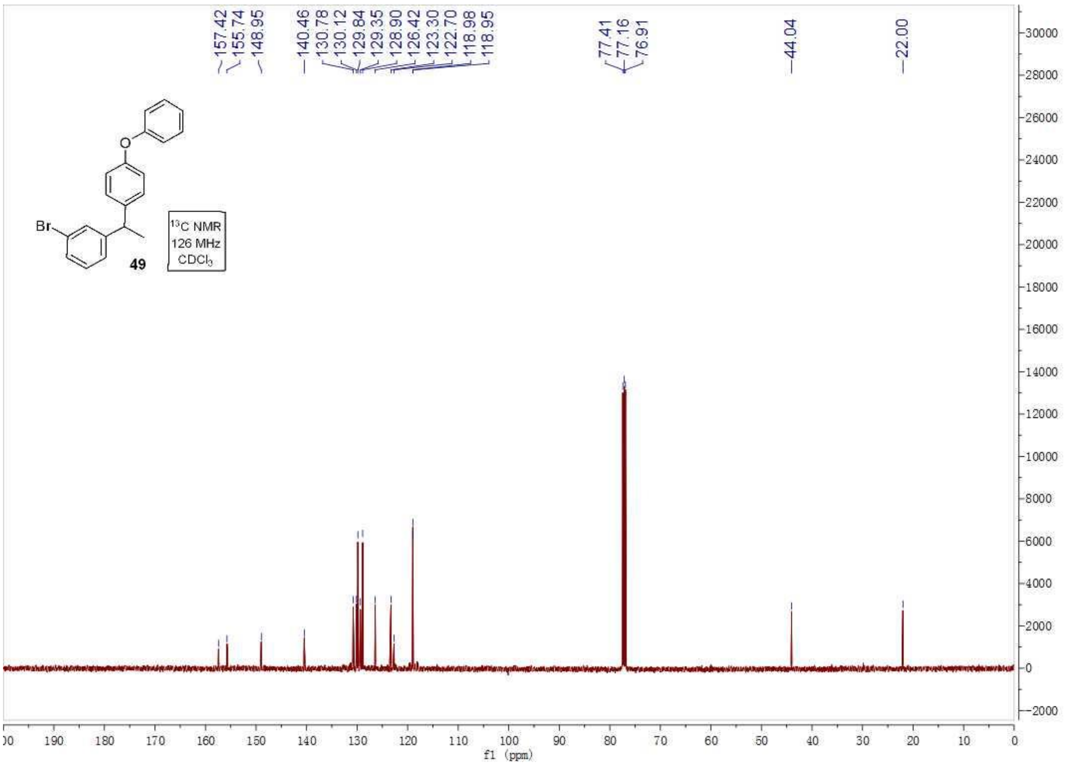
157.42 155.74 148.95
140.46 130.78 130.12 129.84 129.35 128.90 126.42 123.30 122.70 118.98 118.95
77.41 77.16 76.91
44.04
22.00
Br
O
49
^{13}C NMR
126 MHz
CDCl$_3$
f1 (ppm)

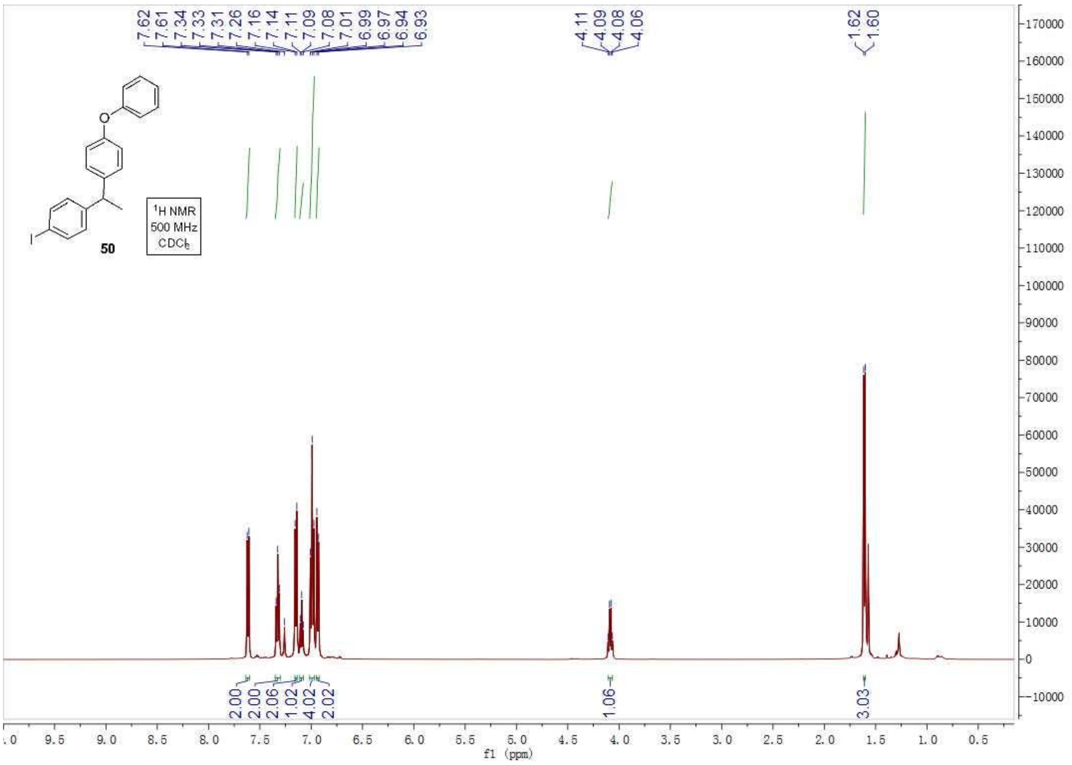

7.62
7.61
7.34
7.33
7.31
7.26
7.16
7.14
7.11
7.09
7.08
7.01
6.99
6.97
6.94
6.93
4.11
4.09
4.08
4.06
1.62
1.60
1H NMR
500 MHz
CDCl3
50
2.00
2.00
2.06
1.02
4.02
2.02
1.06
3.03
f1 (ppm)

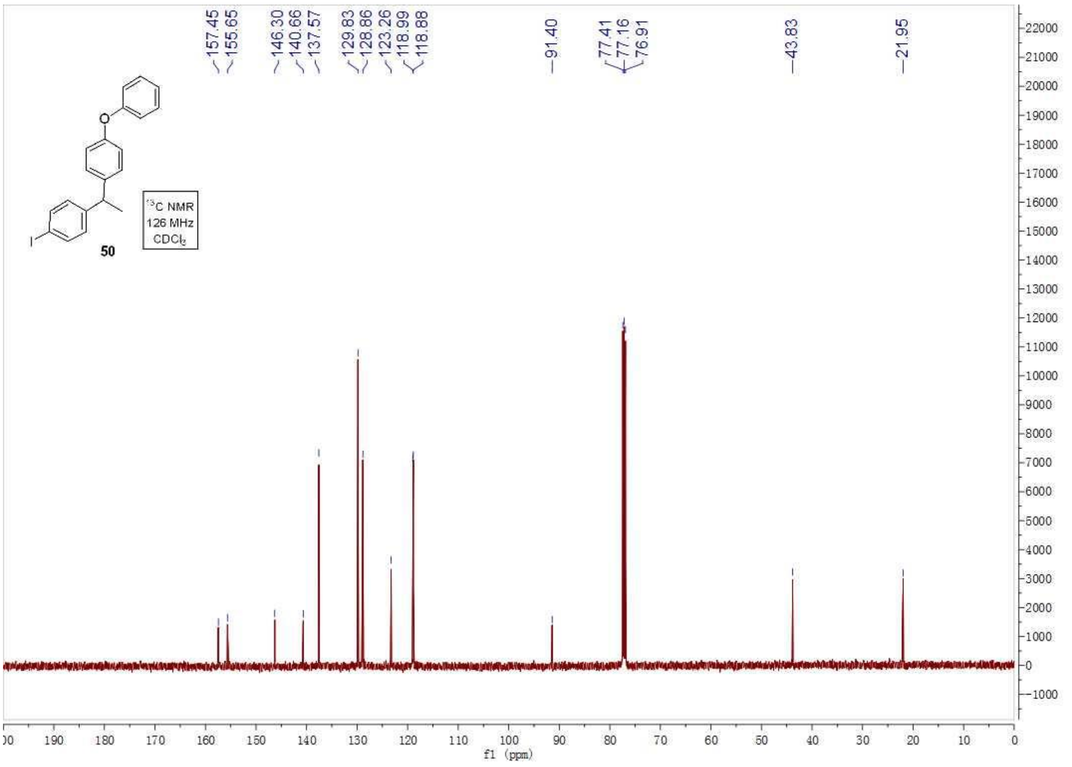

157.45
155.65
146.30
140.66
137.57
129.83
128.86
123.26
118.99
118.88
91.40
77.41
77.16
76.91
43.83
21.95
13C NMR
126 MHz
CDCl3
50
f1 (ppm)

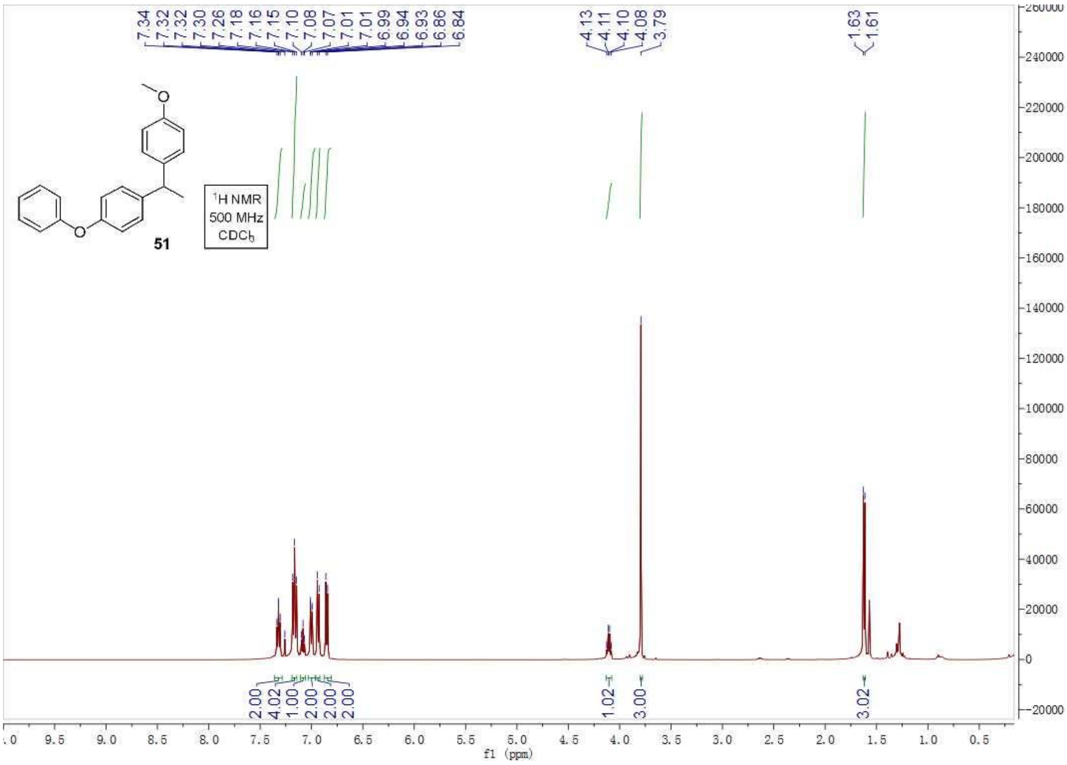

51
1H NMR
500 MHz
CDCl3
7.34 7.32 7.32 7.30 7.26 7.18 7.16 7.15 7.10 7.08 7.07 7.01 6.99 6.94 6.93 6.86 6.84
4.13 4.11 4.10 4.08 3.79
1.63 1.61
2.00 4.02 1.00 2.00 2.00 2.00
1.02 3.00
3.02
f1 (ppm)

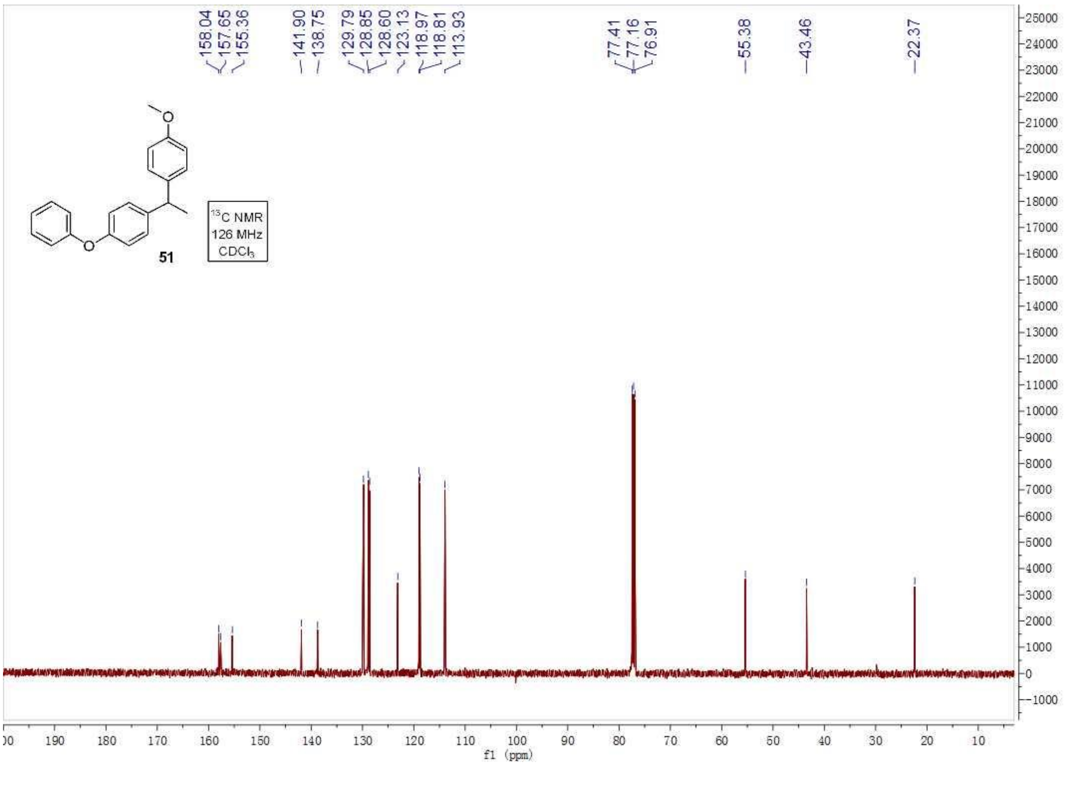

51
13C NMR
126 MHz
CDCl3
158.04 157.65 155.36
141.90 138.75
129.79 128.85 128.60 123.13 118.97 118.81 113.93
77.41 77.16 76.91
55.38
43.46
22.37
f1 (ppm)

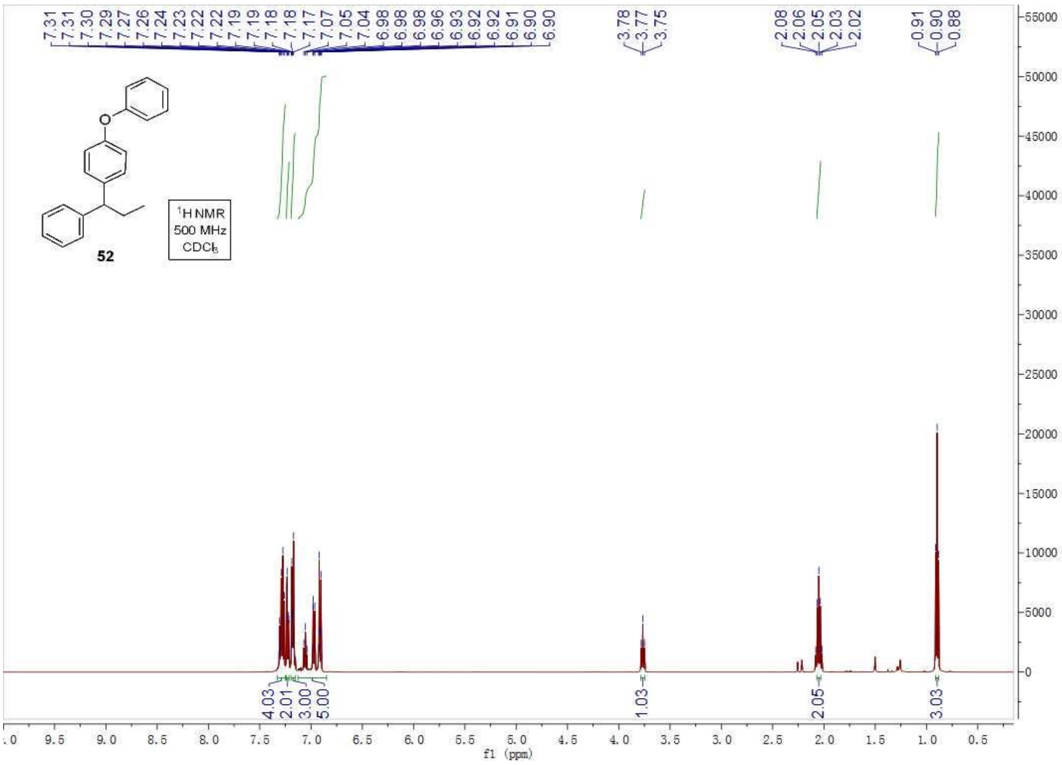

7.31 7.31 7.30 7.29 7.27 7.26 7.24 7.23 7.22 7.22 7.22 7.19 7.18 7.18 7.17 7.07 7.05 7.04 6.98 6.98 6.96 6.93 6.92 6.92 6.91 6.90 6.90
3.78 3.77 3.75
2.08 2.06 2.05 2.03 2.02
0.91 0.90 0.88
1H NMR
500 MHz
CDCl3
52
4.03 2.01 3.00 5.00
1.03
2.05
3.03
f1 (ppm)

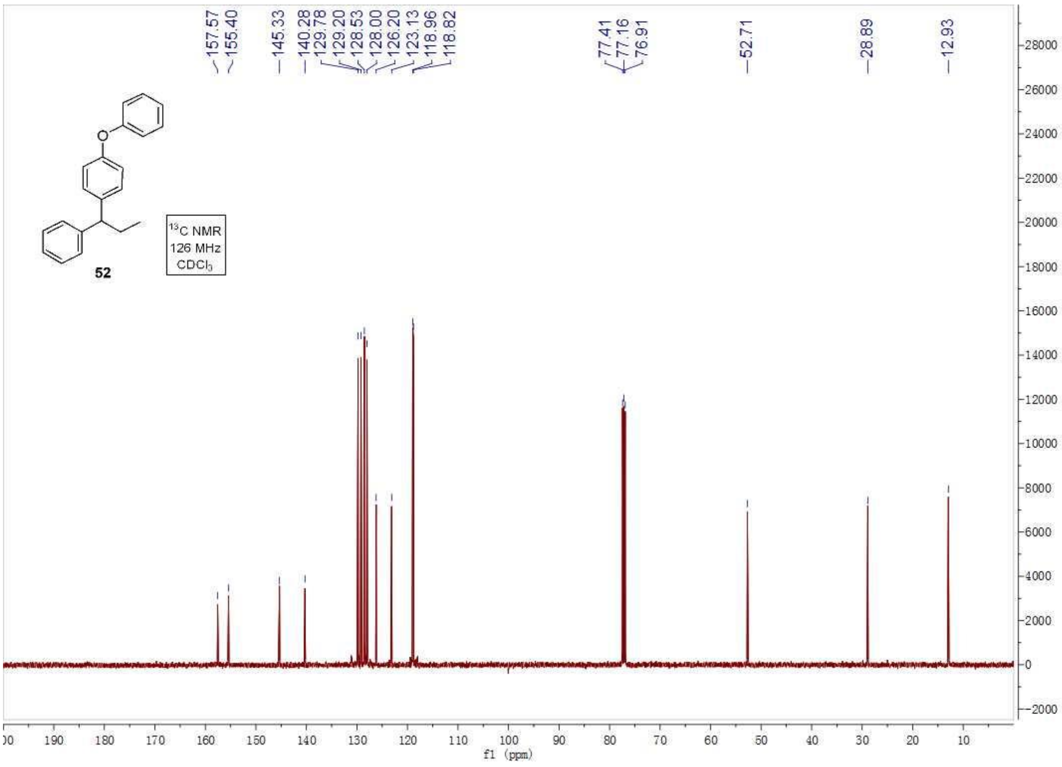

157.57 155.40
145.33
140.28 129.78 129.20 128.53 128.00 126.20 123.13 118.96 118.82
77.41 77.16 76.91
52.71
28.89
12.93
13C NMR
126 MHz
CDCl3
52
f1 (ppm)

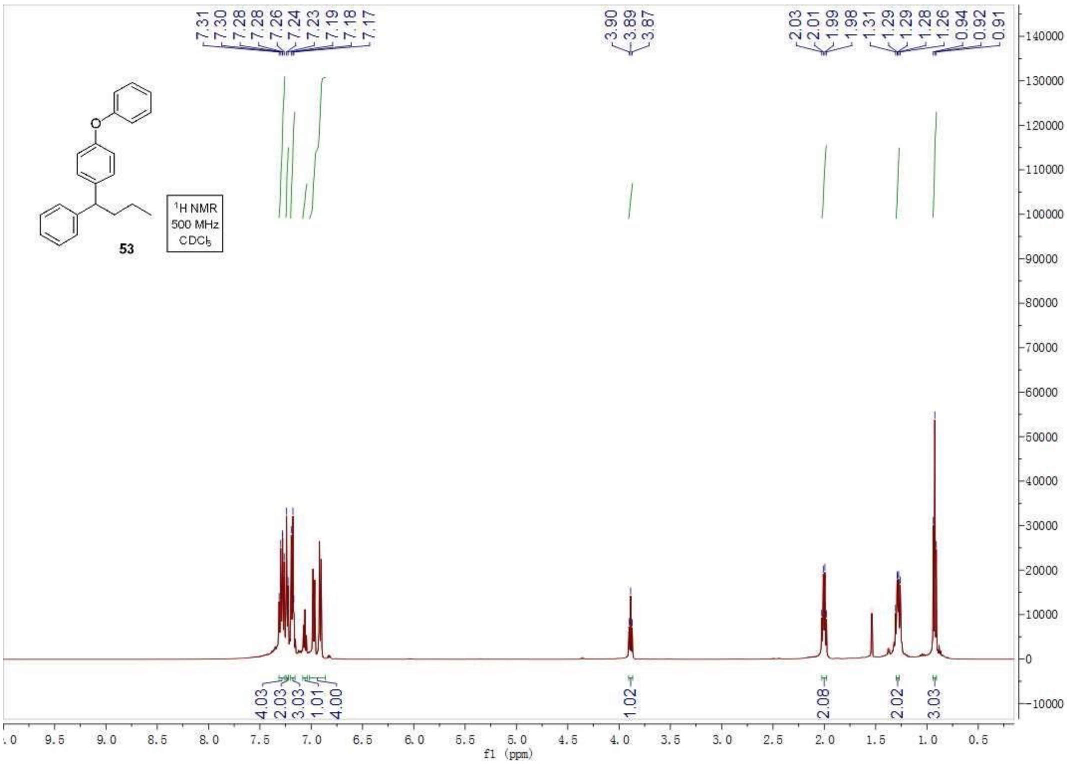
7.31 7.30 7.28 7.28 7.26 7.24 7.23 7.19 7.18 7.17
3.90 3.89 3.87
2.03 2.01 1.99 1.98 1.31 1.29 1.29 1.28 1.26 0.94 0.92 0.91
O
53
1H NMR
500 MHz
CDCl3
4.03 2.03 3.03 1.01 4.00
1.02
2.08
2.02
3.03
f1 (ppm)

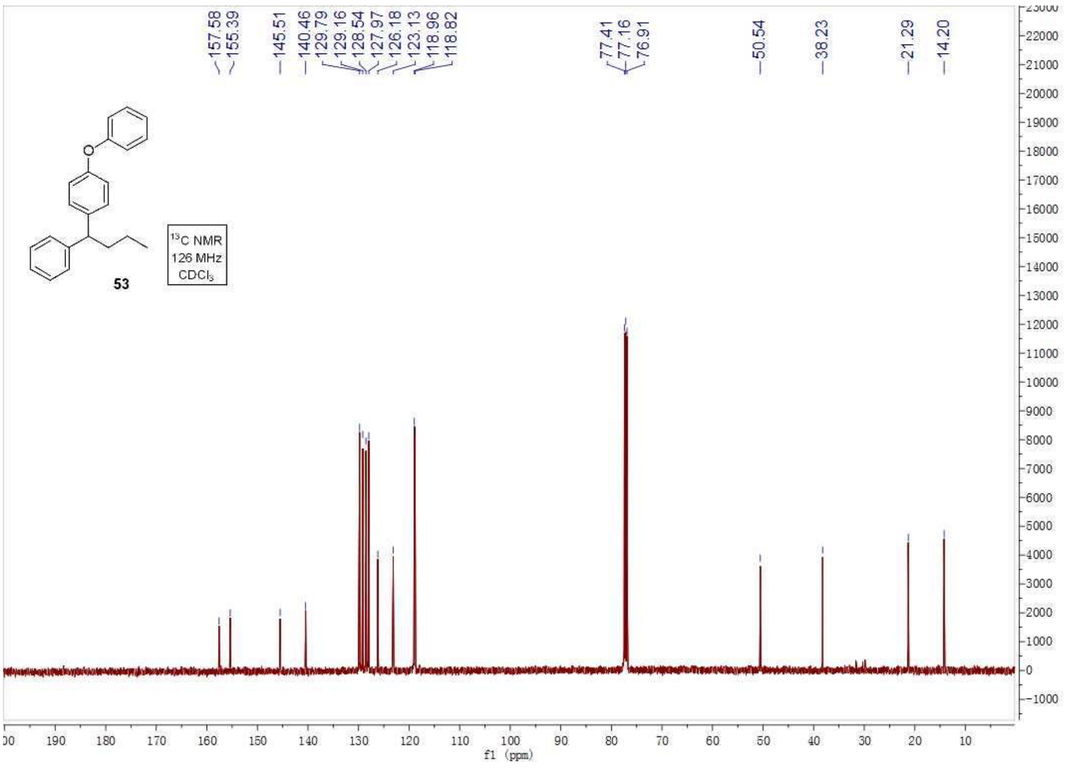
157.58 155.39
145.51 140.46 129.79 129.16 128.54 127.97 126.18 123.13 118.96 118.82
77.41 77.16 76.91
50.54
38.23
21.29
14.20
O
53
13C NMR
126 MHz
CDCl3
f1 (ppm)

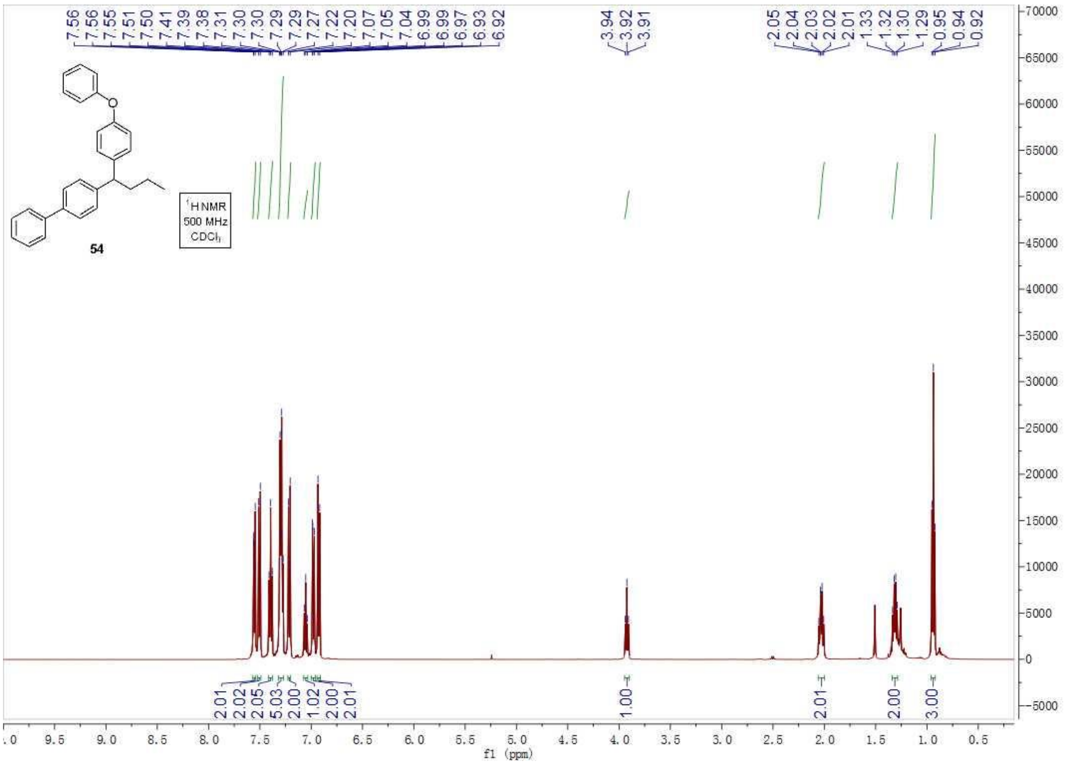

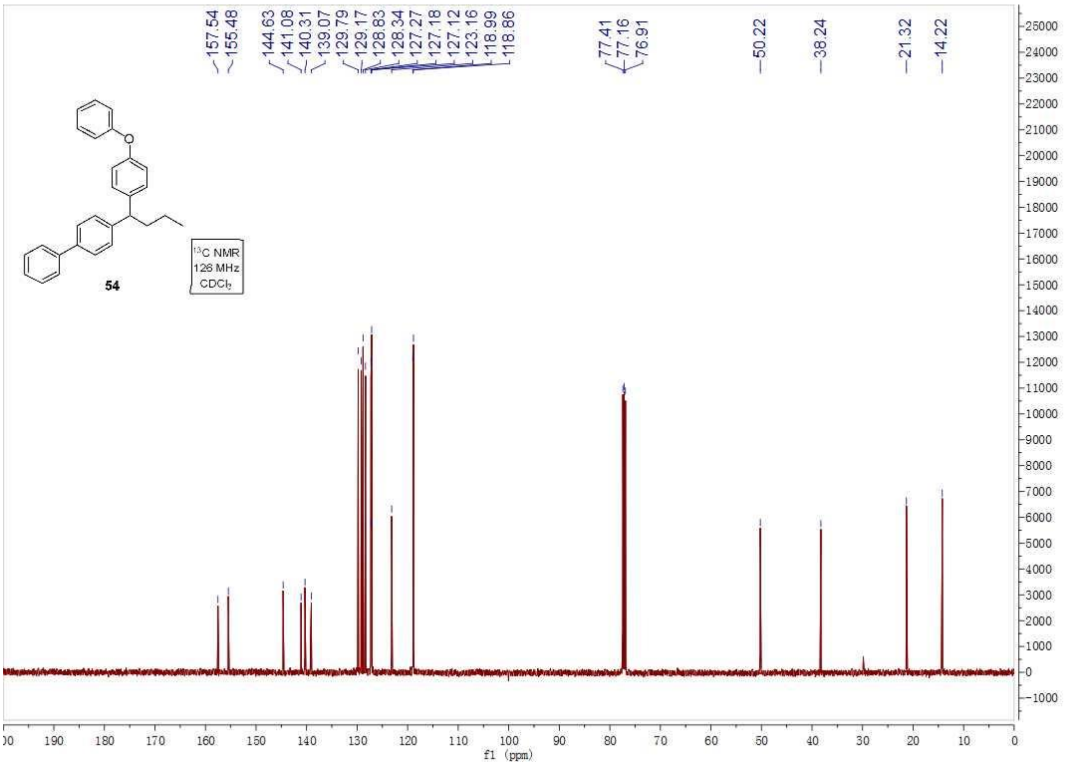

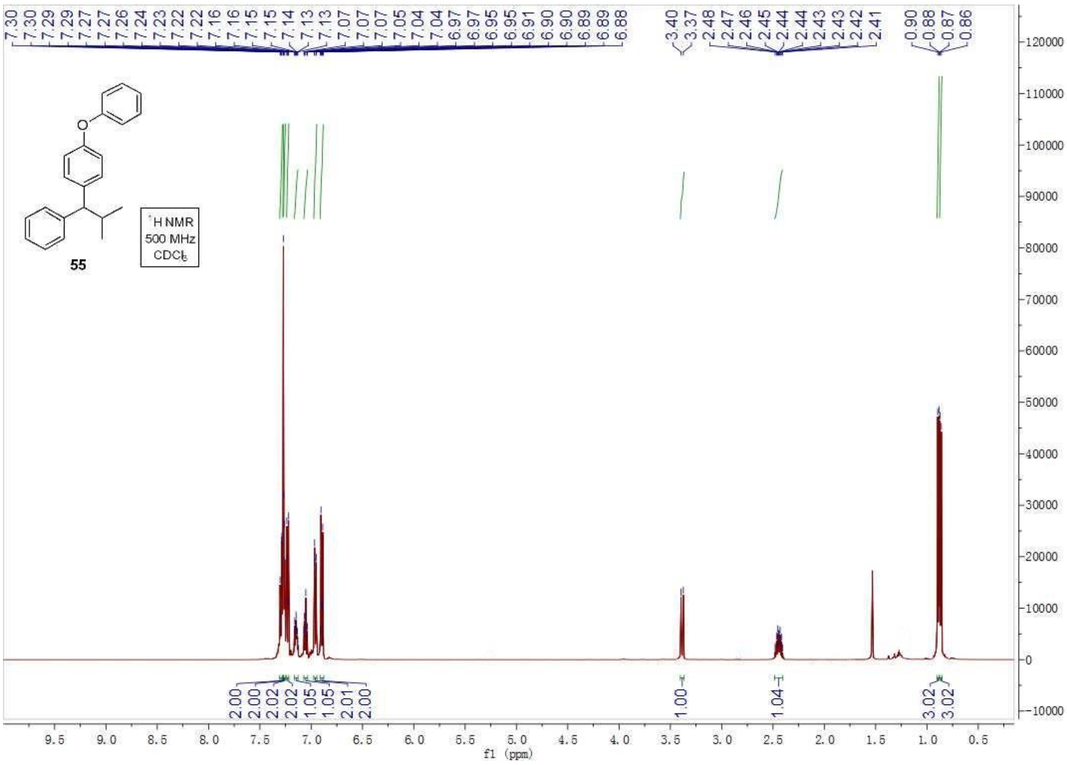
1H NMR
500 MHz
CDCl3
55
f1 (ppm)

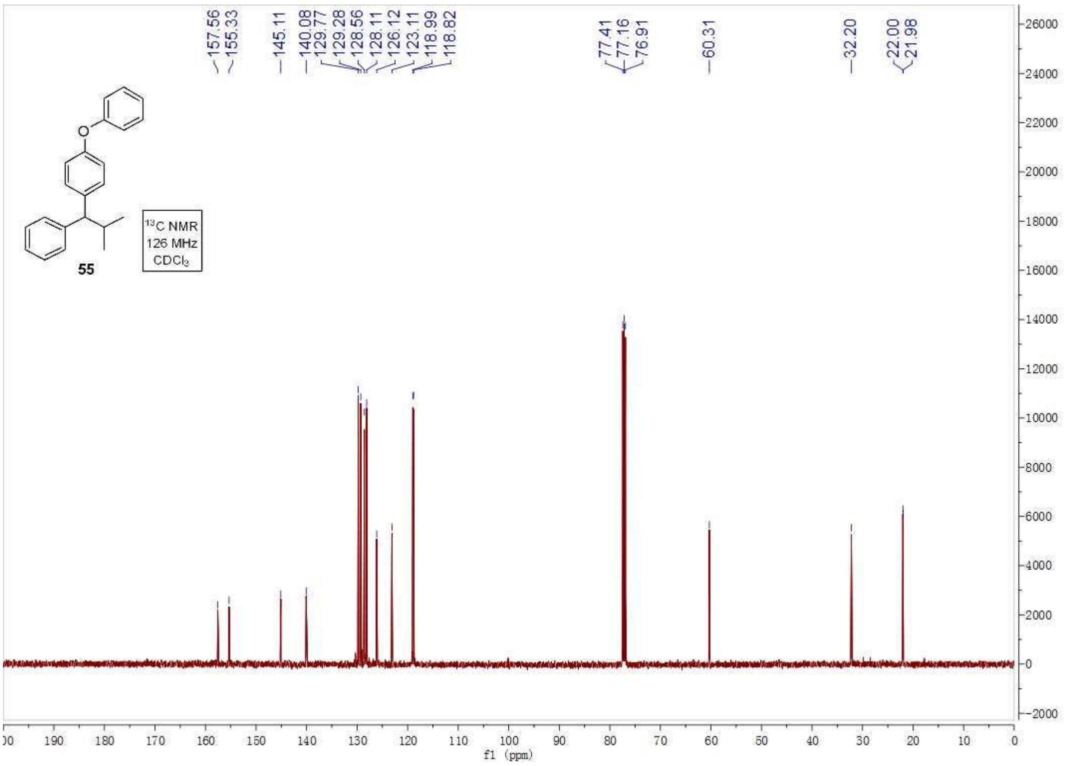
13C NMR
126 MHz
CDCl3
55
f1 (ppm)

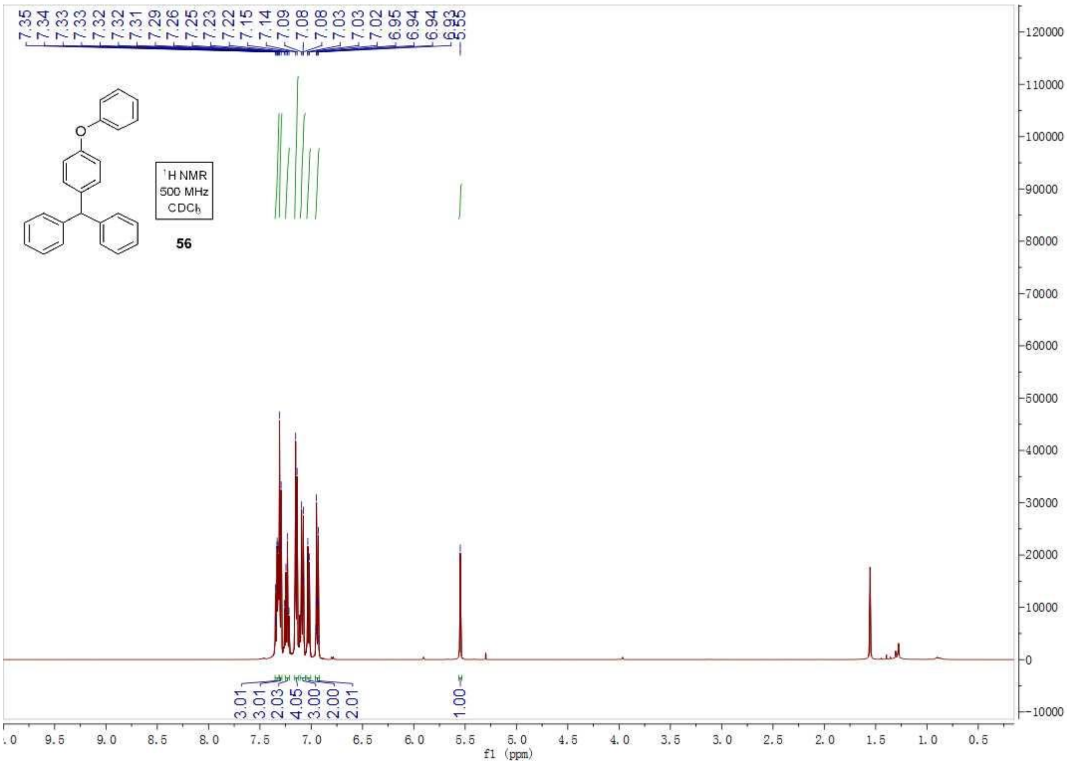
7.35
7.34
7.33
7.33
7.32
7.32
7.31
7.29
7.26
7.25
7.23
7.22
7.15
7.14
7.09
7.08
7.08
7.03
7.03
7.02
6.95
6.94
6.94
6.93
5.55
¹H NMR
500 MHz
CDCl₃
56
3.01
3.01
2.03
4.05
3.00
2.00
2.01
1.00
f1 (ppm)

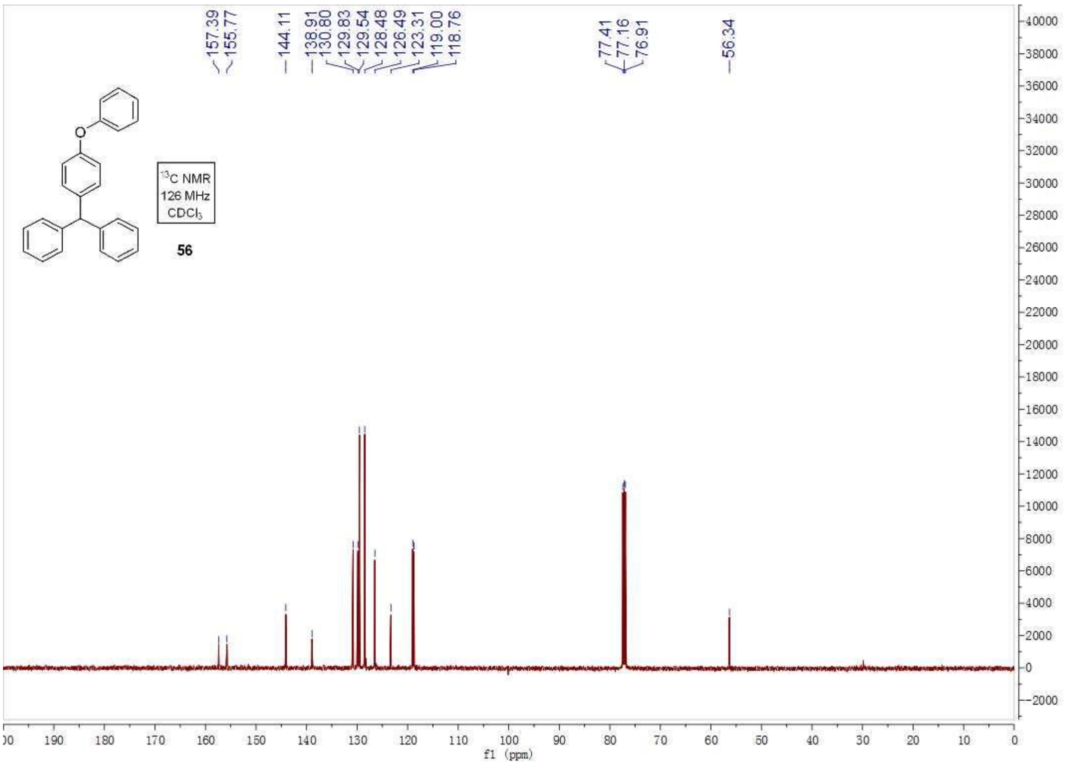
157.39
155.77
144.11
138.91
130.80
129.83
129.54
128.48
126.49
123.31
119.00
118.76
77.41
77.16
76.91
56.34
¹³C NMR
126 MHz
CDCl₃
56
f1 (ppm)

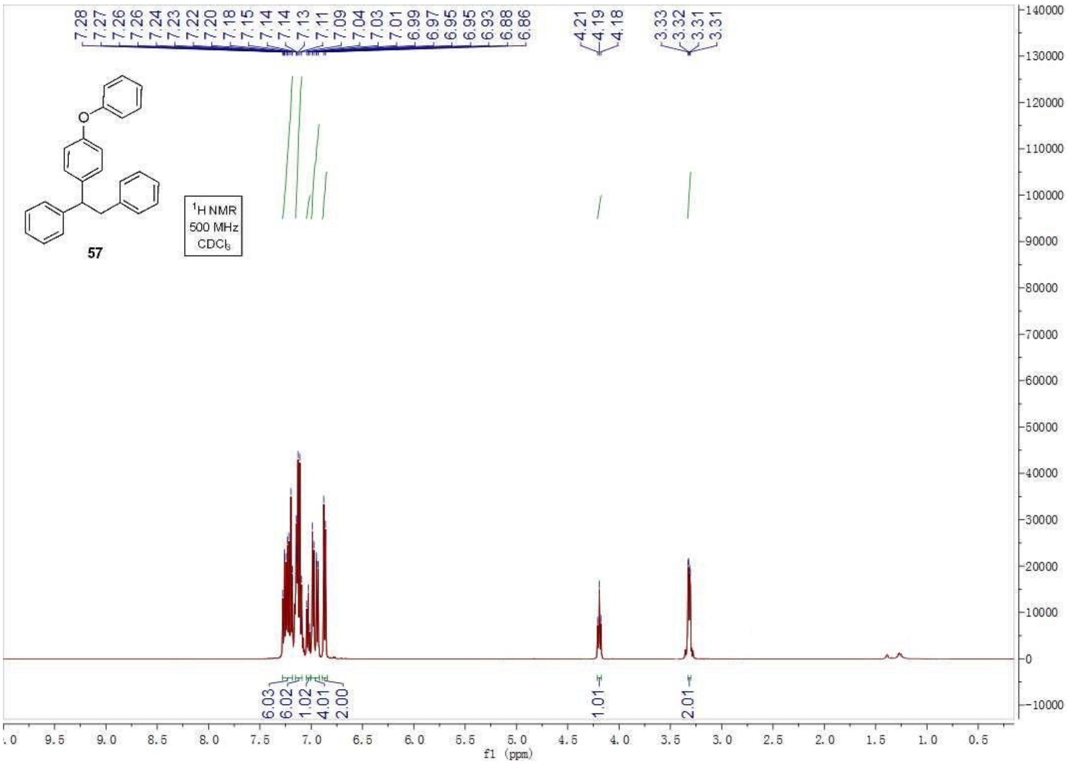
57
^{1}H NMR
500 MHz
$CDCl_3$
f1 (ppm)

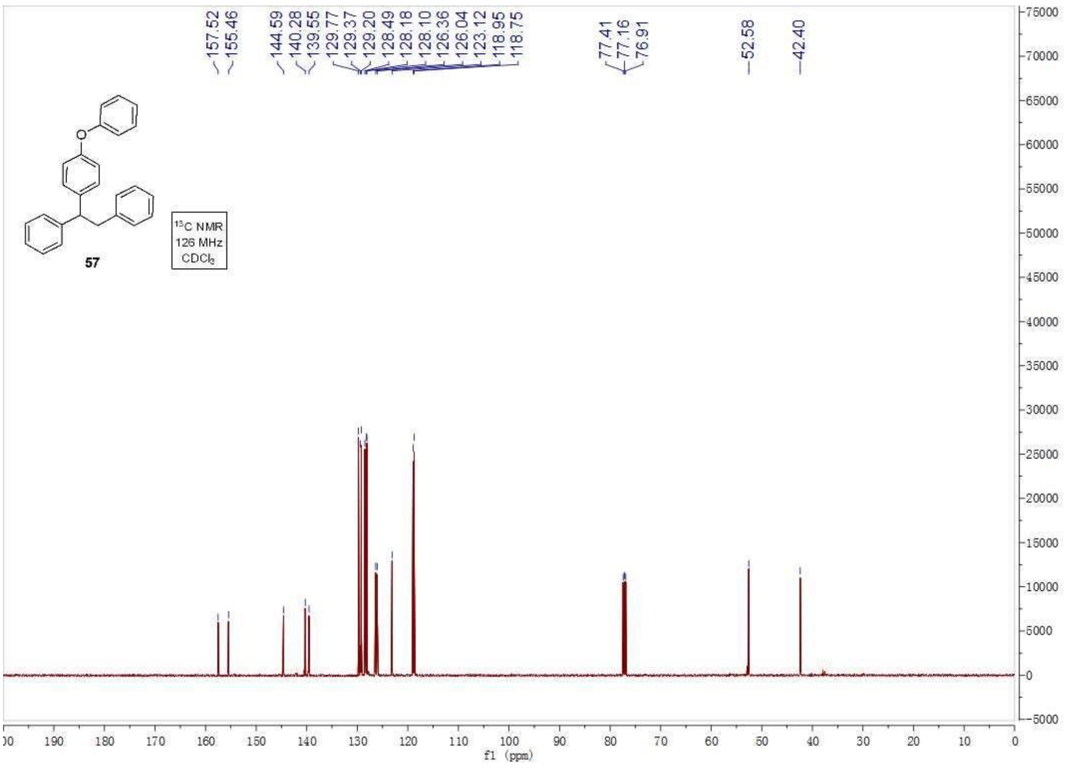
57
^{13}C NMR
126 MHz
$CDCl_3$
f1 (ppm)

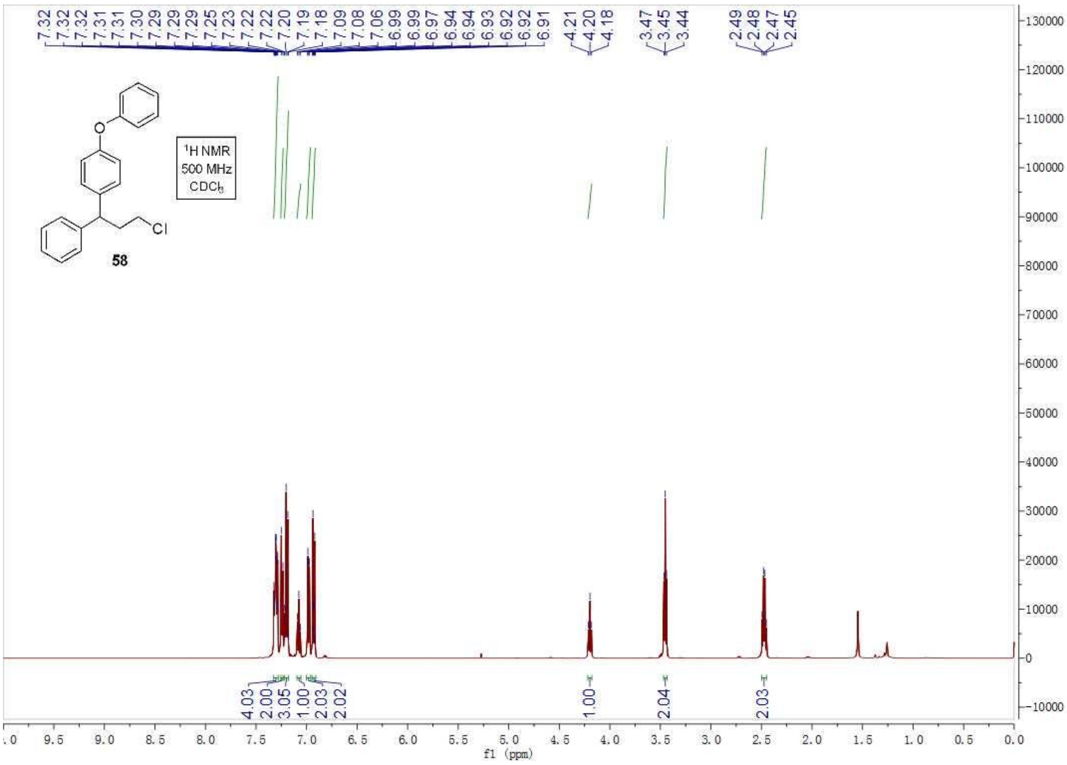

^{1}H NMR
500 MHz
$CDCl_3$
58
7.32
7.32
7.32
7.31
7.30
7.29
7.29
7.29
7.25
7.23
7.22
7.22
7.20
7.19
7.18
7.09
7.08
7.06
6.99
6.99
6.97
6.94
6.94
6.93
6.92
6.92
6.91
4.21
4.20
4.18
3.47
3.45
3.44
2.49
2.48
2.47
2.45
4.03
2.00
3.05
1.00
2.03
2.02
1.00
2.04
2.03
f1 (ppm)

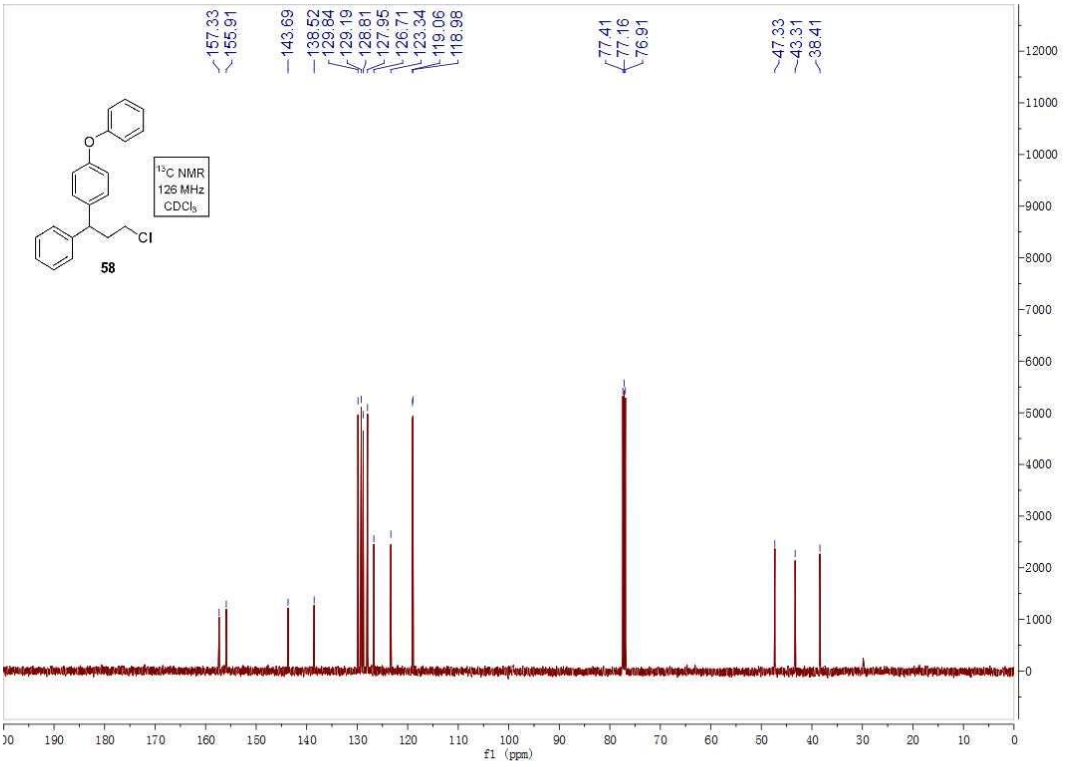

^{13}C NMR
126 MHz
$CDCl_3$
58
157.33
155.91
143.69
138.52
129.84
129.19
128.81
127.95
126.71
123.34
119.06
118.98
77.41
77.16
76.91
47.33
43.31
38.41
f1 (ppm)

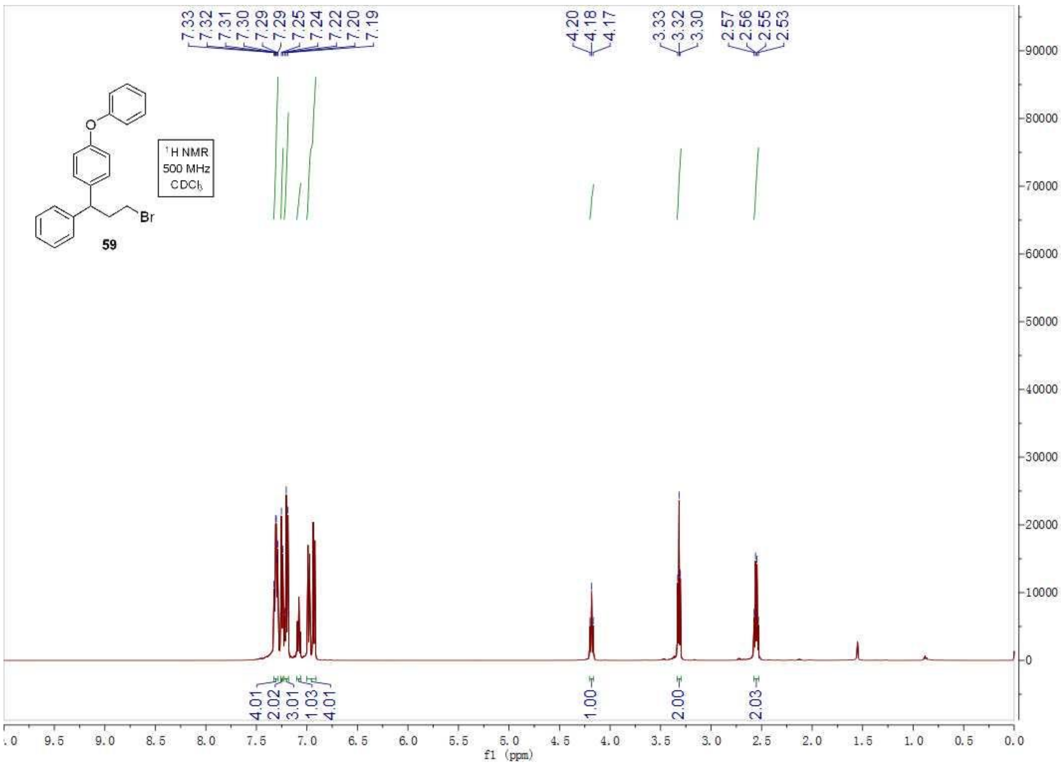

7.33
7.32
7.31
7.30
7.29
7.29
7.25
7.24
7.22
7.20
7.19
4.20
4.18
4.17
3.33
3.32
3.30
2.57
2.56
2.55
2.53
¹H NMR
500 MHz
CDCl₃
Br
59
4.01
2.02
3.01
1.03
4.01
1.00
2.00
2.03
f1 (ppm)

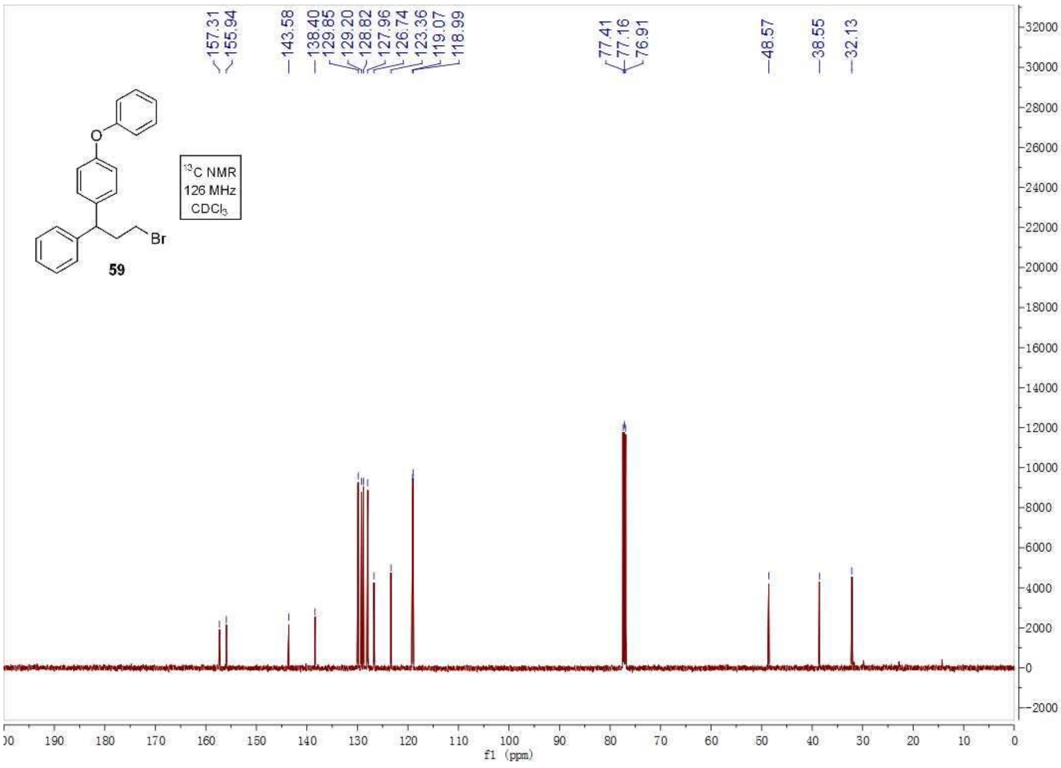

157.31
155.94
143.58
138.40
129.85
129.20
128.82
127.96
126.74
123.36
119.07
118.99
77.41
77.16
76.91
48.57
38.55
32.13
¹³C NMR
126 MHz
CDCl₃
Br
59
f1 (ppm)

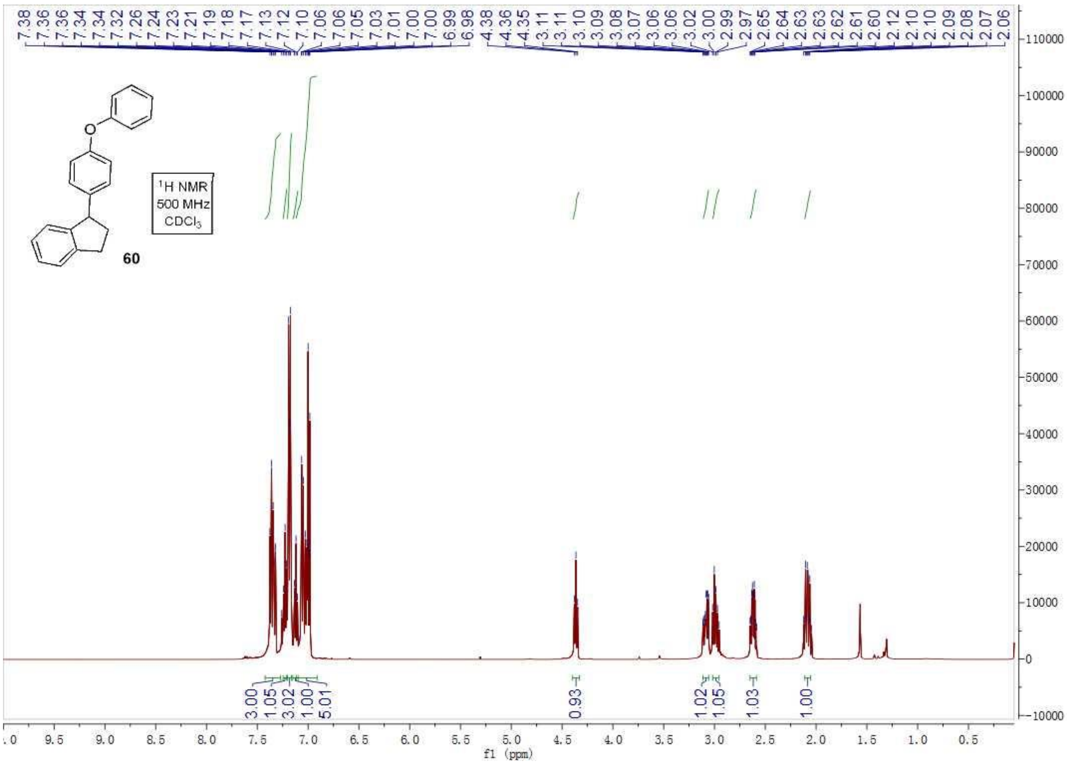

7.38 7.36 7.36 7.34 7.34 7.32 7.26 7.24 7.23 7.21 7.19 7.18 7.17 7.13 7.12 7.10 7.06 7.06 7.05 7.03 7.01 7.00 7.00 6.99 6.98 4.38 4.36 4.35 3.11 3.11 3.10 3.09 3.08 3.07 3.06 3.06 3.02 3.00 2.99 2.97 2.65 2.64 2.63 2.63 2.62 2.61 2.60 2.12 2.10 2.10 2.09 2.08 2.07 2.06
^{1}H NMR
500 MHz
$CDCl_3$
60
3.00 1.05 3.02 1.00 5.01 0.93 1.02 1.05 1.03 1.00
f1 (ppm)

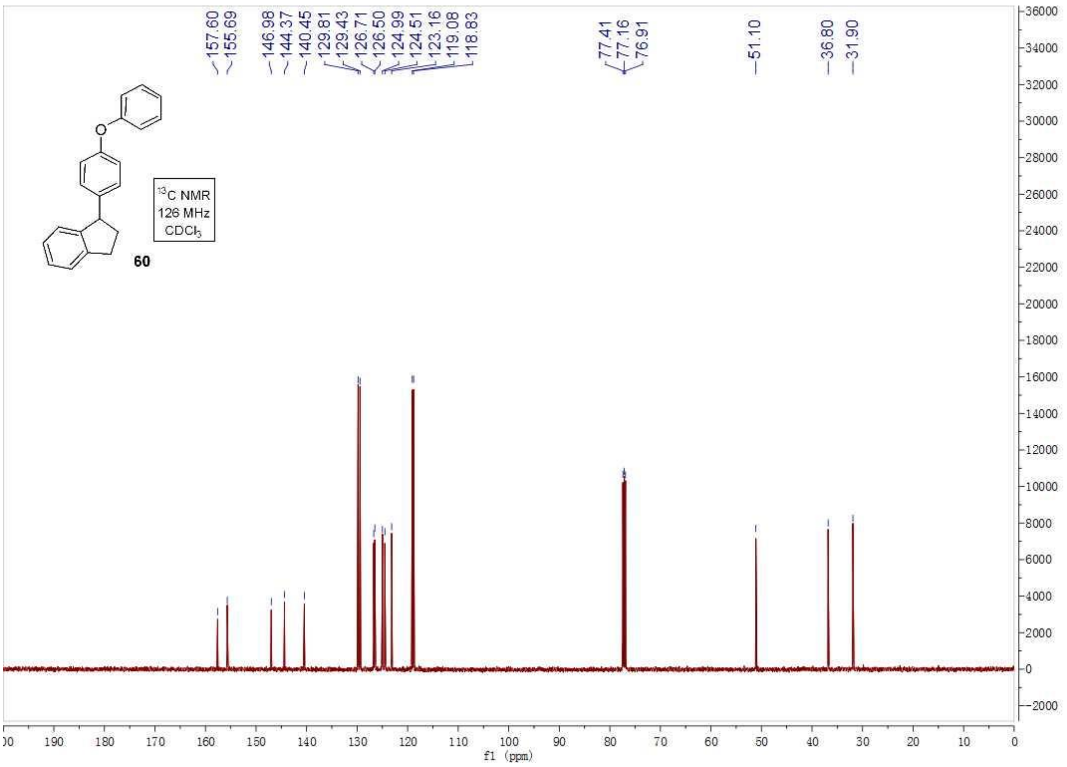

157.60 155.69 146.98 144.37 140.45 129.81 129.43 126.71 126.50 124.99 124.51 123.16 119.08 118.83 77.41 77.16 76.91 51.10 36.80 31.90
^{13}C NMR
126 MHz
$CDCl_3$
60
f1 (ppm)

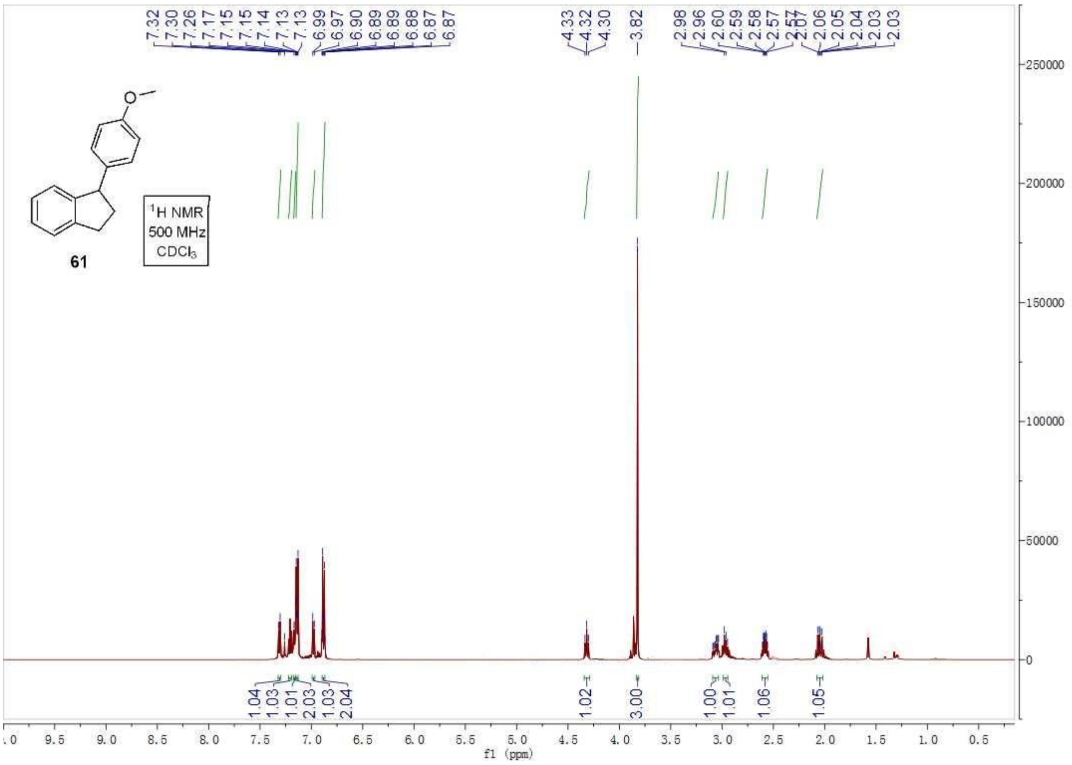

7.32
7.30
7.26
7.17
7.15
7.15
7.14
7.13
7.13
6.99
6.97
6.90
6.89
6.89
6.88
6.87
6.87
4.33
4.32
4.30
3.82
2.98
2.96
2.60
2.59
2.58
2.57
2.57
2.07
2.06
2.05
2.04
2.03
2.03
^{1}H NMR
500 MHz
CDCl$_3$
61
1.04
1.03
1.01
2.03
1.03
2.04
1.02
3.00
1.00
1.01
1.06
1.05
f1 (ppm)

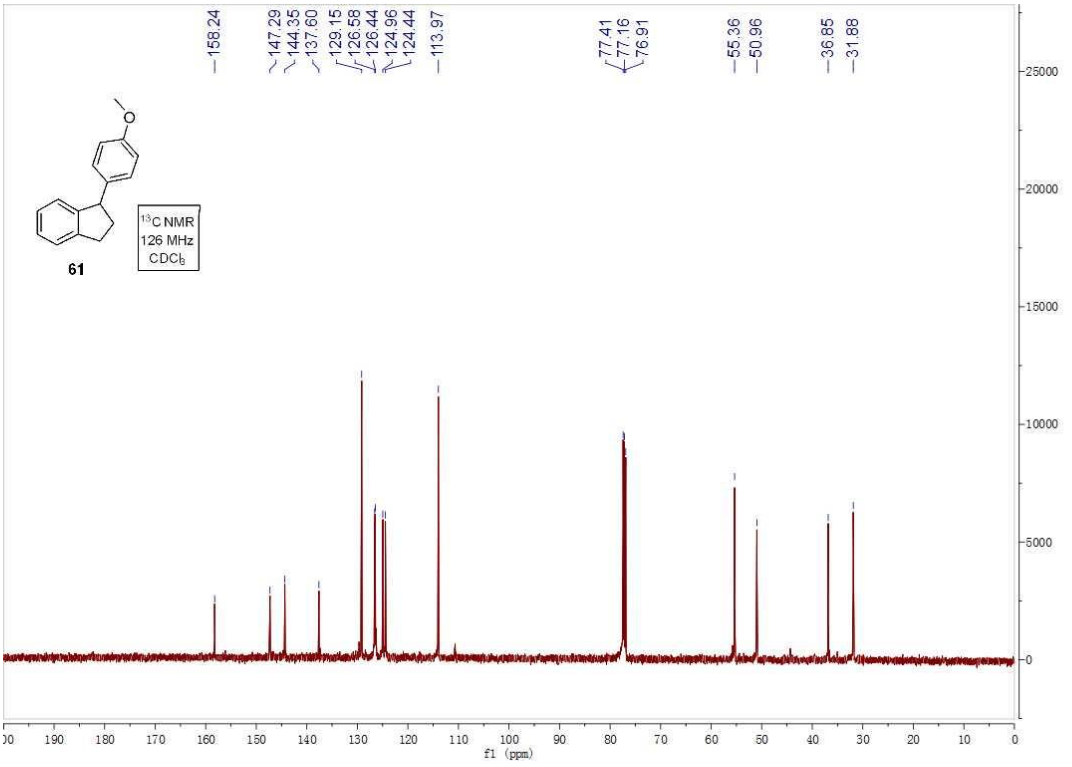

158.24
147.29
144.35
137.60
129.15
126.58
126.44
124.96
124.44
113.97
77.41
77.16
76.91
55.36
50.96
36.85
31.88
^{13}C NMR
126 MHz
CDCl$_3$
61
f1 (ppm)

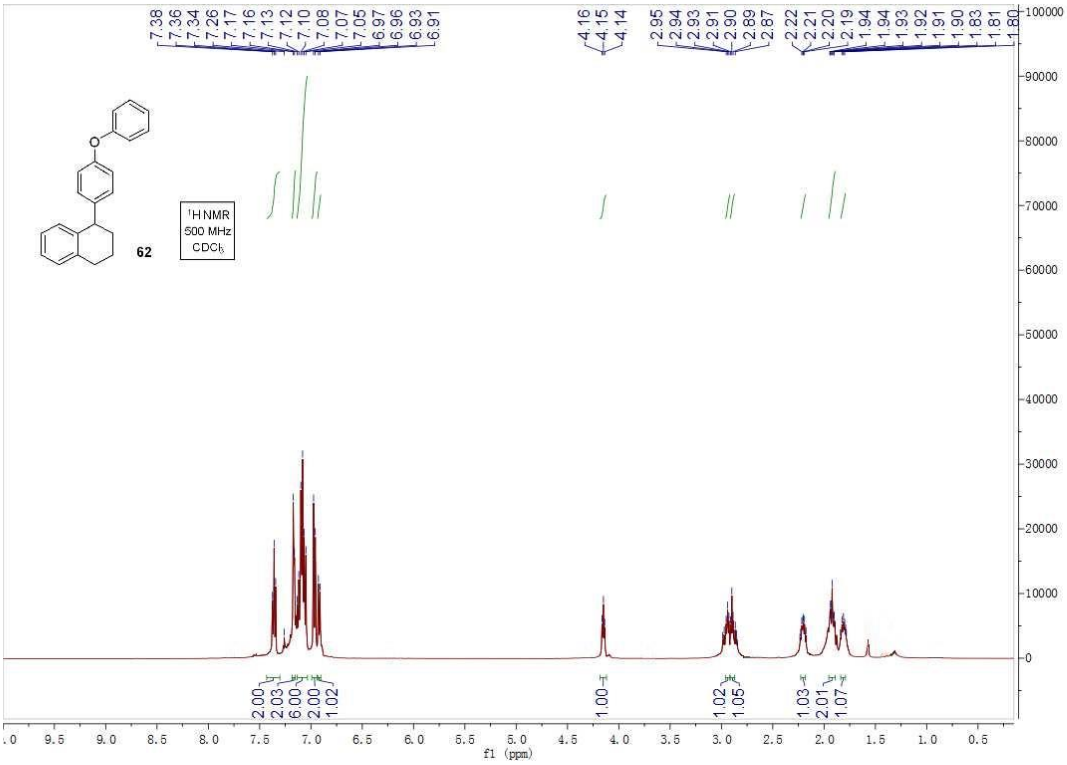

7.38 7.36 7.34 7.26 7.17 7.16 7.13 7.12 7.10 7.08 7.07 7.05 6.97 6.96 6.93 6.91
4.16 4.15 4.14
2.95 2.94 2.93 2.91 2.90 2.89 2.87
2.22 2.21 2.20 2.19 1.94 1.94 1.93 1.92 1.91 1.90 1.83 1.81 1.80
O
62
^{1}H NMR
500 MHz
$CDCl_3$
2.00 2.03 6.00 2.00 1.02
1.00
1.02 1.05
1.03 2.01 1.07
f1 (ppm)

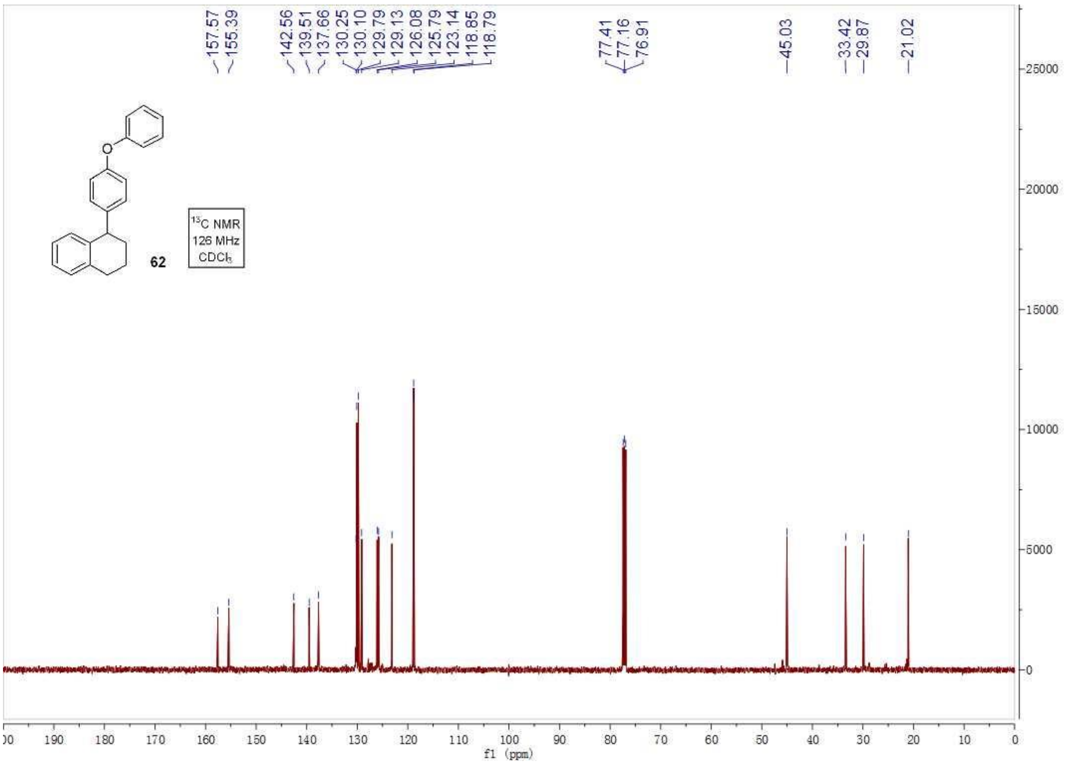

157.57 155.39
142.56 139.51 137.66 130.25 130.10 129.79 129.13 126.08 125.79 123.14 118.85 118.79
77.41 77.16 76.91
45.03
33.42 29.87
21.02
O
62
^{13}C NMR
126 MHz
$CDCl_3$
f1 (ppm)

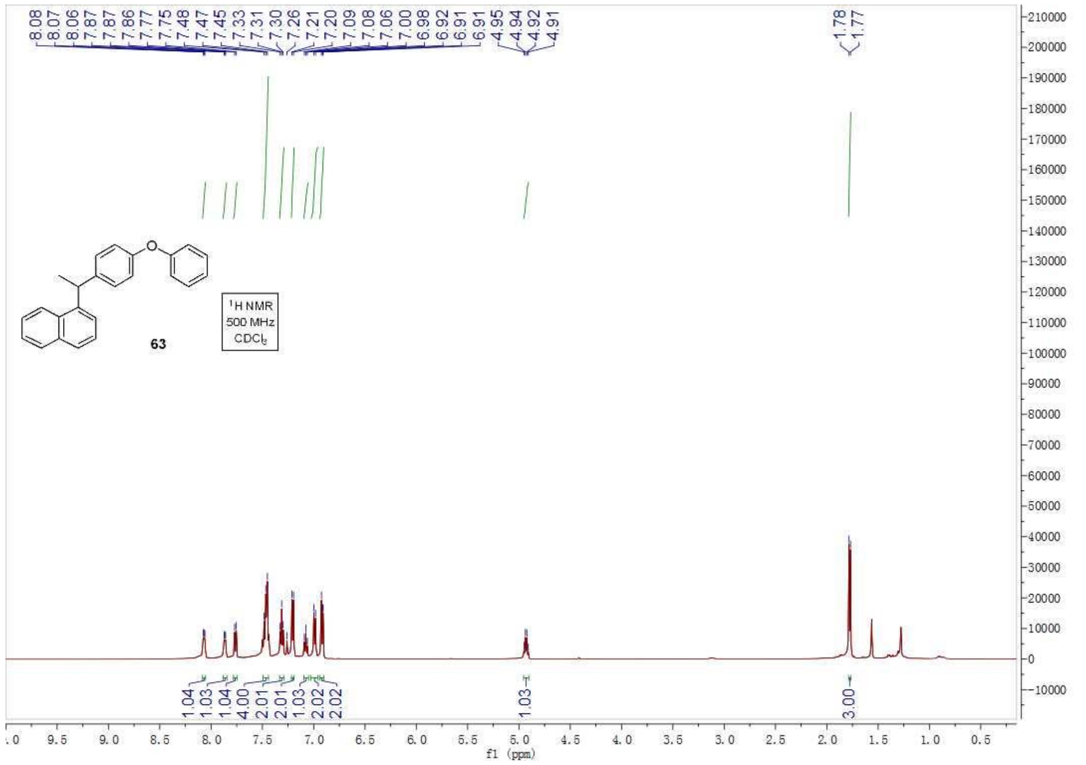

1H NMR
500 MHz
CDCl3
63
f1 (ppm)

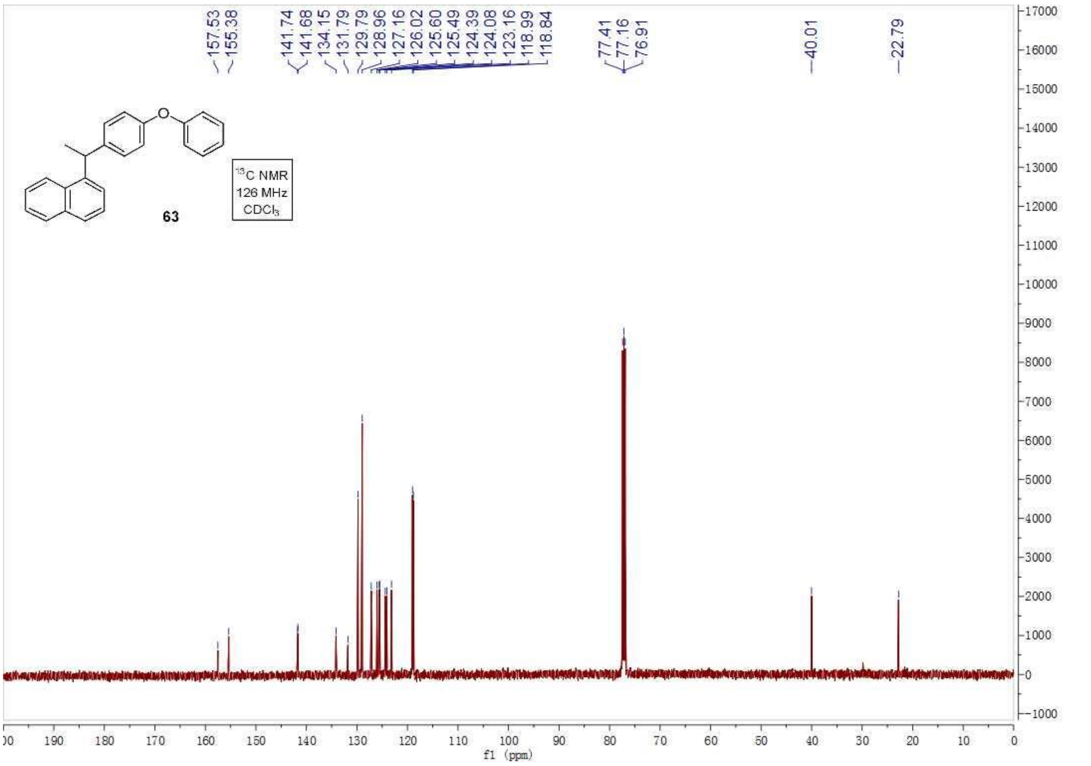

13C NMR
126 MHz
CDCl3
63
f1 (ppm)

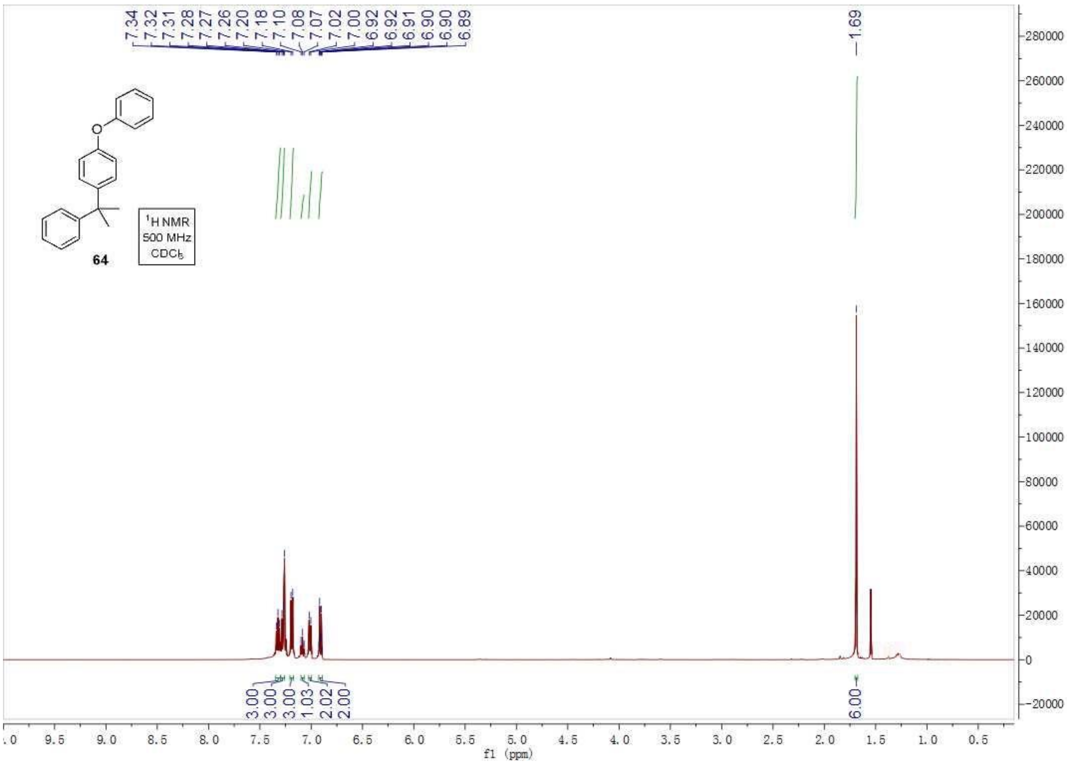

1H NMR
500 MHz
CDCl3
64

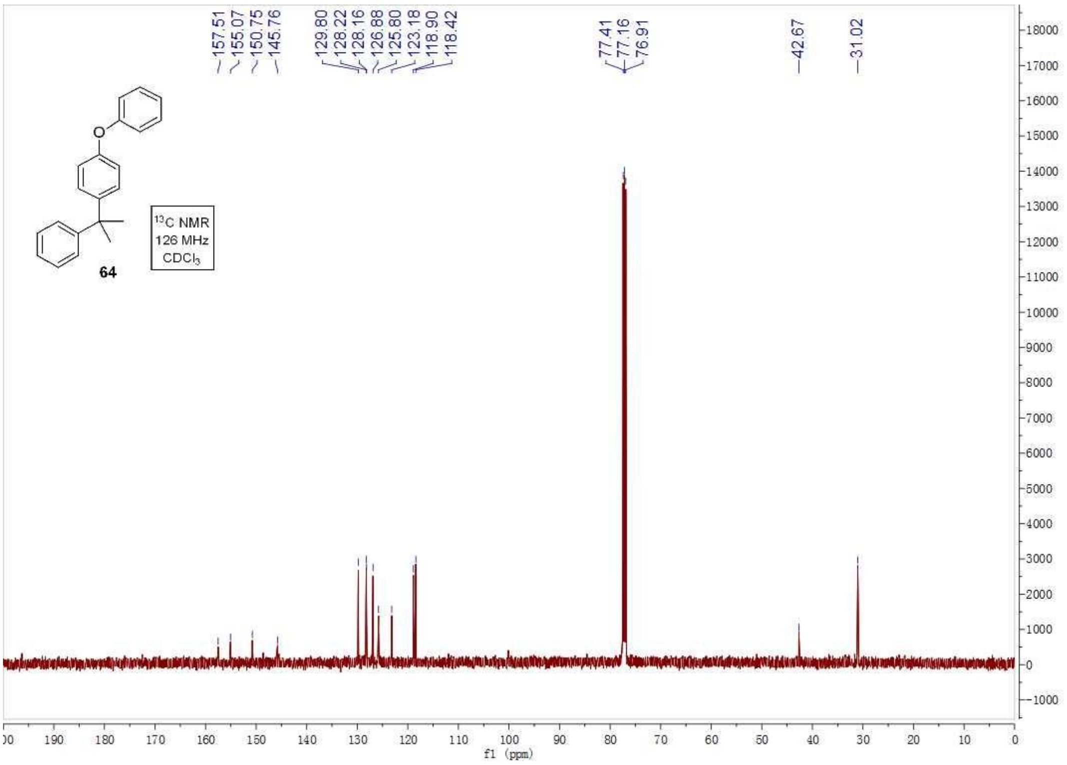

13C NMR
126 MHz
CDCl3
64

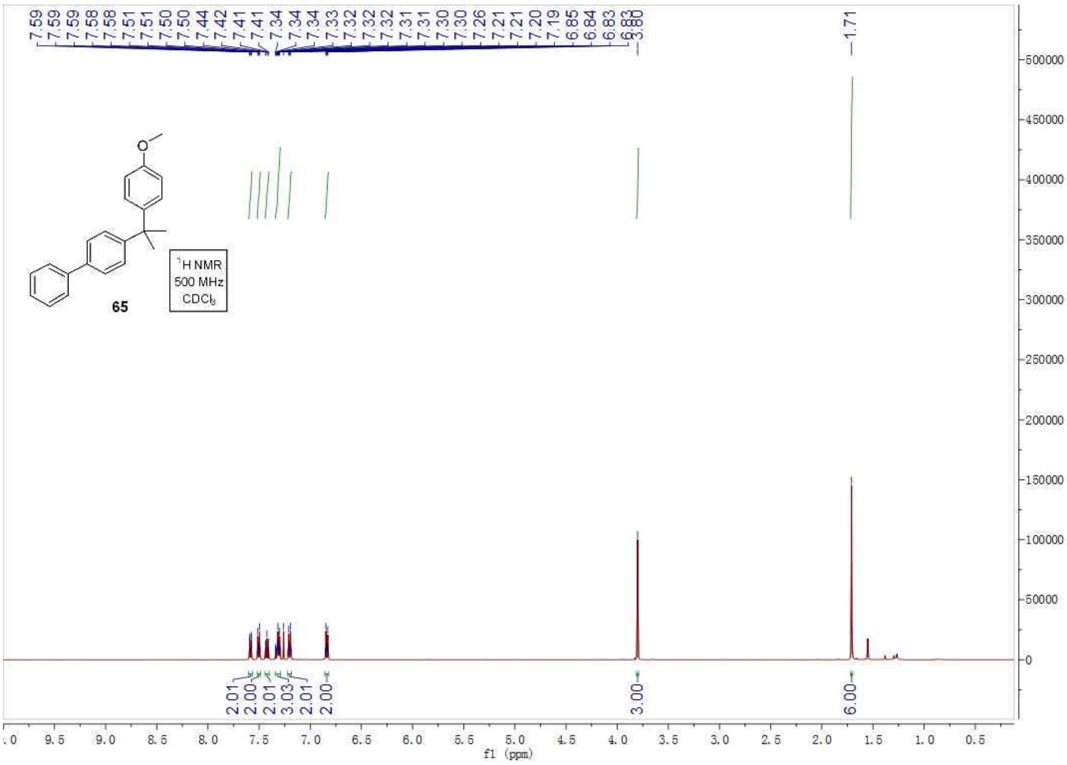

7.59
7.59
7.59
7.58
7.58
7.51
7.51
7.50
7.50
7.44
7.42
7.41
7.41
7.34
7.34
7.34
7.33
7.32
7.32
7.32
7.31
7.31
7.30
7.30
7.26
7.21
7.21
7.20
7.19
6.85
6.84
6.83
3.80
1.71
1H NMR
500 MHz
CDCl3
65
2.01
2.00
2.01
3.03
2.01
2.00
3.00
6.00
f1 (ppm)

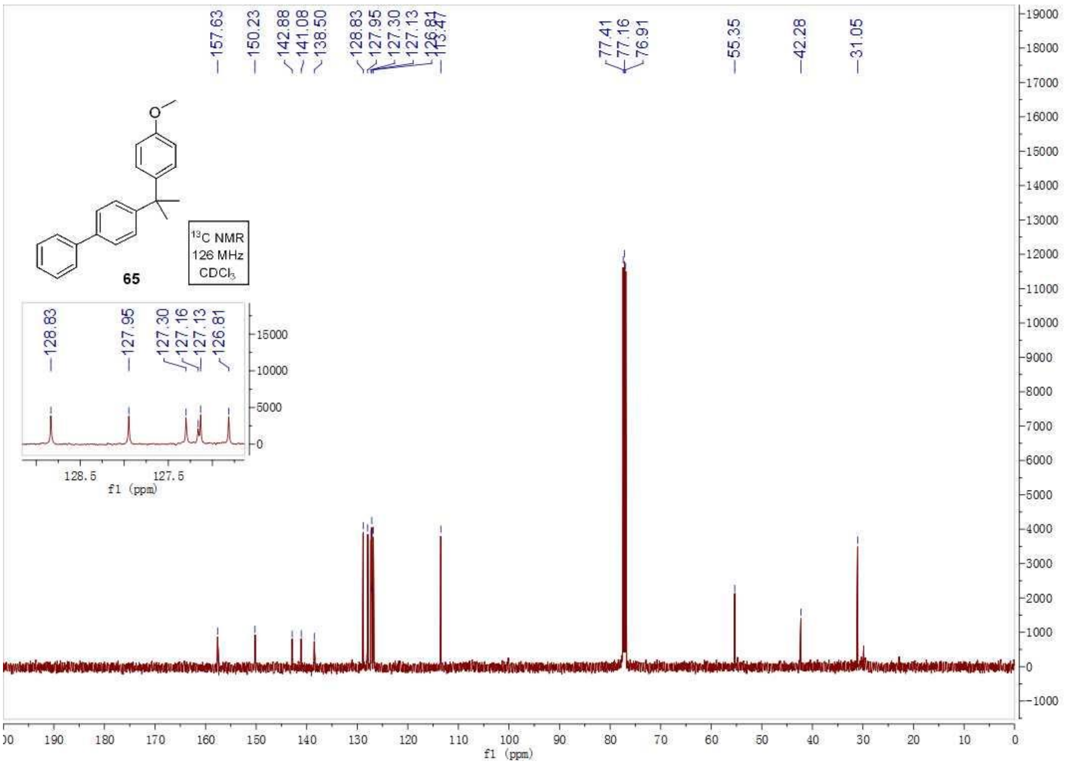

157.63
150.23
142.88
141.08
138.50
128.83
127.95
127.30
127.13
126.81
113.47
77.41
77.16
76.91
55.35
42.28
31.05
13C NMR
126 MHz
CDCl3
65
128.83
127.95
127.30
127.16
127.13
126.81
f1 (ppm)

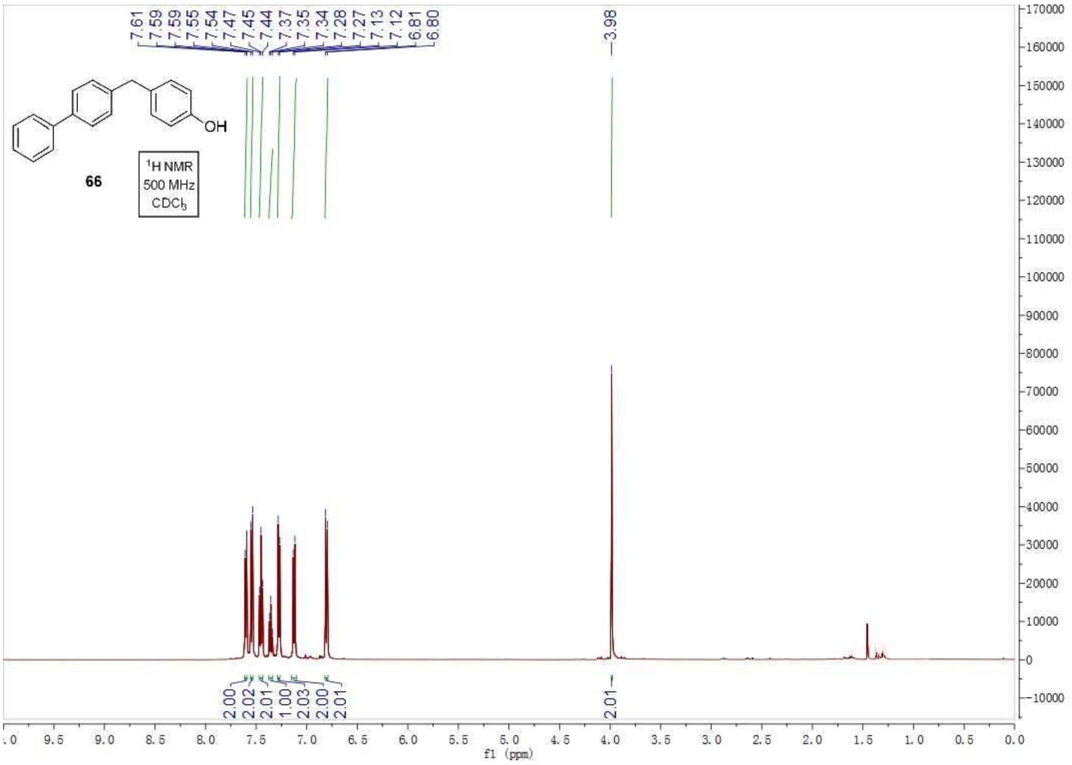
7.61
7.59
7.59
7.55
7.54
7.47
7.45
7.44
7.37
7.35
7.34
7.28
7.27
7.13
7.12
6.81
6.80
3.98
OH
66
^{1}H NMR
500 MHz
$CDCl_3$
2.00
2.02
2.01
1.00
2.03
2.00
2.01
2.01
f1 (ppm)

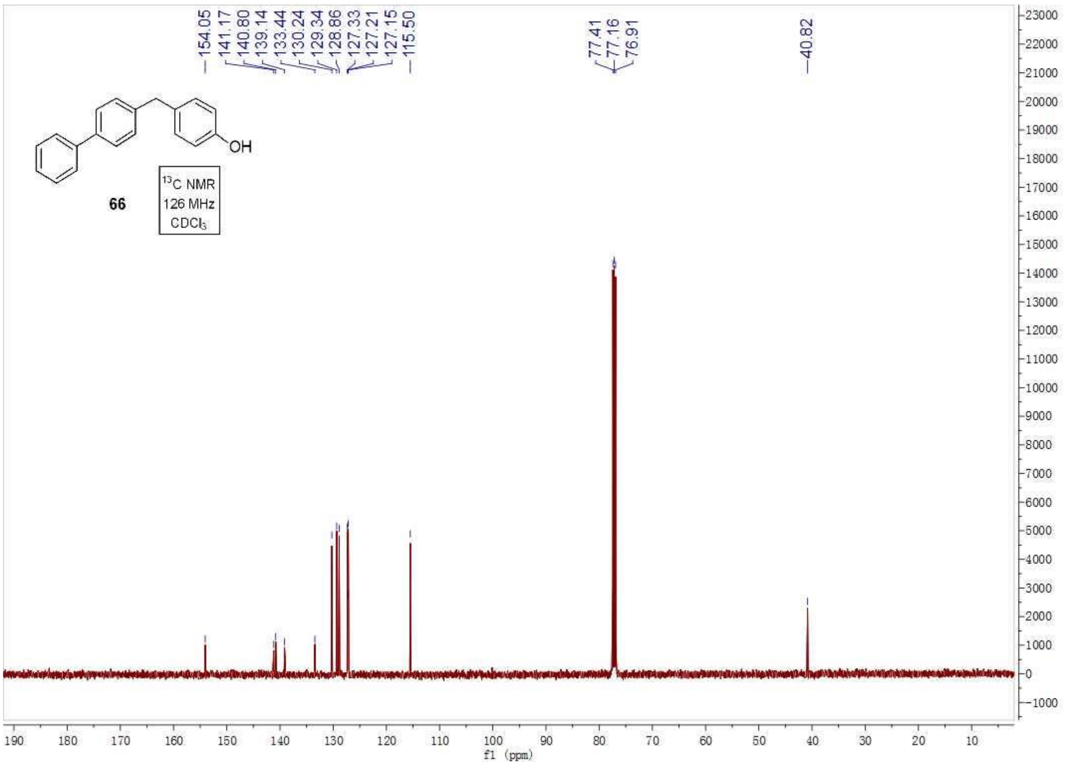
154.05
141.17
140.80
139.14
133.44
130.24
129.34
128.86
127.33
127.21
127.15
115.50
77.41
77.16
76.91
40.82
OH
66
^{13}C NMR
126 MHz
$CDCl_3$
f1 (ppm)

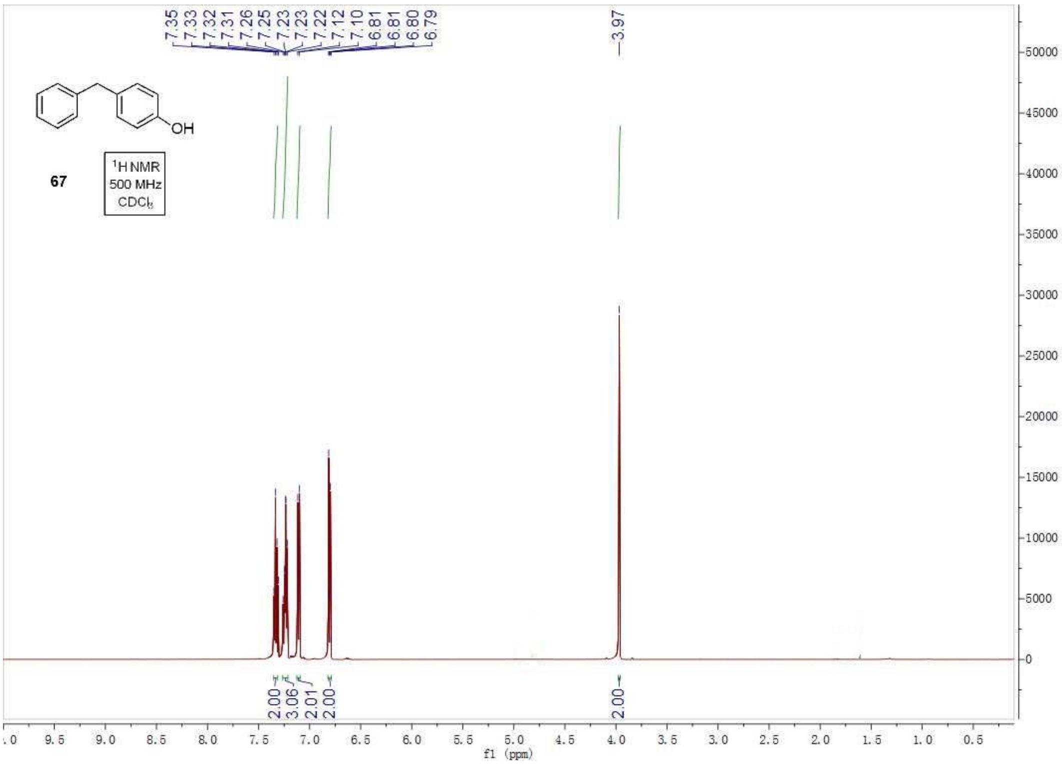
7.35
7.33
7.32
7.31
7.26
7.25
7.23
7.23
7.22
7.12
7.10
6.81
6.81
6.80
6.79
3.97
OH
67
1H NMR
500 MHz
CDCl3
2.00
3.06
2.01
2.00
2.00
f1 (ppm)

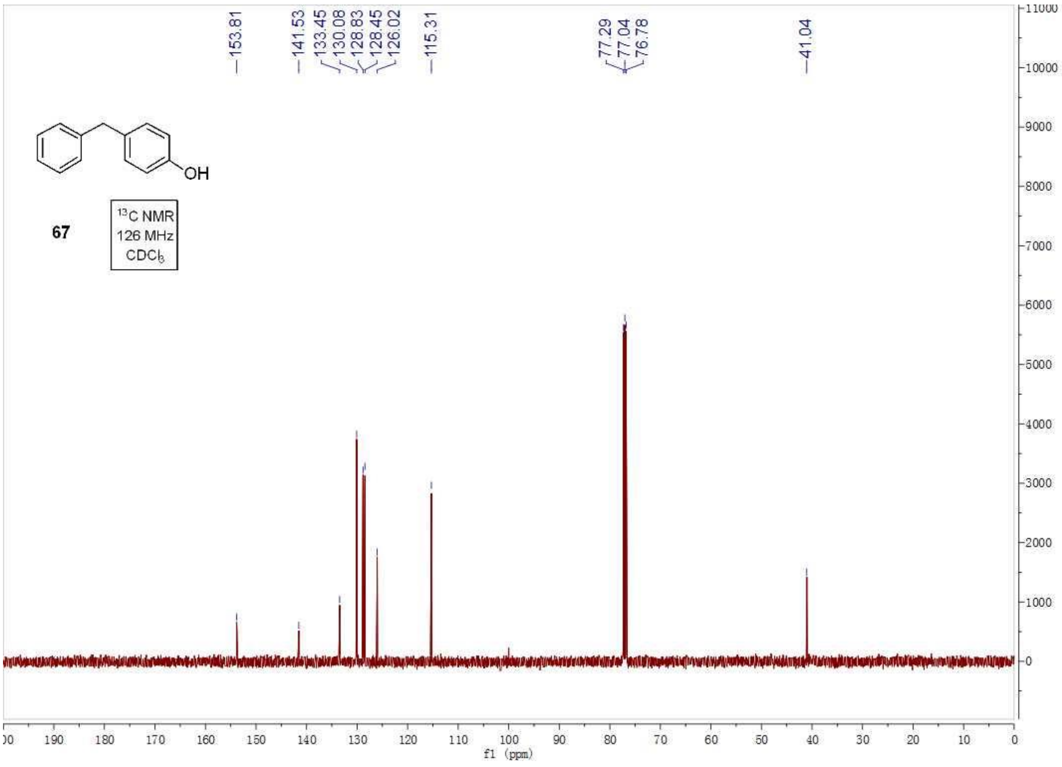
153.81
141.53
133.45
130.08
128.83
128.45
126.02
115.31
77.29
77.04
76.78
41.04
OH
67
13C NMR
126 MHz
CDCl3
f1 (ppm)

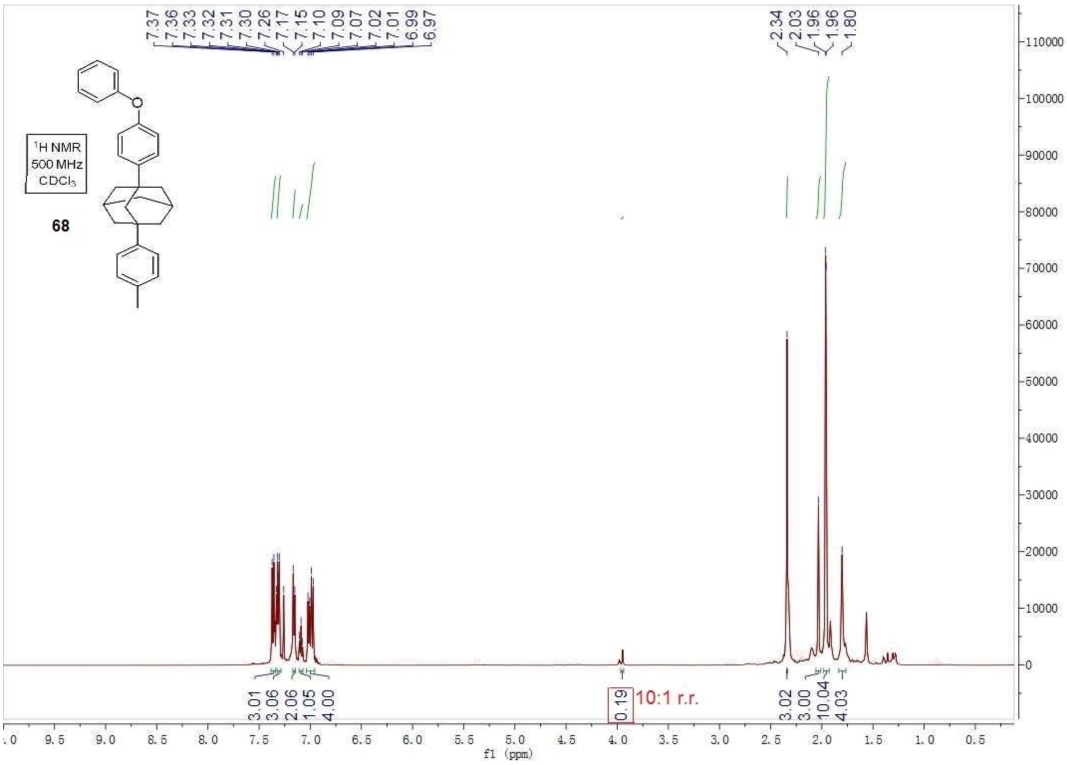

7.37 7.36 7.33 7.32 7.31 7.30 7.26 7.17 7.15 7.10 7.09 7.07 7.02 7.01 6.99 6.97
2.34 2.03 1.96 1.96 1.80
^{1}H NMR
500 MHz
CDCl$_3$
68
3.01 3.06 2.06 1.05 4.00
0.19
10:1 r.r.
3.02 3.00 10.04 4.03
f1 (ppm)

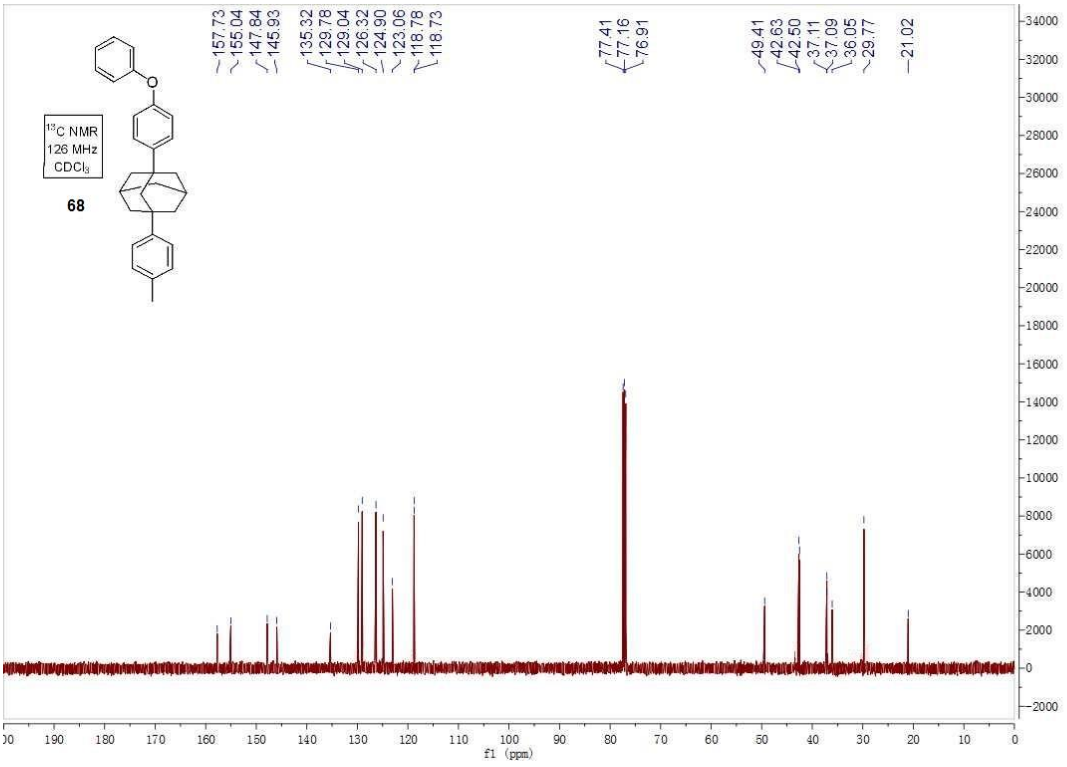

157.73 155.04 147.84 145.93
135.32 129.78 129.04 126.32 124.90 123.06 118.78 118.73
77.41 77.16 76.91
49.41 42.63 42.50 37.11 37.09 36.05 29.77
21.02
^{13}C NMR
126 MHz
CDCl$_3$
68
f1 (ppm)

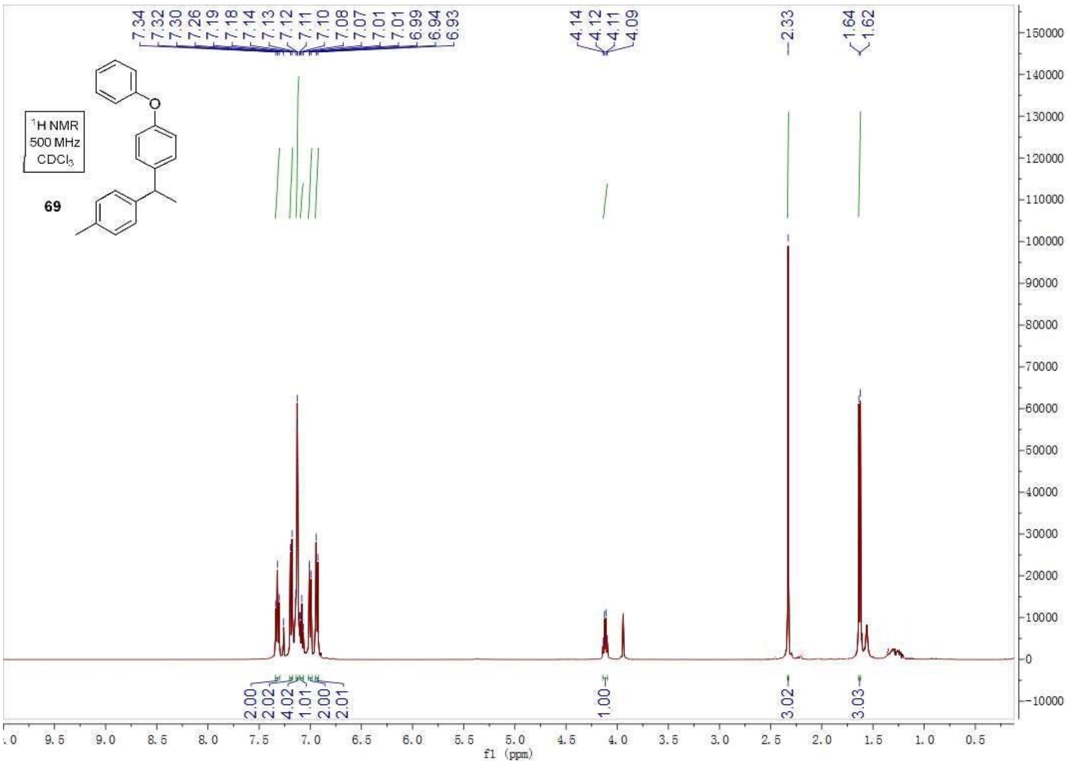
1H NMR
500 MHz
CDCl3
69
7.34 7.32 7.30 7.26 7.19 7.18 7.14 7.13 7.12 7.11 7.10 7.08 7.07 7.01 7.01 6.99 6.94 6.93
4.14 4.12 4.11 4.09
2.33
1.64 1.62
2.00 2.02 4.02 1.01 2.00 2.01
1.00
3.02
3.03
f1 (ppm)

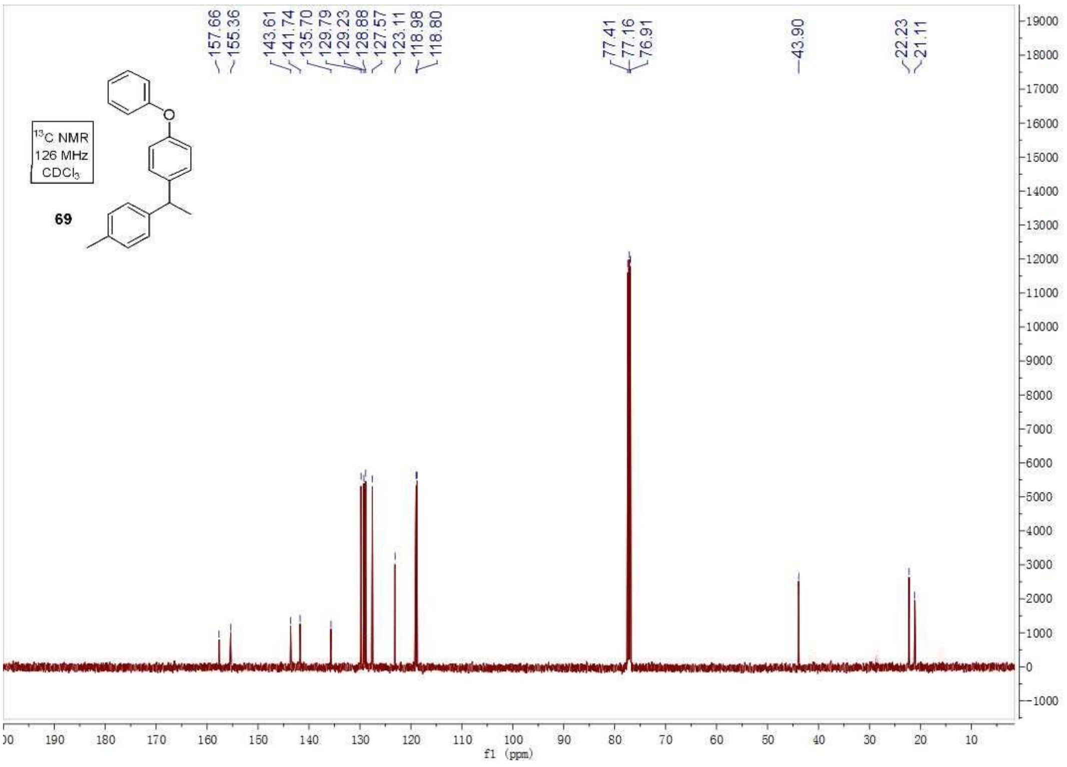
13C NMR
126 MHz
CDCl3
69
157.66 155.36
143.61 141.74 135.70 129.79 129.23 128.88 127.57 123.11 118.98 118.80
77.41 77.16 76.91
43.90
22.23 21.11
f1 (ppm)

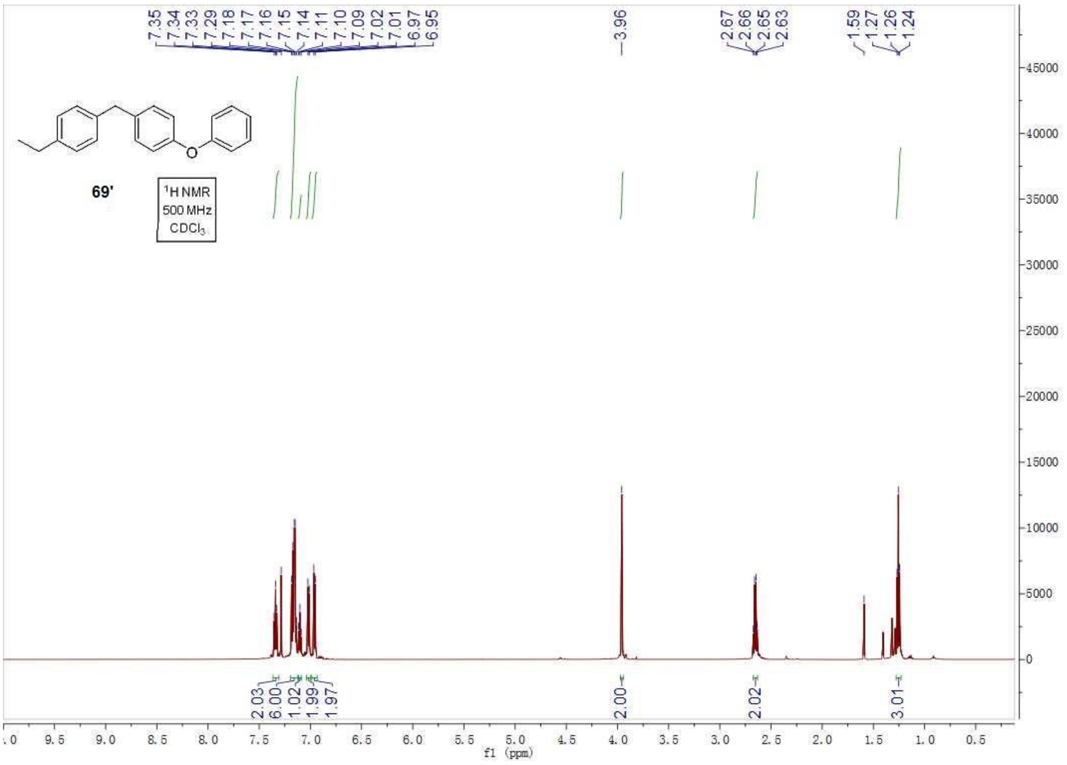

7.35
7.34
7.33
7.29
7.18
7.17
7.16
7.15
7.14
7.11
7.10
7.09
7.02
7.01
6.97
6.95
3.96
2.67
2.66
2.65
2.63
1.59
1.27
1.26
1.24
69'
^{1}H NMR
500 MHz
$CDCl_3$
2.03
6.00
1.02
1.99
1.97
2.00
2.02
3.01
f1 (ppm)

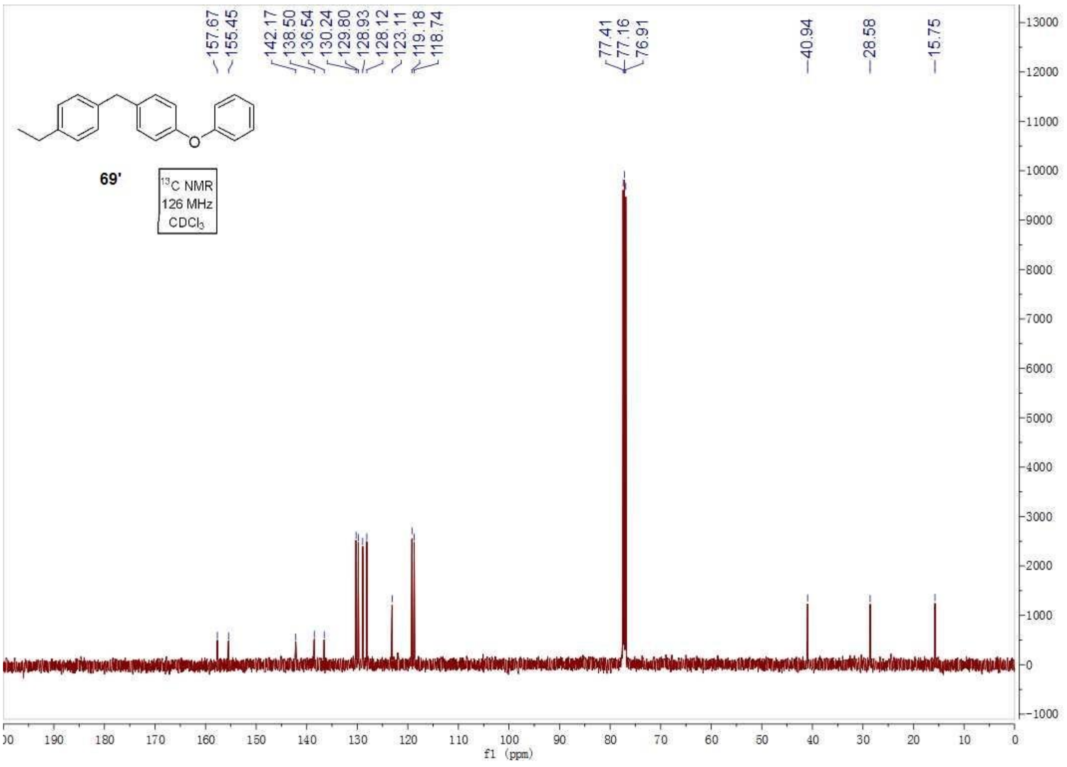

157.67
155.45
142.17
138.50
136.54
130.24
129.80
128.93
128.12
123.11
119.18
118.74
77.41
77.16
76.91
40.94
28.58
15.75
69'
^{13}C NMR
126 MHz
$CDCl_3$
f1 (ppm)

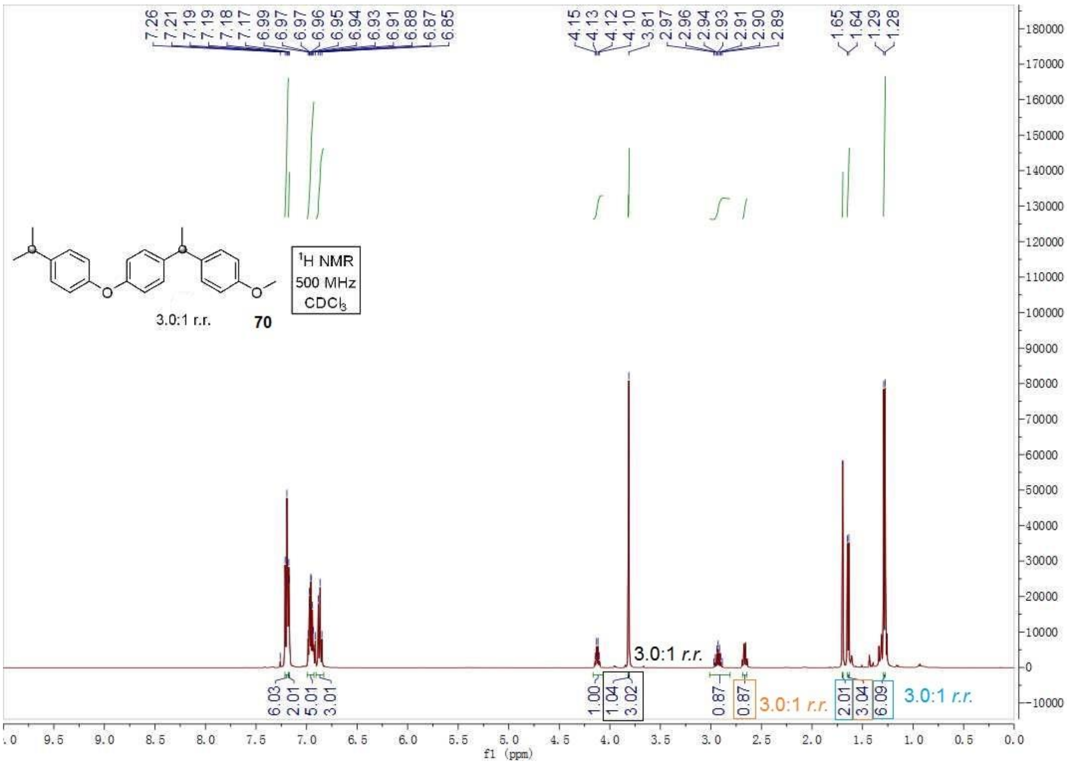

1H NMR
500 MHz
CDCl3
3.0:1 r.r.
70
3.0:1 r.r.
3.0:1 r.r.
3.0:1 r.r.
f1 (ppm)

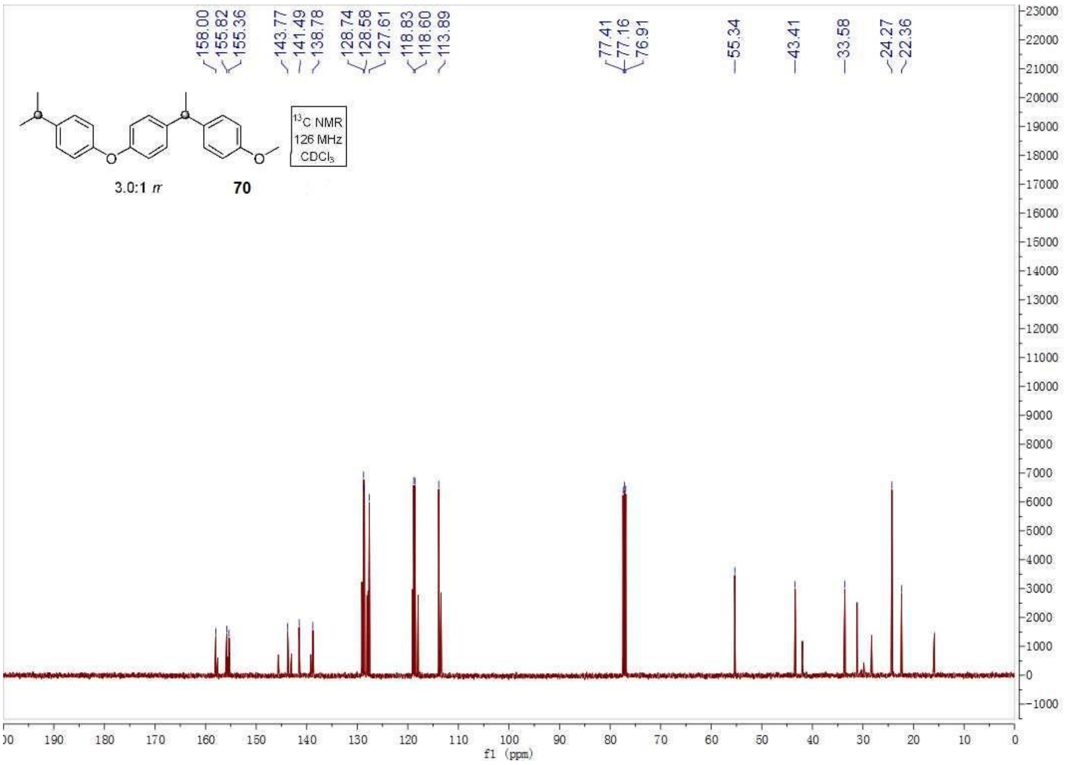

13C NMR
126 MHz
CDCl3
3.0:1 rr
70
158.00
155.82
155.36
143.77
141.49
138.78
128.74
128.58
127.61
118.83
118.60
113.89
77.41
77.16
76.91
55.34
43.41
33.58
24.27
22.36
f1 (ppm)

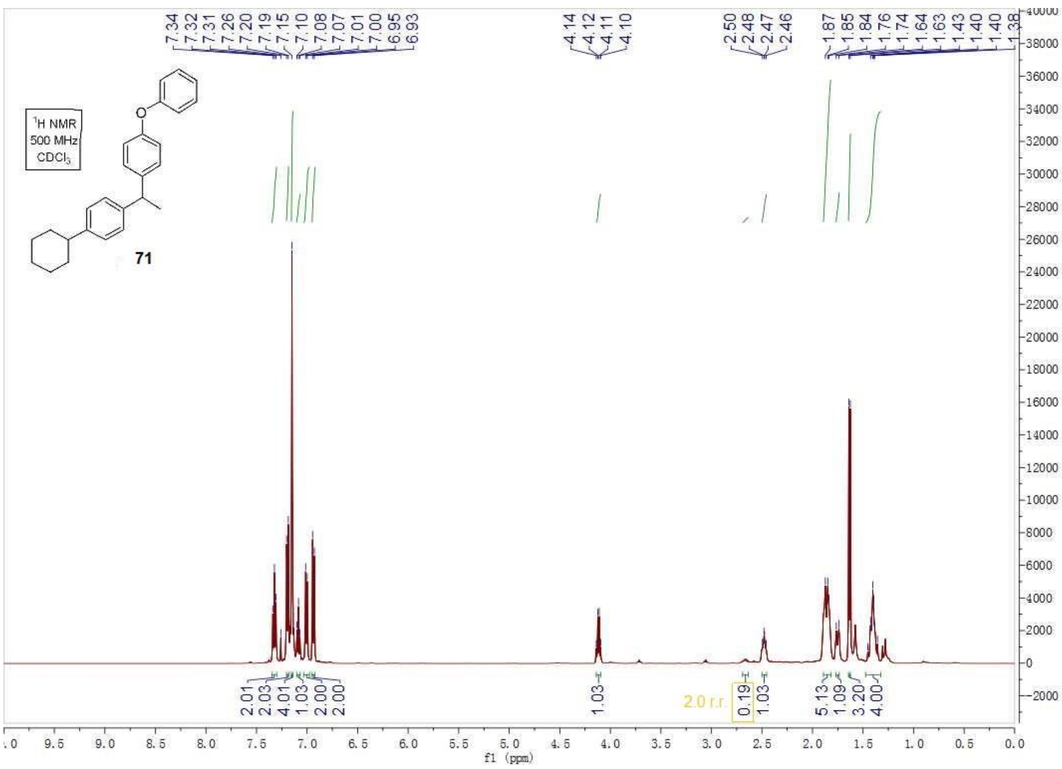

1H NMR
500 MHz
CDCl3
71
7.34 7.32 7.31 7.26 7.20 7.19 7.15 7.10 7.08 7.07 7.01 7.00 6.95 6.93
4.14 4.12 4.11 4.10
2.50 2.48 2.47 2.46
1.87 1.85 1.84 1.76 1.74 1.64 1.63 1.43 1.40 1.40 1.38
2.01 2.03 4.01 1.03 2.00 2.00
1.03
2.0 r.r.
0.19 1.03
5.13 1.09 3.20 4.00
f1 (ppm)

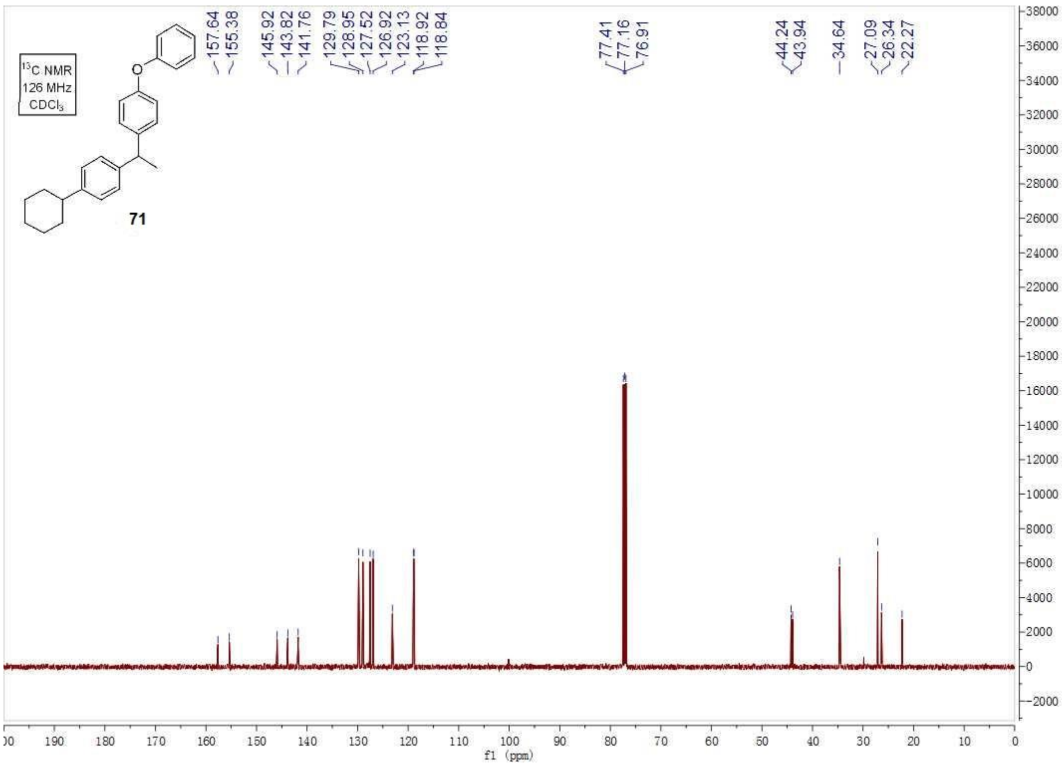

13C NMR
126 MHz
CDCl3
71
157.64 155.38
145.92 143.82 141.76
129.79 128.95 127.52 126.92 123.13 118.92 118.84
77.41 77.16 76.91
44.24 43.94
34.64
27.09 26.34 22.27
f1 (ppm)

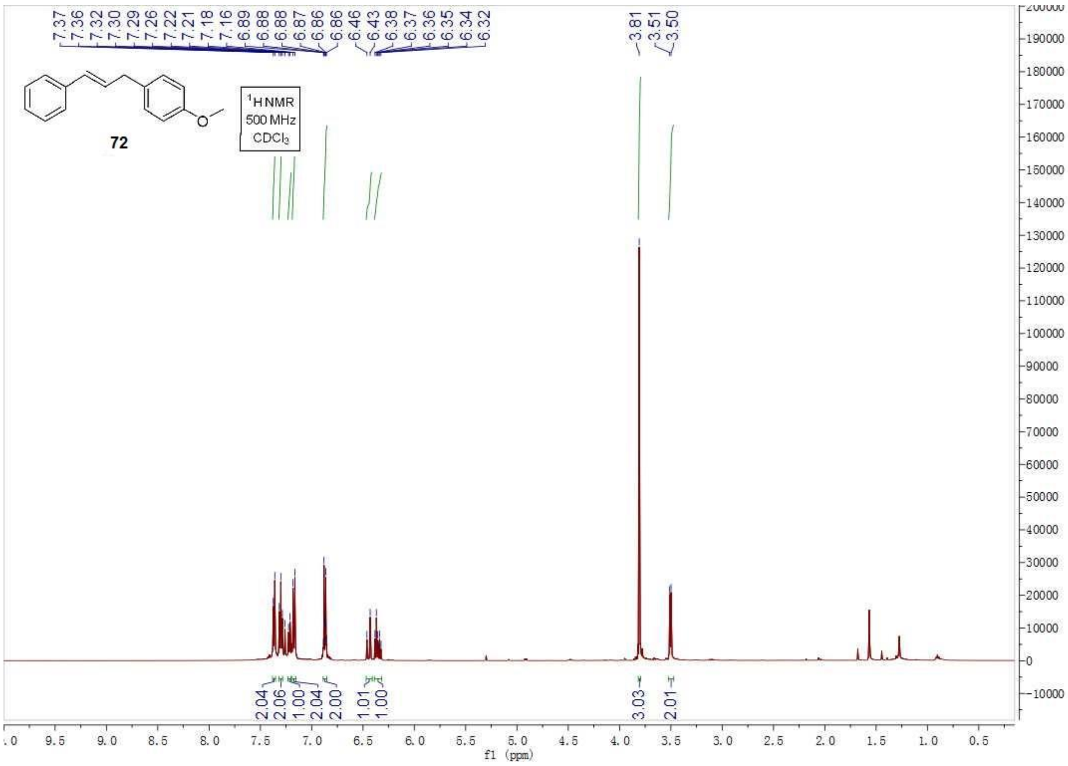
7.37
7.36
7.32
7.30
7.29
7.26
7.22
7.21
7.18
7.16
6.89
6.88
6.88
6.87
6.86
6.86
6.46
6.43
6.38
6.37
6.36
6.35
6.34
6.32
3.81
3.51
3.50
72
1H NMR
500 MHz
CDCl3
2.04
2.06
1.00
2.04
2.00
1.01
1.00
3.03
2.01
f1 (ppm)

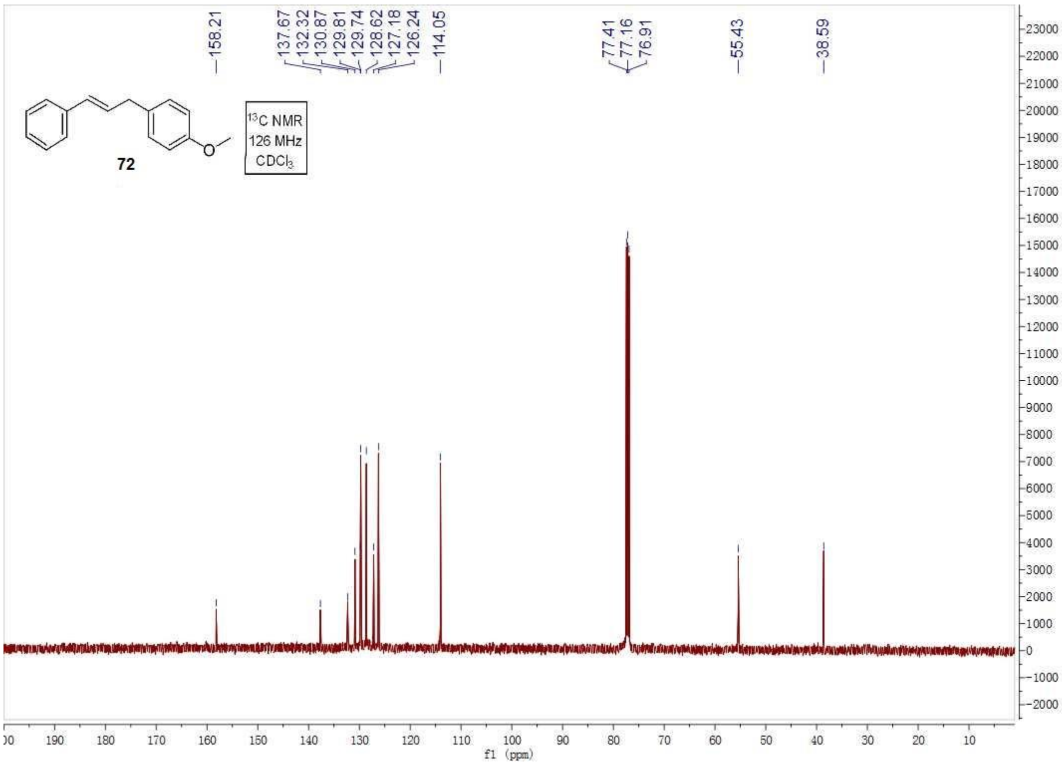
158.21
137.67
132.32
130.87
129.81
129.74
128.62
127.18
126.24
114.05
77.41
77.16
76.91
55.43
38.59
72
13C NMR
126 MHz
CDCl3
f1 (ppm)

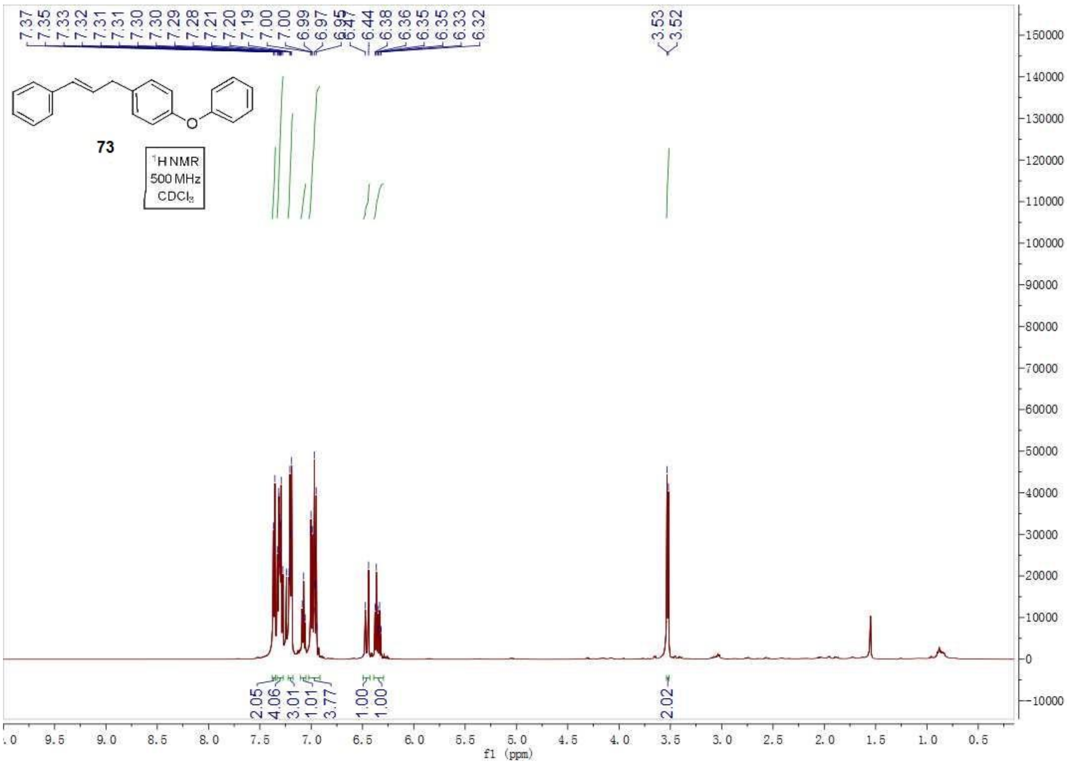
7.37
7.35
7.33
7.32
7.31
7.31
7.30
7.30
7.29
7.28
7.21
7.20
7.19
7.00
7.00
6.99
6.97
6.44
6.38
6.36
6.35
6.35
6.33
6.32
3.53
3.52
73
¹H NMR
500 MHz
CDCl₃
2.05
4.06
3.01
1.01
3.77
1.00
1.00
2.02
f1 (ppm)

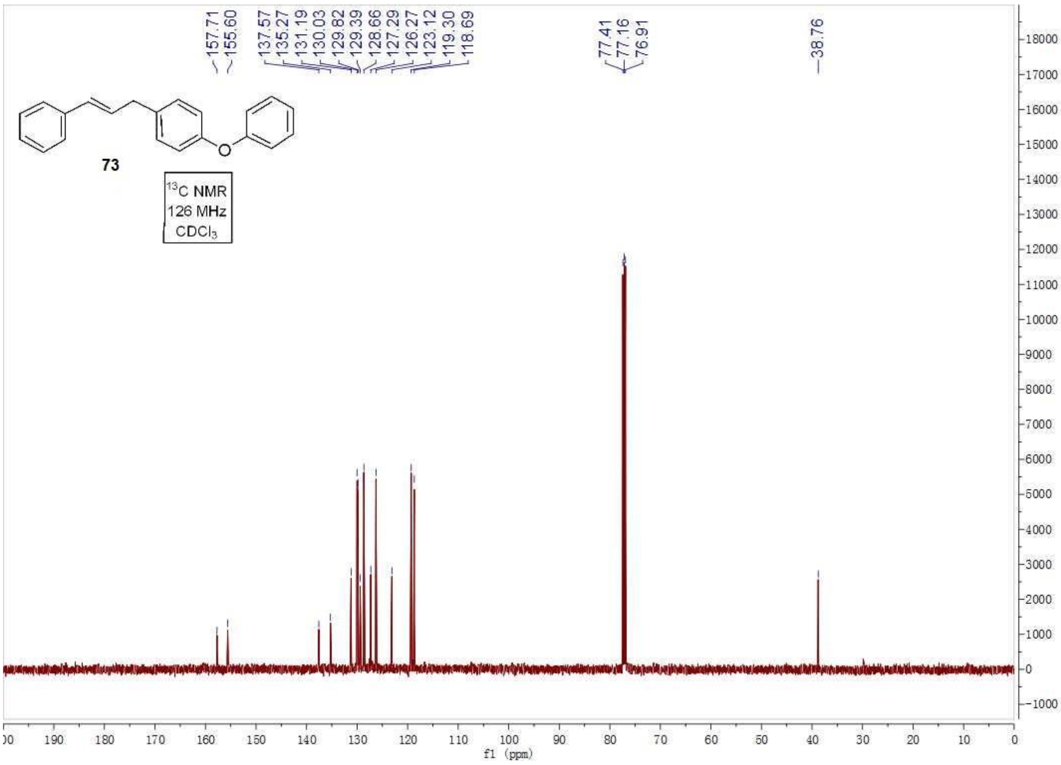
157.71
155.60
137.57
135.27
131.19
130.03
129.82
129.39
128.66
127.29
126.27
123.12
119.30
118.69
77.41
77.16
76.91
38.76
73
¹³C NMR
126 MHz
CDCl₃
f1 (ppm)

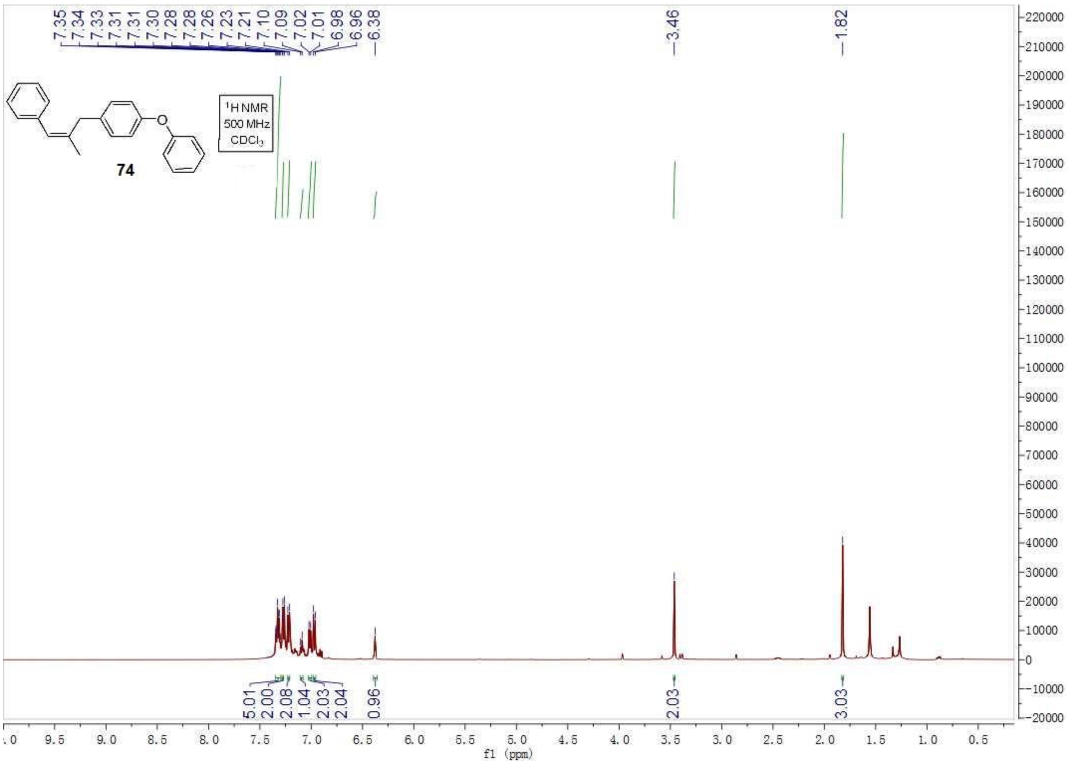

7.35
7.34
7.33
7.31
7.31
7.30
7.28
7.28
7.26
7.23
7.21
7.10
7.09
7.02
7.01
6.98
6.96
6.38
3.46
1.82
74
^{1}H NMR
500 MHz
CDCl$_3$
5.01
2.00
2.08
1.04
2.03
2.04
0.96
2.03
3.03
f1 (ppm)

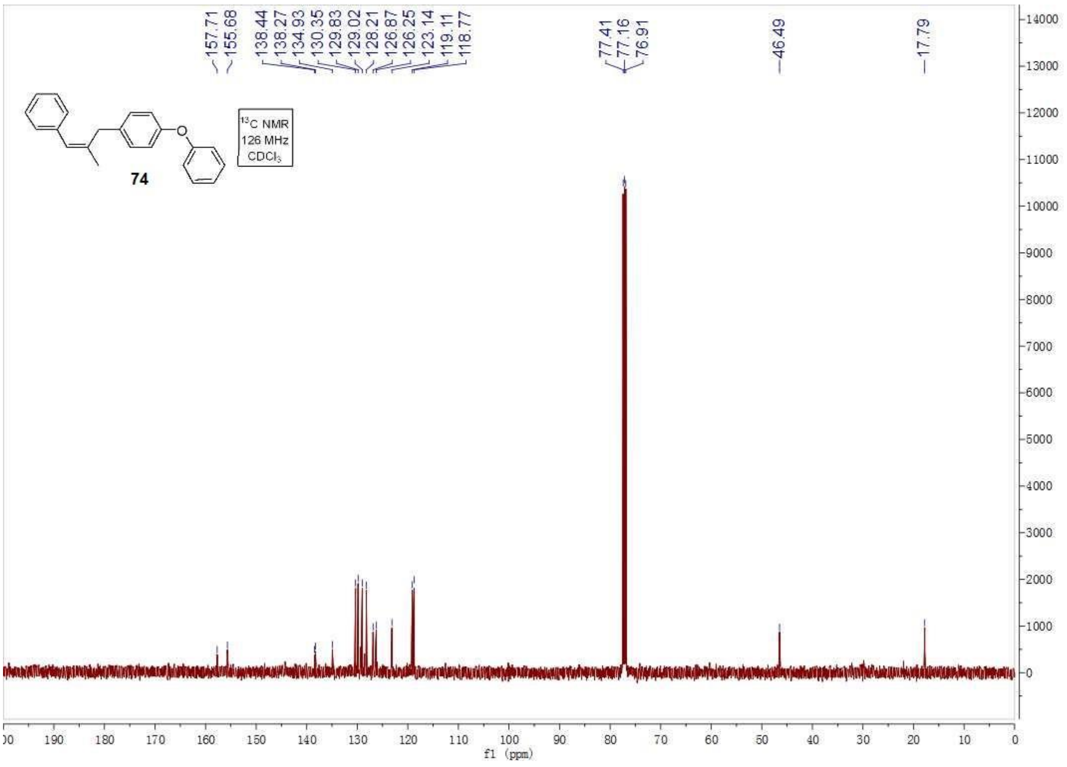

157.71
155.68
138.44
138.27
134.93
130.35
129.83
129.02
128.21
126.87
126.25
123.14
119.11
118.77
77.41
77.16
76.91
46.49
17.79
74
^{13}C NMR
126 MHz
CDCl$_3$
f1 (ppm)

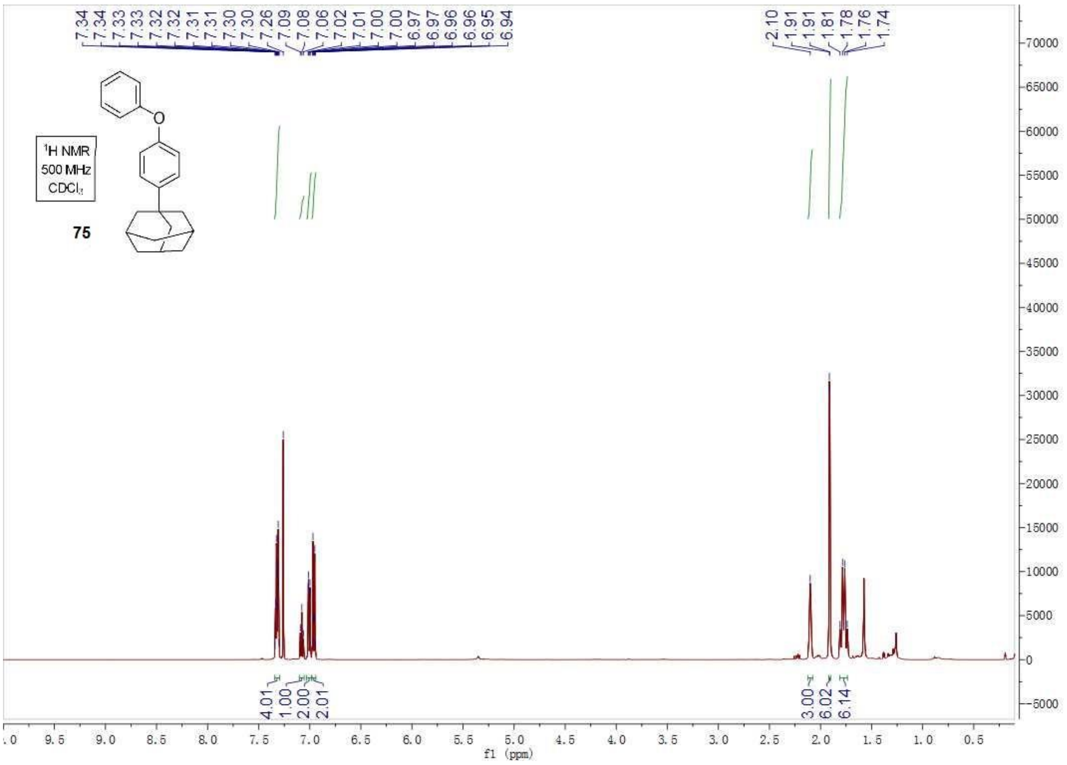
7.34
7.34
7.33
7.33
7.32
7.32
7.31
7.31
7.30
7.30
7.26
7.09
7.08
7.06
7.02
7.01
7.00
6.97
6.96
6.96
6.95
6.94
2.10
1.91
1.91
1.81
1.78
1.76
1.74
^{1}H NMR
500 MHz
CDCl3
75
O
4.01
1.00
2.00
2.01
3.00
6.02
6.14
f1 (ppm)

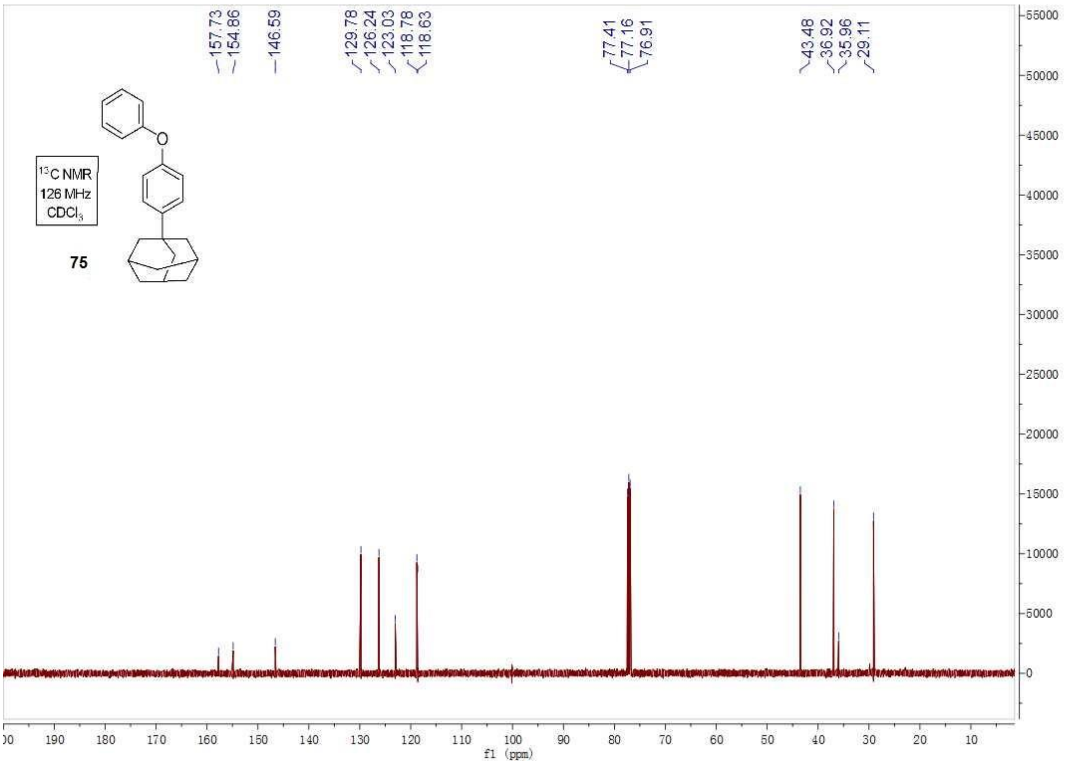
157.73
154.86
146.59
129.78
126.24
123.03
118.78
118.63
77.41
77.16
76.91
43.48
36.92
35.96
29.11
^{13}C NMR
126 MHz
CDCl3
75
O
f1 (ppm)

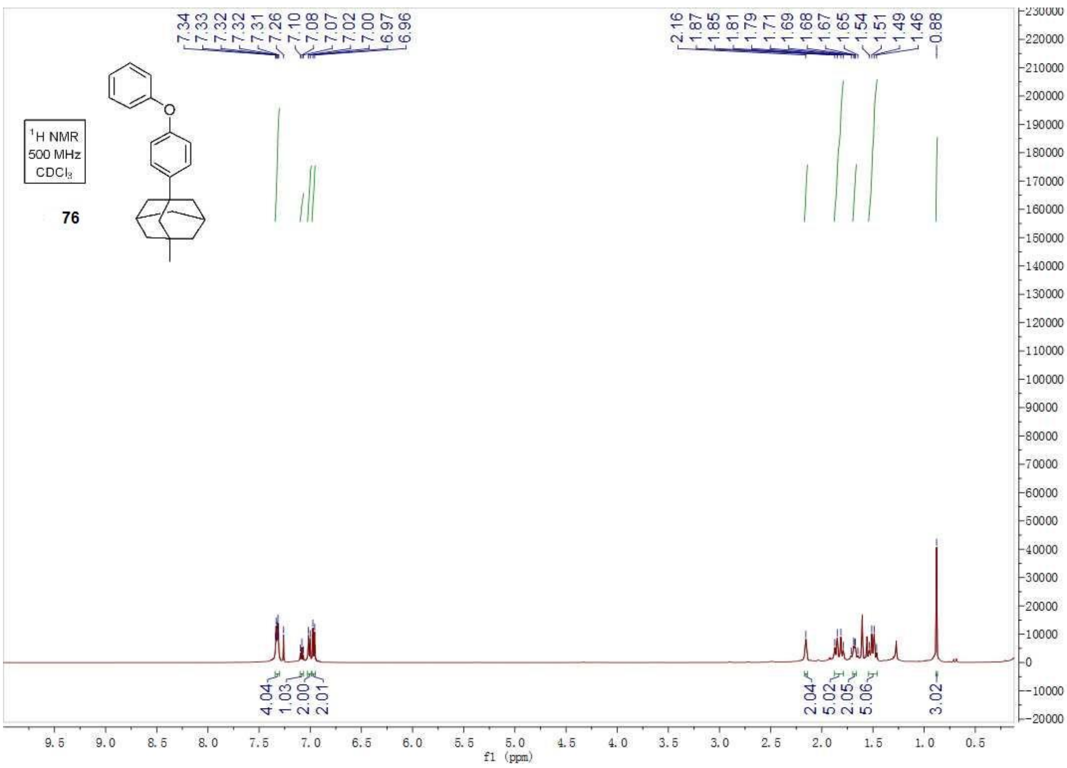

7.34
7.33
7.32
7.32
7.31
7.26
7.10
7.08
7.07
7.02
7.00
6.97
6.96
2.16
1.87
1.85
1.81
1.79
1.71
1.69
1.68
1.67
1.65
1.54
1.51
1.49
1.46
0.88
1H NMR
500 MHz
CDCl3
76
4.04
1.03
2.00
2.01
2.04
5.02
2.05
5.06
3.02
f1 (ppm)

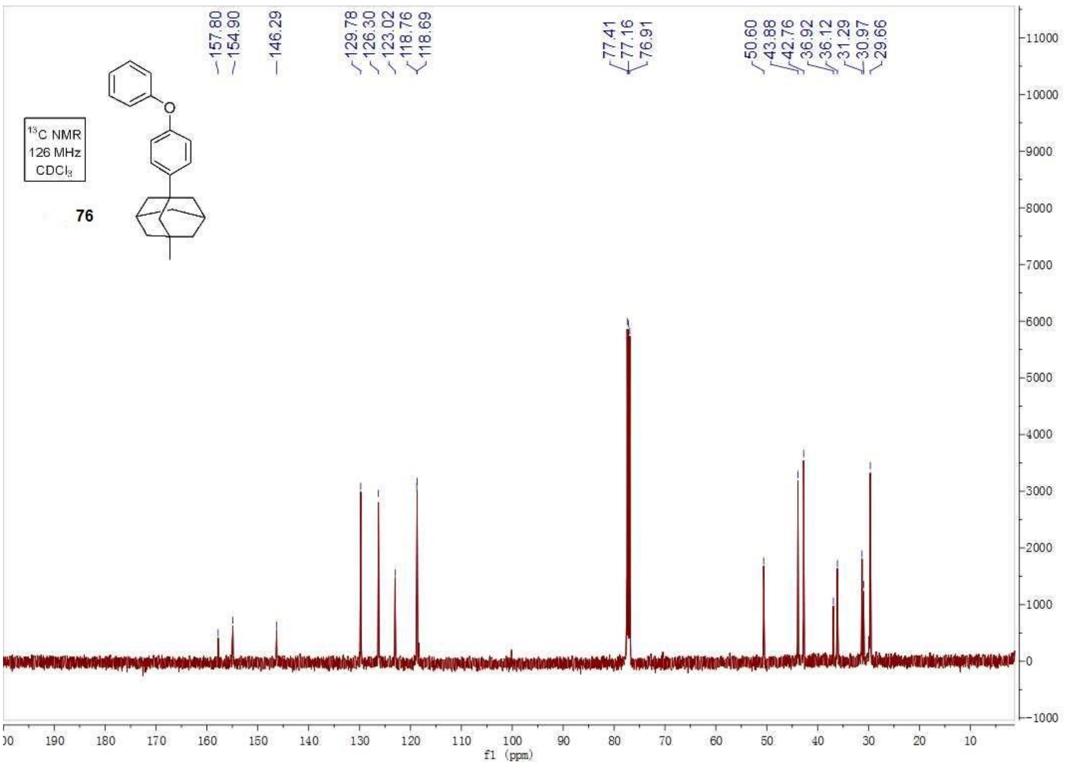

157.80
154.90
146.29
129.78
126.30
123.02
118.76
118.69
77.41
77.16
76.91
50.60
43.88
42.76
36.92
36.12
31.29
30.97
29.66
13C NMR
126 MHz
CDCl3
76
f1 (ppm)

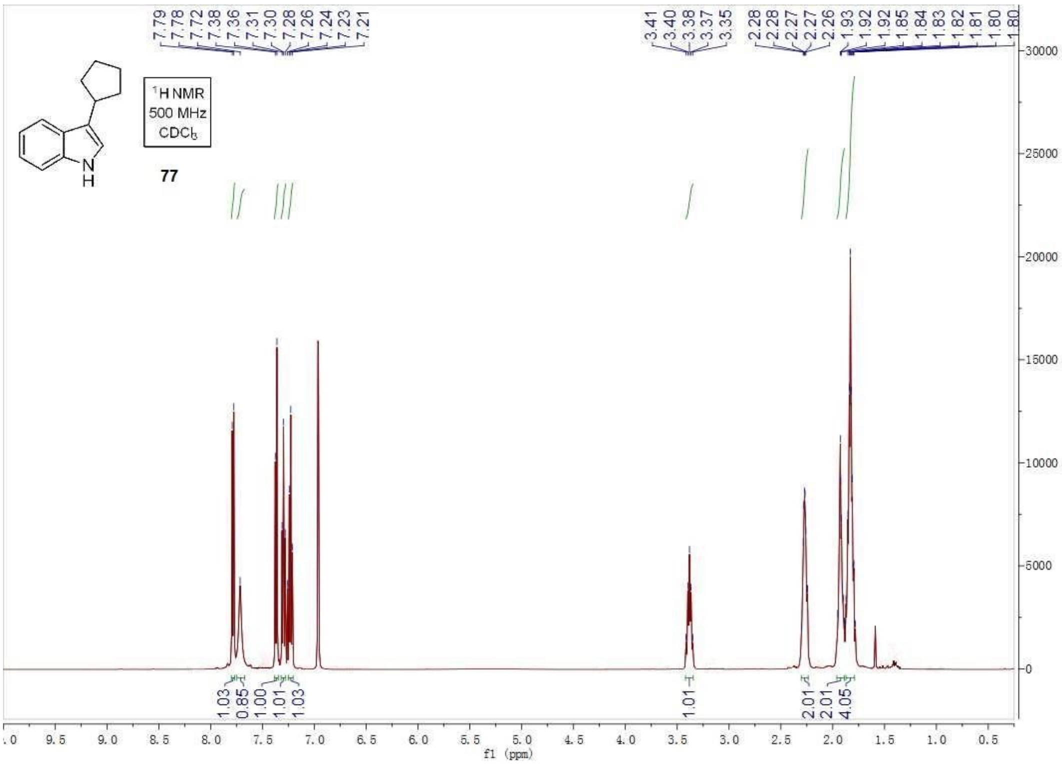
¹H NMR
500 MHz
CDCl₃
77
f1 (ppm)

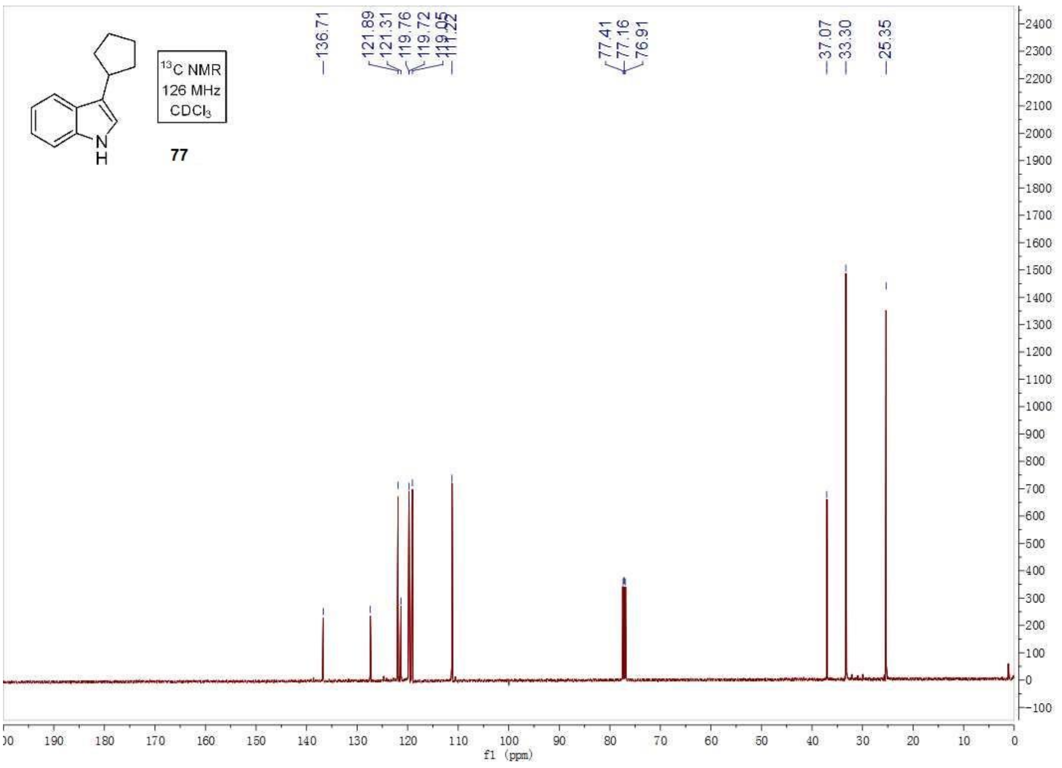
¹³C NMR
126 MHz
CDCl₃
77
f1 (ppm)

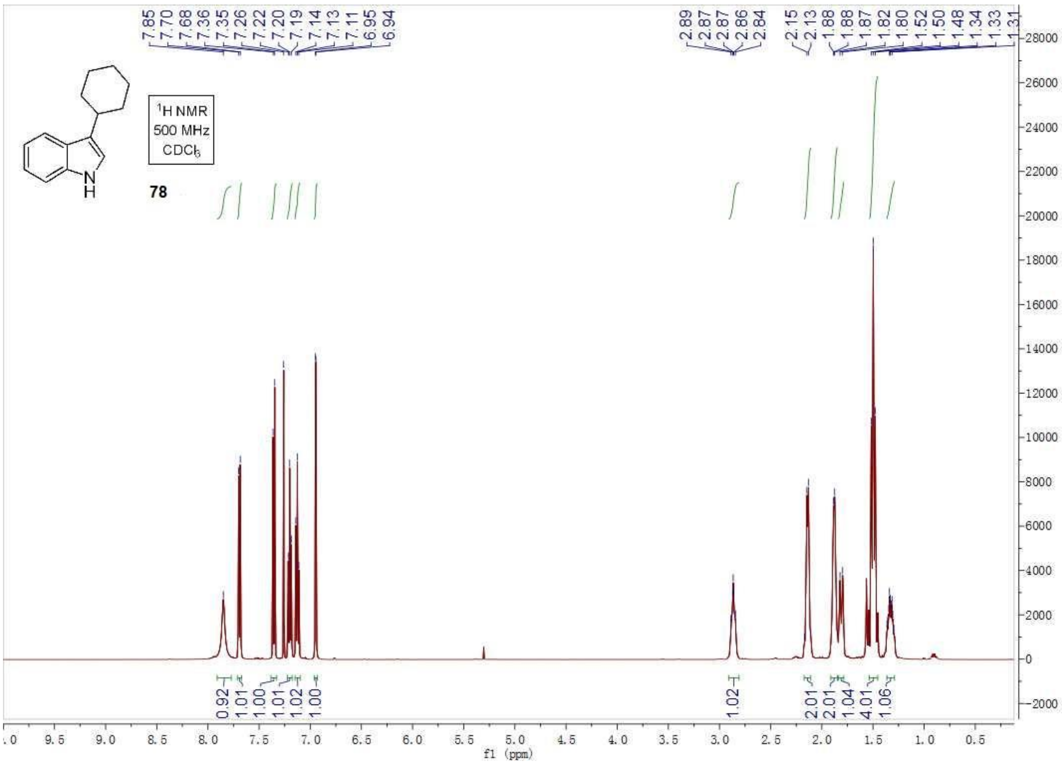
7.85 7.70 7.68 7.36 7.35 7.26 7.22 7.20 7.19 7.14 7.13 7.11 6.95 6.94
2.89 2.87 2.87 2.86 2.84 2.15 2.13 1.88 1.88 1.87 1.82 1.80 1.52 1.50 1.48 1.34 1.33 1.31
^{1}H NMR
500 MHz
CDCl$_3$
78
0.92 1.01 1.00 1.01 1.02 1.00
1.02
2.01 2.01 1.04 4.01 1.06
f1 (ppm)

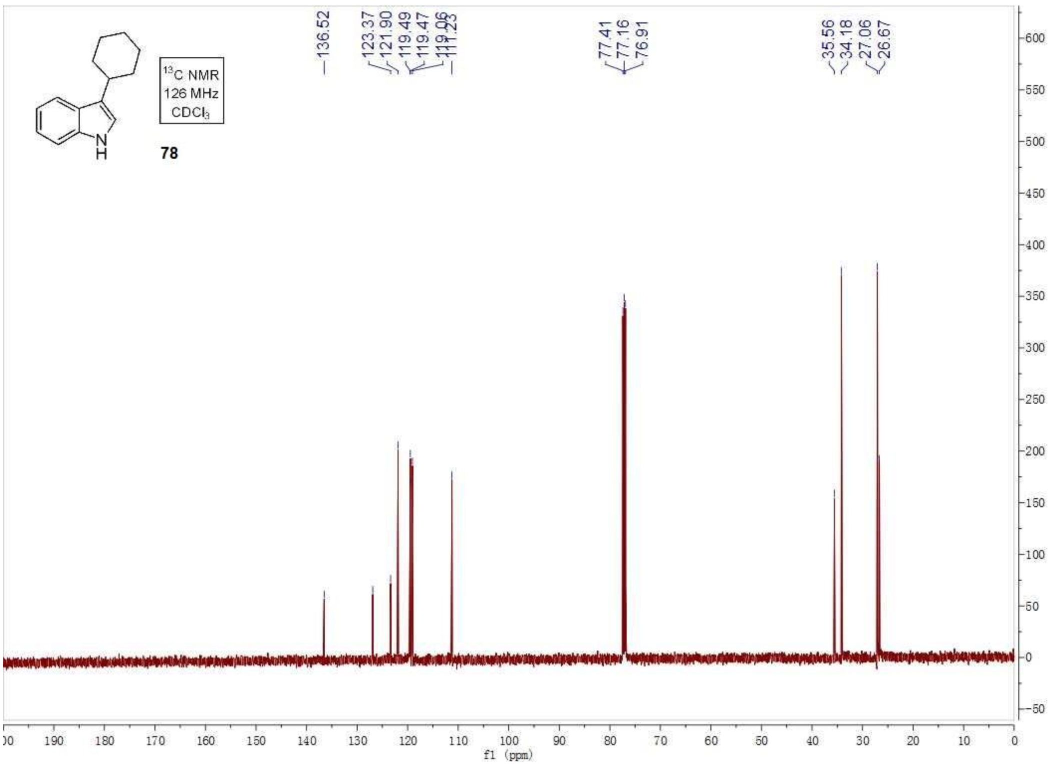
136.52
123.37 121.90 119.49 119.47 119.06 111.23
77.41 77.16 76.91
35.56 34.18 27.06 26.67
^{13}C NMR
126 MHz
CDCl$_3$
78
f1 (ppm)

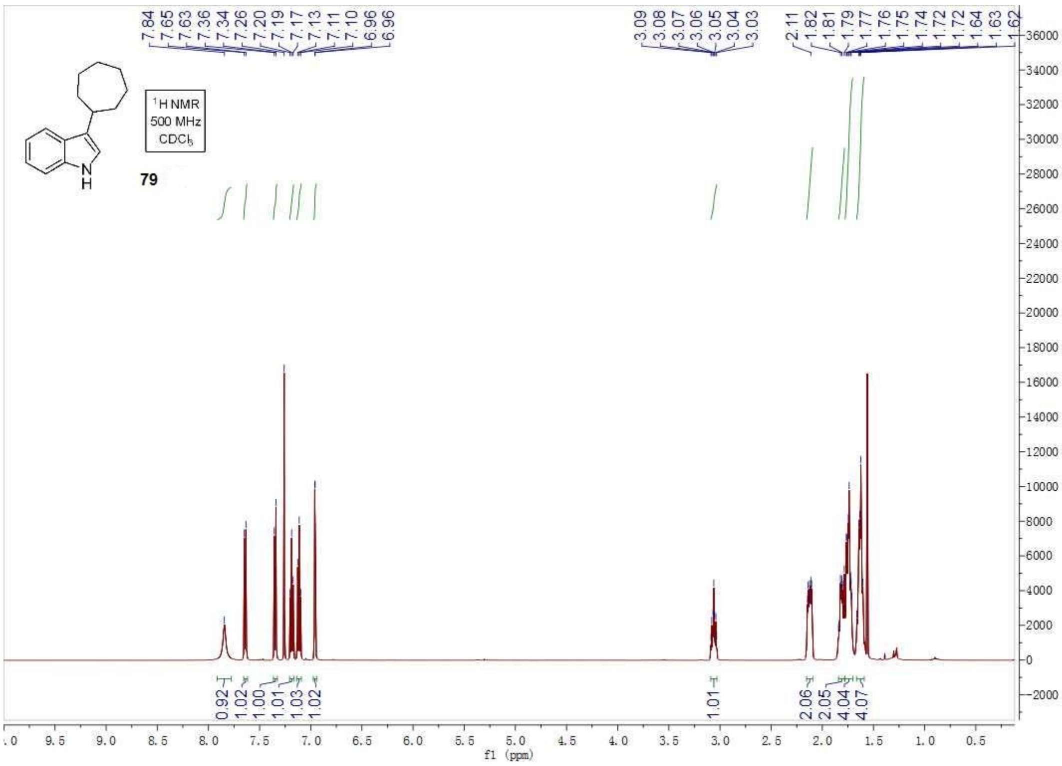
7.84 7.65 7.63 7.36 7.34 7.26 7.20 7.19 7.17 7.13 7.11 7.10 6.96 6.96
3.09 3.08 3.07 3.06 3.05 3.04 3.03
2.11 1.82 1.81 1.79 1.77 1.76 1.75 1.74 1.72 1.72 1.64 1.63 1.62
1H NMR
500 MHz
CDCl3
79
0.92 1.02 1.00 1.01 1.03 1.02
1.01
2.06 2.05 4.04 4.07
f1 (ppm)

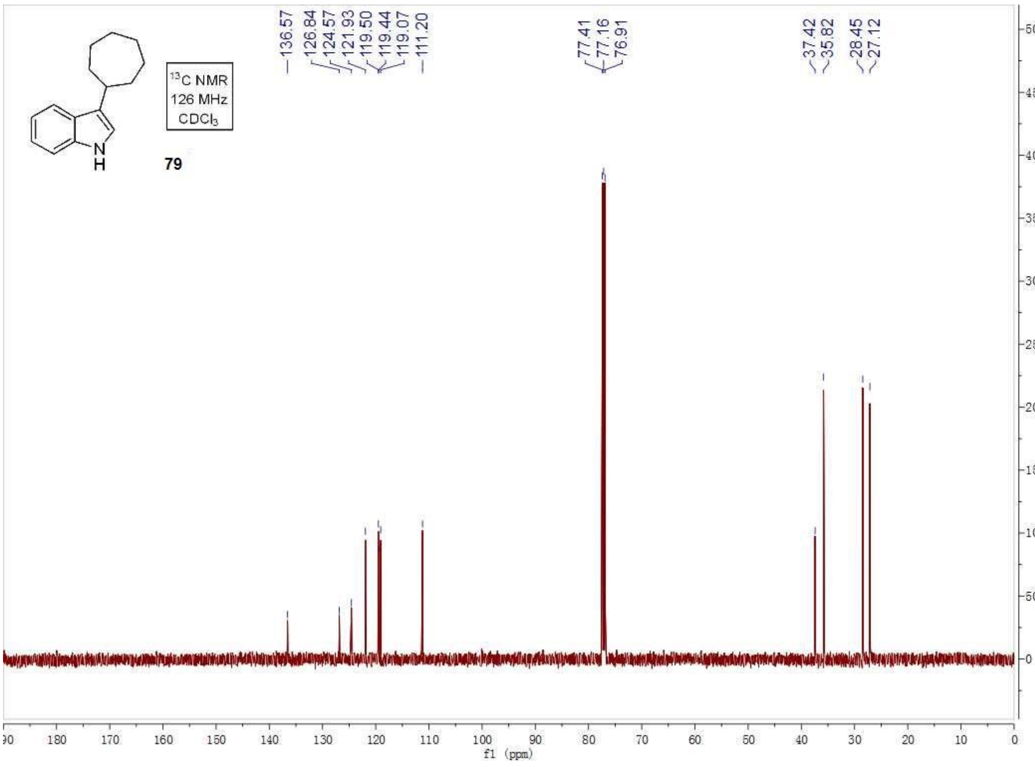
136.57 126.84 124.57 121.93 119.50 119.44 119.07 111.20
77.41 77.16 76.91
37.42 35.82 28.45 27.12
13C NMR
126 MHz
CDCl3
79
f1 (ppm)

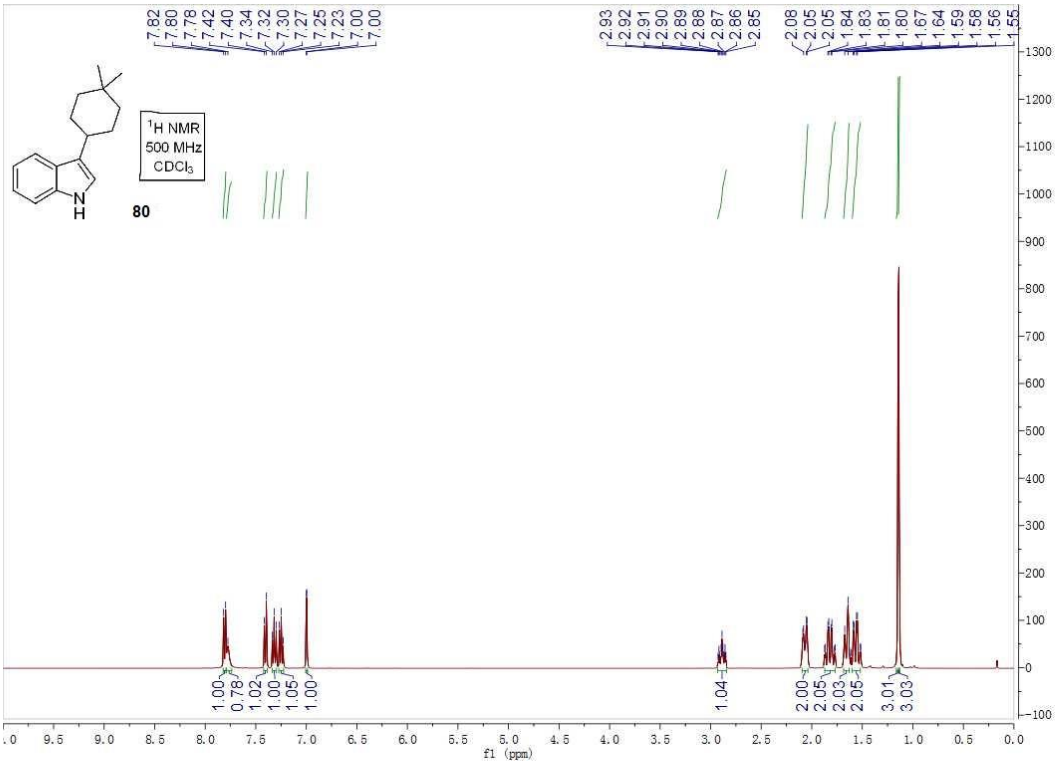
7.82 7.80 7.78 7.42 7.40 7.34 7.32 7.30 7.27 7.25 7.23 7.00 7.00
2.93 2.92 2.91 2.90 2.89 2.88 2.87 2.86 2.85
2.08 2.05 2.05 1.84 1.83 1.81 1.80 1.67 1.64 1.59 1.58 1.56 1.55
1H NMR
500 MHz
CDCl3
N
H
80
1.00 0.78 1.02 1.00 1.05 1.00
1.04
2.00 2.05 2.03 2.05 3.01 3.03
f1 (ppm)

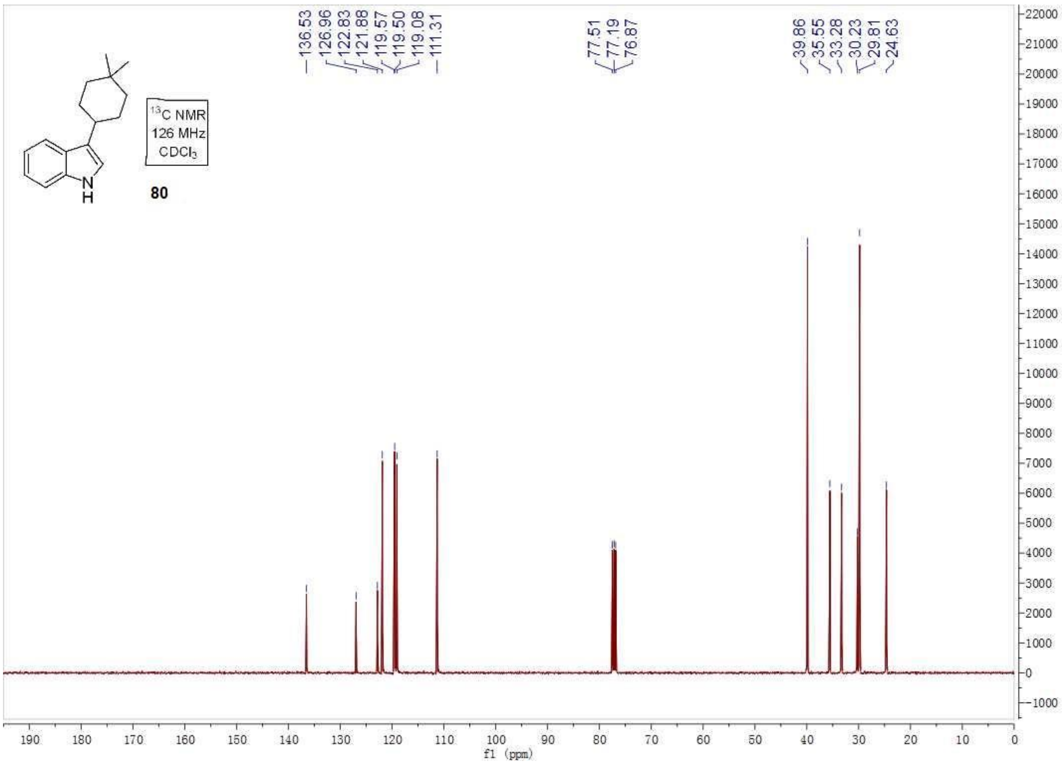
136.53 126.96 122.83 121.88 119.57 119.50 119.08 111.31
77.51 77.19 76.87
39.86 35.55 33.28 30.23 29.81 24.63
13C NMR
126 MHz
CDCl3
N
H
80
f1 (ppm)

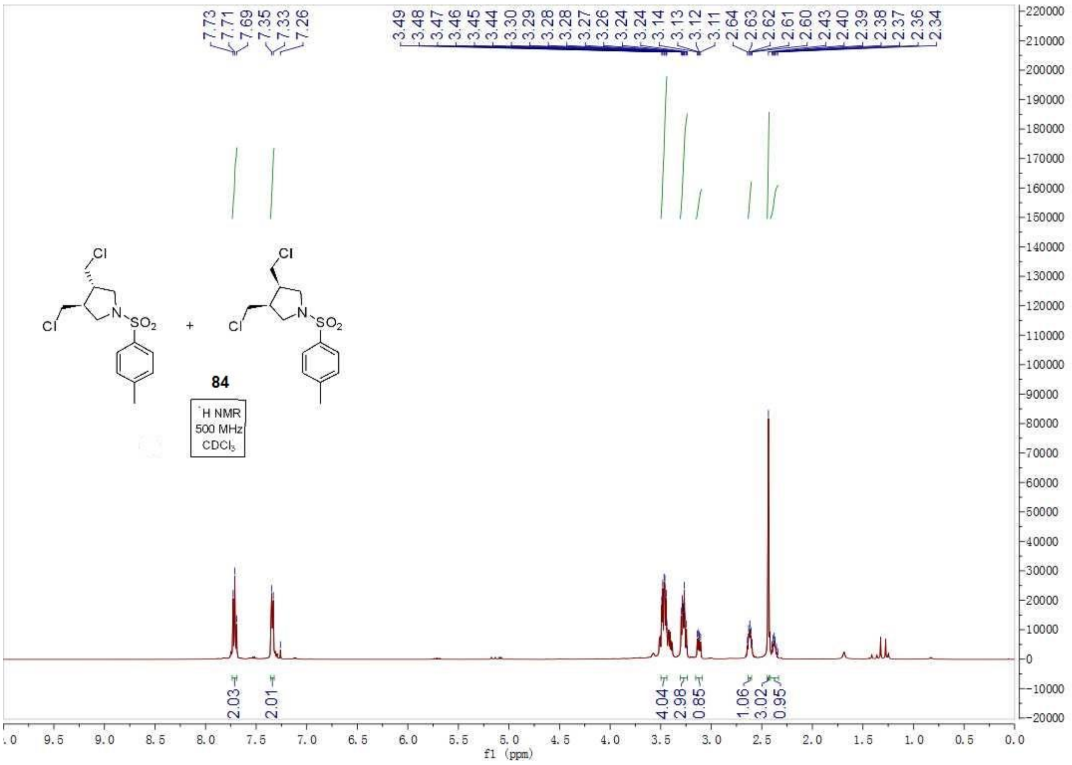

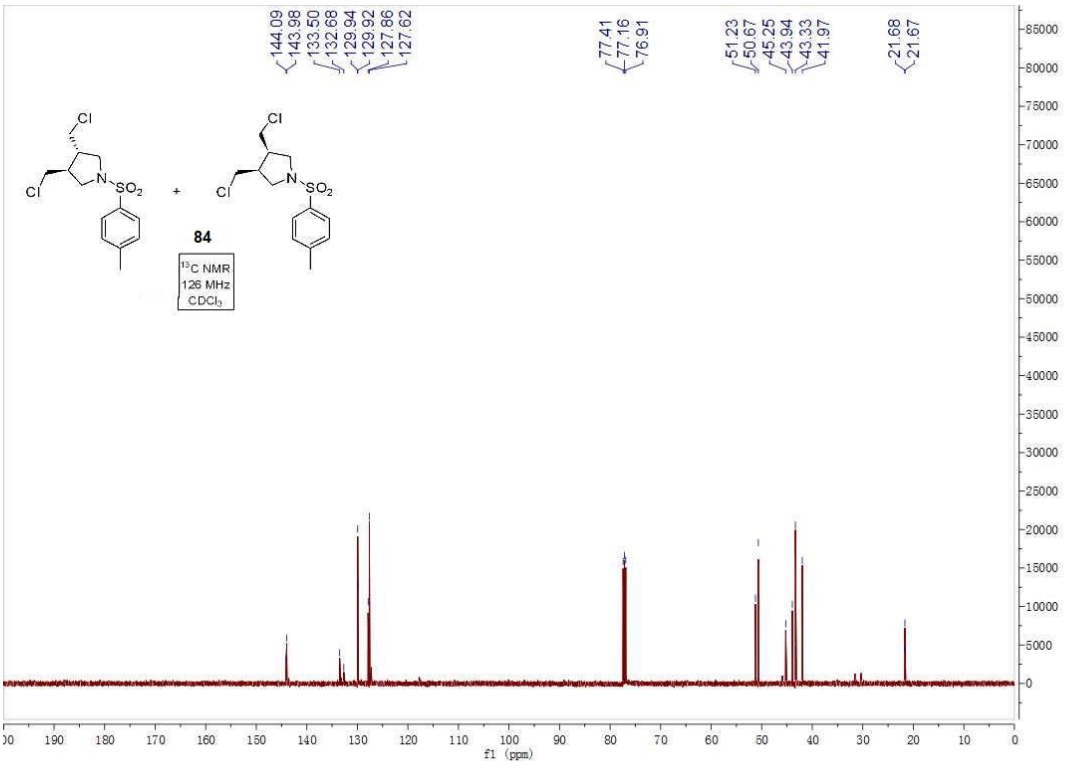

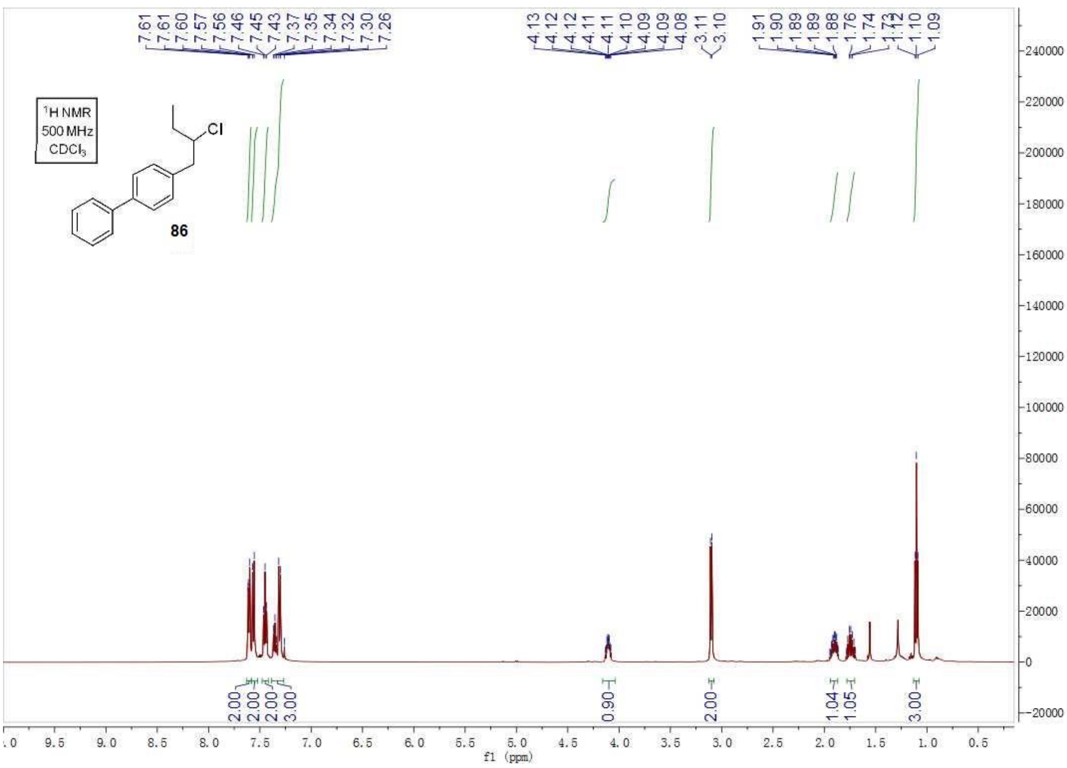

1H NMR
500 MHz
CDCl3
86
f1 (ppm)

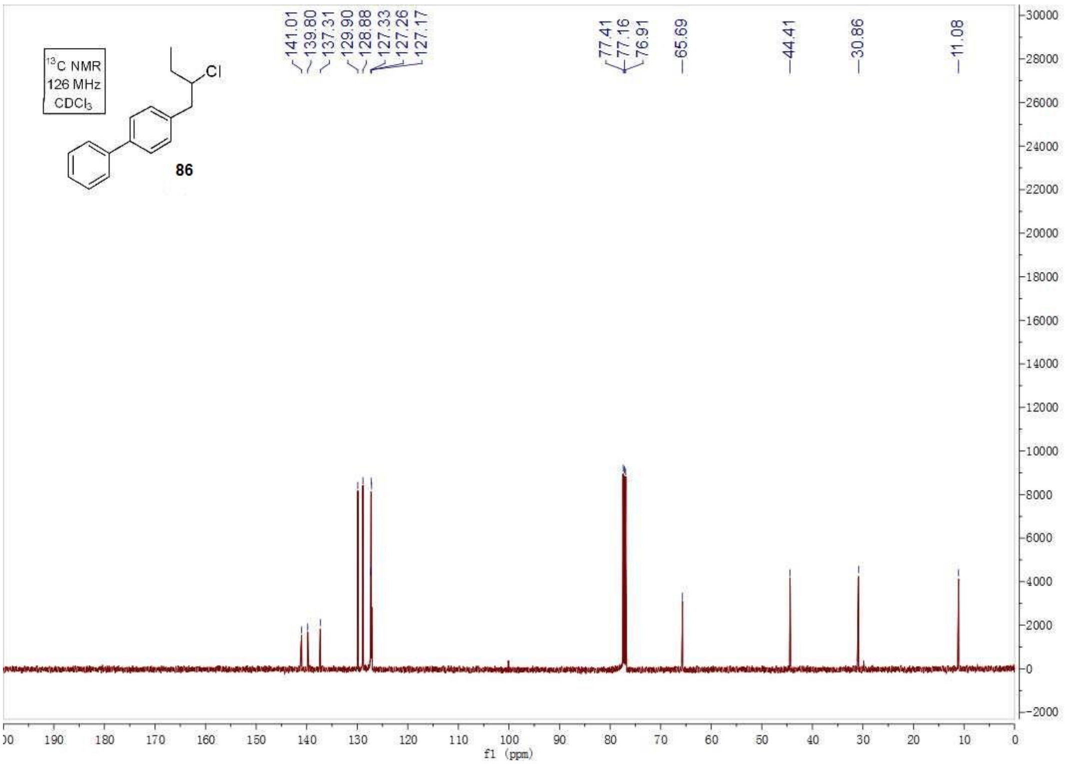

13C NMR
126 MHz
CDCl3
86
f1 (ppm)

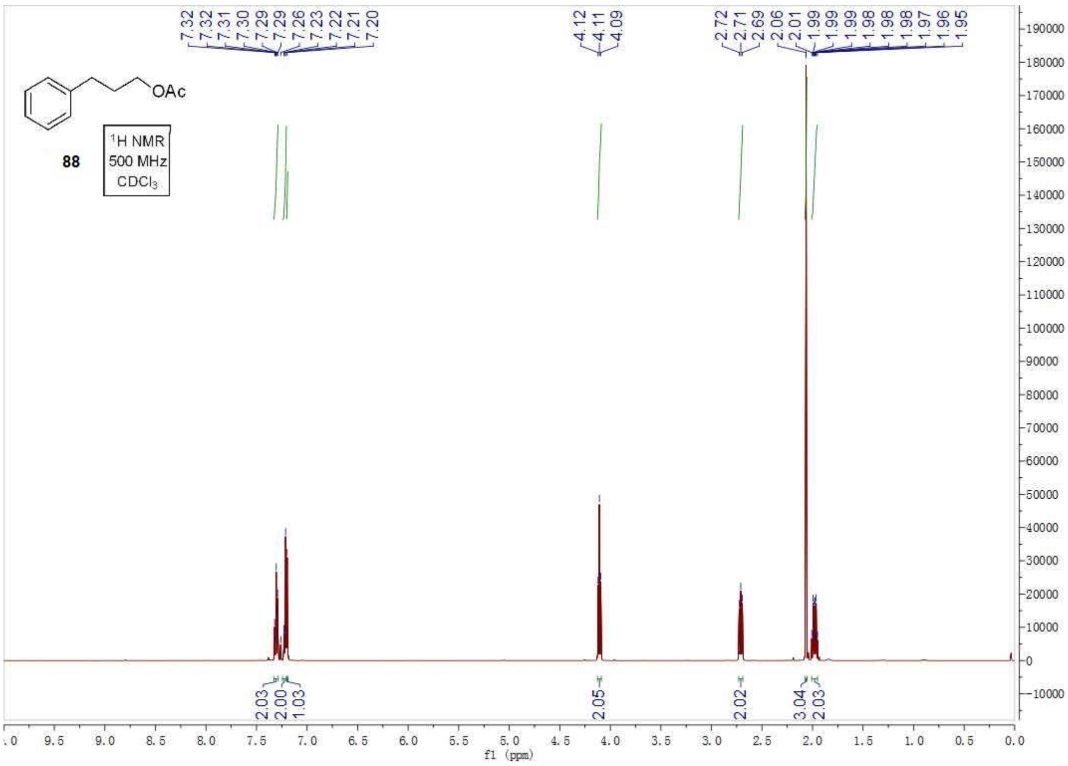
7.32
7.32
7.31
7.30
7.29
7.29
7.26
7.23
7.22
7.21
7.20
4.12
4.11
4.09
2.72
2.71
2.69
2.06
2.01
1.99
1.99
1.99
1.98
1.98
1.98
1.97
1.96
1.95
OAc
88
1H NMR
500 MHz
CDCl3
2.03
2.00
1.03
2.05
2.02
3.04
2.03
f1 (ppm)

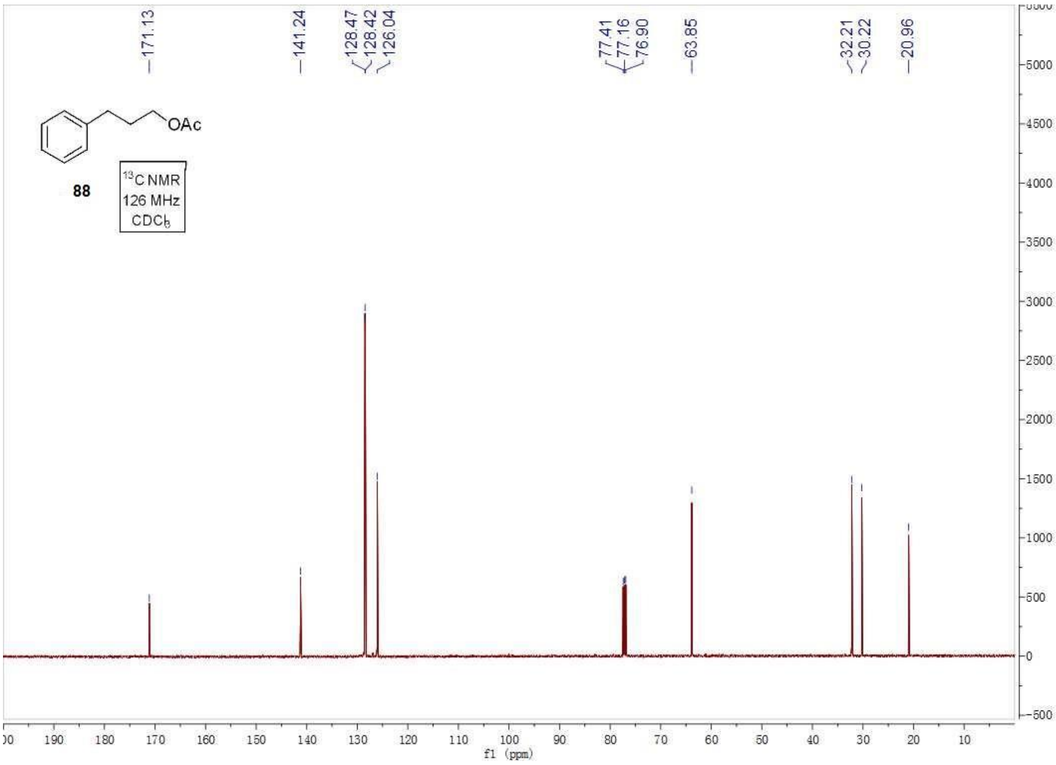
171.13
141.24
128.47
128.42
126.04
77.41
77.16
76.90
63.85
32.21
30.22
20.96
OAc
88
13C NMR
126 MHz
CDCl3
f1 (ppm)

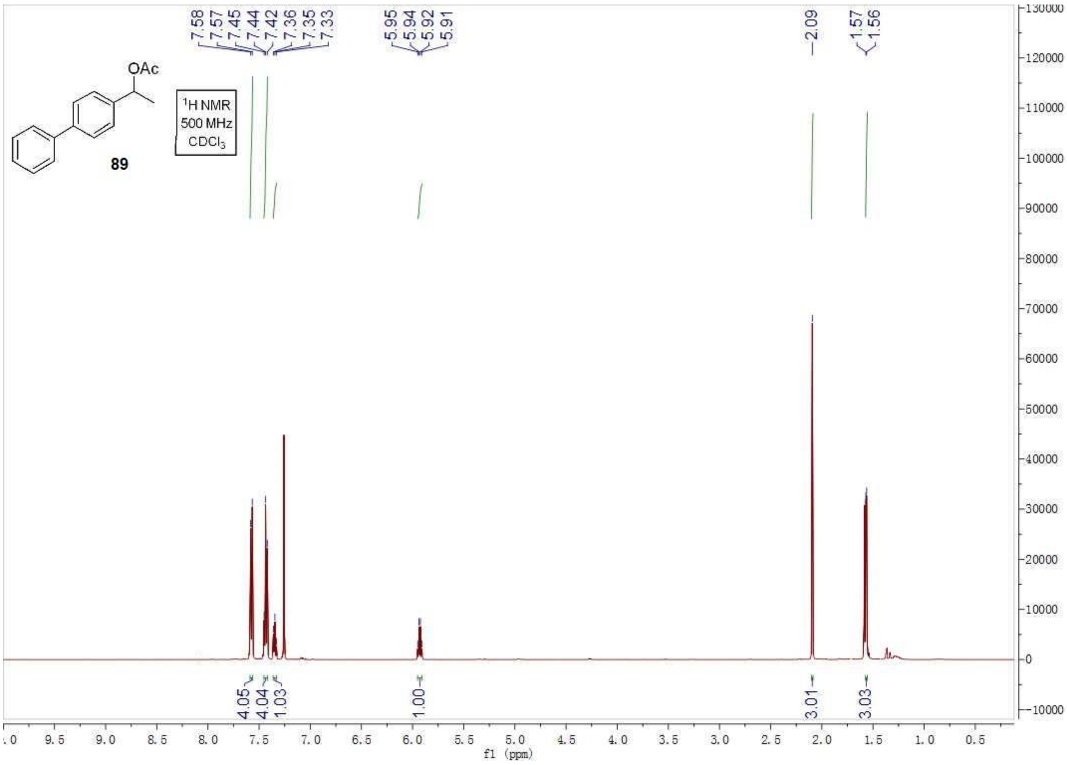
7.58
7.57
7.45
7.44
7.42
7.36
7.35
7.33
5.95
5.94
5.92
5.91
2.09
1.57
1.56
OAc
89
^{1}H NMR
500 MHz
CDCl3
4.05
4.04
1.03
1.00
3.01
3.03
f1 (ppm)

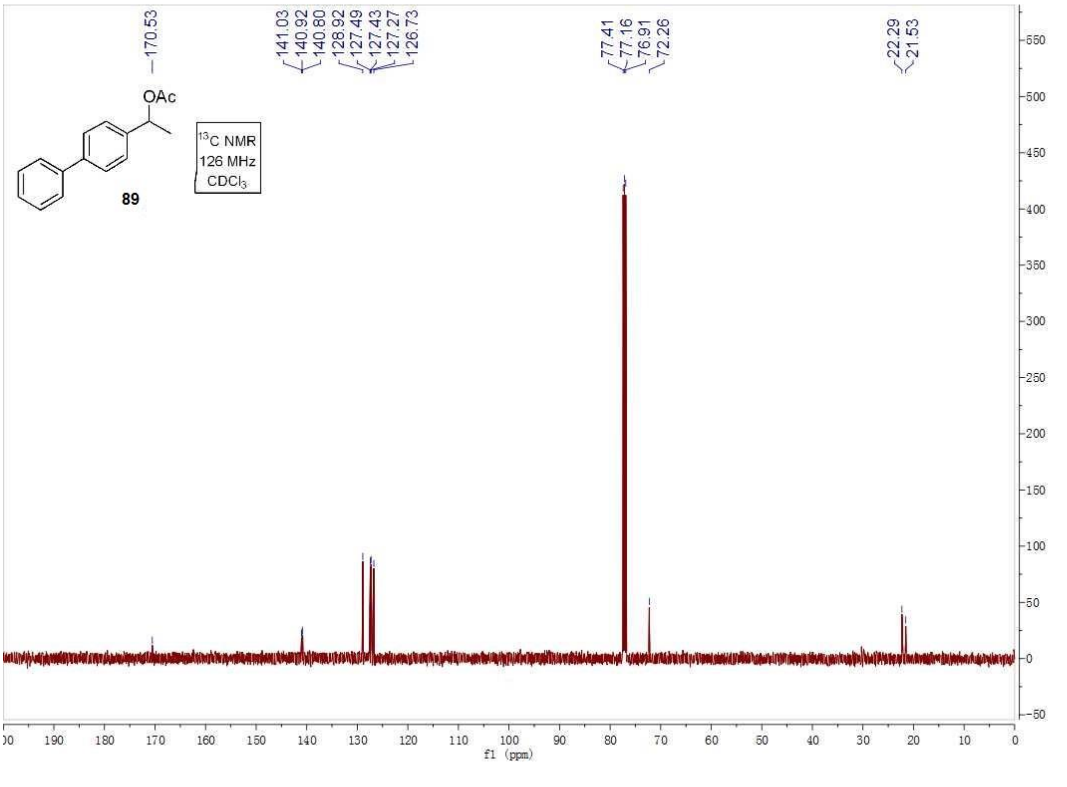
170.53
141.03
140.92
140.80
128.92
127.49
127.43
127.27
126.73
77.41
77.16
76.91
72.26
22.29
21.53
OAc
89
^{13}C NMR
126 MHz
CDCl3
f1 (ppm)

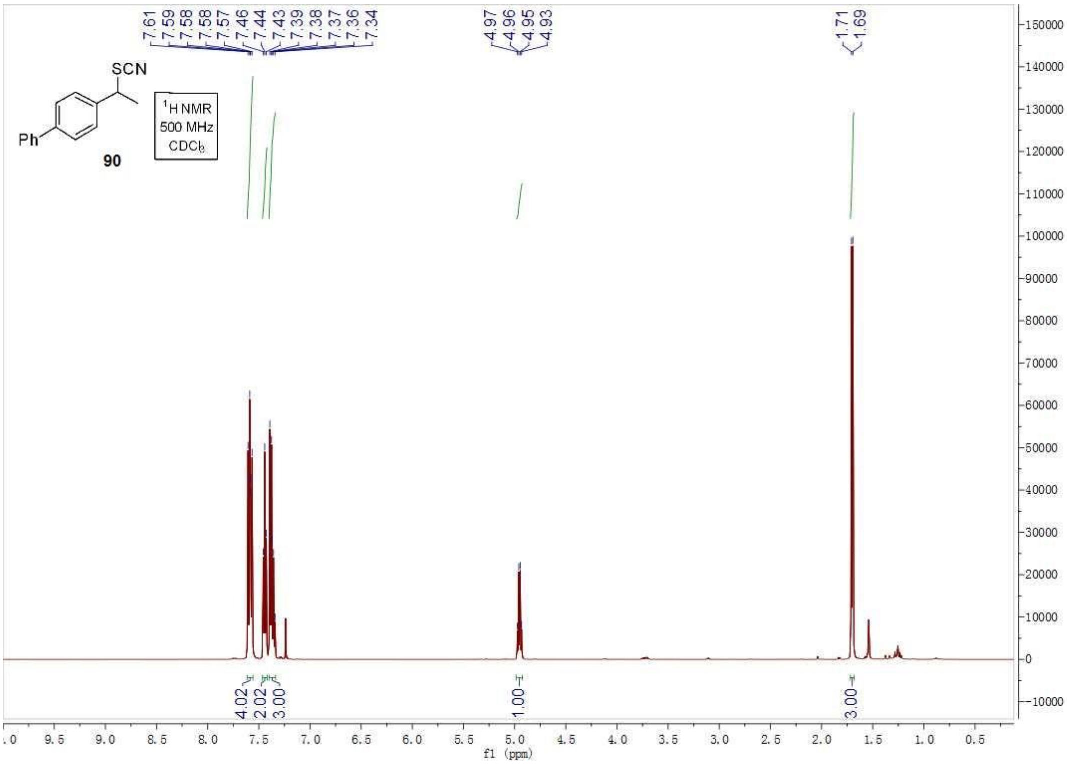
7.61
7.59
7.58
7.57
7.46
7.44
7.43
7.39
7.38
7.37
7.36
7.34
4.97
4.96
4.95
4.93
1.71
1.69
SCN
Ph
90
^{1}H NMR
500 MHz
CDCl$_3$
4.02
2.02
3.00
1.00
3.00
f1 (ppm)

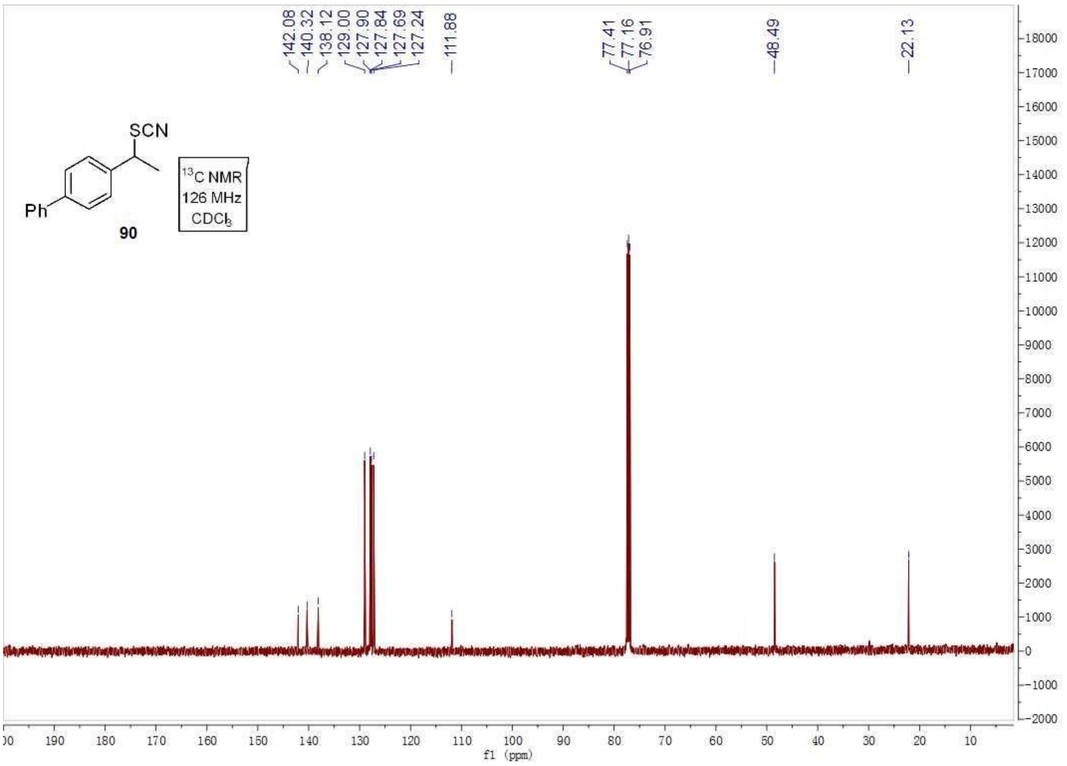
142.08
140.32
138.12
129.00
127.90
127.84
127.69
127.24
111.88
77.41
77.16
76.91
48.49
22.13
SCN
Ph
90
^{13}C NMR
126 MHz
CDCl$_3$
f1 (ppm)

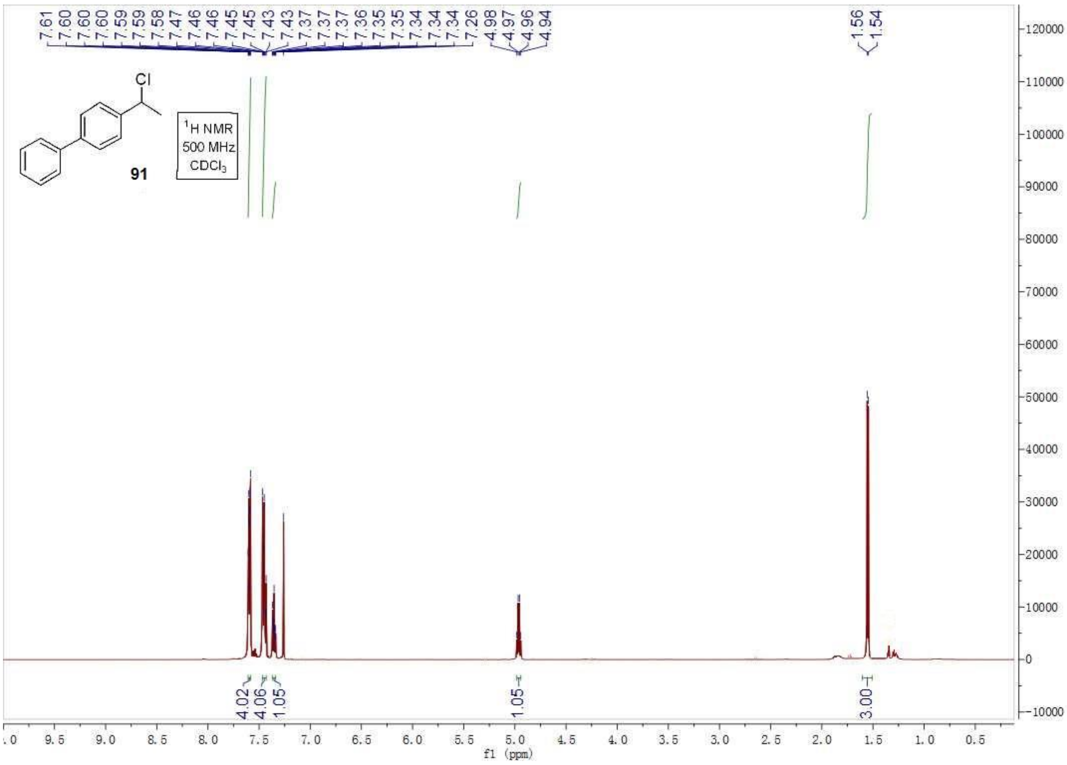
7.61
7.60
7.60
7.59
7.59
7.58
7.47
7.46
7.45
7.45
7.43
7.43
7.37
7.37
7.36
7.35
7.35
7.34
7.34
7.34
7.26
4.98
4.97
4.96
4.94
1.56
1.54
Cl
91
^{1}H NMR
500 MHz
$CDCl_3$
4.02
4.06
1.05
1.05
3.00
f1 (ppm)

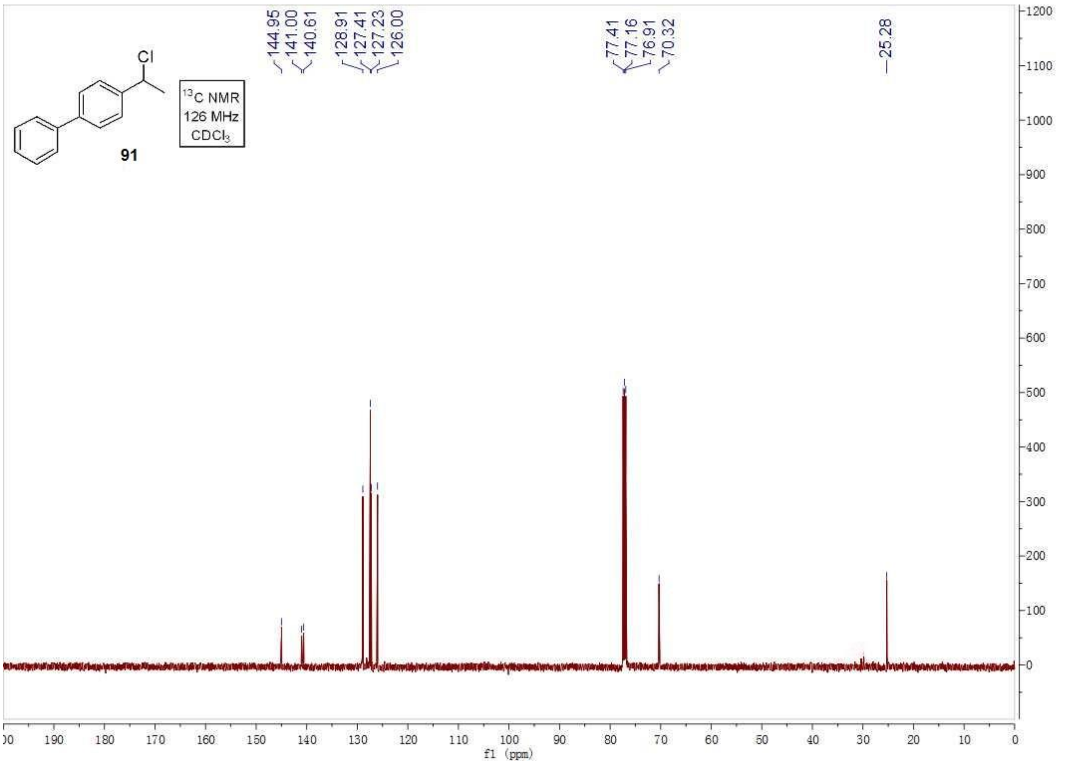
144.95
141.00
140.61
128.91
127.41
127.23
126.00
77.41
77.16
76.91
70.32
25.28
Cl
91
^{13}C NMR
126 MHz
$CDCl_3$
f1 (ppm)

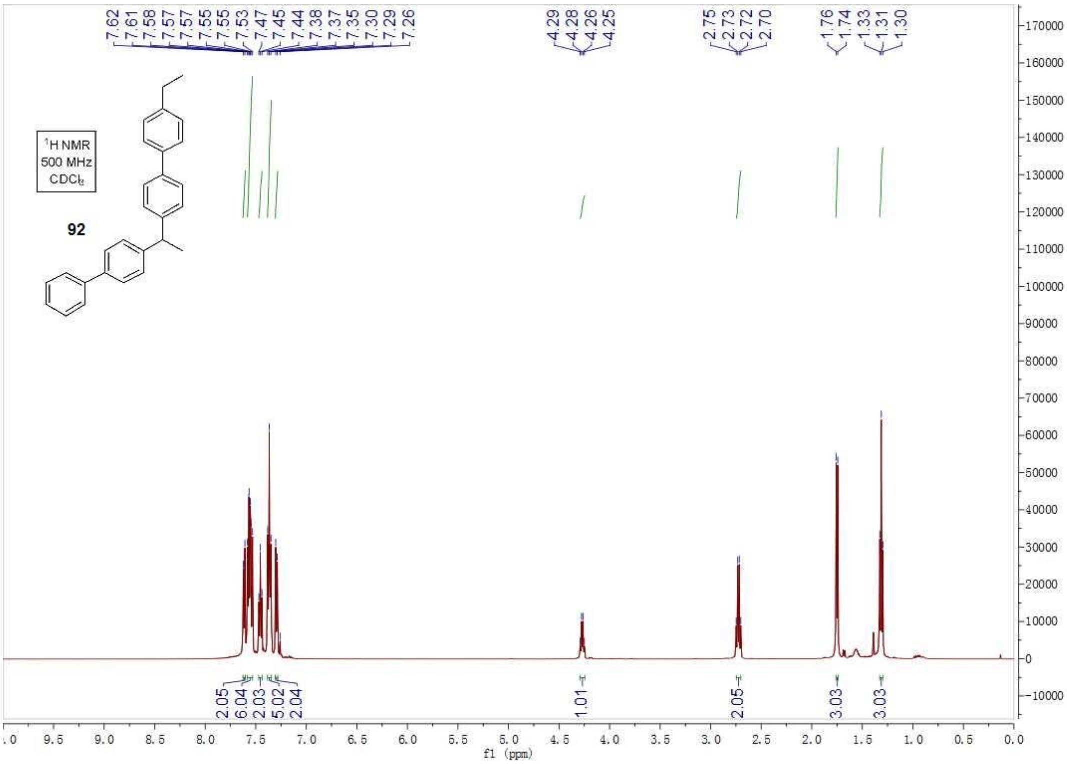

7.62 7.61 7.58 7.57 7.57 7.55 7.55 7.53 7.47 7.45 7.44 7.38 7.37 7.35 7.30 7.29 7.26
4.29 4.28 4.26 4.25
2.75 2.73 2.72 2.70
1.76 1.74 1.33 1.31 1.30
^{1}H NMR
500 MHz
CDCl$_3$
92
2.05 6.04 2.03 5.02 2.04
1.01
2.05
3.03
3.03
f1 (ppm)

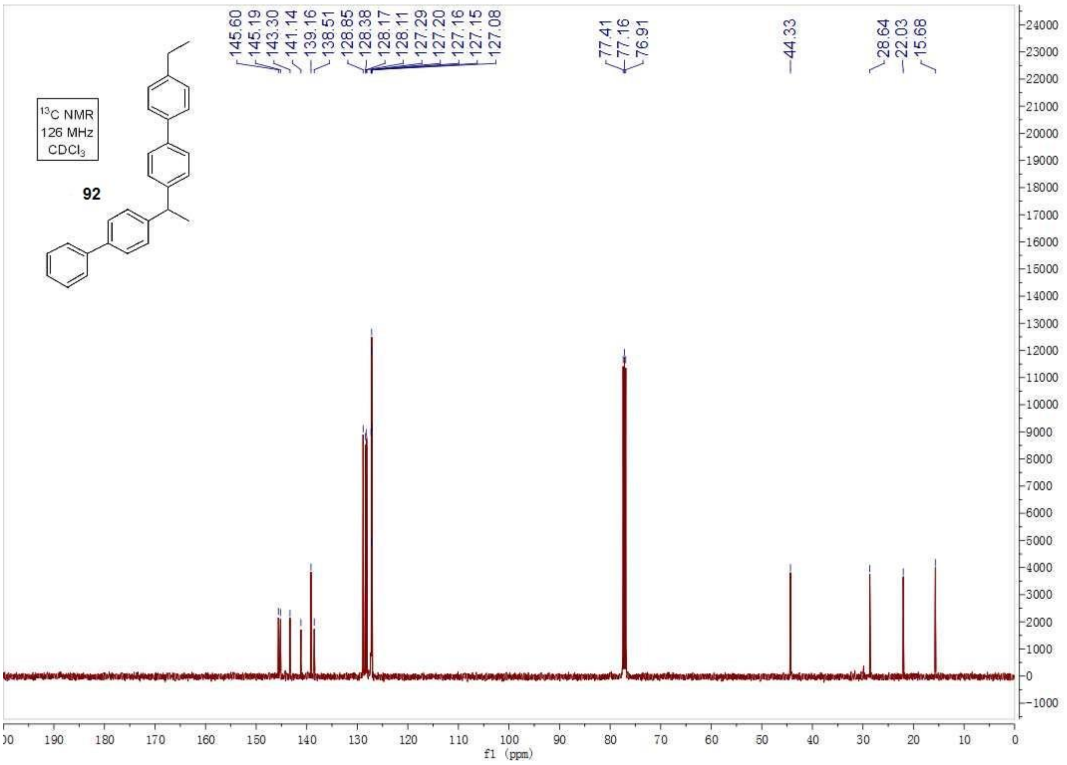

145.60 145.19 143.30 141.14 139.16 138.51 128.85 128.38 128.17 128.11 127.29 127.20 127.16 127.15 127.08
77.41 77.16 76.91
44.33
28.64 22.03 15.68
^{13}C NMR
126 MHz
CDCl$_3$
92
f1 (ppm)

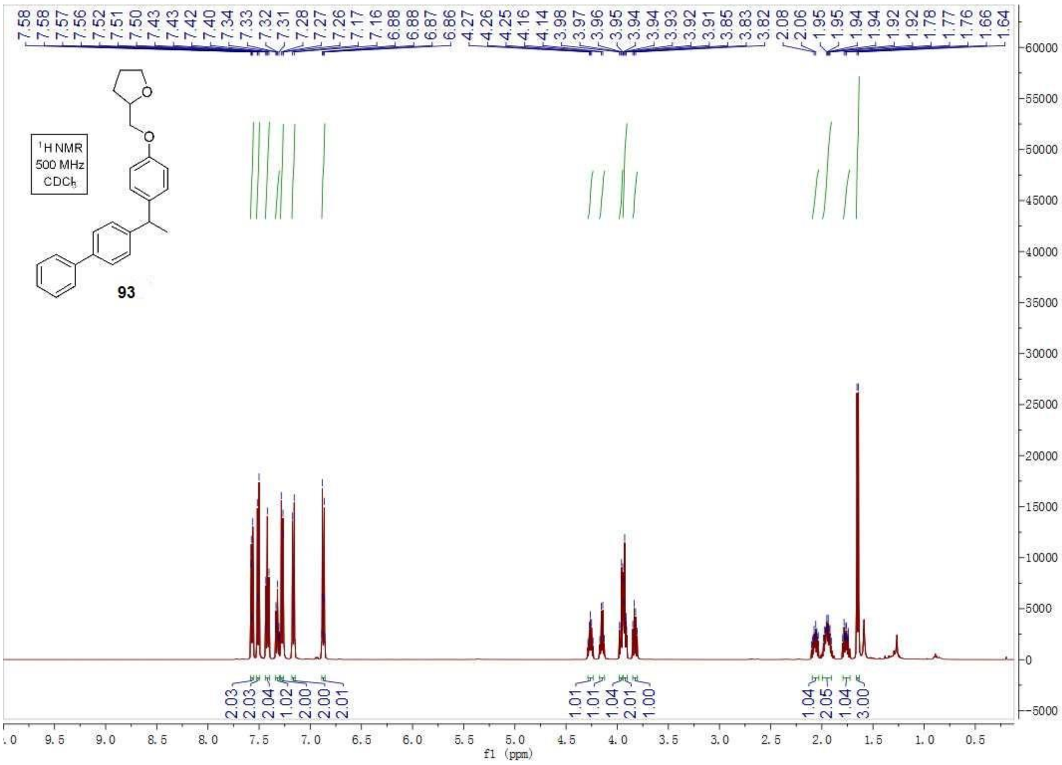

1H NMR
500 MHz
CDCl3
93
f1 (ppm)

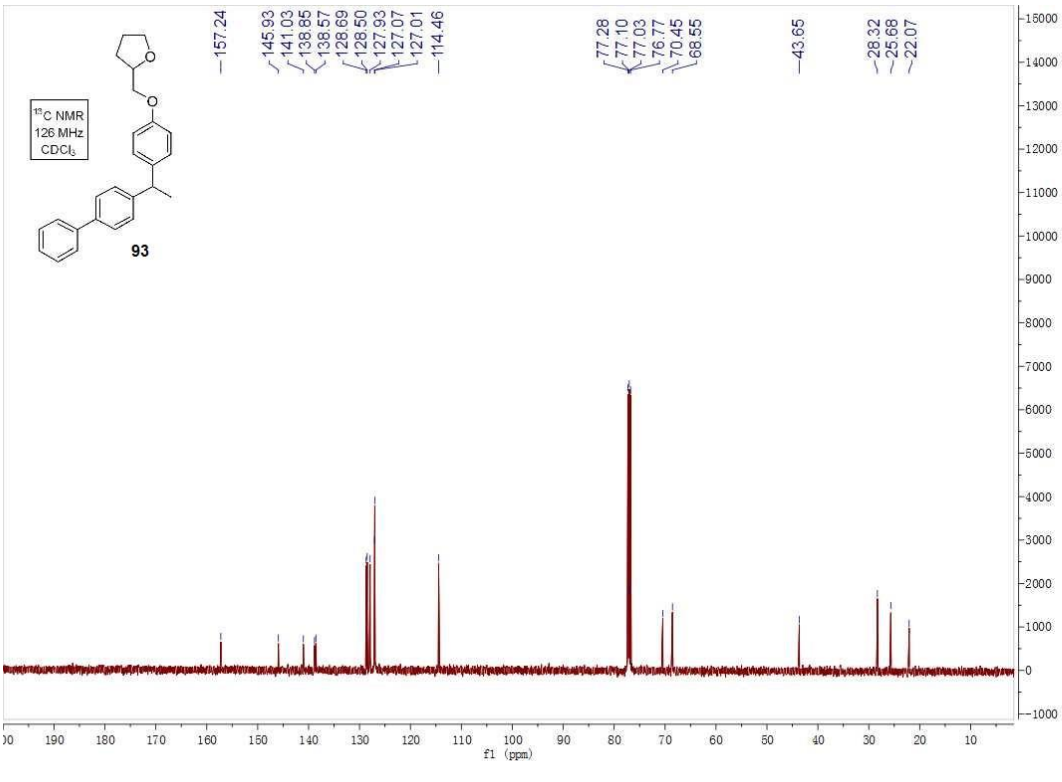

13C NMR
126 MHz
CDCl3
93
157.24
145.93
141.03
138.85
138.57
128.69
128.50
127.93
127.07
127.01
114.46
77.28
77.10
77.03
76.77
70.45
68.55
43.65
28.32
25.68
22.07
f1 (ppm)

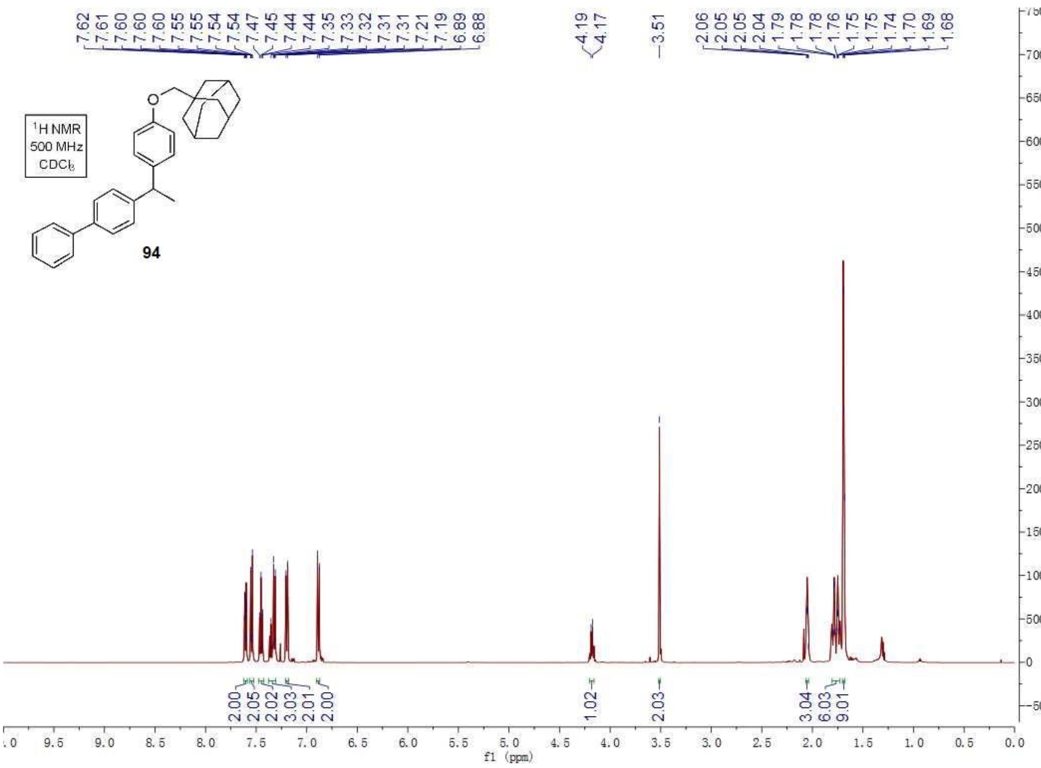
7.62
7.61
7.60
7.60
7.55
7.55
7.54
7.54
7.47
7.45
7.44
7.44
7.35
7.33
7.32
7.31
7.31
7.21
7.19
6.89
6.88
4.19
4.17
3.51
2.06
2.05
2.05
2.04
1.79
1.78
1.78
1.76
1.75
1.75
1.74
1.70
1.69
1.68
1H NMR
500 MHz
CDCl3
94
2.00
2.05
2.02
3.03
2.01
2.00
1.02
2.03
3.04
6.03
9.01
f1 (ppm)

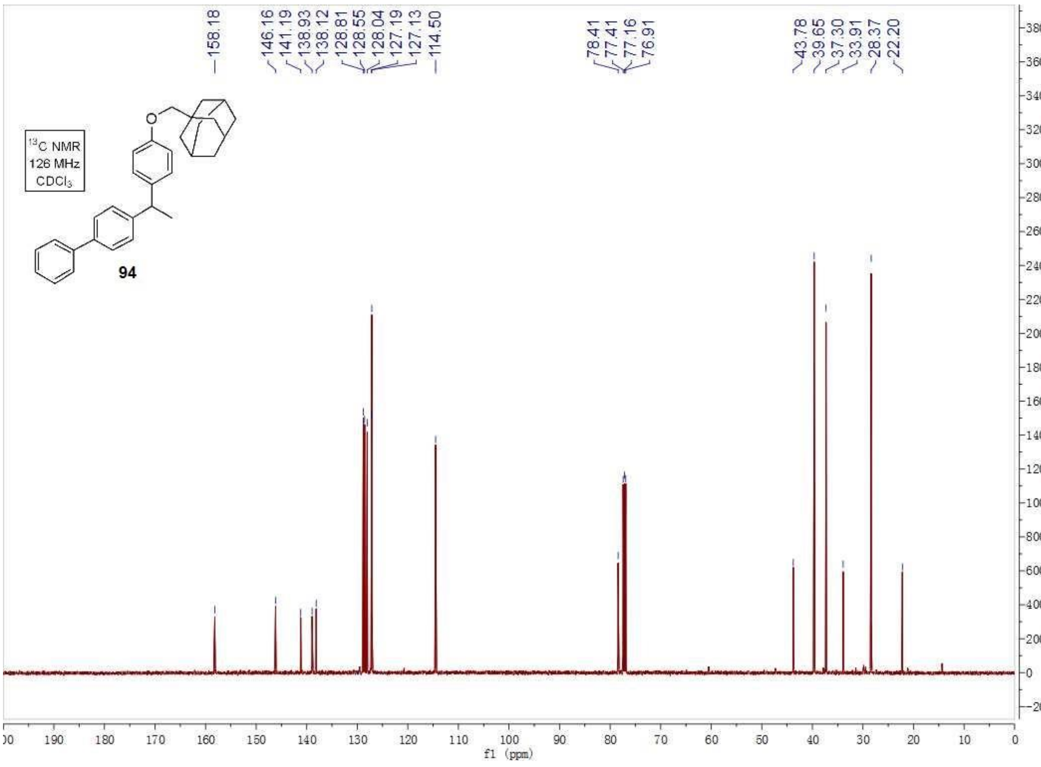
158.18
146.16
141.19
138.93
138.12
128.81
128.55
128.04
127.19
127.13
114.50
78.41
77.41
77.16
76.91
43.78
39.65
37.30
33.91
28.37
22.20
13C NMR
126 MHz
CDCl3
94
f1 (ppm)

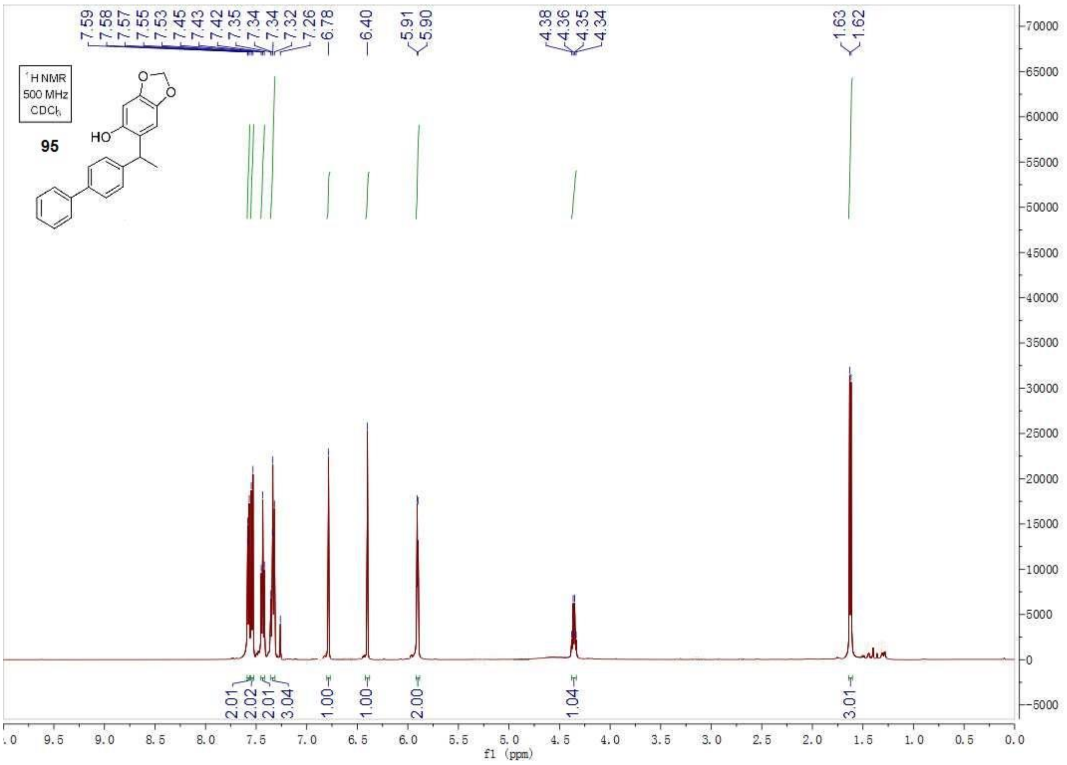

1H NMR
500 MHz
CDCl3
95
HO
f1 (ppm)

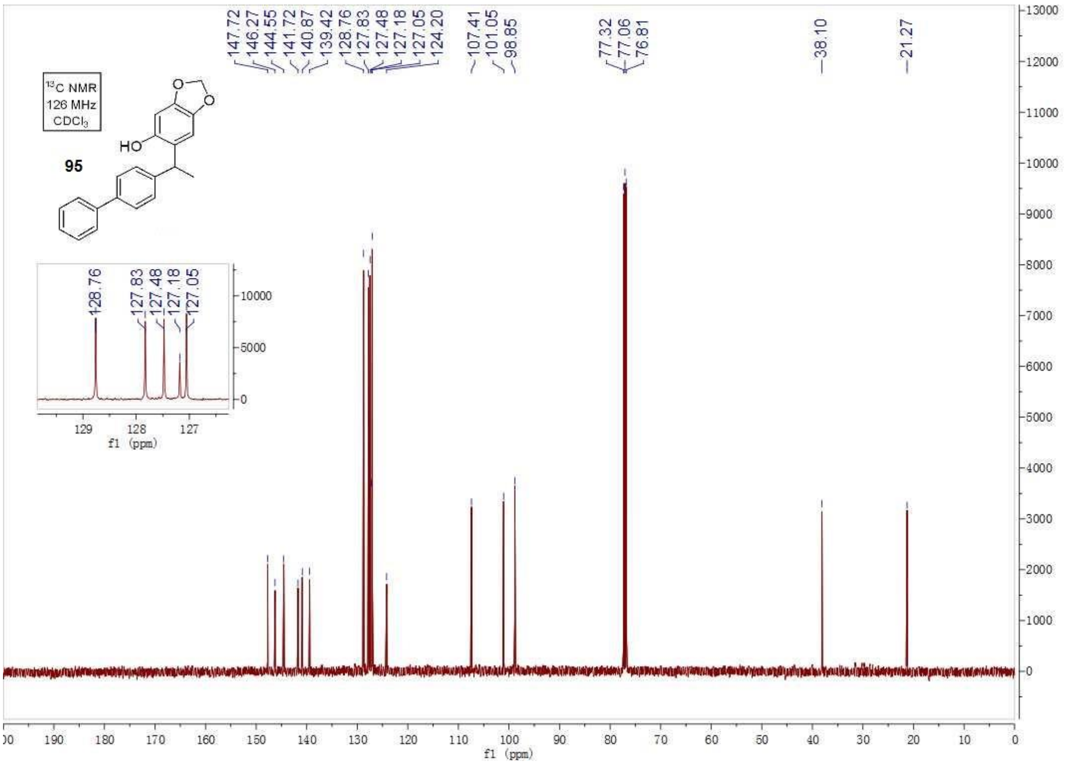

13C NMR
126 MHz
CDCl3
95
HO
f1 (ppm)

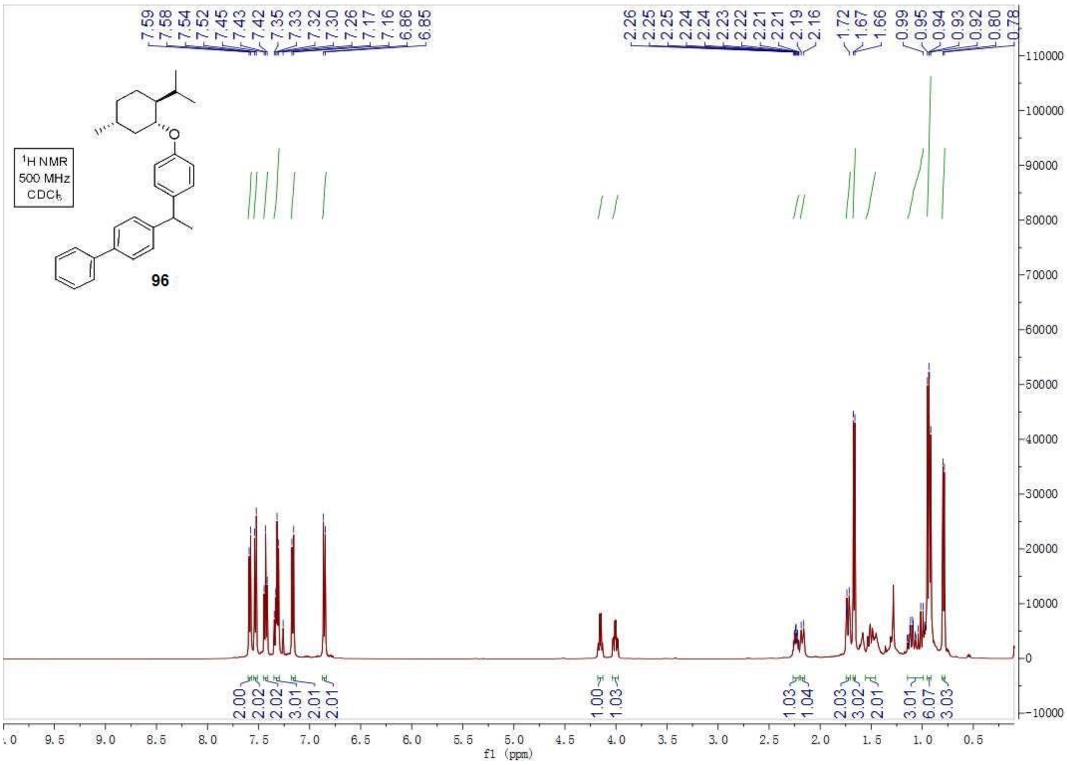

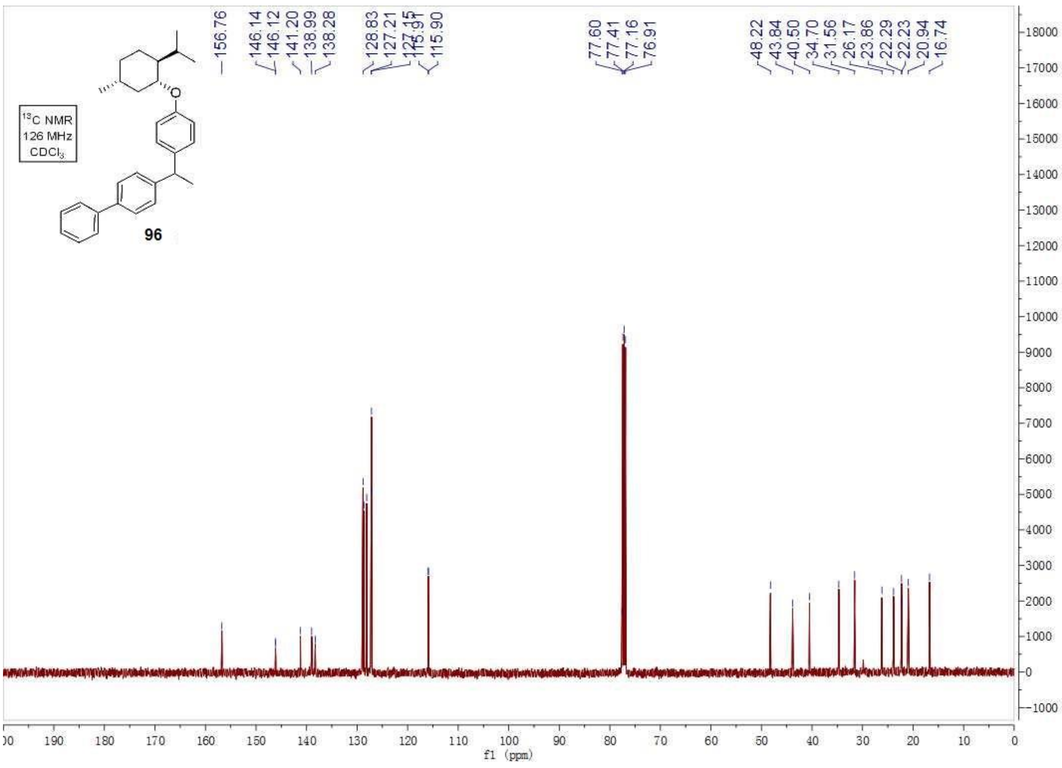

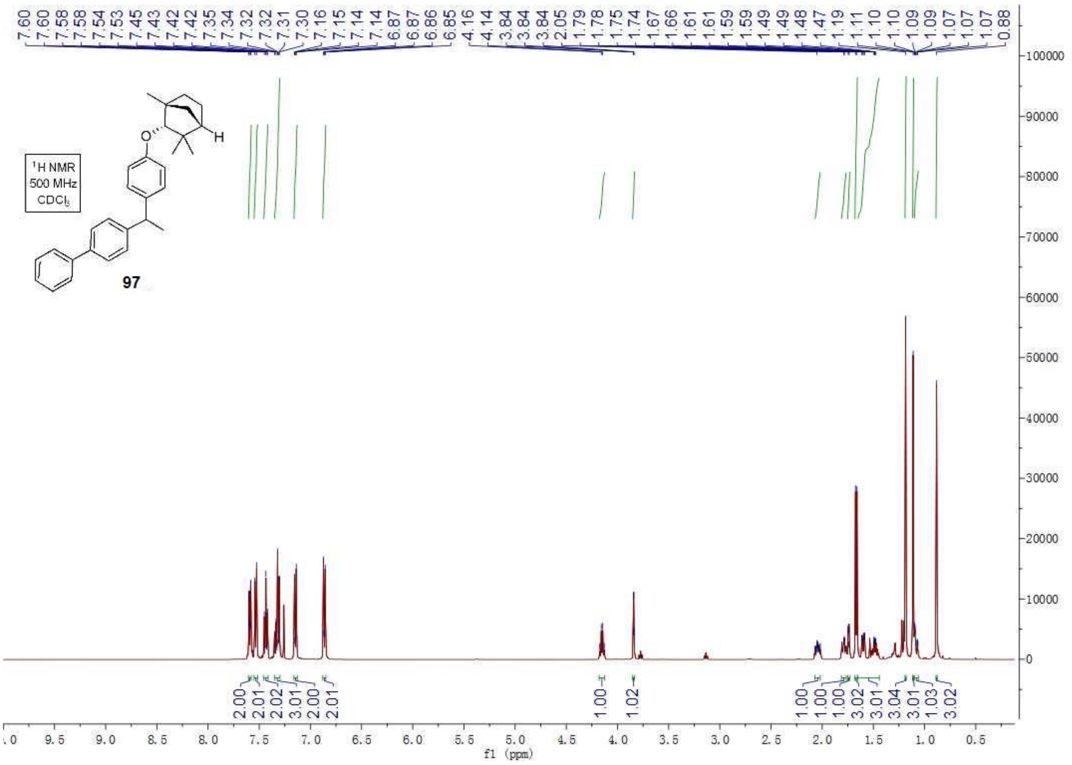

1H NMR
500 MHz
CDCl3
97
7.60 7.60 7.58 7.58 7.54 7.53 7.45 7.43 7.42 7.42 7.35 7.34 7.32 7.32 7.31 7.30 7.16 7.15 7.14 7.14 6.87 6.87 6.86 6.85 4.16 4.14 3.84 3.84 3.84 2.05 1.79 1.78 1.75 1.74 1.67 1.66 1.61 1.61 1.59 1.59 1.49 1.49 1.48 1.47 1.19 1.11 1.10 1.10 1.09 1.09 1.07 1.07 0.88
2.00 2.01 2.02 3.01 2.00 2.01 1.00 1.02 1.00 1.00 1.00 3.02 3.01 3.04 3.01 1.03 3.02
f1 (ppm)

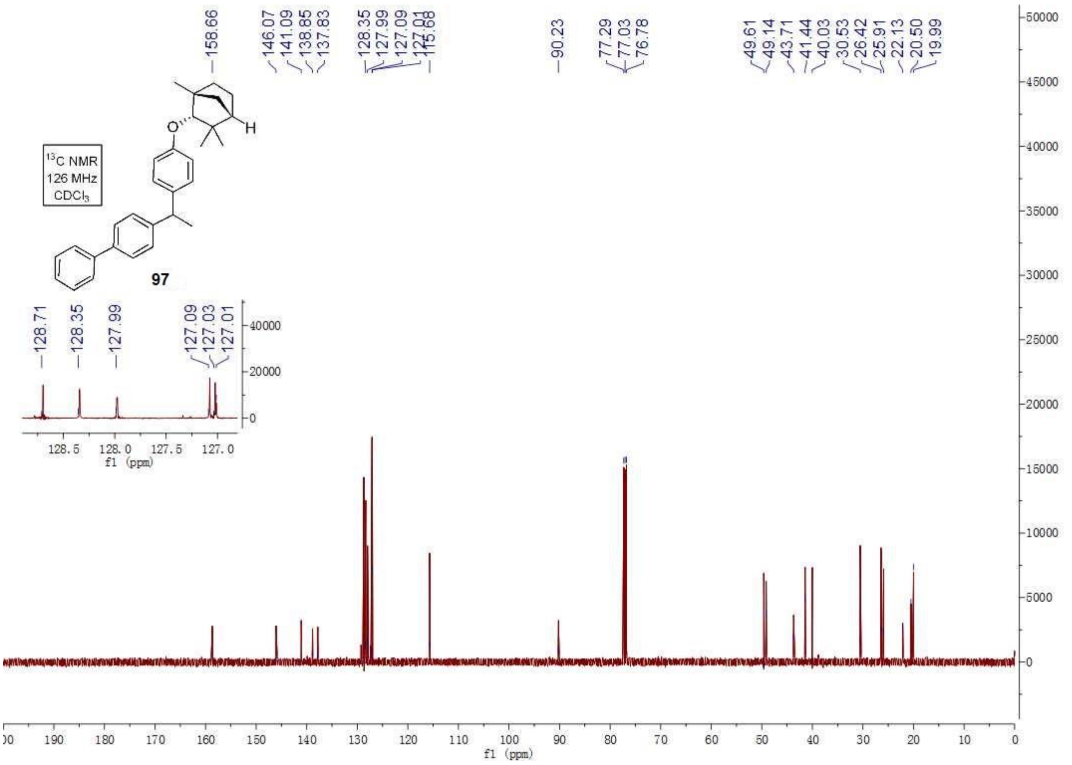

13C NMR
126 MHz
CDCl3
97
158.66 146.07 141.09 138.85 137.83 128.35 127.99 127.09 127.01 115.68 90.23 77.29 77.03 76.78 49.61 49.14 43.71 41.44 40.03 30.53 26.42 25.91 22.13 20.50 19.99
128.71 128.35 127.99 127.09 127.03 127.01
f1 (ppm)

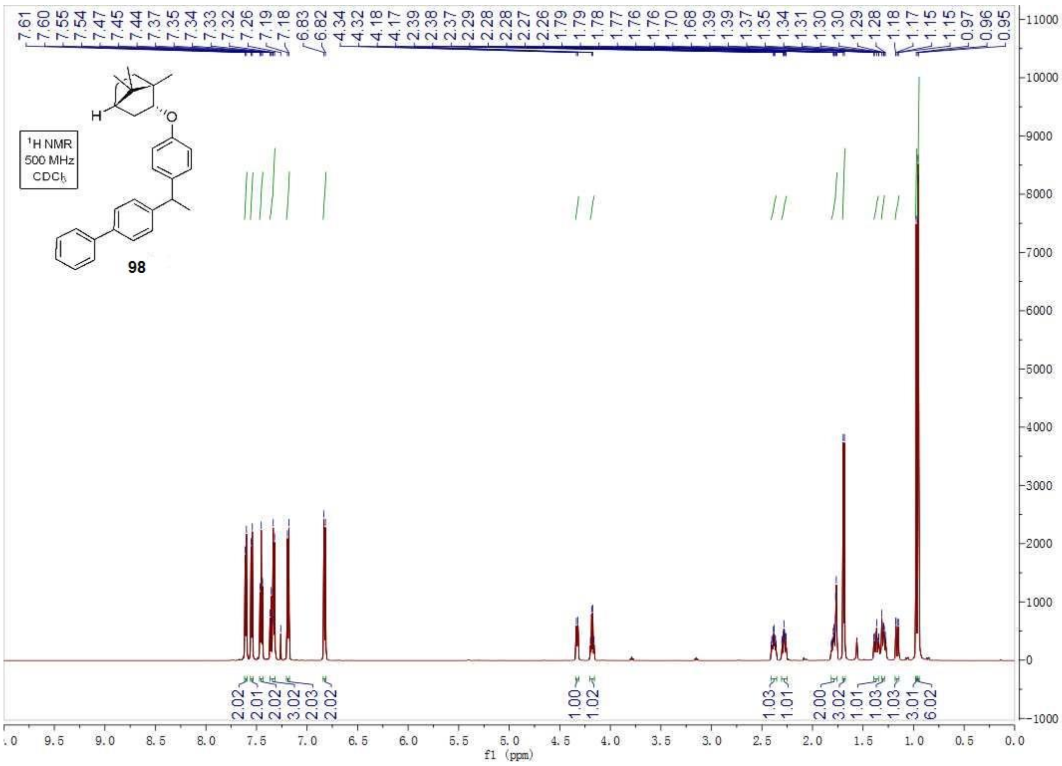

7.61 7.60 7.55 7.54 7.47 7.45 7.44 7.37 7.35 7.34 7.33 7.32 7.26 7.19 7.18 6.83 6.82 4.34 4.32 4.18 4.17 2.39 2.38 2.37 2.29 2.28 2.28 2.27 2.26 1.79 1.79 1.78 1.77 1.76 1.76 1.70 1.68 1.39 1.39 1.37 1.35 1.34 1.31 1.30 1.30 1.29 1.28 1.18 1.17 1.15 0.97 0.96 0.95
H
O
98
^{1}H NMR
500 MHz
CDCl3
2.02 2.01 2.02 3.02 2.03 2.02 1.00 1.02 1.03 1.01 2.00 3.02 1.01 1.03 1.03 3.01 6.02
f1 (ppm)

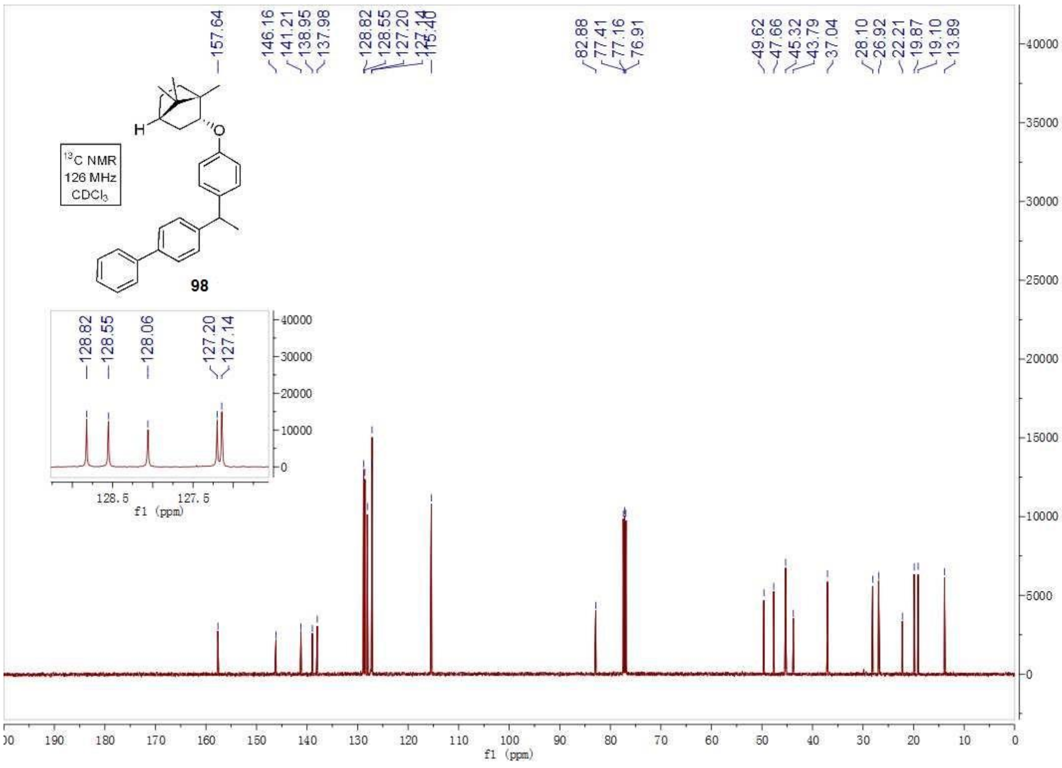

157.64 146.16 141.21 138.95 137.98 128.82 128.55 127.20 127.14 115.40 82.88 77.41 77.16 76.91 49.62 47.66 45.32 43.79 37.04 28.10 26.92 22.21 19.87 19.10 13.89
H
O
98
^{13}C NMR
126 MHz
CDCl3
128.82 128.55 128.06 127.20 127.14
f1 (ppm)

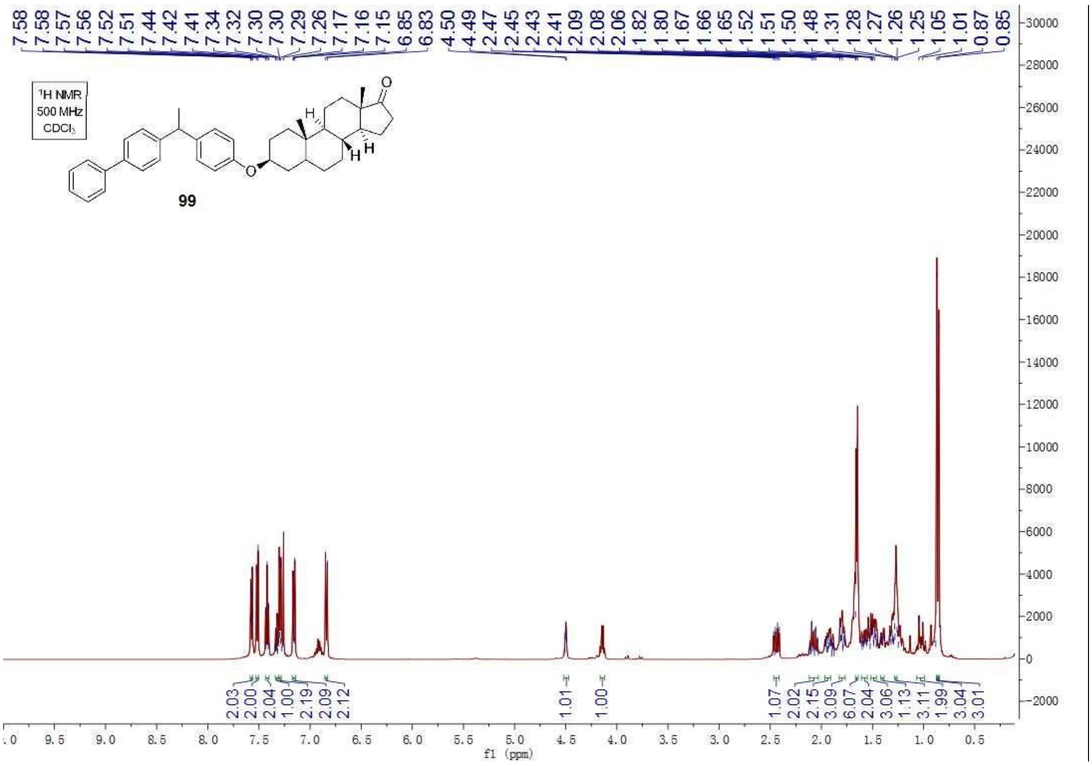

1H NMR
500 MHz
CDCl3
99

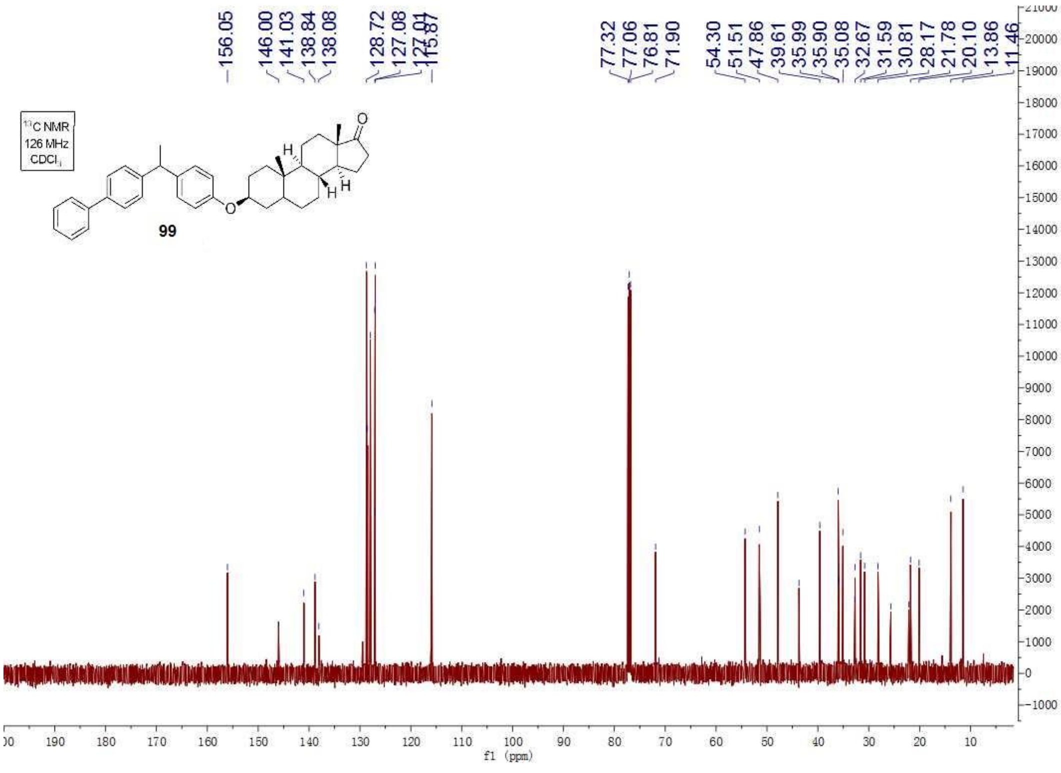

13C NMR
126 MHz
CDCl3
99

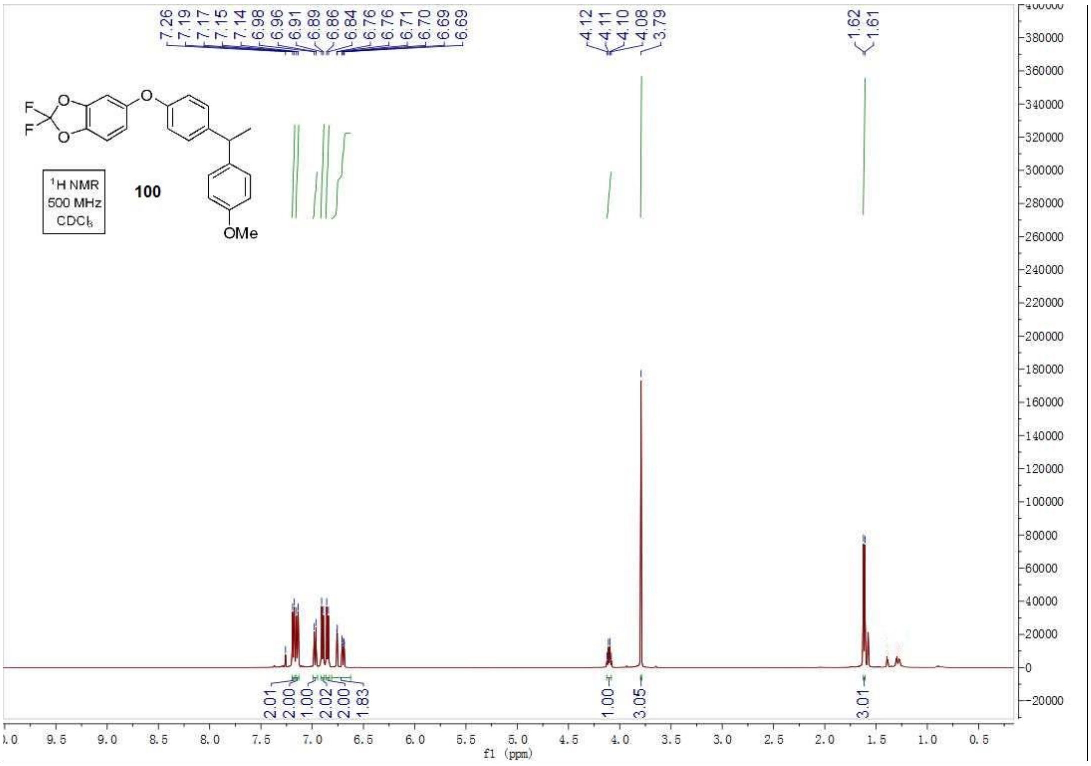

7.26 7.19 7.17 7.15 7.14 6.98 6.96 6.91 6.89 6.86 6.84 6.76 6.71 6.70 6.69 6.69
4.12 4.11 4.10 4.08 3.79
1.62 1.61
F
F
O
O
O
OMe
^{1}H NMR
500 MHz
$CDCl_3$
100
2.01 2.00 1.00 2.02 2.00 1.83
1.00
3.05
3.01
f1 (ppm)

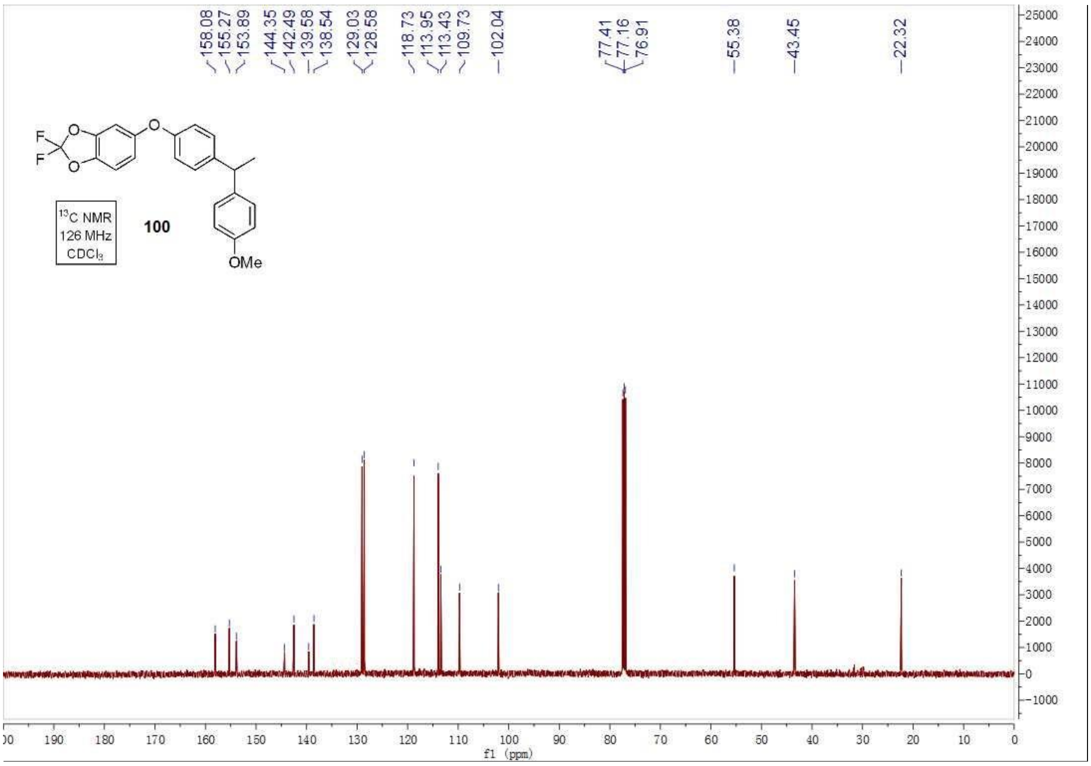

158.08 155.27 153.89 144.35 142.49 139.58 138.54 129.03 128.58 118.73 113.95 113.43 109.73 102.04
77.41 77.16 76.91
55.38
43.45
22.32
F
F
O
O
O
OMe
^{13}C NMR
126 MHz
$CDCl_3$
100
f1 (ppm)

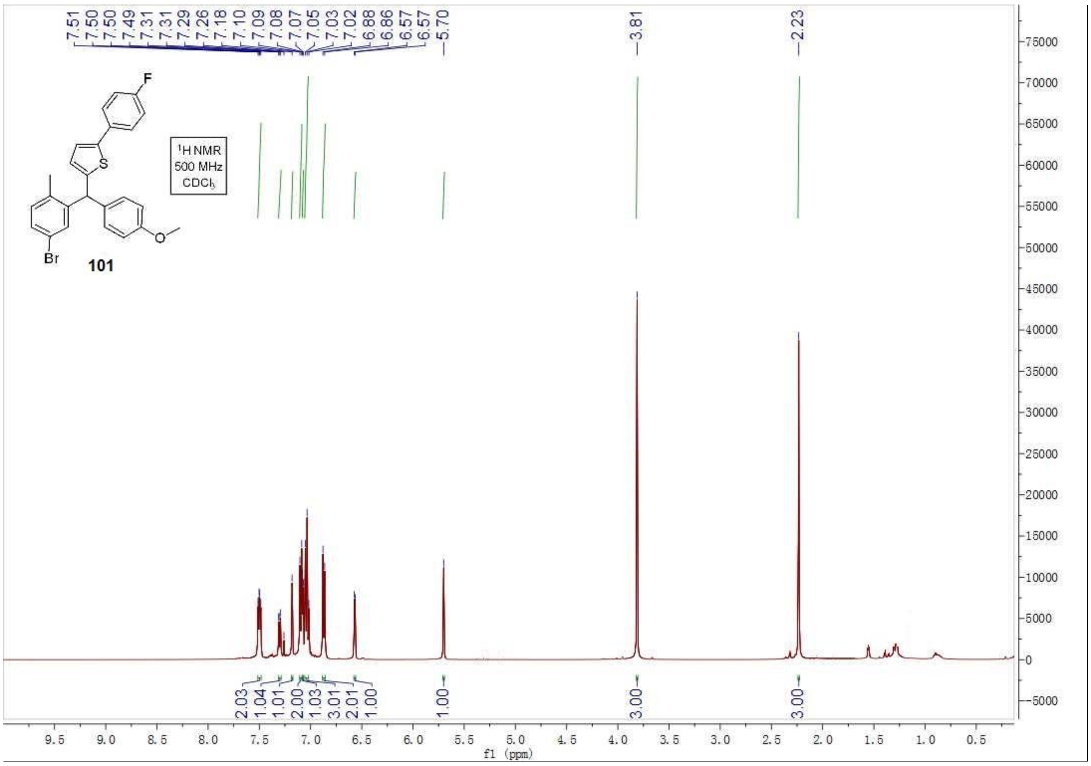
7.51
7.50
7.50
7.49
7.31
7.31
7.29
7.26
7.18
7.10
7.09
7.08
7.07
7.05
7.03
7.02
6.88
6.86
6.57
6.57
5.70
3.81
2.23
F
S
Br
O
101
1H NMR
500 MHz
CDCl3
2.03
1.04
1.01
2.00
1.03
3.01
2.01
1.00
1.00
3.00
3.00
f1 (ppm)

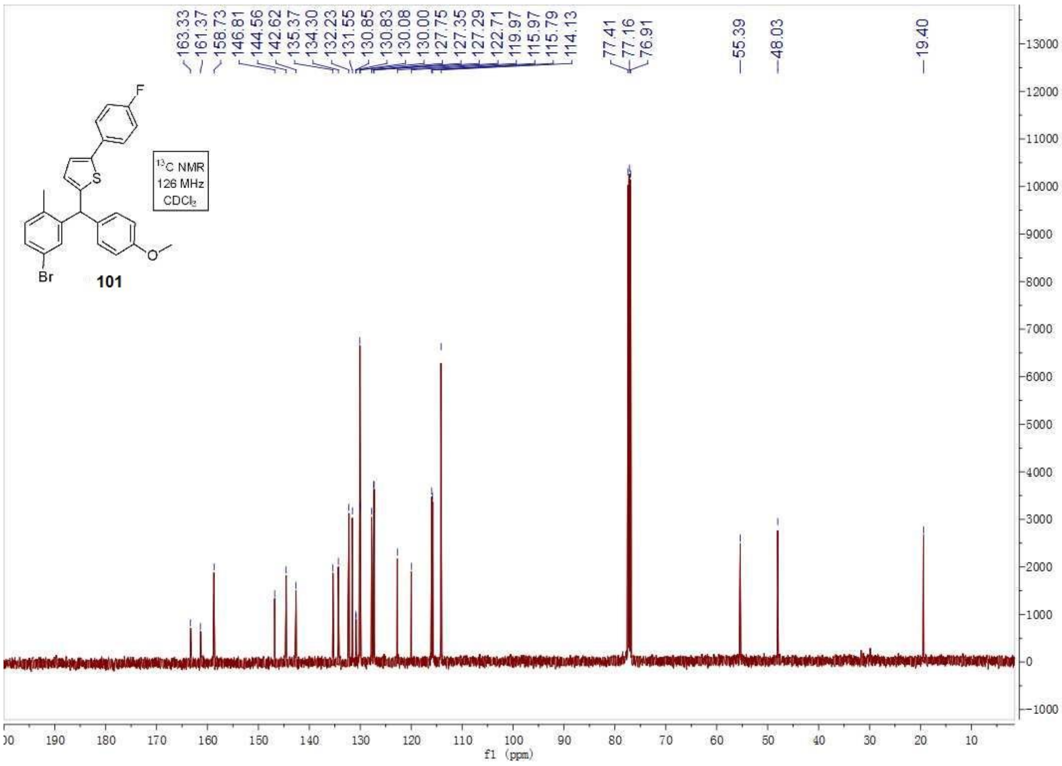
163.33
161.37
158.73
146.81
144.56
142.62
135.37
134.30
132.23
131.55
130.85
130.83
130.08
130.00
127.75
127.35
127.29
122.71
119.97
115.97
115.79
114.13
77.41
77.16
76.91
55.39
48.03
19.40
F
S
Br
O
101
13C NMR
126 MHz
CDCl3
f1 (ppm)

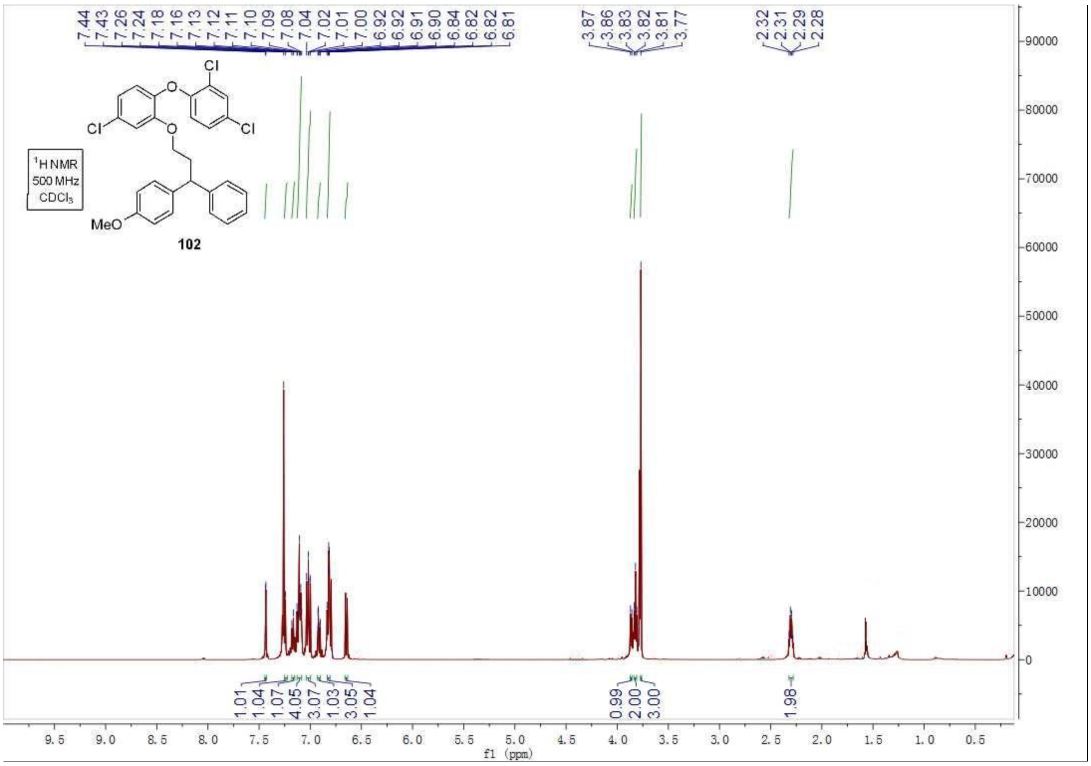

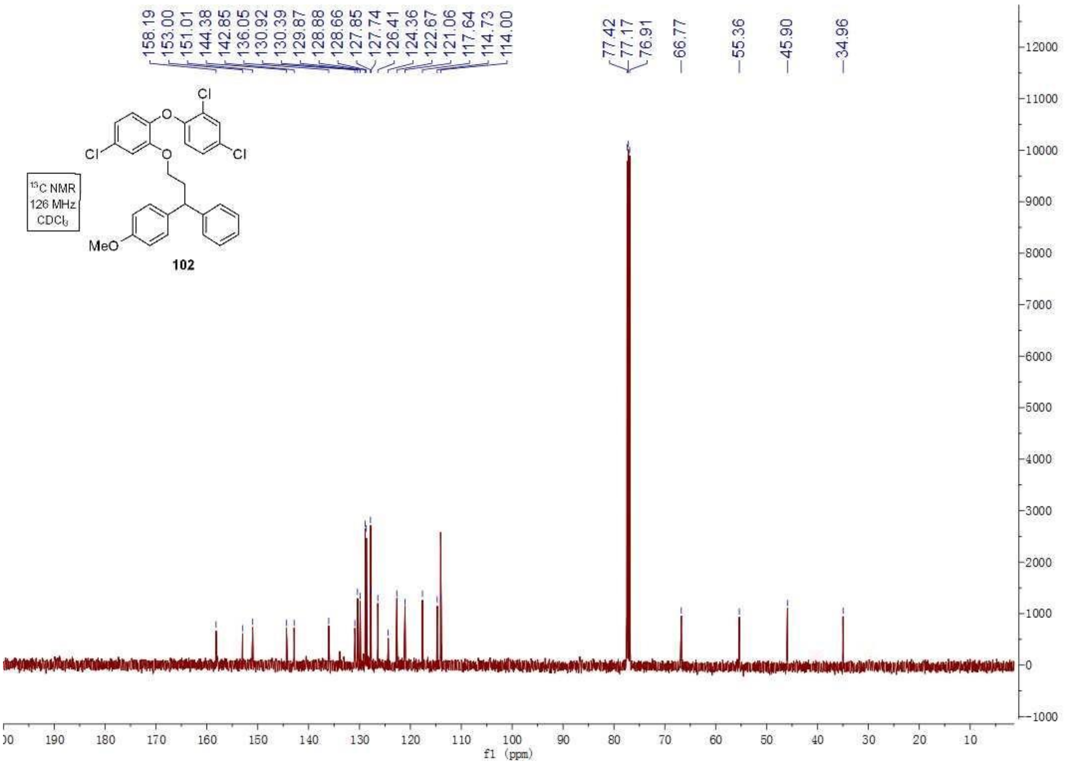

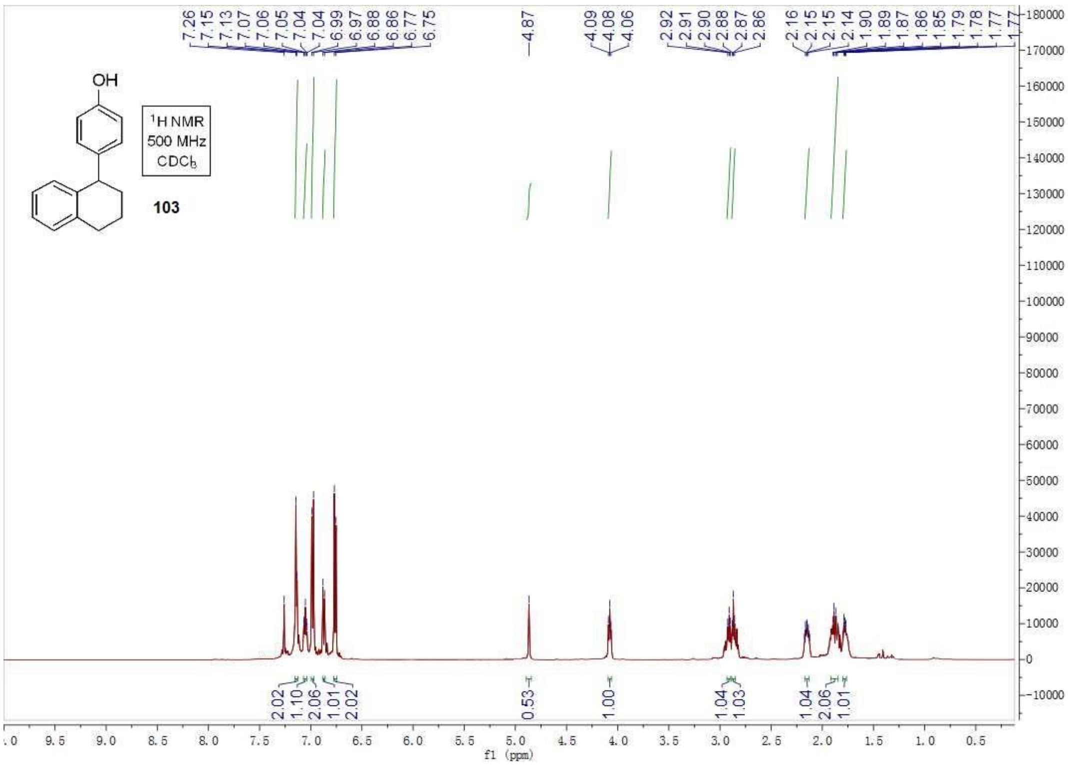
OH
1H NMR
500 MHz
CDCl3
103
7.26 7.15 7.13 7.07 7.06 7.05 7.04 7.04 6.99 6.97 6.88 6.86 6.77 6.75
4.87
4.09 4.08 4.06
2.92 2.91 2.90 2.88 2.87 2.86
2.16 2.15 2.15 2.14 1.90 1.89 1.87 1.86 1.85 1.79 1.78 1.77 1.77
2.02 1.10 2.06 1.01 2.02
0.53
1.00
1.04 1.03
1.04 2.06 1.01
f1 (ppm)

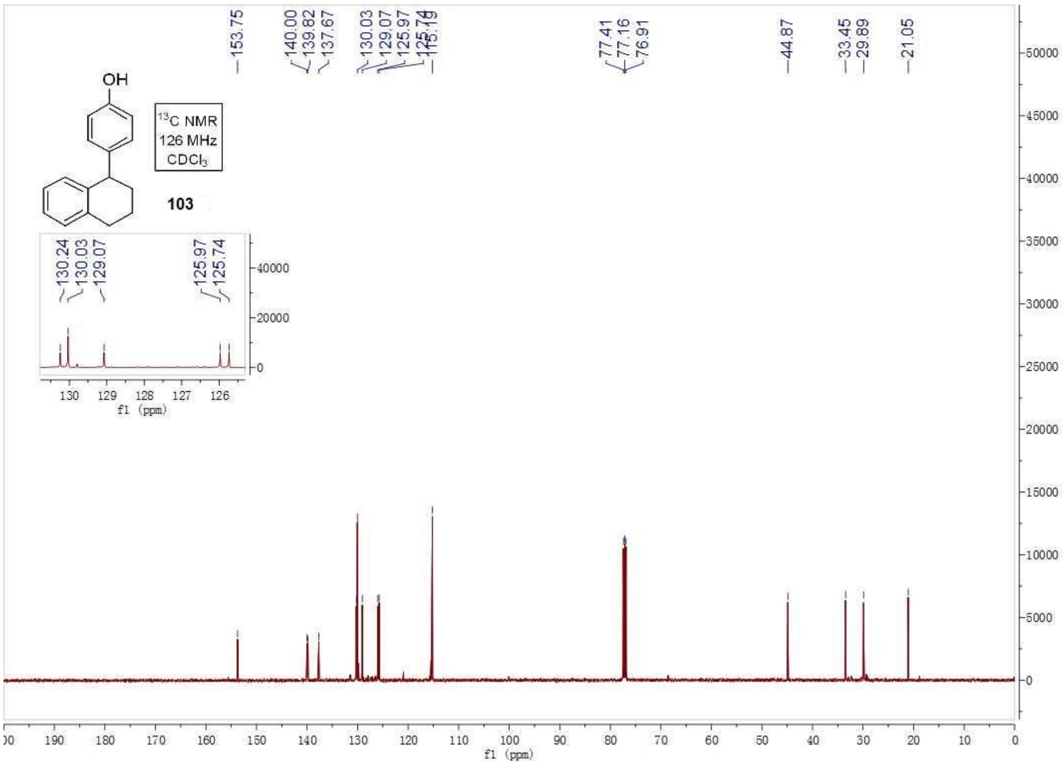
OH
13C NMR
126 MHz
CDCl3
103
153.75
140.00 139.82 137.67
130.03 129.07 125.97 125.74 115.19
77.41 77.16 76.91
44.87
33.45 29.89
21.05
130.24 130.03 129.07
125.97 125.74
f1 (ppm)

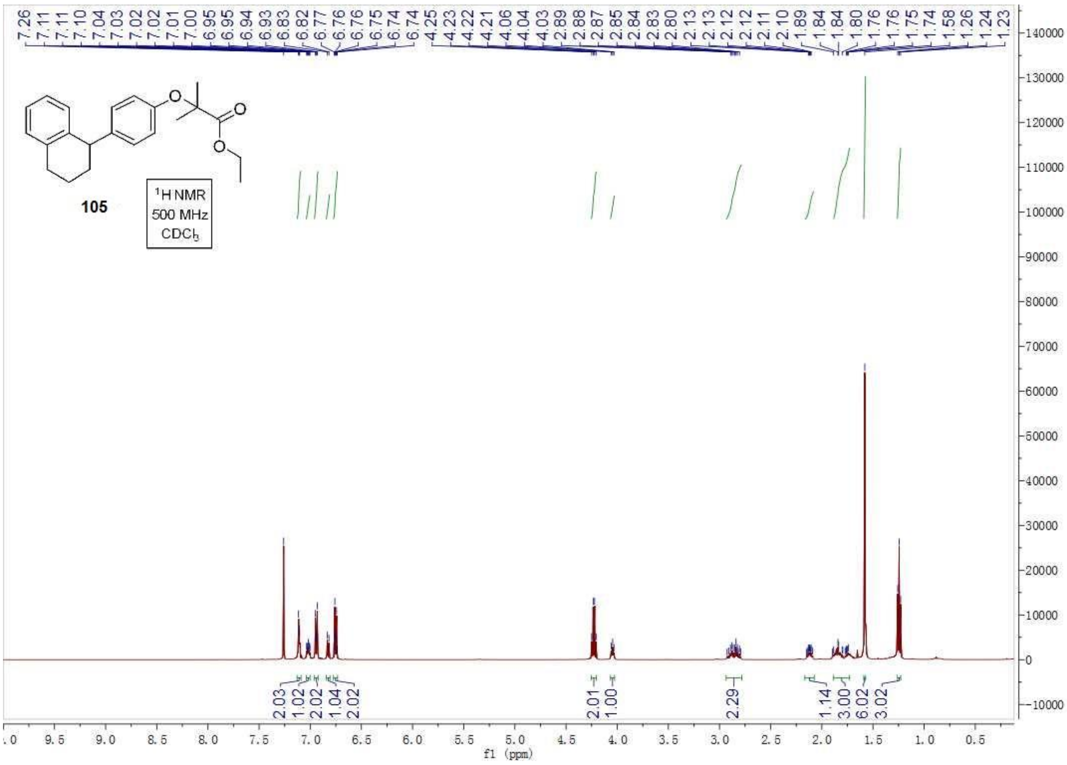
105
^{1}H NMR
500 MHz
CDCl$_3$

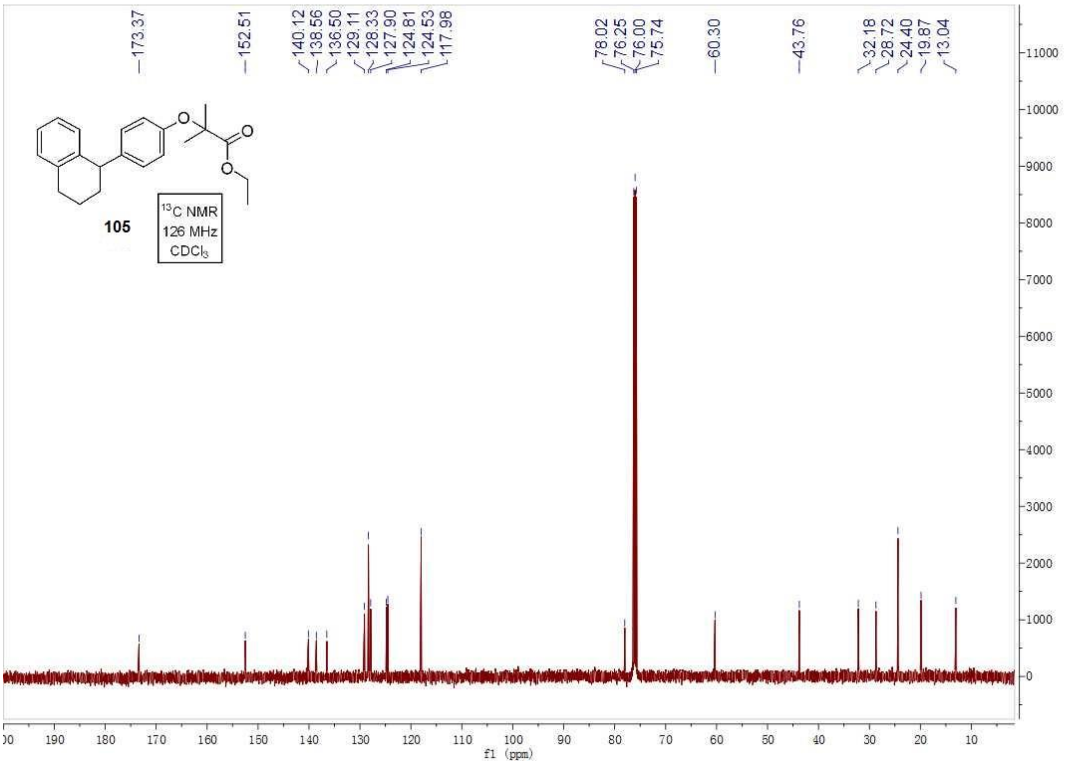
105
^{13}C NMR
126 MHz
CDCl$_3$

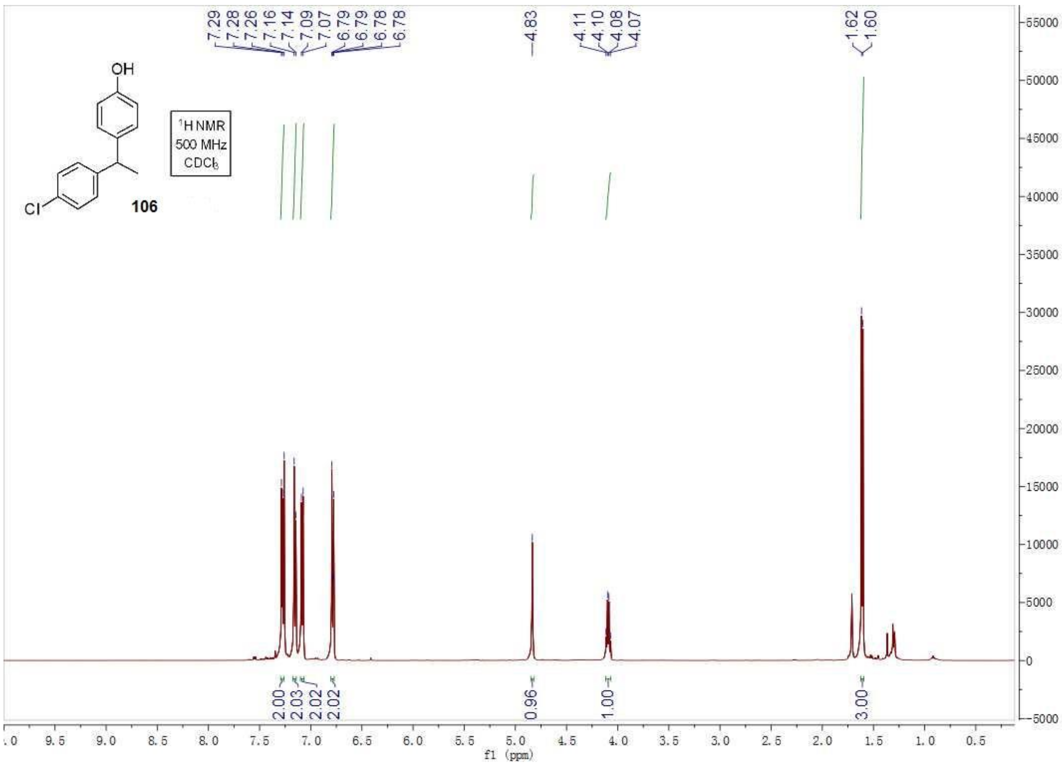

7.29
7.28
7.26
7.16
7.14
7.09
7.07
6.79
6.79
6.78
6.78
4.83
4.11
4.10
4.08
4.07
1.62
1.60
OH
Cl
106
1H NMR
500 MHz
CDCl3
2.00
2.03
2.02
2.02
0.96
1.00
3.00
f1 (ppm)

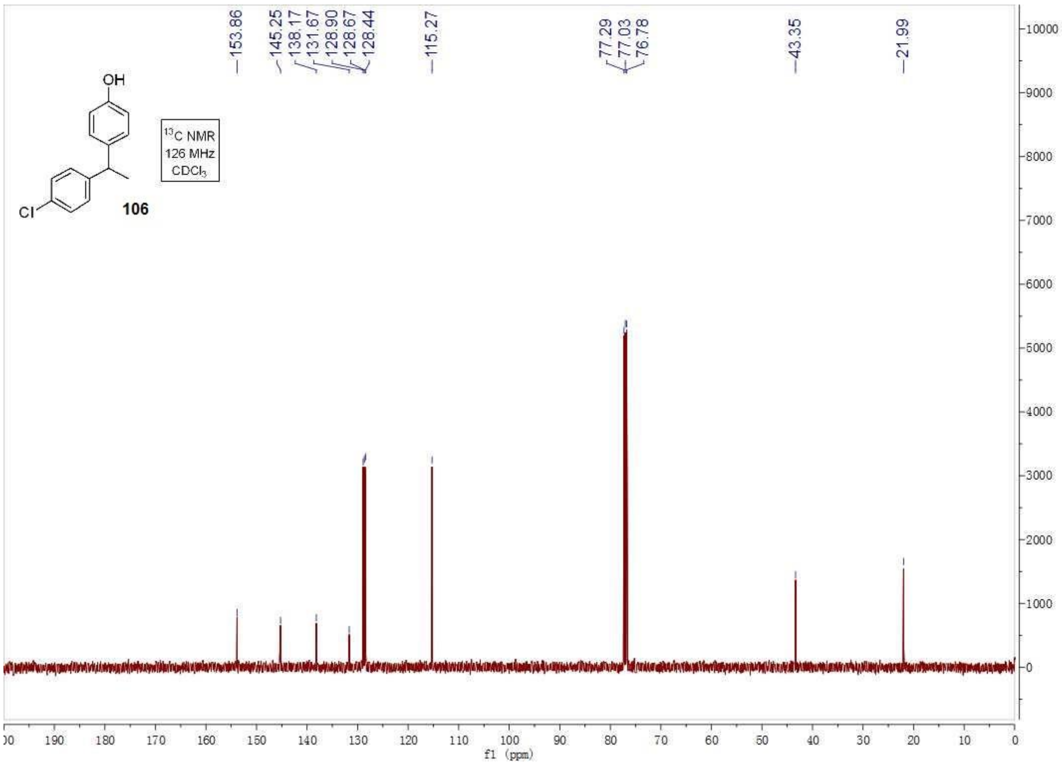

153.86
145.25
138.17
131.67
128.90
128.67
128.44
115.27
77.29
77.03
76.78
43.35
21.99
OH
Cl
106
13C NMR
126 MHz
CDCl3
f1 (ppm)

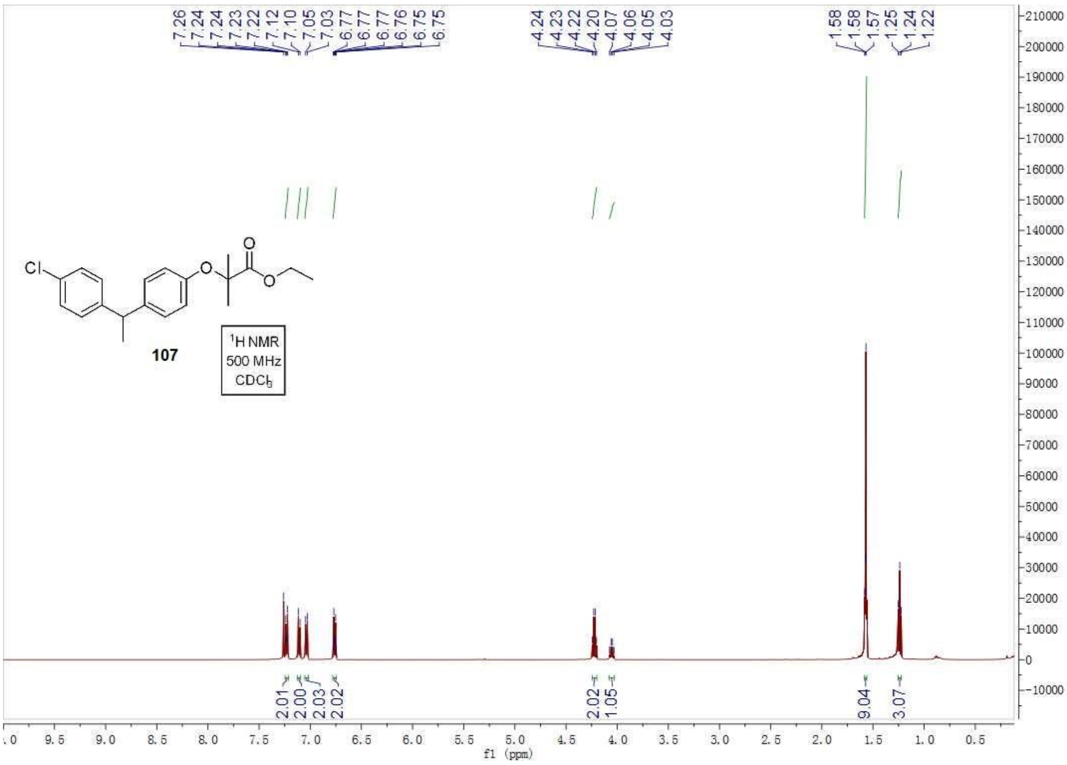
7.26
7.24
7.24
7.23
7.22
7.12
7.10
7.05
7.03
6.77
6.77
6.77
6.76
6.75
6.75
4.24
4.23
4.22
4.20
4.07
4.06
4.05
4.03
1.58
1.58
1.57
1.25
1.24
1.22
Cl
O
O
O
107
1H NMR
500 MHz
CDCl3
2.01
2.00
2.03
2.02
2.02
1.05
9.04
3.07
f1 (ppm)

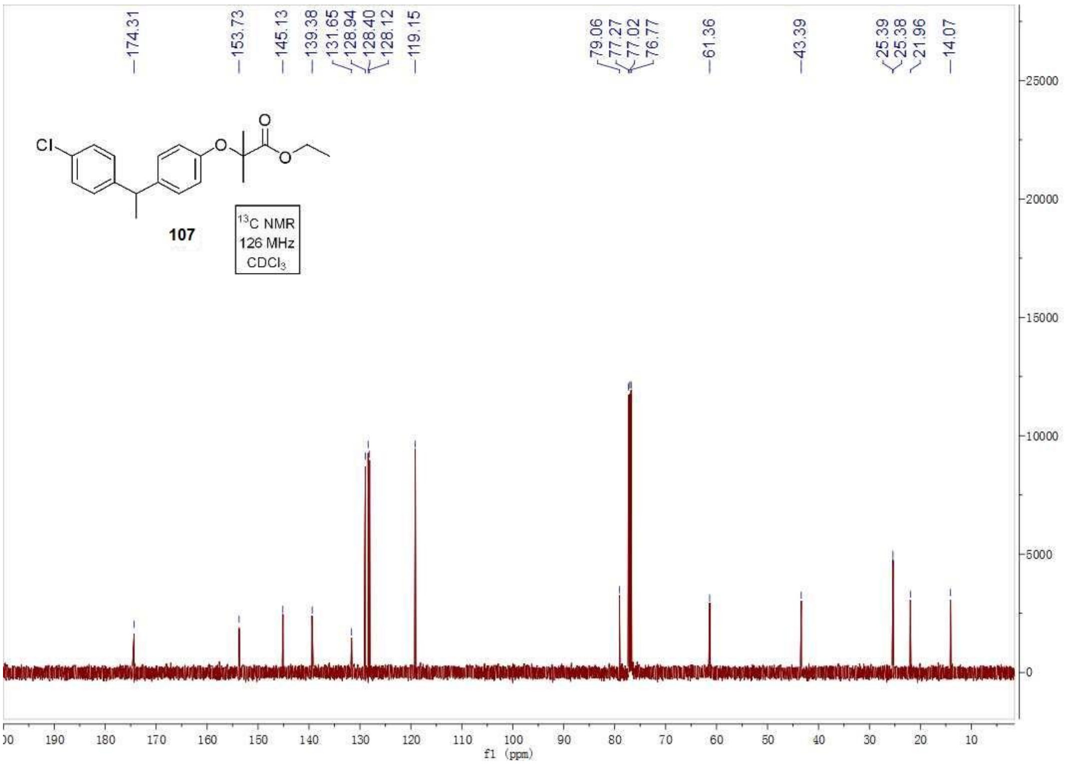
174.31
153.73
145.13
139.38
131.65
128.94
128.40
128.12
119.15
79.06
77.27
77.02
76.77
61.36
43.39
25.39
25.38
21.96
14.07
Cl
O
O
O
107
13C NMR
126 MHz
CDCl3
f1 (ppm)

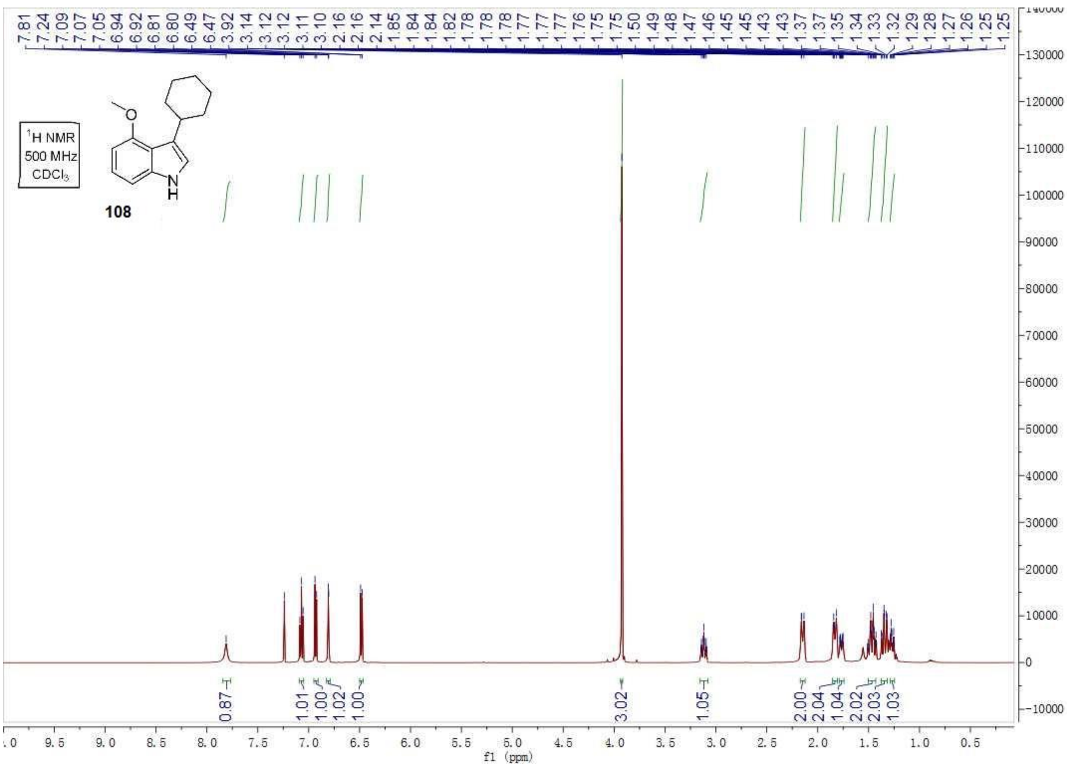
7.81 7.24 7.09 7.07 7.05 6.94 6.92 6.81 6.80 6.49 6.47 3.92 3.14 3.12 3.12 3.11 3.10 2.16 2.16 2.14 1.85 1.84 1.84 1.82 1.78 1.78 1.78 1.77 1.77 1.77 1.76 1.75 1.75 1.50 1.49 1.48 1.47 1.46 1.45 1.45 1.43 1.43 1.37 1.37 1.35 1.34 1.33 1.32 1.29 1.28 1.27 1.26 1.25 1.25
1H NMR
500 MHz
CDCl3
108
0.87 1.01 1.00 1.02 1.00 3.02 1.05 2.00 2.04 1.04 2.02 2.03 1.03
f1 (ppm)

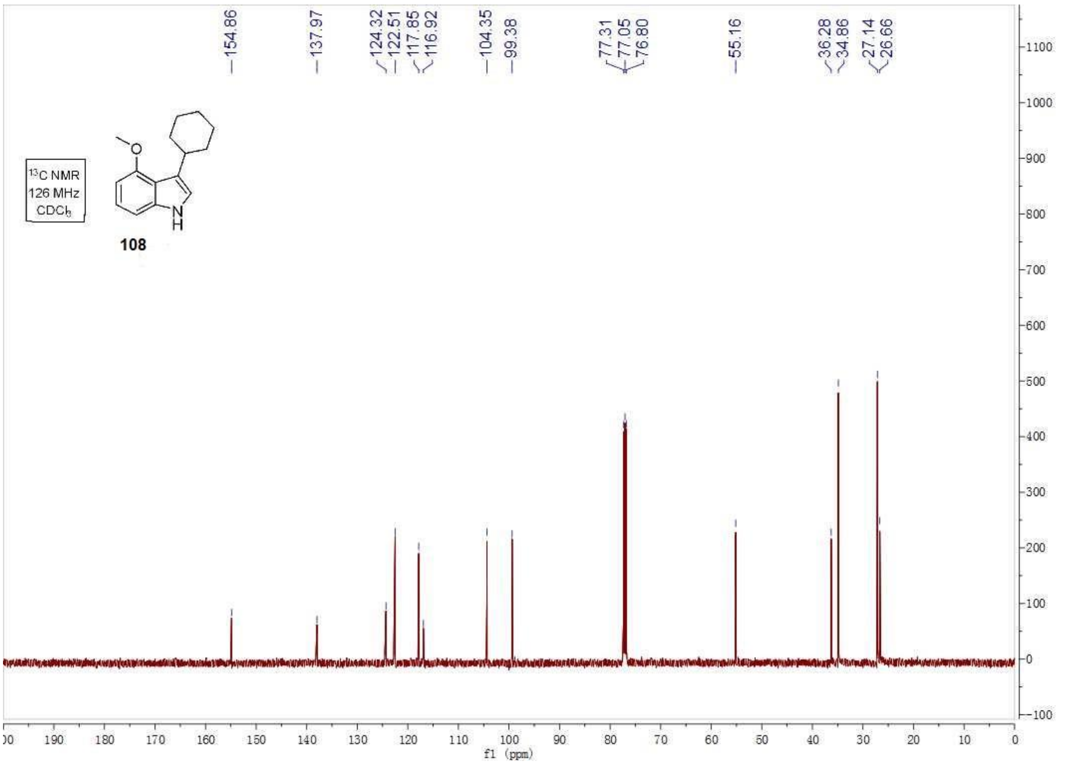
154.86 137.97 124.32 122.51 117.85 116.92 104.35 99.38 77.31 77.05 76.80 55.16 36.28 34.86 27.14 26.66
13C NMR
126 MHz
CDCl3
108
f1 (ppm)

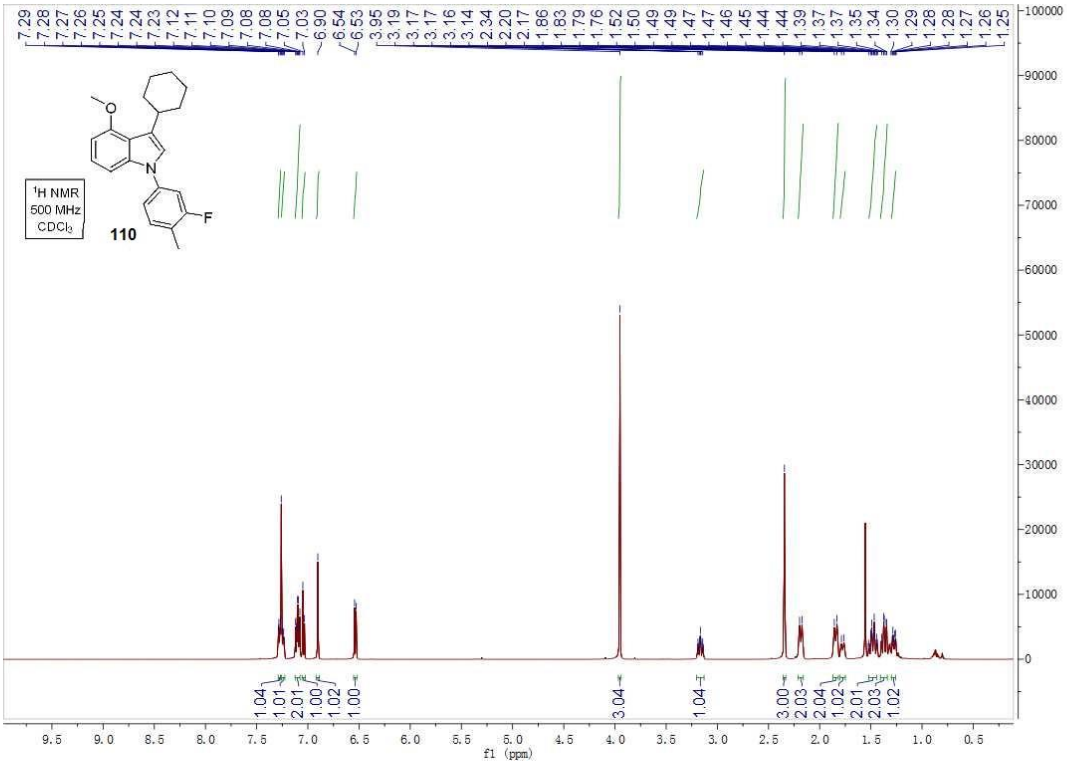
1H NMR
500 MHz
CDCl3
110
f1 (ppm)

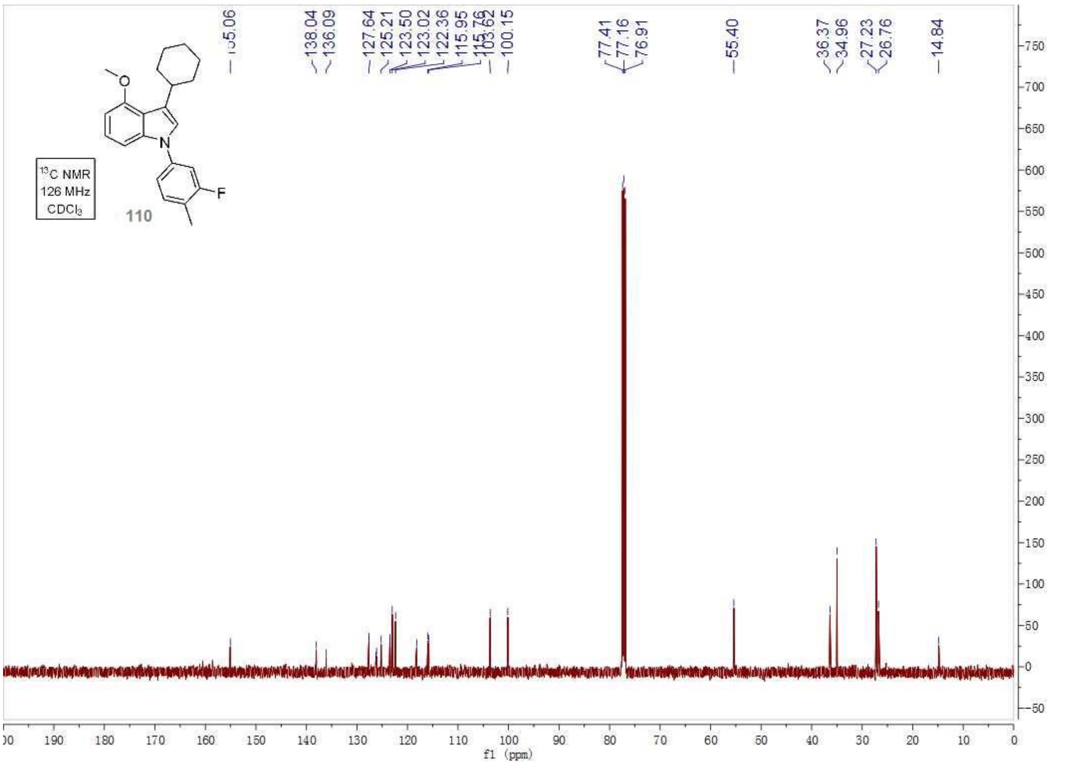
13C NMR
126 MHz
CDCl3
110
f1 (ppm)

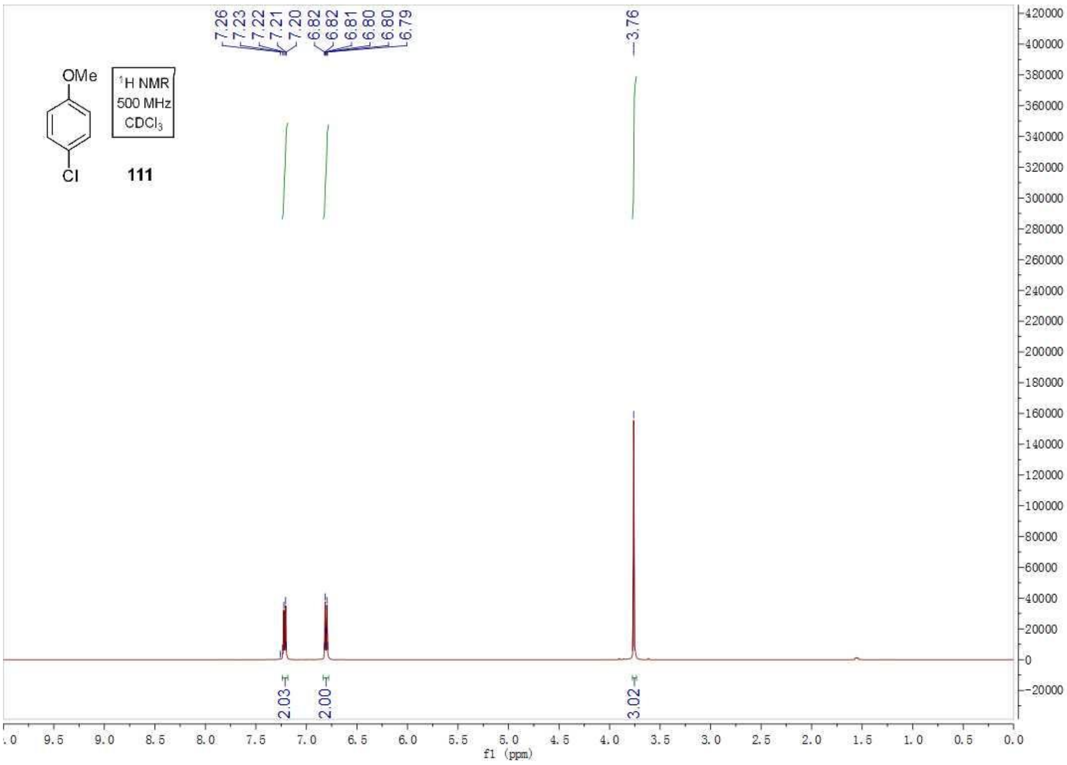
7.26
7.23
7.22
7.21
7.20
6.82
6.82
6.81
6.80
6.80
6.79
3.76
OMe
Cl
¹H NMR
500 MHz
CDCl3
111
2.03
2.00
3.02
f1 (ppm)

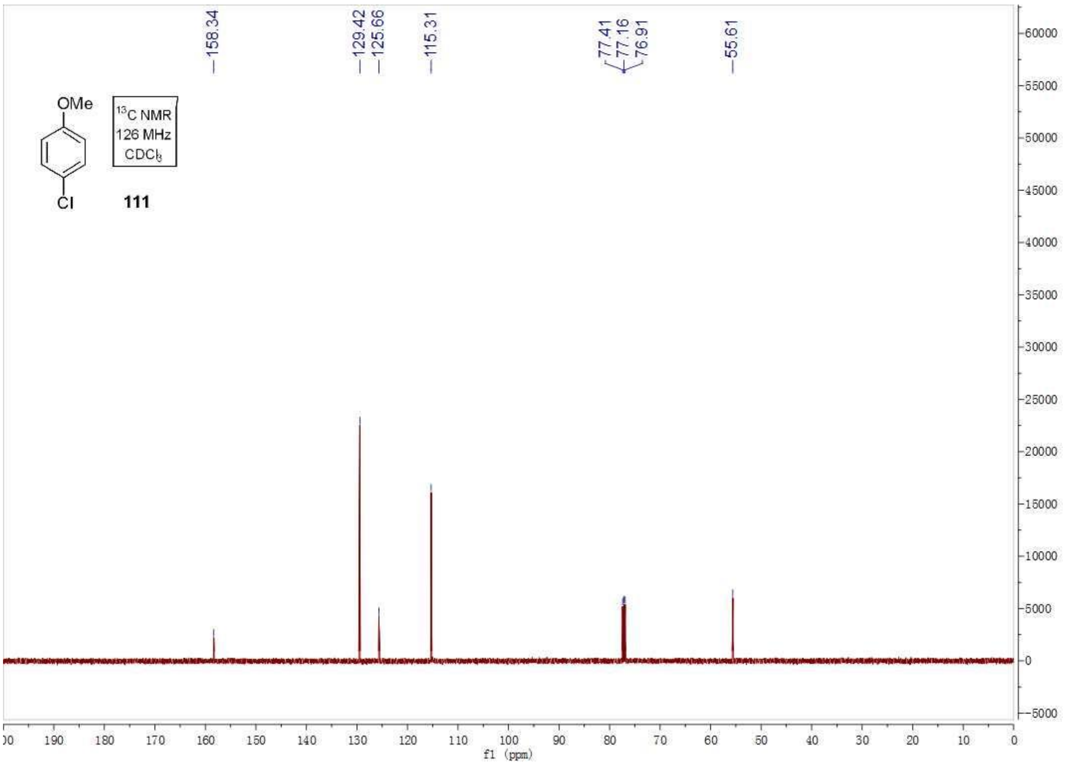
158.34
129.42
125.66
115.31
77.41
77.16
76.91
55.61
OMe
Cl
¹³C NMR
126 MHz
CDCl3
111
f1 (ppm)

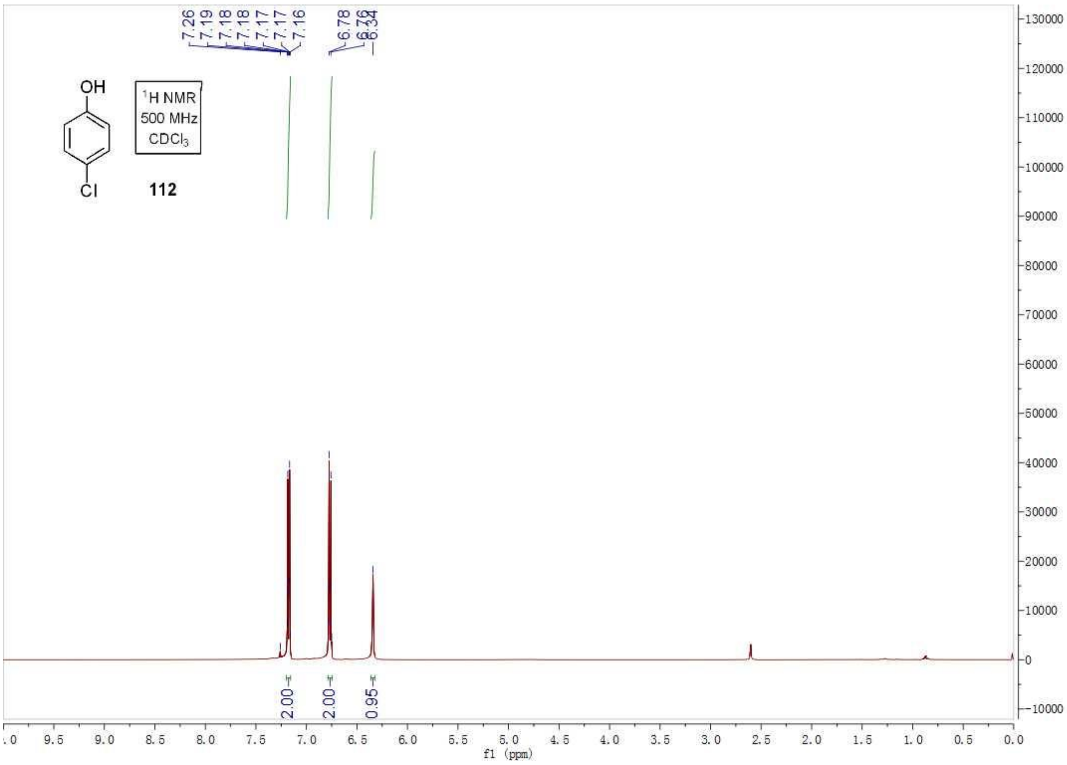

7.26
7.19
7.18
7.18
7.17
7.17
7.16
6.78
6.76
6.34
OH
Cl
^{1}H NMR
500 MHz
$CDCl_3$
112
2.00
2.00
0.95
f1 (ppm)

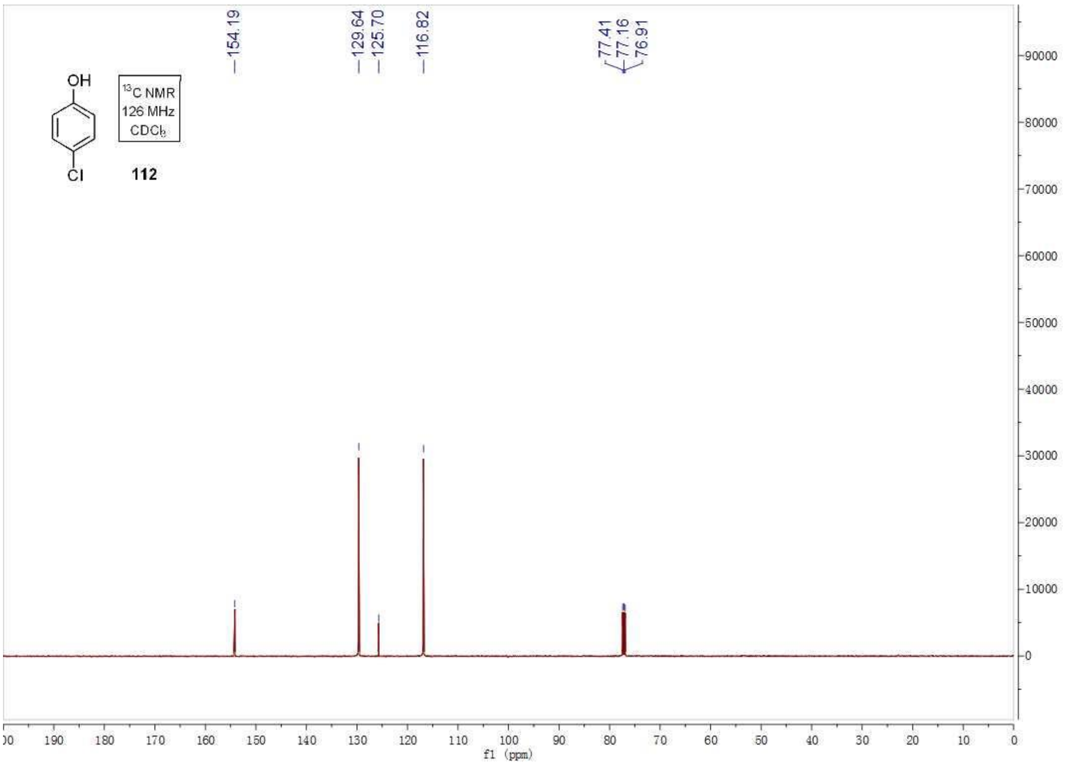

154.19
129.64
125.70
116.82
77.41
77.16
76.91
OH
Cl
^{13}C NMR
126 MHz
$CDCl_3$
112
f1 (ppm)

Supplementary References

1. Ignacio, M.-M. et al. Ascorbic acid as an aryl radical inducer in the gold-mediated arylation of indoles with aryldiazonium chlorides. *Chem. Eur. J.* **26**, 634–642 (2020).
2. MacKenzie, I. K. et al. Discovery and characterization of an acridine radical photoreductant. *Nature* **580**, 76–80 (2020).
3. Bai, J. et al. Radical anion promoted chemoselective cleavage of Csp^2–S bond enables formal cross-coupling of aryl methyl sulfones with alcohols. *Org. Lett.* **23**, 5761–5765 (2021).
4. Cristau, H.-J. et al. A general and mild Ullmann-type synthesis of diaryl ethers. *Org. Lett.* **6**, 913–916 (2004).
5. Wu, W.-B. & Huang, J.-M. Electrochemical cleavage of aryl ethers promoted by sodium borohydride. *J. Org. Chem*. **79**, 10189–10195 (2014).
6. Fischer, S., Ward, T. R. & Liang, A. D. Engineering a metathesis-catalyzing artificial metalloenzyme based on HaloTag. *ACS Catal*. **11**, 6343–6347 (2021).
7. Bering, L., Jeyakumar, K. & Antonchick, A. P. Metal-free C−O bond functionalization: catalytic intramolecular and intermolecular benzylation of arenes. *Org. Lett.* **20**, 3911−3914 (2018).
8. Paia, G. & Chattopadhyay, A. P. *N*-arylation of nitrogen containing heterocycles with aryl halides using copper nanoparticle catalytic system. *Tetrahedron Lett.* **57**, 3140–3145 (2016).
9. Frisch, M. et al. Gaussian 09, Revision E.01 (Gaussian, Inc., Wallingford CT) (2013).
10. Zhao, Y. & Trunhlar, D. The M06 suite of density functionals for main group thermochemistry, thermochemical kinetics, noncovalent interactions, excited states, and transition elements: two new functionals and systematic testing of four M06-class functionals and 12 other functionals. *Theor. Chem. Account*. **120**, 215–241 (2008).
11. Weigend, F. & Ahlrichs. R. Balanced basis sets of split valence, triple zeta valence and quadruple zeta valence quality for H to Rn: design and assessment of accuracy. *Phys. Chem. Chem. Phys.* **7**, 3297–3305 (2005).
12. Marenich, A. V., Cramer, C. J. & Truhlar. D. G. Universal solvation model based on solute electron density and on a continuum model of the solvent defined by the bulk dielectric constant and atomic surface tensions. *J. Phys. Chem. B.* **113**, 6378−6396 (2009).
13. Grimme, S. et al. A consistent and accurate *ab initio* parametrization of density functional dispersioncorrection (DFT-D) for the 94 elements H-Pu. *J. Chem. Phys*. **132**, 154104 (2010).

14. Lu, T. & Chen, F. Multiwfn: a multifunctional wavefunction analyzer. *J. Comput. Chem*. **33**, 580–592 (2012).

15. Petzold, D. et al. Visible-light-mediated synthesis of *β*-chloro ketones from aryl cyclopropanes. *Angew. Chem. Int. Ed.* **58**, 8577–8580 (2019).
